# Autoantibody Landscape Revealed by Wet Protein Array: Sum of Autoantibody Levels Reflects Disease Status

**DOI:** 10.3389/fimmu.2022.893086

**Published:** 2022-05-04

**Authors:** Kazuki M. Matsuda, Ayumi Yoshizaki, Kei Yamaguchi, Eriko Fukuda, Taishi Okumura, Koji Ogawa, Chihiro Ono, Yuta Norimatsu, Hirohito Kotani, Teruyoshi Hisamoto, Ruriko Kawanabe, Ai Kuzumi, Takemichi Fukasawa, Satoshi Ebata, Takuya Miyagawa, Asako Yoshizaki-Ogawa, Naoki Goshima, Shinichi Sato

**Affiliations:** ^1^ Department of Dermatology, The University of Tokyo Graduate School of Medicine, Tokyo, Japan; ^2^ Molecular Profiling Research Center for Drug Discovery, National Institute of Advanced Industrial Science and Technology, Tokyo, Japan; ^3^ ProteoBridge Corporation, Tokyo, Japan; ^4^ Cellular and Molecular Biotechnology Research Institute, National Institute of Advanced Industrial Science and Technology, Tokyo, Japan

**Keywords:** autoantibody, protein array, autoimmunity, inflammatory disease, malignancy

## Abstract

Autoantibodies are found in various pathological conditions such as autoimmune diseases, infectious diseases, and malignant tumors. However their clinical implications have not yet been fully elucidated. Herein, we conducted proteome-wide autoantibody screening and quantification with wet protein arrays consisting of proteins synthesized from proteome-wide human cDNA library (HuPEX) maintaining their three-dimensional structure. A total of 565 autoantibodies were identified from the sera of three representative inflammatory disorders (systemic sclerosis, psoriasis, and cutaneous arteritis). Each autoantibody level either positively or negatively correlated with serum levels of C-reactive protein, the best-recognized indicator of inflammation. In particular, we discovered total levels of a subset of autoantibodies correlates with the severity of clinical symptoms. From the sera of malignant melanoma, 488 autoantibodies were detected. Notably, patients with metastases had increased overall autoantibody production compared to those with tumors limiting to the primary site. Collectively, proteome-wide screening of autoantibodies using the *in vitro* proteome can reveal the “autoantibody landscape” of human subjects and may provide novel clinical biomarkers.

## Introduction

The most primitive role of the immune system is clearance of non-self-antigens derived from external pathogens or toxins (1). Under proper control, differentiation between non-self and self is strictly regulated by the interaction of immune cells in a state of immune tolerance (2). However, the precise regulation sometimes disrupts, and autoimmune response can occur when the structure of foreign antigens resembles that of self-antigens (3), or when co-stimulation signals are enhanced under persistent inflammation (4). One of the most remarkable and sensitive markers of autoimmune reactions is the emergence of autoantibodies in the peripheral blood (5). Hence, the appearance of autoantibodies may reflect the presence of abnormalities in the human body, such as invading foreign pathogens, chronic inflammation, or ongoing tissue destruction.

In fact, autoantibodies are found in various pathological conditions such as autoimmune diseases, infectious diseases, and malignant tumors. Some of them are clinically used as predictors for disease development, and moreover, as barometers that reflect disease activity (6). Nevertheless, such clinical significance has been found for very few autoantibodies, although the Genotype-Tissue Expression project has shown that the transcriptome in human tissues is composed of more than 14,000 protein-coding genes (7). Therefore, an exhaustive approach is desirable to explore the whole picture of association between autoantibodies and clinical symptoms.

In the past several decades, omics-based methodologies have rapidly advanced as powerful tools to describe the “immune landscape” *in vivo* (8, 9). This is mainly due to deciphering of the human genome in 2001 (10, 11), and consequent expansion of genetic bio-information across immune cell types, organs, and diseases through low-cost and high-throughput sequencing technologies (12). In 2008, we launched a proteome-wide human cDNA library named HuPEX (13), and an infrastructure for producing human proteins by Gateway vector system (14) and a wheat germ cell-free system (15–17).

Herein, we employed an updated version of HuPEX that covers approximately 90% of human transcriptome to realize high throughput autoantibody measurement in proteome-wide scale (13). Synthesized human proteins were spotted on glass slides in array format. The arrays were kept in moist conditions during the entire handing process (so-called “wet protein array (WPA)”), which enables displayed antigens to maintain their three-dimensional structure (18). WPAs were reacted with sera from healthy subjects and patients with inflammatory and neoplastic disorders to detect autoantibodies, which provided a new perspective on the “autoantibody landscape” as described below.

## Materials and Methods

### Patients

We recruited Japanese patients with systemic sclerosis (SSc) as a major systemic autoimmune disease, psoriasis (Pso), a systemic inflammatory disease that mainly affects the skin and the joints, and cutaneous arteritis (CA) as an example of single-organ inflammatory diseases that exclusively involves the arterioles in the skin, or malignant melanoma (MM) as a representation of primary cutaneous malignancy. Patients visiting our clinic and for whom sera were available from our sample stock were randomly enrolled. All the SSc patients fulfilled the classification criteria established by the American College of Rheumatology and European League Against Rheumatism in 2013 (19). All the CA patients satisfied the diagnostic criteria of cutaneous polyarteritis nodosa suggested by Nakamura T et al. in 2009 (20). The diagnosis of Pso and MM had been clinically and histologically confirmed by board-certified dermatologists.

In total, 82 patients with SSc, 28 patients with Pso, 24 patients with CA, 40 patients with MM, and 20 healthy controls were recruited. Their demographics are summarized in **Supplementary Table 1**. SSc patients included two patients positive for both anti-centromere antibody (ACA) and anti-RNA polymerase III antibody (RNAP), one patient positive for anti-topoisomerase antibody (ATA) and ACA, one patient positive for ATA and ARA, and one patient positive for ATA, ACA, and RNAP. This study has been approved by The University of Tokyo Ethical Committee (Approval number 695). Written informed consent has been obtained from all the human subjects.

### Clinical Data Acquisition

Clinical data were collected by retrospective review of electric medical records. We gathered basic patient information, symptoms, medications, and laboratory findings from the closest time point from the date of serum collection. SSc patients were categorized by LeRoy’s classification rule into diffuse cutaneous SSc (dcSSc) or limited cutaneous SSc (lcSSc) (21). Skin thickness was semi-quantitatively examined by the modified Rodnan total skin thickness score (mRSS) (22). Interstitial lung disease (ILD) was diagnosed by high-resolution computer tomography as previously described (23). Psoriasis activity and severity index (PASI) was evaluated among Pso patients by board-certified dermatologists (24). The existence of psoriatic arthritis (PsA) was judged based on classification criteria for PsA classification presented by Taylor W et al. in 2006 (25). For CA patients, treatment responders and non-responders were discriminated as previously described (26). Briefly, those requiring intensive treatments such as high dose systemic corticosteroids, intravenous cyclophosphamide pulse therapy, and intravenous immunoglobulin therapy were framed as non-responders. Classification between localized and advanced MM was based on histological examination of the sentinel lymph nodes and/or imaging studies such as fluorodeoxyglucose positron emission tomography.

### *In Vitro* Protein Expression

Proteins were synthesized *in vitro* from 12,946 clones of the HuPEX (**Supplementary Table 2**), a proteome-wide cDNA library, as previously described (13). In brief, entry clones were converted to expression clones using a Gateway LR reaction (Thermo Fisher Scientific). N-terminal FLAG-GST tags were fused to open reading frames (ORFs) of the HuPEX clones using the destination vector pEW-5FG. High-throughput protein synthesis was performed using the wheat germ cell-free synthesis system. WEPRO 7240 G (CellFree Sciences Co., Ltd) was used as a wheat germ extract (13).

### Fabrication of WPA

Expressed proteins were applied onto array-plates by keeping wet condition as described in our previous work (18). Briefly, amino group-modified glass plates (Matsunami glass) were coated with glutathione (GSH), washed, and air dried. Wheat germ cell-free crude solution expressing the target protein harboring FLAG-GST-tag was diluted and spotted onto GSH-coated glass-plates using a HORNET-NX multi-dispense system (Wako Pure Chemical Industries). After spotting, the plates were incubated in blocking buffer, and then stored at -80°C until use.

### Detection of Autoantibody Binding on WPA

The plates were thawed at room temperature, the blocking buffer was discarded, and was treated with serum diluted by 3:1000 in the reaction buffer containing 1x Synthetic block (Invitrogen), phosphate-buffered saline, and 0.1% Tween 20. After the reaction for 1 hour, the plates were washed twice with TBST (#9997; Cell Signaling Technology). Next, the plates were treated with goat anti-human IgG (H+L) Alexa Flour^®^ 647 conjugate (A21445; Thermo Fisher Scientific) diluted in the reaction buffer for 1 hour, washed twice with TBST, washed again under running reverse osmosis water, and air-dried. Finally, the plates were scanned at a 20-µm resolution using a Typhoon FRA 9500 (GE Healthcare) fluorescent imager. The scanned images were saved as 16-bit tiff files. Array-Pro Analyzer ver. 6.3.1 (Media Cybernetics) was used to record the median by drawing an equal-sized circle around the spots. The negative controls were prepared using distilled water instead of mRNA during protein preparation. The positive controls were prepared using mRNA coding IgG for protein synthesis.

### Autoantibody Screening and Quantification

**Figure 1:**
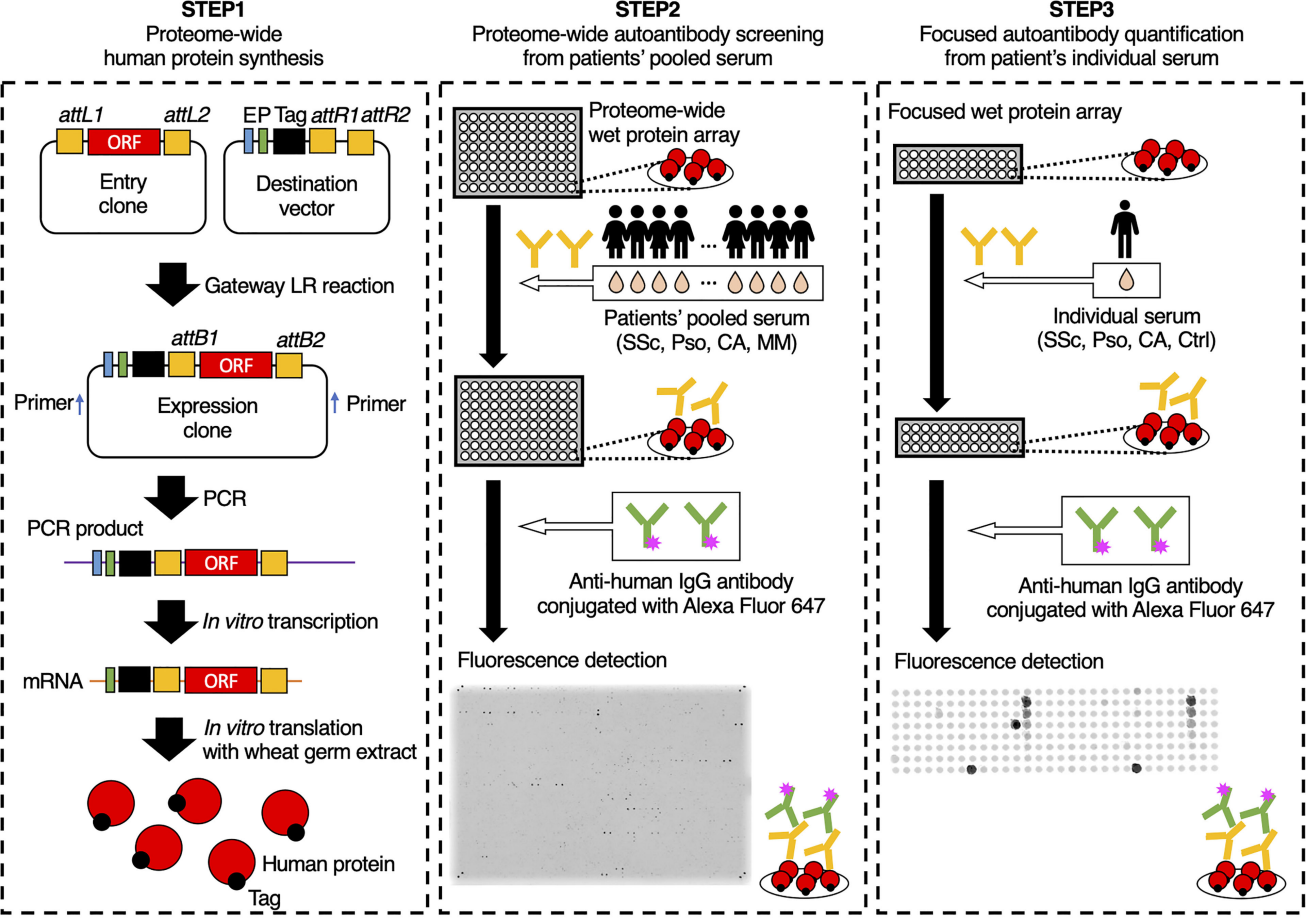
Schematic of the autoantibody screening pipeline. In the first step, proteins were synthesized *in vitro* from the proteome-wide human cDNA library (HuPEX). Promotors (P), Enhancers (E), and FLAG-GST tags were fused to open reading frames of the expression clones by Gateway LR reaction. After polymerase chain reaction amplification and *in vitro* transcription, translation was performed using the wheat germ cell-free synthesis system. In the second step, we conducted autoantibody screening by treating proteome-wide wet protein arrays with pooled serum. In the third step, autoantibody quantification was performed by treating focused wet protein arrays with individual sera. ORF, open reading frame; SSc, systemic sclerosis; Pso, psoriasis; CA, cutaneous arteritis; MM, malignant melanoma; Ctrl, healthy control.

The overview of our workflow is shown in **Figure 1**. As primary selection, proteome-wide autoantibody screening was conducted as below: all the proteins listed in the HuPEX library were synthesized and spotted onto a GSH-coated glass plate. Then the plates were treated with pooled serum from patients with SSc (n = 32), Pso (n = 8), or CA (n = 16). MM sera were divided into either localized group (n = 20) or advanced group (n = 20), pooled respectively, and analyzed as well. Autoantigens whose signal was stronger than that of negative control were regarded to be positive. For secondary selection, positive autoantigens in primary selection were checked by the Cell Atlas for their subcellular spatial distribution (27), and by PubMed for their association with human diseases. Those which express on or outside the cell membrane, or whose association with SSc, Pso, or CA has been previously reported were chosen and mounted on another GSH-coated glass plate. Focused autoantibody quantification was performed by reacting the plate with individual serum from the patients with SSc (n = 82), Pso (n = 28), or CA (n = 24), and the healthy controls (n = 20). The level of each autoantibody was calculated as below:


$$Autoantibody\;titer\;[AU]=\frac{F_{autoantigen}-F_{negative\;control}}{F_{positive\;control\;}-F_{negative\;control}}\times 100$$

*AU*: arbitrary unit

*F _autoantigen_*: fluorescent intensity of autoantigen spot

*F _negative control_*: fluorescent intensity of negative control spot

*F _positive control_*: fluorescent intensity of positive control spot

### Statistical Analysis

Data analysis was performed using Stata 15/IC, GraphPad Prism, R, and a R package “complexheatmap” (28).

## Results

### Proteome-Wide Autoantibody Profiling Revealed Diverse Relationships Between Autoantibody and Clinical Presentation

**Figure 2:**
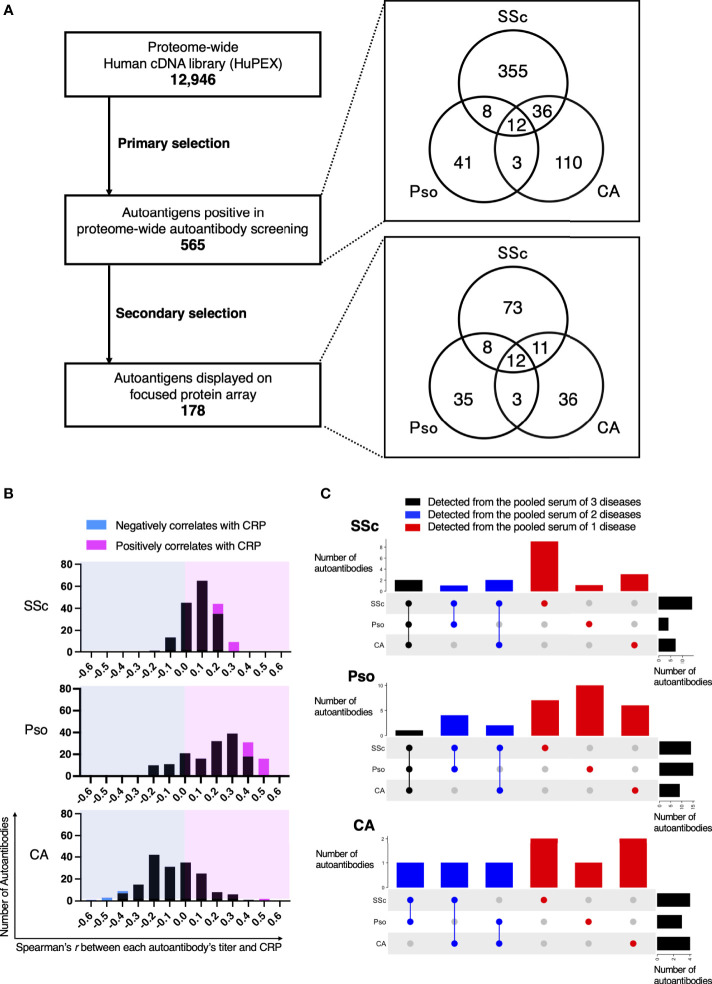
Intra- and Inter-diversity of correlation between serum levels of C-reactive protein and autoantibody level. **(A)** Flow-chart of autoantibody selection. The number of entry cDNA clones was 12,946. As primary selection, proteome-wide autoantibody screening from pooled serum of patients with systemic sclerosis (SSc), psoriasis (Pso), or cutaneous arteritis (CA) detected 565 positivity. Their origin in primary screening is illustrated in a Venn diagram. As secondary selection, a search for association between the diseases by PubMed and for subcellular localization by the Cell Atlas was conducted. Finally, 178 proteins were spotted on the focused protein array. Their origin in primary screening is also shown in a Venn diagram. **(B)** Histograms of Spearman’s correlation coefficient between serum CRP and each autoantibody level in individual sera of patients with SSc, Pso, and CA. Autoantibodies that negatively or positively correlated with serum CRP with statistical significance are highlighted in cyan or in magenta, respectively. **(C)** UpSet plots that visualize the origin of autoantibodies in primary screening that significantly correlates with serum CRP.

We conducted proteome-wide autoantibody profiling for SSc, Pso, and CA. In the primary screening, autoantibodies were measured from pooled sera of these three diseases, resulting in a total of 565 autoantibodies detected. Some autoantibodies were common among multiple disorders, while others were unique to a single disorder (**Figure 2A**). As a secondary selection, we subsequently extracted autoantibodies that have been recognized in the literature as diagnostic or disease activity markers for autoimmune diseases, as well as those targeting proteins that are expressed on the cell membrane or extracellularly. Consequently, 178 autoantigens were selected and plotted on the focused WPA (**Supplementary Figure 1**).

Next, we conducted autoantibody quantification by treating the focused WPA with individual sera of SSc, Pso, and CA, then analyzed relationships between each autoantibody level and clinical manifestations. As a result, the correlation coefficient between each autoantibody level and serum levels of C-reactive protein (CRP), one of the most popular inflammation markers, varied from positive to negative (**Figure 2B**). Autoantibodies whose levels positively correlated with CRP levels with statistical significance were found in all three inflammatory disorders, while those showed significant negative correlation with CRP levels with statistical significance were discovered only in CA.

From which pooled serum each autoantibody whose serum level significantly correlated with serum CRP levels with statistical significance was originally identified in the primary autoantibody screening is described in **Figure 2C**. For instance, there were 18 autoantibodies whose serum levels significantly correlated with serum CRP levels in SSc. Among them, 14 autoantibodies were positive in the primary autoantibody screening from the pooled sera of SSc patients, while one was detected from the pool sera of Pso patients and three were found from the pooled sera of CA.

### Sum of Autoantibody Levels That Correlates With Serum CRP Reflected Clinical Presentation of SSc

**Figure 3:**
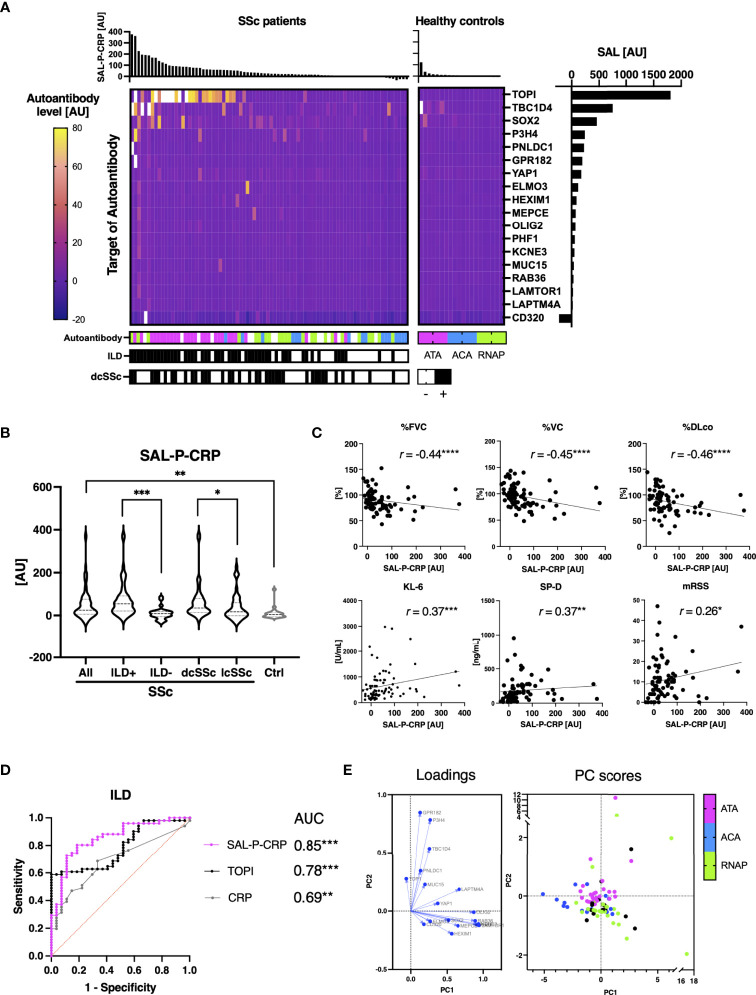
Association between sum of autoantibody levels and clinical presentation in patients with systemic sclerosis. **(A)** Heatmap of 18 autoantibodies that positively correlate with serum levels of C-reactive protein (CRP) with statistical significance in individual sera of systemic sclerosis (SSc). The targets of autoantibodies are described with the gene symbols of their origin. Each case was labeled with the highest serum level among anti-topoisomerase I (ATA), anti-centromere (ACA), and anti-RNA polymerase III (RNAP) antibodies. SAL-P-CRP: sum of autoantibody levels that positively correlated with serum CRP with statistical significance. AU: arbitrary unit. **(B)** Violin plots of SAL-P-CRP in individual sera of SSc patients and healthy controls. Subpopulation analysis revealed that SAL-P-CRP was significantly elevated among SSc patients with interstitial lung disease (ILD) or diffuse cutaneous SSc (dcSSc) compared to limited cutaneous SSc (lcSSc). *P* values were calculated by Mann-Whitney *U* test. **(C)** Correlation between SAL-P-CRP and pulmonary function, serum ILD markers, skin thickness score, and serum CRP levels. *r:* Spearman’s correlation coefficient. *P* values were calculated by estimating *r*. %FVC: percentage of predicted forced lung vital capacity, %DLco: percentage of predicted lung diffusing capacity for carbon monoxide, KL-6: Krebs von den Lungen-6, SP-D: surfactant protein-D, mRSS: modified Rodnan’s total skin thickness score. **(D)** Receiver-operator characteristics curve of accuracy detecting ILD. Area under the curve (AUC) was the biggest for SAL-P-CRP, compared to those for ATA levels (TOP1) or for CRP levels. **(E)** Principal component (PC) analysis. Two-dimensional scattering isolated three representative subpopulations of SSc: ATA, ACA, and RNAP-positive groups. Each case was labeled with the highest serum level among ATA, ACA, and RNAP. PC1, the primary principal component. PC2, the secondary principal component. *P < 0.05, **P < 0.01, ***P < 0.001, ****P < 0.0001.

All the autoantibodies whose serum levels significantly correlated with serum CRP levels are listed in **Figure 3A**. We investigated the relationship between the total level of these 18 autoantibodies (sum of autoantibody levels that positively correlate with serum CRP; SAL-P-CRP) and clinical manifestations of SSc. Consequently, SAL-P-CRP was significantly higher in patients with ILD than in those without ILD (**Figure 3B**). SAL-P-CRP was also higher among dcSSc compared to lcSSc. Furthermore, SAL-P-CRP was significantly correlated with percentage of predicted forced lung vital capacity (%FVC), percentage of predicted lung vital capacity (%VC) and percentage of predicted lung diffusing capacity for carbon monoxide (%DLco) that refer to lung function, serum ILD markers including Krebs von den Lungen-6 (KL-6) and surfactant protein-D (SP-D), skin fibrosis score (modified Rodnan’s total skin thickness score: mRSS), and serum CRP (**Figure 3C**).

Subsequently, ROC curve analysis revealed that area under the ROC curve for ILD prediction of SAL-P-CRP overwhelms that of serum CRP levels, and furthermore, that of ATA level alone (**Figure 3D**). We also conducted a principal component analysis to figure out the low-dimensional features of SAL-P-CRP. As a result, two-dimensional scattering isolated two distinct subpopulation of SSc, ATA-positive group and RNAP-positive group, from the others (**Figure 3E**). Therefore, our proteome-wide autoantibody profiling not only reflects the severity of SSc but also can identify clinical subpopulations of SSc based upon existence of ILD or autoantibody profiles.

**Figure 4:**
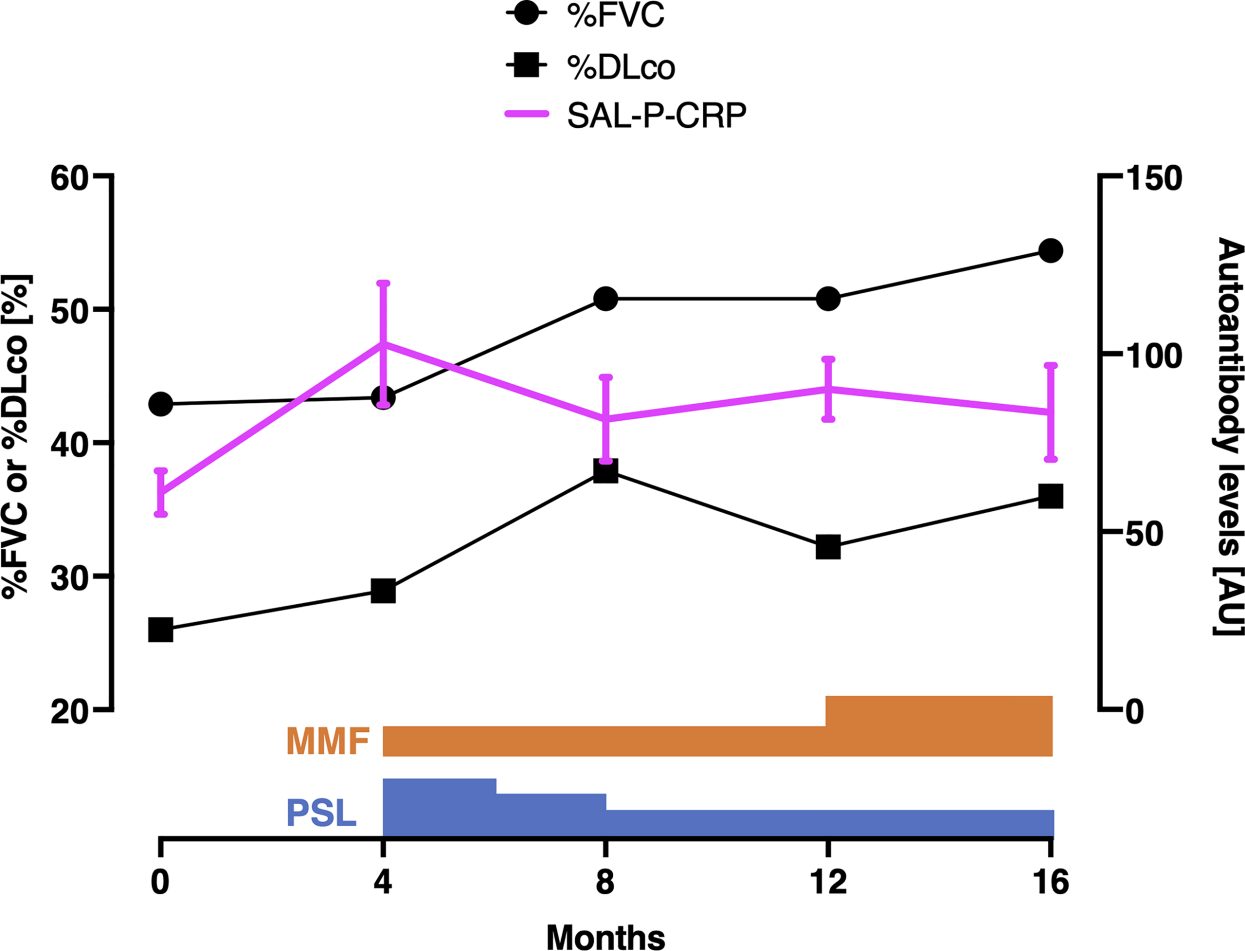
Serial measurements of pulmonary function and autoantibody level in a patient with interstitial lung disease associated with systemic sclerosis. Scales for pulmonary function and autoantibody level are shown at the left and right, respectively. The values of sum of autoantibody levels that positively correlated with serum CRP with statistical significance (SAL-P-CRP) are described as the mean and the standard error of the mean of technical triplicates. %FVC: percentage of predicted forced lung vital capacity, %DLco: percentage of predicted lung diffusing capacity for carbon monoxide, PSL, prednisolone; MMF, mycophenolate mofetil; AU, arbitrary unit.

Indeed, we retrospectively investigated the time course of SAL-P-CRP in an ATA-positive dcSSc patient in whom longitudinal data was available (**Figure 4**). The patient firstly arrived at our clinic presenting severe ILD. During the primary check-up, the patient’s pulmonary function steadily worsened along with SAL-P-CRP increase. We started immunosuppressive therapy by combining oral prednisolone (PSL) 20 mg/day and oral mycophenolate mofetil (MMF) 1 g/day, which resulted in improvement of pulmonary function and SAL-P-CRP decrease. Unfortunately, the patient’s ILD re-exacerbated on the way of PSL tapering, accompanied by SAL-P-CRP re-growth. We raised the dosage of MMF to 2 g/day, which resulted in stabilization of both pulmonary function and SAL-P-CRP. Thus, the serial changes in SAL-P-CRP appeared to be consistent with the clinical course of SSc-related ILD.

**Figure 5:**
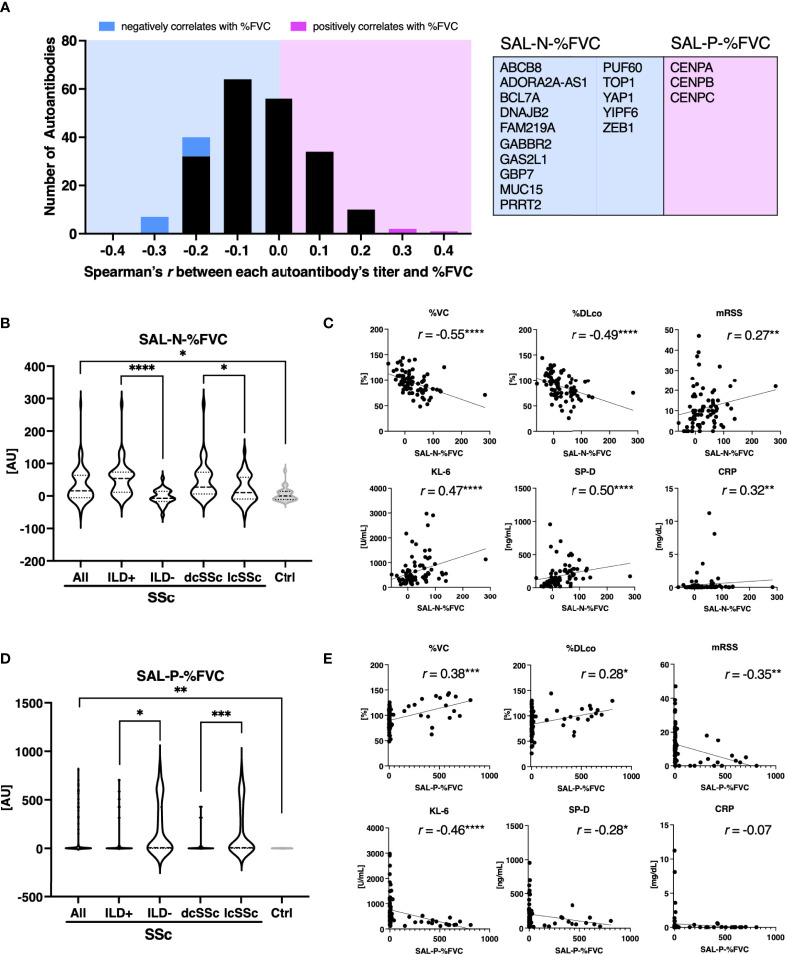
Association between sum of autoantibody levels that correlates with forced lung vital capacity and clinical presentation in patients with systemic sclerosis. **(A)** Histograms of Spearman’s correlation coefficient between percentage of predicted forced lung vital capacity (%FVC) and each autoantibody level in individual sera of patients with systemic sclerosis (SSc). Autoantibodies that negatively or positively correlated with %FVC with statistical significance are highlighted in cyan or in magenta, respectively. Their targets are listed in the table with the gene symbols of their origin. AU, arbitrary unit. **(B)** Violin plots of sum of autoantibody levels that negatively correlated with %FVC with statistical significance (SAL-N-%FVC) in SSc patients and healthy controls. Subpopulation analysis revealed that SAL-N-%FVC was significantly elevated among SSc patients with interstitial lung disease (ILD), or diffuse cutaneous SSc (dcSSc) compared to limited cutaneous SSc (lcSSc). *P* values were calculated by Mann-Whitney *U* test. **(C)** Correlation between SAL-N-%FVC and pulmonary function, serum ILD markers, skin thickness score, and serum CRP levels. *r:* Spearman’s correlation coefficient. *P* values were calculated by estimating *r*. %DLco: percentage of predicted lung diffusing capacity for carbon monoxide, KL-6: Krebs von den Lungen-6, SP-D: surfactant protein-D, mRSS: modified Rodnan’s total skin thickness score. **(D)** Violin plots of sum of autoantibody levels that positively correlated with %FVC with statistical significance (SAL-P-%FVC) in SSc patients and healthy controls. Subpopulation analysis revealed that SAL-P-%FVC was significantly lower among SSc patients with ILD, or dcSSc compared to lcSSc. *P* values were calculated by Mann-Whitney *U* test. **(E)** Correlation between SAL-P-%FVC and pulmonary function, serum ILD markers, skin thickness score, and serum CRP levels. *P < 0.05, **P < 0.01, ***P < 0.001, ****P < 0.0001.

Similarly, we examined the relationship between SSc characteristics and the sum of autoantibody levels which significantly correlated with %FVC, which is the most widely-used measure of SSc-associated ILD (29). There were fifteen autoantibodies, including ATA, that negatively correlated with %FVC with statistical significance, whereas three autoantibodies, all of which targets the components of the centromere, positively correlated with %FVC (**Figure 5A**). Sum of autoantibody levels that negatively correlates with %FVC (SAL-N-%FVC) was significantly associated with the presence of ILD, dcSSc rather than lcSSc, lower %VC, lower %DLco, higher KL-6, higher SP-D, higher mRSS, and higher CRP (**Figures 5B, C** **)**. Conversely, total autoantibody level that were positively correlated with %FVC (SAL-P-%FVC) were associated with the absence of ILD, lcSSc rather than dcSSc, higher %VC, higher %DLco, lower mRSS, lower KL-6, and lower SP-D (**Figures 5D, E** **)**. These results suggest that some of the autoantibodies found by proteome-wide autoantibody profiling are indicators of severe SSc-ILD and diffuse skin sclerosis, while others reflect a favorable status of the disease.

### Sum of Autoantibody Levels That Correlates With Serum CRP Reflects Clinical Features in Pso

**Figure 6:**
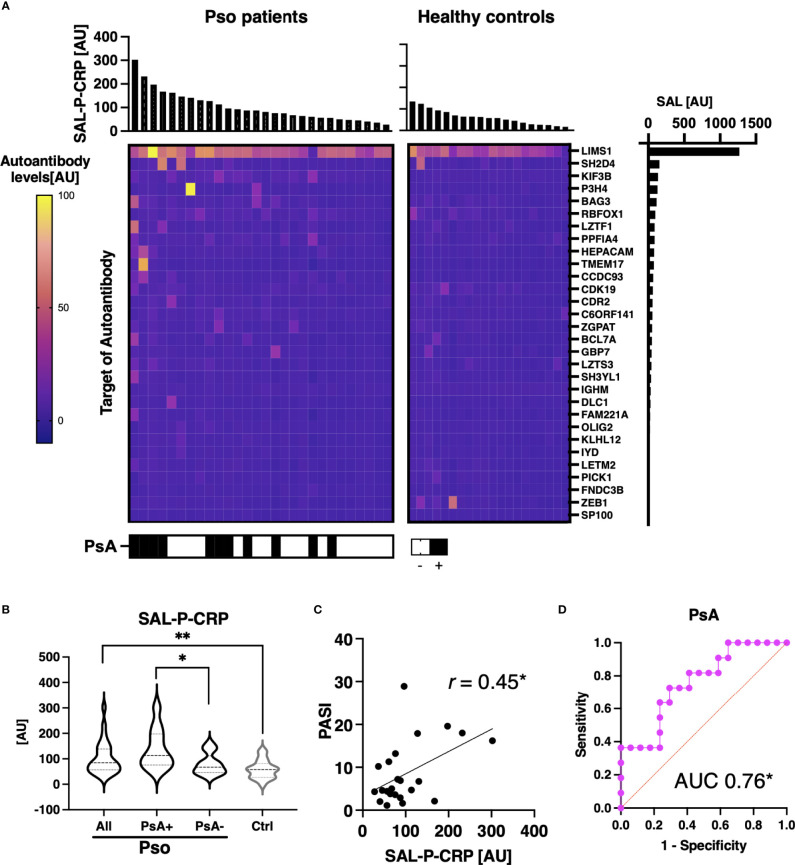
Association between sum of autoantibody levels and clinical presentation in psoriasis patients. **(A)** Heatmap of 30 autoantibodies that positively correlate with serum levels of CRP with statistical significance in individual sera of psoriasis (Pso). The targets of autoantibodies are described with the gene symbols of their origin. SAL-P-CRP: sum of autoantibody levels that positively correlated with serum C-reactive protein (CRP) levels. AU: arbitrary unit. **(B)** Violin plots of SAL-P-CRP in Pso patients and healthy controls. Subpopulation analysis revealed that SAL-P-CRP was significantly elevated among patients with psoriatic arthritis (PsA) compared to the others. *P* values were calculated by Mann-Whitney *U* test. **(C)** Correlation between SAL-P-CRP and psoriasis area and severity index (PASI) score, or serum CRP levels. *r:* Spearman’s correlation coefficient. *P* values were calculated by estimating *r*. **(D)** Receiver-operator characteristics curve of accuracy predicting PsA. Area under the curve (AUC) was 0.76 (95% confidence interval: 0.58-0.94). *P < 0.05, **P < 0.01.

We additionally investigated the association between total levels of autoantibodies that correlated with serum CRP and clinical manifestations of Pso. Among Pso patients, 30 autoantibodies were significantly positively correlated with serum CRP (**Figure 6A**). SAL-P-CRP, calculated from the level of the selected antibodies, was significantly higher among patients with psoriatic arthritis (PsA), which is recognized as a condition with strong autoimmune abnormalities among Pso (**Figure 6B**) (30). In addition, there was a significant correlation between SAL-P-CRP and the psoriasis area severity index (PASI), an indicator of skin symptoms (**Figure 6C**) (24). Of note, ROC curve analysis showed that SAL-P-CRP predicts the presence of PsA with area under the ROC curve at 0.76 (95% confident interval: 0.58-0.94) (**Figure 6D**).

**Figure 7:**
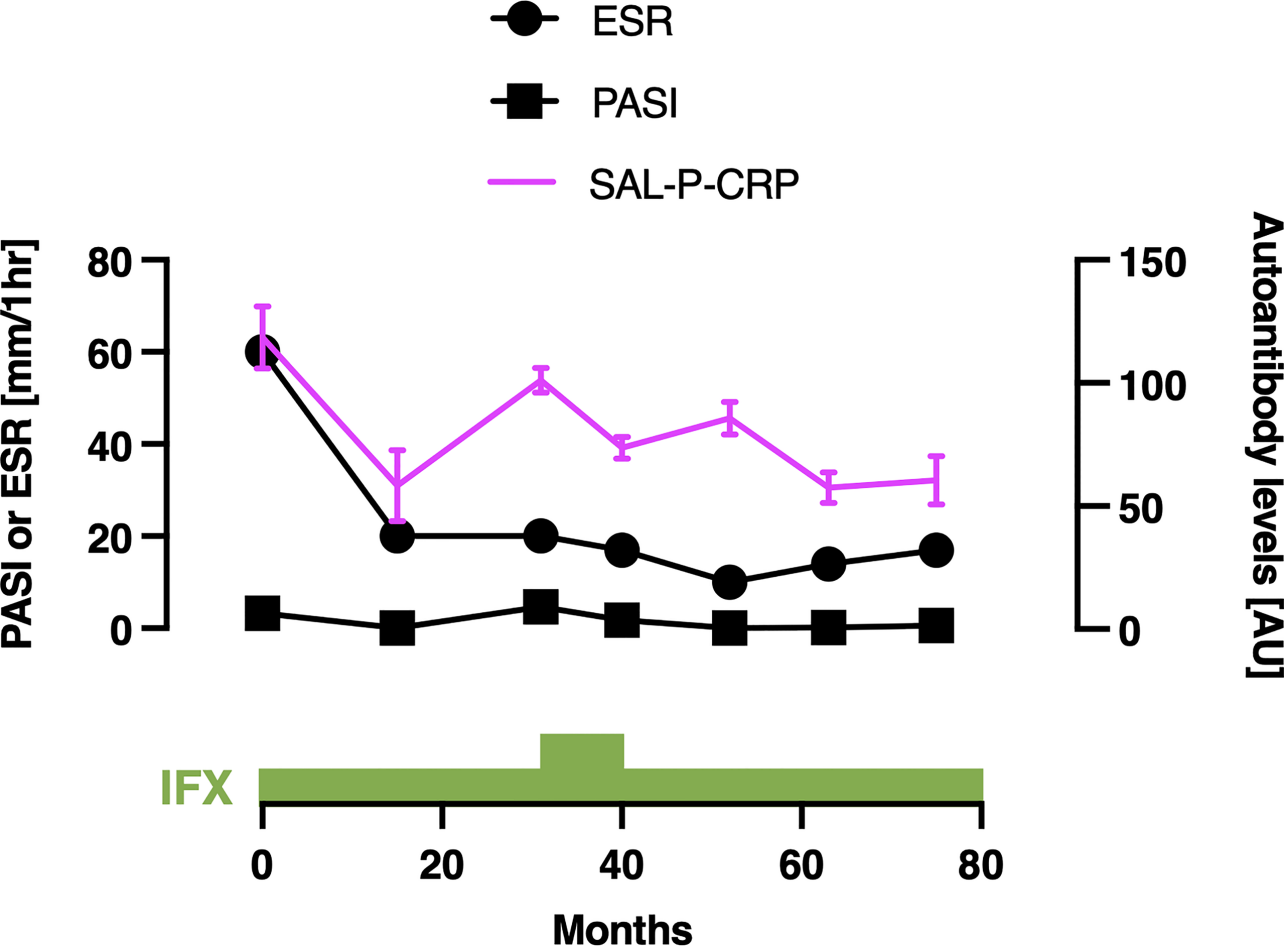
Serial measurements of pulmonary function and autoantibody level in a patient with psoriatic arthritis. Scales for psoriasis area and severity index (PASI) and erythrocyte sedimentation rate (ESR) are shown on the left, and the scale for sum of autoantibody levels that positively correlated with serum C-reactive protein levels (SAL-P-CRP) is shown on the right. The values of SAL-P-CRP are described as the mean and the standard error of the mean of technical triplicates. IFX, infliximab; AU, arbitrary unit.

We also acquired the serial data of SAL-P-CRP in a patient with PsA (**Figure 7**). He received infliximab (IFX) 5 mg/kg per 8 weeks for his severe skin eruption and arthritis. The treatment significantly resolved his cutaneous and joint symptoms, along with reduction of PASI, erythrocyte sedimentation rate (ESR) and SAL-P-CRP. However, two and a half years later his symptoms exacerbated, which led the physicians to increase the dosage of IFX to 10 mg/day per 8 weeks for 10 months. Since then, his PASI, ESR, and SAL-P-CRP remained stable under maintenance therapy with IFX 5 mg/day per 8 weeks. As shown, SAL-P-CRP longitudinally correlated with the clinical course not only in SSc but also in a case of PsA.

### Sum of Autoantibody Levels That Correlates With Serum CRP Is Associated With Clinical Status of CA

**Figure 8:**
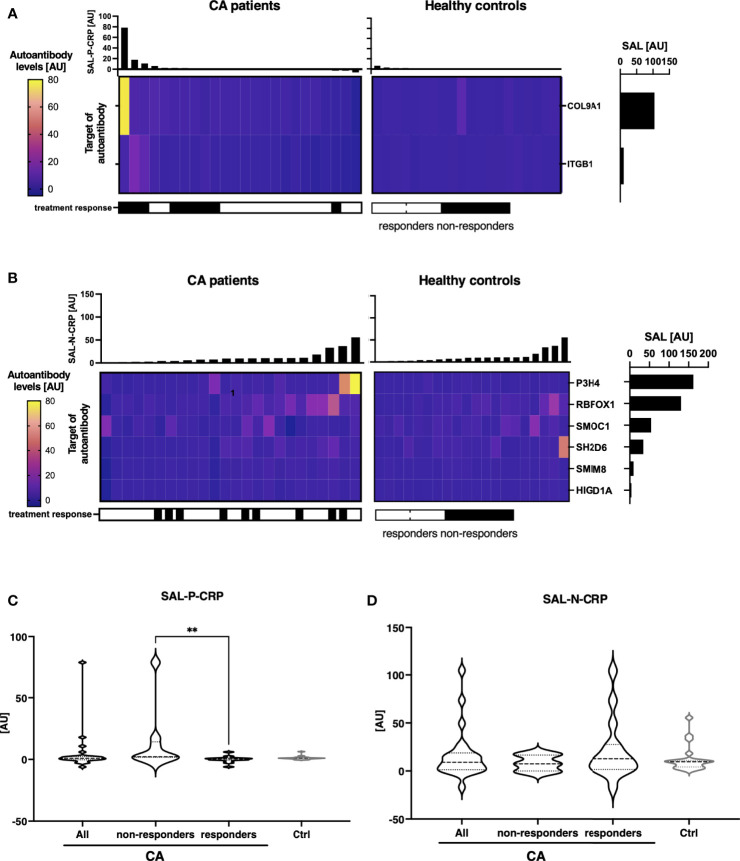
Association between sum of autoantibody levels and clinical presentation in cutaneous arteritis patients. **(A)** Heatmap of 2 autoantibodies that positively correlate with serum levels of C-reactive protein (CRP) with statistical significance in individual sera of cutaneous arteritis (CA). The targets of autoantibodies are described with the gene symbols of their origin. SAL-P-CRP: sum of autoantibody levels that positively correlated with serum CRP levels with statistical significance. AU, arbitrary unit. **(B)** Heatmap of 6 autoantibodies that negatively correlate with serum CRP with statistical significance in individual sera of CA. The targets of autoantibodies are described with the gene symbols of their origin. SAL-N-CRP: sum of autoantibody levels that negatively correlates with serum CRP levels with statistical significance. **(C)** Violin plots of SAL-P-CRP in CA patients and healthy controls. Subpopulation analysis revealed that SAL-P-CRP was significantly elevated among treatment non-responders than among responders. *P* values were calculated by Mann-Whitney *U* test. **(D)** Violin plots of SAL-N-CRP in CA patients and healthy controls. **P < 0.01.

In CA, the numbers of autoantibodies that positively or negatively correlated between serum CRP with statistical significance were 2 and 6, respectively (**Figures 8A, B** **)**. SAL-P-CRP was significantly higher among treatment non-responders than among the others (**Figure 8C** **)**. In contrast, the total autoantibody level which was negatively correlated with serum CRP with statistical significance (SAL-N-CRP), was relatively low in treatment non-responders than in responders, although not statistically significant (**Figure 8D**). These results indicate that proteome-wide autoantibody profiling may be useful not only in SSc, but also in other inflammatory diseases such as Pso and CA for predicting disease activity and even treatment response.

### Autoantibody Production Was Enhanced in Advanced MM Than in Localized MM

**Figure 9:**
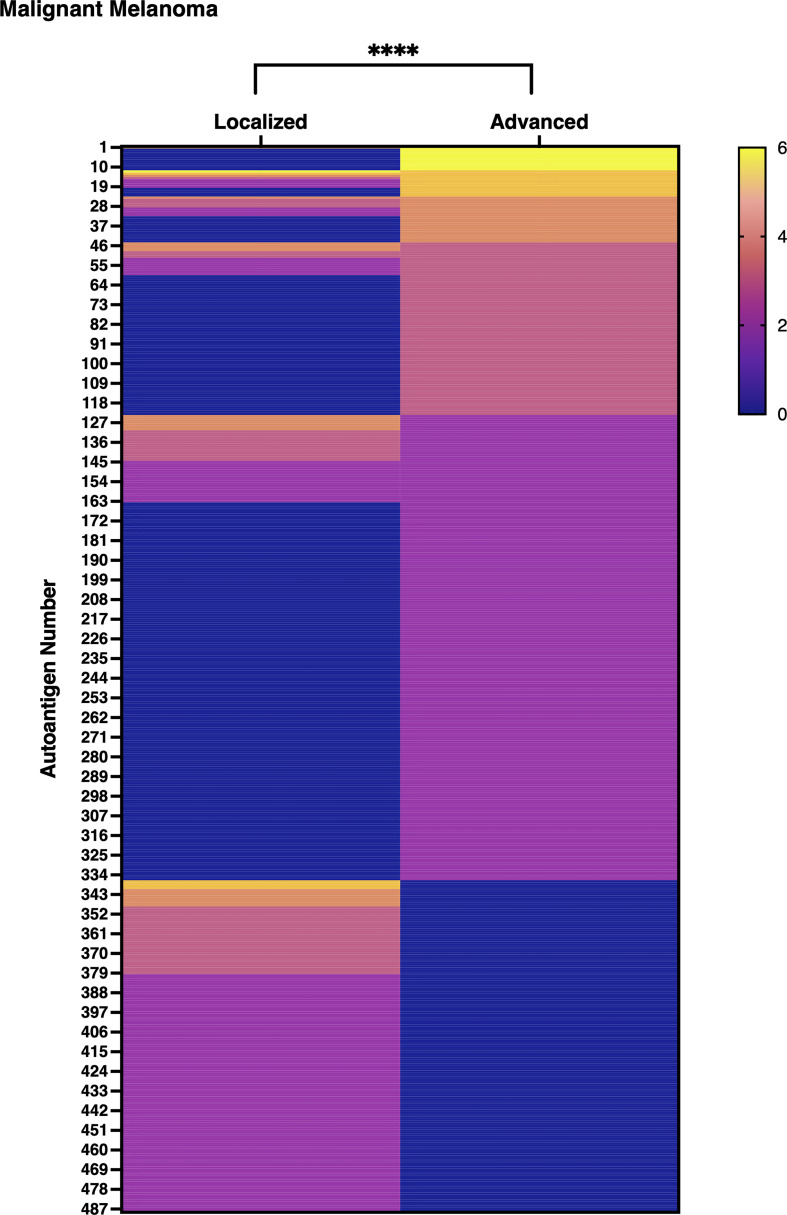
Gross autoantibody product in localized and advanced malignant melanoma. The result of proteome-wide autoantibody detection from pooled serum of localized or advanced malignant melanoma (MM) patients is illustrated. Each row shows autoantibodies spotted on the focused wet protein array (WPA) and their signal strength on the proteome-wide WPA. Semi-quantification of signals on proteome-wide WPA was conducted as below: higher than positive control’s signal strength: 6, higher than 1/2 positive control’s signal strength but lower than positive control’s signal strength: 5, higher than 1/4 but lower than 1/2 of positive control’s signal strength: 4, higher than 1/8 but lower than 1/4 of positive control’s signal strength: 3, higher than 1/16 but lower than 1/8 of positive control’s signal strength: 2, higher than negative control’s signal strength but lower than 1/16 of positive control’s signal strength: 1. P value was calculated by Wilcoxon signed-rank test. ****P < 0.0001.

An exhaustive autoantibody screening using sera obtained from patients with MM detected 488 autoantibodies in total. Of note, the number of autoantibodies in the advanced group (n = 336) overwhelmed that in the localized group (n =225, **Supplementary Table 3**). Moreover, the gross production of autoantibodies was significantly higher in advanced MM than in localized MM with statistical significance (**Figure 9**). These results strengthen our hypothesis that detection of autoantibodies by proteome-wide protein array would be useful in evaluating disease severity or extension, regardless of the type of disease.

## Discussion

In the present study, proteome-wide autoantibody profiling utilizing the *in vitro* human proteome demonstrated inter- and intra-disease variability in the relationship between autoantibodies and clinical symptoms. Namely, the correlation between serum levels of inflammation markers and the level of each autoantibody varied from positive to negative, and the list of autoantibodies that significantly correlated with serum inflammation marker differed from disease to disease. Meanwhile, the sum of appropriately selected autoantibody levels for each disease sensitively reflected disease activity, treatment response, and disease progression. These findings suggest that observation of the “autoantibody landscape” by proteome-wide autoantibody profiling illustrates inter- and intra-disease diversity of relationship between autoantibodies and disease status and might provide an innovative disease assessment tool.

ATA is a well-known indicator of diffuse skin sclerosis and severe ILD among SSc patients (31). Therefore, the fact that ATA was included in the list of autoantibodies whose serum levels positively correlates with serum CRP levels supports the validity of our analysis. Moreover, ROC curve analysis demonstrated that SAL-P-CRP, which contains ATA as its component, was superior to ATA alone in terms of detecting ILD. While the ROC curve of SAL-P-CRP convex upward, that of ATA convex downward. It suggests that ATA could not balance high sensitivity and specificity, but SAL-P-CRP could achieve both high sensitivity and specificity for ILD detection. Presence of ILD has significant implications in clinical practice of SSc, because ILD is the leading cause of disease-related death (32). Although ATA is a good clinical indicator of ILD, absence of ATA is not always equal to absence of ILD. We hypothesize that SAL-P-CRP overwhelmed ATA alone by ILD detection because it could perceive ILD among ATA-negative cases.

In the present study, we adopted serum CRP levels as a touchstone to screen autoantibodies that can be applied to all three inflammatory diseases. However, this scheme might not be the best way to pick up clinically significant autoantibodies, because serum CRP level does not always reflect disease status of some disorders, including SSc. This is the reason why we tried %FVC as another marker to select autoantibodies, which succeeded in discovering autoantibodies whose serum levels either positively or negatively associates with disease status. Although both SAL-P-CRP and SAL-N-%FVC significantly reflected clinical features of SSc, autoantibodies selected by two different methods were not identical. The way how to combine multiple autoantibodies into a single composite measure should be further investigated and be optimized for each disease in the future studies. In addition, it should be noted that negative correlation between serum levels of ACA and disease status might be attributed to confounding effect due to mutual exclusion between ACA and other SSc-specific autoantibodies such as ATA.

The major limitations of the present study include its indistinct patient selection criteria and lack of validation in an independent cohort. Further investigation, ideally with prospective recruitment of consecutive cases among multiple clinics and ethnicities, is required to confirm the findings. In addition, our scheme for autoantibody screening might overlook some autoantibodies whose serum levels are low, but significantly correlate with disease status. This suspicion derives from the fact that some of autoantibodies we found that their serum levels significantly correlate with serum CRP were discovered from pooled sera of different disorder. Primary screening with pooled sera might not be the best way for comprehensive autoantibody evaluation, which should be further validated.

Autoantibodies are found in various pathological conditions, but the significance of autoantibodies has not yet been fully elucidated. In organ-specific autoimmune diseases such as Graves’ disease and autoimmune bullous disease, autoantibodies have been shown to have distinct pathogenic properties (33). Meanwhile, most autoantibodies that appear in systemic autoimmune diseases such as SSc and systemic lupus erythematosus (SLE) recognize nuclear antigens, and the mainstream view is that these autoantibodies are not pathogenic because they cannot access the nucleus through the cell membrane *in vivo*. Furthermore, autoantibodies targeting the same protein may have a paradoxical impact on different conditions. For instance, autoantibody targeting type 1 interferon is associated with mild disease activity in SLE (34), in line with success of the trial of humanized monoclonal antibody (35). In contrast, the presence of anti-type 1 interferon autoantibodies are associated with worse prognosis of coronavirus infection 2019 (36). This may be due to inhibition of antiviral immunity by the neutralization of type 1 interferon.

To fully understand the complex relationship between autoantibodies and disease status, proteome-wide quantification of autoantibodies from individual sera, especially for MM, should be ideally performed in the future investigation. Our WPA has many advantages compared to previously developed human protein arrays or extramembrane antigen displays (37–39). First, the coverage of autoantigens within the human proteome is almost at the level of the whole-proteome-wide. Second, the wheat germ cell-free expression system can produce wider range of proteins including extracellular or membranous proteins, compared to conventional systems using prokaryotes or cultured cells (13). Third, handling the arrays under moist conditions during the entire process enables the displayed proteins to maintain their three-dimensional structure (18). Fourth, the procedures required for autoantibody measurement is relatively simple, which is important to apply them to clinical practice. Our technology will provide big data that illustrates “autoantibody landscape” *in vivo*, which would be useful in developing novel clinical biomarkers as indicated in the present study. In an ongoing project, we are working to develop a database of autoantibody profiles for various immune-related diseases. Our final goal is to build a comprehensive database of autoantibody profiles for a variety of immune-related diseases.

## Data Availability Statement

The raw data supporting the conclusions of this article is not readily available because of participant privacy. The datasets are available from the corresponding authors upon reasonable request.

## Ethics Statement

The studies involving human participants were reviewed and approved by The University of Tokyo Hospital Ethical Committee. The patients/participants provided their written informed consent to participate in this study. Written informed consent was obtained from the individual(s) for the publication of any potentially identifiable images or data included in this article.

## Author Contributions

Conceptualization: AY, SS; Methodology: KM, AY, KY, EF, TO, KO, NG; Investigation: KM, YN, KY, CO; Clinical data acquisition: HK, TH, RK, AK, TF, SE, TM, AY-O; Project administration: AY; Supervision: AY; Writing – original draft: KM, AY; Writing – review and editing: EF, NG, SS. All authors contributed to the article and approved the submitted version.

## Conflict of Interest

Authors KY, TO, KO, CO and NG were employed by ProteoBridge Corporation.

The remaining authors declare that the research was conducted in the absence of any commercial or financial relationships that could be construed as a potential conflict of interest.

## Publisher’s Note

All claims expressed in this article are solely those of the authors and do not necessarily represent those of their affiliated organizations, or those of the publisher, the editors and the reviewers. Any product that may be evaluated in this article, or claim that may be made by its manufacturer, is not guaranteed or endorsed by the publisher.

## Supplementary Material

Supplementary Figure 1 Autoantigens displayed on the focused wet protein array. Each row represents the autoantigens displayed on the focused wet protein array (WPA) and their signal strength on the proteome-wide WPA. Autoantigens are described with the gene symbols of their origin. Semi-quantification of signals by proteome-wide WPA was conducted as below: higher than 1/4 but lower than 1/2 of positive control’s signal strength: 4, higher than 1/8 but lower than 1/4 of positive control’s signal strength: 3, higher than 1/16 but lower than 1/8 of positive control’s signal strength: 2, higher than negative control’s signal strength but lower than 1/16 of positive control’s signal strength: 1. SSc: systemic sclerosis, Pso: psoriasis, CA: cutaneous arteritis.
