# Supplementary figures and images for: Autoantibody Landscape Revealed by Wet Protein Array: Sum of Autoantibody Levels Reflects Disease Status
Source: Front Immunol. 2022 May 4;13:893086. doi: 10.3389/fimmu.2022.893086 (PMC9114879; doi:10.3389/fimmu.2022.893086)

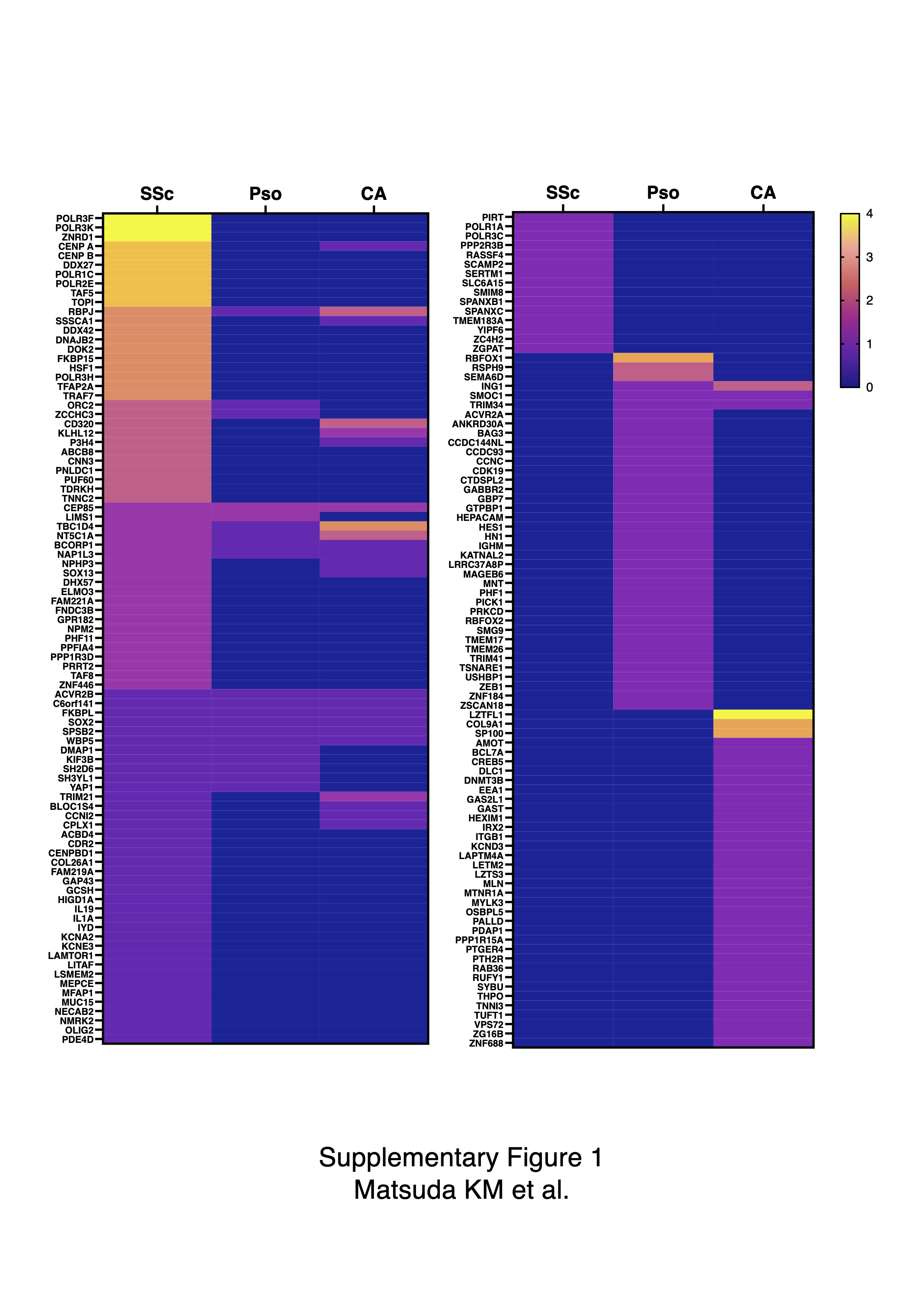

Supplement: Supplementary Figure 1 — Autoantigens displayed on the focused wet protein array. Each row represents the autoantigens displayed on the focused wet protein array (WPA) and their signal strength on the proteome-wide WPA. Autoantigens are described with the gene symbols of their origin. Semi-quantification of signals by proteome-wide WPA was conducted as below: higher than 1/4 but lower than 1/2 of positive control’s signal strength: 4, higher than 1/8 but lower than 1/4 of positive control’s signal strength: 3, higher than 1/16 but lower than 1/8 of positive control’s signal strength: 2, higher than negative control’s signal strength but lower than 1/16 of positive control’s signal strength: 1. SSc: systemic sclerosis, Pso: psoriasis, CA: cutaneous arteritis. [file Image_1.jpg]
