# Supplementary material for: Autoantibody Landscape Revealed by Wet Protein Array: Sum of Autoantibody Levels Reflects Disease Status
Source: Front Immunol. 2022 May 4;13:893086. doi: 10.3389/fimmu.2022.893086 (PMC9114879; doi:10.3389/fimmu.2022.893086)
Supplement: Supplementary file 2 [file Table_1.pdf]

Supplementary Table 1. Demographics of human subjects.

	SSc (n = 82)
Male/Female	15/67
Age, years	57.3 (14.5)
Disease duration, years	2.5 (2.9)
Disease-specific autoantibodies*	
Anti-Topoisomerase I antibody	27 (32.9)
Anti-Centromere antibody	22 (26.8)
Anti-RNA polymerase III antibody	24 (29.3)
Unknown	15 (18.3)
dcSSc	38 (46.3)
mRSS, score	11.3 (9.8)
ILD	51 (63.8)
%FVC, %	89.6 (20.0)
%VC, %	95.0 (20.9)
%DLco, %	87.4 (23.2)
KL-6, U/mL	652 (595)
SP-D, ng/mL	185 (170)
CRP, mg/dL	0.4 (1.6)
	**Pso (n = 28)**
Male/Female	9/19
Age, years	54.9 (14.5)
Disease duration, years	14.2 (11.0)
PsA	11 (39.3)
PASI, score	8.5 (7.2)
CRP, mg/dL	1.4 (5.7)
	**CA (n = 24)**
Male/Female	5/19
Age, years	47.1 (18.2)
Disease duration, years	0.5 (0.8)
Treatment non-responders	9 (37.5)
CRP, mg/dL	1.2 (2.2)
	**MM (n = 40)**
Male/Female	22/18
Age, years	64.2 (15.1)
Clinical stage	
Localized	20 (50)
Advanced	20 (50)
	**Healthy controls (n = 20)**
Male/Female	5/15
Age, years	50.7 (16.8)

Data are n (%) or mean (standard deviation).

* Including two patients positive for both Anti-centromere and anti-RNA polymerase IIII antibodies, one patient positive for anti-topoisomerase I and anti-centoromere antibodies, one patient positive for anti-topoisomerase I and anti-RNA polymerase III antibodies, and one patient positive for anti-topoisomerase I, anti-centromere, and anti-RNA polymerase III antibodies.
