# Supplementary material for: Autoantibody Landscape Revealed by Wet Protein Array: Sum of Autoantibody Levels Reflects Disease Status
Source: Front Immunol. 2022 May 4;13:893086. doi: 10.3389/fimmu.2022.893086 (PMC9114879; doi:10.3389/fimmu.2022.893086)
Supplement: Supplementary file 3 [file Table_2.pdf]

Supplementary Table 2. Features of all cDNA clones used for protein expression.

Clone ID	ORF size (number of amino acid residues)	Molecular weight (kDa)	Symbol
FLJ01013AAAF	465	52.26	[SMAD1]
FLJ01019AAAF	527	60.12	[SGOL1]
FLJ01021AAAF	123	13.77	[PSMG4]
FLJ01022AAAF	412	44.56	[FKBP8]
FLJ01025SAAF	904	101.9	[MCM2]
FLJ01026AAAF	1338	137.33	[IRS2]
FLJ01030AAAF	753	84.15	[PCSK1]
FLJ01031AAAF	1609	182.06	[PIK3C2B]
FLJ01032AAAN	728	80.01	[SKI]
FLJ01033AAAN	418	46.26	[NTSR1]
FLJ01035AAAN	412	45.34	[MLNR]
FLJ01038AAAN	1132	127	[TERT]
FLJ02701AAAN	327	36.31	[PPM1B]
FLJ02809SAAF	869	98.15	[EIF4G2]
FLJ02812AAAF	1255	137.91	[ERBB2]
FLJ02813AAAF	301	32.82	[MED8]
FLJ02814AAAN	594	67.23	[GRB10]
FLJ02816AAAN	206	23.87	[PTPN20]
FLJ02821AAAN	400	46.11	[TRIM21(1-400)]
FLJ02901AAAN	1586	167.78	[GLI2]
FLJ03001AAAF	601	66.21	[PPP2R1B]
FLJ03003AAAF	524	61.06	[PPP2R5C]
FLJ03007AAAN	33	3.5	[VENTXP1]
FLJ03008AAAN	72	8.26	[SPANXN1]
FLJ03009AAAN	99	11.17	[SPANXN4]
FLJ03010AAAN	72	8.28	[SPANXN5]
FLJ03011AAAN	108	12.08	[XAGE5]
FLJ03012AAAN	113	12.48	[PAGE3]
FLJ03014AAAN	141	15.47	[CALCA]
FLJ03015AAAN	153	17.11	[GIP]
FLJ03017AAAN	89	9.81	[IAPP]
FLJ03018AAAN	153	17.73	[NMS]
FLJ03021AAAN	241	26	[TNFRSF18]
FLJ03025AAAN	255	28.3	[MYF5]
FLJ04001AAAF	862	95.63	[AXIN1]
FLJ04002AAAF	1150	132.71	[CCAR1]
FLJ04003AAAF	549	61.19	[PIP5K1A]
FLJ04005AAAF	1084	119.04	[HDAC4]
FLJ04006AAAF	825	88.69	[NFATC1]
FLJ04007AAAF	467	50.36	[ADHFE1]
FLJ04008AAAF	729	81.1	[DCLK1]
FLJ04009AAAF	2109	238.84	[GTF3C1]
FLJ04012AAAF	894	95.37	[ILF3]
FLJ04013AAAF	1191	131.67	[RC3H2]
FLJ04015AAAF	2376	266.94	[CNOT1]

FLJ04016AAAF	951	104.38	[CNNM1]
FLJ04017AAAF	570	62.69	[PANK2]
FLJ04019AAAF	641	73.45	[THEMIS]
FLJ04020AAAF	386	42.96	[TIA1]
FLJ04024AAAN	2696	296.62	[NSD1]
FLJ04025AAAF	500	55.55	[TERF2]
FLJ04026AAAN	1034	117.21	[SLTM]
FLJ04027AAAF	637	69.59	[NOP9]
FLJ04028AAAN	1495	171.26	[ZCCHC6]
FLJ04029AAAF	851	94.07	[DDX31]
FLJ04031AAAF	339	35.61	[AUH]
FLJ04032AAAF	825	90.58	[HNRNPU]
FLJ04033AAAF	465	50.55	[CELF3]
FLJ04034AAAF	881	98.59	[DDX54]
FLJ04035AAAF	1645	185.24	[ZCCHC11]
FLJ04036AAAF	478	53.05	[METTL17]
FLJ04037AAAN	1792	201.55	[RBBP6]
FLJ04038AAAF	660	76.75	[FAM161A]
FLJ04039AAAN	1335	149.04	[RPTOR]
FLJ04040SAAF	633	70.02	[PML]
FLJ04042AAAF	2177	243.07	[MED12]
FLJ04044AAAF	523	58.74	[PSPC1]
FLJ04045AAAF	938	105.37	[GRIN1]
FLJ04046AAAF	1133	125.74	[RC3H1]
FLJ04047AAAN	759	85	[BRCA1]
FLJ04051AAAF	1580	169.86	[GLI3]
FLJ04053AAAN	660	69.06	[FOXK2]
FLJ04054AAAN	516	55.64	[IRF7]
FLJ04056AAAF	505	53.68	[FOXO4]
FLJ04057AAAF	733	75.46	[FOXK1]
FLJ04058AAAF	690	79.07	[ARHGEF4]
FLJ04059AAAF	984	107.08	[NR3C2]
FLJ04060AAAF	788	86.75	[MED15]
FLJ04061AAAF	514	56.45	[PPM1H]
FLJ04062AAAF	747	81.68	[SIRT1]
FLJ04063AAAF	2677	302.88	[SETX]
FLJ04064AAAF	2570	292.28	[USP9X]
FLJ04066AAAN	697	76.01	[SLC7A2]
FLJ04067AAAF	490	56.06	[STEAP2]
FLJ04068AAAN	362	39.12	[MSI1]
FLJ04071AAAF	378	40.53	[DAZAP1]
FLJ04075AAAN	1139	128.31	[RBL2]
FLJ04076AAAN	1977	214.12	[TP53BP1]
FLJ04081AAAN	1572	179.28	[SMARCA2]
FLJ04086AAAN	707	76.15	[SFPQ]
FLJ04088AAAN	1499	157.13	[BCL9L]
FLJ04090AAAN	2357	251.93	[SEC16A]
FLJ04091AAAN	1616	183.17	[DNMT1]

FLJ04093AAAN	1555	173.05	[PCF11]
FLJ04094AAAN	1708	192.22	[RICTOR]
FLJ04096AAAF	1556	178.7	[BAZ1A]
FLJ04097AAAN	1291	143.16	[SETDB1]
FLJ04098AAAN	519	57.53	[IKZF1]
FLJ04101AAAN	1464	159.21	[NCOA2]
FLJ04103AAAN	1594	183.06	[RB1CC1]
FLJ04104AAAN	866	94.93	[FGA]
FLJ04105AAAN	2224	251.7	[F5]
FLJ04107AAAN	2813	309.29	[VWF]
FLJ04109AAAN	2174	239.3	[MED13]
FLJ04116AAAN	1056	119.98	[KDM4C]
FLJ04118AAAN	1151	128.23	[ZFPM2]
FLJ04127AAAN	1205	135.74	[BRPF3]
FLJ04129AAAN	1236	134.53	[GRIN2C]
FLJ04131AAAN	859	97.21	[AGO2]
FLJ04137AAAN	858	95.34	[EEF2]
FLJ04138AAAN	963	109.68	[KIF5B]
FLJ04139AAAN	378	39.59	[HNRNPA3]
FLJ04143AAAF	2768	304.73	[TG]
FLJ04144AAAN	577	67.82	[MSN]
FLJ04153AAAN	911	99.85	[E2F7]
FLJ04160AAAN	955	110.02	[NEDD4L]
FLJ04161AAAN	1046	116.92	[OGT]
FLJ04170AAAN	2058	237.35	[MYO10]
FLJ04172AAAN	312	34.22	[UCP3]
FLJ04173AAAN	1060	120.41	[DNA2]
FLJ04175AAAN	2549	288.89	[MTOR]
FLJ04177AAAN	711	73.12	[KHSRP]
FLJ04182AAAN	1976	229	[MYH10]
FLJ04183AAAN	2508	273.2	[NCOR2]
FLJ04196AAAN	180	17.99	[CTAG1B]
FLJ04197AAAF	1746	195.42	[KNL1]
FLJ04198AAAN	1544	175.66	[KDM5B]
FLJ04199AAAN	2066	233.2	[TDRD6]
FLJ04201AAAN	1451	162.64	[TEX14]
FLJ04202AAAN	1390	158.55	[ATAD2]
FLJ04203AAAN	976	114.19	[SYCP1]
FLJ04204AAAN	188	21.69	[SSX6]
FLJ04206AAAN	97	10.97	[DSCR8]
FLJ04211AAAN	1255	136.58	[PER2]
FLJ04214AAAN	838	90.7	[RNF19A]
FLJ04224AAAF	2442	281.13	[CEP250]
FLJ04225AAAN	2230	261.14	[GOLGA4]
FLJ04227AAAF	2843	311.65	[APC]
FLJ04229AAAF	2302	262.41	[CHD8]
FLJ04241AAAN	1133	125.49	[FBF1]
FLJ04242AAAF	125	12.72	[OXT]

FLJ04250AAAN	1080	121.76	[CEP131]
FLJ04251AAAN	986	112.64	[CEP120]
FLJ04252AAAN	1368	151.93	[KIF24]
FLJ04256AAAN	1094	128.02	[CEP128]
FLJ04260AAAN	391	43.66	[TXNIP]
FLJ04271AAAN	862	91.09	[TAF4B]
FLJ04274AAAN	1132	125.04	[DAB2IP]
FLJ04286AAAF	1332	153.01	[SOS2]
FLJ04303AAAN	1140	133.49	[CEP135]
FLJ04308AAAN	843	93.56	[AXIN2]
FLJ04310AAAN	1539	168.85	[ZFYVE16]
FLJ04311AAAN	693	76.49	[TAB2]
FLJ04317AAAN	773	83.76	[TLE4]
FLJ04323AAAN	908	105.54	[CWC22]
FLJ04324AAAN	1146	127.59	[NOL6]
FLJ04332AAAN	1123	122.84	[USP43]
FLJ04358AAAN	977	107.55	[AP2A1]
FLJ04359AAAN	943	104.75	[ROR2]
FLJ04361AAAN	1426	157.6	[NPHP4]
FLJ04363AAAF	940	104.09	[AP2A2]
FLJ04383AAAN	1512	170.65	[EPRS]
FLJ06014AAAF	251	26.89	[AZU1]
FLJ06017AAAF	952	105.32	[GAA]
FLJ06018AAAF	639	65.43	[KRT2]
FLJ06019AAAF	267	28.52	[ELANE]
FLJ06022AAAF	1390	155.64	[POLR3A]
FLJ06024AAAF	529	58.73	[KCNA6]
FLJ06061AAAF	323	35.89	[PEX7]
FLJ06063AAAF	788	87.06	[ITGB3]
FLJ06214AAAF	635	71.24	[MPL]
FLJ06230AAAF	223	25.91	[POLR3G]
FLJ06233AAAF	285	32.18	[TIMMDC1]
FLJ06235AAAF	619	70.18	[NXF1]
FLJ07002AAAN	2322	250.54	[CSPG4]
FLJ07003AAAF	2321	243.63	[NOTCH3]
FLJ07005AAAF	1238	133.37	[JAG2]
FLJ07007AAAN	377	43.12	[NPSR1]
FLJ07012AAAN	838	90.36	[TACC3]
FLJ08002AAAF	662	73.17	[DDX3X]
FLJ08008AAAF	535	57.46	[CCT2]
FLJ08010AAAF	172	18.65	[CIRBP]
FLJ08011AAAF	347	39.32	[MAP2K3]
FLJ08014AAAF	119	13.28	[SNRPD1]
FLJ08015AAAF	408	46.87	[SSB]
FLJ08020AAAF	472	51.11	[EIF2S3]
FLJ08023AAAN	769	88	[STAT3]
FLJ08029AAAF	602	69.99	[PPP2R5D]
FLJ08030AAAF	467	54.67	[PPP2R5E]

FLJ08033AAAF	184	20.99	[RAP1A]
FLJ08043AAAF	354	39.19	[RFC2]
FLJ08044AAAN	240	27.87	[U2AF1]
FLJ08052AAAF	637	72.68	[PRMT5]
FLJ08058AAAF	126	13.92	[SNRPD3]
FLJ08063AAAF	334	37.49	[MAP2K6]
FLJ08066AAAF	168	18.39	[BAD]
FLJ08067AAAF	584	62.87	[SHC1]
FLJ08068AAAF	588	68	[PES1]
FLJ08069AAAF	306	34.85	[EBNA1BP2]
FLJ08071AAAF	672	76.76	[PRKCA]
FLJ08072AAAF	321	33.78	[FBL]
FLJ08073AAAF	213	21.36	[HIST1H1C]
FLJ08074AAAF	246	28.11	[YWHAB]
FLJ08076AAAF	471	53.14	[U2AF2]
FLJ08079AAAF	463	50.47	[EEF1A2]
FLJ08080AAAF	464	51.03	[HNRNPK]
FLJ08081AAAF	136	14.54	[SRP14]
FLJ08084AAAF	781	85.51	[CTNNB1]
FLJ08086AAAF	596	65.34	[TCF7L2]
FLJ08087AAAN	163	18.66	[SKP1]
FLJ08090AAAF	232	25.61	[AK2]
FLJ08094AAAF	638	72.2	[LIMK2]
FLJ08096SAAN	328	37.73	[GNRHR]
FLJ08097AAAF	496	55.8	[PRCP]
FLJ08098AAAF	427	48.09	[MAPK8]
FLJ08099AAAF	440	47.92	[TNFRSF10B]
FLJ08104AAAF	346	38.68	[ANXA1]
FLJ08105AAAF	269	28.53	[AQP1]
FLJ08110AAAF	191	21.7	[CIB1]
FLJ08113AAAF	417	48.14	[CALR]
FLJ08114AAAF	508	56.92	[CREB5]
FLJ08115AAAF	415	47.46	[CSNK1G2]
FLJ08118AAAF	336	37.81	[CASP7]
FLJ08119AAAF	464	53.65	[CASP8]
FLJ08121AAAF	734	81.9	[CTNNAL1]
FLJ08122AAAF	178	20.47	[CAV1]
FLJ08124AAAF	193	19.89	[CITED1]
FLJ08135AAAF	413	43.96	[E2F4]
FLJ08143AAAF	471	52.58	[GIT2]
FLJ08148AAAF	482	55.1	[HDAC1]
FLJ08153AAAF	569	64.76	[KLC1]
FLJ08161AAAF	70	8.07	[NDUFA1]
FLJ08162AAAF	307	35.06	[NMI]
FLJ08163AAAF	522	59.14	[OCLN]
FLJ08165AAAN	610	66.39	[PICALM]
FLJ08166AAAF	196	22.25	[PDGFA]
FLJ08169AAAF	416	47.59	[PSTPIP1]

FLJ08175AAAF	587	67.27	[PRKCI]
FLJ08179AAAF	201	22.99	[ARHGDIB]
FLJ08192AAAF	469	53.29	[TRAIP]
FLJ08203AAAN	191	22.31	[VEGFA]
FLJ08204AAAF	187	22.29	[IFNB1]
FLJ08205AAAF	166	19.35	[IFNG]
FLJ08206AAAF	153	17.63	[IL2]
FLJ08302AAAN	214	22.03	[PRDX5]
FLJ08305AAAN	462	49.61	[SHH]
FLJ08306AAAN	208	23.44	[FGF10]
FLJ08314AAAN	351	38.72	[WNT8B]
FLJ08315AAAN	348	39.13	[OPN1SW]
FLJ08318AAAN	351	38.85	[WNT8A]
FLJ08319AAAN	87	9.64	[PDE6G]
FLJ08320AAAN	348	38.89	[RHO]
FLJ08327AAAF	340	37.25	[GNB3]
FLJ08335AAAN	352	37.59	[LTB4R]
FLJ08342AAAN	233	24.08	[FRAT2]
FLJ08343AAAF	399	45.86	[SEC62]
FLJ08345AAAN	101	11.25	[APOC2]
FLJ08349AAAN	365	39.26	[FOXH1]
FLJ08351AAAN	355	39.55	[WNT3]
FLJ08356AAAN	129	14.7	[FSHB]
FLJ08364SAAN	840	93.83	[DSC1]
FLJ08372AAAN	207	23.76	[FGF16]
FLJ08374AAAN	141	15.35	[LHB]
FLJ08375AAAN	1337	145.94	[PTPRJ]
FLJ08378AAAN	653	73.26	[KCNA4]
FLJ08385AAAN	206	22.05	[FGF4]
FLJ08388AAAN	493	54.7	[CHRNE]
FLJ08394AAAF	189	21.81	[IFNA4]
FLJ08396AAAN	513	55.78	[DMP1]
FLJ08398AAAN	680	66.16	[COL10A1]
FLJ08399AAAN	745	83.87	[MPO]
FLJ08402AAAF	202	22.2	[C8G]
FLJ08414AAAF	610	68.07	[RYK]
FLJ08418AAAN	1447	158.46	[DCC]
FLJ08422AAAN	853	96.62	[TGS1]
FLJ08424SAAN	288	30.77	[FGF2]
FLJ08435AAAN	776	89.64	[CUL1]
FLJ08440AAAN	215	24.7	[CLEC4D]
FLJ08444AAAN	419	48.28	[FDPS]
FLJ08445AAAN	451	50.02	[TINF2]
FLJ08446AAAN	1071	123.39	[XPO1]
FLJ08449AAAF	353	41.4	[BRIX1]
FLJ08451AAAN	425	47.47	[F2R]
FLJ08453AAAN	498	57.51	[ANGPT1]
FLJ08454AAAF	439	50.44	[ARHGAP1]

FLJ08457AAAF	631	67.36	[HNF1A]
FLJ08461AAAN	1566	172.42	[RERE]
FLJ08475AAAN	1196	137.11	[AHI1]
FLJ08478AAAF	1620	176.44	[ALK]
FLJ08485AAAN	357	41.6	[ATP1B4]
FLJ08601AAAN	2442	265.35	[CREBBP]
FLJ08602AAAN	2555	272.5	[NOTCH1]
FLJ08607AAAN	1127	122.52	[TRIM33]
FLJ08608AAAN	368	41.02	[NT5C1A]
FLJ08609AAAN	213	24.03	[SOST]
FLJ08611SAAN	2000	226.59	[CHD3]
FLJ08613SAAN	439	48.95	[EXOSC9]
FLJ08713AAAN	236	28.19	[APOBEC1]
FLJ09001AAAF	1001	116.06	[TAOK1]
FLJ09002AAAF	819	88.29	[MYLK3]
FLJ09003AAAF	545	59.47	[STK39]
FLJ09004AAAF	333	36.98	[EFNB2]
FLJ09006AAAF	1434	160.87	[NOS1]
FLJ09007AAAF	529	61	[UGT2B28]
FLJ09008AAAF	61	6.91	[LENEP]
FLJ09009AAAF	89	9.85	[CCL18]
FLJ09012AAAF	120	13.78	[CREBL2]
FLJ09014AAAF	194	21.56	[MBD3L1]
FLJ09016AAAF	245	27.3	[PRRX1]
FLJ09017AAAF	247	27.32	[CMA1]
FLJ09018AAAF	277	30.55	[KLK13]
FLJ09020AAAF	415	47.67	[CRHR1]
FLJ09021AAAF	507	55.3	[SLC30A1]
FLJ09029AAAN	309	35.59	[AURKC]
FLJ09035AAAF	512	57.72	[ACVR2B]
FLJ09042AAAF	765	84.63	[ANKK1]
FLJ09043AAAN	806	87.71	[FGFR3]
FLJ09045AAAN	861	96.02	[MUSK]
FLJ09049AAAN	1130	122.87	[ABL1]
FLJ09051SAAN	1308	146.81	[ERBB4]
FLJ09052AAAF	1356	151.5	[KDR]
FLJ09053AAAN	1367	154.79	[IGF1R]
FLJ09054AAAF	1152	127.26	[MAP3K9]
FLJ09055AAAF	1050	112.6	[ULK1]
FLJ09059AAAF	1338	150.73	[FLT1]
FLJ09060AAAF	1354	158.17	[ROCK1]
FLJ09061AAAF	1370	155.2	[INSR]
FLJ09062AAAN	1388	160.9	[ROCK2]
FLJ09071AAAF	1783	191.58	[AKAP12]
FLJ09073AAAF	770	86.23	[DNMT3B]
FLJ09075AAAF	1464	165.31	[GRIN2A]
FLJ09076AAAF	1115	125.47	[GRIN3A]
FLJ09077AAAF	821	94.24	[CAPN3]

FLJ09078AAAF	703	79.14	[CAPN8]
FLJ09080AAAN	933	98.97	[PGR]
FLJ09081AAAF	431	49.46	[QRFPR]
FLJ09082AAAF	371	40.28	[AVPR2]
FLJ09083AAAF	340	38.81	[RASSF1]
FLJ09086AAAF	1086	117.34	[CAPN15]
FLJ09088AAAF	2471	265.35	[NOTCH2]
FLJ09089AAAF	2491	274.21	[IGF2R]
FLJ09090AAAF	348	39.56	[NR1I3]
FLJ09091AAAN	360	41.05	[PPM1L]
FLJ09095AAAF	283	30.77	[PDX1]
FLJ09096AAAF	474	52.75	[HNF4A]
FLJ09097AAAF	445	50.04	[HNF4G]
FLJ09098AAAF	626	68.23	[NR4A3]
FLJ09099AAAF	512	58.23	[RAB11FIP2]
FLJ09102WAAN	521	56.65	[RUNX2]
FLJ09103AAAN	906	99.63	[CBL]
FLJ09106AAAN	346	37.61	[E2F5]
FLJ09111AAAN	373	38.08	[FOXE1]
FLJ09121AAAF	1227	134.26	[RBM20]
FLJ09123AAAF	363	37.63	[GBX1]
FLJ09126AAAN	233	26.05	[BCL2L1]
FLJ09127AAAN	921	99.78	[NFATC2]
FLJ09130AAAN	615	67.82	[F12]
FLJ09131AAAN	400	44.74	[PROZ]
FLJ09133AAAN	1484	166.37	[GRIN2B]
FLJ09134AAAN	508	56.21	[ESRRB]
FLJ09143AAAN	442	44.57	[GATA4]
FLJ09144AAAN	391	39.02	[Sox1]
FLJ09148AAAN	183	21.14	[FTHL17]
FLJ09152AAAN	1016	108.05	[MAML1]
FLJ09153AAAN	307	33	[UCP1]
FLJ09154AAAN	366	41.33	[GHSR]
FLJ09156AAAN	2644	301.37	[ATR]
FLJ09157AAAN	4544	504.61	[LRP1]
FLJ09158AAAF	477	51.32	[ADRB1]
FLJ09160AAAN	395	45.03	[TDRD12]
FLJ09162AAAN	654	67.6	[TCF3]
FLJ09165AAAN	248	26.14	[MBL2]
FLJ09168AAAN	124	13.02	[MAGEA5]
FLJ09175AAAN	94	10.51	[CCL17]
FLJ09177AAAN	197	22.41	[NRTN]
FLJ09185AAAN	418	46.8	[AVPR1A]
FLJ09187AAAN	381	42.73	[NPY2R]
FLJ09188AAAN	349	38.98	[GALR1]
FLJ09189AAAN	387	41.7	[GALR2]
FLJ09214AAAF	798	91.03	[PPARGC1A]
FLJ09226AAAN	1198	130.98	[HIPK2]

FLJ10007AAAF	488	55.99	[SPATA6]
FLJ10029AAAF	141	14.76	[TMEM242]
FLJ10050AAAF	382	42.39	[AMACR]
FLJ10052AAAF	236	26.09	[SUSD4]
FLJ10060AAAF	448	46.34	[SLC52A1]
FLJ10067AAAF	456	51.16	[NARF]
FLJ10085AAAN	176	20.44	[MYH7B]
FLJ10094AAAF	643	73.29	[ENOX1]
FLJ10097AAAF	120	14.07	[BEX4]
FLJ10099AAAF	314	35.05	[TMEM248]
FLJ10110AAAF	194	21.01	[C9orf40]
FLJ10111AAAN	505	57.65	[RNF31]
FLJ10116AAAF	224	26.12	[MREG]
FLJ10123AAAF	518	57.75	[ASB3]
FLJ10134AAAF	275	31.68	[TMEM45A]
FLJ10143AAAF	468	50.22	[TAPBPL]
FLJ10153AAAF	211	23.71	[BAG2]
FLJ10154AAAF	273	33.22	[ARGLU1]
FLJ10156AAAF	238	26.27	[FAM64A]
FLJ10161AAAF	271	28.77	[NUBP2]
FLJ10162AAAF	71	7.85	[GNG2]
FLJ10165AAAF	397	42.75	[RBFOX1]
FLJ10181AAAF	392	42.92	[ILKAP]
FLJ10192AAAF	336	39.61	[TAF1A]
FLJ10193AAAF	146	16.4	[MED9]
FLJ10199AAAF	253	27.76	[TMEM51]
FLJ10204AAAF	205	23.68	[WDYHV1]
FLJ10225AAAF	469	51.9	[SAMM50]
FLJ10228AAAF	621	68.8	[ASMTL]
FLJ10231AAAF	325	38.41	[LUC7L]
FLJ10233AAAF	654	73.2	[WDR70]
FLJ10241AAAF	257	29.22	[ATP5SL]
FLJ10249AAAF	207	23.58	[RAB8B]
FLJ10252AAAF	528	58.94	[GPATCH2]
FLJ10260AAAF	578	66.94	[SLFN12]
FLJ10273AAAN	1000	111.12	[SEC23IP]
FLJ10276AAAF	430	47.16	[BSDC1]
FLJ10290AAAF	420	46.9	[RBM22]
FLJ10292AAAF	148	17.28	[MAGOHB]
FLJ10328AAAN	614	66.17	[HDAC7]
FLJ10330AAAF	546	64.41	[PRPF38B]
FLJ10335AAAF	375	42.91	[FANCL]
FLJ10349AAAF	310	34.55	[GPN2]
FLJ10356AAAF	419	48.16	[BZW2]
FLJ10373AAAF	270	29.86	[SMUG1]
FLJ10374AAAF	323	37.06	[CCDC94]
FLJ10377AAAF	759	85.77	[RBM28]
FLJ10385AAAF	548	59.32	[WRAP53]

FLJ10386AAAF	183	20.2	[MID1IP1]
FLJ10407AAAF	674	76.3	[NDC1]
FLJ10408AAAF	465	49.81	[FAM90A1]
FLJ10413AAAF	185	21.43	[GINS2]
FLJ10418AAAF	203	22.62	[HDGFRP3]
FLJ10419AAAF	493	55.11	[TBRG4]
FLJ10420AAAF	263	28.34	[NECAP2]
FLJ10424AAAF	183	22.22	[SRSF10]
FLJ10435AAAN	503	56.43	[SF3B3]
FLJ10439AAAF	385	42.29	[WDR74]
FLJ10441AAAF	628	68.85	[GSPT2]
FLJ10444AAAF	464	53.48	[ARHGAP8]
FLJ10448AAAF	253	28.02	[TSPAN3]
FLJ10449AAAF	147	16.74	[UBE2D2]
FLJ10458AAAN	485	53.27	[NLE1]
FLJ10459AAAF	209	23.65	[METTL5]
FLJ10460AAAF	235	26.93	[HAUS2]
FLJ10466AAAF	640	73.96	[EFHC1]
FLJ10469AAAF	680	79.62	[ZNF334]
FLJ10472AAAF	624	68.52	[RLIM]
FLJ10477AAAF	213	24.93	[THAP1]
FLJ10478AAAF	418	47.99	[ST3GAL5]
FLJ10479AAAF	344	39.8	[ZDHHC4]
FLJ10485AAAF	245	28.26	[AIG1]
FLJ10486AAAF	582	66.17	[MTPAP]
FLJ10488AAAF	326	37	[DOK4]
FLJ10490AAAF	129	13.72	[C19orf73]
FLJ10493AAAF	291	32.51	[TMEM38B]
FLJ10499AAAF	674	73.36	[PPP1R15A]
FLJ10502AAAF	415	45.67	[HNRNPF]
FLJ10504AAAF	570	61.8	[MSTO1]
FLJ10511AAAF	282	31.28	[ARMC1]
FLJ10525AAAF	247	27.95	[TMEM33]
FLJ10528AAAN	906	100.22	[SEC24D]
FLJ10530AAAF	854	95.14	[ELAC2]
FLJ10534AAAN	656	75.12	[TSR1]
FLJ10535AAAF	261	29.99	[PNPO]
FLJ10537AAAF	186	21.23	[DIABLO]
FLJ10541AAAN	557	63.98	[INTS8]
FLJ10542AAAF	398	45.28	[CCNB2]
FLJ10547AAAF	466	53.02	[IQCC]
FLJ10548AAAF	196	22.18	[MRPS18A]
FLJ10551AAAF	227	26.5	[YEATS4]
FLJ10558AAAF	284	30.38	[PEF1]
FLJ10559AAAN	316	35.24	[PRMT6]
FLJ10560AAAF	557	63.53	[TBCCD1]
FLJ10568AAAF	609	67.97	[KLHL20]
FLJ10569AAAF	597	69.09	[INTS10]

FLJ10572AAAF	708	80.15	[KLHL11]
FLJ10573AAAF	547	62.59	[REC8]
FLJ10574AAAF	437	49.71	[IFT52]
FLJ10575AAAF	494	55.47	[URI1]
FLJ10576AAAF	423	47.03	[FBXL2]
FLJ10581AAAF	420	47.02	[RNMTL1]
FLJ10582AAAF	179	20.21	[FAIM]
FLJ10584AAAF	147	16.65	[UBE2D4]
FLJ10585AAAF	197	21.6	[C11orf73]
FLJ10593SAAF	267	30.29	[PSMG1]
FLJ10604AAAF	202	22.43	[ASF1B]
FLJ10605AAAF	670	75.41	[DDX18]
FLJ10608AAAF	457	50.2	[NFS1]
FLJ10613AAAF	582	65.57	[GNL3L]
FLJ10618AAAF	310	34.06	[SLC25A36]
FLJ10620AAAN	536	60.84	[RIC8B]
FLJ10623AAAN	1197	135.6	[SMC2]
FLJ10624AAAF	796	88.95	[SLC4A1AP]
FLJ10628AAAF	164	18.59	[NUDT11]
FLJ10630AAAF	706	80.19	[ASUN]
FLJ10634AAAF	304	34.69	[DNAJC17]
FLJ10635AAAF	318	36.82	[DEDD]
FLJ10636AAAF	126	13.1	[CDK2AP2]
FLJ10640AAAF	692	78.46	[PRMT7]
FLJ10644AAAF	824	92.18	[DDX20]
FLJ10648AAAF	410	46.04	[BTBD7]
FLJ10649AAAF	339	37.33	[NSFL1C]
FLJ10650AAAF	296	34.73	[C14orf105]
FLJ10654AAAF	710	81.43	[FASTKD2]
FLJ10658WAAF	94	11.05	[SPINK13]
FLJ10662AAAF	364	40.91	[ZDHHC9]
FLJ10665AAAF	790	88.98	[PLEKHG6]
FLJ10666AAAF	220	24.96	[DGCR6L]
FLJ10670AAAF	411	46.65	[SMARCE1]
FLJ10672AAAF	253	29.43	[FGFR1OP2]
FLJ10682AAAN	1186	133.75	[AGTPBP1]
FLJ10693AAAF	206	22.98	[RAB18]
FLJ10702AAAF	186	21.54	[ARL8B]
FLJ10711AAAF	950	108.61	[XRN2]
FLJ10715AAAN	672	75.43	[BBS7]
FLJ10724AAAF	493	57.3	[HHAT]
FLJ10727AAAF	421	49.52	[TMLHE]
FLJ10730AAAF	547	62.58	[BCO1]
FLJ10732SAAF	646	71.64	[AKAP8L]
FLJ10737AAAF	559	63.34	[DNAJC11]
FLJ10738AAAF	496	56.35	[EXD2]
FLJ10739AAAF	347	40.14	[HMG20A]
FLJ10740AAAF	664	75.96	[RNF139]

FLJ10741AAAN	401	43.27	[MTG2]
FLJ10744AAAF	747	83.37	[WDR91]
FLJ10747AAAF	664	76.13	[TMEM57]
FLJ10748AAAF	350	38.86	[KLHDC8A]
FLJ10752AAAF	796	91.61	[VPS35]
FLJ10755AAAF	597	65.71	[SNCAIP]
FLJ10759AAAF	475	54.19	[TRIM62]
FLJ10761AAAF	394	44.87	[ETNK2]
FLJ10763AAAF	600	66.98	[IVNS1ABP]
FLJ10766AAAF	368	41.15	[FBXO28]
FLJ10767AAAF	406	44.67	[ARFGAP1]
FLJ10771AAAN	754	87.25	[SIN3B]
FLJ10778AAAF	882	98.05	[FAM115A]
FLJ10779AAAF	1332	150.25	[IKBKAP]
FLJ10780AAAF	795	87.07	[PLAA]
FLJ10782AAAF	773	85.99	[PANK4]
FLJ10783AAAF	578	64.8	[EDEM2]
FLJ10786AAAF	849	96.47	[CCDC87]
FLJ10789AAAF	469	53.22	[LMBR1L]
FLJ10790SAAF	599	67.49	[DDX52]
FLJ10791AAAF	279	32.67	[ELOVL1]
FLJ10793AAAF	372	42.24	[PARVA]
FLJ10796AAAF	623	69.2	[CHFR]
FLJ10803AAAF	146	16.72	[COA1]
FLJ10804SAAN	1053	112.23	[SALL4]
FLJ10805AAAF	513	57.56	[SMU1]
FLJ10811AAAF	702	78.29	[VRTN]
FLJ10813AAAF	490	54.77	[AP5M1]
FLJ10815AAAF	462	49.97	[SLC38A7]
FLJ10830AAAF	475	52.78	[CNDP2]
FLJ10834AAAF	975	112.48	[IPO11]
FLJ10837AAAF	621	71.45	[FBXO21]
FLJ10842AAAF	422	47.14	[AGK]
FLJ10843AAAF	412	46.66	[PLA2G15]
FLJ10846AAAF	438	50.07	[TMEM184C]
FLJ10847AAAF	570	61.94	[SLC47A1]
FLJ10849AAAF	429	49.3	[SEPT11]
FLJ10850AAAF	299	34.54	[UQCC1]
FLJ10851AAAF	1010	114.48	[OGDHL]
FLJ10856AAAF	361	40.68	[TMEM30A]
FLJ10858AAAF	605	67.88	[NEIL3]
FLJ10862AAAF	874	97.63	[COPG1]
FLJ10871AAAF	658	73.79	[INTS9]
FLJ10872AAAF	615	69.05	[WDTC1]
FLJ10876AAAF	495	55.48	[RCOR3]
FLJ10882AAAF	627	68.62	[ZFP64]
FLJ10883AAAF	361	41.09	[PCMTD2]
FLJ10887AAAF	518	58.16	[KBTBD4]

FLJ10891AAAN	465	53.86	[ZNF701]
FLJ10897AAAN	993	113.2	[IFT122]
FLJ10899AAAF	357	40.25	[GPRC5A]
FLJ10900AAAF	732	83.72	[TYW1]
FLJ10901AAAF	663	72.89	[C1orf106]
FLJ10902AAAF	488	55.72	[TMEM39A]
FLJ10903AAAF	151	16.11	[H2AFJ]
FLJ10904AAAF	459	51.74	[WDR41]
FLJ10913AAAF	179	21.51	[ADI1]
FLJ10914AAAN	204	22.42	[MRGBP]
FLJ10917AAAF	250	28.69	[SBDS]
FLJ10918AAAF	379	43.12	[TOR1AIP1]
FLJ10922AAAF	128	14.3	[TMEM143]
FLJ10928AAAF	141	16.36	[SSH3]
FLJ10945AAAF	138	14.72	[PRR34]
FLJ10948AAAF	261	28.11	[ECHDC2]
FLJ10964AAAN	233	26.92	[LRIF1]
FLJ10973AAAF	450	51.91	[SNX4]
FLJ10975AAAF	410	46.65	[PHF10]
FLJ10982AAAF	265	29.93	[OLAH]
FLJ10983AAAF	612	68.27	[PGM2]
FLJ10989AAAF	528	57.46	[QRSL1]
FLJ10990AAAF	584	64.71	[EIF2D]
FLJ10998AAAF	538	60.62	[CWF19L1]
FLJ11000AAAF	185	20.39	[TMEM140]
FLJ11006AAAF	497	57.74	[MCOLN3]
FLJ11008AAAF	278	29.51	[DHRS4]
FLJ11012AAAF	445	51.44	[DCAF13]
FLJ11016AAAF	405	46.34	[RBM41]
FLJ11017AAAF	314	33.67	[C4orf19]
FLJ11018AAAF	101	11.38	[URM1]
FLJ11025AAAF	328	37.51	[PRKAG2]
FLJ11028AAAF	340	37.57	[GNB4]
FLJ11029AAAF	360	40.15	[PRR11]
FLJ11037AAAF	275	32.38	[ATG5]
FLJ11039AAAF	426	48.63	[ZC3H15]
FLJ11040AAAN	484	55.64	[RHOT1]
FLJ11046AAAF	684	76.4	[TBC1D23]
FLJ11069AAAF	188	21.88	[TNFAIP8]
FLJ11071AAAN	553	62.28	[FBXW7]
FLJ11072AAAF	270	29.75	[MED4]
FLJ11077AAAF	483	53.23	[ACOT2]
FLJ11078AAAF	615	68.14	[KLHL26]
FLJ11082AAAF	526	60.14	[TBC1D19]
FLJ11084AAAF	535	59.84	[NRBP1]
FLJ11092AAAF	365	40.8	[SH3GLB1]
FLJ11094AAAF	331	36.69	[TRUB2]
FLJ11101AAAN	183	20.16	[SAYSD1]

FLJ11103AAAF	351	40.7	[RRM2B]
FLJ11105AAAF	481	54.75	[PI4K2B]
FLJ11107AAAF	582	63.25	[DCP1A]
FLJ11110AAAF	329	37.53	[GIMAP4]
FLJ11111AAAF	865	98.86	[L1TD1]
FLJ11112AAAF	175	19.67	[SPTLC3]
FLJ11115AAAF	485	53.38	[DNPEP]
FLJ11118AAAF	531	60.22	[PARP2]
FLJ11119AAAF	121	13.39	[ENSA]
FLJ11127AAAF	356	42.2	[FAM105A]
FLJ11134AAAN	584	62.27	[EPN2]
FLJ11137AAAF	326	35.12	[ZNF444]
FLJ11149AAAF	162	18.38	[RFK]
FLJ11155AAAF	345	37.64	[TMEM144]
FLJ11159AAAF	552	63.38	[RIOK2]
FLJ11162AAAN	528	60.74	[MKLN1]
FLJ11164AAAF	442	48.71	[RSAD1]
FLJ11168AAAF	200	22.52	[AP5S1]
FLJ11169AAAN	570	64.11	[TM7SF3]
FLJ11173AAAF	564	63.97	[TRIM16]
FLJ11182AAAF	105	11.46	[SLC39A3]
FLJ11184AAAF	203	23.81	[TMA16]
FLJ11185AAAF	422	48.1	[KRT23]
FLJ11188AAAF	513	54.94	[ZNF395]
FLJ11190AAAF	256	27.55	[TMEM74B]
FLJ11191AAAF	555	64.1	[ZNF415]
FLJ11193AAAF	442	49.98	[C5orf22]
FLJ11196AAAF	491	54.68	[LARP6]
FLJ11198AAAF	709	79.77	[ABCF3]
FLJ11199AAAF	1037	117.45	[PITRM1]
FLJ11200AAAF	469	53.19	[UFSP2]
FLJ11202AAAF	558	61.76	[SPATS2L]
FLJ11215AAAF	197	21.83	[LIN7C]
FLJ11218AAAF	315	34.95	[RNLS]
FLJ11219AAAF	481	54.31	[TRMT13]
FLJ11220AAAF	754	85	[RSBN1]
FLJ11222AAAF	495	60.57	[MNS1]
FLJ11230AAAF	217	23.38	[BLOC1S4]
FLJ11237AAAF	243	26.55	[LAT2]
FLJ11240AAAF	540	61.39	[LMBRD1]
FLJ11246AAAF	514	57.19	[PLRG1]
FLJ11250AAAF	282	31.83	[RAD1]
FLJ11265AAAF	562	61.43	[DIDO1]
FLJ11267AAAF	159	18.38	[C8orf44]
FLJ11268AAAF	103	11.59	[TIMM10B]
FLJ11271AAAF	145	15.9	[SYNJ2BP]
FLJ11273AAAF	274	31.11	[TMEM106B]
FLJ11274AAAF	307	32.22	[SLC39A9]

FLJ11275AAAF	247	28.25	[NIPSNAP3B]
FLJ11278AAAF	374	40.54	[FBXL8]
FLJ11282AAAF	540	59.55	[DDX28]
FLJ11285AAAF	202	22.97	[COMMD10]
FLJ11286AAAF	291	33.09	[C19orf66]
FLJ11294AAAF	326	38.29	[WDR33]
FLJ11295AAAF	194	21.49	[SLMO2]
FLJ11297AAAF	449	50.07	[UBA3]
FLJ11303AAAF	213	23.91	[CHMP2B]
FLJ11305AAAF	453	52.09	[PCID2]
FLJ11316AAAF	304	32.94	[MBNL3]
FLJ11323AAAF	323	36.67	[PLCXD1]
FLJ11329AAAF	212	23.41	[C10orf10]
FLJ11336AAAF	509	57.03	[TCP11L1]
FLJ11340AAAF	308	33.84	[SAP30BP]
FLJ11342AAAF	306	33.93	[ABHD10]
FLJ11354AAAF	678	76.64	[DHX58]
FLJ11357AAAF	376	43.16	[SMARCB1]
FLJ11360AAAF	577	65.39	[DCLRE1C]
FLJ11387AAAF	317	35.49	[DPP3]
FLJ11393AAAF	390	42.19	[HNRNPUL1]
FLJ11464AAAF	558	61.07	[ISYNA1]
FLJ11497AAAF	437	51.14	[FBXO9]
FLJ11506AAAF	315	34.59	[AAGAB]
FLJ11513AAAF	421	48	[TES]
FLJ11538AAAN	515	56.66	[POLL]
FLJ11543AAAF	467	52.99	[DMAP1]
FLJ11560AAAF	442	45.55	[FAM214B]
FLJ11577AAAF	301	33.89	[CXorf21]
FLJ11584AAAF	610	68.68	[MYNN]
FLJ11594AAAF	137	14.27	[C17orf53]
FLJ11601AAAF	298	34.81	[THG1L]
FLJ11619AAAF	454	52.59	[SEPT10]
FLJ11623AAAF	209	23.55	[GMNN]
FLJ11626AAAF	315	34.48	[NSUN5P2]
FLJ11637AAAF	456	51.58	[ZNF556]
FLJ11659AAAF	275	30.28	[COPS7A]
FLJ11694AAAF	429	47.46	[ACTL6A]
FLJ11706AAAF	669	69.52	[RBM14]
FLJ11712AAAF	312	35.1	[RNASEH2B]
FLJ11718AAAF	403	46.43	[JMJD6]
FLJ11721AAAF	116	11.75	[TMEM263]
FLJ11730AAAF	191	21.63	[MEAF6]
FLJ11743AAAF	439	47.32	[ABHD8]
FLJ11746AAAF	285	32.75	[CNOT7]
FLJ11747AAAF	503	56.31	[ANAPC7]
FLJ11749AAAF	201	23.45	[CHMP6]
FLJ11752AAAF	394	44.92	[GORAB]

FLJ11767AAAF	211	24.49	[EFCAB1]
FLJ11773AAAF	218	24.98	[ATG101]
FLJ11800AAAF	169	18.79	[FAM106A]
FLJ11807AAAF	227	25.94	[UBTD1]
FLJ11808AAAF	434	47.19	[SUGCT]
FLJ11820AAAF	229	25.86	[DIRAS3]
FLJ11848AAAF	392	42.19	[PAAF1]
FLJ11853AAAF	744	81.85	[PCDHB13]
FLJ11856AAAF	445	45.78	[SLC52A2]
FLJ11866AAAN	206	23.36	[RAB3GAP2]
FLJ11888AAAF	225	25.62	[PRTFDC1]
FLJ11945AAAF	602	67.66	[HAP1]
FLJ11961AAAF	518	55.76	[SOGA1]
FLJ11974AAAF	400	44.1	[WWTR1]
FLJ11993AAAF	488	52.76	[LAP3]
FLJ12013AAAF	485	55.42	[RNF8]
FLJ12015AAAF	145	15.55	[BAALC]
FLJ12027AAAF	435	50.02	[RASSF9]
FLJ12042AAAF	280	31.32	[CDCA8]
FLJ12057AAAF	161	17.67	[EFCC1]
FLJ12068AAAF	542	63.22	[OGFOD1]
FLJ12069AAAF	335	37.46	[GLRX3]
FLJ12076AAAF	258	28.71	[C16orf70]
FLJ12085AAAN	674	77.27	[TBC1D15]
FLJ12089AAAF	394	46.4	[CERS4]
FLJ12114AAAF	365	40.83	[LMCD1]
FLJ12116AAAF	486	54.24	[GAL3ST4]
FLJ12118AAAF	564	62.25	[CARS2]
FLJ12133AAAF	323	36.17	[CALHM2]
FLJ12145AAAF	572	62.8	[MLPH]
FLJ12150AAAF	484	52.8	[GSDMD]
FLJ12154AAAF	538	58.73	[C14orf93]
FLJ12168AAAN	648	72.65	[TBC1D17]
FLJ12171AAAF	309	34.44	[FN3KRP]
FLJ12172AAAF	677	73.37	[OGFR]
FLJ12179AAAF	1025	115.75	[NAT10]
FLJ12193AAAF	160	16.94	[C8orf60]
FLJ12222AAAN	275	30.37	[OVOL2]
FLJ12242AAAF	290	32.49	[KCTD17]
FLJ12249AAAF	310	32.3	[PARM1]
FLJ12255AAAF	277	31.43	[UCK1]
FLJ12260AAAN	212	23.96	[UBR4]
FLJ12262AAAF	451	51.08	[TUBG2]
FLJ12268AAAF	295	33.2	[TBX6]
FLJ12296AAAF	289	32.06	[ZMAT3]
FLJ12298AAAF	561	64.27	[ZNF394]
FLJ12311AAAF	502	55.28	[ZC3HC1]
FLJ12340AAAF	384	42.52	[SPHK1]

FLJ12374AAAF	706	79.29	[NADSYN1]
FLJ12382AAAF	487	53.28	[UBOX5]
FLJ12388AAAF	461	51.67	[NEU3]
FLJ12389AAAF	672	75.07	[AACS]
FLJ12392AAAF	576	60.29	[EPN1]
FLJ12397AAAF	424	48.5	[CCPG1]
FLJ12398AAAF	293	32.79	[EXOSC2]
FLJ12408AAAF	341	38.92	[LIMS2]
FLJ12419AAAF	510	56.43	[PIAS4]
FLJ12429AAAF	331	39.45	[B3GALNT1]
FLJ12432AAAN	806	90.63	[RAB3GAP1]
FLJ12434AAAF	1297	143.67	[RRP12]
FLJ12439AAAF	231	25.71	[COA7]
FLJ12440AAAF	807	92.12	[TMEM63A]
FLJ12441AAAF	124	13.7	[NDUFS6]
FLJ12444AAAF	241	27.57	[ZCCHC17]
FLJ12445AAAF	682	76.91	[GFPT2]
FLJ12447AAAF	962	106.83	[INTS7]
FLJ12448AAAF	262	28.17	[C12orf43]
FLJ12449AAAF	1401	157.2	[NCAPD2]
FLJ12450AAAF	1015	114.22	[NCAPG]
FLJ12451AAAF	140	16.11	[DUSP10]
FLJ12452AAAF	230	26.05	[NMRK2]
FLJ12455AAAF	525	59.29	[GPATCH3]
FLJ12456AAAF	245	28.36	[EIF4E2]
FLJ12457AAAF	209	22.74	[LIN28A]
FLJ12465AAAF	928	103.08	[FAM129A]
FLJ12466AAAF	767	85.73	[CSDE1]
FLJ12472AAAF	417	46.25	[ACTR10]
FLJ12475AAAF	349	40.08	[RPF1]
FLJ12476AAAF	621	71.1	[IQCH]
FLJ12479AAAN	925	101.16	[RBM15]
FLJ12482AAAF	639	70.15	[CTTNBP2NL]
FLJ12486AAAN	973	106.34	[ITGA7]
FLJ12498AAAF	645	72.62	[WHSC1L1]
FLJ12502AAAF	616	66.44	[C14orf159]
FLJ12516AAAF	420	46.29	[GMPPA]
FLJ12517AAAF	463	52.52	[JMJD4]
FLJ12518AAAF	634	71.44	[POT1]
FLJ12524AAAF	355	40.41	[MAEA]
FLJ12525AAAF	734	83.06	[LAS1L]
FLJ12526AAAF	155	17.46	[RNF122]
FLJ12528AAAF	588	66.37	[TARS2]
FLJ12533AAAF	571	64.06	[PRDM14]
FLJ12538AAAF	212	23.49	[RAB17]
FLJ12542AAAF	659	74.29	[CEP76]
FLJ12543AAAF	616	69.87	[KLHL36]
FLJ12549AAAF	656	75	[NUP85]

FLJ12554AAAF	644	72.82	[ECD]
FLJ12563AAAF	224	25.31	[ATP6AP1L]
FLJ12564AAAF	547	62.26	[ELP3]
FLJ12571AAAF	554	64.16	[TTC26]
FLJ12576AAAF	495	54.57	[FKRP]
FLJ12578AAAF	437	48.75	[SEC61A2]
FLJ12581AAAF	305	34.61	[NANOG]
FLJ12582AAAF	158	17.03	[DBNDD1]
FLJ12594AAAF	261	28.93	[ENOPH1]
FLJ12606AAAF	464	52.62	[ZNF669]
FLJ12610AAAF	299	33.34	[NHEJ1]
FLJ12612AAAF	264	29.62	[COPS7B]
FLJ12618AAAF	463	50.32	[L2HGDH]
FLJ12619AAAF	229	27.08	[C6orf62]
FLJ12623AAAF	232	25.21	[UNKL]
FLJ12638AAAF	507	58.18	[ALG6]
FLJ12646AAAN	571	63.1	[KIRREL]
FLJ12647AAAN	800	89.59	[ELP2]
FLJ12648AAAF	314	35.62	[CPPED1]
FLJ12663AAAF	386	42.32	[PPME1]
FLJ12671AAAF	353	39.15	[ISG20L2]
FLJ12673AAAF	561	62.39	[FBXO11]
FLJ12681AAAF	567	64.87	[LMF1]
FLJ12684AAAN	563	63.55	[ERVMER34-1]
FLJ12691AAAF	557	64.29	[GALNT14]
FLJ12695AAAF	350	38.25	[YIPF3]
FLJ12697AAAF	1197	130.5	[USP42]
FLJ12700AAAF	155	17.2	[ZNF767P]
FLJ12701AAAF	332	37.54	[MRPL44]
FLJ12705AAAF	443	51.46	[TADA2A]
FLJ12710AAAF	91	9.96	[LSM5]
FLJ12712AAAF	547	61.58	[DDX56]
FLJ12714AAAF	372	40.06	[H2AFY2]
FLJ12719AAAF	423	47.54	[WDR12]
FLJ12723AAAF	119	14.17	[NDUFC2]
FLJ12732AAAN	772	84.71	[RPAP1]
FLJ12746AAAF	364	41.11	[LPAR1]
FLJ12748AAAF	199	21	[MAP6D1]
FLJ12750AAAF	285	31.31	[VPS37B]
FLJ12754AAAF	702	77.9	[SUN1]
FLJ12761AAAN	613	65.69	[SAP130]
FLJ12765AAAF	860	97.33	[AGO3]
FLJ12766AAAF	678	74.78	[SLC25A12]
FLJ12768AAAF	579	63.47	[PIGZ]
FLJ12784AAAF	749	85.64	[INPP5B]
FLJ12785AAAF	607	68.3	[ACTR5]
FLJ12791AAAF	289	32.26	[SLC6A15]
FLJ12798AAAF	342	36.72	[WDR77]

FLJ12800AAAF	467	49.65	[CCDC71]
FLJ12802AAAF	828	94.07	[USP33]
FLJ12811AAAF	543	59.98	[KIDINS220]
FLJ12812AAAF	785	88.99	[COG4]
FLJ12814AAAF	313	34.27	[ANKRA2]
FLJ12816AAAF	342	38.67	[ABHD4]
FLJ12826AAAF	410	46.32	[WDFY1]
FLJ12827AAAF	720	78.44	[ZNF408]
FLJ12833AAAF	392	46.51	[LUC7L2]
FLJ12834AAAF	534	60.98	[PSMD3]
FLJ12837AAAF	863	96.6	[MCM4]
FLJ12842AAAN	373	40.85	[RCL1]
FLJ12847AAAN	740	82.21	[MCPH1]
FLJ12863AAAF	493	57.01	[VIPAS39]
FLJ12865AAAF	434	48.38	[CMAS]
FLJ12871AAAN	1017	114.16	[HYDIN]
FLJ12872AAAF	799	89.39	[COPG2]
FLJ12875AAAF	352	39.72	[MUL1]
FLJ12879AAAN	825	88.63	[EHMT1]
FLJ12886AAAF	495	54.79	[SMG9]
FLJ12888AAAF	625	70.02	[RMI1]
FLJ12889AAAF	602	68.25	[LRRC40]
FLJ12890AAAF	744	82.36	[CNOT10]
FLJ12896AAAF	111	12.39	[BET1L]
FLJ12899AAAF	370	41.11	[PANK3]
FLJ12910AAAF	441	51.17	[ARMT1]
FLJ12912AAAF	526	59.63	[CCNL1]
FLJ12916AAAF	835	93.67	[FAM35A]
FLJ12920AAAF	682	76.85	[CKAP2]
FLJ12921AAAF	753	79.13	[SYNPO2L]
FLJ12934AAAF	624	70.43	[ACTR8]
FLJ12942AAAF	421	47.14	[ASB6]
FLJ12953AAAF	334	35.89	[WDR54]
FLJ12957AAAN	687	77.58	[EPB41L5]
FLJ12960AAAF	415	46.71	[QTRTD1]
FLJ12963AAAF	675	78.08	[ZNF443]
FLJ12966AAAF	270	31.14	[TMEM189]
FLJ12967AAAN	186	19.75	[PHC3]
FLJ12973AAAF	626	69.83	[WDR76]
FLJ12982AAAF	719	79.62	[UIMC1]
FLJ12983AAAF	177	20.4	[AGBL3]
FLJ12989AAAF	725	80.43	[MCCC1]
FLJ13002AAAF	417	45.47	[PREB]
FLJ13008AAAF	224	24.67	[COMMD5]
FLJ13009AAAF	383	44.63	[SAV1]
FLJ13013AAAF	495	56.19	[RAD18]
FLJ13028AAAF	169	19.4	[NAA50]
FLJ13029AAAF	803	91.92	[ZNF226]

FLJ13030AAAF	212	23.35	[MED20]
FLJ13032AAAF	536	62	[ZNF20]
FLJ13036AAAF	596	69.67	[CEP70]
FLJ13037AAAN	907	105.08	[ZNF112]
FLJ13044AAAF	323	34.47	[SLC25A22]
FLJ13048AAAN	153	16.93	[SSU72]
FLJ13052AAAN	447	49.36	[NADK]
FLJ13055AAAF	343	36.85	[LPPR2]
FLJ13057AAAF	515	58.7	[GMCL1]
FLJ13062AAAF	648	71.99	[SCMH1]
FLJ13067AAAF	553	61.21	[HP1BP3]
FLJ13068AAAF	715	77.54	[ZDHHC5]
FLJ13071AAAF	576	61.91	[MYEF2]
FLJ13075AAAF	563	64.78	[MGAT4B]
FLJ13076AAAF	1208	138.6	[DZIP3]
FLJ13077AAAF	327	37.88	[RNF121]
FLJ13080AAAF	674	73.98	[FIGNL1]
FLJ13081AAAF	642	72.98	[MCMBP]
FLJ13087AAAF	170	18.39	[DBF4B]
FLJ13091AAAF	374	42.25	[FANCF]
FLJ13092AAAF	190	20.06	[HN1L]
FLJ13094AAAF	413	47.38	[FAM187A]
FLJ13102AAAF	334	38.87	[DHDDS]
FLJ13107AAAF	340	36.57	[SRR]
FLJ13108AAAF	570	65.08	[VPS45]
FLJ13110AAAF	201	22.25	[REEP1]
FLJ13113AAAF	591	67.28	[ZWILCH]
FLJ13132AAAF	558	63.24	[ABHD16A]
FLJ13134AAAF	278	29.98	[ASB13]
FLJ13140AAAF	545	59.64	[SPATS2]
FLJ13144AAAF	726	80.93	[ANKZF1]
FLJ13147AAAF	651	72.88	[MED17]
FLJ13149AAAF	764	86.57	[FASTKD5]
FLJ13150AAAF	612	69.45	[RPAP2]
FLJ13152AAAF	677	75.42	[CLPB]
FLJ13153AAAF	412	45.98	[ZDHHC11]
FLJ13154AAAF	265	30.25	[USB1]
FLJ13157AAAF	662	72.96	[PBXIP1]
FLJ13158AAAF	323	36.38	[ATAT1]
FLJ13161AAAF	200	22.54	[RAB10]
FLJ13162AAAF	128	13.65	[LINC00574]
FLJ13163AAAF	491	56.02	[IPPK]
FLJ13167AAAF	520	58.38	[SPATA2]
FLJ13168AAAF	391	44.17	[TTC23]
FLJ13170AAAF	620	66.08	[SPATA5L1]
FLJ13173AAAF	368	41.08	[EXOG]
FLJ13179AAAF	243	27.23	[VAPB]
FLJ13181AAAF	580	64.33	[TRIM45]

FLJ13185AAAN	644	73.05	[USP16]
FLJ13187AAAF	249	27.8	[PLEKHF2]
FLJ13188AAAF	233	27.02	[FAM204A]
FLJ13189AAAF	130	13.88	[LINC00472]
FLJ13191AAAF	348	39.14	[DNAJB5]
FLJ13195AAAF	150	17.02	[STAG3L4]
FLJ13196AAAF	634	70.19	[PHF21A]
FLJ13198AAAF	356	41.87	[HS2ST1]
FLJ13201AAAF	507	56.97	[THUMPD3]
FLJ13203AAAF	486	53.65	[CPEB1]
FLJ13204AAAF	216	25.46	[MOB3B]
FLJ13206AAAF	530	57.89	[CHST2]
FLJ13212AAAF	503	56.33	[UBP1]
FLJ13217AAAF	579	67.43	[VPS11]
FLJ13220AAAF	669	74.27	[GUF1]
FLJ13222AAAF	227	25.18	[ZFAND3]
FLJ13227AAAF	197	22.73	[NXT2]
FLJ13256AAAF	476	54.28	[MICU1]
FLJ13258AAAF	292	33.13	[AKTIP]
FLJ13259AAAF	216	25.05	[MOB1A]
FLJ13263AAAF	123	14.21	[TMEM254]
FLJ13265AAAF	155	16.7	[CNTD2]
FLJ13269AAAF	492	56.25	[TMEM39B]
FLJ13280AAAF	437	48.35	[ERAL1]
FLJ13291AAAF	309	35.1	[SLC7A6OS]
FLJ13297AAAF	247	29.09	[CLUAP1]
FLJ13315AAAF	139	16.1	[HEXA-AS1]
FLJ13316AAAF	350	38.98	[NIF3L1]
FLJ13317AAAF	282	32.29	[SRSF8]
FLJ13319AAAN	416	46.53	[CEPT1]
FLJ13321AAAF	541	60.53	[ATL3]
FLJ13322AAAF	305	34.76	[ACBD4]
FLJ13323AAAF	284	32.72	[TPM4]
FLJ13347AAAF	372	39.59	[H2AFY]
FLJ13348AAAF	583	62.37	[SLCO4A1]
FLJ13352AAAF	318	36.52	[SRD5A3]
FLJ13374AAAF	357	41.23	[ASCC1]
FLJ13386AAAF	541	63.01	[CEP63]
FLJ13391AAAF	152	17.47	[EVA1A]
FLJ13397AAAF	445	49.72	[FAM188A]
FLJ13405AAAF	532	59.93	[ZNF350]
FLJ13417AAAN	853	94.33	[CNNM2]
FLJ13421AAAF	440	49.32	[NUSAP1]
FLJ13431AAAF	289	31.54	[DLX5]
FLJ13441AAAF	501	55.66	[BRD9]
FLJ13444AAAF	327	37.38	[B4GALT7]
FLJ13448AAAF	238	27.07	[COQ10B]
FLJ13462AAAF	215	23.9	[RAB14]

FLJ13465AAAF	949	104.38	[MMRN2]
FLJ13475AAAF	131	14.71	[CHRAC1]
FLJ13490AAAF	445	49.25	[C10orf88]
FLJ13491AAAF	290	32.12	[OGFOD2]
FLJ13498AAAF	328	36.6	[GET4]
FLJ13517AAAF	151	17.26	[RNF138]
FLJ13519AAAF	519	58.47	[SNX2]
FLJ13521AAAF	690	78.19	[CPSF2]
FLJ13540AAAN	165	17.98	[CEACAM21]
FLJ13560AAAF	431	46.81	[MANSC1]
FLJ13584AAAF	346	37.85	[XRCC3]
FLJ13607AAAF	339	39.54	[CENPN]
FLJ13610AAAF	432	51.47	[LUC7L3]
FLJ13614AAAN	300	34.45	[FAM175A]
FLJ13619AAAF	592	68.91	[FNBP1]
FLJ13631AAAF	244	28.32	[METTL7A]
FLJ13639AAAF	242	27.14	[DHRS12]
FLJ13640AAAF	719	81.12	[NOL11]
FLJ13641AAAN	235	27.09	[USP15]
FLJ13642AAAF	441	49.35	[ANKMY2]
FLJ13645AAAN	435	51.43	[IFT74]
FLJ13657AAAF	361	38.34	[CAAP1]
FLJ13660AAAF	506	56.92	[CDK5RAP3]
FLJ13661AAAF	390	43.32	[HS3ST3B1]
FLJ13664AAAF	541	57.45	[ACD]
FLJ13689AAAF	648	74.67	[ZNF202]
FLJ13693AAAN	380	41.32	[TREML2]
FLJ13697AAAN	654	74.18	[CSRP2BP]
FLJ13768AAAF	1727	192.23	[BAZ2A]
FLJ13770AAAF	653	74.67	[EXOC7]
FLJ13782AAAF	625	71.09	[GRHL2]
FLJ13798AAAF	416	47.21	[KDM8]
FLJ13805AAAF	480	52.82	[ATG13]
FLJ13817AAAF	212	24.28	[PXMP4]
FLJ13818AAAN	584	65.26	[IPP]
FLJ13822AAAF	474	53.84	[RNF14]
FLJ13834AAAF	547	59.2	[MON1B]
FLJ13841AAAF	723	77.39	[ZNF750]
FLJ13842AAAF	229	25.83	[ZMAT4]
FLJ13848AAAF	237	27.22	[NAA40]
FLJ13852AAAF	274	28.66	[PYCRL]
FLJ13853AAAF	575	58.85	[DAK]
FLJ13859AAAF	705	78.74	[TTC12]
FLJ13862AAAF	622	67.22	[DTX2]
FLJ13865AAAF	590	66.22	[NELFCD]
FLJ13868AAAF	468	50.96	[C16orf58]
FLJ13870AAAF	270	30.43	[SNX11]
FLJ13875AAAF	594	67.15	[ACOT11]

FLJ13894AAAF	514	59.26	[OASL]
FLJ13896AAAF	650	72.58	[DUS3L]
FLJ13902AAAF	184	21	[PRKRIP1]
FLJ13907AAAN	779	88.87	[NOD1]
FLJ13908AAAF	384	43.9	[AIPL1]
FLJ13910AAAF	391	43.99	[RMND5A]
FLJ13911AAAN	114	12.15	[H2AFV]
FLJ13912AAAF	138	15.66	[GINS3]
FLJ13914AAAF	796	87.92	[YY1AP1]
FLJ13917AAAF	112	12.5	[LIN52]
FLJ13918AAAF	253	29.66	[BIN3]
FLJ13921AAAF	373	41.39	[AUP1]
FLJ13924AAAF	520	57.8	[KLHDC4]
FLJ13925AAAF	486	53.81	[FOXRED1]
FLJ13929AAAF	259	28.43	[PEX11B]
FLJ13930AAAF	587	66.02	[CDK5RAP1]
FLJ13931AAAF	403	41.48	[PCBP4]
FLJ13933AAAF	520	60.72	[NT5DC2]
FLJ13934AAAF	436	48.08	[KPTN]
FLJ13935AAAF	313	34.19	[TEX264]
FLJ13940AAAF	241	27.55	[TUBA4B]
FLJ13941AAAF	497	53.85	[MORN1]
FLJ13943AAAN	406	46.27	[COPS4]
FLJ13949AAAF	510	56.57	[TOE1]
FLJ13952AAAF	193	22.08	[SNX22]
FLJ13953AAAF	247	28.44	[NIPSNAP3A]
FLJ13955AAAF	368	40.78	[NIPAL2]
FLJ13956AAAF	131	14.54	[GEMIN7]
FLJ13957AAAF	252	28.11	[ORC6]
FLJ13958AAAF	388	42.83	[MIIP]
FLJ13970AAAF	419	47.26	[POLR1E]
FLJ13975WAAF	71	8.02	[C12orf73]
FLJ13976AAAF	762	85.62	[CEP85]
FLJ13979AAAF	319	35.42	[CRYL1]
FLJ13983AAAF	426	49.19	[IP6K2]
FLJ13986AAAF	276	30.59	[FBXO22]
FLJ13988AAAF	162	18.66	[UBE2W]
FLJ13992AAAF	358	41.55	[DNAJC18]
FLJ13993AAAF	158	17.53	[CPLX3]
FLJ13998AAAF	532	60	[DCLRE1B]
FLJ14002AAAF	395	44.53	[ASTN2]
FLJ14003AAAF	394	44.4	[PHAX]
FLJ14004AAAF	350	40.07	[TMEM206]
FLJ14007AAAF	268	30.75	[ZFAND1]
FLJ14009AAAF	449	49.83	[TLE6]
FLJ14013AAAF	387	44.16	[FBXO4]
FLJ14027AAAF	593	62.43	[CRTC1]
FLJ14034AAAF	199	22.38	[TUBD1]

FLJ14040AAAF	458	52.38	[KAT8]
FLJ14062AAAF	385	44.34	[MTERF2]
FLJ14084AAAF	167	18.44	[TMEM35]
FLJ14103AAAF	182	20.64	[CXorf36]
FLJ14104AAAF	754	83.29	[EPS15L1]
FLJ14114AAAF	110	12.92	[SND1-IT1]
FLJ14117AAAF	185	20.83	[IFT22]
FLJ14129AAAF	374	40.52	[ZNF696]
FLJ14140AAAF	338	36.34	[ZFP36L1]
FLJ14154AAAF	242	27.45	[NAA60]
FLJ14158AAAF	174	19.29	[C20orf27]
FLJ14164AAAF	256	28.73	[NUDCD3]
FLJ14165AAAF	239	27.56	[MRTO4]
FLJ14168AAAF	247	28.24	[YWHAG]
FLJ14170AAAF	317	34.71	[MC1R]
FLJ14184AAAF	442	49.64	[TULP3]
FLJ14189AAAF	958	107.49	[SLITRK5]
FLJ14191AAAF	533	61.25	[CXorf57]
FLJ14196AAAF	684	75.44	[HBS1L]
FLJ14207AAAN	547	60.7	[TJAP1]
FLJ14217AAAF	436	47.54	[WIPI2]
FLJ14218AAAF	456	50.2	[SYT12]
FLJ14220AAAF	258	28.55	[SYNDIG1]
FLJ14222AAAF	749	84.92	[NOC2L]
FLJ14226AAAF	653	72.95	[RFX2]
FLJ14229AAAF	217	24.31	[COQ7]
FLJ14235AAAN	696	79.64	[PPP4R3A]
FLJ14254AAAN	366	40.15	[CTBP2]
FLJ14256AAAF	366	42.44	[USP46]
FLJ14257AAAF	387	42.65	[GMPPB]
FLJ14264AAAF	357	40.28	[AUNIP]
FLJ14276AAAF	322	32.98	[CXXC5]
FLJ14280AAAF	257	26.27	[C10orf95]
FLJ14297AAAF	156	17.38	[ABCA11P]
FLJ14299AAAF	590	58.22	[ZNF703]
FLJ14340AAAF	282	30.67	[ATF5]
FLJ14345AAAF	613	70.23	[ZNF665]
FLJ14356AAAF	627	72.86	[ZNF442]
FLJ14357AAAF	554	63.88	[DNAJC1]
FLJ14363AAAF	450	49.61	[MXRA8]
FLJ14367AAAF	116	13.3	[SPX]
FLJ14369AAAF	179	20.37	[ARL5B]
FLJ14385AAAF	322	36.06	[PBK]
FLJ14393AAAF	464	52.73	[MINA]
FLJ14398AAAF	515	58.81	[MTA3]
FLJ14399AAAF	351	37.57	[TMEM25]
FLJ14400AAAF	538	62.23	[CLPTM1L]
FLJ14403AAAF	323	35.6	[QKI]

FLJ14409AAAF	165	18.63	[PPIL3]
FLJ14418AAAF	323	35.85	[MAFB]
FLJ14428AAAF	301	33.35	[HAVCR2]
FLJ14443AAAF	452	47.21	[GORASP2]
FLJ14445AAAF	214	23.82	[RHOJ]
FLJ14448AAAF	1311	147.89	[ZNF521]
FLJ14450AAAF	585	64.99	[EIF2A]
FLJ14451AAAF	434	46.05	[ZC3H10]
FLJ14454AAAF	797	88.91	[GPR128]
FLJ14456AAAF	1287	147.18	[USP47]
FLJ14457AAAF	400	45.94	[ZNF514]
FLJ14460AAAF	239	26.78	[TSPAN9]
FLJ14461AAAF	439	49.04	[DBNL]
FLJ14464AAAF	1025	109.88	[PARP10]
FLJ14466AAAN	238	26.15	[ORAI1]
FLJ14467AAAF	452	50.83	[SCPEP1]
FLJ14469AAAF	942	104.46	[ITIH5]
FLJ14472AAAF	479	53.93	[DDX19B]
FLJ14476AAAF	347	38.17	[LRG1]
FLJ14478AAAN	245	27.8	[ZNF397]
FLJ14479AAAF	356	37.98	[CDC42EP4]
FLJ14480AAAF	569	62.43	[EEPD1]
FLJ14481AAAN	182	20.13	[DCTN5]
FLJ14483AAAF	273	29.49	[CXCL16]
FLJ14486AAAF	511	54.01	[ZBTB45]
FLJ14488AAAF	364	39.81	[SLC35C1]
FLJ14491AAAF	710	79.14	[SLC44A4]
FLJ14494AAAF	377	42.23	[RPUSD4]
FLJ14495AAAF	352	39.92	[SYAP1]
FLJ14496AAAN	886	95.46	[SCAP]
FLJ14497AAAF	373	40.53	[AIFM2]
FLJ14499AAAF	356	38.56	[STOML2]
FLJ14500AAAF	901	102.03	[RNF40]
FLJ14503AAAF	732	81.96	[MAP7D2]
FLJ14507AAAF	879	100.22	[EXOC1]
FLJ14511AAAF	416	47.09	[ALG2]
FLJ14515AAAF	362	43.16	[PTPLAD1]
FLJ14518AAAF	784	86.35	[RIPK4]
FLJ14520AAAN	102	11.8	[SPCS1]
FLJ14522AAAF	1504	166.69	[NISCH]
FLJ14525AAAF	327	36.35	[C1orf198]
FLJ14549AAAF	725	80.39	[ZSCAN10]
FLJ14569AAAF	518	57.52	[VWA9]
FLJ14575AAAN	302	33.64	[BTBD2]
FLJ14576AAAF	521	56.85	[ARFGAP2]
FLJ14577AAAF	447	49.34	[ITFG2]
FLJ14583AAAF	706	77.02	[TRO]
FLJ14585AAAF	204	22.43	[PPCDC]

FLJ14592AAAF	390	44.07	[PIPOX]
FLJ14611AAAF	371	41.94	[GLT8D1]
FLJ14614AAAF	366	40.92	[LEFTY2]
FLJ14617AAAF	396	44.72	[OLA1]
FLJ14618AAAF	287	32.65	[BSCL2]
FLJ14619AAAF	271	29.67	[SRPRB]
FLJ14622AAAF	423	48.56	[AP1M1]
FLJ14625AAAF	315	35.41	[SLC25A32]
FLJ14628AAAF	227	25.57	[AK3]
FLJ14629AAAF	723	81.79	[OSBPL9]
FLJ14633AAAF	113	11.9	[C4orf46]
FLJ14640AAAF	536	62.1	[CEP89]
FLJ14651AAAF	628	68.03	[PIAS3]
FLJ14652AAAF	359	38.95	[RNF146]
FLJ14654AAAF	428	48.49	[CYP26A1]
FLJ14655AAAF	819	92	[IWS1]
FLJ14659AAAF	581	65.92	[ZNF613]
FLJ14661AAAF	803	87.57	[ARHGAP17]
FLJ14664AAAF	843	96.57	[TTC27]
FLJ14667AAAF	571	63.64	[TESK2]
FLJ14668AAAF	138	15.64	[FAM136A]
FLJ14670AAAF	589	62.55	[UBQLN1]
FLJ14680AAAF	614	66.45	[RABL6]
FLJ14681AAAF	555	63.54	[TMEM87B]
FLJ14685AAAF	311	35.24	[JKAMP]
FLJ14686AAAF	548	63.79	[ZNF382]
FLJ14687AAAF	493	56.29	[TRIM5]
FLJ14688AAAF	147	17.2	[PLGRKT]
FLJ14690AAAF	612	68.14	[ITFG1]
FLJ14691AAAF	406	44.35	[LHX2]
FLJ14695AAAF	518	59.3	[TRIM41]
FLJ14697AAAF	331	37.42	[SLC35B4]
FLJ14699AAAF	564	62.26	[CYP2S1]
FLJ14700AAAF	492	52.03	[GTPBP3]
FLJ14707AAAF	489	55.15	[SPRTN]
FLJ14710AAAF	575	65.59	[ZNF587]
FLJ14714AAAF	509	58.37	[JADE1]
FLJ14719AAAF	833	94.75	[VEPH1]
FLJ14720AAAF	414	46.62	[WWOX]
FLJ14721AAAF	452	46.88	[FAM222A]
FLJ14729AAAF	721	79.79	[BBS2]
FLJ14731AAAF	418	47.03	[STRADB]
FLJ14732AAAF	488	57.49	[CCDC77]
FLJ14733AAAF	254	28.57	[ORAI2]
FLJ14735AAAF	500	53.31	[LRP11]
FLJ14736AAAF	371	42.57	[CDCA7]
FLJ14739AAAF	630	69.66	[BBS1]
FLJ14740AAAF	958	107.54	[TXNDC11]

FLJ14741AAAF	793	90.98	[DNAJC10]
FLJ14743AAAF	742	85.42	[STRIP1]
FLJ14750AAAF	568	63.28	[KLHL12]
FLJ14754AAAF	611	70.68	[ZNF799]
FLJ14755AAAF	734	81.7	[CPXM1]
FLJ14757AAAF	302	33.25	[STC2]
FLJ14758AAAF	488	56.4	[TRIM6]
FLJ14759AAAF	363	39.92	[NDRG3]
FLJ14768AAAF	496	51.99	[FIZ1]
FLJ14769AAAF	686	76.86	[CIRH1A]
FLJ14771AAAF	253	27.78	[TOR2A]
FLJ14773AAAF	452	50.22	[ZNF672]
FLJ14776AAAF	270	30.67	[CECR1]
FLJ14777AAAF	187	21	[GPX7]
FLJ14779AAAF	418	49.22	[ZNF566]
FLJ14782AAAF	974	110.5	[EXOC4]
FLJ14784AAAF	478	52.09	[DIRC2]
FLJ14791AAAF	736	83.39	[P3H1]
FLJ14797AAAF	667	76.09	[TUBGCP4]
FLJ14798AAAF	308	33.92	[TOMM40L]
FLJ14804AAAF	774	85.52	[MMS19]
FLJ14805AAAF	393	44.88	[CHID1]
FLJ14808AAAF	839	96.8	[ZKSCAN5]
FLJ14809AAAF	248	27.71	[TSPAN18]
FLJ14810AAAN	198	21.56	[FIBCD1]
FLJ14819AAAF	632	68.93	[HIF3A]
FLJ14825AAAF	380	43.33	[C8orf76]
FLJ14827AAAF	269	28.62	[RITA1]
FLJ14829AAAN	865	95.16	[PARD3]
FLJ14840AAAF	396	43.95	[ZFYVE19]
FLJ14844AAAF	158	17.27	[BTF3L4]
FLJ14847AAAF	510	55.38	[PVRL4]
FLJ14848AAAF	617	70.64	[VPS33B]
FLJ14850AAAF	515	59.44	[FAR2]
FLJ14871AAAF	285	31.66	[CORO6]
FLJ14872AAAF	468	52.81	[SCIN]
FLJ14873AAAF	456	50.37	[BLNK]
FLJ14874AAAF	381	43.86	[GPR34]
FLJ14878AAAF	358	41.46	[GPR87]
FLJ14882AAAF	545	62.94	[TM9SF3]
FLJ14886AAAF	352	39	[HSH2D]
FLJ14888AAAF	378	41.6	[WDR73]
FLJ14891AAAF	122	13.89	[SCOC]
FLJ14895AAAF	539	60.79	[BCO2]
FLJ14902AAAF	373	41.15	[LACTB]
FLJ14904AAAF	434	49.22	[MAEL]
FLJ14905AAAF	626	72.2	[SAMHD1]
FLJ14910AAAN	436	51.43	[KIF15]

FLJ14917AAAF	654	74.15	[SERAC1]
FLJ14918AAAF	272	30.57	[TCEA2]
FLJ14919AAAF	487	54.03	[SLC38A1]
FLJ14935AAAF	737	81.95	[PAMR1]
FLJ14938AAAF	483	55.87	[CCDC47]
FLJ14948AAAF	504	56.31	[ATG16L1]
FLJ14970AAAF	488	56.86	[TRIM34]
FLJ14971AAAF	400	44.88	[TMEM43]
FLJ14972AAAF	531	57.44	[ZGPAT]
FLJ14973AAAF	247	26.84	[APH1A]
FLJ14974AAAN	1006	115.3	[RANBP17]
FLJ14975AAAF	690	77.18	[CALCOCO1]
FLJ14978AAAF	927	104.8	[GBA2]
FLJ14980AAAF	613	68.66	[METTL13]
FLJ14981AAAF	451	48.58	[MPND]
FLJ14984AAAF	672	73.68	[STRBP]
FLJ14987AAAF	200	22.54	[COMMD7]
FLJ14988AAAF	285	31.43	[GSG1]
FLJ14991AAAF	583	66.73	[NUDCD1]
FLJ14993AAAF	430	46.11	[RELT]
FLJ14994AAAF	208	24.48	[CCDC25]
FLJ14995AAAF	283	31.94	[KCTD15]
FLJ14996AAAF	550	60.63	[SYTL1]
FLJ16003AAAF	213	25	[CLEC4C]
FLJ16006AAAF	553	59.63	[SLC22A12]
FLJ16007AAAN	525	60.72	[CYP4V2]
FLJ16008AAAF	372	42.63	[CYP27C1]
FLJ16012AAAF	510	57.62	[MMP28]
FLJ16014AAAF	403	44.9	[CPA5]
FLJ16016AAAF	528	61.83	[CATSPER2]
FLJ16018AAAF	234	26.17	[RSPO4]
FLJ16022AAAF	590	67.16	[LRRTM4]
FLJ16024AAAF	671	73.52	[GPAT2]
FLJ16026AAAF	441	49.9	[PPARD]
FLJ16031AAAF	556	63.99	[GALNT13]
FLJ16041AAAF	433	49.28	[GPR22]
FLJ16044AAAF	865	96.96	[PLA2G4F]
FLJ16046AAAF	438	49.41	[TMPRSS11F]
FLJ16047AAAF	336	38.41	[GPR82]
FLJ16048AAAF	419	46.64	[GPR151]
FLJ16049AAAF	617	66.97	[SLC2A12]
FLJ16051AAAF	483	53.24	[SLC36A2]
FLJ16052AAAF	172	19.4	[C1orf186]
FLJ16054AAAF	267	29.7	[MS4A10]
FLJ16055AAAF	681	74.09	[SLC5A9]
FLJ16056AAAF	413	45.32	[FCRL6]
FLJ16060AAAF	430	49.33	[GCNT7]
FLJ16063AAAF	266	29.65	[ZACN]

FLJ16066AAAF	337	38.25	[OXGR1]
FLJ16069AAAN	426	48.27	[ARSI]
FLJ16072AAAF	473	50.6	[ZBTB9]
FLJ16075AAAF	210	22.89	[RNF135]
FLJ16076AAAF	694	77.96	[HACE1]
FLJ16079AAAF	670	73.59	[ZNF526]
FLJ16082AAAF	451	50.86	[LIPH]
FLJ16084AAAF	486	52.93	[GPRC5C]
FLJ16085AAAF	203	23.26	[RASL10B]
FLJ16086AAAN	1023	117.07	[IQGAP3]
FLJ16087AAAF	892	101.64	[DPP9]
FLJ16088AAAF	572	64.04	[TMPRSS7]
FLJ16089AAAF	771	84.05	[SLC7A14]
FLJ16090AAAF	529	58.56	[GPR161]
FLJ16092AAAF	330	35.25	[GPBAR1]
FLJ16096AAAF	447	50.42	[NR1H3]
FLJ16098AAAF	1075	116.79	[HIPK1]
FLJ16101AAAF	391	44.02	[MCM9]
FLJ16105AAAF	621	68.98	[TTBK1]
FLJ16106AAAN	323	35.16	[STARD9]
FLJ16107AAAF	567	62.92	[WEE2]
FLJ16109AAAF	324	36.08	[OR56B1]
FLJ16111AAAF	343	38.17	[MRGPRF]
FLJ16113AAAF	492	56.2	[KRBA2]
FLJ16114AAAF	422	45.75	[PLIN3]
FLJ16119AAAF	187	21.59	[DHFRL1]
FLJ16123AAAF	402	45.37	[ZCCHC12]
FLJ16133AAAF	276	30.57	[ZNF688]
FLJ16139AAAF	339	36.52	[DBX2]
FLJ16141AAAF	422	48.5	[ZNF620]
FLJ16142AAAF	446	50.07	[NFIB]
FLJ16146AAAN	743	81.41	[AP4M1]
FLJ16151AAAF	715	76.4	[ZDHHC8]
FLJ16162AAAF	752	85.26	[RRM1]
FLJ16163AAAF	336	39.02	[AK9]
FLJ16165AAAF	438	50.47	[PAPL]
FLJ16178AAAF	492	55.31	[ATG14]
FLJ16189AAAF	280	31.89	[FHL1]
FLJ16205AAAN	1075	121.13	[POLR3B]
FLJ16206AAAN	1077	119.79	[ADCY4]
FLJ16207AAAF	495	56.56	[MBOAT1]
FLJ16210AAAF	454	51.87	[TMX3]
FLJ16211AAAF	247	26.11	[PGRMC2]
FLJ16213AAAF	725	78.4	[VWA2]
FLJ16214AAAF	678	72.76	[PCSK9]
FLJ16218AAAF	412	45.26	[ZNF704]
FLJ16219AAAF	446	50.74	[ZNF70]
FLJ16225AAAF	443	50.62	[ZNF302]

FLJ16228AAAF	974	107.15	[ERN2]
FLJ16236AAAF	327	35.17	[NAIF1]
FLJ16237AAAF	445	51.48	[AGMO]
FLJ16241AAAF	142	15.76	[LOC100133315]
FLJ16251AAAF	627	66.4	[SPHK2]
FLJ16262AAAF	340	36.78	[RAD51]
FLJ16264AAAF	457	53	[ZNF140]
FLJ16265AAAF	698	79.04	[CNGA3]
FLJ16268AAAF	317	35.53	[UPP2]
FLJ16269AAAF	563	63.4	[PHF19]
FLJ16275AAAF	626	70.48	[FRRS1]
FLJ16278AAAF	994	108.86	[RNF111]
FLJ16295AAAF	157	16.76	[CDCP2]
FLJ16302AAAN	695	72.94	[NOTCH4]
FLJ16304AAAF	667	71.28	[TCF4]
FLJ16306AAAF	471	54.04	[MAPKAPK5]
FLJ16310AAAF	354	38.19	[UBE2Z]
FLJ16323AAAF	141	15.49	[LOC100506422]
FLJ16333AAAF	313	35.25	[SDR9C7]
FLJ16334AAAF	158	18.26	[ZNF780A]
FLJ16338AAAF	373	42.05	[GLUL]
FLJ16339AAAF	562	65.99	[CCDC81]
FLJ16340AAAF	514	57.65	[HBP1]
FLJ16346AAAF	852	96.65	[ZSCAN29]
FLJ16347AAAF	129	14.59	[AKR1C8P]
FLJ16353AAAF	300	34.73	[ZNF705A]
FLJ16356AAAF	551	63.63	[TRAF3IP3]
FLJ16358AAAF	170	19.12	[ISG20]
FLJ16362AAAF	523	57.29	[KRT71]
FLJ16366AAAF	341	39	[ETV7]
FLJ16369AAAF	300	32.52	[PSMB11]
FLJ16371AAAF	182	19.45	[CDIPT]
FLJ16386AAAF	633	72.43	[GBP6]
FLJ16387AAAF	779	86.45	[ZNF438]
FLJ16403AAAN	307	35.21	[RNF217]
FLJ16410AAAF	461	52.8	[ZFP2]
FLJ16413AAAF	425	46.66	[FAM46B]
FLJ16417AAAF	986	108.7	[ZNF574]
FLJ16421AAAF	1167	129.08	[TTBK2]
FLJ16428AAAF	215	24.89	[HMGB1]
FLJ16430AAAN	848	91.19	[AGAP2]
FLJ16443AAAN	851	98.66	[KALRN]
FLJ16449AAAN	301	33.37	[UNC5A]
FLJ16450AAAF	166	18.88	[CCNJL]
FLJ16454AAAF	565	61.23	[SLC1A2]
FLJ16458AAAF	465	53.6	[GABRG1]
FLJ16460AAAF	563	63.75	[PXK]
FLJ16462AAAF	618	71.46	[ZNF569]

FLJ16464AAAF	908	102.88	[GEN1]
FLJ16465AAAF	637	74.99	[ZNF540]
FLJ16470AAAF	788	85.52	[NFKB1]
FLJ16477AAAF	344	39.39	[LPAR6]
FLJ16478AAAF	361	39.95	[ETV3L]
FLJ16482AAAN	238	26.9	[SMAD7]
FLJ16486AAAF	631	67.37	[FOXO3]
FLJ16490AAAF	1099	122.33	[BNC2]
FLJ16493AAAF	852	96.61	[MACC1]
FLJ16496AAAF	553	61.76	[STXBP4]
FLJ16498AAAF	422	48.37	[ZNF550]
FLJ16508AAAF	639	73.65	[ZNF182]
FLJ16509AAAF	1043	117.53	[ZSCAN20]
FLJ16510AAAF	749	85.39	[FSD2]
FLJ16512AAAF	466	52.14	[GCK]
FLJ16515AAAF	808	93.15	[ZNF841]
FLJ16516AAAN	301	33.98	[AEBP2]
FLJ16517AAAF	250	27.08	[LIN28B]
FLJ16519AAAF	831	94.27	[PRICKLE1]
FLJ16520AAAF	672	78.01	[ZNF605]
FLJ16527AAAF	374	43.14	[ZNF793]
FLJ16532AAAF	485	56.11	[ZNF639]
FLJ16535AAAF	709	77.45	[TMCC2]
FLJ16539AAAF	763	86.77	[XIRP2]
FLJ16541AAAF	835	89.36	[WIZ]
FLJ16543AAAN	596	66.21	[TRIO]
FLJ16544AAAN	569	58.83	[MEX3B]
FLJ16545AAAF	302	32.37	[LINC00322]
FLJ16546AAAF	748	84.56	[ABLIM1]
FLJ16548AAAF	480	51.62	[BCL6B]
FLJ16557AAAF	421	45.48	[ZNF385B]
FLJ16564AAAF	904	104.06	[SYNE3]
FLJ16569AAAN	486	52.67	[RNF19B]
FLJ16572AAAF	492	54.65	[ZNF517]
FLJ16575AAAF	367	41.62	[TSSK1B]
FLJ16579AAAF	472	52.67	[WDR88]
FLJ16580AAAF	466	51.45	[SLCO6A1]
FLJ16581AAAF	263	29.85	[RFPL4B]
FLJ16587AAAF	1216	130.03	[SPOCD1]
FLJ16590AAAN	1893	215.04	[CDK5RAP2]
FLJ16591AAAF	396	44.26	[DCAF4L1]
FLJ16594AAAN	1189	132.86	[TDRD1]
FLJ16596AAAN	1170	132.21	[WDR35]
FLJ16598AAAF	1340	150.97	[YTHDC2]
FLJ16604AAAF	1462	158.02	[ASXL1]
FLJ16612AAAF	1119	126.3	[ADCY3]
FLJ16615AAAF	970	108.96	[SLC26A8]
FLJ16619AAAN	1782	197.44	[SIPA1L1]

FLJ16627AAAF	727	83.92	[PREPL]
FLJ16630AAAF	418	46.44	[SERPINH1]
FLJ16633AAAF	530	61.8	[ZNF680]
FLJ16636AAAF	699	80.9	[ZNF782]
FLJ16637AAAF	319	35.77	[ZNF346]
FLJ16639AAAF	814	92.5	[KIF6]
FLJ16640AAAF	503	59.07	[ZNF730]
FLJ16641AAAF	388	45.15	[LEKR1]
FLJ16645AAAF	788	89.37	[MAP3K15]
FLJ16649AAAF	241	27.08	[PRSS58]
FLJ16650AAAF	513	59.08	[ZNF26]
FLJ16654AAAF	805	88.7	[CLCN7]
FLJ16656AAAF	589	64.08	[ZBTB46]
FLJ16658AAAF	455	50.25	[SLC36A3]
FLJ16664AAAF	477	52.65	[TRIM72]
FLJ16670AAAF	283	30.7	[VDAC1]
FLJ16677AAAF	363	40.2	[C3P1]
FLJ16680AAAF	555	62.03	[ACOT12]
FLJ16683AAAF	291	32.11	[STOML3]
FLJ16700AAAF	1052	120.89	[KCNT2]
FLJ16708AAAF	411	45.01	[STAC2]
FLJ16715AAAF	1013	105.11	[ZBTB4]
FLJ16716AAAF	266	29.54	[DENND3]
FLJ16721AAAF	350	39.15	[ZNF561]
FLJ16732AAAF	345	38.59	[CDK15]
FLJ16733AAAF	435	47.91	[RXRA]
FLJ16736AAAF	779	87.35	[GRM4]
FLJ16737AAAF	337	38.33	[ABHD6]
FLJ16740AAAF	436	49.61	[NIM1K]
FLJ16741AAAF	199	22.48	[DIRAS2]
FLJ16743AAAF	460	50.78	[CREB3L2]
FLJ16745AAAF	421	47.57	[TMPRSS11A]
FLJ16757AAAF	1586	177.86	[MTCL1]
FLJ16759AAAF	1331	141.67	[IL16]
FLJ16760AAAF	313	35.65	[CDK10]
FLJ16763AAAF	766	84.8	[BRSK2]
FLJ16769AAAN	1288	147.02	[LRRK2]
FLJ16774AAAF	422	46.4	[NR2E1]
FLJ16777AAAF	802	92.2	[CDC5L]
FLJ16783AAAF	600	68.37	[ZNF543]
FLJ16786AAAF	1176	123.14	[CRB2]
FLJ16793AAAF	369	40.62	[CCNI2]
FLJ16797AAAF	328	36.42	[OR13A1]
FLJ16801AAAF	594	66.48	[ZNF133]
FLJ16803AAAF	695	80.45	[ZNF607]
FLJ16804AAAF	164	18.24	[PPIAL4C]
FLJ16809AAAF	948	104.67	[PKN1]
FLJ16810AAAN	542	63.1	[ZNF136]

FLJ16812AAAF	422	45.32	[GFI1]
FLJ16818AAAF	652	70.42	[NR4A1]
FLJ16822AAAN	924	104.26	[ADCY2]
FLJ16825AAAF	471	52.05	[ADAMTSL5]
FLJ16828AAAF	313	35.34	[OR1J2]
FLJ16830AAAF	1886	215.26	[CHD4]
FLJ17629SAAF	250	27.43	[RUNX1]
FLJ20003AAAF	385	44.3	[NSMCE4A]
FLJ20007AAAF	458	51.78	[FICD]
FLJ20013AAAF	302	33.84	[ALKBH4]
FLJ20022AAAF	313	34.98	[ITLN1]
FLJ20024AAAN	357	40.48	[FAM45A]
FLJ20025AAAF	159	17.28	[TMEM88]
FLJ20026AAAF	338	37.51	[MOV10L1]
FLJ20027AAAF	375	41.79	[DNAJB12]
FLJ20036AAAF	580	61.11	[CDKN2AIP]
FLJ20040AAAN	234	26.09	[KCTD5]
FLJ20041AAAF	1016	113.23	[TRPM4]
FLJ20045AAAF	208	23.65	[MED18]
FLJ20049AAAF	319	36.33	[RWDD2B]
FLJ20051AAAF	204	22.88	[ARL15]
FLJ20057AAAF	382	43.94	[ATP6V1C1]
FLJ20065AAAF	919	101.35	[CLCA4]
FLJ20068AAAF	387	43.13	[PPP1R9A]
FLJ20080AAAF	909	99.46	[AFTPH]
FLJ20084AAAF	382	42.86	[QPCTL]
FLJ20089AAAF	418	46.74	[ING3]
FLJ20090AAAF	415	46.97	[BRE]
FLJ20093AAAF	420	44.71	[ANKRD10]
FLJ20094AAAF	170	18.44	[ZCCHC10]
FLJ20103AAAF	154	17.09	[USP48]
FLJ20130AAAF	184	20.83	[NUP62CL]
FLJ20132AAAN	466	52.01	[BEST2]
FLJ20133AAAF	115	12.51	[PLAC8]
FLJ20142AAAF	497	56.22	[GDAP2]
FLJ20174AAAF	827	93.88	[SIDT1]
FLJ20177AAAF	453	51.26	[SMPDL3A]
FLJ20184AAAF	219	25.51	[ARHGEF38]
FLJ20186AAAF	197	22.98	[DEF8]
FLJ20189AAAN	239	27.32	[ANKRD49]
FLJ20190AAAF	330	38	[PAQR5]
FLJ20192AAAF	363	37.87	[POGZ]
FLJ20200AAAF	478	51.83	[FAM83E]
FLJ20202AAAF	391	44.93	[FAM46C]
FLJ20206AAAF	441	48.65	[C9orf156]
FLJ20208AAAF	209	23.14	[PGPEP1]
FLJ20210AAAF	782	88.64	[CCHCR1]
FLJ20214AAAF	710	76.6	[NCL]

FLJ20217AAAF	267	28.03	[MS4A12]
FLJ20221AAAF	432	48.85	[TADA3]
FLJ20225AAAF	227	24.15	[RNF186]
FLJ20226AAAF	449	49.2	[MFSD11]
FLJ20234AAAF	449	49.65	[STAP2]
FLJ20244AAAF	659	72.23	[TRMT1]
FLJ20245AAAF	423	46.94	[TOR4A]
FLJ20247AAAF	211	23.59	[RHOF]
FLJ20248AAAF	400	45.41	[LRRFIP2]
FLJ20251AAAN	145	16.51	[PPP1R14D]
FLJ20255AAAF	314	34.83	[TMEM104]
FLJ20257AAAN	220	24.95	[MEPCE]
FLJ20258AAAF	596	66.52	[EPS8L1]
FLJ20261AAAF	525	55.17	[KRT24]
FLJ20274AAAF	353	39.28	[THUMPD1]
FLJ20278AAAF	142	16.66	[MRPL42]
FLJ20279AAAF	308	35.14	[ZDHHC7]
FLJ20280AAAF	216	24.19	[DCAF16]
FLJ20287AAAF	929	105.69	[TEX10]
FLJ20291AAAF	425	49.65	[CWC25]
FLJ20292AAAF	460	52.34	[DCTN4]
FLJ20297AAAF	798	90.13	[SMPD4]
FLJ20300AAAF	325	35.82	[LPPR1]
FLJ20308AAAF	378	42.55	[ALKBH5]
FLJ20317AAAN	431	46.93	[A1CF]
FLJ20330AAAF	390	43.16	[GFOD1]
FLJ20333AAAF	706	80.48	[G2E3]
FLJ20334AAAF	296	34.52	[ELOVL2]
FLJ20340AAAF	322	35.43	[LAX1]
FLJ20342AAAF	579	65.07	[CDKAL1]
FLJ20344AAAF	166	19.47	[KRBOX4]
FLJ20346AAAF	581	66.45	[ENOX2]
FLJ20348AAAN	299	32.67	[SIRT5]
FLJ20352AAAF	415	45.07	[ACAD8]
FLJ20355AAAF	946	110.25	[SNX14]
FLJ20371AAAF	526	57.27	[FLVCR2]
FLJ20372AAAF	561	64.37	[ASNS]
FLJ20374AAAF	199	20.66	[RPP25]
FLJ20377AAAN	651	69.51	[CDHR5]
FLJ20380AAAF	219	24.68	[CIDEB]
FLJ20396AAAF	183	20.44	[CMTM6]
FLJ20397AAAN	235	26.33	[DNAAF5]
FLJ20398AAAF	157	17.78	[UBL4A]
FLJ20399AAAF	493	55.04	[DUS2]
FLJ20400WAAF	121	13.31	[CHTF8]
FLJ20402AAAF	403	45.89	[GSDMB]
FLJ20405AAAF	475	52.99	[RRNAD1]
FLJ20406AAAF	295	31.23	[LIME1]

FLJ20407AAAF	100	11.46	[C8orf59]
FLJ20408AAAN	280	29.54	[HES1]
FLJ20409AAAF	505	54.99	[SLC16A5]
FLJ20411AAAF	335	36.43	[OSGEP]
FLJ20412AAAF	201	23.38	[NT5C]
FLJ20419AAAF	238	27.11	[UBE2R2]
FLJ20420AAAF	227	26.15	[CHCHD3]
FLJ20421AAAN	359	38.68	[IMPAD1]
FLJ20422AAAF	479	53.62	[TMEM161A]
FLJ20423AAAN	244	26.91	[GTPBP2]
FLJ20424AAAF	363	42.41	[HAUS4]
FLJ20425AAAF	379	43.63	[LYAR]
FLJ20428AAAF	671	75.73	[ZNF251]
FLJ20429AAAF	234	26.28	[RAB20]
FLJ20430AAAF	460	51.61	[WRAP73]
FLJ20433AAAF	252	26.33	[EXD3]
FLJ20434AAAN	115	13.08	[FAM104B]
FLJ20437AAAF	549	63.49	[MTMR9]
FLJ20442AAAF	150	16.6	[DUSP23]
FLJ20445AAAF	278	31.23	[MARCH5]
FLJ20446AAAF	363	38.95	[ADPRHL2]
FLJ20449AAAF	425	46.92	[SOHLH2]
FLJ20450AAAN	518	58.28	[SARS2]
FLJ20451AAAN	258	29.86	[MRPL39]
FLJ20453AAAF	284	29.83	[VSIG2]
FLJ20455AAAF	245	27.63	[OCIAD1]
FLJ20457AAAF	181	20.38	[FAM206A]
FLJ20460AAAF	602	66.32	[GLS2]
FLJ20461SAAF	388	45.81	[DNAJC28]
FLJ20462AAAF	315	34.46	[RPH3AL]
FLJ20473AAAF	471	50.81	[SLC41A3]
FLJ20476AAAF	637	73.22	[MTMR12]
FLJ20478AAAN	545	58.54	[RTEL1]
FLJ20479AAAF	153	17.2	[NHP2]
FLJ20484AAAF	251	28.45	[MRPL16]
FLJ20487AAAF	166	19.6	[SDHAF2]
FLJ20497AAAF	197	22.51	[UBE2T]
FLJ20498AAAF	424	46.56	[ELP4]
FLJ20500AAAF	232	25.37	[DDIT4]
FLJ20501AAAF	140	14.96	[ACOT13]
FLJ20502AAAF	183	21.12	[COMMD8]
FLJ20503AAAF	287	32.5	[TRNAU1AP]
FLJ20504AAAF	993	110.92	[VARS2]
FLJ20507AAAF	238	25.83	[TMEM127]
FLJ20508AAAF	203	23.37	[C1orf109]
FLJ20509AAAF	207	23.38	[PARP16]
FLJ20510AAAF	1062	120.52	[NLRP2]
FLJ20511AAAF	149	17.01	[TXNL4B]

FLJ20512AAAF	188	19.66	[TMEM160]
FLJ20514AAAF	242	28.64	[GEMIN8]
FLJ20516AAAF	301	34.52	[TIPIN]
FLJ20517AAAF	548	61.11	[UCKL1]
FLJ20521AAAF	460	51.53	[IRAK4]
FLJ20527AAAF	242	26.64	[CT55]
FLJ20531AAAF	474	51.9	[ZNF692]
FLJ20534AAAF	346	39.41	[C4orf27]
FLJ20536AAAF	300	33.32	[TPGS2]
FLJ20539AAAF	774	83.18	[TMEM132A]
FLJ20545AAAF	334	36.59	[WDR5]
FLJ20551AAAF	304	33.51	[SLC25A38]
FLJ20552AAAF	311	33.86	[RNF126]
FLJ20555AAAF	371	40.21	[SLC35F6]
FLJ20556AAAF	162	17.39	[HRASLS2]
FLJ20558AAAF	574	64.14	[C2orf42]
FLJ20559AAAF	199	23.19	[NMRK1]
FLJ20561AAAF	311	35.92	[CLN6]
FLJ20565AAAF	328	37.03	[PINX1]
FLJ20567AAAF	167	17.94	[ISCU]
FLJ20568AAAF	138	15.29	[HCFC1R1]
FLJ20569AAAF	285	33.01	[MIOX]
FLJ20578AAAN	285	32.69	[EAPP]
FLJ20580AAAF	160	18.05	[C1orf123]
FLJ20583AAAF	412	46.75	[VPS13B]
FLJ20587AAAF	309	34.59	[TOMM34]
FLJ20588AAAF	453	49.51	[DLGAP4]
FLJ20589AAAF	193	22.31	[HPCAL1]
FLJ20591AAAF	245	26.35	[EXOSC4]
FLJ20593AAAF	302	34.17	[ST6GALNAC4]
FLJ20594AAAF	206	23.64	[MRPL22]
FLJ20595AAAF	150	16.74	[ZSCAN2]
FLJ20599AAAF	250	28.61	[GOSR1]
FLJ20600AAAF	593	63.54	[GRN]
FLJ20602AAAF	228	26.75	[GID8]
FLJ20604AAAF	459	48.81	[OXSM]
FLJ20606AAAF	497	55.73	[TRMT6]
FLJ20608AAAN	199	22.35	[AURKAIP1]
FLJ20609AAAF	414	46.19	[SUPT7L]
FLJ20611AAAF	113	12.8	[CT83]
FLJ20615AAAF	349	40.21	[HIF1AN]
FLJ20619AAAF	372	41.74	[TTC22]
FLJ20621AAAF	304	33.26	[HMBS]
FLJ20622AAAF	141	15.4	[DUT]
FLJ20624AAAN	391	43.83	[PAK1IP1]
FLJ20625AAAF	161	17.74	[LAMTOR1]
FLJ20626AAAF	450	48.96	[ZNF446]
FLJ20627AAAF	449	51.62	[RMND1]

FLJ20630AAAF	104	11.42	[INIP]
FLJ20633AAAF	226	25.5	[GSTK1]
FLJ20636AAAF	262	27.9	[ASB9]
FLJ20639AAAF	337	38.76	[CDC37L1]
FLJ20641AAAN	498	55.81	[PARPBP]
FLJ20643AAAF	290	32.36	[PIH1D1]
FLJ20644AAAF	453	53.31	[PPP2R3C]
FLJ20646AAAF	123	13.11	[CRB3]
FLJ20649AAAF	69	7.96	[ATP5I]
FLJ20650AAAF	428	48.89	[ACP6]
FLJ20652AAAF	439	49.9	[ACOT9]
FLJ20656AAAF	316	35.96	[JMJD7]
FLJ20657AAAF	608	68.18	[NPLOC4]
FLJ20665AAAF	314	35.81	[RMDN1]
FLJ20668AAAF	272	30.87	[MARCH1]
FLJ20671AAAF	140	16.01	[C14orf119]
FLJ20673AAAF	432	46.96	[PAG1]
FLJ20694AAAF	154	16.98	[LINC00483]
FLJ20695AAAF	305	33.9	[PEX26]
FLJ20718AAAF	594	65.81	[HEATR3]
FLJ20724AAAN	482	52.73	[BTBD1]
FLJ20731AAAF	211	24.17	[FKBP14]
FLJ20732AAAF	913	105.34	[EIF3C]
FLJ20733AAAF	888	98.13	[MOCOS]
FLJ20735AAAF	730	79.48	[DTL]
FLJ20746AAAF	555	61.82	[SMOX]
FLJ20752AAAF	643	72.15	[ASNSD1]
FLJ20759AAAF	675	74.18	[SLC25A13]
FLJ20772AAAN	448	50.14	[TRMT12]
FLJ20778AAAF	632	68.25	[EPN3]
FLJ20789AAAF	139	15.59	[GSKIP]
FLJ20796AAAF	104	11.95	[LYRM7]
FLJ20809AAAF	427	47.35	[FLOT1]
FLJ20815AAAF	490	54.96	[RSL1D1]
FLJ20819AAAF	207	23.49	[RAB7A]
FLJ20823AAAF	194	20.03	[TIMM22]
FLJ20826AAAF	546	59.58	[AAAS]
FLJ20849AAAF	757	81.15	[CEP68]
FLJ20852AAAF	157	17.11	[RBM3]
FLJ20878AAAF	154	16.65	[PFDN2]
FLJ20899AAAF	127	14.98	[PAIP2]
FLJ20902AAAF	136	14.89	[SNAPIN]
FLJ20916AAAF	139	16.09	[CNIH4]
FLJ20919AAAF	423	48.12	[MRPL37]
FLJ20920AAAF	615	68.12	[ACSF2]
FLJ20938AAAF	84	9.49	[RPS27L]
FLJ20940AAAF	168	18.74	[PPP1R1B]
FLJ20958AAAF	257	27.42	[MCUR1]

FLJ20961AAAF	318	35.18	[DERA]
FLJ20973AAAF	421	46.02	[POLDIP3]
FLJ20989AAAF	229	24.97	[C8orf33]
FLJ20991AAAN	253	27.35	[FAAP100]
FLJ20996AAAF	856	99.01	[LRRC8A]
FLJ20999AAAF	717	79.28	[SAMD4A]
FLJ21007AAAF	651	73.16	[TDRD3]
FLJ21023AAAF	1006	111.23	[PLEKHG5]
FLJ21024AAAN	323	35.95	[NAAA]
FLJ21029AAAF	247	27.55	[TMEM69]
FLJ21032AAAF	256	28.92	[SCD5]
FLJ21035AAAF	122	13.33	[AAMDC]
FLJ21040AAAN	824	94.3	[C16orf62]
FLJ21046AAAF	114	11.78	[PPDPF]
FLJ21049AAAF	173	20.12	[MRPS25]
FLJ21058AAAF	325	35.09	[ZC2HC1A]
FLJ21063AAAF	365	41.18	[RRS1]
FLJ21075AAAF	122	14.53	[C7orf69]
FLJ21076AAAF	63	6.95	[HILPDA]
FLJ21078AAAF	142	15.52	[TOMM22]
FLJ21080AAAF	369	42.63	[SMYD3]
FLJ21093AAAF	140	16.13	[PDZD11]
FLJ21097AAAF	241	26.98	[NRIP3]
FLJ21100AAAF	219	24.57	[CHMP5]
FLJ21101AAAF	305	33.58	[WDR61]
FLJ21103AAAF	351	39.44	[FAM118B]
FLJ21111AAAF	95	10.5	[TIMM13]
FLJ21112AAAF	379	42.19	[PARL]
FLJ21120AAAF	498	54.6	[DLC1]
FLJ21123AAAF	106	12.63	[MRPS33]
FLJ21129AAAF	559	63.32	[DENND1A]
FLJ21144AAAF	373	41.82	[EXO5]
FLJ21146AAAF	454	52.7	[SH2D4A]
FLJ21148AAAF	449	50.72	[SETD6]
FLJ21149AAAF	415	46.28	[PDSS1]
FLJ21161AAAF	130	12.93	[IFI6]
FLJ21169AAAN	363	39.93	[ELAC1]
FLJ21172AAAF	343	38.36	[RBFA]
FLJ21174AAAF	215	24.55	[TCEAL4]
FLJ21180AAAF	529	57.83	[KPNA2]
FLJ21182AAAF	309	33.7	[CNN2]
FLJ21183AAAN	343	38.6	[HLA-C]
FLJ21184AAAF	81	9.37	[NDUFA4]
FLJ21190AAAF	170	18.68	[DCTPP1]
FLJ21195AAAF	168	19.32	[GREM2]
FLJ21227AAAN	409	46.01	[ABHD3]
FLJ21231AAAF	93	10.44	[SH3BGRL3]
FLJ21246AAAF	875	100.11	[ENPP3]

FLJ21267AAAF	356	39.32	[FAM134B]
FLJ21269AAAF	235	26.62	[MYCT1]
FLJ21276AAAF	190	21.01	[LINC00341]
FLJ21279AAAF	739	82.2	[ACSL5]
FLJ21302AAAF	370	42.33	[LRRC19]
FLJ21313AAAF	432	47.9	[GRAMD3]
FLJ21316AAAF	406	46.26	[VMP1]
FLJ21319AAAF	474	52.32	[GPBP1L1]
FLJ21332SAAN	729	80.27	[DDX17]
FLJ21347AAAF	786	87.89	[SPATA20]
FLJ21371AAAF	1061	118.92	[NPR1]
FLJ21407AAAN	998	106.97	[ATF7IP]
FLJ21414AAAF	797	87.01	[PKP3]
FLJ21415AAAF	205	23.59	[C12orf49]
FLJ21446AAAF	410	45.71	[SIGIRR]
FLJ21458AAAF	347	38.21	[BTNL8]
FLJ21467AAAF	332	36.19	[KDSR]
FLJ21477AAAF	501	57.52	[ATP10B]
FLJ21486AAAF	300	34.87	[GGPS1]
FLJ21491AAAF	222	24.87	[HABP4]
FLJ21509AAAF	859	96.3	[DDX24]
FLJ21511AAAF	699	78.57	[CWH43]
FLJ21512AAAF	207	23.67	[RAB8A]
FLJ21522AAAF	563	63.53	[EPS8L3]
FLJ21528AAAF	245	27.01	[ISX]
FLJ21570AAAF	707	76.21	[C2CD2L]
FLJ21607AAAF	222	25.07	[CHMP3]
FLJ21610AAAF	875	97.1	[GAREM]
FLJ21614AAAF	466	52.25	[BTNL3]
FLJ21620AAAF	239	27.26	[EGLN3]
FLJ21640AAAN	366	39.07	[DISC1]
FLJ21647AAAF	567	60.21	[RANBP3]
FLJ21649AAAN	746	85.78	[SMARCAD1]
FLJ21661AAAF	513	57.06	[GFM2]
FLJ21662AAAF	598	65.95	[POLA2]
FLJ21665AAAF	446	49.91	[TUBAL3]
FLJ21667AAAF	688	77.56	[SCEL]
FLJ21675AAAF	307	34.86	[LRRC59]
FLJ21676AAAF	350	38.32	[RNF167]
FLJ21677AAAN	1856	206.05	[GBF1]
FLJ21683AAAF	963	111.76	[MYO5B]
FLJ21696AAAF	331	36.55	[APOL3]
FLJ21702AAAF	383	42.12	[WDR55]
FLJ21703AAAF	566	63.43	[REPIN1]
FLJ21732AAAF	683	78.54	[THOC5]
FLJ21739AAAF	1067	117.95	[MICAL1]
FLJ21742AAAN	657	71.33	[C19orf44]
FLJ21749AAAF	195	21.59	[TMEM134]

FLJ21760AAAF	438	47.89	[ADD1]
FLJ21764AAAF	414	47.61	[MRPS27]
FLJ21770AAAF	136	15.43	[RPS19BP1]
FLJ21783AAAF	654	72.79	[PHACTR4]
FLJ21786AAAF	372	41.57	[RNF34]
FLJ21787WAAN	635	72.25	[CPSF3]
FLJ21788AAAF	1176	134.48	[LARS]
FLJ21790AAAF	837	96.79	[TFIP11]
FLJ21799AAAF	254	28.91	[FAM192A]
FLJ21802AAAN	641	71.05	[C14orf169]
FLJ21803WAAN	492	55.69	[ALG11]
FLJ21806AAAF	802	92.34	[HSP90B1]
FLJ21807AAAN	370	40.42	[PSAT1]
FLJ21820AAAF	325	37.32	[LDAH]
FLJ21821AAAN	286	31.05	[RAB5C]
FLJ21823AAAF	673	75.17	[KIZ]
FLJ21827AAAF	355	40.1	[IFT46]
FLJ21840AAAF	422	46.67	[REXO4]
FLJ21845AAAF	618	70.8	[ALG9]
FLJ21853AAAN	1124	125.93	[MIA3]
FLJ21861AAAF	456	50.07	[ADSS]
FLJ21864AAAN	641	73.72	[PSME4]
FLJ21871AAAF	387	44.75	[TTC4]
FLJ21878AAAN	629	71.48	[SPECC1L]
FLJ21884AAAF	647	68.42	[SLC39A4]
FLJ21903AAAF	125	14.69	[MAP1LC3B]
FLJ21908AAAF	665	75.7	[RPAP3]
FLJ21916AAAF	234	27.21	[OTUB2]
FLJ21918AAAN	727	78.4	[ESRP2]
FLJ21931AAAF	631	68.1	[SLC3A2]
FLJ21939AAAF	392	44.93	[AZI2]
FLJ21945AAAF	721	79.14	[C2orf44]
FLJ21946AAAF	653	72.97	[MAN1A1]
FLJ21952AAAF	413	47.68	[ZDHHC6]
FLJ21958AAAF	575	67.1	[CCDC93]
FLJ21961AAAF	643	72.53	[ANAPC5]
FLJ21963AAAF	686	74.78	[ACSS3]
FLJ21972AAAF	707	78.73	[DHX33]
FLJ21975AAAN	512	59.62	[KIF20B]
FLJ21988AAAF	476	53	[NARFL]
FLJ21990WAAF	158	18.32	[MRPL50]
FLJ21991AAAF	373	42.14	[PEX3]
FLJ21992AAAF	261	28.59	[SLA2]
FLJ21995AAAF	738	81.5	[TRIM37]
FLJ22018AAAF	420	44.46	[TASP1]
FLJ22021AAAF	925	100.56	[CORO7]
FLJ22026AAAF	646	73.57	[PPWD1]
FLJ22043AAAN	834	90.9	[TNRC6A]

FLJ22062AAAF	137	15.34	[MRPS16]
FLJ22076AAAF	385	43.38	[PELO]
FLJ22077AAAF	121	13.84	[YPEL5]
FLJ22079AAAN	411	46.49	[ASAH1]
FLJ22085AAAF	204	22.97	[ASF1A]
FLJ22088AAAF	570	62.28	[MKKS]
FLJ22089AAAF	309	33.2	[UCP2]
FLJ22107SAAF	145	15.88	[ECHDC1]
FLJ22129AAAF	213	23.13	[ZFAND5]
FLJ22160AAAF	198	21.92	[ARMC7]
FLJ22162AAAF	335	37.72	[NDE1]
FLJ22171AAAF	715	80.62	[EPS8L2]
FLJ22173AAAF	165	16.93	[C3orf36]
FLJ22177AAAF	373	40.43	[MECR]
FLJ22191AAAF	348	40.79	[ZSCAN16]
FLJ22216AAAF	259	28.01	[LRRC61]
FLJ22221AAAF	355	38.95	[FKBP10]
FLJ22222AAAF	319	35.72	[OGFOD3]
FLJ22223AAAF	352	39.52	[SIRT2]
FLJ22228AAAF	469	51.42	[KRT7]
FLJ22237AAAF	347	39.12	[HDAC11]
FLJ22243AAAN	421	47.81	[MAU2]
FLJ22252AAAF	414	44.12	[SOX17]
FLJ22261AAAN	919	104.44	[RNF213]
FLJ22269AAAF	441	45.72	[MFSD7]
FLJ22273AAAF	223	25.27	[AK4]
FLJ22274AAAF	339	36.98	[SARAF]
FLJ22277AAAF	257	29.4	[REEP4]
FLJ22282AAAF	257	29.4	[FAM57A]
FLJ22317AAAF	329	36.56	[BABAM1]
FLJ22319AAAF	1010	112.35	[PPFIA3]
FLJ22320AAAF	338	38.16	[HEMK1]
FLJ22321AAAF	360	40.24	[CCDC86]
FLJ22326AAAF	490	54.12	[FLAD1]
FLJ22328AAAF	299	33.26	[TMEM38A]
FLJ22337AAAF	701	78.58	[ACSS2]
FLJ22345AAAF	397	46.16	[TOR3A]
FLJ22347AAAF	874	93.79	[TUT1]
FLJ22349AAAF	229	26.56	[CCDC134]
FLJ22351AAAF	328	36.41	[ATPAF1]
FLJ22353AAAF	277	31.6	[TMEM53]
FLJ22356AAAF	105	11.36	[RAB42]
FLJ22357AAAF	855	97.33	[RHBDF1]
FLJ22361AAAF	392	45.79	[CERS5]
FLJ22362AAAF	234	25.16	[FNDC4]
FLJ22371AAAF	395	44.65	[XKR8]
FLJ22377AAAF	154	17.34	[FAM162A]
FLJ22386AAAN	287	32.25	[ROGDI]

FLJ22393AAAF	273	30.93	[SPSB1]
FLJ22402AAAF	390	43.69	[NEIL1]
FLJ22408AAAF	360	40.91	[EPHX3]
FLJ22415AAAF	373	41.28	[CLMP]
FLJ22418AAAF	282	30.89	[VTCN1]
FLJ22419AAAF	395	42.3	[ZNF385D]
FLJ22422AAAF	350	38.02	[CCNO]
FLJ22436AAAF	606	67.69	[NDOR1]
FLJ22449AAAF	580	65.02	[MCOLN1]
FLJ22451AAAF	166	18.03	[MTFP1]
FLJ22457AAAF	468	53.12	[DENND2D]
FLJ22465AAAF	306	33.85	[RTFDC1]
FLJ22470AAAF	467	52.69	[ZBTB43]
FLJ22471AAAF	331	36.96	[CCDC92]
FLJ22477AAAF	158	16.79	[C16orf95]
FLJ22484AAAF	291	32.97	[MFF]
FLJ22509AAAN	327	34.51	[PRRC2B]
FLJ22511AAAF	151	16.94	[MYL6]
FLJ22514AAAF	445	49.83	[TUBB4B]
FLJ22521AAAF	418	46.68	[SERPINA1]
FLJ22530AAAN	406	45.36	[CHD1L]
FLJ22537AAAF	177	20.2	[COPZ1]
FLJ22551AAAF	274	30.67	[ASB7]
FLJ22555AAAF	291	32.49	[C2orf47]
FLJ22559AAAF	368	42.11	[OBFC1]
FLJ22570AAAF	496	53.25	[DOK3]
FLJ22573AAAF	355	37.89	[IGFLR1]
FLJ22575AAAF	331	37.58	[PHF11]
FLJ22578AAAN	155	16.77	[MRM1]
FLJ22580AAAF	277	31.54	[CCNB1IP1]
FLJ22587AAAF	106	12.35	[C8orf4]
FLJ22589AAAF	475	52.93	[TUBE1]
FLJ22595AAAF	192	21.59	[ARL14]
FLJ22603AAAF	256	29.62	[GDPD3]
FLJ22609AAAF	340	38.24	[NSUN3]
FLJ22613WAAF	154	17.03	[FER1L4]
FLJ22626AAAF	415	47.11	[NKAP]
FLJ22634AAAF	252	27.84	[GRHPR]
FLJ22637AAAF	397	45.36	[ELL3]
FLJ22638AAAF	154	17.57	[RPP21]
FLJ22644AAAF	284	32.28	[MOGAT2]
FLJ22645AAAF	83	9.62	[C15orf48]
FLJ22649AAAF	180	20.31	[SPCS3]
FLJ22655AAAF	205	23.85	[RERGL]
FLJ22657AAAF	134	15.33	[CYB5A]
FLJ22659WAAF	167	18.12	[LOC79999]
FLJ22662AAAF	506	58.29	[PLBD1]
FLJ22666AAAF	319	37.07	[FBXO8]

FLJ22696AAAF	117	13.62	[TCEAL8]
FLJ22700AAAF	170	18.89	[ZNF576]
FLJ22707AAAF	140	15.85	[NXT1]
FLJ22709AAAF	264	29.41	[OCEL1]
FLJ22714AAAF	182	19.98	[PRORY]
FLJ22724AAAF	183	20.55	[SPAG16]
FLJ22737AAAF	341	37.86	[ARFIP2]
FLJ22738AAAF	607	68.68	[GNL1]
FLJ22741WAAN	688	76.04	[TRAPPC9]
FLJ22745AAAF	409	46.07	[NLRP1]
FLJ22759AAAF	703	78.81	[DHX35]
FLJ22779AAAF	505	57.28	[HCK]
FLJ22784AAAN	633	72.32	[PPP4R3B]
FLJ22788AAAF	757	85.32	[RASA4]
FLJ22798AAAF	490	55.02	[UBXN7]
FLJ22802AAAF	187	20.96	[VIMP]
FLJ22833AAAF	134	14.68	[NABP1]
FLJ22852AAAF	63	7.25	[COX7C]
FLJ22855AAAN	699	78.41	[FGFR2]
FLJ22856AAAF	346	39.07	[CDK7]
FLJ22861AAAF	125	13.82	[PAM16]
FLJ22869AAAF	387	43.6	[TSSC1]
FLJ22871AAAF	204	22.92	[POLR3H]
FLJ22872AAAF	367	39.43	[GAPDH]
FLJ22873AAAF	165	17.82	[RPL12]
FLJ22889AAAF	110	12.54	[RPL35A]
FLJ22896AAAF	373	41.9	[NSDHL]
FLJ22901AAAF	337	38.61	[DCPS]
FLJ22904AAAF	126	13.8	[HINT1]
FLJ22905AAAF	188	21.63	[RPL18]
FLJ22909AAAF	90	9.39	[HMGN2]
FLJ22911AAAF	394	45.66	[GNAS]
FLJ22916AAAF	165	18.01	[PPIA]
FLJ22923AAAF	492	53.83	[TOM1]
FLJ22926AAAF	114	11.51	[RPLP1]
FLJ22927AAAF	239	27.36	[PSME2]
FLJ22928AAAF	358	38.87	[SLC9A3R1]
FLJ22932AAAN	314	34.9	[EPCAM]
FLJ22935AAAF	398	44.4	[POLR3D]
FLJ22936AAAF	427	48.86	[SEPT6]
FLJ22940AAAF	132	15.24	[SNRNP25]
FLJ22955AAAF	132	14.93	[DCAKD]
FLJ22958AAAF	550	64.69	[SOAT1]
FLJ22960AAAF	134	15.58	[GOLGA7]
FLJ22964AAAN	301	34	[TCEA1]
FLJ22965AAAF	222	25.62	[CXorf56]
FLJ22966AAAF	477	53.2	[YARS2]
FLJ22969AAAN	836	92.88	[CDCP1]

FLJ22970AAAF	150	16.76	[COX5A]
FLJ22973AAAF	419	46.51	[IKZF5]
FLJ22975AAAN	108	11.87	[LEMD1]
FLJ22980AAAF	325	35.36	[CYC1]
FLJ22982AAAF	386	45.13	[CHST4]
FLJ22983AAAF	166	18.24	[PPIL1]
FLJ22985AAAN	547	61.3	[COG6]
FLJ22986AAAF	133	14.39	[FAU]
FLJ22988AAAF	501	54.86	[ALDH1A1]
FLJ22989AAAF	161	17.49	[DBNDD2]
FLJ22992AAAF	708	79.9	[POLR3E]
FLJ22996AAAF	56	6.68	[RPS29]
FLJ23012AAAF	299	32.58	[F11R]
FLJ23030AAAF	413	48.62	[SGMS1]
FLJ23087AAAF	393	44.28	[SDR42E1]
FLJ23101AAAF	594	68.33	[ZBED8]
FLJ23109AAAF	491	54.5	[CBLL1]
FLJ23121AAAF	612	70.09	[ADGB]
FLJ23127AAAN	481	53.56	[WDR19]
FLJ23147AAAF	232	26.7	[CRLF2]
FLJ23155AAAF	110	12.03	[HERC4]
FLJ23157AAAN	302	34.73	[TAF7L]
FLJ23164AAAF	619	70.8	[SPEF2]
FLJ23168AAAF	333	37.47	[CCDC33]
FLJ23182AAAN	396	45.35	[TFB2M]
FLJ23204AAAF	175	20.05	[MRPL17]
FLJ23209AAAF	517	55.68	[PDZD7]
FLJ23212AAAF	221	25.43	[VPS28]
FLJ23221AAAF	131	14.98	[C1orf54]
FLJ23231AAAF	599	65.76	[ZC3H12A]
FLJ23233AAAF	478	54.98	[ZNF419]
FLJ23235AAAF	296	34.32	[TMEM156]
FLJ23236AAAN	125	14.28	[PDS5B]
FLJ23244AAAF	531	59.73	[RIC8A]
FLJ23251AAAF	404	44.86	[UBA5]
FLJ23256AAAF	479	54.56	[UTP3]
FLJ23259AAAF	490	54.65	[LRRC31]
FLJ23263AAAF	508	56.9	[TTI2]
FLJ23280AAAF	431	49.25	[EIF5]
FLJ23281AAAN	627	69.32	[STRN]
FLJ23288AAAN	577	65.95	[ZNF211]
FLJ23290AAAN	425	47.07	[SDPR]
FLJ23292AAAF	358	39.56	[TRIB3]
FLJ23293AAAF	579	66.16	[ATL2]
FLJ23305AAAF	715	82.19	[CCDC170]
FLJ23307AAAF	319	35.12	[SPRY1]
FLJ23312AAAF	145	15.57	[C5orf66]
FLJ23317AAAF	272	31.05	[STX16]

FLJ23318AAAF	473	53.11	[UBXN4]
FLJ23329AAAF	204	21.98	[NRSN2]
FLJ23330AAAF	418	47	[AP3M1]
FLJ23337AAAF	491	55.02	[NPRL3]
FLJ23342AAAF	507	55.4	[MSANTD2]
FLJ23353AAAN	540	59.39	[TOX3]
FLJ23356AAAF	350	40.05	[POMK]
FLJ23362AAAF	759	82.68	[MTSS1]
FLJ23363AAAF	278	32.06	[TAF1D]
FLJ23371AAAF	348	38.97	[TCEA3]
FLJ23378AAAF	265	31.3	[ELOVL6]
FLJ23384AAAF	352	37.7	[AGMAT]
FLJ23392AAAF	574	61.83	[ZBTB3]
FLJ23398AAAF	292	34.08	[DCUN1D4]
FLJ23414AAAF	350	39.95	[SUV39H2]
FLJ23427AAAF	435	46.71	[LTBR]
FLJ23428AAAF	403	42.91	[ZNHIT2]
FLJ23436AAAF	520	57.93	[ZNF768]
FLJ23442AAAF	118	14.38	[FAM103A1]
FLJ23445AAAN	204	23.67	[THOC7]
FLJ23451AAAF	323	36.13	[CAMKMT]
FLJ23458AAAF	76	8.67	[C18orf32]
FLJ23461AAAF	110	12.04	[ZNF655]
FLJ23467AAAF	184	20.56	[TNFAIP8L2]
FLJ23468AAAN	176	20.71	[CENPU]
FLJ23469AAAF	221	24.1	[ISOC2]
FLJ23485AAAF	697	80.2	[FAM13A]
FLJ23499AAAF	150	17.78	[C11orf1]
FLJ23506AAAF	534	61	[ZNF671]
FLJ23592AAAF	658	76.77	[TSNAXIP1]
FLJ23611AAAF	390	41.01	[ZNF414]
FLJ23614AAAF	164	19.23	[ZNF449]
FLJ23617AAAF	245	28.05	[CMBL]
FLJ23624AAAF	381	44.32	[ADAP2]
FLJ23627AAAF	501	54.41	[SLC37A2]
FLJ23636AAAN	209	23.91	[GPX8]
FLJ23637AAAN	506	56.95	[UTP15]
FLJ23660AAAF	789	89.26	[PLCD3]
FLJ23662AAAN	367	41.06	[TRIM44]
FLJ23666AAAF	552	62.04	[SLC22A9]
FLJ23671AAAF	917	105.76	[UGGT1]
FLJ23678AAAF	315	35.82	[RHBDD1]
FLJ23683AAAF	312	33.5	[CIAPIN1]
FLJ23700AAAF	757	85.98	[PDE8A]
FLJ23713AAAF	225	25.43	[GRPEL2]
FLJ23716AAAF	546	61.67	[DCAF11]
FLJ23722AAAF	847	97.37	[FASTKD1]
FLJ23725AAAF	893	100.68	[TBCK]

FLJ23733AAAF	776	86.45	[QRICH1]
FLJ23737AAAF	763	83.93	[SOX5]
FLJ23743AAAF	469	51.75	[NSUN6]
FLJ23747AAAN	785	90.8	[U2SURP]
FLJ23751AAAF	480	55.24	[PXYLP1]
FLJ23753AAAN	496	57.52	[SUZ12]
FLJ23757AAAF	599	66.61	[DQX1]
FLJ23768AAAF	523	56.58	[DYNC1LI1]
FLJ23802AAAF	385	42.29	[GFOD2]
FLJ23810AAAF	216	25.24	[KCNIP1]
FLJ23811AAAF	445	49.91	[MYLIP]
FLJ23818AAAF	148	15.38	[PRR13]
FLJ23825AAAN	478	52.95	[HEXDC]
FLJ23834AAAF	277	31.26	[CDHR3]
FLJ23847AAAF	377	43.63	[ZDHHC16]
FLJ23851AAAF	172	18.87	[NENF]
FLJ23853AAAF	191	21.97	[PIFO]
FLJ23875AAAN	467	51.64	[C11orf16]
FLJ23897AAAF	165	17.67	[CDH26]
FLJ23899AAAN	552	63.8	[C12orf4]
FLJ23907AAAF	495	53.99	[SCARA5]
FLJ23915AAAF	628	66.29	[LRFN3]
FLJ23917AAAN	551	58.96	[GLDN]
FLJ23922AAAF	617	70.67	[SLFN12L]
FLJ23923AAAN	780	88.31	[PPP1R21]
FLJ23927AAAF	437	48.2	[TMPRSS4]
FLJ23928AAAF	291	32.76	[C1QTNF1]
FLJ23936AAAF	384	44.89	[CERS6]
FLJ23941AAAF	354	40.73	[C6orf89]
FLJ23942AAAF	756	84.48	[LOXL4]
FLJ23946AAAF	568	63.06	[SLC13A5]
FLJ23947AAAF	340	37.5	[GPR35]
FLJ23963AAAF	951	107.07	[ATP9A]
FLJ23971AAAN	492	54.06	[SLC39A14]
FLJ23980AAAF	458	49.17	[BPIFB2]
FLJ23984AAAF	537	57.56	[SLC9B2]
FLJ23987AAAF	257	28.8	[TMEM101]
FLJ23993AAAN	512	54.81	[SPNS3]
FLJ23996AAAF	328	35.65	[SIGLEC15]
FLJ23998AAAF	340	37.75	[SLC51A]
FLJ23999AAAF	218	24.28	[TMIGD1]
FLJ24003AAAF	453	49.33	[TMPRSS3]
FLJ24005AAAF	264	28.69	[CYB5D2]
FLJ24006AAAF	576	64.15	[RSPRY1]
FLJ24009AAAF	563	62.95	[NCLN]
FLJ24010AAAF	308	34.9	[SPG21]
FLJ24011AAAF	413	45.78	[TMEM184A]
FLJ24092AAAF	304	32.9	[KAZALD1]

FLJ25006AAAF	274	31.04	[SGK494]
FLJ25008AAAF	612	70.53	[ZNF354B]
FLJ25009AAAF	314	35.42	[SPP1]
FLJ25022AAAF	351	38.59	[ATF4]
FLJ25033AAAF	180	19.78	[TDH]
FLJ25038AAAF	126	13.19	[CARKD]
FLJ25048AAAF	246	27.91	[COQ5]
FLJ25059AAAF	166	18.5	[IMMP1L]
FLJ25062AAAF	240	27.84	[METTL6]
FLJ25063AAAF	245	25.73	[C1QC]
FLJ25066AAAN	223	25.51	[DOK6]
FLJ25068AAAF	440	50.9	[SEPT8]
FLJ25070AAAF	517	58.58	[RNPC3]
FLJ25077AAAF	222	24.76	[C9orf72]
FLJ25078AAAF	269	29.56	[C1orf74]
FLJ25082AAAF	326	37.61	[SAMD8]
FLJ25087AAAF	252	28.48	[ZFYVE21]
FLJ25091WAAF	160	18.7	[PTGES3]
FLJ25102AAAF	159	17.37	[C2orf48]
FLJ25104AAAF	375	41.69	[AS3MT]
FLJ25107AAAF	355	38.56	[HNRNPD]
FLJ25113AAAF	451	50.05	[TUBA1A]
FLJ25115AAAF	476	53.02	[RAB3IP]
FLJ25116AAAF	205	23.76	[EFNA1]
FLJ25125AAAF	134	14.29	[SNCB]
FLJ25137AAAF	360	41.79	[LETMD1]
FLJ25143AAAF	162	17.84	[C2orf50]
FLJ25146AAAF	181	20.7	[ARF1]
FLJ25152AAAF	261	28.09	[WBP2]
FLJ25154AAAF	435	49.63	[AP2M1]
FLJ25157AAAF	201	22.26	[UBE2E2]
FLJ25160AAAF	112	12.47	[TCEB1]
FLJ25161AAAF	140	15.68	[FAM19A4]
FLJ25163AAAF	239	25.81	[LURAP1]
FLJ25168AAAF	157	16.89	[CRHR1-IT1]
FLJ25169AAAF	135	14.32	[NKX6-3]
FLJ25173AAAF	366	39.98	[IST1]
FLJ25174AAAF	143	15.68	[SLC31A2]
FLJ25187AAAF	242	27.21	[IGSF1]
FLJ25199AAAF	205	22.88	[HEBP2]
FLJ25202AAAF	560	63.66	[RSPH3]
FLJ25205AAAF	287	32.58	[FBXO17]
FLJ25219AAAF	123	13.98	[C11orf52]
FLJ25248AAAF	213	24.05	[GTF3C6]
FLJ25250AAAN	482	52.59	[IGHA2]
FLJ25267AAAF	318	36.73	[UBLCP1]
FLJ25284AAAF	266	28.98	[HPGD]
FLJ25287AAAF	372	42.7	[FA2H]

FLJ25290AAAF	375	41.74	[ACTB]
FLJ25303AAAF	1035	114.13	[ATP4A]
FLJ25315AAAF	422	49.44	[PPP1R36]
FLJ25317AAAN	220	23.8	[SH3RF2]
FLJ25321AAAF	241	26.49	[PSMB1]
FLJ25323AAAF	360	39.72	[C12orf42]
FLJ25326AAAF	226	26.07	[ATP6V1E2]
FLJ25331AAAF	148	16.87	[GTSF1L]
FLJ25333AAAF	414	47.73	[FAM81B]
FLJ25339AAAF	395	43.8	[PRSS54]
FLJ25344AAAF	193	21.04	[ZNF534]
FLJ25349AAAF	215	23.88	[TCP10L]
FLJ25355AAAF	384	45	[CALR3]
FLJ25358AAAF	262	30.17	[C9orf24]
FLJ25359AAAF	506	58.51	[BTBD16]
FLJ25360AAAF	149	16.55	[C20orf173]
FLJ25362AAAF	328	37.89	[CCDC54]
FLJ25368AAAF	335	38.87	[CCDC68]
FLJ25369AAAF	180	20.61	[TEX37]
FLJ25370AAAF	283	31.3	[BOLL]
FLJ25379AAAF	226	24.43	[PSMD10]
FLJ25384AAAF	226	25.96	[DNAJC30]
FLJ25385AAAF	375	42.62	[ADIPOR1]
FLJ25390AAAF	330	37.43	[ARMC12]
FLJ25396AAAF	288	31.69	[RHOXF2]
FLJ25402AAAF	319	36.87	[C8orf48]
FLJ25404AAAF	132	14.38	[C16orf92]
FLJ25406AAAF	440	50.34	[ZMYND10]
FLJ25409AAAF	396	44.91	[LETM2]
FLJ25410AAAF	358	40.78	[SEPT12]
FLJ25414AAAF	384	42.29	[SPATA32]
FLJ25429AAAF	280	30.81	[UBXN10]
FLJ25430AAAF	313	33.91	[DHRS1]
FLJ25431AAAN	251	27.77	[MRPS5]
FLJ25436AAAF	354	38.14	[L3HYPDH]
FLJ25439AAAF	361	40.82	[TTC23L]
FLJ25442AAAF	300	33.71	[SPATS1]
FLJ25444AAAF	332	38.85	[CXorf58]
FLJ25445AAAF	299	35.02	[CAPZA3]
FLJ25449AAAF	368	42.61	[PP2D1]
FLJ25452AAAF	332	36.31	[LDHC]
FLJ25459AAAF	475	53.79	[CNOT6L]
FLJ25462AAAF	442	50.55	[CRBN]
FLJ25463AAAF	381	41.98	[LDHAL6B]
FLJ25464AAAF	289	32.66	[PPA1]
FLJ25470AAAF	508	58.14	[VRK2]
FLJ25472AAAF	283	30.66	[VDAC3]
FLJ25473AAAF	239	26.02	[LSM14B]

FLJ25493AAAF	733	81.75	[TRMT1L]
FLJ25501AAAF	284	33.31	[NIPSNAP1]
FLJ25518AAAF	421	47.58	[CNP]
FLJ25523AAAF	415	47.12	[B4GAT1]
FLJ25528AAAF	920	104.84	[USP11]
FLJ25530AAAF	416	45.94	[HEPACAM]
FLJ25531AAAF	326	37.59	[MEST]
FLJ25534AAAF	282	31.89	[LIX1]
FLJ25535AAAF	295	33.2	[NAPA]
FLJ25547AAAF	195	21.48	[GVQW1]
FLJ25557AAAF	485	55.5	[PAF1]
FLJ25568AAAF	358	40.19	[HLA-E]
FLJ25574AAAF	446	49.86	[TUBB6]
FLJ25586AAAF	480	52.15	[PKMYT1]
FLJ25590AAAF	131	13.44	[LINC01465]
FLJ25608AAAF	261	27.72	[CLDN18]
FLJ25620AAAF	244	27.08	[YIPF4]
FLJ25628AAAF	200	22.5	[TCEAL3]
FLJ25636AAAF	391	44.59	[CNTLN]
FLJ25658AAAF	376	42.19	[RNF133]
FLJ25660AAAF	369	42.39	[FAM187B]
FLJ25666AAAF	112	12.94	[LOC286238]
FLJ25671AAAN	245	27.97	[MMACHC]
FLJ25674AAAF	282	31.46	[ESD]
FLJ25675AAAF	339	38.75	[OSCP1]
FLJ25676AAAF	362	40.26	[ZFYVE1]
FLJ25680AAAF	328	36.05	[POU5F2]
FLJ25681AAAF	364	40.78	[PRPF31]
FLJ25683AAAF	543	59.37	[CCT7]
FLJ25690AAAF	174	19.91	[UBL4B]
FLJ25691AAAF	367	41.52	[APOBEC4]
FLJ25697AAAF	240	24.61	[SNRPN]
FLJ25701AAAF	260	30.83	[CCDC127]
FLJ25708AAAF	548	56.56	[ANKHD1]
FLJ25711AAAF	290	33.22	[C21orf59]
FLJ25720AAAF	382	45.43	[RIBC2]
FLJ25722AAAN	318	35.55	[APEX1]
FLJ25725AAAF	361	43.5	[SPATA17]
FLJ25732AAAF	248	27.47	[CMTM2]
FLJ25735AAAF	425	48.78	[ZNF645]
FLJ25736AAAF	255	29.31	[FMR1NB]
FLJ25740AAAF	295	34.28	[ASB17]
FLJ25742AAAF	459	53.41	[XKR3]
FLJ25747AAAF	359	40.26	[LGALS8]
FLJ25748AAAF	526	60.09	[ALG8]
FLJ25750AAAF	489	54.35	[PMPCB]
FLJ25769AAAF	451	50.15	[TUBA1B]
FLJ25773AAAF	125	13.89	[C14orf177]

FLJ25776AAAF	230	26.11	[ROPN1L]
FLJ25782AAAF	491	53.86	[FDXR]
FLJ25787AAAF	212	23.81	[CDKN3]
FLJ25788AAAF	305	34.37	[RNF148]
FLJ25797AAAF	183	20.68	[RHEBL1]
FLJ25800AAAF	229	26.58	[TMEM217]
FLJ25801AAAF	387	43.98	[TRIML2]
FLJ25805AAAF	285	29.64	[DDAH2]
FLJ25811AAAF	313	35.07	[LRRC52]
FLJ25818AAAF	438	50.15	[EFCAB3]
FLJ25821AAAF	199	22.43	[RNASE11]
FLJ25823AAAF	189	21.24	[CT45A4]
FLJ25823AAAN	189	21.24	[CT45A1]
FLJ25832AAAF	333	38.17	[EID3]
FLJ25834AAAF	495	55.76	[ZSCAN5A]
FLJ25837AAAF	307	35.29	[HORMAD2]
FLJ25851AAAF	167	19.19	[SPATA19]
FLJ25853AAAF	233	28.65	[CCDC70]
FLJ25881AAAF	470	53.37	[SYT17]
FLJ25896AAAF	326	35.86	[MLST8]
FLJ25901AAAF	206	23.34	[SNAP25]
FLJ25903AAAF	131	14.93	[MTURN]
FLJ25908AAAF	525	53.35	[FUS]
FLJ25912AAAF	364	39.42	[ALDOA]
FLJ25918AAAF	299	33.34	[NMRAL1]
FLJ25929AAAF	204	24.15	[RPL15]
FLJ25932AAAF	180	20.33	[OPA3]
FLJ25956AAAN	326	36.74	[PRKAR1B]
FLJ25963AAAN	173	18.8	[MYDGF]
FLJ25968AAAF	82	9.04	[CTXN1]
FLJ25971AAAF	717	80.99	[RSPH6A]
FLJ25973AAAF	424	48.52	[SLC35A5]
FLJ25976AAAF	122	13.38	[C14orf178]
FLJ25977AAAF	249	26.67	[TPI1]
FLJ25987AAAN	229	25.76	[UBB]
FLJ25989AAAF	337	38.59	[DGAT2L6]
FLJ25994AAAF	404	46.82	[SNX5]
FLJ25998AAAF	273	31.09	[GJB5]
FLJ26002AAAF	92	9.87	[VKORC1]
FLJ26010AAAF	501	57.14	[DARS]
FLJ26016AAAF	134	14.59	[FUOM]
FLJ26058AAAF	437	50.12	[EEF1G]
FLJ26062AAAF	184	20.78	[GLO1]
FLJ26076AAAF	409	46.05	[KRT18]
FLJ26087AAAF	151	16.32	[ARPC5]
FLJ26090AAAF	128	14.37	[SLC51B]
FLJ26099AAAF	114	12.77	[SH3BGRL]
FLJ26102AAAF	190	21.09	[SLC31A1]

FLJ26104AAAF	131	13.51	[LY6E]
FLJ26122AAAF	146	16.05	[SDIM1]
FLJ26140AAAF	127	14.65	[MRFAP1]
FLJ26144AAAF	276	31.08	[GNPDA2]
FLJ26145AAAF	309	31.86	[WBP2NL]
FLJ26174AAAF	131	13.47	[LOC100131831]
FLJ26178AAAF	470	52.9	[SNX17]
FLJ26184AAAF	133	14.6	[C16orf47]
FLJ26237AAAF	149	17.3	[STMN1]
FLJ26243AAAF	317	34.27	[RPLP0]
FLJ26256AAAF	182	20.51	[VPS29]
FLJ26259AAAF	198	21.68	[SRI]
FLJ26267AAAF	227	24.64	[PCMT1]
FLJ26280AAAF	331	38.89	[RCN1]
FLJ26281AAAF	488	54.62	[MMP11]
FLJ26285AAAF	362	40.48	[HLA-B]
FLJ26303AAAF	320	34.2	[HNRNPA1]
FLJ26307AAAF	282	31.6	[AGPAT1]
FLJ26312AAAF	185	20.48	[DUSP3]
FLJ26314AAAF	154	16.88	[ZMYM6NB]
FLJ26315AAAF	264	29.94	[RPS3A]
FLJ26317AAAF	486	53.03	[KRT28]
FLJ26324AAAF	354	38.69	[MYBPHL]
FLJ26326AAAF	140	14.87	[RPL23]
FLJ26335AAAF	298	33.92	[OGN]
FLJ26344AAAF	334	37.26	[OSTM1]
FLJ26350AAAF	539	57.92	[CCT4]
FLJ26352AAAF	229	25.81	[TMEM182]
FLJ26354AAAF	155	17.62	[ANK1]
FLJ26361AAAF	156	17.64	[RNASE1]
FLJ26362AAAF	541	62.69	[USP39]
FLJ26365AAAF	233	26.8	[LAPTM4A]
FLJ26368AAAF	381	43.1	[CKM]
FLJ26371AAAF	334	36.64	[LDHB]
FLJ26374AAAF	558	63.18	[GPI]
FLJ26377AAAF	390	43.3	[PDHA1]
FLJ26378AAAF	214	24.98	[KDELR3]
FLJ26382AAAF	377	42.02	[ACTC1]
FLJ26383AAAF	380	43.64	[MASP1]
FLJ26388AAAF	109	12.35	[PPP1R1C]
FLJ26390AAAF	553	59.75	[ATP5A1]
FLJ26397AAAF	297	32.88	[ATP5C1]
FLJ26401AAAF	614	69.74	[NCOA4]
FLJ26406AAAF	352	39.75	[CXCR4]
FLJ26418AAAF	295	32.85	[RPSA]
FLJ26424AAAF	640	70.7	[PCK2]
FLJ26440AAAF	263	30.34	[IYD]
FLJ26443AAAF	121	13.42	[ATP11AUN]

FLJ26464AAAF	211	23.74	[ILDR1]
FLJ26468AAAF	680	77.05	[POR]
FLJ26473AAAF	476	52.76	[WDSUB1]
FLJ26475AAAF	487	54.02	[SLC16A4]
FLJ26476AAAF	166	19.2	[MRPL49]
FLJ26489AAAF	320	35.2	[BSND]
FLJ26523AAAF	298	32.54	[KHK]
FLJ26536AAAF	327	35.22	[PNRC1]
FLJ26538AAAF	266	30.34	[ITM2B]
FLJ26540AAAF	237	26.54	[TMBIM6]
FLJ26550AAAF	337	37.54	[TALDO1]
FLJ26556AAAF	193	21.77	[RHOA]
FLJ26563AAAF	202	21.63	[TM4SF1]
FLJ26565AAAF	199	22.7	[AP1S2]
FLJ26568AAAF	396	45.83	[MRPS9]
FLJ26569AAAN	294	33.14	[RARRES1]
FLJ26572AAAF	406	46.15	[EIF4A1]
FLJ26577AAAF	407	46.43	[EIF4A2]
FLJ26578AAAF	311	33.79	[TMEM177]
FLJ26584AAAF	345	37.19	[GHITM]
FLJ26595AAAF	332	36.69	[LDHA]
FLJ26596AAAF	126	13.92	[HIST1H2BN]
FLJ26620AAAF	348	38.47	[CAPG]
FLJ26621AAAF	201	22.19	[SDC2]
FLJ26624AAAF	329	36.97	[CTSK]
FLJ26630AAAF	450	49.89	[SQRDL]
FLJ26639AAAF	148	16.54	[LYZ]
FLJ26643AAAF	365	41.06	[HLA-A]
FLJ26651AAAF	179	20.19	[M1AP]
FLJ26653AAAF	246	27	[MARCH2]
FLJ26657AAAF	468	50.87	[COQ6]
FLJ26661AAAF	291	31.95	[PQLC2]
FLJ26668AAAF	412	44.55	[CTSD]
FLJ26669AAAF	572	65.13	[LMNA]
FLJ26674AAAF	339	37.82	[CTSB]
FLJ26678AAAF	529	59.45	[FGR]
FLJ26682AAAF	462	50.77	[RARA]
FLJ26689AAAF	286	30.88	[OSCAR]
FLJ26726AAAF	467	51.83	[PNLIPRP1]
FLJ26801AAAF	621	68.76	[ACAD9]
FLJ26847AAAF	200	22.97	[CDO1]
FLJ26849AAAF	539	58.22	[PCCB]
FLJ26862AAAF	324	36.75	[FAM49B]
FLJ26865AAAF	556	63.12	[HEXB]
FLJ26902AAAF	398	45.47	[IL1R2]
FLJ26975AAAF	79	8.19	[SMR3B]
FLJ26992AAAF	223	25.13	[SIGMAR1]
FLJ26993AAAF	51	6.15	[HTN3]

FLJ26996AAAF	268	29.16	[APOOL]
FLJ27013AAAF	636	70.67	[PABPC1]
FLJ27017AAAF	385	42.25	[GDPGP1]
FLJ27023AAAF	764	85.45	[CFB]
FLJ27032AAAF	290	30.95	[VGLL4]
FLJ27039AAAF	591	66.69	[CTPS1]
FLJ27047AAAF	354	37.03	[MUC7]
FLJ27054AAAN	235	24.34	[DHX9]
FLJ27066AAAF	239	28.22	[ARHGAP24]
FLJ27072AAAF	214	24.58	[RPL10]
FLJ27084AAAF	405	44.43	[ABTB1]
FLJ27095AAAF	203	23.58	[RPL13A]
FLJ27096AAAF	393	43.14	[SERBP1]
FLJ27106AAAF	470	52.03	[ATP6AP1]
FLJ27118AAAF	301	33.43	[PPIE]
FLJ27123AAAF	325	36.48	[EIF3I]
FLJ27154AAAF	350	37.87	[MTHFD2]
FLJ27173AAAF	923	104.13	[MSH6]
FLJ27177AAAF	469	54.01	[MMP1]
FLJ27223AAAF	613	69.34	[OS9]
FLJ27228AAAF	307	33.88	[VTA1]
FLJ27262AAAF	136	15.33	[H3F3B]
FLJ27267AAAF	217	22.25	[LINC00176]
FLJ27271AAAF	362	39.47	[HSPBP1]
FLJ27274AAAF	427	47.63	[RPL4]
FLJ27277AAAF	390	43.82	[TSG101]
FLJ27278AAAF	161	17.11	[LITAF]
FLJ27302AAAF	373	40.35	[RTN4]
FLJ27310AAAF	172	18.16	[TMEM91]
FLJ27328AAAF	254	27.8	[HLA-DQA1]
FLJ27337AAAF	172	19.6	[TPT1]
FLJ27340AAAF	104	12.26	[ERH]
FLJ27345AAAF	136	14.55	[PCP2]
FLJ27349AAAF	186	22.49	[HMGB4]
FLJ27352AAAF	121	13.76	[C15orf65]
FLJ27353AAAF	214	24.52	[RPL10L]
FLJ27355AAAF	191	21.86	[LRRC69]
FLJ27356AAAF	195	21.48	[SPACA7]
FLJ27357AAAF	168	19.81	[TMEM31]
FLJ27369AAAF	172	19.83	[MYL9]
FLJ27379AAAF	143	15.63	[MDK]
FLJ27441AAAF	134	14.41	[SYCN]
FLJ27460AAAF	156	17.66	[SCP2D1]
FLJ27485AAAF	199	22.41	[C1orf185]
FLJ27505AAAF	134	15.45	[TEX43]
FLJ27508AAAF	205	23.7	[IQCF1]
FLJ27514AAAF	192	21.4	[IZUMO2]
FLJ29001AAAF	348	38.95	[SYT15]

FLJ29002AAAF	235	26.44	[PRSS37]
FLJ29008AAAF	338	38.47	[TSSK4]
FLJ29009AAAF	450	49.87	[TUBA3E]
FLJ29012AAAF	342	39.1	[APTX]
FLJ29013AAAF	435	46.82	[AAMP]
FLJ29015AAAF	199	22.11	[PRDX1]
FLJ29017AAAF	169	18.61	[SDHC]
FLJ29019AAAF	175	20.02	[FTL]
FLJ29027AAAF	279	31.53	[ATP1B3]
FLJ29030AAAF	374	42.5	[EIF3M]
FLJ29033AAAF	99	11.1	[CXCL8]
FLJ29036AAAF	714	78.22	[SLC12A8]
FLJ30001AAAF	309	34.88	[UBAC2]
FLJ30002AAAF	234	25.25	[PAQR4]
FLJ30004AAAF	205	23.52	[STARD4]
FLJ30009AAAF	191	21.51	[NKIRAS2]
FLJ30012AAAF	455	50.63	[DDX47]
FLJ30014AAAF	257	27.99	[YIPF5]
FLJ30020AAAF	578	65.96	[ZUFSP]
FLJ30023AAAF	393	44.83	[DSCC1]
FLJ30031AAAF	270	30.39	[DTNBP1]
FLJ30041AAAF	561	65.1	[DET1]
FLJ30044AAAF	498	55.26	[ZNF79]
FLJ30046AAAF	305	33.52	[SLAIN1]
FLJ30055AAAF	426	46.71	[AMDHD1]
FLJ30067AAAF	852	98.3	[WDR63]
FLJ30079AAAN	402	45.83	[SNUPN]
FLJ30102AAAN	559	63.8	[NXPE3]
FLJ30105AAAF	563	60.44	[IMPDH1]
FLJ30107AAAF	459	49.77	[SLC46A1]
FLJ30111AAAF	397	41.9	[ACAA2]
FLJ30114AAAF	527	59.59	[ZKSCAN1]
FLJ30119AAAF	239	27.21	[GUCD1]
FLJ30127AAAF	182	19.59	[NAP1L5]
FLJ30131AAAF	180	19.76	[CCDC115]
FLJ30134AAAF	539	58.56	[MAP4]
FLJ30140AAAF	248	29.02	[MRO]
FLJ30166AAAF	138	14.89	[CALCB]
FLJ30169AAAF	448	49.92	[TUBA4A]
FLJ30201AAAN	311	35.83	[TRAK2]
FLJ30213AAAF	127	14.3	[YPEL4]
FLJ30225AAAF	618	68.24	[NFKBIZ]
FLJ30231AAAF	198	20.82	[C1orf159]
FLJ30235AAAN	327	35.07	[BARHL1]
FLJ30240AAAF	297	34.65	[TRIM52]
FLJ30246AAAN	336	36.3	[PLK5]
FLJ30254AAAF	183	21.23	[FTH1]
FLJ30259AAAF	285	31.69	[NEURL2]

FLJ30273AAAF	316	35.09	[RDH12]
FLJ30277AAAF	200	21.36	[WWC2-AS2]
FLJ30279AAAF	202	22.29	[HAGHL]
FLJ30296AAAF	783	89.37	[PTCHD1]
FLJ30313AAAF	134	14.38	[C20orf166-AS1]
FLJ30319AAAF	261	30.03	[CALB1]
FLJ30320AAAF	724	81.32	[ACSBG1]
FLJ30322AAAF	361	41.51	[SURF6]
FLJ30323AAAF	306	31.44	[PRRT1]
FLJ30336AAAF	94	11.02	[LINC00312]
FLJ30356AAAN	244	26.75	[C6orf141]
FLJ30358AAAF	219	23.69	[TPPP]
FLJ30359AAAF	130	14.84	[RPS15A]
FLJ30373AAAF	192	19.48	[TTC9B]
FLJ30394AAAF	376	42.5	[PHYHIPL]
FLJ30395AAAF	287	32.31	[NRBF2]
FLJ30396AAAF	115	12.41	[TIAF1]
FLJ30398AAAF	237	25.84	[SRA1]
FLJ30431AAAF	192	21.45	[RAC1]
FLJ30458AAAF	368	42.76	[NAP1L1]
FLJ30466AAAF	269	30.07	[LDLRAD4]
FLJ30469AAAF	208	22.24	[LYPD5]
FLJ30470AAAF	607	66.38	[RAP1GDS1]
FLJ30473AAAF	605	66.79	[AIFM3]
FLJ30479AAAF	252	28.31	[EMX2]
FLJ30480AAAF	368	40.9	[PRR5L]
FLJ30489AAAF	525	57.42	[SLC32A1]
FLJ30496AAAF	462	50.14	[EEF1A1]
FLJ30499AAAF	654	72.81	[SLC39A12]
FLJ30506AAAF	200	21.45	[TPD52L2]
FLJ30515AAAF	526	59.9	[SGK1]
FLJ30524AAAF	445	52.26	[MTRF1]
FLJ30525AAAF	393	44.56	[HENMT1]
FLJ30530AAAF	519	59.57	[GTF3C5]
FLJ30532AAAF	457	51.85	[MARVELD2]
FLJ30542AAAF	756	79.34	[SH2B1]
FLJ30545AAAF	248	26.72	[ADARB2]
FLJ30549AAAF	607	69.57	[GALNT18]
FLJ30567AAAF	122	14.44	[C10orf25]
FLJ30570AAAF	579	62.23	[MFRP]
FLJ30573AAAF	488	51.6	[TOX2]
FLJ30574AAAF	501	55.9	[RFTN2]
FLJ30596AAAF	279	31.72	[NADK2]
FLJ30600AAAF	207	23.38	[C8orf37]
FLJ30603AAAF	457	50.6	[CHIT1]
FLJ30605AAAF	137	14.98	[ZNF295-AS1]
FLJ30614AAAN	1095	123.32	[FANCI]
FLJ30621AAAF	688	76.69	[C1S]

FLJ30651AAAF	450	47.24	[RBFOX2]
FLJ30656AAAN	195	21.7	[LSM12]
FLJ30663AAAF	588	67.49	[ZNF599]
FLJ30666AAAF	522	58.22	[PAK1]
FLJ30668AAAF	305	33.34	[TMEM74]
FLJ30671AAAF	473	52.79	[RUNDC3B]
FLJ30675AAAF	151	16.43	[CENPT]
FLJ30676AAAF	337	38.7	[NEUROD6]
FLJ30689AAAF	779	84.5	[BCL11A]
FLJ30697AAAF	373	40.73	[FBLIM1]
FLJ30701AAAF	371	43.12	[ZNF707]
FLJ30715AAAF	314	36.83	[ELOVL4]
FLJ30723AAAF	346	37.43	[ETFB]
FLJ30726AAAF	502	57.66	[ZFP3]
FLJ30730AAAF	620	70.35	[KLHL32]
FLJ30743AAAN	688	78.65	[KRIT1]
FLJ30756AAAF	516	59.66	[ZNF83]
FLJ30760AAAF	399	43.78	[PNMA6A]
FLJ30763AAAF	364	41.51	[PNMA2]
FLJ30764AAAF	193	22.22	[NKAIN2]
FLJ30771AAAF	363	39.43	[INO80B]
FLJ30784AAAF	372	41.06	[ACTRT3]
FLJ30803AAAF	719	79.48	[LRFN5]
FLJ30805AAAF	168	18.66	[DTD2]
FLJ30817AAAF	543	60.64	[MCM7]
FLJ30818AAAF	383	40.55	[DLK2]
FLJ30826AAAF	802	89.79	[TRIM9]
FLJ30829AAAF	191	19.6	[RBM24]
FLJ30831AAAF	580	62.85	[AGAP3]
FLJ30833AAAF	508	56.8	[MATK]
FLJ30834AAAF	130	14.25	[TMEM155]
FLJ30839AAAF	632	72.24	[DOCK7]
FLJ30841AAAF	577	65.92	[OPTN]
FLJ30845AAAF	306	34.43	[RSPH9]
FLJ30877AAAF	115	13.51	[C12orf79]
FLJ30879AAAF	364	39.53	[OSGEPL1]
FLJ30880AAAF	825	92.51	[BCAR3]
FLJ30881AAAN	135	15.85	[FAM107A]
FLJ30889AAAF	433	46.59	[RUSC1]
FLJ30894AAAN	531	59.37	[NSUN2]
FLJ30909AAAF	816	91.9	[NLGN4X]
FLJ30910AAAF	248	27.81	[NANP]
FLJ30927AAAF	517	60.5	[ZNF582]
FLJ30929AAAF	638	71.46	[DYNC1I2]
FLJ30932AAAF	402	45.75	[ZNF558]
FLJ30936AAAF	672	76.67	[TTC25]
FLJ30946AAAF	792	90.6	[RINT1]
FLJ30954AAAF	914	105.6	[NRDC]

FLJ30968AAAF	458	50.38	[MLIP]
FLJ30970AAAF	270	29.22	[LHPP]
FLJ30972AAAF	230	25.56	[C8orf49]
FLJ30973AAAF	466	54.23	[MYZAP]
FLJ30977AAAF	374	42.28	[LAYN]
FLJ30986AAAF	541	56.93	[SLC2A10]
FLJ30990AAAF	638	73.15	[TTC30B]
FLJ30993AAAF	456	52.15	[METTL14]
FLJ30994AAAF	388	42.2	[SOX7]
FLJ30996AAAF	767	89.91	[MAATS1]
FLJ31011AAAF	172	19.38	[TBC1D20]
FLJ31025AAAF	309	34.16	[GATSL3]
FLJ31029AAAF	517	59.16	[TBC1D22A]
FLJ31030AAAN	569	66.04	[ZNF583]
FLJ31036AAAF	717	77.81	[FBXO42]
FLJ31051AAAF	377	43.08	[IKBIP]
FLJ31056AAAF	272	30.77	[FAM210A]
FLJ31073AAAF	303	34.09	[MEDAG]
FLJ31076AAAF	624	67.68	[AFF4]
FLJ31080AAAF	478	54.11	[CAMK2D]
FLJ31082AAAF	262	30.68	[ZNF138]
FLJ31100AAAF	353	40.48	[ZNF547]
FLJ31101AAAF	461	51.09	[SLC30A6]
FLJ31103AAAF	359	40.92	[MAB21L2]
FLJ31105AAAF	297	32.76	[FAM212B]
FLJ31106AAAF	198	21.82	[COMMD9]
FLJ31111AAAF	340	38.21	[SHD]
FLJ31121AAAF	199	23.58	[ZMAT2]
FLJ31128AAAN	367	42.48	[PPHLN1]
FLJ31129AAAF	292	33.3	[BCCIP]
FLJ31139AAAF	251	29.26	[C3orf33]
FLJ31140AAAF	481	55.61	[PUS3]
FLJ31142AAAF	211	23.95	[DUSP26]
FLJ31145AAAF	372	41.99	[ZNF830]
FLJ31146AAAF	406	45.15	[HERPUD2]
FLJ31153AAAF	174	19.78	[FOPNL]
FLJ31158AAAF	295	31.13	[C9orf66]
FLJ31164AAAF	513	55.83	[TSNARE1]
FLJ31165AAAN	391	42.9	[RANBP10]
FLJ31166AAAF	163	17.91	[TMEM52B]
FLJ31171AAAF	297	33.17	[MPST]
FLJ31185AAAF	341	38.09	[DNAJC22]
FLJ31190AAAF	982	110.01	[ICE2]
FLJ31193AAAF	401	44.2	[RILP]
FLJ31204AAAF	532	58.36	[FAAH2]
FLJ31208AAAF	652	74.82	[SPATA13]
FLJ31213AAAF	333	38.26	[ZNF396]
FLJ31216AAAF	1113	126.96	[MYO3B]

FLJ31225AAAF	695	78.2	[VPS16]
FLJ31231SAAF	615	67.66	[FAM83D]
FLJ31236AAAF	628	70.92	[SLC6A18]
FLJ31240AAAN	399	41.91	[AMN]
FLJ31253AAAF	470	52.15	[SLC7A13]
FLJ31254AAAF	518	54.58	[PODXL]
FLJ31279AAAF	107	11.59	[TMEM213]
FLJ31280AAAF	383	43.09	[DMTN]
FLJ31286AAAF	411	45.14	[PRR5]
FLJ31290AAAF	602	65.52	[WAC]
FLJ31293AAAF	226	24.25	[CLDN10]
FLJ31295AAAF	438	49.47	[ZNF641]
FLJ31300AAAF	676	74.8	[UROC1]
FLJ31312AAAN	702	77.3	[SLCO1B3]
FLJ31320AAAN	634	67.97	[PGLYRP2]
FLJ31329AAAF	573	61.92	[TCTN1]
FLJ31331AAAF	181	19.14	[NPHP3]
FLJ31338AAAF	181	19.14	[C18orf15]
FLJ31346AAAF	472	52.77	[MBOAT7]
FLJ31349AAAF	555	61.89	[CNKSR3]
FLJ31364AAAF	570	63.21	[EME1]
FLJ31382AAAF	481	52.41	[DOK1]
FLJ31394AAAF	147	15.42	[LINC00167]
FLJ31409AAAF	545	61.31	[RPUSD2]
FLJ31411AAAF	665	73.06	[DUSP16]
FLJ31422AAAF	770	88.32	[TTC14]
FLJ31432AAAF	343	36.96	[ASPRV1]
FLJ31434AAAF	235	27.02	[MANEAL]
FLJ31436AAAF	524	57.8	[FAM196B]
FLJ31437AAAF	378	42.08	[RFPL2]
FLJ31441AAAF	194	20.43	[CCDC184]
FLJ31444AAAF	555	63.93	[ZNF714]
FLJ31446AAAF	354	39.93	[GNAO1]
FLJ31448AAAF	394	45.49	[ZNF200]
FLJ31450AAAF	288	32.31	[MORF4L2]
FLJ31451AAAF	205	23.08	[LINC00242]
FLJ31452AAAF	345	38.37	[NDEL1]
FLJ31461AAAF	136	15.08	[LINC00052]
FLJ31466AAAF	178	19.54	[RPE]
FLJ31472AAAF	261	30.28	[ZNF664]
FLJ31476AAAF	388	42.27	[MEIS2]
FLJ31479AAAF	456	49.24	[NDUFV3]
FLJ31484AAAF	172	19.25	[SLMO1]
FLJ31494AAAF	349	40.03	[GLT8D2]
FLJ31512AAAF	188	21.07	[DUSP18]
FLJ31521AAAF	579	62.58	[ACADVL]
FLJ31523AAAN	528	59.59	[P3H4]
FLJ31524AAAF	436	49.78	[STAMBPL1]

FLJ31526AAAF	576	66.62	[ZNF681]
FLJ31528AAAF	525	55.14	[ENTHD2]
FLJ31529AAAF	296	34.69	[ZNF75A]
FLJ31531AAAF	523	58.31	[SIAE]
FLJ31532AAAF	417	46	[GPR137]
FLJ31534AAAF	256	27.99	[FAM120AOS]
FLJ31538AAAF	471	53.11	[WARS]
FLJ31541AAAF	228	24.36	[CLDN15]
FLJ31545AAAF	505	54.39	[ANXA11]
FLJ31547AAAF	525	58.2	[CES5A]
FLJ31550AAAF	212	24.32	[CINP]
FLJ31551AAAF	676	77.79	[ZNF418]
FLJ31563AAAF	667	73.52	[STRA6]
FLJ31564AAAF	626	73.21	[JAKMIP1]
FLJ31570AAAF	262	28.92	[TMEM106A]
FLJ31576AAAF	475	52.64	[ERMAP]
FLJ31579AAAF	304	34.77	[PLCXD2]
FLJ31587AAAF	449	50.05	[C7orf26]
FLJ31600AAAF	523	58.6	[KDM4D]
FLJ31605AAAF	447	51.55	[MGAT2]
FLJ31606AAAF	139	15.46	[SPATA33]
FLJ31607AAAF	172	19.21	[TXNDC12]
FLJ31608AAAF	709	80.76	[PDE1C]
FLJ31612AAAF	153	16.97	[ARPC5L]
FLJ31613AAAF	557	62.42	[CPNE4]
FLJ31615AAAF	204	22.38	[VSTM2L]
FLJ31624AAAF	748	84.74	[POMGNT1]
FLJ31631AAAF	254	28.45	[REEP2]
FLJ31633AAAF	752	86.7	[INCENP]
FLJ31638AAAN	618	67.85	[DCP1B]
FLJ31642AAAF	624	69.64	[KEAP1]
FLJ31644AAAF	332	36.83	[NEIL2]
FLJ31646AAAF	808	88.97	[NOP2]
FLJ31652AAAF	530	57.13	[RAI2]
FLJ31659AAAF	157	17.02	[FAM218A]
FLJ31663AAAF	118	12.95	[MTPN]
FLJ31665AAAF	232	25.89	[KCTD11]
FLJ31679AAAF	336	38.44	[ST6GALNAC5]
FLJ31689AAAF	367	41.95	[GDAP1L1]
FLJ31693AAAF	386	42.95	[BCAT1]
FLJ31695AAAF	473	55.36	[RASGEF1B]
FLJ31697AAAF	647	73.69	[FAM161B]
FLJ31701AAAF	758	84.43	[RRP1B]
FLJ31708AAAF	376	43.89	[ST8SIA5]
FLJ31709AAAF	155	17.38	[CHCHD4]
FLJ31711AAAF	346	39.33	[SEPT5]
FLJ31716AAAF	352	39.36	[WNT3A]
FLJ31717AAAN	555	62.81	[DNM3]

FLJ31723AAAF	342	37.85	[WBP1L]
FLJ31724AAAF	436	47.22	[NOL4L]
FLJ31729AAAF	707	79.51	[TMEM260]
FLJ31732AAAN	365	38.96	[STRN4]
FLJ31736AAAF	406	46.1	[KLHDC2]
FLJ31737AAAF	191	21.33	[VSTM4]
FLJ31741AAAF	357	39.74	[WDR92]
FLJ31747AAAF	661	68.97	[EWSR1]
FLJ31748AAAN	455	51.58	[ZAK]
FLJ31751AAAF	618	67.83	[ZNF48]
FLJ31754AAAF	567	67.03	[CEP83]
FLJ31757AAAF	521	57.63	[FAM126A]
FLJ31759AAAF	220	24.96	[ITM2C]
FLJ31760AAAF	230	25.95	[SCNM1]
FLJ31767AAAF	591	67.3	[TIGD1]
FLJ31773AAAF	259	30.12	[DCUN1D1]
FLJ31777AAAF	508	56.08	[EDC3]
FLJ31781AAAN	266	29.94	[WDR4]
FLJ31791AAAF	454	51.78	[PCED1A]
FLJ31795AAAF	224	25.28	[CCDC43]
FLJ31800AAAF	517	59.68	[HINFP]
FLJ31801AAAF	595	69.18	[CCDC151]
FLJ31805AAAF	273	30.18	[SPSB4]
FLJ31810AAAF	606	68.07	[LINGO2]
FLJ31812AAAF	337	39.33	[ZDHHC15]
FLJ31815AAAF	400	45.66	[KIAA1257]
FLJ31816AAAF	801	87.85	[PRDM4]
FLJ31820AAAF	393	43.5	[SH3BP5L]
FLJ31825AAAN	322	35	[C21orf58]
FLJ31827AAAF	406	46.69	[ZNF585A]
FLJ31828AAAF	564	63.63	[KLHL7]
FLJ31829AAAF	800	87.61	[PCDHB10]
FLJ31837AAAF	1003	101.09	[MBD6]
FLJ31840AAAF	557	61.49	[TDRKH]
FLJ31841AAAN	325	35.82	[TMEM255A]
FLJ31842AAAF	263	30.04	[TMEM56]
FLJ31846AAAF	284	32.24	[CCDC122]
FLJ31850AAAF	227	25.54	[PILRB]
FLJ31858AAAF	1204	132.95	[ATP13A1]
FLJ31862AAAF	566	62.77	[RNF220]
FLJ31863AAAF	492	53.78	[KREMEN1]
FLJ31868AAAF	658	76.81	[YTHDC1]
FLJ31871AAAF	782	88	[VAC14]
FLJ31872AAAF	1066	124.18	[TBC1D31]
FLJ31874AAAF	221	24.32	[C17orf51]
FLJ31876AAAF	235	26.1	[SCAMP5]
FLJ31881AAAF	705	79.14	[L3MBTL2]
FLJ31884AAAF	854	98.11	[HSP90AA1]

FLJ31886AAAF	425	48.44	[SYT6]
FLJ31890AAAF	456	53.54	[ZNF25]
FLJ31892AAAF	772	84.8	[NLRX1]
FLJ31895AAAF	718	80.27	[KLHL4]
FLJ31896AAAF	955	108.65	[HAUS6]
FLJ31898AAAF	776	84.98	[PCDHB16]
FLJ31902AAAN	824	92.68	[TTC7A]
FLJ31907AAAF	1054	115.85	[SH3PXD2A]
FLJ31909AAAF	850	93.61	[JADE2]
FLJ31910AAAF	982	111.74	[CFAP44]
FLJ31911AAAF	121	14.19	[SKA2]
FLJ31916AAAN	637	72.42	[MUM1L1]
FLJ31917AAAF	418	47.54	[SPRED2]
FLJ31934AAAF	127	13.9	[C6orf195]
FLJ31936AAAF	752	87.73	[ZNF425]
FLJ31946AAAF	610	70.18	[ZNF667]
FLJ31950AAAF	360	39.83	[TXNDC15]
FLJ31961AAAF	485	56.23	[TRIM68]
FLJ31963AAAF	711	80.96	[DDHD2]
FLJ31964AAAF	547	62.06	[ITPRIP]
FLJ31968AAAF	637	69.3	[NRXN3]
FLJ31970AAAF	423	47.07	[KIAA1715]
FLJ31974AAAF	995	111.75	[SRBD1]
FLJ31977AAAF	493	55.74	[PIGV]
FLJ31978AAAF	266	29.53	[GLT1D1]
FLJ31979AAAF	1047	121.65	[PBRM1]
FLJ31982AAAN	1034	110.72	[ROBO3]
FLJ31986AAAF	790	91.13	[ZNF560]
FLJ31994AAAF	158	17.52	[C1orf145]
FLJ31998AAAF	816	90.77	[CLCN5]
FLJ32000AAAF	698	78.19	[WDR65]
FLJ32001AAAF	725	79.57	[CNST]
FLJ32005AAAF	443	46.38	[HOXA3]
FLJ32009AAAF	955	99.88	[VWCE]
FLJ32014AAAF	277	30.24	[LSP1]
FLJ32018AAAF	117	13.16	[UBL3]
FLJ32028AAAF	183	20.5	[TMEM154]
FLJ32029AAAF	622	70.43	[PGM2L1]
FLJ32033AAAF	839	94.48	[ATG9A]
FLJ32039AAAF	447	49.55	[C2orf54]
FLJ32066AAAF	1200	134.49	[SORBS2]
FLJ32069AAAF	289	33.13	[KCTD7]
FLJ32071AAAF	674	76.15	[KBTBD6]
FLJ32075AAAF	696	79.88	[NUP93]
FLJ32076AAAF	733	82.58	[SATB2]
FLJ32079AAAF	696	79.36	[ZSWIM3]
FLJ32082AAAF	522	58.63	[LRRTM1]
FLJ32086AAAF	429	47.88	[PNPLA5]

FLJ32087AAAF	660	73.96	[FLRT2]
FLJ32090AAAF	606	68.17	[CPSF3L]
FLJ32091AAAF	707	81.47	[TARSL2]
FLJ32093AAAF	585	64.9	[CSRNP3]
FLJ32104AAAF	340	36.96	[ZNF488]
FLJ32112AAAF	208	24.15	[TCEANC2]
FLJ32115AAAF	273	30.48	[ERP27]
FLJ32116AAAF	269	28.89	[CELA2A]
FLJ32132AAAF	530	62.06	[ZNF257]
FLJ32138AAAF	446	49.67	[SMOC2]
FLJ32144AAAF	605	69.1	[HS6ST2]
FLJ32145AAAF	1461	156.1	[RPRD2]
FLJ32149AAAF	609	69.44	[POGK]
FLJ32150AAAF	686	75.67	[HSPA12B]
FLJ32151AAAF	602	66.81	[SLC13A3]
FLJ32157AAAF	310	34.73	[ZFP42]
FLJ32176AAAF	841	90.6	[DIP2A]
FLJ32178AAAF	533	58.47	[MOCS1]
FLJ32180AAAF	360	40.42	[WNT2]
FLJ32189AAAF	242	27.21	[CYB561A3]
FLJ32191AAAN	536	62.96	[ZNF420]
FLJ32198AAAF	265	28.51	[UPK3B]
FLJ32208SAAF	715	78.64	[FCHO2]
FLJ32210AAAF	693	75.59	[VIT]
FLJ32226AAAN	332	36.84	[ZP3]
FLJ32227AAAF	541	61.4	[FAM20A]
FLJ32228AAAF	544	64.03	[CCDC82]
FLJ32232AAAF	363	42.15	[CYP11A1]
FLJ32234AAAF	144	16.53	[SNRNP48]
FLJ32239AAAF	128	14.63	[FAM26D]
FLJ32261AAAF	728	80.69	[LNX1]
FLJ32262AAAF	251	27.19	[HDGFL1]
FLJ32280AAAF	410	45.95	[MARVELD3]
FLJ32295AAAF	452	45.84	[SP8]
FLJ32300AAAF	660	76.27	[MPHOSPH10]
FLJ32312AAAF	529	60.21	[PUS10]
FLJ32326AAAN	521	56.31	[BAIAP2]
FLJ32332AAAF	270	30.37	[PPM1M]
FLJ32343AAAN	740	85.69	[CWF19L2]
FLJ32347AAAF	735	76.12	[PPP1R3F]
FLJ32350AAAF	322	36.6	[KCTD10]
FLJ32353AAAF	322	35.62	[SFXN1]
FLJ32358AAAN	537	59.15	[FPGS]
FLJ32361AAAF	756	85.25	[ELMO3]
FLJ32362AAAF	697	76.85	[TCTN2]
FLJ32363AAAF	638	72.88	[C5orf34]
FLJ32369AAAF	338	37.48	[ERCC8]
FLJ32370AAAF	257	29.54	[TMEM68]

FLJ32372AAAF	211	23.99	[RILPL2]
FLJ32374AAAN	202	19.86	[EHMT2]
FLJ32375AAAF	116	12.95	[SAMD13]
FLJ32376AAAF	337	37.08	[APOL2]
FLJ32378AAAF	421	46.65	[SEH1L]
FLJ32380AAAF	353	40.25	[TRIM63]
FLJ32384AAAF	286	32.42	[HEXIM2]
FLJ32388AAAF	227	26.78	[IDI2]
FLJ32389AAAF	160	17.14	[HSPB6]
FLJ32390AAAF	357	40.66	[PCMTD1]
FLJ32393AAAF	369	40.93	[COQ3]
FLJ32395AAAF	304	34.31	[NDNL2]
FLJ32405AAAN	325	36.49	[COPS5]
FLJ32406AAAF	250	29.74	[TNNT3]
FLJ32416AAAF	331	36.32	[JSRP1]
FLJ32418AAAF	477	54.04	[NARS2]
FLJ32421AAAF	411	46.46	[BROX]
FLJ32422AAAF	218	25.33	[POLR3GL]
FLJ32423AAAF	117	12.41	[MIR1-1HG]
FLJ32424AAAN	355	41.64	[FBXO32]
FLJ32431AAAN	237	27.51	[DCUN1D5]
FLJ32440AAAN	247	27.9	[NSMCE2]
FLJ32441AAAF	247	27.63	[COQ10A]
FLJ32442AAAF	289	33.69	[C9orf78]
FLJ32443AAAF	264	29.4	[PSMG2]
FLJ32444AAAF	283	31.82	[RPP38]
FLJ32446AAAF	293	33.24	[CCDC101]
FLJ32447AAAF	163	18.25	[CCDC140]
FLJ32451AAAF	364	41.51	[STAC3]
FLJ32464AAAF	766	85.67	[ABCB6]
FLJ32466AAAF	567	64.61	[ZNF512]
FLJ32467AAAF	562	61.95	[TRMT2A]
FLJ32479AAAF	456	50.11	[SLC29A2]
FLJ32480AAAF	668	73.16	[FBXL18]
FLJ32487AAAF	377	41.97	[PTGES2]
FLJ32497AAAF	401	44.71	[KCNAB1]
FLJ32504AAAF	523	59.15	[UGT3A1]
FLJ32509AAAF	179	20.17	[C9orf85]
FLJ32510AAAF	491	52.94	[LILRB5]
FLJ32515AAAF	147	15.01	[C16orf13]
FLJ32532AAAF	377	43.21	[CASP4]
FLJ32536AAAN	948	104.04	[AKAP2]
FLJ32538AAAF	299	34.29	[ST6GALNAC6]
FLJ32543AAAF	483	55.88	[BTK]
FLJ32549AAAF	445	50.41	[C12orf66]
FLJ32576AAAF	376	42.62	[SERPINB6]
FLJ32577AAAF	213	24.65	[NOL12]
FLJ32597AAAF	270	29.85	[C1orf147]

FLJ32603AAAF	540	58.33	[ITFG3]
FLJ32604AAAF	482	53.29	[FAM149A]
FLJ32610AAAN	490	53	[HIAT1]
FLJ32614AAAF	420	47.75	[CCBL2]
FLJ32618AAAF	204	23.73	[C16orf45]
FLJ32622AAAF	385	41.88	[PRICKLE4]
FLJ32623AAAF	141	15.01	[C19orf12]
FLJ32630AAAF	657	75.04	[C18orf8]
FLJ32640AAAF	597	70.19	[UTP6]
FLJ32642AAAF	397	44.34	[VPS37A]
FLJ32647AAAF	441	50.37	[ZNF773]
FLJ32655AAAF	621	69.81	[LOC100653515]
FLJ32658AAAF	562	62.78	[CCDC155]
FLJ32660AAAF	740	87.08	[DRC1]
FLJ32661AAAF	379	41.61	[ANHX]
FLJ32662AAAF	614	69.52	[KLHL10]
FLJ32663AAAF	605	68.06	[USP2]
FLJ32667AAAF	560	63.2	[AATF]
FLJ32670AAAF	457	52.47	[LOXHD1]
FLJ32675AAAF	349	39.15	[HARBI1]
FLJ32682AAAF	690	80.92	[ERICH6B]
FLJ32685AAAF	484	54.22	[NEK10]
FLJ32688AAAN	648	71.32	[PPP6R3]
FLJ32693AAAF	260	28.82	[UBQLNL]
FLJ32702AAAF	395	43.44	[C16orf46]
FLJ32704AAAF	479	54.93	[AK8]
FLJ32711AAAN	342	36.21	[DMRTB1]
FLJ32715AAAF	1081	118.73	[IPO4]
FLJ32724AAAF	296	33.37	[PACRG]
FLJ32725AAAF	514	60.18	[TMEM117]
FLJ32730AAAF	519	59.09	[RIOK3]
FLJ32732AAAF	766	86.6	[FGD4]
FLJ32733AAAF	170	18.61	[HSPB7]
FLJ32734AAAF	316	38.04	[CCDC42]
FLJ32736AAAF	604	69.78	[KLHL15]
FLJ32737AAAF	647	69	[DLAT]
FLJ32739AAAF	847	96.91	[USP32]
FLJ32741AAAF	576	64.41	[ADAD1]
FLJ32745AAAF	665	76.25	[CCDC138]
FLJ32756AAAF	177	19.6	[TBC1D22A-AS1]
FLJ32763AAAF	626	67.83	[TKTL2]
FLJ32771AAAF	425	47.28	[PPP1R32]
FLJ32777AAAF	366	41.38	[ACTL8]
FLJ32780AAAF	873	98.31	[TTC16]
FLJ32781AAAF	589	67.32	[ZNF131]
FLJ32785AAAF	706	80.68	[DCST1]
FLJ32786AAAF	825	93.62	[LRGUK]
FLJ32787AAAF	217	24.96	[C22orf23]

FLJ32790AAAN	194	20.62	[LOC642696]
FLJ32799AAAF	343	38.52	[MAGEB18]
FLJ32800AAAF	508	59.97	[FAM227B]
FLJ32807AAAF	183	20.71	[FATE1]
FLJ32808AAAF	440	50.24	[ENPP6]
FLJ32809AAAF	250	27.9	[NOXRED1]
FLJ32820AAAF	351	39.37	[TBATA]
FLJ32822AAAF	365	41.84	[ZMYND12]
FLJ32825AAAF	656	75.24	[CCDC27]
FLJ32828AAAF	490	56.65	[TEKT3]
FLJ32829AAAN	615	71.28	[TEX11]
FLJ32830AAAF	570	65.77	[HEATR9]
FLJ32831AAAF	178	20.26	[C15orf32]
FLJ32835AAAF	142	15.26	[LINC00479]
FLJ32839AAAF	513	56.42	[DPEP3]
FLJ32841AAAF	647	69.93	[ASPSCR1]
FLJ32858AAAF	335	37.37	[NSUN4]
FLJ32859AAAF	536	60.19	[C3orf30]
FLJ32861AAAN	433	49.54	[DHX57]
FLJ32864AAAF	723	82.59	[AK7]
FLJ32870AAAF	154	18.25	[IQCF3]
FLJ32871AAAF	485	56.22	[TEKT5]
FLJ32875AAAF	445	46.7	[PRRC1]
FLJ32879AAAF	346	39.18	[MAGEB3]
FLJ32880AAAF	556	62.39	[TSGA10IP]
FLJ32883AAAF	441	49.95	[ZBTB26]
FLJ32892AAAN	572	67.07	[CNBD2]
FLJ32894AAAF	166	18.19	[LINC00477]
FLJ32897AAAF	395	43.02	[CABS1]
FLJ32898AAAF	706	80.61	[ZNF132]
FLJ32900AAAF	143	16	[IGF2BP2-AS1]
FLJ32901AAAF	334	38.11	[PLEKHA1]
FLJ32915AAAF	299	34.38	[HYLS1]
FLJ32917AAAF	140	15.45	[TPD52L3]
FLJ32920AAAN	416	48.02	[SPAG1]
FLJ32926AAAF	463	50.38	[CCDC114]
FLJ32934AAAF	639	71.78	[DCST2]
FLJ32942AAAF	167	19.27	[GTSF1]
FLJ32945AAAF	544	61.54	[BTBD9]
FLJ32947AAAN	733	80.65	[NCAM1]
FLJ32949AAAF	758	87.36	[DPY19L2]
FLJ32954AAAF	573	65.23	[RMDN2]
FLJ32962AAAF	173	19.85	[PPP3R2]
FLJ32965AAAF	347	38.88	[MAGEB10]
FLJ32968AAAF	628	71.79	[TTC30A]
FLJ32969AAAF	184	21.85	[IMP3]
FLJ32979AAAF	176	19.44	[DR1]
FLJ32981AAAF	605	64.73	[FAM71B]

FLJ33021AAAF	272	29.38	[PPP4R1L]
FLJ33047AAAF	380	41.83	[CCDC74B]
FLJ33049AAAF	284	32.75	[FHL5]
FLJ33051AAAF	313	34.75	[BCAP31]
FLJ33066AAAF	266	30.42	[GJB4]
FLJ33069AAAF	276	30.61	[TMEM71]
FLJ33071AAAF	424	48.06	[ZNF597]
FLJ33087AAAF	501	57.26	[ACCS]
FLJ33098AAAN	481	53.21	[BBS9]
FLJ33114AAAN	190	21.65	[RBM18]
FLJ33126AAAF	182	20.38	[HINT3]
FLJ33129AAAF	230	25.57	[THTPA]
FLJ33132AAAF	692	79.35	[MCTP1]
FLJ33138AAAF	213	22.02	[ATP6V0E2]
FLJ33140AAAF	406	47.35	[ZSCAN31]
FLJ33154AAAF	228	25.59	[C9orf89]
FLJ33167AAAF	560	64.41	[PRIMPOL]
FLJ33218AAAN	603	69.01	[HIP1R]
FLJ33239AAAF	206	23.08	[C5orf30]
FLJ33240AAAF	544	59.37	[SYBU]
FLJ33245AAAF	420	48.43	[DCP2]
FLJ33253AAAF	555	63.24	[FRMD5]
FLJ33282AAAF	400	44.6	[TMEM237]
FLJ33285AAAF	456	49.23	[KRT15]
FLJ33291AAAF	356	38.31	[EP400NL]
FLJ33296AAAF	456	50.22	[SLC29A1]
FLJ33298AAAN	414	45.73	[WDR62]
FLJ33299AAAF	311	34.55	[TMBIM1]
FLJ33300AAAF	648	71.92	[AVL9]
FLJ33305AAAF	670	74.58	[TRAF7]
FLJ33307AAAF	154	16.18	[LOC100127940]
FLJ33311AAAF	119	13.77	[UTS2B]
FLJ33312AAAF	726	79.44	[NHLRC2]
FLJ33315AAAF	620	65.92	[GLIS1]
FLJ33318AAAF	159	17.24	[TMEM92]
FLJ33319AAAF	381	42.81	[RNF13]
FLJ33320AAAN	619	69.57	[NFASC]
FLJ33325AAAF	161	17.93	[THY1]
FLJ33331AAAF	215	23.38	[NACA]
FLJ33334AAAF	558	63.98	[PHOSPHO2-KLHL23]
FLJ33352AAAF	625	71.47	[SNRNP200]
FLJ33353AAAF	419	46.87	[SYT2]
FLJ33365AAAF	294	31.74	[KCP]
FLJ33387AAAF	167	19.53	[TMEM229B]
FLJ33392AAAF	408	45.58	[S100PBP]
FLJ33393AAAF	340	37.75	[HAPLN2]
FLJ33397AAAF	454	49.16	[CLASP2]
FLJ33409AAAF	270	30.45	[FAIM2]

FLJ33414AAAF	293	33	[HMG20B]
FLJ33427AAAF	447	50.5	[GDI1]
FLJ33437AAAF	416	47.29	[RNF180]
FLJ33450AAAF	580	66.31	[PHACTR1]
FLJ33455AAAF	777	88.13	[PLCD1]
FLJ33456AAAF	205	23.51	[MRAP2]
FLJ33461AAAF	554	61.62	[GABRA4]
FLJ33471AAAF	162	18.02	[UMODL1-AS1]
FLJ33496AAAF	320	35.61	[UNC80]
FLJ33505AAAF	294	33.69	[DEPDC4]
FLJ33534AAAF	174	19.08	[ADGRF3]
FLJ33545AAAF	162	18.95	[RNF170]
FLJ33562AAAF	428	48.6	[LRRC42]
FLJ33580AAAF	252	28.27	[PLD6]
FLJ33590AAAF	572	60.55	[RTP5]
FLJ33596AAAF	107	12.07	[NAP1L6]
FLJ33599AAAF	405	46.03	[NT5DC1]
FLJ33603AAAF	227	24.01	[PTRH1]
FLJ33608AAAF	269	29.68	[EFCAB2]
FLJ33610AAAF	174	18.33	[LINC00269]
FLJ33636AAAN	315	37.62	[FRA10AC1]
FLJ33640AAAN	626	72.29	[MAD1L1]
FLJ33641AAAF	157	17.88	[GAPT]
FLJ33655AAAF	295	32.15	[EPHA10]
FLJ33671AAAF	243	27.29	[RCAN2]
FLJ33682AAAF	575	63.52	[FAM198A]
FLJ33703AAAF	199	23.42	[C4orf33]
FLJ33706AAAF	194	21.26	[C20orf203]
FLJ33711AAAF	106	12.07	[WDR83OS]
FLJ33719AAAF	484	51.57	[NEU4]
FLJ33720AAAF	643	73.06	[ZNF23]
FLJ33725AAAF	264	28.24	[PSMG3-AS1]
FLJ33728AAAF	110	11.67	[ANAPC16]
FLJ33737AAAF	580	65.7	[NELFB]
FLJ33741AAAN	267	30.24	[NDUFAF5]
FLJ33745AAAF	445	48.34	[SLC14A1]
FLJ33758AAAF	1217	134.61	[CTC1]
FLJ33761AAAF	522	59.14	[TTC39C]
FLJ33763AAAF	320	34.23	[HNRNPA1L2]
FLJ33779AAAF	408	45.21	[ZSCAN1]
FLJ33788AAAF	130	13.91	[ST20-AS1]
FLJ33801AAAF	1001	109.81	[ANKRD35]
FLJ33803AAAF	173	18.47	[HES2]
FLJ33810AAAF	138	16.05	[C12orf36]
FLJ33811AAAF	1077	118.61	[ZNF827]
FLJ33812AAAF	1123	119.23	[SREBF1]
FLJ33815AAAF	796	90.25	[LRRC8E]
FLJ33821AAAN	916	100.92	[CLASP1]

FLJ33825AAAF	634	71.36	[ATAD3A]
FLJ33826AAAF	176	19.64	[KXD1]
FLJ33850AAAF	351	38.94	[TMEM30B]
FLJ33851AAAF	168	18.13	[CDKN2C]
FLJ33856AAAF	834	91.91	[PROM2]
FLJ33860AAAF	126	14.46	[C20orf197]
FLJ33861AAAF	194	21.86	[RAB22A]
FLJ33862AAAN	1031	116.16	[MPHOSPH9]
FLJ33863AAAF	816	93.07	[ARHGAP12]
FLJ33868AAAF	616	70.09	[TTC39B]
FLJ33871AAAF	246	27.29	[SLC25A45]
FLJ33879AAAF	436	50.58	[PSMD12]
FLJ33884AAAF	816	93.99	[ZNF484]
FLJ33887AAAF	1034	115.03	[TSHZ2]
FLJ33896AAAF	305	35.41	[ST6GALNAC3]
FLJ33900AAAF	308	33.87	[LDLRAP1]
FLJ33901AAAF	126	13.91	[HIST3H2BB]
FLJ33902AAAF	388	42.8	[FAM199X]
FLJ33903AAAF	444	50.45	[SPRED1]
FLJ33913AAAF	107	12.23	[LINC01588]
FLJ33924AAAF	461	51.39	[USH1G]
FLJ33930AAAF	196	21.39	[ARL11]
FLJ33933AAAF	732	83.36	[CTAGE5]
FLJ33937AAAF	669	75.94	[DYM]
FLJ33947AAAF	795	86.47	[MIB1]
FLJ33965AAAF	682	77.08	[NOL9]
FLJ33976AAAF	695	81.17	[LMBRD2]
FLJ33977AAAF	298	33.42	[DTWD2]
FLJ33978AAAN	533	60.82	[IKBKB]
FLJ33979AAAF	273	30.85	[METTL15]
FLJ33990AAAF	364	39.71	[SLC35G1]
FLJ33991AAAF	452	50.82	[TRIM55]
FLJ33992AAAF	243	27.08	[JAZF1]
FLJ33998AAAF	1047	116.02	[PSD3]
FLJ33999AAAF	416	46.94	[MKRN2]
FLJ34032AAAF	520	61.24	[SAMD3]
FLJ34064AAAF	859	97.73	[FANCB]
FLJ34065AAAF	893	97.93	[SPATA5]
FLJ34074AAAF	1344	147.46	[SETD5]
FLJ34093AAAF	903	103.3	[THAP9]
FLJ34126AAAF	417	45.14	[TSPYL5]
FLJ34135AAAF	250	27.74	[C3orf70]
FLJ34143AAAN	323	32.38	[OLIG2]
FLJ34144AAAF	419	45.81	[NOL4]
FLJ34148AAAF	619	67.24	[RNF157]
FLJ34155AAAF	378	44.03	[B3GNT5]
FLJ34169AAAF	763	83.19	[PFKL]
FLJ34181AAAF	718	82.01	[ERCC3]

FLJ34182AAAF	261	30.11	[RCHY1]
FLJ34190AAAN	181	20.32	[RNF175]
FLJ34198AAAF	555	62.29	[SYT14]
FLJ34203AAAF	869	94.08	[SEZ6]
FLJ34204AAAF	770	88.22	[BANK1]
FLJ34227AAAF	766	87.36	[WDR75]
FLJ34230AAAN	623	73.37	[IFT81]
FLJ34245AAAF	240	27.94	[C6orf203]
FLJ34255AAAF	264	29.26	[RNF2]
FLJ34259AAAF	601	68.2	[KIAA1598]
FLJ34263AAAF	297	33.61	[TMEM169]
FLJ34270AAAF	723	83.58	[LRSAM1]
FLJ34272AAAF	920	107.58	[ANO4]
FLJ34283AAAF	220	23.3	[PDDC1]
FLJ34297AAAN	215	23.74	[MAPKBP1]
FLJ34299AAAF	586	68.49	[ZNF92]
FLJ34304AAAF	206	22.2	[NDUFS7]
FLJ34324AAAN	239	27.67	[FGF17]
FLJ34327AAAF	721	80.4	[EIF2B5]
FLJ34363AAAF	936	109.59	[FAM184A]
FLJ34368AAAF	415	48.31	[ZNF75D]
FLJ34371AAAN	240	24.64	[MLLT4-AS1]
FLJ34373AAAF	638	73.53	[CLK3]
FLJ34389AAAF	471	54.48	[MLKL]
FLJ34392AAAF	262	29.43	[ZNF511]
FLJ34396AAAF	617	69.43	[KLHL9]
FLJ34402AAAF	488	53.49	[ANTXR2]
FLJ34405AAAF	490	56.63	[SMYD1]
FLJ34407AAAF	183	20.57	[TMEM9]
FLJ34434AAAF	391	43.36	[DUSP6]
FLJ34439AAAN	514	58.2	[SREK1]
FLJ34517AAAF	153	16.69	[LOC100130357]
FLJ34520AAAF	557	63.84	[CAPS2]
FLJ34523AAAF	571	61.93	[DACH2]
FLJ34531AAAF	372	41.35	[YY2]
FLJ34532AAAF	598	68.01	[COL4A3BP]
FLJ34541AAAF	454	51.51	[FBXW2]
FLJ34555AAAF	297	33.43	[TST]
FLJ34556AAAF	309	35.56	[PPP2CB]
FLJ34560AAAF	550	60.3	[SLC22A6]
FLJ34569AAAF	499	55.67	[ETNPPL]
FLJ34575AAAF	308	32.05	[ASRGL1]
FLJ34579AAAN	445	50.04	[SP100]
FLJ34581AAAF	107	12.31	[SMLR1]
FLJ34582AAAF	585	64.06	[FAM151A]
FLJ34592AAAF	469	52.31	[RBBP7]
FLJ34599AAAF	411	46.38	[ATAD3C]
FLJ34606AAAF	190	20.79	[C19orf60]

FLJ34618AAAF	142	15.52	[C1orf115]
FLJ34621AAAF	192	21.68	[RNF183]
FLJ34633AAAF	398	43.58	[KDF1]
FLJ34634AAAF	759	83.18	[IQSEC3]
FLJ34640AAAN	757	84.23	[TBCD]
FLJ34641AAAF	132	14.72	[DUSP15]
FLJ34642AAAF	673	73.74	[UMOD]
FLJ34645AAAF	273	29.63	[EGFL7]
FLJ34646AAAF	333	38.64	[GLYATL1]
FLJ34647AAAF	386	41.86	[PDZD3]
FLJ34651AAAF	159	17.24	[ADORA2A-AS1]
FLJ34668AAAF	457	50.27	[UNC93A]
FLJ34674AAAF	532	59.48	[CFAP97]
FLJ34682AAAF	308	33.44	[SLC25A47]
FLJ34689AAAF	1143	130.99	[NCAPG2]
FLJ34693AAAF	561	65.42	[ZNF37A]
FLJ34698AAAF	262	29.59	[SPIN1]
FLJ34702AAAN	1036	113.22	[COL6A3]
FLJ34711AAAN	713	82.16	[PUS7L]
FLJ34719AAAN	312	37.46	[PRPF38A]
FLJ34739AAAF	470	52.12	[RMDN3]
FLJ34774AAAF	508	57.56	[FARSA]
FLJ34783AAAF	137	15.53	[FNDC5]
FLJ34788AAAF	297	31.86	[AMZ1]
FLJ34794AAAN	721	82.58	[TUBGCP2]
FLJ34802AAAF	365	41.53	[SYTL2]
FLJ34828AAAF	580	66.62	[POMGNT2]
FLJ34836AAAN	161	17.4	[C5orf17]
FLJ34837AAAN	458	52	[HDAC2]
FLJ34885AAAF	223	25.52	[DNAJB9]
FLJ34895AAAF	194	21.7	[C15orf57]
FLJ34897AAAF	480	52.29	[PTBP2]
FLJ34902AAAF	370	40.75	[FAM110B]
FLJ34911AAAF	448	48.68	[GABPB2]
FLJ34912AAAF	589	65.94	[KLHL25]
FLJ34913AAAF	617	53.63	[ELN]
FLJ34917AAAF	627	72.86	[ZNF549]
FLJ34919AAAF	858	98.27	[LRRC8D]
FLJ34920AAAF	932	97.4	[RBM12]
FLJ34921AAAF	397	46.51	[ZNF763]
FLJ34922AAAF	901	102.81	[SLFN11]
FLJ34923AAAF	530	58.08	[SLC29A4]
FLJ34924AAAF	489	53.04	[PHF21B]
FLJ34934AAAF	799	92.03	[ZNF227]
FLJ34938AAAF	508	59.02	[CAPN14]
FLJ34940AAAF	409	47.47	[ZNF286A]
FLJ34942AAAF	437	49.92	[ZNF154]
FLJ34944AAAF	122	13.19	[FBXL19-AS1]

FLJ34945AAAF	132	14.17	[LOC100128265]
FLJ34946AAAF	299	30.65	[PRRT2]
FLJ34947AAAN	781	90.27	[ZNF616]
FLJ34950AAAN	416	47.34	[CSNK1E]
FLJ34954SAAN	449	49.23	[HNRNPH1]
FLJ34960AAAF	644	70.61	[KLHL34]
FLJ34970AAAF	1166	124.82	[CASZ1]
FLJ34972AAAF	903	99.16	[ASAP3]
FLJ34980AAAF	569	63.53	[RABEP2]
FLJ34982AAAF	127	14.07	[LIMD2]
FLJ34983AAAF	745	78.99	[ISLR2]
FLJ34984AAAF	316	35.39	[KIAA0895L]
FLJ34985AAAF	742	82.7	[EPC1]
FLJ34988AAAF	354	38.78	[NEGR1]
FLJ34991AAAF	482	54.41	[ICA1L]
FLJ35007AAAF	512	55.56	[ZBTB8B]
FLJ35022AAAF	553	61.1	[ZNF324]
FLJ35027AAAF	395	42.61	[ALX4]
FLJ35037AAAF	890	100.37	[ACAD10]
FLJ35039AAAF	163	17.85	[GATS]
FLJ35041AAAF	371	42.46	[CCNJ]
FLJ35062AAAN	752	82.88	[VLDLR]
FLJ35064AAAF	601	66.42	[FOXP1]
FLJ35070AAAN	406	41.24	[PYGO2]
FLJ35073AAAN	237	25	[C6orf25]
FLJ35079AAAF	395	42.59	[LAMP1]
FLJ35085AAAF	702	78.15	[IQCE]
FLJ35093AAAN	632	71.01	[FAM73A]
FLJ35095AAAF	156	17.52	[MAFK]
FLJ35097AAAF	429	48.61	[FECH]
FLJ35103AAAF	147	16.84	[RNASE4]
FLJ35105AAAF	433	48.96	[ZSCAN4]
FLJ35107AAAF	258	24.14	[TPRXL]
FLJ35119AAAF	229	24.25	[SWSAP1]
FLJ35129AAAN	727	81.52	[RAPGEF3]
FLJ35130AAAF	473	53.84	[HYAL2]
FLJ35136AAAF	300	35	[ALS2CL]
FLJ35145AAAF	385	42.68	[ALDH3B2]
FLJ35151AAAF	233	25.49	[TMEM40]
FLJ35155AAAF	187	21.82	[XXYLT1]
FLJ35162AAAF	518	56.76	[KLC3]
FLJ35165AAAF	214	24.19	[TANGO2]
FLJ35176AAAF	274	29.41	[SLC25A26]
FLJ35197AAAF	407	44.55	[IGSF5]
FLJ35213AAAN	501	56.78	[VPS53]
FLJ35219AAAF	487	54.76	[ZIK1]
FLJ35242AAAF	218	23.15	[CLDN5]
FLJ35245AAAF	263	29.7	[ANKRD23]

FLJ35246AAAN	547	61.01	[EPHA6]
FLJ35269AAAF	232	25.11	[C9orf106]
FLJ35273AAAF	108	11.92	[LOC100507462]
FLJ35274AAAF	129	15.02	[ZNF702P]
FLJ35276AAAF	357	39.71	[BNIPL]
FLJ35278AAAF	776	85.33	[PFKP]
FLJ35279AAAF	385	43.02	[PRSS50]
FLJ35283AAAN	262	27.98	[ADAMTSL1]
FLJ35284AAAF	288	33.16	[CENPP]
FLJ35288AAAF	402	44.38	[BSPRY]
FLJ35291AAAF	256	27.38	[AKT1S1]
FLJ35318AAAF	232	25.67	[SMCO3]
FLJ35320AAAF	383	44.29	[CCDC183]
FLJ35323AAAF	247	25.97	[NUDT16L1]
FLJ35326AAAF	200	22.21	[TSC22D3]
FLJ35335AAAF	354	37.74	[FAM84A]
FLJ35337AAAF	549	62.29	[ZNF18]
FLJ35353AAAF	268	30.69	[ANP32E]
FLJ35355AAAF	362	39.24	[NAA30]
FLJ35370AAAF	362	40.09	[SLC25A3]
FLJ35375AAAF	381	43.12	[ABRA]
FLJ35377AAAF	309	33.38	[UBFD1]
FLJ35379AAAF	122	13.24	[LMO7DN]
FLJ35387AAAF	575	62.24	[ANKLE1]
FLJ35392AAAN	149	16.16	[LOC653602]
FLJ35417AAAF	498	61.1	[TCHP]
FLJ35438AAAF	244	26.64	[CD300LF]
FLJ35441AAAF	553	63.74	[ZNF564]
FLJ35444AAAF	640	70.83	[ARHGAP9]
FLJ35446AAAF	625	68.14	[CLINT1]
FLJ35449AAAF	375	39.87	[ADH6]
FLJ35453AAAF	562	60.42	[ZNF579]
FLJ35454AAAF	580	65.82	[ADCK5]
FLJ35458AAAF	211	22.48	[ZNF252P-AS1]
FLJ35487AAAF	123	13.13	[LINC01555]
FLJ35492AAAF	256	29.57	[ZNF483]
FLJ35498AAAF	137	15.7	[AHSA2]
FLJ35499AAAF	364	39.81	[PICK1]
FLJ35539AAAF	739	80.93	[EXOC3L1]
FLJ35558AAAF	1098	124.63	[MYO1F]
FLJ35562AAAF	661	70.26	[PMEL]
FLJ35573AAAF	474	54.16	[TRIM4]
FLJ35588AAAF	791	86.28	[IL17RC]
FLJ35592AAAF	114	12.61	[COX7A2L]
FLJ35649AAAF	602	62.76	[PDLIM2]
FLJ35674AAAF	608	69.37	[KBTBD3]
FLJ35675AAAF	152	16.59	[EGFLAM]
FLJ35681AAAF	224	24.36	[C16orf54]

FLJ35682AAAF	428	49.55	[CLNK]
FLJ35695AAAF	179	19.74	[C15orf53]
FLJ35698AAAF	619	69.07	[ATG16L2]
FLJ35704AAAF	344	39.04	[BGN]
FLJ35712AAAF	356	40.01	[DSN1]
FLJ35713AAAF	652	66.7	[GGN]
FLJ35716AAAF	809	96.33	[CCDC135]
FLJ35721AAAF	138	15.16	[FER1L6-AS1]
FLJ35722AAAF	569	65.2	[SPATA16]
FLJ35725AAAF	365	41.33	[TRMT44]
FLJ35728AAAF	623	72.35	[CCDC185]
FLJ35729AAAF	274	30.08	[THAP8]
FLJ35730AAAF	448	50.63	[SERPINA3]
FLJ35738AAAF	656	75.37	[VPS52]
FLJ35739AAAF	779	89.18	[MMEL1]
FLJ35743AAAF	815	89.91	[OTOA]
FLJ35744AAAF	153	17.49	[ORMDL3]
FLJ35747AAAF	353	39.85	[SSUH2]
FLJ35753AAAF	600	66.46	[DCAF15]
FLJ35754AAAF	662	73.43	[KIAA0226L]
FLJ35755AAAF	728	81.97	[IL17RD]
FLJ35756AAAF	455	52.12	[MOSPD2]
FLJ35757AAAF	808	90.45	[MARCH10]
FLJ35764AAAF	197	21.69	[SIRPD]
FLJ35767AAAF	164	18.47	[TEX19]
FLJ35770AAAF	517	56.13	[OXCT2]
FLJ35773AAAF	586	63.94	[MFSD6L]
FLJ35775AAAF	395	43.75	[DCAF4L2]
FLJ35777AAAF	610	70.04	[CLGN]
FLJ35779AAAN	550	60.84	[POC5]
FLJ35782AAAF	645	73.92	[FAM47B]
FLJ35784AAAF	505	57.05	[C19orf45]
FLJ35789AAAF	542	60.84	[ZNF280A]
FLJ35791AAAF	575	64.9	[ZNF8]
FLJ35793AAAF	363	40.51	[RFFL]
FLJ35794AAAF	552	63.11	[RNF168]
FLJ35796AAAF	447	51.38	[PPP2R2C]
FLJ35802AAAF	436	50.22	[CNBD1]
FLJ35809AAAF	366	41.58	[EIF3H]
FLJ35810AAAF	448	51.57	[SPERT]
FLJ35811AAAF	148	16.6	[C11orf85]
FLJ35817AAAF	518	56.28	[KIAA1919]
FLJ35821AAAF	414	47.35	[C12orf50]
FLJ35826AAAF	501	58.01	[TTC29]
FLJ35827AAAF	889	95.02	[EML3]
FLJ35833AAAF	411	47.1	[CCDC91]
FLJ35834AAAF	791	92.54	[IQUB]
FLJ35837AAAF	259	28.04	[FAM220A]

FLJ35842AAAN	243	27	[FAM71F1]
FLJ35843AAAF	563	66.25	[CCDC63]
FLJ35844AAAF	543	63.04	[ZNF555]
FLJ35854AAAN	504	59.17	[SSX2IP]
FLJ35863AAAF	475	54.67	[ZNF383]
FLJ35879AAAF	601	67.51	[GPD2]
FLJ35882AAAF	471	55.14	[TRIM60]
FLJ35893AAAF	499	57	[ST7]
FLJ35917AAAF	450	49.57	[TEAD2]
FLJ35921AAAF	367	40.84	[WDR31]
FLJ35923AAAF	893	103.7	[FAM186B]
FLJ35932AAAF	514	57.83	[STK33]
FLJ35936AAAF	623	71.19	[L3MBTL4]
FLJ35939AAAF	512	56.76	[UBXN11]
FLJ35951AAAF	505	54.85	[PPM1J]
FLJ35963AAAF	570	65.53	[SAMD15]
FLJ35976AAAF	180	18.29	[RUSC1-AS1]
FLJ35977AAAF	800	91.39	[ZBBX]
FLJ35989AAAF	581	66.12	[FSIP1]
FLJ35993AAAF	143	15.38	[SH2D6]
FLJ35998AAAF	523	61.06	[LRRC48]
FLJ36002AAAF	169	19.1	[C7orf71]
FLJ36004AAAF	388	43.37	[LMNTD1]
FLJ36010AAAF	275	31.78	[TSGA13]
FLJ36017AAAF	396	44.12	[DDI1]
FLJ36018AAAF	269	29.22	[BSG]
FLJ36023AAAF	811	87.48	[ZNF839]
FLJ36025AAAN	579	66.05	[TRAP1]
FLJ36029AAAF	464	54.3	[NUF2]
FLJ36030AAAF	443	52.05	[CHST9]
FLJ36040AAAF	462	53.45	[ZNF610]
FLJ36042AAAF	298	35.32	[SCLT1]
FLJ36046AAAF	515	56.94	[CCDC116]
FLJ36056AAAF	566	63.9	[ERICH6]
FLJ36059AAAF	177	20.59	[DYDC2]
FLJ36066AAAF	192	20.9	[SPATA3]
FLJ36067AAAF	681	72.69	[GAS2L1]
FLJ36068AAAF	354	38.73	[FAM181A]
FLJ36069AAAF	316	35.96	[HMOX2]
FLJ36070AAAF	312	32.84	[MAMSTR]
FLJ36073AAAF	258	28.91	[THOP1]
FLJ36103AAAN	669	75.61	[FANCM]
FLJ36105AAAF	138	15.36	[TMEM75]
FLJ36113AAAF	573	64.58	[MROH8]
FLJ36114AAAF	666	74.34	[ACSBG2]
FLJ36116AAAF	169	18.89	[LINC01356]
FLJ36117AAAN	389	44.18	[PRKG1]
FLJ36119AAAF	404	45.46	[TTLL10]

FLJ36125AAAN	563	64.48	[NOSTRIN]
FLJ36126AAAF	475	53.43	[ASZ1]
FLJ36129AAAF	406	46.64	[SEC14L4]
FLJ36130AAAF	422	47.06	[FAM71D]
FLJ36132AAAN	293	32.02	[ANKLE2]
FLJ36137AAAF	400	43.45	[TFG]
FLJ36139AAAF	608	66.57	[DNAAF3]
FLJ36144AAAF	650	79.07	[GOLGA6L2]
FLJ36144AAAN	650	79.07	[GOLGA6L2]
FLJ36147AAAF	409	45.77	[EXOC3L2]
FLJ36151AAAN	443	51.1	[KNTC1]
FLJ36156AAAF	852	96.53	[PIWIL4]
FLJ36160AAAF	530	59.53	[ANKRD53]
FLJ36161AAAF	439	49.19	[RNF38]
FLJ36166AAAF	318	36.38	[DPY19L2P2]
FLJ36169AAAF	159	17.49	[HSPB9]
FLJ36171AAAF	816	94.11	[TPCN1]
FLJ36172AAAF	439	51.47	[TTLL9]
FLJ36180AAAF	468	53	[TRIML1]
FLJ36186AAAF	137	13.25	[SPANXA2-OT1]
FLJ36198AAAN	158	17.94	[OOSP2]
FLJ36208AAAF	123	12.63	[NHLRC4]
FLJ36211AAAF	269	30.17	[TCF7]
FLJ36241AAAF	399	43.97	[INPP1]
FLJ36252AAAF	356	38.5	[SCML4]
FLJ36258AAAF	461	51.93	[MKL2]
FLJ36290AAAF	251	27.2	[WDR90]
FLJ36310AAAN	491	56.27	[TMEM63B]
FLJ36315AAAF	249	28.68	[RPS6]
FLJ36350AAAF	540	62.99	[ZNF675]
FLJ36374AAAN	552	62.15	[XPNPEP1]
FLJ36386AAAF	282	32.6	[LZTFL1]
FLJ36387AAAN	734	81.5	[ADCY7]
FLJ36400AAAF	663	75.47	[FERMT3]
FLJ36401AAAF	567	60.13	[KRT5]
FLJ36409AAAF	470	56.12	[UPF3B]
FLJ36429AAAF	229	25.74	[METRNL]
FLJ36437AAAF	697	76.58	[EEF1D]
FLJ36441AAAF	273	30.55	[FBXL12]
FLJ36465AAAF	401	45.19	[SERINC2]
FLJ36493AAAF	218	24.6	[METTL21A]
FLJ36507AAAF	601	63.96	[C1orf116]
FLJ36511AAAN	505	57.66	[CFAP206]
FLJ36528AAAF	1064	114	[PUM2]
FLJ36557AAAF	490	55.26	[TC2N]
FLJ36569AAAF	251	28.42	[MSI2]
FLJ36576AAAF	125	14.16	[NKAPP1]
FLJ36583AAAF	938	106.87	[OSBPL6]

FLJ36587AAAF	440	50.8	[SNX31]
FLJ36601AAAF	502	58.06	[CFAP47]
FLJ36613AAAF	461	51.52	[SLC46A3]
FLJ36655AAAF	420	48.47	[TADA2B]
FLJ36666AAAF	161	17.21	[C19orf25]
FLJ36674AAAF	152	17.64	[CDRT4]
FLJ36687AAAF	453	48.44	[UQCRC2]
FLJ36688AAAF	267	30.31	[DPH6]
FLJ36701AAAF	145	15.61	[LINC00304]
FLJ36741AAAF	406	44.02	[PELI3]
FLJ36753AAAF	378	43.98	[ZNF684]
FLJ36754AAAF	155	18.18	[SREK1IP1]
FLJ36765AAAF	543	61.1	[PTGES3L-AARSD1]
FLJ36766AAAF	280	30.39	[FAM131C]
FLJ36778AAAF	105	12.07	[LINC00523]
FLJ36779AAAF	203	22.15	[C9orf163]
FLJ36783AAAF	637	70.53	[KLC4]
FLJ36793AAAF	180	21.02	[STMN3]
FLJ36797AAAF	234	25.16	[LINC00602]
FLJ36811AAAF	445	50.86	[MGAT1]
FLJ36814AAAF	291	32.41	[SLC25A30]
FLJ36827AAAF	488	55.35	[ZNF345]
FLJ36843AAAF	260	29.85	[DNAJC9]
FLJ36870AAAF	331	38.26	[ZNF660]
FLJ36872AAAF	201	22.25	[C8orf34]
FLJ36876AAAF	892	96.31	[MAP3K12]
FLJ36878AAAF	508	54.23	[TMEM102]
FLJ36880AAAF	224	24.84	[FAHD1]
FLJ36883AAAF	616	70.48	[ZNF135]
FLJ36888AAAF	422	44.75	[C19orf47]
FLJ36890AAAF	417	45.06	[SAMD14]
FLJ36896AAAF	510	57.26	[FBXO15]
FLJ36904AAAF	421	46.34	[ACOT4]
FLJ36929AAAF	213	24.2	[NICN1]
FLJ36935AAAF	289	32.1	[SLC25A43]
FLJ36936AAAF	357	40.26	[HTR5A]
FLJ36948AAAF	214	24.15	[NPM2]
FLJ36982AAAF	351	37.34	[PCBP3]
FLJ36991AAAF	499	57.83	[ZNF565]
FLJ36997AAAF	211	24.33	[MAD2L2]
FLJ37000AAAF	136	15.09	[GATC]
FLJ37016AAAF	123	14.24	[PAIP2B]
FLJ37080AAAF	384	43.09	[AMICA1]
FLJ37081AAAF	100	12.32	[TCEAL7]
FLJ37092AAAF	374	42.5	[FADS6]
FLJ37101AAAF	287	32.85	[MTIF3]
FLJ37118AAAF	656	69.76	[C1orf127]
FLJ37126AAAF	92	10.96	[CNPY1]

FLJ37131AAAF	132	14.53	[C8orf31]
FLJ37133AAAF	353	40.02	[VN1R1]
FLJ37155AAAF	193	22.01	[RHOC]
FLJ37170AAAF	439	49.12	[SPOCK1]
FLJ37182AAAF	141	15.24	[LYPD1]
FLJ37185AAAF	171	19.58	[PPP5D1]
FLJ37194AAAN	340	38.19	[NME7]
FLJ37195AAAF	362	40.7	[CYHR1]
FLJ37254AAAF	185	20.91	[NSG1]
FLJ37274AAAF	545	60.05	[KLC2]
FLJ37297AAAF	422	47.57	[SYT1]
FLJ37305AAAF	523	60.38	[MAGEE2]
FLJ37306AAAF	207	22.27	[HECTD2]
FLJ37313AAAF	332	37.86	[DPF1]
FLJ37345AAAF	758	87.29	[TMC7]
FLJ37346AAAF	593	65.26	[GATAD2B]
FLJ37357AAAF	408	46.77	[DNAH6]
FLJ37362AAAF	655	74.76	[CLASRP]
FLJ37370AAAF	422	45.21	[IL11RA]
FLJ37378AAAN	638	77.87	[RBM25]
FLJ37382AAAF	859	93.23	[ZNF281]
FLJ37393AAAF	664	74.47	[ZNF710]
FLJ37401AAAF	558	61.68	[KCNC2]
FLJ37402AAAF	128	13.3	[LINC01561]
FLJ37415AAAF	184	20.51	[LRRC20]
FLJ37416AAAF	561	61.46	[SLC11A2]
FLJ37419AAAF	547	59.56	[CLIP3]
FLJ37421AAAF	163	19.22	[EFCAB11]
FLJ37424AAAF	341	37.46	[LOC100505502]
FLJ37430AAAF	503	57.65	[NMD3]
FLJ37435AAAF	531	58.83	[ABLIM2]
FLJ37440AAAF	439	47.41	[FBLN7]
FLJ37444AAAF	522	60.07	[ZNF454]
FLJ37451AAAF	314	36.14	[GDPD1]
FLJ37456AAAF	378	42.94	[PSMD13]
FLJ37469AAAF	472	51.46	[SLC38A5]
FLJ37473AAAF	477	53.89	[PRDM11]
FLJ37476WAAF	99	11.26	[BID]
FLJ37511AAAF	263	29.68	[NME9]
FLJ37532AAAF	267	30.74	[C11orf70]
FLJ37543AAAF	130	14.82	[C5orf64]
FLJ37562AAAF	188	20.16	[C5orf24]
FLJ37572AAAF	239	26.74	[LRRC57]
FLJ37598AAAN	676	77.28	[SV2C]
FLJ37604AAAF	238	26.3	[DRAM1]
FLJ37616AAAF	985	110.86	[TMEM67]
FLJ37618AAAF	404	45.79	[KIAA0930]
FLJ37642AAAF	399	44.76	[CEP44]

FLJ37652AAAF	423	46.92	[GPT2]
FLJ37659AAAF	248	28.94	[FAM133A]
FLJ37670AAAF	714	81.77	[ZNF585B]
FLJ37681AAAF	291	32.41	[PHYHD1]
FLJ37684AAAF	369	40.91	[PALM2]
FLJ37685AAAF	1073	121.32	[KIAA1377]
FLJ37702AAAF	256	29.45	[ENKUR]
FLJ37712AAAF	490	54.76	[SLC35F3]
FLJ37740AAAF	343	40.34	[PSMD6]
FLJ37753AAAF	354	40.25	[GRAMD2]
FLJ37767AAAF	917	102.52	[HKDC1]
FLJ37770AAAF	210	24.21	[LOC100506127]
FLJ37794AAAF	903	100.32	[IGSF22]
FLJ37817AAAF	489	55.53	[RASGEF1A]
FLJ37818AAAF	221	23.42	[KCTD18]
FLJ37824AAAF	351	39.06	[PAQR6]
FLJ37839AAAF	359	40.15	[RBM4B]
FLJ37856AAAF	134	14.4	[TMEM100]
FLJ37872AAAF	301	32.67	[POU6F1]
FLJ37873AAAF	506	59.26	[COLGALT2]
FLJ37874AAAF	389	43.02	[ANKRD42]
FLJ37882AAAF	376	44.03	[DYX1C1]
FLJ37896AAAF	440	50.42	[SETD4]
FLJ37898AAAF	745	86.98	[CUL2]
FLJ37913AAAF	949	105	[IQSEC2]
FLJ37915AAAF	867	96.82	[ARMC2]
FLJ37916AAAF	228	25.25	[ANKRD46]
FLJ37921AAAF	271	30.83	[MTRF1L]
FLJ37927AAAF	515	56.86	[CDC20B]
FLJ37939AAAF	327	34.98	[ZNF185]
FLJ37943AAAF	302	33.37	[STX17]
FLJ37964AAAF	247	27.66	[THEM5]
FLJ37965AAAF	308	36.04	[LCLAT1]
FLJ37980AAAF	410	44.64	[NIPAL1]
FLJ37984AAAF	484	54.19	[THNSL2]
FLJ37993AAAF	895	99.35	[WDR36]
FLJ37995AAAF	262	29.44	[CA13]
FLJ38002AAAF	529	61.26	[ZNF572]
FLJ38005AAAF	387	43.21	[WDR89]
FLJ38015AAAF	223	26.05	[GINS4]
FLJ38020AAAF	616	68.65	[AIM1L]
FLJ38023AAAF	426	47.6	[KRT17]
FLJ38024AAAF	1043	117.63	[FBXO18]
FLJ38032AAAF	670	76.85	[ZNF233]
FLJ38050AAAF	804	89.23	[SMYD4]
FLJ38059AAAF	597	67.64	[GAN]
FLJ38065AAAF	712	79.9	[PDE4C]
FLJ38066AAAF	527	59.78	[LOC101060376]

FLJ38069AAAF	711	81.07	[MRE11A]
FLJ38072AAAN	1023	113.18	[PPARGC1B]
FLJ38078AAAF	287	31.93	[GPC1]
FLJ38080AAAF	235	25.99	[MPZL3]
FLJ38086AAAF	505	56.87	[KLHL42]
FLJ38098AAAF	470	53.36	[DCSTAMP]
FLJ38104AAAF	283	32	[FAM78A]
FLJ38116AAAF	305	33.18	[LRRC25]
FLJ38121AAAF	482	55.04	[ERVK13-1]
FLJ38141AAAF	1004	113.85	[NLRP12]
FLJ38144AAAF	595	68.33	[ZNF699]
FLJ38149AAAF	312	34.72	[OR7D2]
FLJ38152AAAF	217	25.46	[MOB3A]
FLJ38154AAAF	436	47.46	[SLC17A9]
FLJ38158AAAF	325	37.5	[C12orf29]
FLJ38159AAAF	374	43.81	[CCDC89]
FLJ38171AAAF	85	9.12	[UFM1]
FLJ38177AAAF	734	80.47	[PCDHB18P]
FLJ38180AAAF	342	36.88	[SIRPB2]
FLJ38183AAAF	197	21.11	[INO80C]
FLJ38187AAAF	340	37.35	[CLYBL]
FLJ38193AAAF	777	85.91	[PALLD]
FLJ38194AAAF	555	59.95	[DAB1]
FLJ38198AAAF	652	72.77	[AKNAD1]
FLJ38200AAAF	920	98.92	[GRIP1]
FLJ38204AAAF	224	25.77	[ALKBH8]
FLJ38208AAAF	327	38	[CLVS2]
FLJ38214AAAF	351	40.57	[NECAB1]
FLJ38234AAAF	315	35.54	[CACNG3]
FLJ38237AAAF	215	23.43	[MRPL43]
FLJ38241AAAF	291	32.33	[RALYL]
FLJ38242AAAF	387	42.97	[GTPBP10]
FLJ38250AAAF	562	59.68	[FAM222B]
FLJ38255AAAF	350	37.04	[EIF3F]
FLJ38256AAAF	444	51.54	[ZNF506]
FLJ38273AAAF	360	39.5	[LSM11]
FLJ38281AAAF	641	74.65	[ZNF709]
FLJ38291AAAF	370	41.16	[LRTM2]
FLJ38301AAAF	386	43.99	[EPT1]
FLJ38313AAAF	839	93.29	[ABTB2]
FLJ38324AAAF	683	77.18	[ZYG11B]
FLJ38331AAAF	325	35.74	[TMED8]
FLJ38335AAAF	763	87.62	[ANKFN1]
FLJ38338AAAF	328	36.22	[GPM6B]
FLJ38344AAAF	952	104.99	[CCDC129]
FLJ38349AAAF	291	33.05	[STARD10]
FLJ38361AAAF	354	37.33	[OTX1]
FLJ38362AAAF	779	88.56	[DHX40]

FLJ38374AAAN	154	17.19	[FAM182A]
FLJ38375AAAF	819	94.36	[ZNF658B]
FLJ38376AAAF	189	21.9	[PRELID2]
FLJ38377AAAF	861	90.45	[AMER3]
FLJ38386AAAF	443	50.02	[ZNF250]
FLJ38394AAAF	476	53.07	[CAMK1G]
FLJ38401AAAF	557	64.72	[ZNF195]
FLJ38405AAAF	458	52.8	[OLFM3]
FLJ38415AAAF	515	55.98	[ZNF384]
FLJ38426AAAF	330	37.22	[FAM98B]
FLJ38427AAAF	353	39.82	[B3GNT4]
FLJ38430AAAF	582	64.14	[ARNTL]
FLJ38431AAAN	237	27.69	[RER1]
FLJ38440AAAF	554	63.11	[ZNF426]
FLJ38476AAAF	559	60.87	[YTHDF1]
FLJ38482AAAF	267	30.49	[TMEM192]
FLJ38483AAAN	265	27.67	[MAPT]
FLJ38499AAAF	484	56.01	[PAPD4]
FLJ38505AAAF	231	24.5	[LURAP1L]
FLJ38517AAAF	383	43.07	[PSMC5]
FLJ38524AAAF	348	40.32	[ZNF883]
FLJ38535AAAF	282	30.08	[PIANP]
FLJ38547AAAF	1124	125.26	[GPR125]
FLJ38554AAAF	372	41.97	[B4GALT2]
FLJ38562AAAF	343	38.13	[APOL6]
FLJ38594AAAF	160	18.05	[C14orf180]
FLJ38596AAAF	161	17.04	[C15orf56]
FLJ38600AAAF	595	65.02	[SIGLEC12]
FLJ38602AAAF	457	50.21	[ADSSL1]
FLJ38607AAAF	189	20.18	[DAND5]
FLJ38608AAAF	164	17.87	[LSMEM2]
FLJ38615AAAF	199	22.67	[C15orf26]
FLJ38625AAAF	639	70.52	[INPP5J]
FLJ38629AAAF	344	39.64	[ZSCAN26]
FLJ38632AAAF	312	34.35	[AIMP1]
FLJ38637AAAF	626	72.19	[ZNF441]
FLJ38643AAAF	201	22.58	[ARFRP1]
FLJ38644AAAF	146	15.2	[DAZAP2]
FLJ38646AAAF	420	48.31	[CDCA7L]
FLJ38656AAAF	855	96.74	[DDR2]
FLJ38663AAAF	166	18.83	[C12orf65]
FLJ38665AAAF	709	80.18	[ARHGEF16]
FLJ38673AAAF	769	84.78	[ITGB2]
FLJ38674AAAF	359	41.67	[ZNF586]
FLJ38679AAAF	523	58.66	[EARS2]
FLJ38680AAAF	599	63.52	[SLC34A3]
FLJ38681AAAF	221	24.21	[PRR26]
FLJ38683AAAF	462	51.43	[HEPACAM2]

FLJ38692AAAF	504	53.19	[DMRTA1]
FLJ38700AAAF	667	73.28	[PANX2]
FLJ38702AAAF	721	81.79	[GYLTL1B]
FLJ38705AAAF	198	22.81	[ZFP41]
FLJ38706AAAF	148	16.35	[ZNF7]
FLJ38708AAAF	350	40.43	[ATP6V0D2]
FLJ38716AAAF	362	42.33	[MAB21L3]
FLJ38720AAAF	577	64.22	[ACSM2A]
FLJ38725AAAF	430	47.82	[LACC1]
FLJ38744AAAF	224	23.21	[CLDN19]
FLJ38755AAAF	437	47.87	[PNPLA1]
FLJ38769AAAF	632	68.14	[SLC34A1]
FLJ38781AAAF	621	67.98	[HSPA8]
FLJ38792AAAF	129	13.99	[TMEM105]
FLJ38800AAAF	285	32.7	[PPP1R3B]
FLJ38819AAAF	460	51.19	[ANKS4B]
FLJ38822AAAF	638	72.51	[GBP7]
FLJ38831AAAF	685	76	[BMPER]
FLJ38833AAAN	435	49.5	[SUGP1]
FLJ38841AAAF	232	26.39	[RNF212]
FLJ38854AAAF	213	23.76	[FAM177A1]
FLJ38865AAAF	475	52.82	[ZCCHC5]
FLJ38866AAAF	506	56.5	[CDYL2]
FLJ38872AAAN	579	66.84	[UPF2]
FLJ38874AAAF	475	51.6	[CAP1]
FLJ38876AAAF	824	94.2	[FILIP1L]
FLJ38891AAAF	419	47.53	[BCS1L]
FLJ38898AAAF	175	19.73	[THAP3]
FLJ38903AAAF	243	27.67	[HOXB8]
FLJ38913AAAF	696	79.25	[SESTD1]
FLJ38924AAAF	615	67.72	[RPN2]
FLJ38927AAAF	467	52.4	[ENAH]
FLJ38934AAAF	802	87.17	[NPAS4]
FLJ38944AAAF	161	16.99	[EID2B]
FLJ38961AAAF	731	80.64	[GSN]
FLJ38962AAAF	579	62.83	[GPC2]
FLJ38964AAAF	340	37.14	[REM2]
FLJ38965AAAF	308	35.3	[LFNG]
FLJ38968AAAN	260	29.34	[ARL14EP]
FLJ38973AAAF	385	43.45	[C2orf69]
FLJ38977AAAF	429	48.73	[TNIP2]
FLJ38981AAAN	492	57.23	[SESN3]
FLJ38983AAAF	660	76.33	[ANO10]
FLJ38984AAAF	229	24.97	[C1orf216]
FLJ38985AAAF	458	52.8	[OLFM1]
FLJ38986AAAF	360	39.19	[NIPA2]
FLJ38989AAAF	350	40.63	[TMEM185A]
FLJ38991AAAF	333	37.03	[COX18]

FLJ38993AAAF	160	18.98	[CNIH3]
FLJ39002AAAF	304	33.43	[SGTB]
FLJ39006AAAF	521	58.66	[TIGD6]
FLJ39009AAAF	167	19.05	[TCAP]
FLJ39024AAAF	471	52.05	[CPSF7]
FLJ39025AAAF	282	30.76	[ENDOV]
FLJ39026AAAF	1133	128.91	[TRAPPC11]
FLJ39027AAAF	812	89.1	[CHAMP1]
FLJ39031AAAF	168	18.56	[FAM219A]
FLJ39034AAAN	1314	148.36	[RNF123]
FLJ39035AAAF	229	25.42	[CYB561D1]
FLJ39039AAAN	950	108.47	[OSBPL1A]
FLJ39043AAAF	272	29.36	[OLIG3]
FLJ39044AAAF	474	53.25	[CORO1C]
FLJ39045AAAF	582	64.38	[FAM124A]
FLJ39058AAAF	122	13.69	[C11orf44]
FLJ39060AAAF	123	13.9	[LOC401589]
FLJ39078AAAF	836	89.89	[ZNRF3]
FLJ39091AAAF	351	38.5	[PTGR2]
FLJ39100AAAF	520	56.82	[KRT78]
FLJ39103AAAN	483	52.99	[LAMA3]
FLJ39106AAAF	210	22.53	[DYNAP]
FLJ39108AAAF	272	30.21	[TPRG1L]
FLJ39110AAAF	539	60.21	[CLEC4F]
FLJ39116AAAF	545	60.02	[OIT3]
FLJ39120AAAF	445	49.33	[GSDMA]
FLJ39122AAAF	329	36.34	[ASB5]
FLJ39140AAAF	668	76.11	[BEST3]
FLJ39141AAAF	725	81.8	[EXOC8]
FLJ39145AAAF	299	32.54	[SPRY4]
FLJ39152AAAF	439	47.83	[BRF1]
FLJ39161AAAF	409	46.84	[FAXC]
FLJ39163AAAN	457	50.89	[LRP8]
FLJ39174AAAF	853	96.55	[C1orf112]
FLJ39176AAAF	203	22.36	[RNF152]
FLJ39183AAAF	253	28.16	[TP53RK]
FLJ39196AAAF	193	22.25	[NCALD]
FLJ39223AAAF	541	61.73	[ZNF329]
FLJ39237AAAF	369	40.46	[NAT16]
FLJ39240AAAF	516	59.46	[ZNF480]
FLJ39249AAAF	244	27.46	[ARL10]
FLJ39252AAAF	498	58.37	[ZNF682]
FLJ39259AAAF	589	66.13	[ENC1]
FLJ39266AAAF	878	98.18	[INO80D]
FLJ39269AAAF	690	77.37	[GUCY1A3]
FLJ39274AAAF	437	50.95	[ZNF491]
FLJ39276AAAF	339	38.74	[GNAI2]
FLJ39279AAAF	773	86.29	[CMIP]

FLJ39292AAAF	643	70.97	[PJA1]
FLJ39294AAAF	716	79.21	[KIAA1958]
FLJ39307AAAF	895	100.4	[LONP1]
FLJ39309AAAF	461	52.8	[THRB]
FLJ39317AAAF	217	24.83	[PID1]
FLJ39320AAAF	566	59.51	[INSM2]
FLJ39321WAAN	575	66.37	[KBTBD8]
FLJ39331AAAF	345	39.19	[TMEM231]
FLJ39335AAAF	531	58.28	[RCBTB1]
FLJ39370AAAF	132	14.72	[C4orf32]
FLJ39373AAAF	558	62.35	[ARMCX5]
FLJ39378AAAF	362	42.24	[RILPL1]
FLJ39383AAAF	274	29.4	[BATF2]
FLJ39384AAAN	732	81.86	[PRMT9]
FLJ39421AAAF	264	28.33	[LINC00482]
FLJ39426AAAF	163	19.37	[HMGN2P46]
FLJ39430AAAF	166	19.17	[CCDC12]
FLJ39436AAAF	706	77.21	[ZNF341]
FLJ39451AAAF	502	57	[EPHX2]
FLJ39454AAAF	233	24.47	[VWA1]
FLJ39458AAAF	201	22.88	[SAMD12]
FLJ39460AAAF	699	74.88	[TLE3]
FLJ39501AAAF	531	61.9	[CYP4F22]
FLJ39504AAAF	307	35.77	[HS3ST1]
FLJ39509AAAF	164	18.96	[ZNF321P]
FLJ39511AAAF	271	29.63	[ZGLP1]
FLJ39512AAAF	599	68.85	[ZNF530]
FLJ39513AAAF	303	33.7	[RNF144B]
FLJ39514AAAF	639	69.99	[SCFD2]
FLJ39516AAAF	101	11.35	[VMA21]
FLJ39521AAAF	591	66.48	[RGS3]
FLJ39526AAAF	283	31.86	[CA12]
FLJ39531AAAF	183	21.04	[C15orf54]
FLJ39540AAAF	527	62.03	[SWAP70]
FLJ39548AAAF	663	73.18	[ANKRD6]
FLJ39553AAAF	374	39.91	[ERICH5]
FLJ39559AAAF	317	33.43	[VGLL2]
FLJ39565AAAF	239	26.85	[MSS51]
FLJ39573AAAF	335	38.79	[LRRC39]
FLJ39575AAAF	131	14.46	[LSMEM1]
FLJ39576AAAF	208	23.48	[YIPF7]
FLJ39577AAAF	318	34.98	[ASB12]
FLJ39592AAAF	599	69.16	[ZNF343]
FLJ39603AAAF	203	22.7	[WIBG]
FLJ39611AAAF	352	38.46	[RAD51AP1]
FLJ39614AAAF	479	51.03	[ZNF513]
FLJ39616AAAF	139	13.17	[MAPKAPK5-AS1]
FLJ39630AAAF	197	22.62	[NKAIN3]

FLJ39647AAAF	159	17.92	[C17orf78]
FLJ39651AAAF	485	54.7	[PDCD7]
FLJ39680AAAF	371	40.8	[NDRG2]
FLJ39681AAAF	640	72.12	[MTMR11]
FLJ39683AAAF	538	57.61	[NAPRT]
FLJ39690AAAF	513	56.58	[PLTP]
FLJ39699AAAF	300	33.02	[ARMCX6]
FLJ39703AAAN	145	15.3	[FDX1L]
FLJ39708AAAN	275	32.08	[UPF3A]
FLJ39711AAAN	807	89.13	[NLRC5]
FLJ39722AAAF	407	46.2	[ZNF552]
FLJ39727AAAF	567	63.56	[ECM1]
FLJ39729AAAF	410	46.5	[CLIC5]
FLJ39730AAAF	247	29.05	[GBAS]
FLJ39735AAAN	152	16.64	[LRRC75B]
FLJ39743AAAF	192	21.43	[FAM169B]
FLJ39752AAAN	595	65.13	[IGHM]
FLJ39759AAAF	666	72.49	[DDX51]
FLJ39767AAAF	919	99.82	[ANKRD44]
FLJ39769AAAF	493	54.43	[TMCO6]
FLJ39771AAAF	731	80.7	[TAGAP]
FLJ39776AAAF	280	32.02	[CCDC106]
FLJ39785AAAF	333	37.61	[CDK3]
FLJ39796AAAF	744	85.11	[ZNF366]
FLJ39802AAAF	620	69.11	[FAM129C]
FLJ39806AAAF	621	70.33	[KLHL6]
FLJ39813AAAF	98	11.47	[S100A13]
FLJ39821AAAF	145	16.26	[BCORP1]
FLJ39822AAAF	384	42.45	[SLC38A11]
FLJ39827AAAF	804	87.87	[AMER1]
FLJ39829AAAF	522	58.83	[MB21D1]
FLJ39837AAAF	585	67.2	[ZNF614]
FLJ39850AAAF	436	50.31	[ZNF101]
FLJ39860AAAF	549	62.52	[FRMD1]
FLJ39873AAAF	244	26.29	[TIGIT]
FLJ39911AAAF	180	20.19	[FGD2]
FLJ39929AAAF	590	68.5	[C7orf31]
FLJ39963AAAF	430	50.17	[ZNF713]
FLJ39968AAAF	435	48.26	[HYAL1]
FLJ39989AAAF	644	72.01	[TMEM214]
FLJ39996AAAF	424	44.47	[SF3B4]
FLJ40006AAAF	167	18.19	[C1orf200]
FLJ40036AAAF	516	56.92	[IGHG3]
FLJ40037AAAN	473	51.9	[IGHG1]
FLJ40053AAAF	626	70.35	[RNPEP]
FLJ40056AAAF	1106	121.49	[SSFA2]
FLJ40059AAAF	289	30.78	[ODF3L2]
FLJ40071AAAF	394	45.2	[HORMAD1]

FLJ40075AAAF	363	42.89	[C20orf96]
FLJ40083AAAF	611	71.09	[CCDC37]
FLJ40089AAAF	563	65.29	[CCDC38]
FLJ40097AAAF	708	81	[TRIM42]
FLJ40098AAAF	410	45.9	[TSPYL6]
FLJ40099AAAF	530	60.1	[LRRC27]
FLJ40105AAAF	535	60.71	[RAB11FIP4]
FLJ40108AAAF	374	40.84	[PBX4]
FLJ40112AAAF	637	66.17	[C19orf57]
FLJ40114AAAF	691	76.11	[ACRC]
FLJ40120AAAF	370	39.44	[ETV2]
FLJ40121AAAF	570	63.09	[C17orf47]
FLJ40125AAAF	340	37.18	[PPM1N]
FLJ40132AAAF	569	58.97	[AGFG1]
FLJ40137AAAF	330	36.91	[CNTD1]
FLJ40141AAAF	242	26.01	[C3orf56]
FLJ40142AAAF	135	14.96	[C12orf76]
FLJ40154AAAF	338	35.38	[SLC35G3]
FLJ40157AAAF	584	66.73	[PDILT]
FLJ40158AAAF	425	48.03	[UBR7]
FLJ40160AAAF	389	42.61	[KLF17]
FLJ40161AAAF	222	23.1	[TRIM14]
FLJ40168AAAF	136	15.16	[TMEM78]
FLJ40172AAAF	177	19.92	[C2orf61]
FLJ40182AAAF	226	26.14	[FAM9B]
FLJ40184AAAF	764	83.94	[OSBPL10]
FLJ40198AAAF	621	77.13	[GOLGA6L1]
FLJ40199AAAF	179	20.24	[TEX35]
FLJ40201AAAF	414	47.3	[C1orf177]
FLJ40206AAAF	658	74.2	[WDR93]
FLJ40215AAAF	307	34.7	[C11orf63]
FLJ40217AAAF	295	31.2	[SLC25A35]
FLJ40218AAAF	735	83.93	[FBXL13]
FLJ40223AAAF	406	45.56	[ARRB2]
FLJ40224AAAF	241	25.77	[KIRREL3-AS3]
FLJ40233AAAF	773	87.4	[PASD1]
FLJ40235AAAF	197	21.28	[SIGLECL1]
FLJ40240AAAF	520	60.12	[PNLDC1]
FLJ40242AAAF	407	44.09	[MAGEB6]
FLJ40244AAAF	210	24.07	[TBC1D28]
FLJ40246AAAF	389	42.83	[C9orf173]
FLJ40261AAAF	381	42.98	[PRKAR1A]
FLJ40268AAAF	110	12.47	[C10orf82]
FLJ40270AAAF	371	41.25	[PAPD5]
FLJ40273AAAF	364	40.31	[RBM4]
FLJ40280AAAF	309	35.59	[PPP2CA]
FLJ40283AAAF	468	51.43	[BEND7]
FLJ40287AAAF	340	38.53	[PRKAG1]

FLJ40288AAAF	150	16.74	[MCF2L]
FLJ40289AAAF	543	61.51	[ZNF280B]
FLJ40290AAAF	348	39.39	[SLFNL1]
FLJ40296AAAN	221	23.26	[PRR20A]
FLJ40297AAAF	304	34.52	[SH3GL1]
FLJ40316AAAF	244	28.17	[SSMEM1]
FLJ40319AAAF	141	15.98	[LINC00469]
FLJ40337AAAF	553	60.39	[TXNDC2]
FLJ40338AAAF	332	35.97	[HNRNPAB]
FLJ40343AAAF	128	14.48	[LINC00303]
FLJ40344AAAN	682	77.51	[CCDC62]
FLJ40348AAAF	406	43.7	[IGSF11]
FLJ40365AAAF	499	56.91	[CCDC105]
FLJ40366AAAF	104	11.6	[ZNF561-AS1]
FLJ40370AAAF	776	86.66	[ANKEF1]
FLJ40371AAAF	166	18.5	[CFL1]
FLJ40391AAAF	551	61.89	[CDC25C]
FLJ40404AAAF	149	17.55	[PLEKHJ1]
FLJ40405AAAF	290	30.42	[ARIH2OS]
FLJ40419AAAF	317	35.39	[RFPL3]
FLJ40422AAAF	127	14.68	[LINC00521]
FLJ40430AAAF	158	16.56	[MRGPRG-AS1]
FLJ40432AAAF	289	33.43	[CCNYL1]
FLJ40441AAAF	824	95.46	[CAGE1]
FLJ40442AAAF	232	25.72	[SLC25A41]
FLJ40449AAAN	307	33	[POM121L12]
FLJ40478AAAF	582	62.19	[SLCO3A1]
FLJ40488AAAF	811	89.93	[RNF10]
FLJ40496AAAF	268	30.6	[HCCS]
FLJ40511AAAF	486	55.11	[USP30]
FLJ40522AAAF	102	11.86	[C3orf22]
FLJ40523AAAF	534	60.63	[EHD1]
FLJ40542AAAF	170	18.11	[LINC00528]
FLJ40562AAAF	716	78.56	[MPEG1]
FLJ40563AAAF	320	35.51	[SLC25A19]
FLJ40595AAAF	263	29.6	[RPS4X]
FLJ40597AAAF	444	49.5	[PARP15]
FLJ40606AAAF	135	14.18	[LOC643549]
FLJ40611AAAF	427	47.6	[UGDH]
FLJ40623AAAF	220	23.99	[CD8A]
FLJ40626AAAF	318	35.2	[ZFYVE27]
FLJ40634AAAF	182	19.72	[MAFF]
FLJ40680AAAF	211	22.7	[CACTIN-AS1]
FLJ40686AAAF	465	51.37	[CCM2]
FLJ40688AAAF	275	30.95	[HLA-DQB2]
FLJ40705AAAF	629	72.92	[SCAI]
FLJ40709AAAF	299	32.85	[DNASE1L2]
FLJ40710AAAF	315	35.31	[OTUB1]

FLJ40727AAAF	244	26.96	[GYG2]
FLJ40738AAAF	190	21.4	[CYGB]
FLJ40744AAAF	281	31.33	[NRIP2]
FLJ40746AAAF	509	58.88	[CYP4X1]
FLJ40747AAAF	462	52.08	[NUDT12]
FLJ40749AAAF	223	24.82	[RAB37]
FLJ40760AAAF	426	47.9	[DNAJA4]
FLJ40773AAAF	444	50.8	[PLD5]
FLJ40787AAAF	275	31.8	[TMEM45B]
FLJ40798AAAF	666	76.93	[ZNF844]
FLJ40802AAAF	593	66.59	[MARS2]
FLJ40803AAAF	707	77.92	[FCRL3]
FLJ40862AAAF	435	48.99	[ZBTB25]
FLJ40863AAAF	407	43.65	[EGLN2]
FLJ40868AAAF	587	68.24	[C14orf39]
FLJ40873AAAF	179	20.73	[TCTEX1D1]
FLJ40876AAAF	223	25.49	[STYX]
FLJ40877AAAF	171	18.64	[TSEN15]
FLJ40900AAAF	179	19.19	[PTRH2]
FLJ40905AAAF	894	99.31	[COG1]
FLJ40906AAAF	236	25.94	[RSPO1]
FLJ40918AAAF	259	29.79	[TYW3]
FLJ40919AAAF	139	16.36	[FAM216B]
FLJ40927AAAF	686	77.03	[RHPN2]
FLJ40936AAAF	194	21.1	[IL18BP]
FLJ40943AAAF	396	44.83	[CCDC130]
FLJ40976AAAN	429	47.28	[TCTN3]
FLJ40981AAAF	673	77.24	[ZNF433]
FLJ40986AAAF	483	55.91	[ATP6V1H]
FLJ40999AAAN	278	31.87	[FAM76A]
FLJ41008AAAF	735	79.38	[TRAPPC12]
FLJ41012AAAF	600	69.03	[PDS5A]
FLJ41024AAAF	99	10.89	[NCBP2-AS2]
FLJ41026AAAF	622	70.33	[LRRC70]
FLJ41027AAAF	243	28.3	[RSPO2]
FLJ41028AAAF	766	88.45	[BRINP3]
FLJ41037AAAF	258	28.41	[AMN1]
FLJ41040AAAF	690	75.39	[SLC27A5]
FLJ41042AAAF	458	50.76	[FBXW9]
FLJ41043AAAF	1284	141.44	[FRMPD2]
FLJ41044AAAF	971	106.75	[SCUBE2]
FLJ41048AAAF	351	39.04	[LPAR2]
FLJ41055AAAF	355	41.12	[CCR3]
FLJ41065AAAF	369	41.17	[CCDC14]
FLJ41070AAAF	239	27.45	[SPAG7]
FLJ41090AAAF	157	18.61	[FBXO36]
FLJ41113AAAF	842	93.95	[AFAP1L2]
FLJ41190AAAF	160	18.34	[CPLX4]

FLJ41214AAAF	163	18.26	[NMNAT3]
FLJ41238AAAF	275	31.23	[TPRG1]
FLJ41329AAAF	215	22.99	[LOC100128429]
FLJ41342AAAF	678	76.52	[BRINP2]
FLJ41353AAAF	473	52.44	[KCTD8]
FLJ41358AAAF	433	49.04	[PRMT2]
FLJ41359AAAF	195	20.89	[PRPF40B]
FLJ41361AAAF	408	44.9	[C1orf94]
FLJ41388AAAF	347	36.94	[RGS14]
FLJ41395AAAF	614	68.41	[ANKRD55]
FLJ41423AAAF	167	17.96	[LOC399886]
FLJ41434AAAF	492	55.06	[PLXNA4]
FLJ41449AAAF	125	13.59	[FAM19A5]
FLJ41463AAAF	474	53.56	[SAAL1]
FLJ41485AAAF	132	14.29	[CENPM]
FLJ41502AAAF	656	72.04	[ANKS3]
FLJ41550AAAF	215	23.91	[TLDC2]
FLJ41559AAAN	710	79.06	[BBS12]
FLJ41567AAAF	236	25.37	[NUDT8]
FLJ41586AAAF	131	14.62	[FAM19A2]
FLJ41587AAAF	275	31.29	[ACER2]
FLJ41606SAAN	1069	121.34	[PAXIP1]
FLJ41610AAAF	1139	122.32	[MAML3]
FLJ41640AAAN	340	38.85	[MCHR2]
FLJ41688AAAN	723	83.73	[DPY19L4]
FLJ41692AAAF	735	79.79	[SYDE1]
FLJ41716AAAF	222	24.74	[PLEKHB2]
FLJ41725AAAF	304	34.29	[DCUN1D3]
FLJ41743AAAF	784	86.79	[ABI3BP]
FLJ41766AAAF	109	12.31	[CRYM-AS1]
FLJ41767AAAF	228	26.24	[CENPH]
FLJ41770AAAF	147	15.87	[RMI2]
FLJ41774AAAF	206	24.49	[MRPL40]
FLJ41801AAAF	358	39.01	[STBD1]
FLJ41814AAAF	182	18.37	[LINC00612]
FLJ41823AAAF	176	20.18	[LRRC16A]
FLJ41827AAAF	303	32.06	[SLC25A29]
FLJ41835AAAF	227	25.09	[SH3YL1]
FLJ41838AAAF	838	93.8	[GPRASP2]
FLJ41852AAAF	1111	127.4	[XPO6]
FLJ41855AAAF	147	16.83	[MIR4697HG]
FLJ41859AAAF	163	16.91	[LOC401286]
FLJ41861AAAF	676	69.61	[PERM1]
FLJ41866AAAF	238	26.56	[WDR82]
FLJ41868AAAF	219	24.84	[CALN1]
FLJ41872AAAF	797	91.68	[CASD1]
FLJ41891AAAF	357	42.73	[SH2D4B]
FLJ41914AAAF	250	28.54	[LRRC10]

FLJ41923AAAF	214	24.6	[SMCO1]
FLJ41938AAAN	377	42.69	[PAQR9]
FLJ41944AAAN	538	59.52	[ERVFRD-1]
FLJ41947AAAN	275	30.85	[NCCRP1]
FLJ41961AAAF	576	58.87	[NUPL1]
FLJ41962AAAF	148	17.04	[MAVS]
FLJ41993AAAF	336	37.65	[IL17REL]
FLJ42063AAAN	327	37.43	[IL10RB]
FLJ42081AAAF	396	45.81	[ACTR6]
FLJ42089AAAF	440	50.62	[SOCS4]
FLJ42098AAAF	407	46.99	[METTL8]
FLJ42102AAAF	165	18.17	[XIRP1]
FLJ42113AAAF	527	57.99	[LRRC43]
FLJ42117AAAF	563	62.87	[C3orf67]
FLJ42118AAAF	512	56.88	[SCARB1]
FLJ42119AAAF	444	52.41	[CCDC83]
FLJ42122AAAF	356	38.72	[PRR19]
FLJ42130AAAF	459	51.09	[GRSF1]
FLJ42168AAAF	169	18.35	[VMAC]
FLJ42213AAAF	151	17.01	[LOC100132272]
FLJ42221AAAF	601	68.31	[ESCO2]
FLJ42270AAAF	377	41.14	[MLC1]
FLJ42279AAAF	426	45.08	[HFE2]
FLJ42330AAAF	298	32.25	[LOC389602]
FLJ42340AAAN	507	55.56	[TOM1L2]
FLJ42346AAAF	278	33.06	[CCDC121]
FLJ42348AAAF	396	45.44	[GJC1]
FLJ42407AAAF	196	22.12	[RHOB]
FLJ42447AAAF	162	17.5	[C3orf18]
FLJ42455AAAF	539	58.66	[NUDT18]
FLJ42456AAAF	434	49.45	[CCDC136]
FLJ42459AAAF	361	39.91	[HOMER3]
FLJ42461AAAF	317	34.86	[SMTNL2]
FLJ42486AAAF	197	21.71	[TMEM179]
FLJ42527AAAF	200	22.22	[MED22]
FLJ42528AAAF	102	11.08	[NCMAP]
FLJ42556AAAF	813	92.43	[ABR]
FLJ42568AAAF	1005	108.19	[ESPNL]
FLJ42603AAAF	945	106.02	[DGKZ]
FLJ42625AAAF	776	82.43	[GPRIN3]
FLJ42649AAAF	711	77.93	[RAP1GAP2]
FLJ42654AAAF	132	14.89	[GOLT1A]
FLJ42655AAAF	180	20.26	[PMF1]
FLJ42684AAAF	515	58.43	[TTC8]
FLJ42701AAAN	919	103.87	[ADCY5]
FLJ42706AAAF	367	41	[KCNAB2]
FLJ42731AAAF	518	58.37	[ANKRD13D]
FLJ42743AAAF	171	19.09	[LYPD6]

FLJ42744AAAF	799	88.25	[CDH8]
FLJ42754AAAF	304	32.63	[PPTC7]
FLJ42765AAAF	440	46.48	[GORASP1]
FLJ42783AAAF	841	87.93	[NYAP1]
FLJ42801AAAF	426	48.13	[MAPK10]
FLJ42802AAAF	221	25.48	[SRSF2]
FLJ42811AAAF	183	20.74	[RAP2C]
FLJ42818AAAF	127	14.48	[C2orf68]
FLJ42826AAAF	326	37.07	[PEX10]
FLJ42838AAAF	546	61.96	[C1orf87]
FLJ42874AAAF	337	38.55	[ABHD13]
FLJ42879AAAF	339	37.44	[SH2D5]
FLJ42886AAAF	302	33.92	[NAPB]
FLJ42890AAAF	613	67.94	[PPP1R18]
FLJ42903AAAF	131	14.82	[C2orf91]
FLJ42909AAAF	190	20	[C9orf139]
FLJ42913AAAN	778	82.68	[TNKS1BP1]
FLJ42914AAAN	1430	160.83	[FGD6]
FLJ42925AAAF	388	42.78	[LANCL3]
FLJ42944AAAF	608	68.88	[GALNT11]
FLJ42946AAAF	295	34.55	[FAM47E]
FLJ42957AAAF	143	16.39	[LINC00173]
FLJ42964AAAF	208	23.85	[MRAS]
FLJ42970AAAF	144	16.58	[LOC100130451]
FLJ42972AAAF	151	16.31	[C22orf34]
FLJ43030AAAF	272	28.88	[CD99L2]
FLJ43037AAAF	357	40.26	[FAM118A]
FLJ43058AAAF	340	38.92	[C17orf85]
FLJ43063AAAF	446	48.24	[IRX6]
FLJ43070AAAF	1317	145.66	[PNPLA7]
FLJ43083AAAF	383	42.05	[FAM217B]
FLJ43096AAAF	740	83.53	[DTX3L]
FLJ43107AAAF	662	75.68	[CUX1]
FLJ43118AAAF	165	17.76	[CGB7]
FLJ43121AAAF	1047	110.75	[C15orf39]
FLJ43122AAAF	581	64.39	[LRRC15]
FLJ43129AAAF	293	33.21	[NUS1]
FLJ43132AAAF	272	29.06	[C1orf226]
FLJ43144AAAF	708	80.98	[P3H2]
FLJ43149AAAF	1309	144.23	[SBNO2]
FLJ43151AAAF	409	46.77	[GXYLT1]
FLJ43154AAAF	373	41.61	[SAMSN1]
FLJ43169AAAF	806	93.28	[TMEM63C]
FLJ43178AAAF	341	38.99	[C20orf195]
FLJ43185AAAF	139	14.5	[LOC646034]
FLJ43189AAAF	451	51.33	[GABRA2]
FLJ43198AAAF	517	58.99	[MTUS1]
FLJ43223AAAN	528	59.14	[YARS]

FLJ43232AAAF	493	51.85	[SLC1A5]
FLJ43233AAAF	675	73.45	[IDUA]
FLJ43236AAAF	409	43.17	[RAD23B]
FLJ43237AAAF	228	24.47	[LHFPL2]
FLJ43242AAAF	491	53.53	[BANP]
FLJ43259AAAN	743	83.07	[THNSL1]
FLJ43264AAAF	295	32.57	[GJD3]
FLJ43269AAAF	118	13.29	[COX20]
FLJ43277AAAF	742	81.42	[SLC5A11]
FLJ43287AAAF	468	52.53	[ATG4B]
FLJ43288AAAF	622	67.02	[KIAA1467]
FLJ43309AAAF	252	28.16	[LOC344967]
FLJ43310AAAF	542	62.57	[CSGALNACT2]
FLJ43313AAAF	196	21.73	[CCDC66]
FLJ43317AAAF	243	27.4	[FGF12]
FLJ43328AAAF	590	65.71	[KDM1B]
FLJ43329AAAF	175	18.62	[FOXL2NB]
FLJ43330AAAF	235	25.78	[TMEM215]
FLJ43333AAAF	1309	149.31	[DENND5B]
FLJ43334AAAF	759	82.15	[FIGN]
FLJ43339AAAF	534	57.32	[C15orf52]
FLJ43342AAAF	586	65.68	[CTPS2]
FLJ43343AAAF	128	14.22	[LOC100129223]
FLJ43346AAAF	714	81.6	[FRMD7]
FLJ43347AAAF	393	44.41	[RMND5B]
FLJ43349AAAF	352	39.33	[MKX]
FLJ43350AAAF	292	34.11	[C11orf57]
FLJ43357AAAF	277	29.1	[WDR86]
FLJ43366AAAF	655	73.88	[KLHL13]
FLJ43370AAAF	588	65.77	[ASB15]
FLJ43372AAAF	517	57.86	[CHRNG]
FLJ43382AAAF	881	97.84	[DDHD1]
FLJ43385AAAF	254	28.37	[HACD2]
FLJ43389AAAF	1076	115.1	[ANKRD52]
FLJ43406AAAF	332	36.49	[LDHAL6A]
FLJ43409AAAF	408	43.61	[FOXD4L1]
FLJ43424AAAF	66	7.37	[SERP1]
FLJ43457AAAF	184	20.5	[RHEB]
FLJ43472AAAF	429	48.35	[PYROXD1]
FLJ43475AAAF	1130	125.82	[SORCS1]
FLJ43505AAAF	120	13.8	[FAM177B]
FLJ43529AAAF	452	49.41	[ANKRD33]
FLJ43540AAAF	235	25.99	[FAM3B]
FLJ43547AAAF	548	61.73	[ARHGAP27]
FLJ43571AAAF	167	18.18	[ZG16]
FLJ43577AAAF	452	49.58	[ASB10]
FLJ43578AAAF	398	42.61	[FAM53A]
FLJ43580AAAF	474	55.02	[PRAMEF1]

FLJ43582AAAF	223	24.74	[C8orf86]
FLJ43599AAAF	470	53.68	[PRPH]
FLJ43623AAAF	368	41.64	[TMEM26]
FLJ43636AAAF	331	37.27	[SLAMF6]
FLJ43640AAAF	388	41.99	[PGA3]
FLJ43646AAAF	145	15.56	[C11orf45]
FLJ43647AAAF	805	90.4	[PIK3AP1]
FLJ43653AAAN	1215	139.61	[DOCK11]
FLJ43654AAAF	267	30.14	[C3orf62]
FLJ43659AAAF	153	17.35	[MALL]
FLJ43671AAAF	784	89.83	[TLR2]
FLJ43679AAAF	324	36.07	[NSUN5]
FLJ43692AAAF	484	53.31	[ARHGEF35]
FLJ43733AAAF	929	101.68	[UNC45A]
FLJ43738AAAF	572	66.04	[LOC100132731]
FLJ43752AAAF	198	20.9	[LINC00336]
FLJ43754AAAF	259	30.35	[PCGF1]
FLJ43792AAAF	201	22.92	[GUCA1A]
FLJ43793AAAF	1394	157.99	[LRPPRC]
FLJ43801AAAF	807	91.09	[EPC2]
FLJ43807AAAN	575	66.08	[UBR2]
FLJ43829AAAF	164	19.55	[C17orf105]
FLJ43842AAAF	225	23.87	[DERL3]
FLJ43857AAAF	1445	157.14	[SPATA31E1]
FLJ43859AAAN	917	102.39	[SPATA31D4]
FLJ43860AAAF	1318	149.16	[MROH5]
FLJ43869AAAF	522	53.24	[NUP62]
FLJ43889AAAF	470	53.25	[RBM46]
FLJ43909AAAN	969	105.78	[SEC31A]
FLJ43920AAAF	184	21.8	[DYDC1]
FLJ43933WAAF	131	15.7	[FAR2P1]
FLJ43958AAAF	232	25.69	[DNAJB8]
FLJ43968AAAF	1264	136.52	[UBN2]
FLJ43986AAAF	566	60.71	[GATAD2A]
FLJ43995AAAF	694	79.16	[KIAA0408]
FLJ44003AAAN	266	29.1	[FAM122B]
FLJ44005AAAF	233	25.81	[LINC00083]
FLJ44025AAAF	794	88.39	[MYRIP]
FLJ44026AAAF	1233	136.8	[KIAA1211]
FLJ44029AAAF	323	35.92	[LGALS9]
FLJ44037AAAF	803	91.03	[PTCHD3]
FLJ44038AAAF	520	56.32	[SLC2A14]
FLJ44045AAAF	195	21.84	[TMEM239]
FLJ44046AAAF	1116	126.85	[CATSPERB]
FLJ44050AAAF	829	97.63	[CCDC178]
FLJ44068AAAF	539	58.5	[NELFA]
FLJ44083AAAF	266	29.16	[REC114]
FLJ44108AAAF	151	16.77	[C7orf65]

FLJ44139AAAF	450	49.72	[FAM189A2]
FLJ44146AAAF	186	20.65	[ACSM6]
FLJ44148AAAF	572	61.84	[AKNA]
FLJ44158AAAF	360	41.26	[AMZ2]
FLJ44175AAAF	924	98.2	[ARHGAP30]
FLJ44177AAAF	1260	139.18	[SH3TC1]
FLJ44182AAAF	271	29.38	[TPGS1]
FLJ44186AAAF	409	42.82	[KLRG2]
FLJ44189AAAF	507	57.73	[LIN9]
FLJ44193AAAF	121	13.58	[ATG2A]
FLJ44195AAAF	515	55.49	[SLC16A10]
FLJ44219AAAF	401	45.99	[B3GNT7]
FLJ44241AAAF	851	93.27	[PFKM]
FLJ44258AAAN	208	23.19	[NKAIN4]
FLJ44269AAAF	127	14.55	[APOC4]
FLJ44274AAAF	192	21.87	[AKIRIN1]
FLJ44298AAAF	548	61.39	[GAB3]
FLJ44299AAAF	304	34.78	[FAM92B]
FLJ44313AAAF	163	17.24	[C18orf65]
FLJ44321AAAF	331	37.08	[UBXN2B]
FLJ44324AAAF	527	57.61	[MCAM]
FLJ44338AAAF	209	22.08	[CLDN4]
FLJ44339AAAF	211	24.26	[SIKE1]
FLJ44373AAAN	403	47.73	[PIK3R1]
FLJ44374AAAF	369	43.38	[RASSF6]
FLJ44378AAAF	325	36.62	[CD200R1]
FLJ44383AAAF	366	41	[PLEKHS1]
FLJ44403AAAF	251	28.17	[UNC119B]
FLJ44407AAAF	192	21.86	[RPL9]
FLJ44415AAAF	287	30.5	[FAM122A]
FLJ44424AAAF	461	52.31	[CDC14B]
FLJ44438AAAF	191	21.84	[MRPL30]
FLJ44489AAAF	172	18.78	[HYKK]
FLJ44500AAAF	962	108.18	[EFTUD2]
FLJ44501AAAF	281	29.54	[TGOLN2]
FLJ44502AAAF	647	71.95	[ADCK3]
FLJ44514AAAN	934	107.99	[CCDC132]
FLJ44528AAAN	1253	142.67	[DOCK9]
FLJ44553AAAF	180	19.53	[LOC100130691]
FLJ44554AAAF	1222	140.22	[CADPS2]
FLJ44569AAAF	624	68.26	[GAS6]
FLJ44575AAAN	550	61.93	[LOC400499]
FLJ44589AAAF	402	44.62	[RNF128]
FLJ44604AAAF	310	33.66	[PSRC1]
FLJ44605AAAF	339	40.25	[TMEM120B]
FLJ44611AAAF	391	42.89	[NDRG4]
FLJ44614AAAF	135	15.44	[FAM101A]
FLJ44635AAAF	140	15.76	[TPT1]

FLJ44662AAAF	701	78.08	[PPFIA4]
FLJ44691AAAF	496	54.2	[LRIT3]
FLJ44706AAAF	418	46.54	[MMAA]
FLJ44733AAAN	1004	111.12	[LLGL2]
FLJ44734AAAN	180	20.14	[IGF2]
FLJ44737AAAF	616	71.54	[ZNF567]
FLJ44768AAAF	303	34.11	[FBXL17]
FLJ44773AAAF	498	54.65	[ZNF497]
FLJ44790AAAF	211	22.1	[OSBPL2]
FLJ44795AAAF	347	37.99	[DTX3]
FLJ44800AAAF	187	21.75	[ZNF833P]
FLJ44804AAAF	716	83.1	[DPY19L3]
FLJ44815AAAF	167	17.86	[C17orf102]
FLJ44831AAAN	756	81.07	[TRIM67]
FLJ44842AAAN	887	102.97	[TTLL7]
FLJ44854AAAN	969	107.97	[NIPBL]
FLJ44857AAAF	246	26.71	[RIMS4]
FLJ44858AAAF	493	55.3	[MICALL2]
FLJ44862AAAF	291	30.96	[SLC25A48]
FLJ44872AAAF	551	61.09	[MUS81]
FLJ44881AAAF	130	14.35	[LOC400661]
FLJ44882AAAF	160	17.12	[DLEU7]
FLJ44886AAAF	630	69.61	[LSR]
FLJ44887AAAF	575	66.45	[MYSM1]
FLJ44893AAAF	219	24.22	[LHFPL5]
FLJ44903AAAF	1101	122.19	[ANK3]
FLJ44906AAAN	854	94	[KDM4B]
FLJ44908AAAF	113	13.05	[NATD1]
FLJ44912AAAF	439	48.8	[PI4KAP2]
FLJ44914AAAF	537	61.05	[PDP1]
FLJ44923AAAF	743	86.45	[ZNF700]
FLJ44927AAAF	701	78.83	[EPB41L1]
FLJ44952AAAF	329	36.78	[FAM43B]
FLJ44964AAAN	629	69.79	[SYVN1]
FLJ44965AAAF	347	39.78	[LRP2BP]
FLJ44970AAAF	524	55.69	[GLIS2]
FLJ44973AAAF	1105	125.31	[SENP6]
FLJ44977AAAF	394	42.84	[NDRG1]
FLJ44984AAAF	249	28.65	[SPIN4]
FLJ44986AAAF	412	46.4	[DPF3]
FLJ44992AAAF	1349	153.33	[RIMS2]
FLJ44996AAAF	1042	116.55	[USP38]
FLJ45003AAAF	293	32.33	[HNRNPC]
FLJ45004AAAF	197	20.88	[FLCN]
FLJ45008AAAF	213	23.6	[TTYH2]
FLJ45024AAAF	1232	140.7	[PPFIA2]
FLJ45031AAAN	1006	110.31	[VCL]
FLJ45032AAAF	453	49.46	[TMPPE]

FLJ45033AAAF	834	92.05	[EML1]
FLJ45041AAAF	360	39.31	[ARMCX4]
FLJ45048AAAF	369	41.81	[KY]
FLJ45072AAAF	769	86.17	[PDZD4]
FLJ45075AAAF	641	74.08	[RUFY2]
FLJ45090AAAF	206	23.41	[MOBP]
FLJ45099AAAF	1032	112.72	[TSHZ1]
FLJ45102AAAF	1173	130.79	[ARHGEF18]
FLJ45104AAAF	511	57.69	[AMY2B]
FLJ45106AAAF	929	99.42	[SYNPO]
FLJ45122AAAF	645	71.91	[ZBTB47]
FLJ45133AAAN	1162	133.75	[SYNE1]
FLJ45146AAAN	926	100.63	[MTHFD1L]
FLJ45151AAAF	822	94.52	[CYFIP1]
FLJ45152AAAF	186	21.04	[VAX1]
FLJ45191SAAF	1675	191.61	[CLTC]
FLJ45192AAAF	603	68.99	[GALNT10]
FLJ45195AAAF	186	21.42	[ARL8A]
FLJ45197AAAF	199	22.49	[RAB7B]
FLJ45199AAAF	322	36.52	[PLCXD3]
FLJ45202AAAN	123	14.79	[FAM74A4]
FLJ45208AAAF	146	16.68	[CYP2G1P]
FLJ45209AAAF	327	37.96	[IQCD]
FLJ45212AAAF	121	13.91	[LINC00610]
FLJ45224AAAF	164	17.81	[LCNL1]
FLJ45231AAAF	131	14.41	[ZCCHC23]
FLJ45232AAAF	271	30.31	[MRPL10]
FLJ45233AAAF	1009	112.13	[GRID1]
FLJ45235AAAF	266	29.98	[ANKRD45]
FLJ45241AAAF	791	87.96	[MFSD6]
FLJ45242AAAF	279	31.93	[LRRC28]
FLJ45246AAAF	439	49.2	[ZNF621]
FLJ45256AAAF	202	22.96	[LINC01567]
FLJ45261AAAF	386	43.88	[ADIPOR2]
FLJ45267AAAF	406	46.72	[KLHDC1]
FLJ45276AAAN	597	65.88	[MRVI1]
FLJ45278AAAF	163	17.54	[DNAH10OS]
FLJ45281AAAF	465	52.41	[PTPN7]
FLJ45285AAAF	904	100.23	[ERMP1]
FLJ45293AAAF	905	100.45	[CTNNA2]
FLJ45302AAAF	328	36.82	[FLYWCH1]
FLJ45309AAAF	763	85.96	[SATB1]
FLJ45333AAAF	240	26.01	[PKD1L2]
FLJ45339AAAN	1211	136.98	[KCNT1]
FLJ45342AAAF	272	28.61	[LDLRAD2]
FLJ45349AAAN	871	97.25	[ARHGEF26]
FLJ45355AAAF	163	16.66	[LOC440896]
FLJ45362AAAF	641	71.9	[BTBD11]

FLJ45370AAAF	486	53.06	[SLC16A12]
FLJ45381AAAF	533	60.03	[PTPN5]
FLJ45389AAAF	465	49.47	[SLC16A3]
FLJ45390AAAF	176	20.58	[GSG1L]
FLJ45393AAAF	350	40.19	[ST3GAL2]
FLJ45395AAAF	991	110.44	[HYOU1]
FLJ45399AAAF	1265	144.77	[KDM2B]
FLJ45406AAAF	432	47.11	[NPTX1]
FLJ45415AAAF	422	45.77	[FAM53B]
FLJ45431AAAF	638	70.21	[SCNN1D]
FLJ45436AAAF	140	15.19	[C11orf39]
FLJ45446AAAF	981	109.7	[TDRD5]
FLJ45452AAAN	349	39.08	[NECAB2]
FLJ45457AAAF	453	50.49	[SERINC1]
FLJ45465AAAF	255	29.17	[YWHAE]
FLJ45489AAAF	719	81	[CAPN12]
FLJ45505AAAF	306	34.76	[FAM107B]
FLJ45513AAAF	130	13.72	[ROBO1]
FLJ45517AAAF	169	17.66	[C1orf64]
FLJ45537AAAF	121	13.37	[C9orf170]
FLJ45549AAAF	745	86.26	[GRAMD1B]
FLJ45557AAAF	479	52.91	[FAM196A]
FLJ45559AAAF	346	39.53	[RNF165]
FLJ45586AAAF	1176	129.52	[TRERF1]
FLJ45589AAAN	1375	149.71	[ANKRD50]
FLJ45605AAAF	931	104.85	[NCOA7]
FLJ45617AAAF	913	100.36	[RALGDS]
FLJ45624AAAF	117	12.74	[YAF2]
FLJ45629AAAF	496	54.67	[TBX15]
FLJ45637AAAN	264	29.48	[B3GNT6]
FLJ45639AAAF	224	24.96	[FAM3D]
FLJ45645AAAF	357	40.67	[RBM43]
FLJ45647AAAF	223	25.86	[GJB7]
FLJ45653AAAF	765	85.1	[ZBTB49]
FLJ45657AAAF	315	35.74	[C6orf136]
FLJ45659AAAF	575	62.6	[ACSS1]
FLJ45669AAAF	585	63.86	[YTHDF3]
FLJ45673AAAF	139	15.65	[LINC00299]
FLJ45674AAAF	211	24.26	[RPL13]
FLJ45678AAAF	161	18.25	[ANKRD54]
FLJ45679AAAF	362	41.97	[FAM172BP]
FLJ45686AAAF	341	37.65	[LRRC55]
FLJ45690AAAF	435	50.13	[SMARCD3]
FLJ45713AAAN	329	35.61	[WNT9B]
FLJ45717AAAF	237	24.36	[C1orf229]
FLJ45719AAAF	211	23.55	[SOCS1]
FLJ45721AAAF	163	18.14	[OLR865]
FLJ45723AAAF	682	76.47	[ZNF16]

FLJ45729AAAF	556	61.79	[JRK]
FLJ45738AAAF	371	41.2	[GRINA]
FLJ45739AAAF	529	59.07	[GK5]
FLJ45744AAAF	235	25.15	[RGS9BP]
FLJ45759AAAF	232	25.92	[C2orf49]
FLJ45763AAAF	571	62.12	[WDR1]
FLJ45770AAAF	450	50.7	[CSK]
FLJ45775AAAN	301	33.7	[NFYC]
FLJ45778AAAF	473	50.51	[C19orf35]
FLJ45786AAAN	129	14.15	[ADAMTSL4-AS1]
FLJ45793AAAF	270	29.85	[ADO]
FLJ45801AAAN	382	42.27	[FBXL19]
FLJ45803AAAF	124	13.4	[COLCA1]
FLJ45804AAAF	1366	148.94	[DCHS2]
FLJ45808AAAF	1181	128.81	[HEATR6]
FLJ45824AAAF	382	42.75	[LMX1A]
FLJ45825AAAF	159	17.35	[LOC100505530]
FLJ45827AAAF	478	56.05	[MGAT4C]
FLJ45831AAAF	121	13.06	[LOC400576]
FLJ45842AAAF	234	26.17	[MALSU1]
FLJ45843AAAF	576	63.94	[WFIKKN2]
FLJ45850AAAF	544	60.54	[ZNF324B]
FLJ45851AAAF	162	18.06	[TMC2]
FLJ45852AAAF	326	36.51	[ADORA1]
FLJ45854AAAF	572	62.21	[CRMP1]
FLJ45865AAAF	476	54.98	[ZNF776]
FLJ45879AAAF	198	20.98	[CXXC4]
FLJ45880AAAF	426	48.42	[ZNF662]
FLJ45881AAAF	439	48.43	[SDE2]
FLJ45893AAAF	1223	132.31	[FAM65A]
FLJ45894AAAF	1044	114.8	[R3HDM1]
FLJ45899AAAF	470	51.78	[EYA1]
FLJ45909AAAF	452	50.05	[RINL]
FLJ45911AAAF	378	42.87	[CCR7]
FLJ45913AAAF	719	79.33	[C10orf71]
FLJ45920AAAN	474	52.47	[ENOSF1]
FLJ45924AAAF	363	39.33	[GPR78]
FLJ45925AAAF	151	17.8	[WTAP]
FLJ45929AAAF	1204	132.87	[FNDC3B]
FLJ45931AAAF	280	31.95	[AADACL3]
FLJ45932AAAF	317	35.37	[PITX2]
FLJ45935AAAF	328	35	[HSD17B1]
FLJ45952AAAF	394	44.28	[MAGEA11]
FLJ45953AAAF	376	39.84	[SP6]
FLJ45957AAAF	202	22.31	[C9orf47]
FLJ45959AAAF	749	81.48	[ADAM12]
FLJ45977AAAF	209	22.5	[RBPMS2]
FLJ45987AAAF	803	92.36	[LRRC8C]

FLJ45992AAAF	291	32.43	[DUSP13]
FLJ45997AAAF	163	17.62	[TMEM107]
FLJ46007AAAF	640	73.38	[CROT]
FLJ46038AAAF	186	20.46	[SHROOM3]
FLJ46050AAAF	402	43.95	[ARRDC2]
FLJ46053AAAF	703	79.92	[PATL1]
FLJ46057AAAN	454	50.29	[SON]
FLJ46058AAAF	664	77.3	[ZNF17]
FLJ46061AAAF	821	85.94	[KANK3]
FLJ46063AAAF	515	56.07	[NAGPA]
FLJ46069AAAF	163	16.9	[LINC00696]
FLJ46078AAAF	172	18.06	[BOD1L2]
FLJ46079AAAF	459	53.25	[TTLL13]
FLJ46082AAAN	320	36.5	[C9orf171]
FLJ46083AAAF	572	66.6	[EFCAB12]
FLJ46088AAAF	566	66.63	[DEUP1]
FLJ46089AAAF	148	16.88	[KRT17P5]
FLJ46095AAAF	900	103.1	[SWT1]
FLJ46099AAAF	316	35.34	[OR2H1]
FLJ46104AAAF	498	56.54	[SLC38A9]
FLJ46112AAAF	230	24.96	[C1orf86]
FLJ46114AAAF	403	45.35	[ZDHHC23]
FLJ46116AAAF	265	28.27	[PRR23B]
FLJ46125AAAF	633	72.73	[ZSWIM2]
FLJ46131AAAF	211	23.13	[DECR2]
FLJ46133AAAF	304	34	[YOD1]
FLJ46156AAAF	1111	128.13	[LRRC9]
FLJ46157AAAF	190	21.32	[RNF4]
FLJ46164AAAF	1135	128.19	[POLR1B]
FLJ46165AAAF	1281	137.86	[RFX7]
FLJ46170AAAF	496	52.64	[ANKRD34A]
FLJ46178AAAF	1301	141.69	[ZNF532]
FLJ46182AAAF	357	39.23	[SLC35F2]
FLJ46190AAAF	513	54.9	[SLC41A1]
FLJ46198AAAN	576	65.18	[QSOX2]
FLJ46199AAAF	845	94.14	[AP1G1]
FLJ46204AAAF	225	25.5	[LOC100129240]
FLJ46205AAAF	1177	135.18	[SLC9C1]
FLJ46209AAAF	358	39.95	[CATSPERD]
FLJ46210AAAF	262	27.81	[PRR23C]
FLJ46214AAAF	208	21.16	[LOC100128770]
FLJ46218AAAF	1018	112.29	[HMHA1]
FLJ46225AAAF	1124	129.04	[SLC9C2]
FLJ46235AAAF	215	21.77	[LOC401357]
FLJ46244AAAF	600	67.37	[DCAF8L1]
FLJ46246AAAF	154	17.66	[C1QTNF6]
FLJ46253AAAF	945	103.64	[UNC5B]
FLJ46260AAAF	524	57.45	[TRIM46]

FLJ46264AAAF	1263	144.46	[SMC4]
FLJ46266AAAF	178	20.43	[C11orf88]
FLJ46269AAAF	389	44.51	[FAM46D]
FLJ46278AAAF	799	87.87	[BEND2]
FLJ46285AAAN	656	75.62	[APLP2]
FLJ46290AAAF	753	82.13	[MIB2]
FLJ46302AAAF	557	63.02	[SNX1]
FLJ46304AAAF	620	68.74	[KLHL8]
FLJ46312AAAF	664	76.04	[CNGA2]
FLJ46321AAAN	1576	175.65	[SPATA31D1]
FLJ46323AAAF	976	111.26	[FMNL3]
FLJ46327AAAF	191	21.46	[ERVK11-1]
FLJ46335AAAF	547	61.66	[ARHGAP36]
FLJ46344AAAF	798	87.55	[SLC45A4]
FLJ46353AAAN	799	91.78	[CATSPERG]
FLJ46369AAAF	221	24.12	[LRRC37A8P]
FLJ46370AAAF	153	16.64	[FAM159A]
FLJ46371AAAF	928	102.4	[TJP3]
FLJ46377AAAF	232	26.8	[CLEC12B]
FLJ46380AAAF	244	25.98	[RNF207]
FLJ46385AAAF	136	15.51	[ZNF818P]
FLJ46438AAAF	528	59	[ADGRG5]
FLJ46451AAAF	205	20.56	[MIEF2]
FLJ46460AAAF	589	64.99	[SLC17A8]
FLJ46479AAAF	334	36.32	[MUC15]
FLJ46481AAAF	276	30.62	[C4orf50]
FLJ46500AAAF	166	19.48	[ATAD3B]
FLJ46504AAAF	682	77.95	[ZNF615]
FLJ46506AAAF	325	35.73	[KCTD12]
FLJ46512AAAF	295	32.19	[PPAPDC2]
FLJ46530AAAF	1090	118.39	[FUK]
FLJ46544AAAF	445	46.46	[COLQ]
FLJ46558AAAF	190	19.9	[C17orf107]
FLJ46562AAAF	649	69.91	[CD6]
FLJ46581AAAF	501	58.78	[SF3A3]
FLJ46585AAAN	224	24.8	[PLSCR4]
FLJ46597AAAF	410	46.97	[FBXO3]
FLJ46599AAAF	869	95.18	[ATP2A1]
FLJ46607AAAF	235	25.32	[ABHD12B]
FLJ46608AAAF	310	34.67	[ZCCHC16]
FLJ46636AAAF	124	13.99	[C1orf140]
FLJ46641AAAF	194	19.41	[LOC730668]
FLJ46643AAAN	545	60.91	[SH3D19]
FLJ46645AAAF	157	17.9	[CYBRD1]
FLJ46648AAAF	491	56.84	[CLUL1]
FLJ46651AAAF	724	78.95	[SLCO4C1]
FLJ46678AAAF	431	44.99	[SP7]
FLJ46688AAAF	379	44.27	[PLEKHG7]

FLJ46689AAAN	756	84.68	[EPB41L3]
FLJ46696AAAF	347	36.68	[CCDC78]
FLJ46731AAAF	408	46.13	[DPAGT1]
FLJ46741AAAF	1020	109.73	[EDC4]
FLJ46751AAAF	1093	125.32	[DNAH12]
FLJ46756AAAF	570	63.08	[FKBP9]
FLJ46765AAAF	808	91.76	[VWA3A]
FLJ46769AAAF	221	24.69	[SLC22A5]
FLJ46774AAAF	516	58.31	[TSTD2]
FLJ46777AAAN	211	24.53	[POLR2M]
FLJ46786AAAF	938	101.37	[SMTN]
FLJ46788AAAF	661	75.04	[PUS7]
FLJ46792AAAF	126	14.34	[LOC100130976]
FLJ46798AAAF	405	44.81	[PRKAR2B]
FLJ46801AAAF	156	17.76	[C4orf22]
FLJ46805AAAF	161	17.28	[LRRC74B]
FLJ46813AAAF	568	62.34	[ZNF648]
FLJ46819AAAN	453	49.67	[C17orf97]
FLJ46828AAAF	215	23.9	[C19orf40]
FLJ46834AAAF	178	20.25	[ZNF720]
FLJ46863AAAF	698	78.43	[NOA1]
FLJ46874AAAF	160	17.35	[LOC100129940]
FLJ46876AAAF	518	56.75	[ALDH1A2]
FLJ46883AAAF	491	54.56	[ZSCAN22]
FLJ46898AAAF	1058	117.4	[ESYT1]
FLJ46899AAAF	482	51.94	[ATF2]
FLJ46904AAAF	145	17.26	[RPL26L1]
FLJ48001SAAF	105	11.91	[NDUFB1]
FLJ48005SAAF	314	35.19	[SIRT4]
FLJ50004AAAF	197	21.54	[SHISA4]
FLJ50006AAAF	400	45.19	[KIAA1045]
FLJ50009AAAF	326	37.21	[MPPED1]
FLJ50010AAAF	121	14.12	[YBEY]
FLJ50011AAAF	1068	118.57	[SPECC1]
FLJ50018AAAF	109	12.07	[TMEM233]
FLJ50021AAAF	276	31.64	[STX12]
FLJ50024AAAF	192	20.7	[MSRB3]
FLJ50025AAAF	713	82.71	[SDCCAG8]
FLJ50026AAAF	579	67.09	[ZNF248]
FLJ50031AAAN	423	45.81	[VSIG1]
FLJ50033AAAN	501	55.65	[TCTE1]
FLJ50034AAAF	175	20.36	[MORN5]
FLJ50035AAAF	366	40.95	[TDRD10]
FLJ50037AAAF	159	17.06	[ANKRD37]
FLJ50044AAAF	642	72.1	[PODN]
FLJ50045AAAF	330	37.93	[C6orf58]
FLJ50046AAAF	536	61.5	[ARSK]
FLJ50048AAAF	612	70.06	[RPGRIP1]

FLJ50050AAAF	563	64.46	[ZNF879]
FLJ50058AAAF	338	37.43	[TWISTNB]
FLJ50059AAAN	448	49.93	[PNMA5]
FLJ50061AAAF	327	36.88	[EMB]
FLJ50063AAAF	502	56.98	[TMEM161B]
FLJ50064AAAF	297	33.11	[MFAP4]
FLJ50065AAAF	686	77.61	[MAMDC2]
FLJ50072AAAF	1149	129.49	[KCNU1]
FLJ50073AAAF	173	19.73	[FAM180A]
FLJ50077AAAN	881	94.33	[CHRD]
FLJ50081AAAN	441	49.65	[ZNF500]
FLJ50082AAAF	205	22.92	[CD300E]
FLJ50083AAAF	185	19.2	[BOD1]
FLJ50088AAAF	150	16.63	[TMEM220]
FLJ50089AAAF	506	55.64	[PLD4]
FLJ50096AAAN	348	37.67	[LIN54]
FLJ50100AAAF	270	31.2	[NEBL]
FLJ50101AAAF	165	19.83	[FUCA2]
FLJ50102AAAF	742	81.23	[RNF43]
FLJ50106AAAF	491	55.72	[MTF2]
FLJ50107AAAF	178	19.87	[VKORC1L1]
FLJ50109AAAF	520	60.24	[CDC45]
FLJ50112AAAF	267	28.96	[SLC17A4]
FLJ50131AAAF	169	17.93	[CDIP1]
FLJ50134AAAF	748	84.38	[GRIK1]
FLJ50152AAAF	382	42.47	[SYT5]
FLJ50160AAAF	390	42.58	[SIGLEC8]
FLJ50182AAAN	413	47.85	[BRAP]
FLJ50186AAAN	497	55.81	[FSD1L]
FLJ50190AAAF	666	74.35	[CLCN4]
FLJ50204AAAF	326	34.99	[DECR1]
FLJ50210AAAF	573	64.49	[SLC44A3]
FLJ50212AAAN	220	24.63	[GTF2H4]
FLJ50263AAAF	270	30.94	[CCDC64]
FLJ50266AAAF	291	33.38	[CYP2B6]
FLJ50272AAAN	562	62.87	[EPS8]
FLJ50273AAAN	338	37.42	[PROCA1]
FLJ50294AAAF	503	58.66	[TOP1MT]
FLJ50302AAAF	470	52.33	[GPR176]
FLJ50314AAAF	834	92.7	[ARHGAP11A]
FLJ50319AAAF	113	12.83	[POP5]
FLJ50361AAAN	462	53.86	[TBC1D4]
FLJ50365AAAF	314	35.17	[CRISPLD1]
FLJ50395AAAN	1133	126.52	[CPD]
FLJ50398AAAF	1071	122.75	[IQGAP2]
FLJ50400AAAN	512	57.94	[DLG3]
FLJ50417AAAF	168	19.22	[CLEC7A]
FLJ50429AAAF	1113	124.05	[TSC1]

FLJ50447AAAF	353	40.77	[FDFT1]
FLJ50454AAAF	510	53.81	[GGT5]
FLJ50460AAAF	724	80.5	[DLG4]
FLJ50465AAAN	442	51.44	[KAT7]
FLJ50475AAAF	217	24.02	[CAP2]
FLJ50476AAAF	483	55	[ULK3]
FLJ50505AAAF	163	18.09	[NME6]
FLJ50509AAAN	788	87.22	[DLG1]
FLJ50510AAAN	840	94.36	[HSPA4]
FLJ50511AAAF	167	19.06	[LMO3]
FLJ50531AAAN	746	84.52	[APP]
FLJ50542AAAF	826	92.15	[GRIA1]
FLJ50546AAAN	306	35.8	[FAM172A]
FLJ50552AAAF	682	75.85	[LDLR]
FLJ50555AAAF	371	41.58	[BTBD3]
FLJ50561AAAF	362	41.47	[MORF4L1]
FLJ50610AAAN	674	73.79	[CDH13]
FLJ50612AAAF	182	21.35	[TPM3]
FLJ50626AAAF	677	77	[EZH1]
FLJ50635AAAF	447	50.43	[DDX19A]
FLJ50644AAAF	216	24.95	[AGPAT4]
FLJ50648AAAF	612	68.84	[CACNB2]
FLJ50678AAAF	649	72.19	[SLC9A6]
FLJ50713AAAN	255	29.75	[FBXO44]
FLJ50733AAAF	102	11.63	[FAM96A]
FLJ50735AAAF	956	106.1	[CLSTN3]
FLJ50745AAAF	421	47.49	[CCNA1]
FLJ50758AAAF	119	13.85	[HPCAL4]
FLJ50759AAAN	532	60.44	[SV2B]
FLJ50765AAAF	463	54.96	[ARHGEF9]
FLJ50776AAAN	649	73.46	[ARHGEF6]
FLJ50796AAAF	543	61.65	[AOAH]
FLJ50797AAAF	482	51.92	[LILRB2]
FLJ50805AAAF	691	77.04	[EPB42]
FLJ50812AAAF	122	13.78	[UBE2A]
FLJ50815AAAF	133	15.43	[TNFSF4]
FLJ50823AAAF	154	16.95	[OCIAD2]
FLJ50828AAAN	577	62.18	[FCAMR]
FLJ50838AAAF	1096	122.56	[ACIN1]
FLJ50843AAAF	596	67.74	[NET1]
FLJ50863AAAF	135	15.5	[SRSF6]
FLJ50864AAAF	157	16.67	[SIVA1]
FLJ50868AAAF	694	78.63	[FES]
FLJ50883AAAF	609	70.9	[ZNF527]
FLJ50888AAAF	470	50.93	[ALDH2]
FLJ50892AAAF	138	14.9	[MESDC2]
FLJ50897AAAF	627	69.15	[EHHADH]
FLJ50903AAAF	153	17.12	[RBP1]

FLJ50905AAAF	245	26.76	[ZNF575]
FLJ50914AAAF	487	54.3	[RBPJ]
FLJ50932AAAF	987	109.83	[BNC1]
FLJ50941AAAF	253	28.13	[BEND6]
FLJ50942AAAN	427	47.91	[SERPINF2]
FLJ50946AAAF	509	58.3	[LGI1]
FLJ50947AAAF	975	109.22	[CORIN]
FLJ50952AAAF	144	17	[AP4S1]
FLJ50966AAAN	756	86.53	[PMS1]
FLJ50967AAAF	448	50.44	[CKMT1A]
FLJ50979AAAF	391	44.35	[ILK]
FLJ51025AAAF	599	69.7	[NOX4]
FLJ51033AAAF	395	45.32	[ACP2]
FLJ51042AAAF	412	43.18	[SELPLG]
FLJ51052AAAN	1131	124.09	[SLC4A3]
FLJ51066AAAF	144	16.62	[RAB6A]
FLJ51104AAAF	493	50.54	[GGT6]
FLJ51121AAAF	558	60.89	[IL10RA]
FLJ51146AAAF	545	62.1	[SETMAR]
FLJ51188AAAF	584	65.81	[GNS]
FLJ51203AAAF	1085	120.18	[THBS1]
FLJ51209AAAF	172	17.9	[C17orf49]
FLJ51263AAAF	107	11.32	[DDT]
FLJ51268AAAF	150	16.48	[GCKR]
FLJ51277AAAF	186	20.09	[GM2A]
FLJ51278AAAF	279	30.98	[PTCD2]
FLJ51303AAAF	464	49.71	[HTRA4]
FLJ51305AAAF	361	40.7	[CLP1]
FLJ51323AAAF	405	43.63	[ACADS]
FLJ51327AAAF	231	26.48	[NOP16]
FLJ51388AAAN	461	53.08	[SNW1]
FLJ51390AAAF	481	54.85	[ZNF177]
FLJ51407AAAF	131	14.47	[INSL3]
FLJ51410AAAF	480	54.2	[B4GALNT2]
FLJ51413AAAF	689	78.26	[PLA2G4A]
FLJ51422AAAN	329	36.71	[IL9R]
FLJ51431AAAF	817	95.04	[ZNF33A]
FLJ51439AAAF	859	96.96	[GRIK2]
FLJ51440AAAF	605	67.5	[NCAPH]
FLJ51451AAAF	715	82.42	[SEMA3E]
FLJ51467AAAF	519	59.18	[TM9SF1]
FLJ51474AAAF	295	34.16	[ATP1B1]
FLJ51506AAAF	790	88.43	[OSBPL5]
FLJ51513AAAF	439	49.04	[PWP1]
FLJ51536AAAF	751	82.36	[TGM1]
FLJ51559AAAF	376	42.67	[EDEM1]
FLJ51561AAAF	104	11.58	[DNAJC5G]
FLJ51588AAAF	426	48.69	[MMP8]

FLJ51595AAAF	253	28.82	[MTX2]
FLJ51601AAAF	555	63.88	[GBP1]
FLJ51612AAAF	501	57.32	[CYP2U1]
FLJ51618AAAF	485	55.55	[NUP160]
FLJ51619AAAF	524	56.82	[PTBP3]
FLJ51624AAAF	263	30.09	[MIF4GD]
FLJ51632AAAF	606	65.54	[ZNF143]
FLJ51650AAAF	130	14.58	[MS4A1]
FLJ51663AAAF	1049	116.04	[CPS1]
FLJ51685AAAF	1001	116.08	[MCF2]
FLJ51687AAAF	430	47.9	[NFIC]
FLJ51689AAAF	282	31.61	[RNPS1]
FLJ51693AAAF	752	84.79	[KCNQ3]
FLJ51694AAAF	606	68.95	[ARHGAP25]
FLJ51700AAAF	208	23.3	[HDHD1]
FLJ51708AAAN	657	75.45	[PIK3CB]
FLJ51715AAAF	336	36.65	[SLC25A39]
FLJ51721AAAF	413	44.58	[SLC37A4]
FLJ51724AAAF	234	25.02	[NPAS1]
FLJ51742AAAF	888	98.35	[ITIH4]
FLJ51749AAAF	290	33.49	[SET]
FLJ51751AAAF	256	29.71	[PCGF5]
FLJ51754AAAF	513	57.91	[API5]
FLJ51756AAAF	318	35.52	[TBP]
FLJ51765AAAF	213	23.6	[MAPK11]
FLJ51800AAAF	1075	119.6	[PEX1]
FLJ51802AAAF	960	107.03	[NPC1]
FLJ51806AAAF	906	96.89	[PELP1]
FLJ51813AAAF	122	13.85	[RASD1]
FLJ51826AAAF	502	56.42	[CACNB4]
FLJ51827AAAF	172	19.78	[CAPZA2]
FLJ51833AAAF	204	23.59	[ATP6V1E1]
FLJ51834AAAF	205	23.18	[LASP1]
FLJ51835AAAF	1018	113.7	[ERCC5]
FLJ51844AAAF	440	49.3	[SERINC3]
FLJ51845AAAF	342	38.95	[PPP6C]
FLJ51853AAAF	319	34.08	[NUBPL]
FLJ51855AAAF	255	27.57	[RTN3]
FLJ51860AAAF	520	58.44	[SLC6A8]
FLJ51863AAAF	729	82.1	[UFL1]
FLJ51871AAAF	462	53.7	[GABRR1]
FLJ51876AAAF	514	58.11	[CHRNA2]
FLJ51883AAAF	375	41.6	[MRPL3]
FLJ51885AAAF	505	57.62	[SOCS5]
FLJ51904AAAF	645	70.65	[PHACTR2]
FLJ51913AAAF	231	24.56	[SFTPA1]
FLJ51925AAAF	516	57.56	[PROC]
FLJ51927AAAF	709	78.8	[RPS6KA4]

FLJ51935AAAF	471	53.09	[BTN3A1]
FLJ51936AAAF	598	67.7	[TNIP1]
FLJ51937AAAF	177	19.4	[CEACAM3]
FLJ51938AAAF	145	15.9	[NTMT1]
FLJ51941AAAF	438	50.1	[DNAJC7]
FLJ51972AAAF	144	16.25	[IL33]
FLJ52012AAAN	198	22.94	[CCNC]
FLJ52030AAAF	148	16.62	[DSTN]
FLJ52043AAAF	160	18.51	[THAP6]
FLJ52044AAAF	238	27.42	[CNOT8]
FLJ52059AAAF	234	24.87	[IL15RA]
FLJ52062AAAF	237	25.96	[STOM]
FLJ52066AAAF	209	23.33	[PEX19]
FLJ52072AAAF	777	88.03	[IFT80]
FLJ52074AAAF	827	95.83	[CCDC110]
FLJ52075AAAF	629	68.5	[SPTY2D1]
FLJ52077AAAF	1253	144.8	[RPGRIP1L]
FLJ52079AAAF	926	104.94	[KCTD19]
FLJ52082AAAF	467	55.57	[ZMAT1]
FLJ52085AAAF	567	62.01	[KCTD3]
FLJ52094AAAF	865	95.53	[ARMC3]
FLJ52099AAAF	621	67.11	[FAM63B]
FLJ52102AAAF	258	27.97	[TMEM99]
FLJ52103AAAF	754	85.6	[C17orf104]
FLJ52105AAAF	778	89.09	[PDZRN4]
FLJ52108AAAF	651	71.5	[POLH]
FLJ52113AAAF	843	96.4	[TTLL6]
FLJ52129AAAF	100	11.39	[LIAS]
FLJ52158AAAF	126	14.69	[TMEM136]
FLJ52161AAAF	141	15.25	[EMP1]
FLJ52181AAAF	197	23.58	[PITPNA]
FLJ52188AAAF	307	34.67	[MFNG]
FLJ52198AAAF	145	16.62	[SCIMP]
FLJ52202AAAF	151	16.1	[NKX2-5]
FLJ52212AAAF	146	16.56	[CXorf40A]
FLJ52232AAAF	209	24.4	[GSTO2]
FLJ52241AAAF	235	26.76	[ING1]
FLJ52245AAAF	460	52.21	[IFT57]
FLJ52276AAAF	338	37.27	[OPCML]
FLJ52298AAAF	154	17.27	[ALKBH6]
FLJ52299AAAF	173	19.47	[KCNMB3]
FLJ52301AAAF	879	99.79	[DRP2]
FLJ52312AAAF	645	71.11	[RPH3A]
FLJ52322AAAF	650	72.76	[PTPN12]
FLJ52324AAAF	418	46.47	[PLAG1]
FLJ52325AAAF	668	75.14	[KIAA0753]
FLJ52349AAAF	827	94.16	[PKN2]
FLJ52352AAAF	240	27.54	[DNAJA1]

FLJ52354AAAF	429	49.08	[CHRNB1]
FLJ52364AAAF	860	97.46	[HSPH1]
FLJ52369AAAF	916	87.98	[NUP214]
FLJ52372AAAF	654	72.29	[PEX5]
FLJ52381AAAF	387	43.97	[ERG]
FLJ52388AAAF	622	67.75	[CCDC17]
FLJ52392AAAF	350	38.87	[TP73]
FLJ52393AAAF	520	59.74	[CYP4F3]
FLJ52394AAAF	739	83.54	[RGL1]
FLJ52395AAAF	469	53.66	[NR5A2]
FLJ52396AAAF	539	61.76	[SPARCL1]
FLJ52402AAAF	698	78.53	[SLC15A2]
FLJ52403AAAF	430	47.23	[FRMD8]
FLJ52410AAAF	426	46.62	[MFSD1]
FLJ52417AAAF	272	29.19	[BPNT1]
FLJ52424AAAF	349	39.24	[TFDP2]
FLJ52426AAAF	747	79.59	[ATXN7]
FLJ52431AAAF	816	93.85	[RB1]
FLJ52432AAAF	561	63.83	[LTA4H]
FLJ52438AAAF	826	92.73	[RIMS1]
FLJ52451AAAF	291	32.15	[LRTOMT]
FLJ52453AAAF	767	88.81	[AMPD3]
FLJ52456AAAF	129	15	[PDCD5]
FLJ52468AAAF	197	22.36	[ING4]
FLJ52485AAAF	174	20.03	[NARS]
FLJ52486AAAF	239	25.77	[TSC2]
FLJ52500AAAF	234	24.62	[SLC1A4]
FLJ52503AAAF	172	20.04	[NDUFB8]
FLJ52518AAAF	470	51.87	[PGD]
FLJ52519AAAF	890	100.7	[LIMCH1]
FLJ52537AAAF	134	14.95	[C8orf46]
FLJ52544AAAF	207	22.48	[TPD52]
FLJ52605AAAF	320	37.52	[ZDHHC20]
FLJ52626AAAF	191	20.77	[C6orf120]
FLJ52634SAAF	377	42.25	[FFAR4]
FLJ52647AAAF	187	20.96	[CDNF]
FLJ52650AAAF	123	13.41	[SYNPO2]
FLJ52655AAAF	280	31.05	[NHLRC3]
FLJ52658AAAF	187	21.4	[CFAP221]
FLJ52662AAAF	233	26.11	[FAM173B]
FLJ52663AAAF	418	47.31	[SMYD5]
FLJ52665AAAF	461	51.34	[SERINC5]
FLJ52670AAAF	310	34.5	[AMMECR1L]
FLJ52671AAAF	332	37.87	[SRRD]
FLJ52673AAAF	202	21.26	[BLOC1S3]
FLJ52685AAAF	416	44.18	[SLC35D3]
FLJ52686AAAF	227	24.98	[LYSMD1]
FLJ52687AAAF	137	15.33	[PIRT]

FLJ52692AAAF	281	31.18	[TATDN3]
FLJ52702AAAF	329	35.97	[CD44]
FLJ52713AAAF	394	44.91	[LRRFIP1]
FLJ52714AAAF	299	33.17	[BHMT2]
FLJ52717AAAF	256	28.93	[BTN2A2]
FLJ52725AAAF	446	50.58	[MRS2]
FLJ52728AAAF	401	45.34	[IFRD1]
FLJ52732AAAF	402	45.55	[ZNF587B]
FLJ52754AAAF	305	34.98	[AMD1]
FLJ52756AAAF	253	29.3	[MED6]
FLJ52783AAAF	274	29.9	[PDHX]
FLJ52805AAAF	168	19.28	[DNAI1]
FLJ52840AAAF	348	39.55	[CLEC4M]
FLJ52845AAAF	224	25.16	[RHNO1]
FLJ52861AAAF	275	30.86	[HSDL1]
FLJ52870AAAF	196	21.36	[MED29]
FLJ52872AAAF	329	35.92	[ABI1]
FLJ52877AAAF	461	51.31	[PSMD5]
FLJ52889AAAF	437	48.04	[ALDH8A1]
FLJ52890AAAF	473	53.66	[RFX8]
FLJ52924AAAF	415	45	[DYNC1LI2]
FLJ52927AAAF	225	24.02	[QTRT1]
FLJ52930AAAF	348	37.01	[RAD51D]
FLJ52938AAAF	188	21.74	[CLEC1A]
FLJ52997AAAF	191	19.76	[CACFD1]
FLJ53012AAAF	242	27.31	[TUBB]
FLJ53021AAAF	287	32.46	[HAX1]
FLJ53030AAAF	258	30.06	[MTUS2]
FLJ53032AAAF	211	24.07	[TMED4]
FLJ53057AAAF	195	21.4	[MS4A7]
FLJ53062AAAF	168	19.7	[STMN2]
FLJ53064AAAF	387	43.43	[SERPINE1]
FLJ53095AAAF	365	40.72	[ANXA8]
FLJ53097AAAF	176	20.47	[C4orf26]
FLJ53101AAAF	254	26.91	[HMGCL]
FLJ53112AAAF	419	47.63	[HLA-F]
FLJ53160AAAF	485	52.03	[ZYX]
FLJ53161AAAF	543	59.84	[DAGLB]
FLJ53166AAAF	536	58.16	[DPYSL2]
FLJ53176AAAF	688	74.81	[NASP]
FLJ53195AAAF	618	66.35	[CCDC120]
FLJ53213AAAF	660	75.68	[SGSM3]
FLJ53225AAAF	535	59.67	[BTN3A3]
FLJ53231AAAF	508	55.26	[ALDH9A1]
FLJ53232AAAF	686	80.02	[HOOK1]
FLJ53243AAAF	1134	122.89	[KIAA1683]
FLJ53251AAAF	627	70.66	[NT5C1B]
FLJ53256AAAF	424	47.5	[VRK3]

FLJ53259AAAF	757	82.13	[DENND1C]
FLJ53263AAAF	1074	119.69	[JARID2]
FLJ53266AAAF	483	53.75	[AHCYL1]
FLJ53268AAAF	505	58.33	[GUSB]
FLJ53269AAAF	483	55.24	[SMARCD2]
FLJ53270AAAF	685	79.55	[DNAH3]
FLJ53272AAAF	1275	145.45	[LMO7]
FLJ53280AAAF	490	56.03	[PTGS1]
FLJ53295AAAF	419	47.48	[KCTD20]
FLJ53300AAAF	406	42.23	[SLC35A2]
FLJ53318AAAF	784	90.11	[RXFP1]
FLJ53319AAAF	685	76.25	[DLGAP1]
FLJ53324AAAF	1221	137.33	[TJP2]
FLJ53331AAAF	691	79.58	[LRRC49]
FLJ53333AAAF	516	58.88	[CDC25B]
FLJ53335AAAF	532	61.44	[NT5C2]
FLJ53339AAAF	395	44.17	[DTNA]
FLJ53345AAAF	821	92.39	[LZTR1]
FLJ53346AAAF	900	98.62	[DNAJC6]
FLJ53349AAAF	601	69.82	[TM9SF4]
FLJ53357AAAF	900	99.34	[PSMD2]
FLJ53369AAAF	440	49.89	[CPE]
FLJ53387AAAF	595	64.89	[ELF1]
FLJ53405AAAF	723	79.16	[SLC26A6]
FLJ53406AAAF	719	81.95	[UTP14A]
FLJ53407AAAF	555	63.57	[INTS3]
FLJ53410AAAF	738	85.13	[EIF3B]
FLJ53418AAAF	854	93.57	[CLCN2]
FLJ53425AAAF	665	69.86	[FUBP1]
FLJ53429AAAF	638	74.49	[HMMR]
FLJ53431AAAF	479	55.82	[CDC23]
FLJ53436AAAF	835	92.74	[KCNH6]
FLJ53439AAAF	562	61.74	[TKTL1]
FLJ53441AAAF	681	77.22	[ASCC2]
FLJ53446AAAF	579	66.08	[MPP4]
FLJ53464AAAF	756	86.86	[TARS]
FLJ53478AAAF	573	64.1	[LGALS3BP]
FLJ53493AAAF	732	81.13	[CASS4]
FLJ53496AAAF	415	47.31	[CASC4]
FLJ53497AAAF	356	40.8	[MKS1]
FLJ53500AAAF	511	57.45	[SMG6]
FLJ53506AAAF	519	57.51	[SIGLEC10]
FLJ53549AAAF	242	28.04	[DPY19L2P1]
FLJ53564AAAF	193	21.47	[HIST2H2BC]
FLJ53567AAAF	482	55.24	[SYNC]
FLJ53568AAAF	436	47.78	[PM20D2]
FLJ53572AAAF	787	84.68	[ZFYVE28]
FLJ53576AAAF	1231	137.67	[ZFAT]

FLJ53579AAAF	954	106.61	[ITGA6]
FLJ53589AAAF	868	98.87	[PTPN3]
FLJ53593AAAF	1086	126.27	[USP7]
FLJ53595AAAF	460	52.42	[IDS]
FLJ53597AAAF	504	57.59	[CDK18]
FLJ53656AAAF	311	34.6	[SLC7A8]
FLJ53661AAAF	267	29.24	[LDB3]
FLJ53662AAAF	343	38.58	[ACTA1]
FLJ53670AAAF	355	39.2	[EFEMP1]
FLJ53672AAAF	551	61.06	[HACL1]
FLJ53678AAAF	414	46.11	[NDUFAF7]
FLJ53679AAAF	203	23.09	[PPT1]
FLJ53684AAAF	516	59.05	[PDE1B]
FLJ53686AAAF	641	72.39	[ANXA6]
FLJ53691AAAF	678	74.83	[TF]
FLJ53703AAAF	435	48.57	[HARS]
FLJ53708AAAF	248	27.4	[RDH11]
FLJ53716AAAF	247	28.16	[VIPR1]
FLJ53717AAAF	176	20.64	[ZC4H2]
FLJ53736AAAF	319	34.55	[PKNOX1]
FLJ53740AAAF	390	44.19	[SKP2]
FLJ53749AAAF	284	31.51	[NUP43]
FLJ53751AAAF	388	43.53	[CD1E]
FLJ53761AAAF	327	34.65	[CD68]
FLJ53781AAAF	371	42.03	[TMEM194A]
FLJ53782AAAF	320	35.16	[FRMPD2B]
FLJ53798AAAF	646	71.08	[SLC27A1]
FLJ53799AAAF	257	28.95	[RGS7BP]
FLJ53800AAAF	139	15.75	[AKR1C2]
FLJ53805AAAF	240	25.91	[METTL12]
FLJ53807AAAF	206	22.94	[ERLIN2]
FLJ53808AAAF	541	59.83	[DMPK]
FLJ53810AAAF	636	74.54	[ZNF790]
FLJ53811AAAF	120	14.64	[NUFIP2]
FLJ53812AAAF	217	24.85	[CHIC1]
FLJ53814AAAF	145	17.33	[BUD31]
FLJ53816AAAF	227	26.84	[SULT1C4]
FLJ53839AAAF	383	43.88	[ENPP5]
FLJ53850AAAF	752	85.79	[PYGB]
FLJ53870AAAF	475	54.75	[DHCR24]
FLJ53882AAAF	488	56.82	[TPTE2]
FLJ53897AAAF	139	15.11	[C19orf54]
FLJ53920AAAF	592	67.46	[TGFBR2]
FLJ53922AAAF	340	36.51	[DUSP1]
FLJ53926AAAF	406	44.09	[ENO3]
FLJ53927AAAF	540	62.01	[HEXA]
FLJ53930AAAF	521	56.13	[ELF2]
FLJ53932AAAF	406	46.4	[NDUFS2]

FLJ53942AAAF	798	90.38	[USP13]
FLJ53952AAAF	474	54.25	[FGB]
FLJ53958AAAF	358	37.94	[LTB4R2]
FLJ53964AAAF	375	41.27	[CREB3L4]
FLJ53975AAAF	426	44.64	[ACAT2]
FLJ53984AAAF	411	46.43	[ATG4D]
FLJ54007AAAF	631	73.01	[ELMO1]
FLJ54018AAAF	474	51.25	[PDLIM5]
FLJ54046AAAF	427	46.85	[MFSD4]
FLJ54052AAAF	783	86.64	[CTNNA1]
FLJ54054AAAF	204	22.78	[SLAMF7]
FLJ54057AAAF	847	96.29	[GANAB]
FLJ54060AAAF	380	42.11	[APOL1]
FLJ54088AAAF	684	76.98	[KIF2C]
FLJ54099AAAF	1037	116.98	[IPO5]
FLJ54101AAAF	160	17.36	[MBP]
FLJ54106AAAF	325	34.89	[VAT1]
FLJ54136AAAF	527	58.51	[PSAP]
FLJ54147AAAF	207	23.6	[DPF2]
FLJ54151AAAF	270	30.49	[ANXA8L1]
FLJ54156AAAF	443	48.96	[ARFGAP3]
FLJ54164AAAF	292	31.76	[SDCBP]
FLJ54166AAAF	355	37.45	[CD34]
FLJ54178AAAF	450	47.89	[SLC39A7]
FLJ54195AAAF	400	43.99	[DAW1]
FLJ54200AAAF	367	40.87	[HERPUD1]
FLJ54214AAAF	142	16.34	[DCAF7]
FLJ54220AAAF	664	74.25	[ACSL1]
FLJ54265AAAF	746	81.52	[ITGB6]
FLJ54268AAAF	264	30.48	[ERMN]
FLJ54269AAAF	258	28.49	[DOK2]
FLJ54279AAAF	209	24.01	[HCLS1]
FLJ54292AAAF	665	73.05	[KIFC1]
FLJ54303AAAF	572	62.45	[HSPA1A]
FLJ54311AAAF	693	78.56	[RANBP6]
FLJ54313AAAF	751	84.09	[OLFML2B]
FLJ54316AAAF	466	51.34	[POLD3]
FLJ54319AAAF	959	111.19	[CEP152]
FLJ54320AAAN	972	109.45	[KIT]
FLJ54322AAAF	867	96.18	[SEC24B]
FLJ54324AAAF	242	27.13	[ELP6]
FLJ54327AAAF	229	25.81	[TRMT10B]
FLJ54328AAAF	618	67.53	[HSPA1B]
FLJ54332AAAF	560	64.91	[LMOD3]
FLJ54334AAAF	649	75.03	[ORC3]
FLJ54340AAAF	530	58.6	[DGCR2]
FLJ54356AAAF	903	101.92	[XPC]
FLJ54372AAAF	598	63.82	[TOX4]

FLJ54380AAAF	511	57.33	[IFT172]
FLJ54381AAAF	703	77.96	[NSF]
FLJ54386AAAF	619	71.19	[MELK]
FLJ54396AAAF	1040	113.27	[KDM6A]
FLJ54397AAAF	677	76.42	[BTAF1]
FLJ54409AAAF	905	102.84	[DST]
FLJ54414AAAF	565	63.77	[WSCD2]
FLJ54415AAAF	339	39.47	[CYTH1]
FLJ54427AAAF	1076	115.56	[RBMXL3]
FLJ54428AAAF	696	77.7	[SLITRK1]
FLJ54435AAAF	510	57.96	[ENTPD1]
FLJ54439AAAF	788	90.5	[ATP6V0A1]
FLJ54440AAAF	628	70.91	[ARHGEF37]
FLJ54443AAAF	756	85.9	[CPXM2]
FLJ54448AAAF	704	81.19	[FAM111B]
FLJ54449AAAF	781	81.93	[SP3]
FLJ54451AAAF	519	59.81	[STIP1]
FLJ54453AAAF	725	82.25	[QARS]
FLJ54458AAAF	387	42.48	[WIF1]
FLJ54464AAAF	764	86.95	[STAT5A]
FLJ54467AAAF	505	57.7	[TRABD2A]
FLJ54468AAAF	630	71.17	[SASS6]
FLJ54469AAAF	900	104.15	[NEDD4]
FLJ54471AAAF	719	81.89	[C1R]
FLJ54472AAAF	822	91.75	[SECISBP2]
FLJ54476AAAF	657	71.95	[LPPR4]
FLJ54478AAAF	578	63.03	[EBF1]
FLJ54479AAAF	503	55.12	[ALDH4A1]
FLJ54480AAAF	657	76.45	[TMEM232]
FLJ54483AAAF	718	82.42	[TLK2]
FLJ54489AAAF	609	67.38	[PDE12]
FLJ54492AAAF	616	69.73	[EIF4B]
FLJ54495AAAF	1025	112.35	[NEFH]
FLJ54497AAAF	458	51.49	[FAM155A]
FLJ54502AAAF	475	52.04	[FEZF1]
FLJ54503AAAF	421	46.28	[ACOT1]
FLJ54515AAAF	923	102.28	[ARHGAP4]
FLJ54531AAAF	504	55.84	[SLC43A3]
FLJ54532AAAF	381	43.24	[SMAD3]
FLJ54538AAAF	880	99.67	[TNPO2]
FLJ54539AAAF	271	30.36	[GPM6A]
FLJ54551AAAF	410	47.88	[SEPT7]
FLJ54565AAAF	577	65.7	[TTC39A]
FLJ54576AAAF	729	83.28	[ASPH]
FLJ54577AAAF	562	62.94	[HDAC9]
FLJ54578AAAF	357	40.48	[ZNF562]
FLJ54584AAAF	472	51.76	[ALPL]
FLJ54586AAAF	565	62.12	[SLC8B1]

FLJ54588AAAF	884	98.3	[MVP]
FLJ54602AAAF	557	63.63	[STXBP1]
FLJ54623AAAF	628	68.55	[SH3KBP1]
FLJ54628AAAF	137	15.2	[UMAD1]
FLJ54629AAAF	692	76.77	[TBC1D25]
FLJ54630AAAF	660	75.82	[MTMR7]
FLJ54639AAAF	1048	115.95	[ITGA8]
FLJ54642AAAF	475	52.61	[APBB1]
FLJ54648AAAF	414	46.69	[LRR1]
FLJ54657AAAF	549	58.54	[KRT6A]
FLJ54660AAAF	646	73.15	[ZFP37]
FLJ54664AAAF	512	57	[ETV3]
FLJ54677AAAF	204	22.29	[CCDC117]
FLJ54679AAAF	723	81.78	[RPS6KA5]
FLJ54686AAAF	1217	136.25	[ARHGAP21]
FLJ54687AAAF	424	48.19	[TBCEL]
FLJ54691AAAF	580	65.76	[TBXAS1]
FLJ54700AAAF	662	77.15	[NOL10]
FLJ54714AAAF	124	14.59	[AP3S2]
FLJ54717AAAF	1024	113.2	[ATP1A3]
FLJ54721AAAF	824	88.72	[ADAM15]
FLJ54724AAAF	348	39.88	[DLG2]
FLJ54725AAAF	589	67.48	[ANKRD13A]
FLJ54727AAAF	500	57.27	[PFKFB3]
FLJ54732AAAF	860	93.29	[SORBS1]
FLJ54737AAAF	613	65.39	[SF1]
FLJ54741AAAF	831	94.64	[CARS]
FLJ54745AAAF	848	95.22	[SAFB]
FLJ54748AAAF	1019	115.5	[OGDH]
FLJ54751AAAF	370	42.03	[SUV420H1]
FLJ54754AAAF	422	46.41	[LSM14A]
FLJ54760AAAF	248	28.61	[TPM1]
FLJ54764AAAF	408	46.22	[ZSCAN32]
FLJ54768AAAF	585	66.15	[ZC3H14]
FLJ54773AAAF	405	44.91	[MKNK2]
FLJ54774AAAF	224	24.12	[FAM213B]
FLJ54775AAAF	604	67.7	[STXBP2]
FLJ54779AAAF	510	56.39	[PODNL1]
FLJ54789AAAF	392	44.09	[CARD8]
FLJ54795AAAF	253	27.42	[MS4A6A]
FLJ54810AAAF	324	36.95	[PCGF3]
FLJ54831AAAF	218	24.69	[SYCE2]
FLJ54836AAAF	330	36.82	[OXNAD1]
FLJ54844WAAF	197	21.73	[GUK1]
FLJ54849AAAF	270	30.8	[NTF3]
FLJ54853AAAF	142	15.96	[BLOC1S2]
FLJ54865AAAF	389	43.66	[UBE3D]
FLJ54866AAAF	301	33.98	[EVPLL]

FLJ54868AAAF	703	77.67	[RNF214]
FLJ54872AAAF	540	63.57	[ZNF461]
FLJ54873AAAF	440	49.11	[NCEH1]
FLJ54882AAAF	567	63.47	[SEPT9]
FLJ54884AAAF	597	67.28	[SLC9A8]
FLJ54885AAAF	260	29.65	[KCTD21]
FLJ54888AAAF	253	27.03	[CFD]
FLJ54895AAAN	614	68.94	[CRY2]
FLJ54898AAAF	291	32.53	[TMEM41B]
FLJ54902AAAF	182	19.84	[PRR29]
FLJ54903AAAF	383	44.4	[SAXO2]
FLJ54906AAAF	1006	114.85	[TMC5]
FLJ54913AAAF	503	54.35	[MSL2]
FLJ54917AAAF	373	41.42	[SH2B3]
FLJ54920AAAF	330	36.4	[ZNF747]
FLJ54926AAAF	422	47.61	[C1orf27]
FLJ54942AAAF	385	43.17	[BIN1]
FLJ54952AAAF	258	29.18	[SPIN3]
FLJ54988AAAF	501	57.47	[PDE1A]
FLJ54994AAAF	196	22.12	[MPV17L]
FLJ55024AAAF	527	61.01	[SPDL1]
FLJ55042AAAF	824	95.35	[ODF2]
FLJ55044SAAF	736	81.88	[DNM1L]
FLJ55061AAAF	231	26.22	[RPAIN]
FLJ55063AAAF	164	19.03	[TRAFD1]
FLJ55071AAAF	334	38.35	[SPATA6L]
FLJ55072AAAF	616	67.25	[SDHA]
FLJ55085AAAF	212	24.05	[GNPDA1]
FLJ55086AAAF	148	17.28	[TSN]
FLJ55095AAAF	289	32.05	[GLOD4]
FLJ55099AAAF	336	36.73	[PHF23]
FLJ55102AAAF	195	22.23	[PDCD2]
FLJ55104AAAF	165	18.45	[TRAPPC4]
FLJ55109AAAF	204	23.51	[MOB4]
FLJ55110AAAF	123	13.28	[PACRGL]
FLJ55133AAAF	204	23.22	[DAP]
FLJ55149AAAF	315	36.55	[TYW5]
FLJ55158WAAF	166	18.38	[TXN2]
FLJ55164AAAF	417	46.67	[RAD9B]
FLJ55176AAAF	429	47.7	[GPRC5B]
FLJ55196AAAF	213	24.11	[TVP23A]
FLJ55207AAAF	259	28.87	[EVI2A]
FLJ55219AAAF	379	41.93	[DHRS9]
FLJ55243AAAF	197	22.77	[RCAN1]
FLJ55248AAAF	254	28.46	[NFU1]
FLJ55290AAAF	659	74.67	[GALC]
FLJ55294AAAF	1379	155.05	[KDM5C]
FLJ55307AAAF	364	41.45	[BDKRB2]

FLJ55312AAAF	600	69.83	[NGEF]
FLJ55323AAAF	478	52.86	[B4GALNT1]
FLJ55329AAAF	703	81.14	[PLOD2]
FLJ55330AAAF	1283	141.75	[ERBB3]
FLJ55332AAAF	889	96.23	[PTPRN]
FLJ55333AAAF	1012	115.08	[USP8]
FLJ55335AAAF	1078	119.23	[PTK7]
FLJ55336AAAF	466	53.2	[BTN2A1]
FLJ55350AAAF	362	42.01	[ORC4]
FLJ55356AAAF	440	51.55	[DEPDC1B]
FLJ55367AAAF	650	75.54	[SDAD1]
FLJ55369AAAF	805	91.33	[IFT88]
FLJ55394AAAF	564	64.03	[GLB1L]
FLJ55395AAAF	187	21.61	[BLOC1S5]
FLJ55414AAAF	267	28.64	[SPSB2]
FLJ55416AAAF	198	22.56	[LCN2]
FLJ55421AAAF	261	29.83	[FAM78B]
FLJ55424AAAF	197	20.62	[C11orf87]
FLJ55436AAAF	563	63.03	[PTPN22]
FLJ55437AAAF	578	64.87	[DCAF8]
FLJ55439AAAF	568	65.64	[TRAF5]
FLJ55440AAAF	1227	142.59	[CYFIP2]
FLJ55453AAAF	951	108.31	[BRDT]
FLJ55462AAAF	573	64.3	[ZNF74]
FLJ55463AAAF	567	64.6	[RUFY3]
FLJ55464AAAF	494	55.76	[ARHGAP19]
FLJ55467AAAF	396	45.46	[SEPT2]
FLJ55472AAAF	573	61.51	[ZBTB7B]
FLJ55476AAAF	542	60.58	[SYNE2]
FLJ55483AAAF	620	70.46	[ZNF300]
FLJ55488AAAF	523	58.79	[ACBD5]
FLJ55495AAAF	357	40.53	[PCYT2]
FLJ55497AAAF	737	85.4	[N4BP2L2]
FLJ55498AAAF	402	45.55	[WDR48]
FLJ55500AAAF	675	75.38	[CD22]
FLJ55501AAAF	423	48.51	[BZW1]
FLJ55506AAAF	915	102.93	[NPEPPS]
FLJ55507AAAF	521	59.21	[TESPA1]
FLJ55513AAAF	566	60.82	[ZSCAN18]
FLJ55514AAAF	1157	128.65	[EGFR]
FLJ55521AAAF	866	96.85	[NELL2]
FLJ55523AAAF	714	77	[TAF6]
FLJ55525AAAF	463	52.65	[CES4A]
FLJ55557AAAF	585	63.88	[EYA4]
FLJ55562AAAF	369	40.79	[ELAVL4]
FLJ55569AAAF	417	47.67	[SCRN3]
FLJ55580AAAF	891	98.28	[SYNJ2]
FLJ55585AAAF	479	52.53	[C7orf25]

FLJ55589AAAF	392	43.14	[LHX6]
FLJ55591AAAF	532	60.78	[ARHGEF3]
FLJ55596AAAF	1142	123.68	[MGA]
FLJ55599AAAF	853	95.91	[MCM3]
FLJ55605AAAF	421	48.31	[FAM69A]
FLJ55610AAAF	353	40.55	[PRMT1]
FLJ55620AAAF	373	43.27	[KIF3B]
FLJ55623AAAF	677	77.34	[RNF145]
FLJ55642AAAF	637	69.21	[SGIP1]
FLJ55668AAAF	1214	134.9	[ZMYND8]
FLJ55689AAAF	582	64.85	[LMOD1]
FLJ55692AAAF	387	42.99	[GOT2]
FLJ55697AAAF	749	84.49	[SEC23B]
FLJ55701AAAF	677	74.36	[VCAM1]
FLJ55707AAAF	642	71.86	[ARMC8]
FLJ55710AAAF	872	98.1	[KIF20A]
FLJ55720AAAF	525	61.45	[PRC1]
FLJ55721AAAF	521	60.72	[FUT10]
FLJ55724AAAF	667	73.28	[NVL]
FLJ55726AAAF	378	43.52	[CHRDL1]
FLJ55729AAAF	738	83.89	[CPT1B]
FLJ55738AAAF	397	43.61	[F7]
FLJ55742AAAF	824	94.4	[PIK3C3]
FLJ55747AAAF	407	46.77	[LPCAT3]
FLJ55749AAAF	862	97.18	[MARCH6]
FLJ55761AAAF	493	56.61	[SEPT4]
FLJ55770AAAF	469	51.73	[MSR1]
FLJ55772AAAF	505	58.53	[RABGEF1]
FLJ55779AAAF	402	44.31	[CCDC149]
FLJ55817AAAF	270	31.19	[KCNMB2]
FLJ55824AAAF	145	16.49	[TMEM216]
FLJ55851AAAF	149	16.05	[SNX21]
FLJ55857AAAF	447	47.81	[NIPAL4]
FLJ55860AAAF	344	39.1	[GRTP1]
FLJ55864AAAF	369	44.08	[TNIP3]
FLJ55875AAAF	625	67.74	[TRIM3]
FLJ55878AAAF	528	59.42	[NAE1]
FLJ55880AAAF	1190	133.75	[ZEB2]
FLJ55884AAAF	1337	147.35	[SCAF8]
FLJ55892AAAF	1137	123.07	[PASK]
FLJ55893AAAF	939	102.79	[KIAA1324]
FLJ55894AAAF	792	88.93	[DLGAP5]
FLJ55900AAAF	1220	139.68	[DHX38]
FLJ55913AAAN	1115	127.14	[STAG3]
FLJ55916SAAF	998	112.42	[GTF2I]
FLJ55956AAAF	519	57.76	[TUBA1C]
FLJ55962AAAF	394	44.58	[ANKS1B]
FLJ55970AAAF	403	45.98	[CYP46A1]

FLJ55972AAAF	656	72.4	[MBD1]
FLJ55974AAAF	883	99.26	[ZC3H12C]
FLJ55978AAAF	741	81	[NDUFS1]
FLJ55980AAAF	252	27.9	[PMEPA1]
FLJ55996AAAF	639	71.21	[COG8]
FLJ55999AAAF	674	76.59	[FBXL5]
FLJ56004AAAF	460	48.39	[FBRS]
FLJ56006AAAF	704	77.46	[ARHGAP22]
FLJ56007AAAF	924	102.98	[CIZ1]
FLJ56010AAAF	359	40.26	[FCGR3A]
FLJ56013AAAF	672	73.92	[NUAK2]
FLJ56019AAAF	468	52.38	[SLC25A23]
FLJ56022AAAF	820	95.35	[EXOC6]
FLJ56035AAAF	894	99.06	[DDR1]
FLJ56059AAAF	537	62.12	[ELMO2]
FLJ56084AAAF	597	69.35	[PPEF1]
FLJ56106AAAF	839	91.65	[FYB]
FLJ56152AAAF	721	81.4	[ARHGEF7]
FLJ56176AAAF	761	84.55	[PAPOLA]
FLJ56178AAAF	414	45.13	[TSPYL4]
FLJ56180AAAF	387	43.92	[NELFE]
FLJ56208AAAN	692	78.23	[SCNN1A]
FLJ56231AAAF	438	50.17	[ZNF2]
FLJ56234AAAF	193	22.13	[EPM2A]
FLJ56238AAAF	376	43.1	[BNIP2]
FLJ56243AAAF	345	37.83	[MAGEA4]
FLJ56246AAAF	946	103.15	[ATP2C2]
FLJ56249AAAF	394	43.36	[SLC35C2]
FLJ56258AAAF	977	111.82	[DSE]
FLJ56266AAAF	518	60.05	[GTPBP4]
FLJ56271AAAF	877	99.05	[COPB2]
FLJ56274AAAF	576	62.88	[TKT]
FLJ56288AAAF	603	65.9	[NUMB]
FLJ56298AAAF	346	35.98	[PYCR1]
FLJ56301AAAN	334	38.23	[CYB5R3]
FLJ56305AAAF	208	23.25	[TMEM222]
FLJ56308AAAF	236	25.77	[LHFPL3]
FLJ56309AAAF	196	22.51	[TEX261]
FLJ56314AAAF	186	19.48	[FAM104A]
FLJ56317AAAF	315	35.81	[ZNF691]
FLJ56318AAAF	311	35.08	[IL20RB]
FLJ56321AAAF	378	43.44	[METTL2B]
FLJ56323AAAF	238	25.05	[MGMT]
FLJ56327AAAF	350	39.61	[PSMD8]
FLJ56330AAAF	284	32.49	[IDI1]
FLJ56332AAAF	349	39.13	[SLC35F1]
FLJ56334AAAF	368	40.75	[SEC13]
FLJ56335AAAF	325	37.57	[CSNK1A1]

FLJ56339AAAF	226	25.04	[SPCS2]
FLJ56347AAAF	357	38.62	[C8orf58]
FLJ56350AAAF	176	19.29	[ISCA1]
FLJ56354AAAF	293	33.09	[MAD2L1BP]
FLJ56357AAAF	307	33.63	[APOA1BP]
FLJ56394AAAF	390	42.04	[NAGK]
FLJ56400AAAF	377	39.42	[GPR6]
FLJ56410AAAF	1049	117.08	[OTUD4]
FLJ56435AAAF	544	62.34	[ANGEL2]
FLJ56444AAAF	1022	115.13	[HERC6]
FLJ56474AAAN	1229	132.78	[HDAC6]
FLJ56490AAAF	413	46.99	[SERPINI2]
FLJ56492AAAF	388	42.18	[TMPRSS5]
FLJ56497AAAF	605	68.28	[ABCC8]
FLJ56501AAAF	396	43.34	[FOXP3]
FLJ56503AAAF	382	42.45	[KIR3DS1]
FLJ56506AAAF	915	102.31	[HK1]
FLJ56521AAAN	905	102.99	[TTF1]
FLJ56527AAAF	376	43.15	[RBM48]
FLJ56575AAAF	733	84.4	[PRKG2]
FLJ56580AAAF	688	76.71	[MEFV]
FLJ56596AAAF	871	99.45	[XPO7]
FLJ56638AAAF	401	45.07	[PEG10]
FLJ56640AAAF	370	42.37	[EED]
FLJ56645AAAF	1249	137.23	[PATJ]
FLJ56679AAAF	154	18.4	[AP1S3]
FLJ56732AAAF	285	31.56	[DMC1]
FLJ56768AAAF	195	21.47	[XG]
FLJ56784AAAF	210	22.81	[BCL7A]
FLJ56788AAAF	149	17.52	[GNN]
FLJ56803AAAF	433	46.4	[C16orf59]
FLJ56847AAAF	619	66.88	[DVL1]
FLJ56849AAAF	814	89.84	[AFAP1]
FLJ56872AAAF	263	29.1	[ZDHHC22]
FLJ56877AAAF	251	27	[NOTO]
FLJ56910AAAF	254	29.03	[ANKRD7]
FLJ56978AAAF	381	43.24	[TCEANC]
FLJ56990AAAF	271	31.15	[ZNF501]
FLJ56991AAAF	430	49.31	[ZNF557]
FLJ56997AAAF	765	84.11	[EMR2]
FLJ57010AAAF	316	35.44	[KNSTRN]
FLJ57016AAAF	412	46.79	[ZNF821]
FLJ57026AAAF	997	107.01	[GPR113]
FLJ57053AAAF	456	51.72	[ZC2HC1C]
FLJ57068AAAF	199	22.28	[FGF13]
FLJ57090AAAF	222	24.15	[TNFSF13]
FLJ57126AAAF	328	38.21	[ELMOD1]
FLJ57145AAAF	380	42.45	[GDA]

FLJ57181AAAF	340	37.69	[FPGT]
FLJ57191AAAF	286	33	[SEPT3]
FLJ57218AAAF	362	40.16	[PTK2]
FLJ57230AAAF	565	62.82	[CDC14A]
FLJ57231SAAN	215	23.43	[SPACA3]
FLJ57289AAAF	255	29.18	[CSNK2A1]
FLJ57338AAAF	693	79.08	[UGT2A1]
FLJ57340AAAF	401	45.54	[KIAA0513]
FLJ57342AAAF	1010	110.63	[LAMC2]
FLJ57350AAAF	317	36.84	[PPP1R7]
FLJ57417AAAF	301	34.82	[NDUFAF6]
FLJ57430AAAF	424	47.45	[POLR2C]
FLJ57433AAAF	199	20.77	[MS4A15]
FLJ57445AAAF	174	19.56	[CD302]
FLJ57446AAAN	302	34.77	[GNAI1]
FLJ57452AAAF	194	21.82	[NUDT16]
FLJ57490AAAF	215	24.09	[CLCF1]
FLJ57496AAAF	129	15.79	[TBCA]
FLJ57504AAAF	290	33.18	[LAPTM5]
FLJ57515AAAF	306	34.59	[CABLES1]
FLJ57525AAAN	763	84.89	[CDC27]
FLJ57537AAAF	257	27.88	[B9D1]
FLJ57595AAAF	120	13.47	[PNRC2]
FLJ57630AAAF	155	18.1	[MRPL20]
FLJ57726AAAF	238	24.88	[HNRNPH3]
FLJ57765AAAF	175	19.74	[CD8B]
FLJ57819AAAF	300	33.29	[SLC25A27]
FLJ57847AAAF	587	65.92	[MAPK4]
FLJ57866SAAN	310	34.26	[TAF8]
FLJ57894AAAF	337	38.86	[HFE]
FLJ57906AAAF	253	29.43	[HVCN1]
FLJ57996AAAF	443	48.88	[PKNOX2]
FLJ58001AAAF	294	33.82	[SNX6]
FLJ58085AAAF	479	52.49	[SIGLEC9]
FLJ58115AAAN	572	61.64	[FUBP3]
FLJ58116AAAF	567	62.01	[SRPK3]
FLJ58120AAAF	395	43.52	[SS18]
FLJ58124AAAF	591	66.7	[CFI]
FLJ58190AAAF	319	35.18	[C1QTNF3]
FLJ58193AAAF	1450	161.16	[SSH2]
FLJ58196AAAF	756	82.77	[ZC3H11A]
FLJ58236AAAF	920	105.82	[USP28]
FLJ58277AAAF	344	38.58	[SHISA9]
FLJ58305AAAF	355	39.87	[KLHDC3]
FLJ58314AAAF	348	40.42	[MAPK1]
FLJ58322SAAF	512	58.13	[PPP3CC]
FLJ58346AAAF	388	42.82	[MXD3]
FLJ58465SAAF	530	59.38	[RBM39]

FLJ58470AAAF	416	45.29	[SS18L1]
FLJ58579AAAF	407	47.52	[ZFP1]
FLJ58593AAAN	600	66.51	[WDR20]
FLJ58632AAAF	545	61.5	[DTNB]
FLJ58645AAAF	434	48.88	[TGFBR1]
FLJ58700AAAF	348	40.31	[TRIM16L]
FLJ58711WAAF	1132	120.33	[NUTM1]
FLJ58713AAAF	386	42.46	[RIMKLB]
FLJ58725AAAF	899	96.16	[ANKRD28]
FLJ58728AAAF	780	87.46	[MARK1]
FLJ58731AAAF	1077	122.48	[NCKAP1L]
FLJ58759AAAF	384	42.27	[ZIC4]
FLJ58842AAAF	209	21.79	[ZNRF1]
FLJ58926AAAF	243	27.1	[CDK20]
FLJ58957AAAF	128	15.06	[C6orf201]
FLJ58961AAAF	259	29.25	[APIP]
FLJ58984AAAF	392	44.27	[TDP2]
FLJ58995AAAF	327	36.24	[CD1B]
FLJ59004AAAF	281	32.01	[HLA-DQB1]
FLJ59013AAAF	125	13.53	[LOC100287036]
FLJ59015AAAF	312	34.35	[MRGPRE]
FLJ59016AAAF	277	29.96	[CLEC18B]
FLJ59027AAAF	410	44.86	[HAUS8]
FLJ59032AAAF	143	15.99	[PINLYP]
FLJ59060AAAF	279	30.68	[TARM1]
FLJ59062AAAF	290	33	[ARL13A]
FLJ59065AAAF	432	50.02	[SEPT14]
FLJ59087AAAF	288	31.5	[LOC100134391]
FLJ59152AAAF	772	90.24	[CDK11B]
FLJ59190AAAF	253	28.25	[TSPAN11]
FLJ59209AAAF	887	102.66	[ACTN1]
FLJ59210AAAF	578	64.85	[TBCE]
FLJ59211AAAF	535	60.13	[PRKCSH]
FLJ59212AAAF	738	83.57	[ECE1]
FLJ59215AAAF	518	58.29	[HSF2]
FLJ59216AAAF	664	74.52	[ALOX15B]
FLJ59223AAAF	587	65.16	[REL]
FLJ59224AAAF	410	46.42	[ANGPT4]
FLJ59226AAAF	426	47.47	[KLF5]
FLJ59232AAAF	770	85.45	[GIT1]
FLJ59236AAAF	507	54.84	[ABI2]
FLJ59237AAAF	659	74.53	[IL12RB2]
FLJ59241AAAF	755	81.71	[TLE1]
FLJ59248AAAF	470	53.48	[CYP20A1]
FLJ59249AAAF	570	63.66	[ALCAM]
FLJ59268AAAF	366	40.74	[TACR2]
FLJ59277AAAF	695	75.29	[CAST]
FLJ59321AAAF	1098	125.57	[PLEKHA5]

FLJ59337AAAF	670	75.19	[APPL2]
FLJ59437AAAF	445	49.93	[STARD3]
FLJ59484AAAF	859	85.25	[POM121]
FLJ59497AAAF	845	92.87	[SPIDR]
FLJ59553AAAF	1072	117.76	[KIAA0319]
FLJ59573AAAF	247	28.18	[ZNF471]
FLJ59588AAAF	176	19.81	[C10orf131]
FLJ59590AAAF	130	13.76	[C10orf142]
FLJ59637AAAF	307	34.94	[SLC25A52]
FLJ59638AAAF	355	40.78	[ATXN3L]
FLJ59640AAAF	331	34.84	[FAM110C]
FLJ59653AAAF	465	50.28	[LPP]
FLJ59660AAAF	364	41.82	[CASP5]
FLJ59682SAAN	824	91.76	[NPAS2]
FLJ59760AAAF	661	75.85	[GBE1]
FLJ59765AAAF	385	44.97	[ADAP1]
FLJ59811AAAF	360	40.36	[PNCK]
FLJ59817WAAN	622	69.23	[SOX13]
FLJ59880AAAF	247	28.88	[TK2]
FLJ60013AAAF	144	16.65	[FAM153C]
FLJ60016AAAF	300	33.15	[LPPR5]
FLJ60019AAAF	244	26.98	[RNASE10]
FLJ60051AAAF	176	20.85	[TMEM97]
FLJ60060AAAN	514	58.67	[ORC2]
FLJ60076WAAF	326	36.09	[ELAVL1]
FLJ60135SAAF	369	40.75	[SLC30A8]
FLJ60402AAAN	1165	129.24	[EGF]
FLJ60536AAAN	1430	160.06	[DAPK1]
FLJ60690SAAF	441	49.32	[DCX]
FLJ60895AAAF	183	20.2	[PSMF1]
FLJ60927AAAN	561	57.23	[MUC1]
FLJ61100SAAN	306	35.24	[MRPL45]
FLJ61177SAAF	668	73.23	[PIP5K1C]
FLJ61234AAAN	1833	194	[TNRC6B]
FLJ61493AAAN	1321	146.21	[SPAG9]
FLJ61615SAAN	485	55.31	[TRAF3]
FLJ61659SAAF	796	89.84	[DDX27]
FLJ61704AAAN	1104	118.55	[UBN1]
FLJ75001AAAF	629	71.98	[EFCAB7]
FLJ75006AAAF	437	46.34	[CD177]
FLJ75017AAAF	266	30.03	[HLA-DRB1]
FLJ75031AAAF	374	43.02	[TRAM1]
FLJ75041AAAF	405	46.17	[ZNF785]
FLJ75042AAAF	188	20.78	[TRAPPC5]
FLJ75044AAAF	649	70.95	[RAB11FIP1]
FLJ75049AAAF	235	25.06	[FAM168A]
FLJ75053AAAF	778	88.03	[ACAP2]
FLJ75055AAAF	456	51.07	[TLDC1]

FLJ75067AAAF	300	33.66	[HSD17B13]
FLJ75068AAAF	392	44.06	[SERPINB11]
FLJ75078AAAF	301	31.91	[CGREF1]
FLJ75086AAAF	421	47.35	[CPA4]
FLJ75088AAAF	524	58.04	[PAK2]
FLJ75089AAAF	889	96.86	[FCHO1]
FLJ75090AAAF	582	62.3	[MNT]
FLJ75092AAAF	723	78.26	[GGA3]
FLJ75101AAAF	453	48.76	[DLST]
FLJ75102AAAF	440	46.29	[WIPF2]
FLJ75103AAAF	808	90.33	[PTPDC1]
FLJ75108AAAF	271	28.84	[TMEM150A]
FLJ75121AAAF	820	91.58	[FGFR1]
FLJ75128AAAF	313	35.07	[SLC35E3]
FLJ75130AAAF	529	57.26	[HSF1]
FLJ75131AAAF	940	106.27	[GALNT5]
FLJ75132AAAF	95	10.2	[LINC00526]
FLJ75135AAAF	899	96.68	[NFKB2]
FLJ75142AAAF	254	28.71	[SPATA9]
FLJ75144AAAN	628	68.75	[ASH2L]
FLJ75145AAAF	146	16.39	[MCFD2]
FLJ75150AAAF	565	62.66	[USP21]
FLJ75159AAAF	690	77.15	[HDX]
FLJ75165AAAF	211	23.39	[BAK1]
FLJ75169AAAF	260	29.78	[UVSSA]
FLJ75172AAAF	315	36.42	[PGAP2]
FLJ75190AAAN	1068	124.28	[PIK3CA]
FLJ75191AAAF	227	25.37	[RND2]
FLJ75193AAAF	489	55.32	[CAMK2A]
FLJ75194AAAF	384	42.19	[SPPL3]
FLJ75198AAAF	501	55.86	[TRAF2]
FLJ75202AAAF	454	50.34	[RARG]
FLJ75212AAAF	392	43.12	[BCKDHB]
FLJ75219AAAF	419	48.25	[RASSF8]
FLJ75225AAAF	288	33.05	[CD80]
FLJ75234AAAF	324	34.65	[SLC35A4]
FLJ75237AAAF	530	61.01	[UGT2B15]
FLJ75243AAAF	298	33.02	[SLC25A21]
FLJ75244AAAF	916	104.46	[ERCC4]
FLJ75246AAAF	400	45.59	[RRAGD]
FLJ75248AAAF	417	46.85	[PPP4R2]
FLJ75254AAAF	382	43.33	[PABPC5]
FLJ75261AAAF	375	39.8	[ADH1A]
FLJ75262AAAF	450	51.81	[PDE7B]
FLJ75263AAAF	599	66.12	[IGF2BP2]
FLJ75267AAAF	555	62.36	[NRP2]
FLJ75269AAAF	375	42.75	[MSTN]
FLJ75272AAAF	316	35.68	[NUDT6]

FLJ75274AAAF	225	25.4	[KLRB1]
FLJ75277AAAF	156	17.47	[RNASE7]
FLJ75279AAAN	621	68.36	[ELL]
FLJ75281AAAF	462	52.15	[GABRA5]
FLJ75289AAAF	311	34.01	[SLC25A1]
FLJ75294AAAF	461	52.98	[CALCRL]
FLJ75296AAAF	453	51.51	[FGG]
FLJ75300AAAF	434	50.14	[TRNT1]
FLJ75303AAAF	491	55.98	[KATNA1]
FLJ75307AAAF	648	73.58	[KIAA0020]
FLJ75309AAAF	411	46.87	[EIF4A3]
FLJ75317AAAF	542	60.39	[CAMK2B]
FLJ75318AAAF	789	84.73	[LRFN2]
FLJ75323AAAN	676	75.12	[PROS1]
FLJ75326AAAF	363	42.01	[TECRL]
FLJ75327AAAF	407	46.56	[ZNF365]
FLJ75329AAAF	380	44.85	[CERS2]
FLJ75330AAAN	780	90.96	[CUL5]
FLJ75345AAAF	474	54.61	[GABRB2]
FLJ75350AAAF	560	62.67	[HABP2]
FLJ75354AAAF	327	35.25	[HOGA1]
FLJ75359AAAF	416	46.27	[TMPRSS11B]
FLJ75367AAAF	528	60.51	[UGT2B4]
FLJ75384AAAF	195	22.14	[RWDD3]
FLJ75386AAAF	329	36.36	[KCTD13]
FLJ75387AAAF	459	51.22	[RNF25]
FLJ75390AAAF	253	27.52	[KLK7]
FLJ75394AAAF	360	41.06	[CCR2]
FLJ75395AAAF	313	33.53	[MYEOV]
FLJ75397AAAF	440	50.64	[GABRP]
FLJ75400AAAF	245	27.87	[KITLG]
FLJ75404AAAF	417	45.38	[PVR]
FLJ75413AAAF	318	34.78	[SGCB]
FLJ75415AAAF	420	48.69	[NPFFR2]
FLJ75418AAAF	901	104.22	[OCRL]
FLJ75422AAAF	286	32.91	[CAPZA1]
FLJ75423AAAF	159	17.36	[AGTRAP]
FLJ75427AAAF	357	39.74	[ANXA13]
FLJ75428AAAN	670	74.59	[ATF6]
FLJ75436AAAF	240	26.35	[TNFSF14]
FLJ75441AAAF	396	45.45	[VRK1]
FLJ75444AAAF	404	45.5	[PRKAR2A]
FLJ75450AAAF	621	69.73	[FXR1]
FLJ75456AAAN	732	82.66	[XRCC5]
FLJ75458AAAF	604	66.82	[QSOX1]
FLJ75459AAAF	392	43.45	[TIAL1]
FLJ75466AAAF	418	47.08	[RASSF5]
FLJ75471AAAF	419	46.55	[UHMK1]

FLJ75474AAAF	343	39.42	[HOMER2]
FLJ75480AAAF	711	78.34	[LTF]
FLJ75497AAAF	750	85.51	[MME]
FLJ75506AAAN	859	90.81	[PHC2]
FLJ75540AAAN	661	75.52	[F13B]
FLJ75548AAAF	173	19.63	[MFAP5]
FLJ75552AAAF	175	19.68	[C15orf62]
FLJ75554AAAF	248	27.6	[IAH1]
FLJ75559AAAF	541	59.67	[CCT5]
FLJ75562AAAF	326	37.36	[AKR1D1]
FLJ75573AAAF	403	46.4	[SNX32]
FLJ75574AAAF	176	20.28	[PNOC]
FLJ75579AAAF	484	54.53	[CACNB3]
FLJ75580AAAF	570	65.34	[CYBB]
FLJ75582AAAF	451	50.66	[CDK14]
FLJ75583AAAF	456	51.8	[GABRA1]
FLJ75587AAAF	379	40.91	[FGFR1OP]
FLJ75588AAAF	593	68.01	[PTPN11]
FLJ75589AAAF	1128	126.76	[MSH3]
FLJ75593AAAF	563	63.53	[GRK1]
FLJ75594AAAF	337	35.82	[HEY2]
FLJ75595AAAF	444	50.97	[PACSIN1]
FLJ75596AAAF	736	83.34	[PDE4B]
FLJ75602AAAF	528	59.86	[ASIC1]
FLJ75607AAAF	395	44.03	[CBWD2]
FLJ75608AAAF	299	33.25	[RGN]
FLJ75609AAAF	290	33.37	[ATP1B2]
FLJ75611AAAF	647	72.61	[LIMK1]
FLJ75614AAAN	683	77.83	[PRKCH]
FLJ75619AAAF	418	46.17	[SLC25A46]
FLJ75622AAAF	793	89.7	[PTPRA]
FLJ75625AAAF	262	29.46	[METTL20]
FLJ75627AAAF	425	48.3	[ABHD2]
FLJ75628AAAF	943	103.97	[CLCA2]
FLJ75632AAAF	595	68.57	[P2RX7]
FLJ75633AAAF	642	72.41	[SCFD1]
FLJ75637AAAF	334	38.7	[SUCNR1]
FLJ75638AAAF	490	55.98	[IFIT3]
FLJ75641AAAF	512	58.62	[LYN]
FLJ75646AAAF	378	43.33	[IL3RA]
FLJ75652AAAF	291	32.38	[SGCG]
FLJ75660AAAF	324	34.91	[TMEM165]
FLJ75672AAAF	304	33.7	[RNF115]
FLJ75673AAAF	372	40.56	[RABEPK]
FLJ75674AAAF	338	35.5	[MDH2]
FLJ75678AAAF	180	19.9	[IL1RN]
FLJ75681AAAF	259	29.28	[UBXN2A]
FLJ75686AAAF	144	16.08	[PLA2G2A]

FLJ75690AAAF	575	61.59	[BAG3]
FLJ75694AAAN	395	43.3	[PTGDR2]
FLJ75695AAAF	342	37.77	[GALM]
FLJ75704AAAN	345	36.11	[CEBPB]
FLJ75705AAAF	359	41.06	[AGTR1]
FLJ75712AAAF	373	42.25	[HSD3B1]
FLJ75716AAAF	454	49.87	[PPM1F]
FLJ75718AAAF	251	28.09	[TNFSF15]
FLJ75720AAAF	458	51.31	[ESRRG]
FLJ75721AAAF	410	46.03	[COX15]
FLJ75731AAAF	1124	125.8	[TEK]
FLJ75732AAAF	482	54.12	[G3BP2]
FLJ75733AAAF	277	30.71	[COLEC10]
FLJ75736AAAF	212	24.43	[EDN1]
FLJ75737AAAF	441	50.32	[ETS1]
FLJ75742AAAF	296	33.43	[BLVRA]
FLJ75750AAAF	390	44.34	[TGFB1]
FLJ75752AAAF	320	33.64	[PYCR2]
FLJ75753AAAN	509	58.83	[PRIM2]
FLJ75755AAAF	413	47.07	[PRSS35]
FLJ75760AAAF	353	40.49	[BDKRB1]
FLJ75786AAAF	338	36.81	[FBP1]
FLJ75790AAAF	267	30.78	[APOA1]
FLJ75794AAAF	400	44.2	[DRD3]
FLJ75796AAAF	425	48.05	[RBM34]
FLJ75797AAAF	338	38.68	[ZPBP2]
FLJ75800AAAF	487	54.37	[NAB1]
FLJ75805AAAF	536	58.71	[FANCE]
FLJ75806AAAF	320	36.46	[IMPACT]
FLJ75808AAAN	415	48.47	[PTPN2]
FLJ75809AAAF	358	41.33	[GDAP1]
FLJ75811AAAF	201	24	[DPT]
FLJ75815AAAF	185	20.81	[TGIF2LY]
FLJ75818AAAF	352	39.22	[SETD8]
FLJ75821AAAN	360	41.4	[CCR4]
FLJ75822AAAF	153	17.03	[KIAA0040]
FLJ75824AAAF	328	34.63	[SDS]
FLJ75826AAAF	167	19.46	[UFC1]
FLJ75828AAAF	370	42.85	[PDGFD]
FLJ75835AAAF	186	21.1	[ARL6]
FLJ75838AAAF	367	40.33	[WISP1]
FLJ75841AAAF	515	57.76	[IFNAR2]
FLJ75842AAAF	272	30.16	[SYPL2]
FLJ75843AAAF	425	47.24	[PLEKHA2]
FLJ75847AAAF	543	60.69	[GPR108]
FLJ75850AAAF	690	79.54	[CNGA1]
FLJ75852AAAN	585	66.85	[APPBP2]
FLJ75858AAAF	519	59.05	[DCT]

FLJ75865AAAF	522	59.57	[TRAF6]
FLJ75866AAAF	412	46.22	[CHRFAM7A]
FLJ75867AAAF	236	26.42	[RABL3]
FLJ75868AAAF	400	46.21	[CSF2RA]
FLJ75870AAAF	507	51.78	[NOVA1]
FLJ75872AAAF	535	61.48	[MGAT4A]
FLJ75874AAAN	780	85.82	[AMOTL2]
FLJ75881AAAN	760	84.87	[TFRC]
FLJ75891AAAF	541	59.57	[CAMKK2]
FLJ75893AAAF	416	46.34	[CASP9]
FLJ75906AAAF	230	25.53	[RNF141]
FLJ75907AAAF	323	35.21	[CD47]
FLJ75909AAAF	385	42.88	[PNKD]
FLJ75911AAAF	245	27.63	[C12orf60]
FLJ75914AAAF	441	51.73	[LRRC17]
FLJ75920AAAF	198	23.37	[FCF1]
FLJ75924AAAF	313	34.03	[FCN2]
FLJ75927AAAF	352	41.55	[CHST11]
FLJ75929AAAF	426	46.88	[SYT13]
FLJ75932AAAF	257	28.46	[APH1B]
FLJ75937AAAF	233	26.25	[BCL10]
FLJ75938AAAF	402	44.24	[KLF12]
FLJ75939AAAF	422	46.09	[HTR1A]
FLJ75940AAAF	326	37.79	[RASSF2]
FLJ75941AAAF	306	34.54	[LYSMD3]
FLJ75942AAAF	336	39.13	[CDC123]
FLJ75944AAAF	342	37.66	[C9orf91]
FLJ75945AAAF	353	37.46	[HNRNPA2B1]
FLJ75954AAAF	432	47.02	[CADM3]
FLJ75955AAAF	288	31.22	[SPRY3]
FLJ75957AAAF	289	32.78	[BTLA]
FLJ75958AAAF	198	22.15	[DNAJC5]
FLJ75961AAAF	231	26.56	[PXDC1]
FLJ75975AAAF	460	53.65	[CEP57L1]
FLJ75978AAAF	354	39.94	[OTC]
FLJ75980AAAF	263	29.46	[RPS4Y1]
FLJ75981AAAF	359	40.05	[PTGFR]
FLJ75982AAAF	310	36.16	[B3GALT5]
FLJ75986AAAF	312	36.38	[FGL1]
FLJ75987AAAF	179	20.68	[ZBTB8OS]
FLJ75992AAAF	949	100.06	[ZCCHC14]
FLJ75994AAAF	224	25.27	[FNDC9]
FLJ75997AAAF	316	34.83	[GTF2B]
FLJ76001AAAF	220	23.78	[LHFPL1]
FLJ76009AAAF	253	28.48	[MARCH3]
FLJ76014AAAF	314	34.06	[SLC25A11]
FLJ76015AAAF	438	48.13	[GCDH]
FLJ76017AAAF	314	35.05	[MAGEA2]

FLJ76023AAAF	322	36.64	[EPYC]
FLJ76024AAAF	284	29.65	[PHOX2A]
FLJ76027AAAF	259	29.97	[KCTD4]
FLJ76032AAAF	391	44.78	[KCNJ1]
FLJ76034AAAF	395	43.63	[MAT1A]
FLJ76037AAAF	486	52.84	[MATN3]
FLJ76038AAAF	238	27.12	[CD300LB]
FLJ76043AAAF	726	80.59	[CCNT1]
FLJ76044AAAF	224	26.15	[SPC25]
FLJ76046AAAF	302	32.82	[ECI1]
FLJ76051AAAF	393	43.44	[MAP2K1]
FLJ76062AAAF	820	92.76	[ADAM29]
FLJ76076AAAN	300	34.33	[ARPC2]
FLJ76080AAAF	669	76.75	[CAPN13]
FLJ76090AAAF	380	42.34	[WNT5A]
FLJ76107WAAF	255	27.32	[DLX1]
FLJ76121AAAF	902	101.46	[ZC3HAV1]
FLJ76143AAAF	552	60.44	[SMAD4]
FLJ76158AAAN	291	32.84	[MBD3]
FLJ76162AAAF	429	46.3	[ZBP1]
FLJ76167AAAF	308	33.07	[NXPH4]
FLJ76193AAAN	727	82.66	[RHOBTB2]
FLJ76194AAAF	984	109.89	[EPHB1]
FLJ76198AAAF	390	44.37	[ALG3]
FLJ76201AAAF	440	48.12	[PDIA6]
FLJ76205AAAF	257	28.02	[RPL8]
FLJ76206AAAF	269	29.38	[GAMT]
FLJ76209AAAF	274	31.58	[PPA2]
FLJ76214AAAF	834	95.7	[RASA3]
FLJ76219AAAF	441	50.24	[IP6K1]
FLJ76224AAAF	573	62.66	[EYA3]
FLJ76225AAAF	253	27.98	[PNPLA4]
FLJ76231AAAF	380	41.8	[ACOT7]
FLJ76238AAAF	963	109.93	[SART3]
FLJ76240AAAF	443	48.98	[ERICH1]
FLJ76242AAAF	323	35.94	[LGALS4]
FLJ76243AAAF	418	46.26	[TMPRSS11D]
FLJ76248AAAF	441	50.03	[PLA2G7]
FLJ76251AAAF	281	31.84	[E2F6]
FLJ76253AAAN	484	53.94	[ETV4]
FLJ76256AAAF	208	23.56	[RTN1]
FLJ76258AAAF	318	35.2	[UBE2J1]
FLJ76259AAAF	403	43.95	[AMT]
FLJ76286AAAN	768	88.93	[CUL3]
FLJ76302AAAF	391	44.6	[TRDMT1]
FLJ76306AAAF	728	82.97	[HGF]
FLJ76307AAAF	1108	127.06	[MYO1E]
FLJ76308AAAN	861	97.38	[ORC1]

FLJ76309AAAF	913	104.04	[USP26]
FLJ76318AAAF	1186	126.47	[PUM1]
FLJ76330AAAN	532	60.7	[FBXW8]
FLJ76337AAAF	468	52.23	[PPARA]
FLJ76349AAAF	246	29.45	[SNRNP35]
FLJ76355AAAF	353	40.68	[GPR139]
FLJ76359AAAF	266	29.83	[HLA-DRB5]
FLJ76369AAAN	118	12.89	[VAMP1]
FLJ76387AAAF	221	25.51	[SRSF9]
FLJ76398AAAF	219	24.33	[NUDT5]
FLJ76399AAAF	203	23.33	[ARL6IP1]
FLJ76402AAAF	216	23.74	[PPIB]
FLJ76408AAAF	160	18.12	[TNNC2]
FLJ76411AAAF	199	22.39	[TAGLN2]
FLJ76422AAAF	109	12.11	[BAGE2]
FLJ76425AAAF	68	7.4	[ATOX1]
FLJ76426AAAF	275	30.7	[ICAM2]
FLJ76427AAAN	1173	133.53	[CTR9]
FLJ76428AAAF	442	51.47	[MIPOL1]
FLJ76437AAAF	502	56.86	[CDK19]
FLJ76455AAAF	695	78.3	[FSHR]
FLJ76457AAAF	686	75.54	[LCMT2]
FLJ76468AAAN	1698	191.16	[CUL7]
FLJ76492AAAF	502	56.45	[CHRNA7]
FLJ76500AAAF	348	37.05	[ZNF843]
FLJ76501AAAF	374	43.01	[FUT5]
FLJ76504AAAF	87	10.74	[MRPS21]
FLJ76514AAAF	86	10.11	[SRP9]
FLJ76535AAAF	160	17.78	[SFT2D2]
FLJ76594AAAF	246	28.38	[LIN37]
FLJ76620AAAF	244	26.69	[EMG1]
FLJ76630AAAF	293	35.22	[MSMO1]
FLJ76631AAAF	154	16.83	[EIF5A]
FLJ76634AAAF	236	26.23	[YIPF6]
FLJ76639AAAF	406	42.43	[RING1]
FLJ76641AAAF	424	49.03	[CSNK1G3]
FLJ76651AAAF	757	84.18	[PPM1E]
FLJ76652AAAN	1399	152.35	[NCOA1]
FLJ76663AAAF	1298	145.59	[FLT4]
FLJ76675AAAF	850	96.55	[RASA2]
FLJ76677AAAF	378	40.97	[FOXI1]
FLJ76684AAAF	439	50.22	[TERF1]
FLJ76705AAAN	1068	120.85	[RBL1]
FLJ76731SAAN	825	91.77	[PRDM1]
FLJ76769AAAN	555	62.75	[GPC6]
FLJ76778AAAF	1333	152.46	[SOS1]
FLJ76788AAAN	895	100.16	[SF3B2]
FLJ76790AAAN	963	108.15	[INTS4]

FLJ76791AAAF	338	39.11	[DFFB]
FLJ76833AAAF	796	87.95	[CDH11]
FLJ76841AAAF	520	58.13	[MITF]
FLJ76851AAAF	430	46.65	[PBX1]
FLJ76877AAAF	1042	117.9	[SKIV2L2]
FLJ76902AAAF	381	43.23	[NFKBIL1]
FLJ76913AAAF	522	58.5	[FBXO7]
FLJ76917AAAF	811	94.6	[TLR10]
FLJ76920AAAF	536	62.24	[CARD9]
FLJ76942AAAF	1242	147.62	[SFI1]
FLJ76944AAAN	1172	129.99	[THBS2]
FLJ76948AAAN	871	100.18	[ZNF473]
FLJ76954AAAF	579	62.33	[YTHDF2]
FLJ76965AAAF	540	61.04	[PIP5K1B]
FLJ76966AAAN	528	60.77	[UGT2B10]
FLJ76977AAAF	939	107.11	[MORC3]
FLJ77005AAAN	1424	155.27	[NCOA3]
FLJ77008AAAF	647	71.41	[WHSC1]
FLJ77009AAAF	598	65.71	[CACNB1]
FLJ77011AAAN	407	46.25	[TACR1]
FLJ77029AAAF	418	46.17	[RAD52]
FLJ77063AAAF	425	47.52	[HCRTR1]
FLJ77064AAAF	236	25.45	[ASCL1]
FLJ77070AAAF	288	34.59	[TNNT2]
FLJ77087AAAN	208	23.44	[FGF9]
FLJ77091AAAF	380	40.22	[ADH4]
FLJ77106AAAF	335	37.73	[FAS]
FLJ77107SAAN	521	59.71	[VANGL2]
FLJ77109AAAN	870	96.7	[ADAM22]
FLJ77122AAAF	254	27.98	[MEOX1]
FLJ77129AAAF	350	40.21	[TWF1]
FLJ77133AAAF	484	53.46	[PAX3]
FLJ77165AAAF	168	18.97	[VIP]
FLJ77184AAAF	495	55.48	[ESR2]
FLJ77200AAAN	461	51.78	[F9]
FLJ77203AAAF	421	45.9	[RBMS3]
FLJ77223AAAN	839	95.68	[TLR4]
FLJ77245AAAN	1028	117.95	[MYO1C]
FLJ77247AAAN	1001	112.37	[TOP3A]
FLJ77250AAAF	1262	144.5	[IARS]
FLJ77251AAAN	466	51.49	[ADRA1A]
FLJ77255AAAN	1290	136.19	[PER1]
FLJ77258AAAF	983	110.16	[EPHA3]
FLJ77262AAAN	453	51.02	[GABRA6]
FLJ77269AAAF	661	74.28	[NUAK1]
FLJ77275AAAN	975	110.75	[BICD1]
FLJ77278AAAN	466	51.72	[CHRM2]
FLJ77284AAAN	500	56.93	[CEP57]

FLJ77337AAAN	292	33.46	[TAZ]
FLJ77346AAAF	483	54.28	[DDX49]
FLJ77377AAAF	559	59.88	[PUF60]
FLJ77399AAAF	1454	160.62	[MED14]
FLJ77417AAAF	461	51.1	[NR1H2]
FLJ77457AAAN	1049	119.28	[TAOK2]
FLJ77491AAAN	896	102.49	[LEPR]
FLJ77516AAAN	165	18.03	[LMO4]
FLJ77531AAAN	1647	184.65	[SMARCA4]
FLJ77533AAAN	926	103.89	[SIK2]
FLJ77543AAAF	603	67.33	[NR2C1]
FLJ77551AAAF	852	97.97	[UBE3A]
FLJ77556AAAF	730	81.03	[CCNT2]
FLJ77562AAAF	498	55.65	[STEAP3]
FLJ77569SAAN	514	55.96	[WT1]
FLJ77571AAAF	724	80.57	[LARP4]
FLJ77587AAAF	688	79.63	[ADRBK2]
FLJ77597AAAN	808	92.12	[ANAPC4]
FLJ77602AAAF	977	109.65	[ERN1]
FLJ77603AAAF	724	79.31	[DDX4]
FLJ77754AAAF	483	53.75	[KRT8]
FLJ77793AAAF	395	42.87	[DHODH]
FLJ77868AAAF	375	41.86	[PDGFRL]
FLJ77890AAAN	765	90.75	[TOP1]
FLJ77990AAAN	666	76.34	[FZD3]
FLJ78181AAAF	323	36.85	[AKR1C3]
FLJ78372SAAN	495	54.25	[A1BG]
FLJ78424AAAN	1173	133.34	[UBE4B]
FLJ78639AAAN	534	57.33	[CD276]
FLJ78656AAAF	1128	128.79	[NCKAP1]
FLJ78672AAAN	846	95.3	[CLOCK]
FLJ78705AAAN	1011	116.54	[OFD1]
FLJ78733AAAF	951	105.63	[AP2B1]
FLJ78752AAAN	1338	153.03	[CENPJ]
FLJ78759AAAN	1541	170.8	[MADD]
FLJ79253AAAF	488	52.05	[CELF2]
FLJ80001AAAF	233	25.64	[TNF]
FLJ80002AAAF	241	27.28	[PDGFB]
FLJ80003AAAF	252	28.48	[OSM]
FLJ80004AAAF	206	23.39	[PIGF]
FLJ80005AAAF	369	40.97	[DHPS]
FLJ80006AAAF	297	33.58	[PIGC]
FLJ80007AAAF	188	21.08	[PIGH]
FLJ80010AAAN	760	84.08	[PIGQ]
FLJ80012AAAF	931	105.79	[PIGN]
FLJ80013SAAF	84	9.3	[DPM2]
FLJ80015AAAF	484	54.13	[PIGA]
FLJ80016AAAF	212	23.86	[MRPL48]

FLJ80017AAAF	505	54.83	[WASL]
FLJ80018AAAF	482	53.48	[RPS6KB2]
FLJ80019AAAF	505	56.81	[ACVR1B]
FLJ80020AAAF	164	18.12	[CDKN1A]
FLJ80021AAAF	344	39.28	[AURKB]
FLJ80022AAAF	857	97.07	[TTK]
FLJ80023AAAF	403	45.81	[AURKA]
FLJ80026AAAF	382	42.99	[MAPKAPK3]
FLJ80027AAAF	372	42.78	[CDK9]
FLJ80029AAAF	733	83.24	[RPS6KA2]
FLJ80030AAAF	603	68.25	[PLK1]
FLJ80031AAAF	606	67.59	[ARAF]
FLJ80032AAAF	406	46.43	[PHKG2]
FLJ80035AAAF	196	21.6	[HSPB8]
FLJ80036AAAF	465	51.34	[MKNK1]
FLJ80037AAAF	898	105.41	[TAOK3]
FLJ80038AAAF	359	41.22	[SKAP2]
FLJ80039AAAN	298	33.93	[CDK2]
FLJ80041AAAF	349	39.55	[TWF2]
FLJ80042AAAF	415	47.33	[CSNK1D]
FLJ80043AAAF	303	33.73	[CDK4]
FLJ80044AAAF	476	54.42	[CHEK1]
FLJ80045AAAF	543	60.91	[CHEK2]
FLJ80046AAAF	365	42.09	[MAPK13]
FLJ80047AAAF	835	88.55	[TRIM28]
FLJ80048AAAF	292	33.3	[CDK5]
FLJ80050AAAF	407	46.15	[PDK2]
FLJ80053AAAF	412	46.36	[BCKDK]
FLJ80054AAAF	505	57.71	[BLK]
FLJ80055AAAF	435	49	[STK40]
FLJ80056AAAF	426	48.11	[STK25]
FLJ80057AAAF	433	48.64	[STK11]
FLJ80058AAAF	592	67.66	[PRKCZ]
FLJ80060AAAF	527	58.02	[OXSR1]
FLJ80061AAAF	350	41.21	[CSNK2A2]
FLJ80062AAAF	576	65.95	[GRK6]
FLJ80064AAAF	491	56.3	[STK3]
FLJ80066AAAF	591	64.07	[PAK4]
FLJ80069AAAF	549	61.1	[FASTK]
FLJ80072AAAF	465	54.19	[STK38]
FLJ80073AAAF	556	63.15	[PDPK1]
FLJ80075AAAF	313	35.71	[NEK6]
FLJ80076AAAF	505	58.25	[FRK]
FLJ80077AAAF	544	60.07	[ADCK4]
FLJ80078AAAF	539	61.19	[LCK]
FLJ80079AAAF	685	78.24	[PLK2]
FLJ80081AAAF	379	43.14	[MAPK3]
FLJ80083AAAF	1187	133.67	[TYK2]

FLJ80084AAAF	297	34.1	[CDK1]
FLJ80085AAAF	273	30.33	[TSSK6]
FLJ80086AAAF	688	76.74	[SCYL3]
FLJ80089WAAF	537	60.76	[FYN]
FLJ80091AAAF	568	63.96	[DYRK3]
FLJ80092AAAF	496	55.72	[CDK16]
FLJ80093AAAF	367	41.94	[MAPK12]
FLJ80095AAAF	406	46.94	[PDK3]
FLJ80096AAAF	372	42.34	[STK17B]
FLJ80097AAAF	675	78.01	[BMX]
FLJ80099AAAF	311	34.19	[PIM2]
FLJ80100AAAF	648	73.05	[RAF1]
FLJ80101AAAF	400	44.42	[MAP2K2]
FLJ80104AAAF	506	57.7	[NEK3]
FLJ80105AAAF	313	35.69	[PIM1]
FLJ80108AAAF	76	7.99	[PKIA]
FLJ80109AAAF	493	54.98	[ACVR1C]
FLJ80110AAAF	719	80.77	[PAK7]
FLJ80112AAAF	473	51.93	[CAMK4]
FLJ80113AAAF	1315	144	[STK36]
FLJ80114AAAF	483	50.98	[GSK3A]
FLJ80115AAAF	1085	122.38	[BUB1]
FLJ80116AAAF	581	62.74	[PINK1]
FLJ80117AAAF	532	60.2	[BMPR1A]
FLJ80118AAAF	464	54	[STK38L]
FLJ80119AAAF	341	38.55	[PDIK1L]
FLJ80120AAAF	337	39.07	[CSNK1A1L]
FLJ80121AAAF	484	57.29	[CLK1]
FLJ80122AAAF	360	41.29	[MAPK14]
FLJ80123AAAF	1106	124.03	[PDGFRB]
FLJ80124AAAF	894	98.36	[AXL]
FLJ80126AAAF	424	48.14	[MAPK9]
FLJ80127AAAF	766	86.7	[TLK1]
FLJ80128AAAF	725	82.22	[EEF2K]
FLJ80129AAAF	523	59.58	[CDK17]
FLJ80131AAAF	509	57.15	[ACVR1]
FLJ80132AAAF	527	59.05	[CAMK2G]
FLJ80134AAAF	729	83.64	[TBK1]
FLJ80135AAAF	1007	116.99	[PRPF4B]
FLJ80136AAAF	1036	112.73	[ULK2]
FLJ80138AAAF	688	77.53	[SRPK2]
FLJ80139AAAF	268	30.1	[TSSK3]
FLJ80141AAAF	721	82.67	[MAPK6]
FLJ80142AAAF	947	104.04	[MAP3K14]
FLJ80143AAAF	431	47.91	[STK24]
FLJ80144AAAF	681	74.87	[PAK6]
FLJ80145AAAF	385	42.91	[CAMK1D]
FLJ80147AAAF	846	95.05	[MAP4K5]

FLJ80148AAAF	970	108.97	[PLK4]
FLJ80149AAAF	399	44.36	[MAP2K4]
FLJ80151AAAF	537	60.17	[CPNE3]
FLJ80152AAAF	673	77.03	[PRKCB]
FLJ80154AAAN	976	108.27	[EPHA2]
FLJ80157AAAF	550	62.81	[PRKAA1]
FLJ80158AAAF	358	41	[TSSK2]
FLJ80159AAAF	689	79.57	[ADRBK1]
FLJ80162AAAF	655	74.31	[SRPK1]
FLJ80163AAAF	626	67.73	[TESK1]
FLJ80164AAAF	783	84.93	[SIK1]
FLJ80165AAAF	436	49.24	[PDK1]
FLJ80166AAAF	351	40.59	[PRKACA]
FLJ80167AAAF	351	40.46	[PRKACG]
FLJ80169AAAF	411	46.47	[PDK4]
FLJ80171AAAF	358	40.9	[PRKX]
FLJ80172AAAF	592	67.51	[CDKL3]
FLJ80173AAAF	1009	115.87	[PTK2B]
FLJ80174AAAF	503	56.12	[ACVRL1]
FLJ80176AAAF	505	55.74	[CAMKK1]
FLJ80177AAAF	445	51.76	[NEK2]
FLJ80179AAAF	414	46.56	[STK17A]
FLJ80183AAAF	419	48.2	[IKBKG]
FLJ80184AAAF	890	96.91	[TYRO3]
FLJ80185AAAF	646	71.65	[WEE1]
FLJ80186AAAF	998	110.33	[EPHB3]
FLJ80187AAAF	1038	115.2	[BMPR2]
FLJ80189AAAN	499	60.09	[CLK2]
FLJ80190AAAF	619	69.87	[ZAP70]
FLJ80191AAAF	305	34.66	[STK16]
FLJ80192AAAF	1374	154.54	[MAP3K5]
FLJ80194AAAF	618	64.62	[DLL3]
FLJ80195AAAF	193	21.12	[CD70]
FLJ80196AAAF	594	68.21	[GLMN]
FLJ80197AAAF	211	23.03	[SDF2]
FLJ80198AAAF	266	28.67	[DKK1]
FLJ80199AAAF	359	40.32	[WNT5B]
FLJ80200AAAF	227	25.21	[TMED1]
FLJ80201SAAF	317	34.8	[FST]
FLJ80202AAAF	365	39.72	[WNT6]
FLJ80203AAAF	91	9.99	[SCGB1A1]
FLJ80204AAAF	683	74.68	[TGFBI]
FLJ80205AAAF	110	11.98	[INS]
FLJ80208AAAF	131	14.51	[MIA]
FLJ80209AAAF	168	18.94	[PTN]
FLJ80210AAAF	185	21.15	[RLN1]
FLJ80211AAAF	207	23.99	[FGF18]
FLJ80212AAAF	366	39.67	[INHA]

FLJ80215AAAF	193	22.33	[IL18]
FLJ80216AAAF	350	38.29	[DKK3]
FLJ80217AAAF	296	31.7	[NRG1]
FLJ80218AAAF	170	19.34	[PGF]
FLJ80219AAAF	426	47.44	[INHBA]
FLJ80220AAAF	181	20.63	[PDAP1]
FLJ80221AAAF	238	24.8	[GAP43]
FLJ80222AAAF	303	34.63	[SPARC]
FLJ80224AAAF	114	11.97	[CXCL5]
FLJ80225AAAF	431	49.31	[BMP7]
FLJ80226AAAF	91	9.99	[CCL5]
FLJ80227AAAF	98	10.99	[CCL13]
FLJ80228AAAF	269	30.75	[IL1B]
FLJ80229AAAF	349	39	[WNT7A]
FLJ80230AAAF	207	21.6	[VEGFB]
FLJ80231AAAF	230	25.15	[FAM3A]
FLJ80232AAAF	165	17.92	[ISG15]
FLJ80233AAAF	99	11.03	[CCL2]
FLJ80235AAAF	252	27.9	[AREG]
FLJ80236AAAF	98	10.88	[CXCL10]
FLJ80237AAAF	202	22.57	[CLEC3B]
FLJ80238AAAF	178	19.78	[BTC]
FLJ80239AAAF	107	11.3	[CXCL1]
FLJ80240AAAF	493	57.1	[ANGPTL2]
FLJ80241AAAF	210	22.43	[NTF4]
FLJ80242AAAF	199	21.43	[IL11]
FLJ80243AAAF	94	10.36	[CXCL11]
FLJ80244AAAF	109	12.66	[CXCL13]
FLJ80245AAAF	174	19.74	[NMU]
FLJ80246AAAF	271	30.61	[IL1A]
FLJ80247AAAF	114	11.9	[CXCL6]
FLJ80248AAAF	265	28.16	[ACRV1]
FLJ80249AAAF	357	39.18	[NOV]
FLJ80250AAAF	212	23.72	[IL6]
FLJ80251AAAF	107	11.39	[CXCL2]
FLJ80252AAAF	227	25.88	[PRL]
FLJ80253AAAF	397	42.2	[CX3CL1]
FLJ80254AAAF	281	31.48	[FASLG]
FLJ80255AAAF	216	24	[FGF19]
FLJ80256AAAF	238	26.35	[EFNA3]
FLJ80257AAAF	57	6.96	[HTN1]
FLJ80258AAAF	97	10.73	[CCL11]
FLJ80259AAAF	162	18.09	[IL15]
FLJ80260AAAN	209	22.28	[FGF21]
FLJ80261AAAF	240	26.79	[HDGF]
FLJ80264AAAF	408	46.53	[BMP4]
FLJ80265AAAF	285	31.22	[TNFSF13B]
FLJ80266AAAF	160	18.45	[LY96]

FLJ80267AAAF	217	25	[GH2]
FLJ80268AAAF	77	8.57	[APLN]
FLJ80269AAAF	554	60.12	[CSF1]
FLJ80270AAAF	188	21.14	[TDGF1]
FLJ80271AAAF	145	16.37	[GNLY]
FLJ80272AAAF	93	10.16	[SCGB3A2]
FLJ80273AAAF	155	16.96	[IL36RN]
FLJ80274AAAF	201	23.54	[ORM1]
FLJ80275AAAF	139	15.45	[INSL4]
FLJ80276AAAF	366	40.88	[LEFTY1]
FLJ80277AAAN	93	10.16	[CCL3L3]
FLJ80281AAAF	454	51.74	[BMP5]
FLJ80282AAAF	98	10.99	[CCL19]
FLJ80283AAAF	128	13.89	[PPBP]
FLJ80285AAAF	104	10.1	[SCGB3A1]
FLJ80286AAAF	247	27.85	[BDNF]
FLJ80287AAAF	133	15.04	[NDP]
FLJ80288AAAF	401	46.03	[TNFRSF11B]
FLJ80289AAAF	751	85.21	[SEMA3C]
FLJ80290AAAF	364	41.49	[GDF3]
FLJ80291AAAF	477	51.99	[OSGIN1]
FLJ80293AAAF	155	17.46	[FGF1]
FLJ80294AAAF	281	32.51	[TNFSF10]
FLJ80295AAAF	381	42.12	[SFTPB]
FLJ80296AAAF	84	9.15	[TFF1]
FLJ80300AAAF	232	25.77	[NOG]
FLJ80302AAAF	205	22.35	[LTA]
FLJ80303AAAF	349	39.33	[WNT7B]
FLJ80304AAAF	419	46.88	[VEGFC]
FLJ80305AAAF	431	47.04	[NPTX2]
FLJ80306AAAF	175	19.4	[REG3A]
FLJ80307AAAF	417	45.42	[GIF]
FLJ80308AAAF	553	61.32	[EGFL6]
FLJ80310AAAF	89	10.1	[CXCL12]
FLJ80311AAAF	352	39	[AMBP]
FLJ80312AAAF	785	88.4	[SEMA3F]
FLJ80314AAAF	93	10.68	[CCL14]
FLJ80315AAAF	177	20.19	[IL7]
FLJ80316AAAN	367	39.32	[AHSG]
FLJ80317AAAF	711	80.32	[MST1]
FLJ80318AAAF	346	38.01	[EFNB1]
FLJ80319AAAF	115	12.48	[MIF]
FLJ80320AAAF	159	16.93	[TGFA]
FLJ80321AAAF	308	34.14	[GDF15]
FLJ80322AAAF	207	23.91	[IL24]
FLJ80324SAAF	197	21.95	[IL37]
FLJ80325AAAF	95	10.69	[CCL20]
FLJ80326AAAF	407	45.18	[INHBB]

FLJ80327WAAF	201	21.23	[CTF1]
FLJ80328WAAF	241	26.99	[NGF]
FLJ80329WAAF	244	27.69	[TNFSF11]
FLJ80331SAAF	178	20.46	[CRISP1]
FLJ80332SAAF	1922	218.68	[DICER1]
FLJ80333AAAF	338	39.79	[GPR160]
FLJ80334AAAF	328	36.43	[P2RY6]
FLJ80335AAAF	447	48.42	[CCKBR]
FLJ80336AAAF	422	45.96	[MCHR1]
FLJ80338AAAF	350	38.45	[FPR1]
FLJ80339AAAF	377	41.91	[HTR1D]
FLJ80340AAAF	331	38.13	[GPR18]
FLJ80341AAAF	350	39.29	[C5AR1]
FLJ80342AAAF	373	41.48	[GPR173]
FLJ80343AAAF	787	86.4	[SMO]
FLJ80344AAAF	438	49.48	[VIPR2]
FLJ80345AAAF	295	32.35	[RGR]
FLJ80346AAAF	375	42.26	[GPER1]
FLJ80347AAAF	397	44.13	[F2RL1]
FLJ80348AAAF	442	49.64	[EDNRB]
FLJ80349AAAF	384	41.62	[S1PR4]
FLJ80350AAAF	574	63.62	[FZD7]
FLJ80351AAAF	415	47.77	[NMUR2]
FLJ80352AAAF	336	35.63	[ACKR1]
FLJ80353AAAF	396	43.71	[GPR84]
FLJ80354AAAF	443	50.62	[DRD2]
FLJ80356AAAF	410	45.46	[NTSR2]
FLJ80357AAAF	374	41.76	[PTGER3]
FLJ80358AAAF	332	36.33	[ADORA2B]
FLJ80359WAAF	537	59.88	[FZD4]
FLJ80360AAAF	368	37.62	[GPR62]
FLJ80361AAAF	742	81.74	[ADGRE5]
FLJ80362AAAF	363	41.85	[HCAR2]
FLJ80363AAAF	351	38.96	[FPR2]
FLJ80365AAAF	319	36.64	[GPR55]
FLJ80366AAAF	330	35.01	[GPR3]
FLJ80367AAAF	494	51.74	[GPR135]
FLJ80370AAAF	404	45.32	[GPR182]
FLJ80371AAAF	338	38.97	[P2RY14]
FLJ80372AAAF	870	96.92	[GPR155]
FLJ80373AAAF	391	42.69	[SSTR1]
FLJ80374AAAF	346	38.65	[FFAR3]
FLJ80376AAAF	362	41.48	[ACKR3]
FLJ80378AAAF	319	36.74	[GPR171]
FLJ80379AAAF	374	42.49	[CCR6]
FLJ80380AAAF	352	40.52	[CCR5]
FLJ80382AAAF	613	67.11	[GPR37]
FLJ80383AAAF	359	40.27	[PTGDR]

FLJ80384AAAF	532	60.09	[CHRM5]
FLJ80386AAAF	445	49.49	[HTR7]
FLJ80387AAAF	915	99.29	[LGR6]
FLJ80388AAAF	450	48.96	[ADRA2A]
FLJ80389AAAF	339	38.77	[P2RY10]
FLJ80390SAAF	359	40.1	[HRH2]
FLJ80391AAAF	1328	148.87	[MYBBP1A]
FLJ80392AAAF	708	78.24	[PJA2]
FLJ80395AAAF	292	32.23	[MLEC]
FLJ80402AAAN	575	59.95	[TMEM108]
FLJ80403AAAF	502	55.88	[IL17RB]
FLJ80412AAAF	283	30.42	[TNFRSF14]
FLJ80413AAAF	479	51.36	[PVRL2]
FLJ80417AAAF	1072	119.82	[RET]
FLJ80420AAAF	261	28.16	[RTBDN]
FLJ80421AAAF	150	16.37	[GYPA]
FLJ80422AAAF	257	29.6	[FCER1A]
FLJ80425AAAF	293	33.97	[TBC1D7]
FLJ80429AAAF	814	88.92	[CDH15]
FLJ80430AAAF	240	25.41	[CD7]
FLJ80432AAAF	128	15.49	[NDUFB6]
FLJ80434AAAF	226	25.4	[PRRG4]
FLJ80438AAAF	216	24.15	[ALG14]
FLJ80439AAAF	170	19.16	[SAT2]
FLJ80443AAAF	277	30.62	[CD40]
FLJ80446AAAF	195	21.83	[KLRG1]
FLJ80449AAAF	364	39.25	[HAVCR1]
FLJ80450AAAN	493	54.77	[FZR1]
FLJ80454WAAF	370	38.25	[SDC3]
FLJ80458AAAF	255	27.12	[SUSD3]
FLJ80462AAAF	282	30.7	[TMIGD2]
FLJ80463AAAN	798	86.9	[ITGB7]
FLJ80465AAAF	199	22.5	[DNAJC5B]
FLJ80468AAAF	287	32.55	[ASGR2]
FLJ80469AAAF	215	24.48	[MPZL2]
FLJ80470AAAF	155	16.89	[C1orf162]
FLJ80472AAAN	588	68.82	[TAF1B]
FLJ80474AAAF	206	21.65	[NAT14]
FLJ80477AAAN	798	88.42	[ITGB1]
FLJ80478AAAF	311	33.92	[C10orf54]
FLJ80481AAAF	685	77.42	[STIM1]
FLJ80484AAAN	328	36.7	[CD207]
FLJ80488AAAF	332	36.06	[CD300LG]
FLJ80489AAAF	333	36.97	[LECT1]
FLJ80490AAAF	387	42.88	[SGCA]
FLJ80493AAAF	410	46.13	[TEX28]
FLJ80498AAAF	190	19.95	[FAM174A]
FLJ80499AAAF	561	64.93	[CHST15]

FLJ80500AAAF	287	32.26	[FCAR]
FLJ80501AAAF	626	68.97	[GP1BA]
FLJ80502AAAF	636	69.47	[IL27RA]
FLJ80503AAAF	444	49.09	[KIR3DL1]
FLJ80504AAAF	790	88.94	[ADAM30]
FLJ80507AAAF	291	33.37	[ATP4B]
FLJ80509AAAF	315	34.42	[NPHS2]
FLJ80512AAAF	288	34.17	[TMCO5A]
FLJ80513AAAF	294	32.14	[SPACA1]
FLJ80514AAAF	500	56.7	[B3GALNT2]
FLJ80515AAAF	743	81.88	[DCBLD2]
FLJ80516AAAF	151	16.65	[TEX29]
FLJ80517AAAF	359	40.22	[CD72]
FLJ80518AAAF	384	42.74	[CD46]
FLJ80520AAAF	205	23.04	[CD83]
FLJ80522AAAF	291	33.19	[ASGR1]
FLJ80525AAAF	374	42.63	[FCGR1A]
FLJ80526AAAF	1032	115.86	[TLR9]
FLJ80528AAAF	120	12.31	[RPRML]
FLJ80529AAAF	986	108.06	[PTPRN2]
FLJ80534AAAF	380	40.93	[TMEFF1]
FLJ80542AAAF	1169	128.52	[ITGAX]
FLJ80543AAAF	1016	113.93	[SMG5]
FLJ80545AAAN	1391	155.2	[NUP155]
FLJ80547AAAF	216	25.27	[KLRK1]
FLJ80559AAAF	207	23.15	[CD3E]
FLJ80562AAAF	775	87.82	[ECEL1]
FLJ80565AAAF	461	48.29	[TNFRSF1B]
FLJ80566AAAF	862	96.21	[SEMA4D]
FLJ80571AAAF	148	16.99	[RAMP1]
FLJ80573AAAF	254	28.99	[EMD]
FLJ80574AAAF	129	15.21	[NDUFB4]
FLJ80575AAAF	266	29.82	[HLA-DRB3]
FLJ80578AAAF	372	40.63	[CNTFR]
FLJ80580AAAF	160	18.02	[BIK]
FLJ80581AAAF	100	11.44	[VAMP8]
FLJ80582AAAF	96	9.97	[SEC61B]
FLJ80589AAAF	129	13.91	[TNFRSF12A]
FLJ80590AAAF	116	12.66	[VAMP2]
FLJ80591AAAF	690	76.19	[LATS1]
FLJ80598AAAF	173	19	[SSR4]
FLJ80602AAAF	146	16.33	[CYB5B]
FLJ80609AAAF	390	43.15	[FAIM3]
FLJ80612AAAF	418	48.31	[ACPP]
FLJ80617AAAF	389	43.95	[ART3]
FLJ80618AAAN	259	29.35	[USE1]
FLJ80619AAAF	99	10.71	[SMIM14]
FLJ80620AAAF	74	8.29	[TMEM167B]

FLJ80624AAAF	622	68.01	[MSLN]
FLJ80628AAAN	212	24.78	[GOSR2]
FLJ80629AAAF	236	26.91	[STX8]
FLJ80634AAAF	82	8.97	[IER3IP1]
FLJ80636AAAF	228	26.13	[BNIP1]
FLJ80637AAAN	620	69.88	[LINGO1]
FLJ80638AAAF	989	110.31	[MED24]
FLJ80639AAAF	261	29.21	[HLA-DMA]
FLJ80642AAAF	449	46.14	[C11orf24]
FLJ80643AAAF	261	29.82	[STX7]
FLJ80644AAAF	380	42.38	[CCDC51]
FLJ80648AAAF	246	28	[RTP4]
FLJ80649AAAF	121	13.24	[C10orf35]
FLJ80655AAAF	335	38.67	[STX18]
FLJ80656AAAN	618	68.12	[RHOT2]
FLJ80657AAAN	459	49.73	[CA9]
FLJ80661AAAF	103	11.4	[ATP5L]
FLJ80665AAAF	650	70.82	[LILRB1]
FLJ80666AAAF	243	27.68	[CD48]
FLJ80669AAAF	383	42.95	[MICA]
FLJ80671AAAF	411	45.67	[DPEP1]
FLJ80673AAAF	356	40.23	[LMAN2]
FLJ80680AAAF	116	12.8	[VAMP5]
FLJ80681AAAF	453	51.64	[ENPP4]
FLJ80686AAAF	84	9.41	[HAMP]
FLJ80687AAAF	380	44.18	[IL13RA2]
FLJ80697AAAN	831	92.07	[SORT1]
FLJ80698AAAF	123	12.91	[PSCA]
FLJ80704AAAF	263	28.94	[HLA-DMB]
FLJ80705AAAF	239	26.27	[BCL2]
FLJ80709AAAF	335	37.72	[CD1D]
FLJ80710AAAF	201	22.18	[FKBP11]
FLJ80715AAAF	318	34.94	[RDH5]
FLJ80718AAAF	177	19.08	[GP9]
FLJ80719WAAF	304	34.48	[NCR1]
FLJ80720AAAF	965	109.36	[VPS54]
FLJ80723AAAF	230	25.7	[LRAT]
FLJ80724AAAF	477	53.05	[NTRK2]
FLJ80726AAAF	341	37.88	[KIR2DL3]
FLJ80728AAAF	382	43.81	[PRELP]
FLJ80742AAAF	246	27.37	[ULBP2]
FLJ80744AAAF	716	80.16	[ADAM2]
FLJ80746AAAF	737	78.49	[DNER]
FLJ80763AAAF	611	69.39	[RHOBTB3]
FLJ80768AAAF	692	76.37	[NRROS]
FLJ80770AAAF	417	45.31	[TNFRSF19]
FLJ80771AAAF	287	33.34	[STX2]
FLJ80776AAAF	240	25.72	[TMEM219]

FLJ80777AAAF	292	32.86	[RNF144A]
FLJ80778AAAF	215	24.45	[CABP7]
FLJ80781AAAF	613	65.03	[IGSF8]
FLJ80782AAAF	997	114.14	[RABGAP1]
FLJ80788AAAF	145	16.3	[TOMM20]
FLJ80789AAAF	393	42.79	[IDH3G]
FLJ80799AAAF	155	17.6	[MGST1]
FLJ80801AAAF	199	22.56	[CD69]
FLJ80809AAAF	103	11.32	[G0S2]
FLJ80813AAAF	113	12.18	[TYROBP]
FLJ80821AAAF	321	36.47	[FCER2]
FLJ80826AAAF	107	11.34	[CXCL3]
FLJ80835AAAF	283	30.49	[CCDC107]
FLJ80838AAAF	1194	133.94	[DHX30]
FLJ80843AAAF	281	31.2	[HAS3]
FLJ80844AAAF	250	27.47	[KLK11]
FLJ80845AAAF	273	30.96	[OLR1]
FLJ80849AAAF	105	11.73	[PROK1]
FLJ80851AAAF	569	61.41	[GGT1]
FLJ80860AAAF	95	11.01	[LINC00301]
FLJ80862AAAF	170	18.7	[TMEM252]
FLJ80864AAAF	89	9.96	[TMEM196]
FLJ80866AAAF	431	48.55	[FAM69B]
FLJ80868AAAF	529	59.58	[NOP58]
FLJ80874AAAF	563	64.32	[ITPRIPL1]
FLJ80879AAAF	107	11.39	[SERTM1]
FLJ80884AAAF	316	35.45	[CLEC10A]
FLJ80885AAAF	422	47.2	[P2RX5]
FLJ80886AAAF	228	25.09	[BRICD5]
FLJ80888AAAF	570	64.45	[FAM20C]
FLJ80893AAAF	700	80.64	[PTPRE]
FLJ80895AAAF	166	19.79	[STK32A]
FLJ80896AAAF	541	59.17	[RPS6KL1]
FLJ80897AAAF	616	69.43	[HIPK4]
FLJ80899AAAF	323	36.78	[PPP2R4]
FLJ80900AAAF	443	51.71	[PPP2R2B]
FLJ80901AAAF	447	51.69	[PPP2R2A]
FLJ80902AAAF	497	57.39	[PPP2R5B]
FLJ80903AAAF	575	65.09	[PPP2R3B]
FLJ80904AAAF	1003	113.66	[MOV10]
FLJ80905AAAF	614	69.15	[DDX5]
FLJ80906AAAF	973	109.85	[PIWIL2]
FLJ80907AAAF	861	98.55	[PIWIL1]
FLJ80908WAAF	1058	120.01	[GEMIN4]
FLJ80909WAAF	773	86.05	[DGCR8]
FLJ80910AAAF	1226	135.97	[ADAR]
FLJ80911AAAN	1192	138.16	[DROSHA]
FLJ80912AAAF	857	97.21	[AGO1]

FLJ80913AAAF	313	34.4	[PRKRA]
FLJ80915AAAF	701	76.6	[ADARB1]
FLJ80917AAAF	597	68.05	[KARS]
FLJ80918AAAF	475	53.26	[PARS2]
FLJ80920AAAF	455	49.87	[TUFM]
FLJ80922AAAF	346	37.66	[TSFM]
FLJ80923AAAF	903	101.98	[LARS2]
FLJ80924AAAF	645	73.56	[DARS2]
FLJ80925AAAF	751	83.47	[GFM1]
FLJ80930AAAF	102	12.27	[MRPL57]
FLJ80931AAAF	235	27.42	[HSCB]
FLJ80933AAAF	316	35.15	[YIPF2]
FLJ80934AAAF	92	10.16	[MRPL34]
FLJ80935AAAF	378	44.47	[CDC37]
FLJ80936AAAF	411	46.56	[NAGA]
FLJ80937AAAF	546	60.44	[CDT1]
FLJ80938AAAF	354	40.18	[GNAT2]
FLJ80939AAAF	325	35.51	[MACROD1]
FLJ80940AAAF	248	27.77	[SFN]
FLJ80941AAAF	139	15.35	[LSM4]
FLJ80942AAAF	224	24.81	[SYNGR2]
FLJ80943AAAF	106	11.53	[HIGD2A]
FLJ80944AAAF	428	48.85	[HDAC3]
FLJ80945AAAF	179	19.08	[SCAND1]
FLJ80946AAAF	119	13.6	[SSNA1]
FLJ80947AAAF	211	22.34	[NABP2]
FLJ80948AAAF	210	23.47	[RHOD]
FLJ80950AAAF	110	12.02	[EMC6]
FLJ80951AAAF	307	35.08	[PPP4C]
FLJ80952AAAF	140	15.65	[POP7]
FLJ80953AAAF	244	25.91	[DCXR]
FLJ80954AAAF	455	48.34	[MFSD10]
FLJ80955AAAF	302	33.89	[DNASE1L1]
FLJ80957AAAF	384	43.47	[AAR2]
FLJ80958AAAF	361	37.89	[TOMM40]
FLJ80959AAAF	325	34.16	[OTP]
FLJ80961AAAF	166	17.7	[CDKN2D]
FLJ80962AAAF	267	28.53	[TEX101]
FLJ80963AAAF	208	24.5	[TRIM48]
FLJ80964AAAF	253	28.77	[PGAM2]
FLJ80966AAAF	268	28.14	[C21orf33]
FLJ80967AAAF	557	59.63	[PTBP1]
FLJ80968AAAF	208	23.06	[EMC9]
FLJ80969AAAF	593	64.8	[BEGAIN]
FLJ80970AAAF	204	21.64	[C9orf142]
FLJ80971AAAF	112	12.15	[UCN2]
FLJ80972AAAF	140	15.99	[CENPA]
FLJ80973AAAF	226	25.29	[MYL10]

FLJ80975AAAF	153	17.91	[RNF181]
FLJ80976AAAF	302	32.9	[DOHH]
FLJ80977AAAF	210	21.09	[CTAG2]
FLJ80978AAAF	260	27.37	[PCSK1N]
FLJ80979AAAF	258	28.43	[RSG1]
FLJ80980AAAF	294	33.16	[TSPAN15]
FLJ80981AAAF	142	15.65	[FKBP2]
FLJ80982AAAF	217	24.99	[ZBED2]
FLJ80983AAAF	114	13.46	[TCL1A]
FLJ80984AAAF	175	19.28	[B9D2]
FLJ80985AAAF	319	35.81	[TMEM121]
FLJ80986AAAF	115	12.49	[SHANK2-AS3]
FLJ80987AAAF	211	23.31	[CBX2]
FLJ80989AAAF	376	42.29	[ACTR1B]
FLJ80990AAAF	270	28.5	[CITED2]
FLJ80991AAAF	146	16.15	[PAGE1]
FLJ80992AAAF	143	15.14	[H2AFX]
FLJ80993AAAF	448	47.55	[SLC10A3]
FLJ80994AAAF	293	33.72	[TRIM51]
FLJ80996AAAF	260	29.23	[C19orf52]
FLJ80997AAAF	432	45.73	[HOXD3]
FLJ80998AAAF	185	21.25	[ANAPC10]
FLJ80999AAAF	180	20.91	[GCG]
FLJ81000AAAN	188	21.86	[SSX4]
FLJ81001AAAF	222	25.77	[LXN]
FLJ81002AAAF	97	11	[SPANXD]
FLJ81003AAAF	248	27.66	[CAPNS2]
FLJ81004AAAF	269	31.66	[CENPK]
FLJ81005AAAF	153	16.71	[NPPA]
FLJ81006AAAF	115	12.4	[MIEN1]
FLJ81007AAAF	329	34.29	[TSSC4]
FLJ81008AAAF	400	43.53	[POU2F2]
FLJ81010AAAF	358	39.42	[PDCD2L]
FLJ81011AAAF	194	22.08	[C7orf50]
FLJ81012AAAF	129	13.7	[COX5B]
FLJ81013AAAF	197	22.1	[CRYBA2]
FLJ81014AAAF	198	23.95	[AICDA]
FLJ81015AAAF	290	30.87	[VAX2]
FLJ81016AAAF	284	30.25	[TLX2]
FLJ81017AAAF	272	30.17	[HOXA9]
FLJ81018AAAF	236	26.65	[CLIC3]
FLJ81019AAAF	122	13.53	[SAA1]
FLJ81020AAAF	223	25.39	[APCS]
FLJ81021AAAF	234	25.13	[ZBED3]
FLJ81024AAAF	110	12.41	[PHF5A]
FLJ81025AAAF	303	34.86	[STUB1]
FLJ81026AAAF	366	38.98	[ABI3]
FLJ81028WAAF	224	25.22	[FAM57B]

FLJ81030AAAF	293	32.02	[KLK5]
FLJ81031AAAF	345	37.38	[GMPR]
FLJ81032AAAF	550	62.63	[CCDC102A]
FLJ81033AAAF	116	13.2	[CDKN2AIPNL]
FLJ81034AAAF	61	6.04	[MT1H]
FLJ81035AAAF	214	22.93	[MS4A3]
FLJ81036AAAF	504	55.18	[PRPF19]
FLJ81037AAAF	477	54.27	[ZNF622]
FLJ81038AAAF	241	26.64	[PEX11G]
FLJ81039AAAF	258	26.45	[C1QL1]
FLJ81040AAAF	1107	123.55	[POLD1]
FLJ81041AAAF	238	25.45	[EDN3]
FLJ81042AAAF	64	7.71	[NOP10]
FLJ81043AAAF	290	31.4	[ECHS1]
FLJ81044AAAF	185	20.65	[RABAC1]
FLJ81046AAAF	95	10.83	[LSM2]
FLJ81047AAAF	130	14.05	[PAGE5]
FLJ81048AAAF	136	15.81	[TMEM203]
FLJ81049AAAF	314	34.6	[FAHD2A]
FLJ81050AAAF	63	6.99	[STRA13]
FLJ81051AAAF	192	21.38	[RAC3]
FLJ81052AAAF	163	18.43	[EMP3]
FLJ81053AAAF	336	39.15	[VPS26B]
FLJ81054AAAF	473	53.19	[PPAN]
FLJ81055AAAF	329	34.67	[SDSL]
FLJ81056AAAF	404	46.21	[KRT33B]
FLJ81058AAAF	205	22.35	[DRAP1]
FLJ81059AAAF	308	35.31	[MAGEF1]
FLJ81060AAAF	330	36.89	[EEF2KMT]
FLJ81061AAAF	184	19.39	[FDX1]
FLJ81062SAAF	288	32.39	[HACD1]
FLJ81063AAAF	251	27.38	[C11orf68]
FLJ81064AAAF	492	54.43	[FRS3]
FLJ81065AAAF	475	54.17	[TRIM21]
FLJ81065WAAN	286	32.85	[TRIM21(1-286)]
FLJ81066AAAF	296	34.93	[SULT1B1]
FLJ81067AAAF	126	13.9	[ZNRD1]
FLJ81068AAAF	210	23.34	[GSTP1]
FLJ81069AAAF	326	36.96	[ALX1]
FLJ81070AAAF	182	20.25	[CSN3]
FLJ81071AAAF	117	12.27	[PI3]
FLJ81072AAAF	454	51.39	[OLFM2]
FLJ81073AAAF	424	48.83	[CHST8]
FLJ81074AAAF	470	51.72	[NR0B1]
FLJ81075AAAF	261	27.64	[PRKCDBP]
FLJ81076AAAF	302	31.83	[PITX3]
FLJ81077AAAF	174	19.11	[DNPH1]
FLJ81078AAAF	390	43.12	[LHX4]

FLJ81079AAAF	312	34.76	[RPUSD1]
FLJ81080AAAF	191	20.89	[TSR2]
FLJ81081AAAF	108	12.32	[POLR3K]
FLJ81082AAAF	412	42.7	[MFSD3]
FLJ81083AAAF	218	21.71	[TAF10]
FLJ81084AAAF	359	39.7	[ARR3]
FLJ81085AAAF	239	25.7	[CLDN14]
FLJ81088AAAF	221	23.52	[HES4]
FLJ81089AAAF	265	30.47	[PQBP1]
FLJ81090AAAF	237	25.94	[NRL]
FLJ81091AAAF	502	52.91	[WAS]
FLJ81093AAAF	408	45.39	[KCNK13]
FLJ81094AAAF	101	11.71	[S100A3]
FLJ81095AAAF	227	26.43	[ZMYND19]
FLJ81096AAAF	496	53.5	[SMAD6]
FLJ81097AAAF	205	22.95	[PSMB3]
FLJ81098AAAF	205	24.21	[MRPS26]
FLJ81099AAAF	227	25.95	[RAB3C]
FLJ81100AAAF	330	35.43	[FOXS1]
FLJ81101AAAF	266	30.37	[RASD2]
FLJ81102AAAF	328	37.49	[RCN3]
FLJ81103AAAF	513	58.49	[TRIM27]
FLJ81104AAAF	270	29.35	[HOXA5]
FLJ81107AAAF	223	25.84	[CCDC124]
FLJ81108AAAF	247	28.55	[TLCD1]
FLJ81109AAAF	256	28.87	[MAF1]
FLJ81110AAAF	140	14.56	[FLYWCH2]
FLJ81111AAAF	670	75.95	[LRRC45]
FLJ81112AAAF	212	23.94	[NXNL1]
FLJ81114AAAF	255	28.84	[CTSG]
FLJ81115AAAF	283	31.62	[FBXO27]
FLJ81116AAAF	233	26.12	[GLIPR1L1]
FLJ81117AAAF	395	43.97	[SH3GLB2]
FLJ81118AAAF	253	27.08	[PRRX2]
FLJ81119AAAF	264	28.71	[ZNF524]
FLJ81120AAAF	264	30.53	[LENG1]
FLJ81121AAAF	229	24.69	[OIP5]
FLJ81122AAAF	446	52.27	[TRIM43]
FLJ81123AAAF	229	25.4	[EBI3]
FLJ81127WAAF	136	15.39	[HIST2H3D]
FLJ81128AAAF	223	26.11	[ELSPBP1]
FLJ81129AAAF	143	14.8	[LAGE3]
FLJ81130AAAF	165	17.66	[NKG7]
FLJ81131AAAF	211	23.71	[RAB38]
FLJ81132AAAF	290	33.51	[NAT2]
FLJ81133AAAF	479	50.94	[LENG9]
FLJ81134AAAF	350	37.14	[FOXA3]
FLJ81135AAAF	248	27.74	[C14orf80]

FLJ81138AAAF	571	59.35	[PRR35]
FLJ81139AAAF	229	26.28	[SSX5]
FLJ81140AAAF	159	19.14	[RGS13]
FLJ81141AAAF	220	23.31	[SAP30]
FLJ81142AAAF	294	34.27	[GLYATL2]
FLJ81145AAAF	291	31.87	[TLX3]
FLJ81147AAAF	322	36.26	[RNF113B]
FLJ81148AAAF	198	23.1	[CAPSL]
FLJ81149AAAF	145	16.56	[DNAJB3]
FLJ81150AAAF	78	8.46	[GYPE]
FLJ81152AAAF	165	18.68	[PMCH]
FLJ81153AAAF	138	16.56	[UCMA]
FLJ81154AAAF	298	33.78	[DPPA2]
FLJ81155AAAF	98	11.4	[NDUFB3]
FLJ81156AAAF	206	23.2	[EBPL]
FLJ81157AAAF	740	81.54	[ACAP1]
FLJ81158AAAF	477	50.82	[SLC2A8]
FLJ81159AAAF	475	50.81	[ZNF296]
FLJ81160AAAF	159	16.99	[TMEM42]
FLJ81161AAAF	647	72.22	[PDE4A]
FLJ81163AAAF	222	25.28	[GSTA3]
FLJ81164AAAF	125	14.65	[SDHAF3]
FLJ81165AAAF	201	23.04	[RBP4]
FLJ81166AAAF	188	21.25	[APOM]
FLJ81167AAAF	62	6.14	[MT1G]
FLJ81168AAAF	223	25.43	[PEBP4]
FLJ81170AAAF	219	23.52	[CLDN20]
FLJ81171AAAF	166	18.01	[ZCCHC13]
FLJ81172AAAF	265	30.82	[C16orf78]
FLJ81173AAAF	215	23.63	[HAND1]
FLJ81174AAAF	177	19.48	[C7orf33]
FLJ81175AAAF	260	28.13	[CACNG6]
FLJ81176AAAF	194	21.56	[LYZL1]
FLJ81177AAAF	382	42.64	[RAB3IL1]
FLJ81178AAAF	375	37.66	[SFTPD]
FLJ81179AAAF	99	11.56	[S100Z]
FLJ81181AAAF	135	14.73	[NXNL2]
FLJ81184AAAF	238	25.03	[FEV]
FLJ81185AAAF	523	57.98	[RCOR2]
FLJ81188AAAF	779	85.4	[PLEKHA4]
FLJ81190AAAF	274	30.93	[PRR7]
FLJ81192AAAF	509	57.15	[PLA2G3]
FLJ81195AAAF	257	27.45	[GZMM]
FLJ81196AAAF	331	37.18	[AKR7A3]
FLJ81197AAAF	223	24.58	[FGFBP2]
FLJ81198AAAF	670	73.59	[RHPN1]
FLJ81199AAAF	134	14.73	[NPPB]
FLJ81200AAAF	117	12.93	[GHRL]

FLJ81201AAAF	109	12.4	[CIRBP-AS1]
FLJ81202AAAF	520	58.66	[TULP2]
FLJ81203AAAF	109	13.19	[CCDC26]
FLJ81205AAAF	253	27.95	[PPP1R35]
FLJ81207AAAF	591	64.52	[FZD9]
FLJ81209AAAF	300	34.51	[GIMAP7]
FLJ81210AAAF	207	22.9	[LIN7B]
FLJ81211AAAF	261	28.87	[CA1]
FLJ81212AAAF	142	15.64	[HBZ]
FLJ81213AAAF	94	10.24	[DEFA3]
FLJ81214AAAF	246	27.31	[GZMH]
FLJ81215AAAF	175	19.61	[RAMP2]
FLJ81216AAAF	99	10.85	[APOC3]
FLJ81217AAAF	118	13.36	[LINC00518]
FLJ81218AAAF	177	20.19	[FANCD2OS]
FLJ81220AAAF	312	35.63	[OR2L13]
FLJ81221AAAF	439	47.49	[LILRA3]
FLJ81222AAAF	253	29.48	[C7orf62]
FLJ81223AAAF	243	27.05	[VSTM2A]
FLJ81224AAAF	379	43.44	[THEG]
FLJ81225AAAF	474	52.38	[CBLC]
FLJ81226AAAF	194	21.43	[LYG1]
FLJ81227AAAF	129	13.72	[PRR15]
FLJ81228AAAF	275	30.53	[TPSB2]
FLJ81229AAAF	61	6.13	[MT1A]
FLJ81230AAAF	226	25.32	[CLRN3]
FLJ81231AAAF	421	47.25	[GOT1L1]
FLJ81232AAAF	509	55.24	[ZNF683]
FLJ81233AAAF	55	6.42	[TNP1]
FLJ81234AAAF	220	25.32	[C15orf43]
FLJ81235AAAF	288	30.08	[CT47A11]
FLJ81236AAAF	196	23.14	[CLEC1B]
FLJ81237AAAF	97	11.09	[LINC00305]
FLJ81239AAAF	97	10.82	[COX6A2]
FLJ81240AAAF	277	31.2	[TNFAIP6]
FLJ81241AAAF	123	13.3	[GAL]
FLJ81242AAAF	289	33.6	[TEX26]
FLJ81243AAAF	96	10.5	[LINC00337]
FLJ81245AAAF	265	29.45	[WBSCR28]
FLJ81246AAAF	145	15.4	[C10orf91]
FLJ81247AAAF	119	12.92	[LINC00311]
FLJ81248AAAF	57	5.96	[PRAC1]
FLJ81249AAAF	206	24.01	[BTG4]
FLJ81250WAAF	505	56.67	[OSGIN2]
FLJ81251AAAF	214	22.88	[FAM101B]
FLJ81252AAAF	136	15.58	[CYTL1]
FLJ81256AAAF	111	12.25	[RBAKDN]
FLJ81259AAAF	446	47.31	[SOX8]

FLJ81260AAAF	80	8.59	[SPINK6]
FLJ81261AAAF	113	13.51	[FAM27E3]
FLJ81262AAAF	171	20.01	[TTC9C]
FLJ81263AAAF	182	21.34	[TNNI2]
FLJ81264AAAF	95	10.45	[PPY]
FLJ81265AAAF	125	14.12	[BATF]
FLJ81266AAAF	248	29.18	[SPIC]
FLJ81267AAAF	188	20.46	[C12orf45]
FLJ81268AAAF	176	18.69	[FAM89B]
FLJ81269AAAF	152	16.87	[ATOH7]
FLJ81270AAAF	334	36.38	[DHDH]
FLJ81271AAAF	129	14.28	[TFF2]
FLJ81272AAAF	265	28.29	[AQP5]
FLJ81273AAAF	780	90.18	[CATSPER1]
FLJ81274AAAF	261	29.16	[C1orf111]
FLJ81275AAAF	108	12.11	[LINC00471]
FLJ81277AAAF	250	25.7	[MAST4]
FLJ81278AAAF	353	38.69	[DND1]
FLJ81279AAAF	214	24.07	[PIH1D3]
FLJ81280AAAF	336	39.22	[TBC1D21]
FLJ81281AAAF	287	33.2	[STX11]
FLJ81282AAAF	411	47.18	[ZNF679]
FLJ81283AAAF	362	38.23	[KLF1]
FLJ81284AAAF	468	53.05	[CHRNA5]
FLJ81285AAAF	451	50.33	[TUBB1]
FLJ81286AAAF	437	51.02	[CPA6]
FLJ81287AAAF	557	59.39	[CCT8L2]
FLJ81289AAAF	224	24.95	[CHMP4B]
FLJ81290AAAF	173	18.74	[NAA38]
FLJ81292AAAF	468	51.28	[TMEM151A]
FLJ81293AAAF	215	24.15	[C19orf18]
FLJ81294AAAF	105	11.73	[SPATA8]
FLJ81295AAAF	270	31.5	[ELOVL3]
FLJ81298AAAF	395	41.6	[C2orf57]
FLJ81300AAAF	103	11.83	[SPANXB1]
FLJ81303AAAF	101	11.46	[S100A7]
FLJ81306AAAF	155	17.18	[FUNDC1]
FLJ81307AAAF	634	69.16	[KIAA2013]
FLJ81310AAAF	164	17.88	[UBALD2]
FLJ81311AAAF	329	35.26	[C1QTNF4]
FLJ81312AAAF	463	51.14	[DCAF12L1]
FLJ81313AAAF	184	21.46	[C10orf67]
FLJ81315AAAF	281	30.6	[CEBPE]
FLJ81316AAAF	577	64.79	[HAS1]
FLJ81317AAAF	102	11.84	[AHSP]
FLJ81318AAAF	80	8.95	[COX7B2]
FLJ81319AAAF	93	9.49	[HCST]
FLJ81320AAAF	542	58.73	[LRRC56]

FLJ81321AAAF	346	38.22	[CALHM1]
FLJ81322AAAF	196	21.17	[MGC39545]
FLJ81324AAAF	405	44.07	[HSD11B2]
FLJ81325AAAF	340	36.91	[SVOPL]
FLJ81326AAAF	272	28.62	[NEUROG2]
FLJ81328AAAF	194	20.43	[MED19]
FLJ81329AAAF	282	30.86	[PNMT]
FLJ81330AAAF	606	63.88	[RAVER1]
FLJ81333AAAF	289	31.84	[HOXD8]
FLJ81334AAAF	627	66.48	[LZTS3]
FLJ81335AAAF	217	23.43	[CALY]
FLJ81336AAAF	146	15.89	[CALML5]
FLJ81338AAAF	928	102.43	[RFX6]
FLJ81340AAAF	593	67.47	[DNAI2]
FLJ81341AAAF	236	25.39	[C11orf53]
FLJ81342AAAF	507	54.99	[SLC7A5]
FLJ81345AAAF	274	31.06	[ODF3L1]
FLJ81346AAAF	137	15.38	[MRPL41]
FLJ81347AAAF	507	54.87	[LDHD]
FLJ81349AAAF	414	47.18	[SERPINA12]
FLJ81351AAAF	434	45.31	[TFAP2E]
FLJ81352AAAF	173	19.55	[CMTM8]
FLJ81355AAAF	679	75.36	[MISP]
FLJ81356AAAF	191	22.03	[PIP5KL1]
FLJ81357AAAF	195	21.84	[TEX33]
FLJ81359AAAF	417	47.48	[KRT36]
FLJ81360AAAF	459	49.15	[ZBTB12]
FLJ81361AAAF	477	52.05	[MYBPH]
FLJ81363AAAF	251	25.38	[C17orf82]
FLJ81364AAAF	503	51.89	[CXorf67]
FLJ81367AAAF	68	7.42	[DEFB1]
FLJ81368AAAF	157	16.63	[GLRX5]
FLJ81369AAAF	430	46.92	[KCNK12]
FLJ81370AAAF	406	45.06	[REN]
FLJ81371AAAF	160	18.93	[CNIH2]
FLJ81372AAAF	348	38.6	[TMPRSS12]
FLJ81374AAAF	389	42.24	[SAPCD2]
FLJ81375AAAF	435	45.86	[PROSER2]
FLJ81377AAAF	272	31.08	[ABT1]
FLJ81380AAAF	317	35.58	[SFRP5]
FLJ81381AAAF	128	14.94	[TCL1B]
FLJ81382AAAF	402	41.8	[PTGER1]
FLJ81383AAAF	219	23.55	[TMEM179B]
FLJ81384AAAF	1191	125.54	[ATN1]
FLJ81385AAAF	343	40.58	[TMEM120A]
FLJ81387AAAF	1008	102.37	[GPRIN1]
FLJ81389AAAF	599	65.17	[CENPB]
FLJ81391AAAF	224	25.04	[MYOG]

FLJ81394AAAF	111	12.09	[PAGE2]
FLJ81395AAAF	97	11	[SPANXC]
FLJ81397AAAF	148	16.96	[LYZL6]
FLJ81398AAAF	663	73.33	[RAP1GAP]
FLJ81399AAAF	90	10.31	[BANF2]
FLJ81400AAAF	89	9.37	[FXYD4]
FLJ81403AAAF	202	22.66	[PPP1R2P9]
FLJ81404AAAF	240	27.59	[MORN3]
FLJ81405AAAF	171	20.01	[COX4I2]
FLJ81406AAAF	203	22.54	[RASL10A]
FLJ81407AAAF	184	20.14	[TNFRSF17]
FLJ81408AAAF	649	74.27	[SCNN1G]
FLJ81409AAAF	129	13.99	[HIST2H2AC]
FLJ81410AAAF	742	81.56	[COLEC12]
FLJ81413AAAF	85	9.7	[FDCSP]
FLJ81414AAAF	138	14.25	[LACRT]
FLJ81415AAAF	95	10.88	[SCGB2A1]
FLJ81416AAAF	243	27.48	[IL27]
FLJ81417AAAF	376	38.77	[FOXL2]
FLJ81419AAAF	141	16.44	[CST2]
FLJ81420AAAF	111	12.3	[XAGE3]
FLJ81421AAAF	110	11.28	[DCD]
FLJ81422AAAF	90	9.9	[SCGB1D1]
FLJ81423AAAF	215	23.22	[NACA2]
FLJ81424AAAF	172	19.14	[MRAP]
FLJ81426AAAF	423	45.81	[OXER1]
FLJ81427AAAF	257	28.15	[GSC]
FLJ81428AAAF	184	19.85	[GPIHBP1]
FLJ81429AAAF	254	27.3	[BARX1]
FLJ81430AAAF	216	23.89	[C8orf82]
FLJ81432AAAF	138	15.82	[MRPL54]
FLJ81433AAAF	176	19.25	[LCN1]
FLJ81434AAAF	379	43.71	[AADACL2]
FLJ81435AAAF	267	29.42	[POMC]
FLJ81436AAAF	126	13.99	[HIST1H2BM]
FLJ81440AAAF	162	18.65	[IL21]
FLJ81441AAAF	179	20.01	[IL22]
FLJ81442AAAF	189	20.73	[IL23A]
FLJ81443AAAF	171	19.84	[IL26]
FLJ81444AAAF	152	17.22	[IL3]
FLJ81445AAAF	134	15.24	[IL5]
FLJ81446AAAF	144	15.91	[IL9]
FLJ81447AAAF	194	21.58	[LYZL2]
FLJ81448AAAF	218	24.36	[BPIFA3]
FLJ81450AAAF	372	41.97	[GPR45]
FLJ81453AAAF	463	50.29	[IRGC]
FLJ81454AAAF	381	42.57	[POTEB3]
FLJ81456AAAF	322	36.48	[MRGPRX3]

FLJ81457AAAF	315	34.12	[SOX12]
FLJ81458AAAF	202	22.77	[SAMD10]
FLJ81459AAAF	356	40.09	[GPR32]
FLJ81460AAAF	333	36.87	[NPBWR2]
FLJ81461AAAF	328	37.17	[IL12B]
FLJ81462AAAF	153	17.49	[IL4]
FLJ81463AAAF	315	34.69	[ABHD11]
FLJ81464AAAF	631	71.17	[DDX53]
FLJ81467AAAF	222	25.38	[GADD45GIP1]
FLJ81470AAAF	163	18.06	[IL17F]
FLJ81473AAAF	114	12.57	[XCL2]
FLJ81474AAAF	114	12.52	[XCL1]
FLJ81476AAAF	258	29.14	[SPIN2A]
FLJ81478AAAF	92	10.09	[CCL3]
FLJ81483AAAF	256	28.34	[C21orf2]
FLJ81485WAAF	209	22.28	[FAM127A]
FLJ81486AAAF	201	21.41	[KRTAP4-12]
FLJ81487AAAF	287	31.57	[FAM212A]
FLJ81488AAAF	106	10.9	[C19orf33]
FLJ81491WAAF	193	20.18	[ASCL2]
FLJ81493WAAF	118	11.63	[LCE1B]
FLJ81495WAAF	346	39.72	[PAQR7]
FLJ81497AAAF	304	33.06	[GPR162]
FLJ81500AAAF	384	43.44	[ACKR2]
FLJ81504AAAF	342	39.44	[P2RY12]
FLJ81505AAAF	369	41.33	[SSTR2]
FLJ81506AAAF	361	41.22	[GPR183]
FLJ81509AAAF	586	66.39	[CRY1]
FLJ81513AAAF	337	39.33	[GPR65]
FLJ81516AAAF	402	44.87	[OPN3]
FLJ81519AAAF	879	98.91	[GRM3]
FLJ81520AAAF	359	40.64	[P2RY8]
FLJ81521AAAF	647	71.16	[FZD1]
FLJ81522AAAF	355	41.17	[CCR1]
FLJ81523AAAF	353	39.97	[FPR3]
FLJ81525AAAF	342	39.2	[PTAFR]
FLJ81526AAAF	549	60.9	[GPR97]
FLJ81527AAAF	1346	149.46	[ADGRF5]
FLJ81530AAAF	423	48.34	[GPR83]
FLJ81532AAAF	362	40.98	[GPR4]
FLJ81534AAAF	424	46.05	[GPR143]
FLJ81536AAAF	386	40.96	[PTGIR]
FLJ81537AAAF	414	46.39	[ARRDC3]
FLJ81538AAAF	418	45.45	[ARRDC4]
FLJ81539AAAF	433	45.98	[ARRDC1]
FLJ81540AAAF	757	86.4	[MFN2]
FLJ81543AAAF	333	37.8	[SUGT1]
FLJ81544AAAF	305	30.84	[HNRNPA0]

FLJ81545AAAF	325	38.71	[FAM50B]
FLJ81546AAAF	263	29.56	[PSMA1]
FLJ81547AAAF	709	81.08	[SSRP1]
FLJ81549AAAF	268	28.5	[MESP1]
FLJ81550AAAF	351	38.77	[THOC3]
FLJ81552AAAF	296	33.25	[MRPS2]
FLJ81553AAAF	132	14.56	[IFITM2]
FLJ81555AAAF	367	41.73	[FBXO31]
FLJ81556AAAF	199	21.47	[SSSCA1]
FLJ81557AAAF	217	23.98	[HOXB7]
FLJ81558AAAF	336	37.98	[TOR1B]
FLJ81560AAAF	685	77.92	[MID2]
FLJ81561AAAF	616	68.14	[RPA1]
FLJ81562AAAF	959	106.03	[GTF2IRD1]
FLJ81563AAAF	481	54.85	[LACE1]
FLJ81564AAAF	415	46.32	[SERPINA7]
FLJ81565AAAF	225	24.35	[RAB21]
FLJ81566AAAF	551	61.49	[TRIM8]
FLJ81567AAAN	655	69.66	[FOXO1]
FLJ81568AAAF	540	61.12	[MPP6]
FLJ81569AAAF	572	64.15	[ME1]
FLJ81570AAAF	574	61.7	[NEURL1]
FLJ81571AAAF	800	88.16	[LENG8]
FLJ81572AAAF	418	45.89	[FBXL14]
FLJ81573AAAF	242	26.01	[C6orf223]
FLJ81574AAAF	338	38.54	[PHYH]
FLJ81577AAAF	830	92.97	[TCIRG1]
FLJ81578AAAF	1425	156.4	[ZFYVE9]
FLJ81579AAAF	837	93.93	[KAT2A]
FLJ81580AAAF	519	56.63	[DPYS]
FLJ81581AAAF	115	12.37	[CDK2AP1]
FLJ81582AAAF	453	48.61	[HTRA3]
FLJ81584AAAF	530	56.42	[GMEB2]
FLJ81586AAAF	535	58.33	[TBX21]
FLJ81587AAAF	274	30.73	[GCLM]
FLJ81588AAAF	426	48.05	[KCNN3]
FLJ81590AAAF	264	29.78	[HOXC4]
FLJ81592AAAF	1242	131.59	[IRS1]
FLJ81593AAAF	84	9.28	[NDUFA3]
FLJ81594AAAF	145	17.04	[RPS15]
FLJ81596AAAF	524	59.97	[VANGL1]
FLJ81598AAAF	503	57.47	[CYP3A7]
FLJ81599AAAF	361	41.35	[GPR52]
FLJ81604AAAF	525	59.76	[CORO2A]
FLJ81605AAAF	400	43.4	[MVD]
FLJ81606AAAF	432	47.44	[WDR18]
FLJ81607AAAF	258	29.16	[SPIN2B]
FLJ81609AAAN	458	48.84	[HTRA2]

FLJ81612AAAF	253	28.78	[C1orf43]
FLJ81613AAAF	347	38.29	[SCAMP3]
FLJ81614AAAF	128	15.09	[MRPL51]
FLJ81615AAAF	225	24.76	[EEF1B2]
FLJ81616AAAF	148	17.21	[RNF24]
FLJ81618AAAF	169	19.02	[NME3]
FLJ81619AAAF	490	54.82	[THRA]
FLJ81620AAAF	293	33.31	[CASP6]
FLJ81621AAAF	235	26.46	[NAA10]
FLJ81622AAAF	302	33.82	[SRM]
FLJ81624AAAF	314	34.75	[MAGEA3]
FLJ81625AAAF	123	14.55	[RPL35]
FLJ81626AAAF	480	52.22	[UMPS]
FLJ81627AAAF	261	26.92	[HSD17B10]
FLJ81628AAAF	476	52.23	[GPKOW]
FLJ81629AAAN	432	48.55	[TRIP13]
FLJ81631AAAF	213	22.49	[H1FX]
FLJ81632AAAF	282	31.36	[C1QBP]
FLJ81633AAAF	422	47.46	[PSMD11]
FLJ81634AAAF	198	21.89	[PRDX2]
FLJ81635AAAF	346	39.28	[PEX16]
FLJ81636AAAF	332	35.54	[TP53I3]
FLJ81637AAAF	118	13.53	[SNRPD2]
FLJ81638AAAF	351	39	[EIF2B2]
FLJ81640AAAF	424	47.97	[KCNJ8]
FLJ81641AAAF	408	45.89	[ACY1]
FLJ81642AAAF	236	25.94	[PEMT]
FLJ81643AAAF	314	35.61	[DDRGK1]
FLJ81645AAAF	176	18.99	[TPPP3]
FLJ81646AAAF	150	17.14	[POLR2H]
FLJ81648AAAF	203	22.5	[AKIRIN2]
FLJ81649AAAF	327	37.76	[NDUFAF1]
FLJ81650AAAF	203	22.77	[RAB13]
FLJ81651AAAF	194	22.59	[RPS9]
FLJ81652AAAF	159	18.64	[TCEAL1]
FLJ81653AAAF	101	11.73	[S100A4]
FLJ81654AAAF	288	32.81	[LACTB2]
FLJ81655AAAF	126	13.89	[HIST1H2BK]
FLJ81656AAAF	125	13.94	[IFITM1]
FLJ81657AAAF	295	34.15	[SULT1A1]
FLJ81658AAAF	183	20.81	[CBX3]
FLJ81660AAAF	193	20.95	[CSRP2]
FLJ81661AAAF	188	21.93	[SSX1]
FLJ81662AAAF	169	18.78	[TSPO]
FLJ81663AAAF	224	25.26	[TMEM147]
FLJ81665AAAF	218	25.65	[MRPS34]
FLJ81666AAAF	487	56.25	[PTDSS2]
FLJ81667AAAF	233	26.06	[PBDC1]

FLJ81668AAAF	353	39.96	[PLEK2]
FLJ81670AAAF	293	32.01	[YIF1A]
FLJ81671AAAF	217	24.64	[RGS19]
FLJ81672AAAF	280	31.19	[FHL3]
FLJ81673AAAF	215	24.74	[SEC22B]
FLJ81676AAAF	138	15.17	[MRPS12]
FLJ81677AAAF	180	20.58	[MRPL18]
FLJ81678AAAF	106	12.29	[COX16]
FLJ81679AAAF	304	33.83	[CRK]
FLJ81680AAAF	163	17.66	[FAM96B]
FLJ81681AAAF	169	19.86	[NDUFAF2]
FLJ81682AAAF	185	19.9	[MEA1]
FLJ81683AAAF	92	10.71	[S100B]
FLJ81684AAAF	470	53.54	[TRAF4]
FLJ81685AAAF	117	13.29	[RPL34]
FLJ81686AAAF	160	17.83	[FAM195A]
FLJ81688AAAF	910	102.97	[RAD54B]
FLJ81689AAAF	198	21.35	[MRPL12]
FLJ81690AAAF	331	38.24	[NUDC]
FLJ81692WAAF	242	27.12	[HTATIP2]
FLJ81693AAAF	241	26.9	[CCDC28B]
FLJ81694AAAF	346	38.73	[G6PC3]
FLJ81695AAAF	212	24.7	[PDCD10]
FLJ81696AAAF	290	32.11	[PIR]
FLJ81697AAAF	137	16.4	[NDUFB7]
FLJ81699AAAF	328	36.24	[CA11]
FLJ81701AAAF	79	9.08	[TMEM258]
FLJ81702AAAF	316	34.89	[ELP5]
FLJ81703AAAF	531	59.7	[CCDC9]
FLJ81704AAAF	335	36.98	[PLAUR]
FLJ81705AAAF	280	29.94	[LYL1]
FLJ81706AAAF	348	39	[NCF4]
FLJ81707AAAF	277	30.85	[CBR3]
FLJ81708AAAF	223	25.17	[SSX2]
FLJ81710AAAF	78	7.62	[NRGN]
FLJ81711AAAF	103	11.71	[PRR15L]
FLJ81712AAAF	257	29.82	[FOLR1]
FLJ81713AAAF	236	25.81	[CD81]
FLJ81714AAAF	231	25.73	[PAFAH1B3]
FLJ81715AAAF	173	19.64	[TMEM208]
FLJ81716AAAF	119	12.97	[ID3]
FLJ81717AAAF	364	40.59	[VPS72]
FLJ81718AAAF	105	11.74	[TXN]
FLJ81719AAAF	264	28.97	[TAF9]
FLJ81721AAAF	368	42.13	[ZNF24]
FLJ81722AAAF	219	24.28	[RBPMS]
FLJ81723AAAF	271	30.54	[PRDX4]
FLJ81724AAAF	234	26.26	[FGFBP1]

FLJ81725AAAF	502	57.26	[TFCP2]
FLJ81726AAAF	418	47.07	[ARRB1]
FLJ81727AAAF	132	14.72	[FABP4]
FLJ81728AAAF	99	10.92	[NDUFA2]
FLJ81729AAAF	325	35.12	[DHRS7B]
FLJ81730AAAF	423	46.16	[NR2F1]
FLJ81731AAAF	480	50.41	[RBM42]
FLJ81733AAAF	253	28.28	[TFPT]
FLJ81734AAAF	122	13.1	[PSMG3]
FLJ81736AAAF	278	30.75	[CNPY3]
FLJ81737AAAF	148	16.21	[GRP]
FLJ81738AAAF	344	37.79	[PCGF2]
FLJ81740AAAF	297	32.65	[ENDOG]
FLJ81741AAAF	147	16.83	[NUDT2]
FLJ81742AAAF	405	45.34	[UBAC1]
FLJ81744AAAF	172	19.78	[MYL12B]
FLJ81745AAAF	173	18.91	[MARVELD1]
FLJ81746AAAF	192	20.68	[MRPL11]
FLJ81747AAAF	497	51.45	[ZFP36L2]
FLJ81748AAAF	104	11.66	[S100A14]
FLJ81749AAAF	152	17.09	[PHLDA2]
FLJ81750AAAF	219	24.76	[RAB3B]
FLJ81751AAAF	235	25.52	[MOSPD3]
FLJ81752AAAF	121	14.28	[ANAPC15]
FLJ81753AAAF	379	42.5	[ARMCX3]
FLJ81754AAAF	162	19.26	[TMEM138]
FLJ81755AAAF	141	16.02	[C1D]
FLJ81756AAAF	144	16.3	[HSPB11]
FLJ81757AAAF	213	24.38	[EBAG9]
FLJ81758AAAF	323	37.64	[CCNH]
FLJ81759AAAF	174	19.81	[EEF1E1]
FLJ81760AAAF	207	22.83	[NFYB]
FLJ81761AAAF	359	39.75	[DCN]
FLJ81762AAAF	245	28.23	[TPMT]
FLJ81763AAAF	166	18.73	[REG1A]
FLJ81765AAAF	167	19.55	[CETN3]
FLJ81766AAAF	263	27.87	[CTRB1]
FLJ81767AAAF	156	16.83	[CUTA]
FLJ81769AAAF	145	17.08	[NDUFA12]
FLJ81770AAAF	72	7.91	[GNG12]
FLJ81771AAAF	221	25.03	[SLC50A1]
FLJ81772AAAF	244	27.33	[TBCB]
FLJ81773AAAF	147	16.6	[UBE2D1]
FLJ81774AAAF	117	13.67	[GABARAPL2]
FLJ81775AAAF	469	51.95	[PNLIPRP2]
FLJ81776AAAF	94	10.96	[LINC00467]
FLJ81777AAAF	235	26.46	[CHCHD6]
FLJ81779AAAF	295	31.5	[ORAI3]

FLJ81780AAAF	303	32.59	[NUDT22]
FLJ81781AAAF	117	13.09	[C19orf48]
FLJ81782AAAF	234	25.95	[INF2]
FLJ81784AAAF	270	28.35	[HSD17B14]
FLJ81785AAAF	340	37.69	[DUSP12]
FLJ81788AAAF	104	12	[PCBD1]
FLJ81789AAAF	1012	111.69	[UBA7]
FLJ81790AAAF	79	9.86	[CKS2]
FLJ81791AAAF	433	48.37	[CCNB1]
FLJ81793AAAF	600	67.69	[LMNB2]
FLJ81794AAAF	133	14.63	[IFITM3]
FLJ81795AAAF	95	10.4	[S100P]
FLJ81796AAAF	352	39.75	[DYNC2LI1]
FLJ81797AAAF	101	11.22	[CRIPT]
FLJ81798AAAF	334	38.71	[RSRC1]
FLJ81799AAAF	61	6.04	[MT2A]
FLJ81801AAAF	423	48.41	[CPB2]
FLJ81802AAAF	511	57.71	[AMY2A]
FLJ81803AAAF	112	11.95	[CLPS]
FLJ81804AAAF	260	29.63	[DPM1]
FLJ81805AAAF	240	26.7	[EFHD2]
FLJ81806AAAF	524	59.09	[CDC25A]
FLJ81807AAAF	113	12.5	[DAD1]
FLJ81808AAAF	154	16.31	[NMB]
FLJ81809AAAF	158	17.98	[ACP1]
FLJ81810AAAF	846	93.98	[EXO1]
FLJ81812AAAF	538	56.93	[AJUBA]
FLJ81814AAAF	205	23.02	[PPP1R2]
FLJ81815WAAF	183	20.9	[UBE2M]
FLJ81817AAAF	734	82.06	[MZF1]
FLJ81818AAAF	150	17.42	[PDE6D]
FLJ81819AAAF	246	25.03	[MDFI]
FLJ81822AAAF	380	40.51	[UBL7]
FLJ81823AAAF	61	6.51	[URB1-AS1]
FLJ81824AAAF	486	55.74	[PACSIN2]
FLJ81825AAAF	527	59.68	[MAOA]
FLJ81826AAAF	261	26.97	[HSD17B8]
FLJ81827AAAF	178	20.28	[MED30]
FLJ81828AAAF	346	38.76	[ENKD1]
FLJ81829AAAF	432	49.73	[PCED1B]
FLJ81830AAAF	118	13.76	[ATP6V1G1]
FLJ81831AAAF	154	17.47	[KIAA1143]
FLJ81832AAAF	79	8.18	[ROMO1]
FLJ81833AAAN	142	15.26	[HBA2]
FLJ81835AAAF	319	35.24	[ACY3]
FLJ81837AAAF	284	32.16	[SIX1]
FLJ81838AAAF	253	26.72	[C1QB]
FLJ81840AAAF	485	54.93	[POLG2]

FLJ81841AAAF	111	12.35	[XAGE2]
FLJ81842AAAF	244	28.38	[CTDNEP1]
FLJ81843AAAF	230	25.46	[FAM207A]
FLJ81844AAAF	396	44.93	[DXO]
FLJ81845AAAF	181	21.21	[RGS10]
FLJ81849AAAF	392	45.12	[FEZ1]
FLJ81850AAAF	145	16.39	[PTS]
FLJ81851AAAF	213	24.62	[RAB39B]
FLJ81852AAAF	170	19.97	[ZMAT5]
FLJ81853AAAF	317	33.73	[PRSS22]
FLJ81854AAAF	236	26.74	[CDC34]
FLJ81855AAAF	311	34.92	[MRPL4]
FLJ81856WAAF	491	55.23	[MDM2]
FLJ81858AAAF	713	80.61	[MIPEP]
FLJ81859AAAF	260	29.34	[HLA-DPA1]
FLJ81860AAAF	194	21.45	[ITPA]
FLJ81861AAAF	172	18.27	[TIMM17B]
FLJ81862AAAF	82	9.24	[DPH3]
FLJ81863AAAF	394	45.95	[ZSCAN9]
FLJ81864AAAF	98	11.01	[CSTA]
FLJ81867AAAF	362	38.51	[AVEN]
FLJ81869AAAF	103	11.8	[S100A16]
FLJ81870AAAF	338	38.73	[TFAP4]
FLJ81871AAAF	169	19.82	[SNX24]
FLJ81872AAAF	147	16.13	[HBG1]
FLJ81873AAAF	147	16.13	[HBG2]
FLJ81875AAAF	356	40.52	[AIM2]
FLJ81877AAAF	968	106.81	[AARS]
FLJ81878AAAF	1024	114.68	[POP1]
FLJ81879AAAF	343	38.95	[CCDC97]
FLJ81881AAAF	1178	129.63	[PC]
FLJ81883AAAF	308	33.25	[RRAD]
FLJ81884AAAF	440	47.54	[CTBP1]
FLJ81885WAAF	285	29.95	[C1QTNF2]
FLJ81886AAAF	420	46.44	[HNRNPDL]
FLJ81887AAAF	632	67.87	[ILVBL]
FLJ81888AAAF	339	39.85	[STEAP1]
FLJ81891AAAF	318	36.38	[C1GALT1C1]
FLJ81892AAAF	296	34.16	[BTG3]
FLJ81893AAAF	325	34.35	[DBP]
FLJ81895AAAF	437	46.5	[SLC10A4]
FLJ81897AAAF	167	19.02	[MRPS24]
FLJ81898AAAF	114	12.26	[PDZK1IP1]
FLJ81899AAAF	162	17.85	[MAFG]
FLJ81900AAAF	183	20.5	[RAP2B]
FLJ81902AAAF	227	25.63	[NAT8]
FLJ81903AAAF	311	35.92	[FOXR2]
FLJ81904AAAF	261	29.2	[CTDSP1]

FLJ81905AAAF	68	6.93	[MT3]
FLJ81907AAAF	209	22.4	[TM4SF19]
FLJ81908AAAF	210	24.36	[RGS17]
FLJ81913AAAF	375	42.58	[KCNJ15]
FLJ81914AAAF	744	73.36	[COL8A1]
FLJ81915AAAF	604	69	[PTGS2]
FLJ81916AAAF	378	42.77	[BTBD8]
FLJ81917AAAF	312	33.6	[TSR3]
FLJ81918AAAF	258	27.55	[PGLS]
FLJ81920AAAF	640	73.45	[USP49]
FLJ81921AAAF	260	28.79	[EAF2]
FLJ81922AAAF	184	21.45	[TIFA]
FLJ81923AAAF	92	10.28	[RPL37A]
FLJ81924AAAF	138	14.72	[CDKN2B]
FLJ81925AAAF	298	32.8	[BIRC7]
FLJ81926AAAF	210	24.62	[ALMS1P]
FLJ81927AAAF	739	83.35	[LETM1]
FLJ81928AAAF	122	13.53	[C7orf34]
FLJ81929AAAF	376	41.7	[ACTRT1]
FLJ81930AAAF	224	25.43	[HOXB6]
FLJ81931AAAF	161	16.62	[ID4]
FLJ81932AAAF	228	24.82	[RYBP]
FLJ81933AAAF	273	31.37	[XPA]
FLJ81934AAAF	268	29.48	[CTRC]
FLJ81936AAAF	500	56.38	[RBCK1]
FLJ81937AAAF	294	31.85	[SMN2]
FLJ81938WAAF	301	32.59	[CRLS1]
FLJ81939AAAF	427	47.7	[KCNN4]
FLJ81940AAAF	118	13.47	[CHCHD1]
FLJ81941AAAF	335	37.38	[TADA1]
FLJ81942AAAF	317	34.3	[ANKRD9]
FLJ81943AAAF	104	9.55	[IFI27L1]
FLJ81944AAAF	271	31.57	[CALB2]
FLJ81945AAAF	267	28.87	[MSX2]
FLJ81946AAAF	218	25.56	[GSTM4]
FLJ81950AAAF	821	88.02	[BRAT1]
FLJ81951AAAF	262	28.97	[GZMA]
FLJ81952AAAF	210	23.55	[COPZ2]
FLJ81953AAAF	76	8.5	[ZNF706]
FLJ81954AAAF	186	21	[RBBP9]
FLJ81955AAAF	460	49.67	[MOCS3]
FLJ81956AAAF	188	20.48	[ZNF428]
FLJ81958AAAF	97	11.2	[S100A10]
FLJ81959WAAF	276	30.04	[EXOSC8]
FLJ81960AAAF	404	43.4	[C18orf25]
FLJ81961AAAF	189	21.1	[HEBP1]
FLJ81963AAAF	218	23.48	[RRAS]
FLJ81964AAAF	271	31.05	[ARV1]

FLJ81965AAAF	336	38.29	[XRCC4]
FLJ81967AAAF	117	13.35	[C4orf36]
FLJ81968AAAF	146	16.43	[LYZL4]
FLJ81969AAAF	330	37.65	[CFHR1]
FLJ81970AAAF	219	22.17	[TMEM125]
FLJ81972AAAF	277	31.61	[CASP3]
FLJ81974AAAF	425	48.24	[TRIM31]
FLJ81975AAAF	198	23.42	[DNAJC12]
FLJ81976AAAF	203	23.44	[VTI1A]
FLJ81977AAAF	158	18.23	[REG4]
FLJ81978AAAF	288	31.32	[IMPA2]
FLJ81982AAAF	504	55.32	[PNPLA2]
FLJ81984AAAF	322	34.14	[RBKS]
FLJ81985AAAF	363	39.68	[RFC4]
FLJ81986AAAF	115	12.72	[NRG4]
FLJ81987AAAF	186	20.86	[TNFAIP8L1]
FLJ81988AAAF	346	39.54	[TFB1M]
FLJ81991AAAF	109	12.35	[SLIRP]
FLJ81992AAAF	184	20.78	[CHAC2]
FLJ81993AAAF	322	36.14	[CRHBP]
FLJ81994AAAF	278	30.96	[RAB40B]
FLJ81995AAAF	129	15	[TAC1]
FLJ81996AAAF	314	33.78	[CRYM]
FLJ81998AAAF	324	34.73	[TMEM171]
FLJ81999AAAF	108	12.22	[C6orf57]
FLJ82001AAAF	61	6.07	[MT1X]
FLJ82002AAAF	340	39.08	[ST3GAL1]
FLJ82003AAAF	674	74.09	[FLRT1]
FLJ82004AAAF	249	29.31	[MITD1]
FLJ82005AAAF	319	36.25	[ANKRD1]
FLJ82006AAAF	125	13.67	[BRI3]
FLJ82007AAAF	315	35.96	[PIH1D2]
FLJ82008AAAF	501	53.45	[SPAG8]
FLJ82009AAAF	260	29.11	[BAMBI]
FLJ82012AAAF	538	57.69	[SLC22A17]
FLJ82013AAAF	962	109.99	[XPOT]
FLJ82014AAAF	307	34.06	[CDK5R1]
FLJ82015AAAF	259	28.97	[RRP15]
FLJ82016AAAF	241	28.67	[CCDC59]
FLJ82017AAAF	99	11.06	[HIGD1B]
FLJ82018AAAF	400	44.91	[BLZF1]
FLJ82019AAAF	199	23.34	[HPGDS]
FLJ82020AAAF	290	32.2	[SGCD]
FLJ82021AAAF	297	34.81	[EMC2]
FLJ82022AAAF	122	13.51	[SAA2]
FLJ82023AAAF	351	38.84	[HAO2]
FLJ82024AAAF	147	16.69	[PPP1R14A]
FLJ82027AAAF	92	10.32	[C9orf116]

FLJ82030AAAF	305	34.75	[SPATA4]
FLJ82031AAAF	148	17.18	[C2orf40]
FLJ82033AAAF	381	43.77	[PHF7]
FLJ82034AAAF	315	35.02	[SLC25A31]
FLJ82035AAAF	137	15.5	[CRABP1]
FLJ82036WAAF	173	20.68	[N4BP2L1]
FLJ82039AAAF	270	30.65	[CFHR2]
FLJ82040AAAF	527	59.18	[RAG2]
FLJ82041AAAF	128	14.43	[FABP6]
FLJ82042AAAF	574	64.07	[TRIM23]
FLJ82043AAAF	217	24.62	[ZCRB1]
FLJ82044AAAF	411	46.71	[CHST1]
FLJ82045AAAF	165	17.74	[CGB8]
FLJ82046AAAF	250	26.31	[MS4A8]
FLJ82047AAAF	210	23.14	[FXN]
FLJ82048AAAF	501	52.1	[IRX3]
FLJ82049AAAF	157	17.76	[RFESD]
FLJ82050AAAF	416	45.52	[ACTL9]
FLJ82055AAAF	325	36.6	[ACP5]
FLJ82056AAAF	250	27.9	[GCH1]
FLJ82058AAAF	764	84.5	[SLC26A3]
FLJ82059AAAF	179	19.72	[TCF21]
FLJ82060AAAF	186	21.21	[TRAT1]
FLJ82061AAAF	327	36.6	[BCL2L14]
FLJ82062AAAF	389	43.85	[ALKBH1]
FLJ82063AAAF	79	8.51	[SPINK1]
FLJ82064AAAF	221	26.15	[RP9]
FLJ82066AAAF	370	42.84	[USP12]
FLJ82067AAAF	429	47.48	[SLC30A4]
FLJ82068AAAF	485	55.77	[ZNF165]
FLJ82069AAAF	307	33.92	[C1orf228]
FLJ82070AAAF	379	43.08	[SUN5]
FLJ82071AAAF	511	58.4	[SPAM1]
FLJ82073AAAF	179	19.15	[MPLKIP]
FLJ82074AAAF	594	64.04	[SHC3]
FLJ82076AAAF	673	74.27	[ZBTB16]
FLJ82077AAAF	338	38.12	[SLC25A40]
FLJ82079AAAF	660	71.97	[NEDD1]
FLJ82081AAAF	179	20.5	[KLRD1]
FLJ82082AAAF	670	74	[PRAM1]
FLJ82083AAAF	579	62.13	[RELB]
FLJ82086AAAF	217	25.01	[RAB39A]
FLJ82089AAAF	157	17.56	[C10orf53]
FLJ82090AAAF	923	99.03	[HK3]
FLJ82091AAAF	1159	126.23	[PCDH17]
FLJ82092AAAF	61	6.14	[MT1M]
FLJ82093AAAF	300	33.33	[SURF1]
FLJ82095AAAF	1154	128.56	[PDZD8]

FLJ82096AAAF	709	79.66	[APPL1]
FLJ82097AAAF	521	57.89	[KPNA4]
FLJ82098AAAF	525	58.16	[STAM2]
FLJ82099AAAF	405	46.91	[NXPE1]
FLJ82100AAAF	65	7.43	[SERP2]
FLJ82102AAAF	74	8.5	[GNGT1]
FLJ82103AAAF	66	7.81	[CCDC23]
FLJ82104AAAF	207	22.33	[TM2D1]
FLJ82105AAAF	129	14.32	[PFN4]
FLJ82106AAAF	313	36.72	[C11orf65]
FLJ82107AAAF	145	16.45	[ZFAND2A]
FLJ82110AAAF	293	33.81	[OTUD6B]
FLJ82111AAAF	116	12.83	[CARTPT]
FLJ82112AAAF	200	22.28	[MS4A5]
FLJ82113AAAF	226	26.69	[UBE2U]
FLJ82116AAAF	247	27.66	[GZMB]
FLJ82118AAAF	257	28.06	[NR0B2]
FLJ82119AAAF	384	42.2	[RAD51B]
FLJ82120AAAF	438	49.37	[NTNG1]
FLJ82121AAAF	197	21.56	[MYL4]
FLJ82122AAAF	291	33.04	[GTF2E2]
FLJ82123AAAF	902	102.66	[RBBP8]
FLJ82124AAAF	589	65.47	[PLBD2]
FLJ82129AAAF	500	55.49	[FAM83F]
FLJ82131AAAF	520	59.61	[DYRK4]
FLJ82132AAAF	141	16.4	[VAMP4]
FLJ82134AAAN	117	12.25	[POLE4]
FLJ82135AAAF	212	24.08	[SENP8]
FLJ82136AAAF	315	33.85	[SLC25A18]
FLJ82137AAAF	253	28.31	[CIDEA]
FLJ82138AAAF	206	23.86	[C7orf61]
FLJ82140AAAF	90	10.33	[TIMM10]
FLJ82141AAAF	163	17.63	[RPP25L]
FLJ82144AAAF	131	14.03	[LYNX1]
FLJ82145AAAF	399	45.73	[AADAC]
FLJ82146AAAF	147	16.86	[FHIT]
FLJ82147AAAF	712	78.65	[TAB3]
FLJ82148AAAF	304	35.25	[DTWD1]
FLJ82149AAAF	346	38.8	[KHDRBS3]
FLJ82150AAAF	130	12.41	[IFI27L2]
FLJ82152AAAN	411	43.25	[MBD2]
FLJ82154AAAF	153	17.21	[C20orf144]
FLJ82156AAAF	223	25.16	[PPAPDC1B]
FLJ82157AAAF	418	47.96	[KCNJ16]
FLJ82158AAAF	347	38.09	[CD5L]
FLJ82159AAAF	548	60.77	[SVOP]
FLJ82160AAAF	452	51.36	[TIMM44]
FLJ82162AAAF	238	27.03	[DOLPP1]

FLJ82163AAAF	198	22.17	[BCL2L11]
FLJ82165WAAF	549	63.24	[TIGD7]
FLJ82166AAAF	140	15.73	[HDDC3]
FLJ82170AAAF	158	18.36	[LMO2]
FLJ82171AAAF	872	99.84	[DNAH2]
FLJ82174AAAF	331	37.49	[PARVG]
FLJ82175AAAF	360	41.45	[BVES]
FLJ82177AAAF	251	27.84	[BRI3BP]
FLJ82178AAAF	564	60.07	[KRT6B]
FLJ82180AAAF	442	52.65	[FBXO39]
FLJ82181AAAF	466	52.81	[KATNAL2]
FLJ82183AAAF	1148	128.25	[RFC1]
FLJ82184AAAF	508	57.65	[GSDMC]
FLJ82185AAAF	153	16.72	[AKR7L]
FLJ82186AAAF	410	47.08	[CCNE1]
FLJ82187AAAF	324	37.62	[KIAA0825]
FLJ82188AAAF	1195	133.32	[MTMR4]
FLJ82189AAAF	220	23.98	[TP53INP2]
FLJ82190AAAF	224	24.11	[CBLN2]
FLJ82191AAAF	241	27.49	[RCAN3]
FLJ82192AAAF	217	25.34	[GRAP]
FLJ82194AAAF	444	50.87	[XK]
FLJ82195AAAF	1092	120.35	[USPL1]
FLJ82196AAAF	922	105.2	[WWP1]
FLJ82197AAAF	139	15.61	[GAGE1]
FLJ82198AAAF	88	9.5	[SNN]
FLJ82199AAAF	202	21.89	[IL17D]
FLJ82200AAAF	359	40.48	[CHAD]
FLJ82202AAAF	442	49.1	[KLHDC10]
FLJ82204AAAF	299	34.15	[SETD9]
FLJ82205AAAF	261	29.73	[LRRC18]
FLJ82206AAAF	290	34.02	[TP53TG5]
FLJ82207AAAF	252	28.02	[CRYBB1]
FLJ82209AAAF	327	37.21	[FKBP6]
FLJ82210AAAF	459	50.66	[STPG2]
FLJ82211AAAF	240	26.93	[LOC388882]
FLJ82213AAAF	215	22.31	[LOC439951]
FLJ82214AAAF	414	44.71	[YY1]
FLJ82217AAAF	402	46.18	[NKAPL]
FLJ82220AAAF	162	17.91	[LY86]
FLJ82221AAAF	589	63.52	[CSRNP1]
FLJ82222AAAF	170	18.46	[TPPP2]
FLJ82225AAAF	181	20	[TMEM47]
FLJ82227AAAF	444	49.3	[VPS4B]
FLJ82228AAAF	353	39.76	[PNMA1]
FLJ82229AAAF	227	23.83	[SPATA25]
FLJ82230AAAF	78	8.4	[SFTA2]
FLJ82231AAAF	126	14.27	[PATE1]

FLJ82232AAAF	555	62.56	[SLC22A2]
FLJ82233AAAF	164	19.63	[IQCF2]
FLJ82234AAAF	834	92.86	[NEDD9]
FLJ82235AAAF	751	85.5	[CCDC57]
FLJ82236AAAF	1158	126.97	[NRIP1]
FLJ82241AAAF	259	29.27	[LRRC3B]
FLJ82242AAAF	208	23.52	[RAB15]
FLJ82243AAAF	701	80.36	[ALOX12B]
FLJ82244AAAF	178	19.34	[NPM3]
FLJ82245AAAF	646	71.9	[ABCG4]
FLJ82246AAAF	943	106.87	[CENPC]
FLJ82248AAAF	240	25.54	[TMEM65]
FLJ82252AAAF	271	28.84	[AQP2]
FLJ82253AAAF	525	61.41	[ZNF678]
FLJ82255AAAF	265	31.39	[ZDHHC21]
FLJ82257AAAF	545	60.62	[TCERG1L]
FLJ82258AAAF	545	61.96	[ZNF77]
FLJ82259AAAF	330	37.49	[GFI1B]
FLJ82260AAAF	452	53	[ETV6]
FLJ82261AAAF	303	33.78	[CRKL]
FLJ82262AAAF	209	22.07	[C2orf27B]
FLJ82263AAAF	541	60.34	[RFT1]
FLJ82264AAAF	283	31.54	[TSPAN33]
FLJ82265AAAF	241	27.59	[DNAJC4]
FLJ82266AAAF	507	55.64	[MKRN3]
FLJ82267AAAF	406	44.9	[HS3ST3A1]
FLJ82268AAAF	703	76.28	[CASC3]
FLJ82269AAAF	258	31.04	[CCDC172]
FLJ82270AAAF	218	23.5	[LINC01547]
FLJ82271AAAF	530	60.71	[MICU3]
FLJ82272AAAF	412	44.59	[PRR30]
FLJ82273WAAF	257	28.04	[EMX1]
FLJ82274AAAF	450	52.31	[CIR1]
FLJ82276AAAF	421	45.25	[FOXJ1]
FLJ82278AAAF	605	69.17	[ZNF354A]
FLJ82279AAAN	1217	141.51	[SMC3]
FLJ82280AAAF	187	21.64	[CIB2]
FLJ82281AAAF	299	32.56	[PPP1R3D]
FLJ82283AAAF	222	23.97	[CYB561D2]
FLJ82284AAAF	74	8.38	[COA5]
FLJ82286AAAF	361	40.88	[QPCT]
FLJ82287AAAF	690	77.67	[NKRF]
FLJ82288AAAF	500	57.45	[TRIM69]
FLJ82289AAAF	168	18.75	[HRASLS]
FLJ82292AAAF	388	44.17	[ANGPTL5]
FLJ82294AAAF	164	18.53	[NUDT10]
FLJ82295AAAF	346	36.97	[USF2]
FLJ82298AAAF	697	80.53	[LCA5]

FLJ82300AAAF	657	76.16	[TIPARP]
FLJ82301AAAF	508	57.36	[MMP19]
FLJ82302AAAF	437	46.92	[E2F1]
FLJ82305AAAF	353	38.1	[CRELD2]
FLJ82306AAAF	373	41.05	[TPRA1]
FLJ82307AAAF	713	82.02	[ZBTB1]
FLJ82308AAAF	163	18.7	[JDP2]
FLJ82309AAAF	456	52.07	[AGPAT6]
FLJ82312AAAF	251	28.39	[CBX7]
FLJ82313AAAF	757	89.8	[PIBF1]
FLJ82315AAAF	257	26.89	[ALYREF]
FLJ82316AAAF	577	62.5	[TBL1X]
FLJ82318AAAF	201	21.59	[NCR3]
FLJ82319AAAF	275	29.95	[CENPV]
FLJ82320AAAF	595	65.12	[ZNF467]
FLJ82321AAAF	387	43.9	[CATIP]
FLJ82322AAAF	310	34.47	[FAM84B]
FLJ82323AAAF	1475	153.99	[NUP153]
FLJ82324AAAF	711	81.61	[ZNF175]
FLJ82325AAAF	565	59.33	[DEAF1]
FLJ82326WAAF	427	48.48	[ZNF134]
FLJ82327AAAF	193	21.86	[ZNF740]
FLJ82328AAAF	292	33.2	[BCDIN3D]
FLJ82329AAAF	114	12.49	[NRARP]
FLJ82330AAAF	359	41.29	[ST8SIA4]
FLJ82331AAAF	233	26.29	[KLRC1]
FLJ82332AAAF	242	27.75	[HOXC8]
FLJ82333AAAF	238	25.26	[LRRN4CL]
FLJ82334AAAF	258	29.17	[FRG1]
FLJ82337AAAF	154	17.8	[C16orf87]
FLJ82338AAAF	142	15.51	[HBQ1]
FLJ82339AAAF	463	53.44	[TRMT11]
FLJ82340AAAF	229	25.06	[DRICH1]
FLJ82341WAAF	446	50.38	[GPATCH4]
FLJ82342AAAF	963	107.5	[SH3BP4]
FLJ82343AAAF	210	22.55	[KRTAP26-1]
FLJ82344AAAF	139	15.88	[NR2C2AP]
FLJ82345AAAF	255	29.28	[FOLR2]
FLJ82346AAAF	166	19.17	[AGR3]
FLJ82347WAAF	185	20.33	[GKN1]
FLJ82348AAAF	132	14.64	[LGALS2]
FLJ82349AAAF	258	29.34	[TRIM40]
FLJ82351AAAF	832	93.01	[KAT2B]
FLJ82352AAAF	372	41.18	[PARD6B]
FLJ82354AAAF	131	14.23	[HIST1H2AA]
FLJ82356AAAF	361	39.28	[ANKRD16]
FLJ82357AAAF	384	42.11	[DUSP5]
FLJ82358AAAF	889	99.42	[PKN3]

FLJ82359AAAF	706	79.15	[ADD3]
FLJ82360AAAF	614	64.85	[KIAA0907]
FLJ82361AAAF	236	27.73	[SYCP3]
FLJ82362AAAF	142	16.08	[CST5]
FLJ82365AAAN	2511	273.4	[FASN]
FLJ82367AAAF	372	41.01	[TRIB1]
FLJ82368AAAF	431	47.16	[FOXN2]
FLJ82369AAAF	323	37.23	[DEGS2]
FLJ82370AAAF	257	29.4	[KCTD1]
FLJ82372AAAF	314	34.61	[FAHD2B]
FLJ82373AAAF	387	42.5	[SIRPG]
FLJ82374AAAF	479	54.63	[TFCP2L1]
FLJ82376AAAF	471	49.13	[IRX2]
FLJ82377AAAF	787	89.87	[STAT5B]
FLJ82378AAAF	142	14.15	[CHCHD10]
FLJ82379AAAF	579	63.71	[IGF2BP3]
FLJ82380AAAF	458	48.98	[SERGEF]
FLJ82382AAAF	726	80.82	[ADD2]
FLJ82383AAAF	195	22.7	[RD3]
FLJ82385AAAF	141	16.21	[CST4]
FLJ82386AAAF	241	26.53	[FBXL22]
FLJ82387AAAF	158	16.23	[MZT2B]
FLJ82388AAAF	139	16.12	[LGALS13]
FLJ82389AAAF	197	22.81	[AKAP14]
FLJ82391AAAF	135	15.24	[H3F3C]
FLJ82394AAAF	341	38.09	[RDH10]
FLJ82395AAAF	948	105.85	[ARHGEF1]
FLJ82396AAAF	1461	155	[PRX]
FLJ82397AAAF	483	55.04	[ZNF774]
FLJ82400AAAF	123	13.55	[MRPL52]
FLJ82403AAAF	255	29.26	[REEP3]
FLJ82405AAAF	498	56.58	[B3GALTL]
FLJ82406AAAF	452	48.15	[RCC1]
FLJ82407AAAF	522	56.26	[GSR]
FLJ82408AAAF	714	81.1	[KIAA1161]
FLJ82409AAAF	77	8.81	[LEAP2]
FLJ82410AAAF	107	12.44	[POLD4]
FLJ82411AAAF	92	10.15	[CCL4]
FLJ82412AAAF	261	29.32	[ALKBH2]
FLJ82413AAAF	436	49.59	[KCNG3]
FLJ82416AAAF	261	29.27	[CD40LG]
FLJ82419AAAF	929	105.23	[DSTYK]
FLJ82420AAAN	474	47.26	[SOX4]
FLJ82421AAAF	855	96.81	[BICD2]
FLJ82425AAAF	142	16.8	[GMFG]
FLJ82426AAAF	64	7.07	[TMA7]
FLJ82427AAAF	504	53.97	[TAPBP]
FLJ82430AAAF	362	37.76	[MESDC1]

FLJ82432AAAF	785	85.78	[JUP]
FLJ82433WAAF	456	53.78	[FAF2]
FLJ82434AAAF	219	24.98	[HDDC2]
FLJ82435AAAF	779	87	[MARK2]
FLJ82439WAAF	136	14.99	[LY6G6C]
FLJ82441WAAF	431	46.9	[ELK4]
FLJ82442SAAN	800	86.83	[TAF5]
FLJ82443AAAF	262	29.35	[BMF]
FLJ82445WAAF	184	20.44	[UBE2I]
FLJ82446WAAF	105	12.06	[NDUFB2]
FLJ82448WAAF	331	35.68	[JUN]
FLJ82449WAAF	154	17.33	[PFDN5]
FLJ82452WAAF	139	16.28	[UTS2]
FLJ82453WAAF	394	47.48	[CERS3]
FLJ82455AAAF	431	47.91	[P2RX6]
FLJ82456WAAF	489	52.41	[FOXG1]
FLJ82457AAAF	295	32.92	[MXI1]
FLJ82459WAAF	278	31.75	[TTPA]
FLJ82461WAAF	141	15.7	[IDNK]
FLJ82463AAAF	151	17.3	[TTC32]
FLJ82464AAAF	193	20.46	[AVP]
FLJ82469AAAF	186	20.48	[IFT27]
FLJ82470AAAF	102	11.84	[DDA1]
FLJ82471AAAF	345	38.8	[OGG1]
FLJ82472AAAF	134	14.7	[RAD51C]
FLJ82473AAAF	393	44.26	[KCNQ2]
FLJ82474AAAF	649	73.46	[RECQL]
FLJ82476AAAF	237	27.04	[CYB5R2]
FLJ82480AAAF	192	21.43	[RAC2]
FLJ82483AAAF	391	42.46	[RGP1]
FLJ82484AAAF	249	28.38	[GTF2F2]
FLJ82485AAAF	523	57.56	[EIF2B4]
FLJ82486AAAF	137	15.49	[C20orf24]
FLJ82487AAAF	277	29.99	[INSIG1]
FLJ82488AAAF	575	63.72	[MEN1]
FLJ82489AAAF	1064	120.72	[KDM4A]
FLJ82490AAAF	386	43.48	[DNMT3L]
FLJ82491AAAF	80	8.8	[FKBP1B]
FLJ82496AAAF	300	31.5	[DHRS2]
FLJ82497AAAF	288	32.57	[PPAP2C]
FLJ82499AAAF	184	20.35	[NDUFAF3]
FLJ82500AAAF	296	32.54	[FBXL15]
FLJ82501AAAF	308	32.8	[RIMS3]
FLJ82502AAAF	148	16.78	[PTP4A3]
FLJ82503AAAF	1126	118.69	[BAG6]
FLJ82508AAAF	87	9.26	[FXYD3]
FLJ82510AAAF	74	8.52	[ANAPC13]
FLJ82511AAAF	465	53.76	[IQCB1]

FLJ82512AAAF	170	19.46	[SSX3]
FLJ82513AAAF	93	10.83	[S100A8]
FLJ82514AAAF	289	32.74	[USP45]
FLJ82515AAAF	150	15.92	[NFKBID]
FLJ82516AAAF	170	18.87	[HRAS]
FLJ82517AAAF	477	54.68	[PPARG]
FLJ82519AAAF	48	5.22	[CSAG3]
FLJ82520AAAF	268	29.78	[COL2A1]
FLJ82521AAAF	213	22.08	[CDV3]
FLJ82522AAAF	217	24.06	[OSTF1]
FLJ82528AAAF	108	11.87	[TMEM141]
FLJ82529AAAF	198	22.09	[DENR]
FLJ82532AAAF	162	17.7	[BTF3]
FLJ82536AAAF	219	24.43	[PDE4D]
FLJ82542AAAF	289	33.23	[CCDC137]
FLJ82544WAAF	81	9.08	[XAGE1E]
FLJ82545AAAF	91	10.74	[LYRM4]
FLJ82546AAAF	335	35.43	[GATA1]
FLJ82548AAAF	342	39.42	[CFAP36]
FLJ82549AAAF	175	18.83	[CMTM7]
FLJ82551AAAF	647	70.03	[ASIC4]
FLJ82553AAAF	121	13.51	[HHLA3]
FLJ82554AAAF	342	39.5	[OSMR]
FLJ82555AAAF	744	81.53	[TRIM2]
FLJ82558AAAF	127	14.68	[ATP5S]
FLJ82559AAAF	233	24.99	[LAT]
FLJ82564AAAF	621	71.81	[KIF1BP]
FLJ82565AAAF	313	34.33	[THAP11]
FLJ82569AAAF	522	59.81	[CENPI]
FLJ82571AAAF	196	23	[GINS1]
FLJ82574AAAF	156	17.44	[CMTM5]
FLJ82576AAAF	188	21.43	[KRAS]
FLJ82577AAAF	323	36.98	[PPP1CC]
FLJ82579AAAF	367	39.55	[ELAVL3]
FLJ82580AAAF	192	21.18	[BAX]
FLJ82581AAAF	533	60.09	[PARP3]
FLJ82584AAAF	776	86.83	[RASAL1]
FLJ82586AAAF	181	19.81	[CD160]
FLJ82587WAAF	337	37.41	[SLC9A3R2]
FLJ82589AAAF	81	9.06	[ABRACL]
FLJ82590AAAF	290	31.99	[CBX4]
FLJ82591AAAF	293	32.19	[MPG]
FLJ82592AAAF	202	23.2	[JOSD1]
FLJ82594AAAF	585	67.16	[RTF1]
FLJ82595AAAF	224	24.96	[SLC26A1]
FLJ82596AAAF	345	37.32	[PARD6A]
FLJ82597AAAF	165	17.81	[PPP1R14C]
FLJ82598AAAF	121	14.27	[MAP1LC3A]

FLJ82599AAAF	354	40.79	[CCNK]
FLJ82600AAAF	479	53.47	[PAIP1]
FLJ82601AAAF	240	26.26	[DLX4]
FLJ82602AAAF	753	81.87	[CNOT3]
FLJ82605SAAF	877	96.79	[MED16]
FLJ82607AAAF	454	51.86	[CDR2]
FLJ82608AAAF	302	33.27	[ZBTB32]
FLJ82609AAAF	887	101.25	[OSBPL3]
FLJ82614AAAF	81	8.93	[C8orf22]
FLJ82619AAAF	182	21.51	[CBFB]
FLJ82628SAAF	265	30.08	[POU5F1]
FLJ82630AAAN	374	43.52	[CCNE2]
FLJ82632AAAF	911	100.68	[GTF3C2]
FLJ82633AAAF	302	34.02	[NMNAT2]
FLJ82636AAAF	193	20.77	[BCL2L2]
FLJ82638AAAF	72	8.01	[C5orf46]
FLJ82639AAAF	387	42.41	[PARK2]
FLJ82640AAAF	75	7.82	[ENHO]
FLJ82643AAAF	317	35.27	[OR51E1]
FLJ82644AAAF	252	28.05	[NXPH3]
FLJ82646AAAF	138	14.7	[KISS1]
FLJ82647AAAF	181	21.25	[GSTM1]
FLJ82648AAAF	313	35.82	[STYXL1]
FLJ82650AAAF	117	13.29	[POLR2J]
FLJ82653AAAF	125	13.83	[PHPT1]
FLJ82655AAAF	778	89.09	[FAM47A]
FLJ82657AAAF	377	42.91	[TYR]
FLJ82659AAAF	335	37.99	[IL5RA]
FLJ82662AAAF	529	59.98	[PDP2]
FLJ82664AAAF	370	43.33	[TRAM2]
FLJ82665AAAF	1163	111.9	[COL3A1]
FLJ82666AAAF	545	59.94	[MIER2]
FLJ82668AAAF	515	56.83	[SLC7A6]
FLJ82672AAAF	635	72.19	[DMD]
FLJ82673AAAF	381	42.39	[IL12RB1]
FLJ82675AAAN	138	15.89	[APITD1]
FLJ82679AAAF	119	12.66	[EXOC3-AS1]
FLJ82685AAAF	150	15.44	[SLC22A18AS]
FLJ82687AAAF	475	53.91	[NR6A1]
FLJ82689AAAF	346	38.02	[ELAVL2]
FLJ82691AAAF	315	35.89	[NGDN]
FLJ82693WAAF	745	85.28	[CTAGE1]
FLJ82694AAAF	265	27.12	[MBLAC1]
FLJ82695AAAF	422	49.49	[ALS2CR12]
FLJ82697AAAF	310	34.04	[FCGR2B]
FLJ82699AAAF	121	13.44	[TAC3]
FLJ82703AAAF	135	15.28	[CISD2]
FLJ82704AAAF	299	33.2	[CD300A]

FLJ82705AAAF	418	44.79	[EVL]
FLJ82706AAAF	127	15.01	[TMEM18]
FLJ82708AAAF	230	27.09	[MRPL47]
FLJ82710AAAF	1101	121.81	[SECISBP2L]
FLJ82714AAAF	419	45.9	[VAT1L]
FLJ82715AAAF	358	40.36	[SPO11]
FLJ82716AAAF	384	43.15	[RALGPS1]
FLJ82717AAAF	673	74.01	[TNXB]
FLJ82719AAAF	269	29.26	[HRASLS5]
FLJ82720AAAF	695	76.23	[AMPH]
FLJ82722AAAF	370	43.09	[LPGAT1]
FLJ82723AAAF	211	22.59	[LYPD4]
FLJ82724AAAF	346	40.17	[RRAGB]
FLJ82725AAAF	659	73.54	[SLC6A16]
FLJ82726AAAF	1103	122.95	[KIF1C]
FLJ82727AAAF	883	99.9	[GABBR2]
FLJ82730AAAF	538	59.27	[DOLK]
FLJ82731AAAF	1023	112.6	[STARD8]
FLJ82738AAAF	716	78.91	[ARNT2]
FLJ82739AAAF	786	87.99	[SUPV3L1]
FLJ82740AAAF	1178	131.67	[SMG7]
FLJ82742AAAN	627	70.6	[CNTN1]
FLJ82743AAAF	991	111.29	[CCP110]
FLJ82746AAAF	240	25.78	[LKAAEAR1]
FLJ82748AAAF	284	32.15	[GTPBP8]
FLJ82749AAAF	738	83.73	[FAM160B1]
FLJ82750AAAF	931	106.2	[RAG1]
FLJ82751WAAF	160	17.79	[ADAT2]
FLJ82753AAAF	234	25.31	[PRR16]
FLJ82754AAAF	559	58.95	[MAP2]
FLJ82757AAAF	444	52.05	[RAPGEF5]
FLJ82760AAAF	913	101.89	[USP20]
FLJ82762AAAF	284	32.16	[PPAP2A]
FLJ82763AAAF	206	23.57	[RALA]
FLJ82764AAAF	356	39.27	[LHX8]
FLJ82767AAAF	255	26.79	[AQP8]
FLJ82768AAAF	227	25.55	[IL23R]
FLJ82770AAAF	97	11.1	[PYY]
FLJ82772AAAF	604	70.22	[ZSCAN12]
FLJ82774AAAF	96	10.78	[FITM1]
FLJ82776AAAF	396	42.79	[CTSE]
FLJ82777AAAF	288	32.6	[SH3GL3]
FLJ82780AAAF	828	94.1	[USP6NL]
FLJ82784AAAF	356	40.97	[KCNA2]
FLJ82785AAAF	288	32.21	[ABHD17B]
FLJ82786AAAF	523	62.2	[PKD2L2]
FLJ82787AAAF	422	46.3	[CRLF1]
FLJ82789AAAF	1008	113.86	[HLTF]

FLJ82793AAAF	263	30.06	[NUDT13]
FLJ82800AAAF	707	80.67	[SETDB2]
FLJ82802AAAF	459	52.37	[CDR2L]
FLJ82803AAAF	105	12.05	[GALNT1]
FLJ82805AAAF	358	41.33	[SKAP1]
FLJ82812AAAF	576	63.22	[MGRN1]
FLJ82813AAAF	1050	116.98	[ANKRD27]
FLJ82815AAAF	957	103.24	[MAGEE1]
FLJ82816AAAF	208	23.68	[ABCC5]
FLJ82822AAAF	106	12.49	[CMC1]
FLJ82823AAAF	128	15.13	[MRPL55]
FLJ82824AAAF	406	44.11	[LCOR]
FLJ82825AAAF	836	92.16	[CSF3R]
FLJ82826SAAF	682	73.42	[IRAK1]
FLJ82831AAAF	228	26.21	[PPP1R42]
FLJ82832AAAN	1141	123.69	[SREBF2]
FLJ82835WAAF	315	36.64	[NCOA5]
FLJ82840AAAN	587	62.67	[TCF7L1]
FLJ82843AAAF	84	9.82	[DEFB119]
FLJ82847AAAF	163	18.04	[LCN6]
FLJ82851AAAF	1299	139.07	[NFRKB]
FLJ82852AAAF	1066	118.88	[ZBTB21]
FLJ82855AAAF	428	47.19	[HCFC1]
FLJ82860AAAF	460	51.23	[SLC45A2]
FLJ82861AAAF	688	72.93	[KIRREL2]
FLJ82862AAAF	640	72.34	[MYB]
FLJ82863AAAF	306	35.82	[CCDC50]
FLJ82865AAAF	1028	115.04	[KIF17]
FLJ82866AAAF	131	15.21	[TMEM60]
FLJ82867AAAF	123	12.71	[FAM168B]
FLJ82870AAAF	134	15.66	[STAG3L3]
FLJ82872AAAF	329	36.74	[TSPAN17]
FLJ82873AAAF	243	26.03	[C1orf174]
FLJ82879AAAF	209	23.53	[MXD4]
FLJ82881AAAF	636	73.71	[ZNF510]
FLJ82884AAAF	321	35.41	[ANKRD26P1]
FLJ82887AAAF	117	13.13	[MED11]
FLJ82890AAAF	140	15.81	[PTRHD1]
FLJ82891AAAF	187	21.13	[ALG1L]
FLJ82892AAAF	957	100.2	[PHC1]
FLJ82894AAAF	960	111.66	[OPA1]
FLJ82896AAAF	1532	174.76	[AGL]
FLJ82899AAAF	174	18.24	[ZNF385C]
FLJ82902AAAF	307	32.75	[TMEM200B]
FLJ82905AAAF	579	64.75	[ARNTL2]
FLJ82906AAAF	306	33.76	[NTHL1]
FLJ82915AAAF	141	15.01	[PIN4]
FLJ82918AAAF	183	20.83	[MANF]

FLJ82930WAAF	260	29.44	[XRCC6BP1]
FLJ82931AAAF	641	72.61	[HAUS5]
FLJ82942AAAF	319	35.81	[ASB4]
FLJ82945AAAF	245	27.41	[RNF151]
FLJ82949AAAF	326	37.27	[NODAL]
FLJ82956AAAF	813	92.32	[SRRT]
FLJ82960AAAF	1024	112.56	[ELMSAN1]
FLJ82962AAAF	1024	116.69	[SYCP2]
FLJ82963AAAF	385	42.16	[SSTR3]
FLJ82973WAAF	393	43.83	[ROR1]
FLJ82987SAAN	481	55.33	[LIPI]
FLJ82992SAAF	1212	131.45	[SLC12A2]
FLJ83001WAAF	109	12.63	[PMS2P3]
FLJ83004AAAF	76	8.5	[SNRPG]
FLJ83005AAAF	380	42.92	[NCK2]
FLJ83006AAAF	229	25.57	[PAFAH1B2]
FLJ83007AAAF	375	42.81	[LDB1]
FLJ83008AAAF	307	34.5	[UFD1L]
FLJ83009AAAF	115	13.02	[RPS26]
FLJ83011AAAF	151	15.51	[CHCHD2]
FLJ83012AAAF	314	34.14	[PITX1]
FLJ83013AAAF	222	23.85	[UBE2S]
FLJ83014AAAF	155	15.74	[ATP6V0C]
FLJ83016AAAF	63	7.31	[UQCR10]
FLJ83017AAAF	211	23.18	[CLTB]
FLJ83018AAAF	165	17.71	[RANGRF]
FLJ83020AAAF	268	31.77	[PITPNC1]
FLJ83022AAAF	137	14.49	[HRSP12]
FLJ83024AAAF	418	46.41	[TM7SF2]
FLJ83025AAAF	218	23.66	[CLTA]
FLJ83026AAAF	377	42.51	[NDUFA9]
FLJ83027AAAF	210	23.55	[STXBP6]
FLJ83029AAAF	213	23.5	[RAB25]
FLJ83031AAAF	434	47.31	[GPSM1]
FLJ83033AAAF	146	16.24	[MORN4]
FLJ83034AAAF	396	45.32	[ARC]
FLJ83035AAAF	1040	116.4	[DCLRE1A]
FLJ83036AAAF	118	13.13	[TCEB2]
FLJ83037AAAF	337	38.02	[GIMAP2]
FLJ83038AAAF	154	16.52	[ISCA2]
FLJ83039SAAN	132	13.9	[CDKN2A]
FLJ83040WAAF	175	19.66	[TPRKB]
FLJ83042AAAF	250	27.96	[IFI30]
FLJ83044AAAF	477	54.24	[KCNS2]
FLJ83045AAAF	489	54.65	[TULP1]
FLJ83046AAAF	849	98.17	[DIAPH3]
FLJ83048AAAF	280	29.79	[PRSS33]
FLJ83050AAAF	367	42.02	[ZDHHC2]

FLJ83052AAAF	390	44.08	[DAZ4]
FLJ83054AAAF	497	55.73	[CYP2D6]
FLJ83056WAAF	290	33.9	[NAT1]
FLJ83057WAAF	403	43.55	[ZCCHC3]
FLJ83058WAAF	148	17.01	[DNAJC24]
FLJ83059AAAF	409	46.43	[FAM20B]
FLJ83060AAAF	848	96.15	[AHR]
FLJ83063AAAF	217	23.47	[BCL7C]
FLJ83065AAAF	280	30.95	[PEX7]
FLJ83066AAAF	856	98.11	[KIF23]
FLJ83067AAAF	466	52.43	[SCARA3]
FLJ83070AAAF	235	26.5	[RGS20]
FLJ83072AAAF	61	6.6	[CD52]
FLJ83073AAAF	357	40.3	[PPIG]
FLJ83074AAAF	112	11.56	[TMEM14C]
FLJ83077AAAF	166	18.81	[THYN1]
FLJ83078AAAF	116	12.26	[TMEM261]
FLJ83081AAAF	220	24.98	[RAB3A]
FLJ83082AAAF	161	17.92	[TAF12]
FLJ83083AAAF	200	22.79	[TMEM126B]
FLJ83084AAAF	208	22.76	[MYL6B]
FLJ83086AAAF	404	45.29	[WSB2]
FLJ83089AAAF	176	19.88	[GPLD1]
FLJ83094AAAF	458	49.6	[SPAG6]
FLJ83095AAAF	70	8.28	[SHFM1]
FLJ83097AAAF	180	19.77	[BST2]
FLJ83102AAAF	538	59.15	[RBBP5]
FLJ83104AAAF	126	13.85	[MLLT10]
FLJ83108AAAF	144	16.44	[EIF1AY]
FLJ83109AAAF	266	29.94	[HLA-DRB4]
FLJ83114AAAF	160	18.12	[TWIST2]
FLJ83119AAAF	927	101.68	[LGR4]
FLJ83121AAAF	175	19.6	[H2BFWT]
FLJ83124AAAF	144	15.34	[CYSRT1]
FLJ83126AAAF	625	71.5	[TRAF3IP1]
FLJ83127AAAF	78	8.7	[CSAG1]
FLJ83128AAAF	71	8.05	[GTF2H5]
FLJ83133AAAF	102	12.13	[RPL13AP3]
FLJ83134AAAF	221	23.81	[CCDC144NL]
FLJ83136AAAF	189	21.15	[CT45A10]
FLJ83141WAAF	194	21.54	[BNIP3]
FLJ83142WAAF	176	18.05	[ARRDC1-AS1]
FLJ83146WAAF	101	10.57	[C6orf226]
FLJ83149AAAF	442	49.67	[FAM46A]
FLJ83150AAAF	147	16.76	[AVPI1]
FLJ83158AAAF	102	11.6	[DNM1P34]
FLJ83159AAAF	119	14.17	[ANKRD36BP1]
FLJ83166AAAF	158	17.78	[CXorf40B]

FLJ83176AAAF	226	24.61	[CCNL2]
FLJ83180AAAF	336	38.14	[KCNK1]
FLJ83188WAAF	133	14.14	[EEF1DP3]
FLJ83190WAAF	84	9.17	[ERICH1-AS1]
FLJ83195AAAF	123	13.3	[TYMSOS]
FLJ83199WAAF	105	11.58	[BAALC-AS2]
FLJ83205AAAF	82	9.66	[KLHL30-AS1]
FLJ83217AAAF	374	41.94	[ST6GALNAC2]
FLJ83221AAAF	109	12.21	[FAM72B]
FLJ83224WAAF	251	27.54	[ANKRD30BL]
FLJ83225AAAF	392	42.76	[RBMXL2]
FLJ83228AAAF	247	25.34	[SBSN]
FLJ83229WAAF	204	22.7	[MBD3L2]
FLJ83233WAAF	327	37.59	[ADAM5]
FLJ83234AAAF	261	28.59	[LRRK1]
FLJ83237AAAF	190	21.65	[OAZ3]
FLJ83238AAAF	549	62.3	[TBC1D3F]
FLJ83256WAAF	317	35.08	[GNB2L1]
FLJ83259WAAF	461	49.97	[RNH1]
FLJ83273WAAF	178	19.97	[NDUFB11]
FLJ83277WAAF	152	17.15	[NME1]
FLJ83279WAAF	386	42.26	[CUEDC1]
FLJ83284WAAF	219	23.26	[PSMB9]
FLJ83312WAAF	721	80.29	[MCM3AP]
FLJ83330AAAF	178	18.68	[ASPDH]
FLJ83337WAAF	294	32.58	[NPM1]
FLJ83345WAAF	212	23.34	[RAB43]
FLJ83346AAAF	114	13.22	[ZNF717]
FLJ83347AAAF	94	10.73	[LOC554249]
FLJ83348AAAF	250	28.15	[GDF5OS]
FLJ83358WAAF	142	14.67	[INAFM1]
FLJ83362AAAF	498	54.62	[NFIA]
FLJ83363AAAF	283	31.25	[MTAP]
FLJ83364AAAF	633	71.64	[EPB41L2]
FLJ83365AAAF	300	34.89	[EHF]
FLJ83367AAAF	1024	118.22	[TUBGCP5]
FLJ83371AAAF	505	57.48	[CHRNA3]
FLJ83373AAAF	274	29.65	[UQCRFS1]
FLJ83374AAAF	320	35.61	[EIF3G]
FLJ83376AAAF	1075	115.59	[NFATC3]
FLJ83380AAAF	310	33.93	[UPP1]
FLJ83381AAAF	1019	116.46	[LARP1]
FLJ83383AAAF	544	61.57	[ABLIM3]
FLJ83386AAAF	340	38.96	[EI24]
FLJ83387AAAF	452	50.7	[CASP2]
FLJ83389AAAF	919	102.97	[DHTKD1]
FLJ83391WAAF	292	31.59	[SDCBP2]
FLJ83392AAAF	330	35.38	[HOXC13]

FLJ83393AAAF	494	56.68	[SRSF4]
FLJ83401AAAF	777	86.19	[HGS]
FLJ83402AAAF	289	32.77	[ATPAF2]
FLJ83404AAAF	930	103.53	[RBM10]
FLJ83405AAAN	476	50.32	[TRIP6]
FLJ83406AAAF	400	46.55	[CYTH2]
FLJ83409AAAF	297	30.83	[QPRT]
FLJ83410AAAF	204	22.43	[TRPT1]
FLJ83411AAAF	366	39.04	[TARBP2]
FLJ83412AAAF	240	27.04	[CD58]
FLJ83413AAAF	312	35.3	[PHF6]
FLJ83414AAAN	669	72.76	[LZTS2]
FLJ83416AAAF	499	55.39	[INA]
FLJ83417AAAF	919	104.75	[EXTL3]
FLJ83419WAAF	1142	126.5	[SYMPK]
FLJ83421AAAF	444	48.05	[GATA3]
FLJ83424AAAF	341	38.21	[HSD17B7]
FLJ83426AAAF	468	51.53	[FOXN3]
FLJ83430AAAF	963	108.21	[IPO13]
FLJ83432AAAF	235	26.12	[TMEM176A]
FLJ83434AAAF	297	33.67	[SLC25A51]
FLJ83435AAAF	455	51.44	[ST6GAL2]
FLJ83437AAAF	1219	132.86	[TONSL]
FLJ83438AAAN	567	62.08	[PHF1]
FLJ83439AAAF	300	32.94	[ZC3HAV1L]
FLJ83440AAAF	770	86.69	[MYO19]
FLJ83441WAAF	270	28.31	[YRDC]
FLJ83442WAAF	311	34.82	[FAM175B]
FLJ83443AAAF	911	99.12	[BCAN]
FLJ83448AAAF	438	48.86	[OXA1L]
FLJ83449AAAF	359	39.59	[AKR7A2]
FLJ83456WAAF	529	56.56	[ATP5B]
FLJ83458AAAF	808	89.03	[TBL3]
FLJ83459AAAF	376	42	[C9orf114]
FLJ83460AAAF	252	27.6	[CDCA5]
FLJ83465AAAF	462	51.8	[MCRS1]
FLJ83468AAAF	335	38.02	[MARC2]
FLJ83471AAAF	367	41.97	[SEPT1]
FLJ83472AAAF	1031	117.36	[DDX46]
FLJ83473AAAF	717	83.01	[HOOK2]
FLJ83474AAAF	268	30.35	[R3HDM4]
FLJ83475WAAN	504	55.99	[SHMT2]
FLJ83477AAAF	324	34.61	[SIAH2]
FLJ83480AAAF	374	43.51	[GNA15]
FLJ83488AAAN	572	63.4	[PIAS2]
FLJ83492AAAF	451	51.77	[IRF4]
FLJ83495AAAF	715	78.21	[NCKIPSD]
FLJ83497AAAF	237	26.33	[LYPLAL1]

FLJ83499AAAF	407	42.15	[ADRM1]
FLJ83500AAAF	261	28.05	[SPR]
FLJ83501AAAF	680	77.86	[FERMT2]
FLJ83503AAAF	577	60.96	[CSTF2]
FLJ83504AAAN	431	47.18	[TFAP2A]
FLJ83510AAAF	1094	118.31	[SEC24C]
FLJ83511AAAF	326	34.61	[TMEM255B]
FLJ83514AAAF	203	23.26	[MTHFS]
FLJ83515AAAF	352	39.58	[TIMM50]
FLJ83517AAAF	584	63.75	[EYA2]
FLJ83522AAAF	245	26.95	[PYHIN1]
FLJ83526AAAN	903	101.25	[CNTROB]
FLJ83529AAAF	243	27.26	[CRISP2]
FLJ83530AAAF	1058	118.23	[DCAF1]
FLJ83531AAAF	326	36.59	[DCAF5]
FLJ83543AAAF	289	31.47	[TMEM163]
FLJ83544AAAF	594	66.9	[GAD1]
FLJ83547AAAF	690	78.33	[RASGRP3]
FLJ83550AAAF	304	32.22	[SLC25A34]
FLJ83551AAAF	525	59.12	[PPP3CB]
FLJ83552AAAF	619	68.28	[JAK3]
FLJ83553AAAF	767	86.37	[TBC1D16]
FLJ83558AAAF	150	16.54	[LTC4S]
FLJ83563AAAF	813	91.08	[MALT1]
FLJ83564AAAF	245	26.68	[WBSCR27]
FLJ83571AAAF	406	46.6	[ST6GAL1]
FLJ83578AAAF	716	77.98	[DVL3]
FLJ83579AAAF	461	51.64	[NR5A1]
FLJ83595AAAF	634	70.96	[CTTN]
FLJ83596SAAF	775	83.67	[GLIS3]
FLJ83604AAAF	349	40.06	[ERI1]
FLJ83610AAAF	224	25.03	[PRDX6]
FLJ83616AAAN	1377	154.98	[ZMYM2]
FLJ83617SAAN	1382	156.23	[NINL]
FLJ83619AAAF	680	75.68	[STKLD1]
FLJ83622AAAF	338	35.93	[FOSB]
FLJ83623AAAF	476	54.84	[RNMT]
FLJ83633AAAF	1068	122.31	[DAAM1]
FLJ83644AAAN	2002	226.69	[MYO18A]
FLJ83645AAAF	1118	123.04	[UPF1]
FLJ83648AAAF	885	100.83	[EXOSC10]
FLJ83654WAAF	789	87.41	[SF3B1]
FLJ83658AAAF	890	101.31	[TNPO1]
FLJ83659AAAN	483	53.41	[SRSF11]
FLJ83660AAAN	873	98.15	[ZHX1]
FLJ83663AAAF	511	56.96	[APLF]
FLJ83666AAAF	483	51.66	[NOXA1]
FLJ83669AAAF	522	56.08	[RCC2]

FLJ83675AAAF	912	101.86	[DNMT3A]
FLJ83676AAAN	1186	131.3	[PALB2]
FLJ83682WAAN	841	93.44	[SOX6]
FLJ83686AAAF	273	30.69	[OAF]
FLJ83690AAAN	755	81.49	[TRIM56]
FLJ83700AAAF	632	72.64	[ZDHHC17]
FLJ83702AAAF	1208	138.67	[TIMELESS]
FLJ83705AAAN	851	97.92	[STAT2]
FLJ83708SAAF	615	67.33	[NR2C2]
FLJ83710AAAF	569	63.37	[ZFP91]
FLJ83712AAAF	1122	121.99	[HDAC5]
FLJ83715AAAF	365	40.96	[VASH1]
FLJ83721AAAF	987	108.3	[EPHB4]
FLJ83726AAAF	857	97.67	[NOP14]
FLJ83727AAAF	666	73.18	[APBB1IP]
FLJ83728AAAF	694	75.48	[SAMD4B]
FLJ83729AAAF	521	55.94	[MEF2D]
FLJ83733AAAF	1369	155.28	[DHX29]
FLJ83736AAAF	359	42.14	[GNAQ]
FLJ83737AAAF	312	35.03	[CA4]
FLJ83746AAAF	443	52.3	[EOGT]
FLJ83748AAAF	259	28.57	[SYPL1]
FLJ83750AAAF	298	33.08	[FAM221A]
FLJ83757AAAF	337	38	[SFXN4]
FLJ83758AAAF	1664	177.6	[PPRC1]
FLJ83759AAAF	836	92.03	[BRD2]
FLJ83760AAAF	708	81.45	[NEK5]
FLJ83766AAAF	311	35.43	[MED27]
FLJ83768AAAF	303	32.09	[SUSD6]
FLJ83771AAAF	563	61.33	[MCCC2]
FLJ83774AAAF	1019	108.61	[COL6A2]
FLJ83776AAAN	1132	129.09	[MIS18BP1]
FLJ83777AAAF	1167	126.68	[ABL2]
FLJ83778SAAN	1271	142.79	[BCR]
FLJ83779AAAF	137	14.59	[RGCC]
FLJ83786AAAF	598	67.75	[WBSCR17]
FLJ83787AAAF	1009	112.91	[LIG3]
FLJ83788AAAF	252	28.53	[PIGL]
FLJ83792AAAF	856	98.08	[ATP6V0A2]
FLJ83797AAAF	643	73	[AMFR]
FLJ83799AAAF	990	111.66	[GOLGA2]
FLJ83802AAAN	681	75.59	[ESRP1]
FLJ83803AAAF	850	93	[PPP6R1]
FLJ83810AAAF	491	55.52	[NAMPT]
FLJ83815AAAF	543	57.51	[EGR1]
FLJ83817AAAF	1222	136.89	[GPR126]
FLJ83818AAAN	684	73.91	[DPYSL3]
FLJ83822AAAF	791	88.86	[H6PD]

FLJ83826AAAF	359	42.12	[GNA11]
FLJ83828WAAF	131	15.35	[TOR1AIP2]
FLJ83831WAAF	138	15.61	[LINC00116]
FLJ83835AAAF	860	99.55	[PDE6A]
FLJ83836WAAF	74	8.63	[SMIM15]
FLJ83837WAAF	186	21.12	[LINC00473]
FLJ83838AAAF	246	28.66	[RPL7L1]
FLJ83839WAAF	90	10.28	[TP53TG1]
FLJ83840WAAF	92	10.38	[GNRH1]
FLJ83841AAAF	92	10.04	[WT1-AS]
FLJ83843WAAN	574	62.39	[FOXJ2]
FLJ83854WAAF	326	35.86	[PIM3]
FLJ83864AAAF	136	14.96	[PMAIP1]
FLJ83868AAAF	448	50.34	[RARB]
FLJ83870WAAF	153	17.82	[DNASE2B]
FLJ83871WAAF	201	20.6	[TP73-AS1]
FLJ83875AAAF	226	24.47	[DMRT2]
FLJ83876WAAF	76	8.61	[CASC2]
FLJ83878AAAF	423	45.51	[ESRRA]
FLJ83883WAAF	32	3.7	[MIR17HG]
FLJ83885AAAF	365	41.08	[GPR68]
FLJ83888AAAF	819	89.17	[UNK]
FLJ83889SAAF	125	13.1	[NPB]
FLJ83898WAAF	312	35.03	[OR6B2]
FLJ83904WAAF	396	44.63	[BMP2]
FLJ83907SAAF	918	103.46	[IL6ST]
FLJ83909SAAF	156	17.83	[LMO1]
FLJ83910AAAF	698	77.67	[MTRR]
FLJ83913WAAF	136	15.4	[HIST1H3A]
FLJ83916SAAN	993	112.9	[FLT3]
FLJ83918WAAF	269	28.47	[CEBPD]
FLJ83919AAAF	322	34.94	[PURA]
FLJ83920WAAF	246	27.69	[SIX6]
FLJ83922SAAF	722	80.46	[BRD4]
FLJ83923AAAF	306	34.98	[TAAR2]
FLJ83932WAAF	128	13.94	[HIST1H2AJ]
FLJ83933WAAN	669	71.46	[HDAC10]
FLJ83934WAAF	240	25.39	[MGARP]
FLJ83936WAAN	397	41.33	[GATA5]
FLJ83938AAAF	190	20.48	[SPATA12]
FLJ83944AAAF	329	36.74	[SORBS3]
FLJ83945AAAF	335	37.52	[SPDEF]
FLJ83949AAAF	784	83.48	[E4F1]
FLJ83952AAAF	304	33.81	[HOXC11]
FLJ83954AAAF	58	7	[POLR2K]
FLJ83965AAAF	328	34.49	[SOHLH1]
FLJ83969AAAF	59	6.66	[RHPN1-AS1]
FLJ83973AAAF	208	23.88	[C10orf107]

FLJ83974AAAF	674	76.86	[DBF4]
FLJ83975AAAF	288	33.24	[STX1B]
FLJ83976AAAF	53	5.94	[IGIP]
FLJ83989AAAF	414	46.42	[SCRN1]
FLJ83991AAAF	297	32.35	[LYSMD4]
FLJ83993AAAF	293	31.21	[METRN]
FLJ83996AAAF	190	21.88	[NCS1]
FLJ83999AAAF	421	48.18	[BEND5]
FLJ84001AAAF	205	23.58	[TVP23B]
FLJ84002AAAF	355	37.63	[SHOX2]
FLJ84003AAAF	348	38.59	[RSPH14]
FLJ84008AAAN	1264	140.47	[VARS]
FLJ84009AAAF	280	32.19	[DAPP1]
FLJ84012AAAF	376	41.14	[GLI4]
FLJ84015AAAF	366	42.08	[GKAP1]
FLJ84016AAAF	316	35.91	[TAMM41]
FLJ84017AAAF	309	33.79	[UPRT]
FLJ84018AAAF	298	34.69	[OTULIN]
FLJ84020AAAN	588	65.83	[TRIM29]
FLJ84023AAAF	272	31.26	[SRSF5]
FLJ84028AAAF	435	50.65	[TEKT4]
FLJ84029AAAF	322	36.23	[SFXN2]
FLJ84031AAAF	622	69.19	[PCK1]
FLJ84035AAAN	640	72.35	[ELL2]
FLJ84038WAAF	281	32.18	[RBM11]
FLJ84039AAAN	509	58.02	[IKZF3]
FLJ84042AAAF	215	23.46	[LYSMD2]
FLJ84044AAAF	307	34.32	[CCDC24]
FLJ84053AAAF	1094	121.35	[AP3B1]
FLJ84054AAAN	680	76.79	[TP63]
FLJ84055SAAF	1077	120.55	[RAPGEF1]
FLJ84058AAAF	199	22.75	[COMMD2]
FLJ84060WAAF	242	25.89	[FAM102A]
FLJ84071AAAF	293	32.6	[C1orf131]
FLJ84072AAAF	173	18.16	[LAMTOR5]
FLJ84073AAAF	195	22.52	[FAM122C]
FLJ84074AAAF	429	49.98	[POFUT2]
FLJ84078AAAF	249	27.01	[BPIFA2]
FLJ84079AAAF	478	51.92	[RGMB]
FLJ84080AAAF	711	77.47	[MAPK8IP1]
FLJ84089AAAF	349	38.18	[FKBPL]
FLJ84091AAAF	237	26.12	[RNF166]
FLJ84097AAAN	508	51.59	[SRF]
FLJ84100AAAF	390	44.65	[NCF1]
FLJ84101AAAF	217	22.85	[CLDN9]
FLJ84105AAAF	270	30.12	[CCSAP]
FLJ84106WAAF	268	30.69	[CENPQ]
FLJ84108AAAF	567	65.07	[RABGGTA]

FLJ84109AAAN	689	78.55	[PTCD3]
FLJ84113AAAF	1220	138.8	[EIF5B]
FLJ84115AAAN	956	106.57	[AMOTL1]
FLJ84125AAAF	272	29.77	[PSMB8]
FLJ84127AAAF	318	35.51	[COQ9]
FLJ84128AAAF	959	108.31	[ARHGEF2]
FLJ84132AAAF	453	50.49	[DCAF12]
FLJ84134AAAF	364	41.74	[OAS1]
FLJ84142AAAF	225	24.78	[INSIG2]
FLJ84145AAAF	249	28.39	[TRIM74]
FLJ84148AAAF	152	17.99	[SCAND2P]
FLJ84150AAAF	345	41.15	[MSANTD4]
FLJ84152AAAF	362	40.8	[NECAB3]
FLJ84155AAAN	1024	113.91	[PHF8]
FLJ84156AAAF	222	25.79	[FKBP7]
FLJ84157AAAF	351	38.42	[GPD1L]
FLJ84160AAAF	457	50.43	[POLM]
FLJ84161AAAF	388	43.83	[DGAT2]
FLJ84163AAAF	294	31.57	[VDAC2]
FLJ84170AAAF	775	85.06	[TAF1C]
FLJ84171AAAF	479	51.64	[FBXL16]
FLJ84186AAAF	199	21.91	[C1orf50]
FLJ84189AAAF	302	32.72	[MFI2]
FLJ84190AAAF	318	33.92	[NFYA]
FLJ84195AAAF	430	48.39	[MYO5C]
FLJ84198AAAF	425	47.08	[PAICS]
FLJ84200AAAF	267	30.26	[OVOL1]
FLJ84201AAAF	484	54.89	[ADSL]
FLJ84203AAAF	196	20.64	[ANAPC11]
FLJ84204AAAF	230	24.59	[DHRS4L2]
FLJ84205AAAF	243	27.3	[NUCKS1]
FLJ84206AAAF	326	36.71	[NUP37]
FLJ84210AAAF	312	35.11	[UBXN1]
FLJ84212AAAF	260	28.97	[TMEM70]
FLJ84213AAAF	416	46.48	[APMAP]
FLJ84216AAAN	911	104.85	[ACTN4]
FLJ84217AAAF	327	36.5	[AEN]
FLJ84218AAAF	262	28.92	[KLK1]
FLJ84221AAAF	855	100.01	[XAB2]
FLJ84225AAAF	255	28.01	[PGAM5]
FLJ84228AAAF	360	41.28	[MRPS22]
FLJ84229AAAF	475	54.85	[LTV1]
FLJ84231AAAF	352	39.24	[PCGF6]
FLJ84234AAAF	273	28.78	[MVB12A]
FLJ84237AAAF	704	78.92	[MTMR8]
FLJ84240WAAF	212	22.74	[PPIC]
FLJ84242AAAF	183	20.83	[MFAP2]
FLJ84244AAAF	243	27.94	[RWDD1]

FLJ84245AAAF	258	27.93	[IGFBP4]
FLJ84246AAAF	226	24.91	[METTL21B]
FLJ84247AAAF	238	26.75	[CIDEC]
FLJ84248AAAF	522	55.67	[NRF1]
FLJ84252AAAF	310	34.68	[NTAN1]
FLJ84253AAAF	328	35.76	[ECH1]
FLJ84255AAAF	257	28.02	[ZFAND2B]
FLJ84256AAAF	406	44.51	[RBMS1]
FLJ84257AAAF	270	30.54	[UBXN8]
FLJ84259AAAF	207	23.1	[SPRYD4]
FLJ84260AAAF	189	20.69	[MZB1]
FLJ84262AAAF	238	27.37	[SRSF7]
FLJ84264AAAF	238	24.89	[PDPN]
FLJ84265AAAF	226	24.37	[TMEM86B]
FLJ84266AAAF	318	35.96	[GGH]
FLJ84269AAAF	240	26.96	[UNC119]
FLJ84271AAAF	485	52.35	[CELF5]
FLJ84272AAAF	284	31.94	[ICMT]
FLJ84273AAAF	367	39.12	[DMRTC2]
FLJ84274AAAF	282	31.43	[DNASE1]
FLJ84276AAAF	600	68.5	[DDX55]
FLJ84277AAAF	292	33.53	[MRPL19]
FLJ84278AAAF	401	43.04	[TGIF1]
FLJ84281AAAF	328	34.24	[DLX2]
FLJ84285AAAF	335	38.3	[ARHGEF39]
FLJ84289AAAF	409	47.19	[C9orf41]
FLJ84293AAAF	192	20.46	[RNF185]
FLJ84294AAAF	466	53	[CTDSPL2]
FLJ84298AAAF	379	44.01	[RIBC1]
FLJ84300AAAN	504	54.46	[YAP1]
FLJ84302AAAF	323	36.79	[AKR1C1]
FLJ84304AAAF	865	96.98	[CEP97]
FLJ84310WAAF	307	35.94	[SFR1]
FLJ84311AAAF	333	37	[MTFR1]
FLJ84314AAAF	198	23.08	[TMEM17]
FLJ84315AAAF	1494	166.8	[RALGAPB]
FLJ84316AAAF	580	64.03	[ARHGEF25]
FLJ84321AAAF	379	42.19	[TMEM173]
FLJ84323AAAF	360	42.4	[DNAJC25]
FLJ84326AAAF	577	63.18	[STAU1]
FLJ84327AAAF	386	43.82	[VPS36]
FLJ84333AAAF	272	30.3	[PRKAB2]
FLJ84334AAAF	783	85.94	[PNPT1]
FLJ84335AAAF	1085	124.27	[SRGAP1]
FLJ84341AAAF	828	92.45	[FAM171B]
FLJ84343AAAF	437	49.6	[BYSL]
FLJ84345AAAF	400	43.99	[DOC2A]
FLJ84347AAAF	510	55.22	[CNOT11]

FLJ84348AAAN	828	89.08	[PPP1R13L]
FLJ84356AAAF	343	37.64	[C5orf45]
FLJ84361AAAF	1123	122.81	[USP36]
FLJ84362AAAF	220	24.4	[TIMP2]
FLJ84367AAAF	703	76.72	[ATF6B]
FLJ84369AAAF	379	44.41	[FNTA]
FLJ84370AAAF	245	27.75	[YWHAZ]
FLJ84375AAAF	853	98.24	[PDE6B]
FLJ84378AAAF	424	48.49	[PACSIN3]
FLJ84381AAAF	294	31.58	[YIF1B]
FLJ84382AAAF	847	94.62	[MATR3]
FLJ84386AAAF	823	92	[NLGN1]
FLJ84387AAAF	321	36.75	[RASSF4]
FLJ84388WAAF	403	47.35	[TRMT10C]
FLJ84394AAAF	1208	132.82	[AMBRA1]
FLJ84399AAAF	756	82.44	[RAB11FIP3]
FLJ84402WAAF	1127	125.49	[TP53BP2]
FLJ84404AAAF	745	85.57	[EXOC3]
FLJ84405AAAF	1221	140.09	[STAG1]
FLJ84406AAAF	339	38.6	[ANXA2]
FLJ84411AAAF	1055	122.22	[USP25]
FLJ84413AAAF	419	46.4	[RNF130]
FLJ84424AAAF	365	41.78	[GABRE]
FLJ84425AAAF	266	29.77	[DRAM2]
FLJ84431WAAF	259	26.72	[CHMP1A]
FLJ84436AAAF	662	73.87	[BMP2K]
FLJ84442AAAF	453	52.04	[PPP2R2D]
FLJ84455AAAF	457	49.84	[PDLIM7]
FLJ84457AAAF	179	20.73	[ARL5A]
FLJ84458AAAF	437	51.56	[SNRNP70]
FLJ84462AAAF	263	29.41	[C1orf35]
FLJ84463AAAF	267	29.66	[MMP7]
FLJ84471AAAF	253	30.45	[UTP11L]
FLJ84472AAAF	419	47.16	[CPA1]
FLJ84473AAAF	303	34.27	[SEC22C]
FLJ84476AAAF	41	4.68	[LOC84843]
FLJ84488AAAF	337	37.47	[MARC1]
FLJ84489AAAF	313	35.24	[DIMT1]
FLJ84490AAAF	390	43.53	[CHI3L2]
FLJ84491WAAF	463	48.91	[FOXA2]
FLJ84492AAAF	322	35.74	[SLC35B1]
FLJ84494AAAF	271	30.04	[COMT]
FLJ84495AAAF	913	101.65	[PCSK5]
FLJ84501AAAF	417	47.97	[MTERF3]
FLJ84502AAAF	400	45.15	[WDFY2]
FLJ84507AAAF	58	6.89	[SMIM11]
FLJ84508AAAF	198	21.1	[FAM219B]
FLJ84509AAAF	464	50.82	[NDUFV1]

FLJ84510AAAF	469	51.28	[CFP]
FLJ84512WAAF	389	43.83	[MTFMT]
FLJ84523AAAF	410	47.49	[EPSTI1]
FLJ84527AAAF	57	6.19	[LINC01558]
FLJ84541AAAF	388	43.34	[P2RX4]
FLJ84543AAAF	273	30.86	[DNAJC27]
FLJ84547AAAF	205	22.94	[C20orf196]
FLJ84548AAAF	315	34.34	[GIPC2]
FLJ84554AAAN	682	78.24	[ZNF45]
FLJ84555AAAF	279	31.34	[MBLAC2]
FLJ84557AAAF	405	45.22	[ABHD1]
FLJ84558AAAF	887	100.39	[INTS6]
FLJ84560AAAF	258	28.22	[RHOU]
FLJ84563AAAF	464	51.54	[GFRA2]
FLJ84567SAAF	504	56.16	[SLC36A4]
FLJ84570AAAF	377	41.74	[HDAC8]
FLJ84571AAAN	953	106.36	[ZC3H18]
FLJ84572AAAF	221	25.41	[C11orf74]
FLJ84575AAAN	668	75.02	[MTA2]
FLJ84577AAAF	235	26.13	[MSRA]
FLJ84580AAAF	188	20.76	[JOSD2]
FLJ84583AAAF	781	89.36	[GCFC2]
FLJ84585WAAF	454	47.58	[BCL3]
FLJ84588AAAF	388	40.42	[SSBP3]
FLJ84589AAAF	256	27.93	[RAB26]
FLJ84590AAAF	243	27.22	[TPK1]
FLJ84604AAAF	253	28.3	[CD151]
FLJ84605AAAF	231	24.74	[LYPLA2]
FLJ84607AAAF	293	34.25	[NIFK]
FLJ84608AAAF	237	25.3	[CBR4]
FLJ84618AAAF	349	37.78	[KLHDC9]
FLJ84621AAAF	233	25.25	[SOX15]
FLJ84623AAAF	350	40.59	[TMEM185B]
FLJ84624AAAF	900	101.12	[MARS]
FLJ84625AAAF	301	33.2	[NOSIP]
FLJ84627AAAF	203	23.12	[RAB24]
FLJ84628AAAF	217	24.78	[TMED3]
FLJ84629AAAF	181	20.6	[ARF3]
FLJ84631AAAF	340	37.33	[GNB2]
FLJ84632AAAF	399	44.26	[TERF2IP]
FLJ84633AAAF	737	82.57	[DDX50]
FLJ84634AAAF	613	67.18	[GGA2]
FLJ84635AAAF	208	23.28	[FADD]
FLJ84637AAAF	244	25.79	[QDPR]
FLJ84641AAAF	399	43.57	[SIRT3]
FLJ84644AAAF	212	23.82	[DTYMK]
FLJ84646AAAF	148	15.96	[IFT20]
FLJ84647AAAN	464	49.56	[MYCN]

FLJ84648AAAF	163	18.24	[PIN1]
FLJ84649AAAF	160	18.27	[MAX]
FLJ84654AAAF	396	43.59	[RTN4IP1]
FLJ84662AAAF	337	39.16	[CAB39L]
FLJ84665AAAF	460	49.63	[SLC39A8]
FLJ84666AAAF	275	30.34	[PROSC]
FLJ84667AAAF	140	15.67	[CCDC126]
FLJ84670AAAF	262	29.28	[MRRF]
FLJ84671AAAF	429	45.92	[RUNX3]
FLJ84672AAAF	243	26.22	[CTHRC1]
FLJ84675AAAF	299	33.3	[PHB2]
FLJ84676AAAF	626	71.63	[NXF2]
FLJ84679AAAF	356	37.77	[NFKBIB]
FLJ84683AAAF	259	30.98	[KRCC1]
FLJ84686AAAF	400	44.9	[SIRT7]
FLJ84689AAAF	406	46.22	[PIP4K2A]
FLJ84695AAAF	277	31.19	[ZWINT]
FLJ84698AAAF	190	21.18	[COMMD1]
FLJ84701AAAF	493	54.77	[CETP]
FLJ84703AAAF	505	53.9	[SNTA1]
FLJ84704AAAF	304	34.77	[KATNBL1]
FLJ84707AAAN	518	57.71	[TBX5]
FLJ84708AAAF	364	39.73	[CD33]
FLJ84713AAAF	149	16.51	[CST6]
FLJ84718AAAF	250	28.73	[KCNIP4]
FLJ84719AAAF	553	60.55	[GLYR1]
FLJ84730AAAF	306	35.58	[RPF2]
FLJ84731AAAF	348	39.63	[PSTK]
FLJ84733AAAF	1042	114.76	[ATP2A2]
FLJ84735AAAF	337	36.56	[LIX1L]
FLJ84751AAAF	423	46.66	[MPI]
FLJ84754AAAF	367	43.29	[FBXO25]
FLJ84756AAAF	395	42.48	[GABPB1]
FLJ84765AAAF	351	39.05	[WNT4]
FLJ84766AAAF	523	59.72	[ADCK1]
FLJ84770AAAF	1019	108	[INTS5]
FLJ84773AAAF	518	56.89	[RIPK3]
FLJ84775AAAF	929	103.71	[SCYL2]
FLJ84777AAAF	351	39.6	[TMOD2]
FLJ84780AAAF	452	50.97	[ETNK1]
FLJ84783AAAF	432	46.51	[SUCLG2]
FLJ84784AAAF	624	65.7	[UBQLN2]
FLJ84787AAAF	167	19.13	[PTP4A2]
FLJ84788AAAF	88	10.6	[LYRM5]
FLJ84790AAAF	360	39.47	[TMEM198]
FLJ84791AAAF	602	66.02	[CKAP4]
FLJ84792AAAF	381	44.25	[GNA12]
FLJ84793WAAF	232	25.91	[TMEM129]

FLJ84794WAAF	400	46.53	[TBC1D13]
FLJ84795AAAF	361	39.77	[ZBTB37]
FLJ84799AAAF	275	29.57	[EXOSC3]
FLJ84801AAAF	424	44.29	[ACAA1]
FLJ84803AAAF	307	34.57	[SLC25A17]
FLJ84807AAAF	425	46.56	[SCRN2]
FLJ84808AAAF	448	49.54	[AZIN1]
FLJ84817AAAN	509	56.14	[SOX9]
FLJ84818AAAF	392	42.91	[SEPHS1]
FLJ84819AAAF	309	34.1	[SDR16C5]
FLJ84821AAAF	290	33.54	[RSRP1]
FLJ84823AAAF	428	48.99	[DDX39B]
FLJ84825AAAF	168	17.49	[ATP5D]
FLJ84826AAAF	1265	147.94	[PLCG2]
FLJ84828AAAF	471	54.23	[NONO]
FLJ84832AAAF	527	59.46	[PPIL2]
FLJ84839AAAF	1011	113.74	[MAN2B1]
FLJ84840AAAF	235	27.28	[TMED9]
FLJ84846AAAF	213	23.79	[STARD5]
FLJ84847AAAF	380	40.7	[FOS]
FLJ84849AAAF	261	28.67	[KLK2]
FLJ84852AAAF	328	35.11	[HEYL]
FLJ84854AAAF	280	31.69	[HUS1]
FLJ84855AAAF	333	39	[FAXDC2]
FLJ84857AAAF	141	16.09	[TMEM128]
FLJ84866AAAF	200	21.81	[ITGB1BP1]
FLJ84867AAAF	324	36.95	[ALG5]
FLJ84872AAAF	327	35.85	[DHRS13]
FLJ84874AAAF	405	44.51	[CTH]
FLJ84877AAAF	160	17.89	[PMP22]
FLJ84879AAAF	239	25.44	[MS4A4A]
FLJ84881AAAF	78	8.96	[DLEU1]
FLJ84884AAAF	323	34.83	[AQP4]
FLJ84889AAAF	444	50.46	[IFI44]
FLJ84890AAAF	1052	121.91	[SMARCA5]
FLJ84891AAAF	340	36.33	[HMGCLL1]
FLJ84892AAAF	393	44.93	[HPD]
FLJ84893AAAF	776	87.1	[ADAM20]
FLJ84904AAAF	790	90.02	[KIF9]
FLJ84905AAAF	603	69.93	[MTM1]
FLJ84907AAAF	481	50.48	[CELF6]
FLJ84910AAAF	271	27.91	[OLIG1]
FLJ84911AAAF	141	15.68	[OPALIN]
FLJ84912AAAF	349	38.86	[KHDRBS2]
FLJ84921AAAF	571	64.73	[GALNT2]
FLJ84927AAAF	412	46.37	[SKA3]
FLJ84928AAAF	402	46.94	[ZNF322]
FLJ84931AAAF	311	35.42	[VASH2]

FLJ84932AAAF	323	35.56	[ERCC1]
FLJ84934AAAF	902	95.45	[NFATC4]
FLJ84939AAAF	474	53.35	[RBM45]
FLJ84946WAAF	405	44.78	[SLC22A23]
FLJ84950WAAF	260	28.86	[HAGH]
FLJ84951AAAF	737	83.79	[GYS1]
FLJ84952WAAF	433	49.41	[LGMN]
FLJ84953AAAF	219	23.93	[BNIP3L]
FLJ84954AAAF	432	47.72	[AHCY]
FLJ84955AAAF	627	70.76	[CCDC22]
FLJ84957AAAF	340	38.5	[RFC5]
FLJ84961AAAF	641	70	[WBP11]
FLJ84964AAAF	338	37.92	[SCAMP1]
FLJ84968AAAF	397	46.02	[B3GNT2]
FLJ84972AAAF	209	21.94	[TIMM23]
FLJ84974AAAF	335	37.12	[B3GAT3]
FLJ84976AAAF	313	34.06	[SGTA]
FLJ84977AAAF	393	43.93	[B4GALT3]
FLJ84980AAAF	162	18.76	[SNX3]
FLJ84981AAAF	375	41.79	[ACTG1]
FLJ84985WAAF	297	33.73	[MEMO1]
FLJ84986AAAF	326	34.77	[NUP35]
FLJ84990AAAF	433	48.03	[GSK3B]
FLJ84991AAAF	606	64.95	[MAGED2]
FLJ85004AAAF	115	12.67	[CCK]
FLJ85005AAAF	288	31.79	[PBLD]
FLJ85007AAAF	213	23.92	[IFT43]
FLJ85009AAAF	379	40.68	[RTCA]
FLJ85015AAAF	375	42.82	[UBE2Q2]
FLJ85018AAAF	464	51.57	[SRPX]
FLJ85030AAAF	183	19.68	[ANKRD39]
FLJ85035AAAF	304	33.53	[DPPA4]
FLJ85036AAAF	982	109.42	[CBLB]
FLJ85039AAAF	376	43.18	[FMOD]
FLJ85044AAAF	318	35.41	[LCORL]
FLJ85052AAAF	458	52.49	[ZNF19]
FLJ85054AAAF	984	112.85	[MORC1]
FLJ85055WAAF	483	54.69	[DDX25]
FLJ85056AAAF	328	37.43	[SUPT3H]
FLJ85057AAAN	706	75.85	[TCF12]
FLJ85062AAAN	684	77	[SKIL]
FLJ85066AAAN	724	80.01	[GAB1]
FLJ85067AAAF	483	54.42	[DDX6]
FLJ85073AAAF	380	42.5	[GPR132]
FLJ85077AAAF	391	42.33	[RBMX]
FLJ85078AAAF	218	25.06	[EIF3K]
FLJ85082AAAF	391	44.34	[ELMOD3]
FLJ85087WAAF	446	50.92	[ZNF3]

FLJ85092AAAF	179	20.3	[NUDT1]
FLJ85093AAAF	466	53.65	[VIM]
FLJ85096AAAF	364	42.07	[AGPAT5]
FLJ85101AAAF	195	21.7	[RAB31]
FLJ85104AAAF	207	23.36	[NAT9]
FLJ85105AAAF	194	21.44	[DESI2]
FLJ85113AAAF	408	44.97	[ZNF764]
FLJ85114AAAF	346	39.25	[POLR1C]
FLJ85122AAAF	337	38.13	[C11orf49]
FLJ85126AAAF	558	63.43	[FANCC]
FLJ85128AAAF	542	60.08	[HNRNPLL]
FLJ85133AAAF	164	17.84	[RNASEH2C]
FLJ85134AAAF	1087	121	[SUPT5H]
FLJ85137AAAF	272	31.28	[FAM124B]
FLJ85141AAAF	349	39.04	[ISL1]
FLJ85142AAAF	429	48.63	[SRFBP1]
FLJ85146AAAF	221	24.84	[RAB28]
FLJ85149AAAF	870	98.06	[DNM2]
FLJ85150AAAF	446	48.69	[WIPI1]
FLJ85151AAAF	329	37.73	[CD86]
FLJ85161AAAF	1220	138.19	[BRPF1]
FLJ85163AAAF	475	55.2	[GABRG2]
FLJ85165AAAF	951	104.05	[CC2D1A]
FLJ85168AAAF	386	43.41	[HIBCH]
FLJ85171AAAF	361	39.94	[WDR45]
FLJ85173AAAF	392	44.65	[SPOPL]
FLJ85176AAAF	155	16.13	[ID1]
FLJ85179AAAF	229	26.17	[RABL2B]
FLJ85185AAAF	738	82.45	[PECAM1]
FLJ85187AAAF	282	29.13	[IGFBP7]
FLJ85189WAAF	298	32.85	[SLC25A5]
FLJ85200AAAF	141	15.59	[SPANXN3]
FLJ85201AAAF	85	9.78	[CDC26]
FLJ85202AAAF	193	21.71	[ANXA2R]
FLJ85217WAAF	160	17.82	[GADD45B]
FLJ85233WAAF	172	19.47	[C10orf126]
FLJ85239AAAF	149	16.62	[FAM72A]
FLJ85240AAAF	193	22.09	[C2orf80]
FLJ85254WAAF	128	15.29	[LINC01553]
FLJ85284WAAF	94	10.53	[SFTA3]
FLJ85292WAAF	140	15.34	[THEG5]
FLJ85295WAAF	173	19.14	[C12orf74]
FLJ85302WAAF	124	14.09	[CCDC153]
FLJ85318WAAF	97	11.27	[TSTD3]
FLJ85320WAAF	139	15.01	[LOC388820]
FLJ85332WAAF	107	11.44	[ARMS2]
FLJ85333WAAF	434	48.86	[GOLGA8G]
FLJ85404SAAN	572	61.88	[DPYSL4]

FLJ85450AAAF	538	60.25	[KPNA1]
FLJ85452AAAF	653	71.99	[TRIM32]
FLJ85457AAAF	430	48.64	[SMAD9]
FLJ85459AAAF	763	84.28	[FOXM1]
FLJ85465AAAF	595	69.67	[NF2]
FLJ85466AAAF	487	56.75	[RGS7]
FLJ85467AAAF	756	87.06	[DIEXF]
FLJ85472AAAF	790	87.92	[CDH18]
FLJ85473AAAF	294	33.36	[MPPED2]
FLJ85481AAAN	1017	111.84	[HIRA]
FLJ85486AAAF	614	66.81	[NR1D1]
FLJ85487AAAF	442	50.59	[PLVAP]
FLJ85490AAAF	569	65.4	[IL1R1]
FLJ85498AAAN	217	24.85	[GH1]
FLJ85501SAAF	521	58.69	[PPP3CA]
FLJ85505AAAF	785	80.69	[SP1]
FLJ85506AAAF	205	22.66	[RHOQ]
FLJ85507SAAF	766	88.28	[DPP4]
FLJ85509AAAF	953	107.14	[COPB1]
FLJ85510AAAF	491	55.54	[GPS1]
FLJ85512AAAF	470	53.54	[DES]
FLJ85518AAAF	400	45.57	[MAPKAPK2]
FLJ85543SAAN	1021	110.7	[EPHB6]
FLJ85552AAAF	558	61.4	[GLUD1]
FLJ85554AAAF	679	76.96	[KIF2A]
FLJ85555AAAF	868	96.02	[PDCD6IP]
FLJ85556AAAF	933	104.1	[CTNND1]
FLJ85558AAAF	709	78.37	[CAPRIN1]
FLJ85561AAAN	548	59.91	[RELA]
FLJ85563AAAF	375	39.85	[ADH1B]
FLJ85564AAAF	375	39.87	[ADH1C]
FLJ85565AAAF	777	85.66	[NR3C1]
FLJ85566AAAF	533	56.92	[RXRB]
FLJ85567AAAF	414	45.57	[NR2F2]
FLJ85568AAAF	598	66.59	[NR4A2]
FLJ85569SAAF	433	49.65	[NR1I2]
FLJ85570AAAF	556	60.34	[TCP1]
FLJ85571AAAF	544	60.4	[CCT3]
FLJ85572AAAF	530	57.77	[CCT6B]
FLJ85573AAAF	548	59.62	[CCT8]
FLJ85574AAAF	582	66.9	[LARP7]
FLJ85575SAAF	865	97.2	[PROM1]
FLJ85578AAAF	195	20.98	[CYBA]
FLJ85580AAAF	296	33.23	[MYD88]
FLJ85581AAAF	100	10.87	[EIF4EBP3]
FLJ85582AAAF	217	25.1	[EIF4E]
FLJ85583SAAF	1403	154.8	[EIF4G1]
FLJ85584AAAF	268	28.32	[CAPNS1]

FLJ85585SAAF	454	50.57	[MYC]
FLJ85587AAAF	407	43.96	[RBMS2]
FLJ85588AAAF	1327	141.98	[TNKS]
FLJ85590SAAF	1585	176.65	[EIF4G3]
FLJ85591AAAN	225	25.1	[ARHGDIG]
FLJ85592AAAF	191	21.26	[CDC42]
FLJ85593SAAF	201	23.31	[RANBP1]
FLJ85594AAAF	317	34.31	[SOX2]
FLJ85595WAAF	201	22.84	[PSMB2]
FLJ85596SAAF	566	61.58	[FBLN1]
FLJ85597SAAF	324	35.96	[PPM1A]
FLJ85600SAAF	748	86.2	[SMURF2]
FLJ85602AAAF	1087	114.54	[UBAP2L]
FLJ85603AAAF	910	102	[SND1]
FLJ85604AAAF	399	44.22	[RRAGC]
FLJ85605AAAF	615	67.26	[VGF]
FLJ85607AAAF	427	49.13	[DDX39A]
FLJ85608AAAF	783	87.34	[DDX21]
FLJ85609AAAF	202	22	[U2AF1L4]
FLJ85610AAAF	483	51.62	[CELF1]
FLJ85614WAAF	200	22.73	[PABPC1L2B]
FLJ85616SAAN	269	30.25	[CPSF4]
FLJ85619SAAF	593	64.07	[RBM47]
FLJ85620AAAF	647	71.72	[CEP72]
FLJ85621SAAF	464	53.28	[CDK8]
FLJ85622AAAF	233	27.25	[MED7]
FLJ85624AAAF	178	19.52	[MED28]
FLJ85626AAAF	747	78.17	[MED25]
FLJ85627SAAF	632	71.17	[FMR1]
FLJ85628AAAF	712	78.8	[LRCH3]
FLJ85629AAAF	743	76.47	[POU2F1]
FLJ85631AAAF	72	8.67	[RABGAP1L]
FLJ85632AAAN	1074	124.18	[PLD1]
FLJ85633AAAF	490	53.84	[CYP2W1]
FLJ85634AAAF	520	59.85	[CYP4F2]
FLJ85635AAAF	137	14.87	[CREM]
FLJ85638AAAF	870	96.46	[EPAS1]
FLJ85641AAAF	729	81.99	[IFI16]
FLJ85642AAAF	382	40.99	[MBNL1]
FLJ85644AAAF	510	57.84	[ETV5]
FLJ85648AAAF	100	10.66	[HMGN1]
FLJ85649AAAF	107	11.68	[HMGA1]
FLJ85652AAAF	486	52.44	[MECP2]
FLJ85653AAAF	499	53.85	[MEF2A]
FLJ85658AAAF	189	21.23	[NRAS]
FLJ85663AAAF	608	67.45	[TOMM70A]
FLJ85664AAAF	211	23.66	[SCG5]
FLJ85666AAAF	730	77.52	[HNRNPM]

FLJ85667AAAN	645	72.93	[PDIA4]
FLJ85668SAAF	277	29.97	[PSMB7]
FLJ85670SAAF	218	24.88	[ADGRF1]
FLJ85671SAAF	486	51.97	[CELF4]
FLJ85672AAAN	324	35.92	[YBX1]
FLJ85673WAAF	394	43.79	[PA2G4]
FLJ85676WAAN	513	56.67	[RTKN]
FLJ85679AAAN	1105	122.91	[SMARCC1]
FLJ85682SAAN	435	48.68	[TEAD3]
FLJ85684AAAN	800	90.25	[SART1]
FLJ85685AAAN	743	79.84	[TLE2]
FLJ85686AAAN	411	46.75	[SNAPC3]
FLJ85691SAAN	580	66.05	[MBD4]
FLJ85693SAAN	482	58.04	[ZRSR2]
FLJ85695SAAN	515	58.23	[SMARCD1]
FLJ85696SAAN	170	19.31	[UBE2V1]
FLJ85699WAAN	330	37.4	[ZRANB2]
FLJ85702AAAN	251	28.29	[C4BPB]
FLJ85703AAAN	406	45.7	[SERPINA5]
FLJ85708WAAN	366	38.65	[PCBP2]
FLJ85710SAAN	938	102.98	[DDX42]
FLJ85714AAAN	258	28.86	[SNF8]
FLJ85716AAAN	393	43.7	[IRF9]
FLJ85721AAAN	727	82.79	[CTCF]
FLJ85722AAAN	534	60.61	[POLR3C]
FLJ85725AAAN	146	16.97	[LDOC1]
FLJ85730AAAN	399	44.2	[LEF1]
FLJ85733SAAN	772	85.92	[L3MBTL1]
FLJ85735AAAN	433	48.63	[PSMC2]
FLJ85736AAAF	195	22.25	[PXMP2]
FLJ85738WAAN	168	19.67	[ARPC4]
FLJ85739AAAN	654	72.33	[HSPA5]
FLJ85742SAAN	933	102.96	[TPO]
FLJ85757AAAN	328	38.14	[SUDS3]
FLJ85759AAAN	81	9.17	[CTNNBIP1]
FLJ85771AAAN	298	33.06	[SLC25A4]
FLJ85784AAAN	1025	113.24	[OBSL1]
FLJ85785AAAN	1102	126.45	[PIK3CG]
FLJ85786AAAN	728	81.62	[PIK3R2]
FLJ85788SAAN	149	16.89	[UBE2D3]
FLJ85789AAAN	708	79.88	[MYH2]
FLJ85792AAAN	262	31.24	[TNNT1]
FLJ85793SAAN	173	19.53	[MYL5]
FLJ85794AAAN	284	32.99	[TPM2]
FLJ85796AAAN	490	54.86	[MDM4]
FLJ85800AAAN	426	47.35	[NMUR1]
FLJ85805AAAN	88	10.53	[COX6B2]
FLJ85806AAAN	1264	147.16	[RGS22]

FLJ85808AAAN	402	45.29	[SEMG1]
FLJ85809AAAN	314	34.89	[MAGEA6]
FLJ85810WAAN	594	66.34	[CCDC36]
FLJ85812WAAN	58	6.71	[ROPN1]
FLJ85813SAAN	389	44.53	[KIAA0100]
FLJ85814SAAN	183	20.66	[LYPD6B]
FLJ85816SAAN	43	4.71	[BAGE]
FLJ85817AAAN	116	12.76	[GAGE2E]
FLJ85818WAAN	136	15.41	[CT62]
FLJ85819AAAN	559	61.2	[BORA]
FLJ85836AAAF	440	49.58	[LCAT]
FLJ85854WAAN	445	50.73	[NPY5R]
FLJ85858AAAN	127	14.21	[FABP1]
FLJ85870WAAN	105	11.53	[CORT]
FLJ85918SAAN	467	54.29	[GABRG3]
FLJ85921SAAN	458	51.84	[HTR2C]
FLJ85930WAAN	94	10.92	[ATP5J2]
FLJ85935AAAN	328	35.14	[IGFBP2]
FLJ85941AAAN	855	96.26	[SPICE1]
FLJ85942AAAN	934	104.74	[MSH2]
FLJ85943SAAN	603	69.65	[HAUS3]
FLJ85949AAAN	833	92.77	[KIFC3]
FLJ85961WAAF	439	45.16	[COL26A1]
FLJ85971SAAN	852	92.9	[KDM1A]
FLJ85990AAAN	234	25.9	[PSMA2]
FLJ85991AAAN	377	40.74	[PSMD4]
FLJ85995AAAF	252	28.23	[SPINT2]
FLJ85996AAAN	474	52.94	[GC]
FLJ85999AAAN	310	34.58	[PSMD14]
FLJ86006AAAN	496	56.81	[NMT1]
FLJ86009AAAF	613	66.05	[SEC24A]
FLJ86019AAAN	770	86.34	[COG7]
FLJ86023AAAN	736	78.95	[DVL2]
FLJ86030AAAN	1202	135.35	[PAN2]
FLJ86047SAAN	589	66.12	[FARSB]
FLJ89003AAAN	1748	195.46	[TJP1]
FLJ89004AAAN	1663	187.15	[C3]
FLJ89019AAAN	256	27.81	[PRTN3]
FLJ90018AAAN	636	68.67	[LTBP4]
FLJ90022AAAN	423	45.83	[FAM43A]
FLJ90023AAAF	370	40.11	[GJD4]
FLJ90035AAAF	357	39.32	[SNRNP40]
FLJ90037AAAF	312	35.57	[OSR2]
FLJ90038AAAF	517	59.1	[CERCAM]
FLJ90039AAAF	377	42.55	[ERGIC2]
FLJ90043AAAF	739	82.93	[GARS]
FLJ90050AAAF	167	18.67	[PRR3]
FLJ90057AAAF	377	41.91	[TPST2]

FLJ90058AAAF	577	67.75	[ZNF573]
FLJ90061AAAF	463	51.1	[RUVBL2]
FLJ90065AAAF	441	50.17	[ZBTB8A]
FLJ90066AAAF	149	14.95	[CEND1]
FLJ90067AAAF	310	33.96	[ABHD17A]
FLJ90068AAAF	215	21.69	[SFT2D3]
FLJ90069AAAF	388	43.76	[ERGIC3]
FLJ90072AAAF	440	48.96	[TTC5]
FLJ90075AAAN	789	86.09	[BOC]
FLJ90079AAAF	389	43.44	[CBX8]
FLJ90089AAAF	239	27.34	[ANKRD36B]
FLJ90093AAAF	231	25.84	[PRRG3]
FLJ90094AAAF	590	66.06	[CHRM3]
FLJ90104AAAF	193	21.39	[PERP]
FLJ90110AAAF	266	29.61	[OSR1]
FLJ90112AAAF	581	64.14	[MATN4]
FLJ90119AAAF	240	26.37	[TMEM86A]
FLJ90121AAAF	528	59.87	[NSMF]
FLJ90123AAAF	196	21.77	[SERTAD3]
FLJ90132AAAN	405	45.35	[BRD7]
FLJ90136AAAF	369	42.15	[TRAM1L1]
FLJ90138AAAF	447	49.82	[TBL2]
FLJ90143AAAF	461	52.09	[SIL1]
FLJ90144AAAF	692	77.3	[MTO1]
FLJ90151AAAF	374	41.43	[TMEFF2]
FLJ90156AAAF	863	96.33	[PTGFRN]
FLJ90157AAAF	707	78.51	[ZCCHC8]
FLJ90162AAAF	229	25.75	[FAM213A]
FLJ90166AAAF	501	55.58	[APCDD1L]
FLJ90167AAAF	245	27.46	[TMEM116]
FLJ90169AAAF	415	45.03	[SLC52A3]
FLJ90171AAAF	236	24.7	[SERTAD1]
FLJ90174AAAF	423	49.4	[PIGM]
FLJ90175AAAF	508	57.12	[TBC1D10A]
FLJ90177AAAF	622	71.24	[GALNT6]
FLJ90178AAAF	427	47.47	[PUS1]
FLJ90180AAAF	398	41.78	[S1PR5]
FLJ90182AAAF	563	65.22	[CTNNBL1]
FLJ90186AAAF	161	17.51	[TMEM159]
FLJ90187AAAF	428	46	[ISLR]
FLJ90188AAAF	285	31.67	[SLAMF8]
FLJ90192AAAF	721	80.12	[PRKD2]
FLJ90193AAAF	471	47.79	[SLC16A11]
FLJ90195AAAF	446	49.35	[GRWD1]
FLJ90196AAAF	198	22.53	[TMEM9B]
FLJ90200AAAF	321	34.63	[ATOH8]
FLJ90201AAAF	593	67.57	[PGBD3]
FLJ90203AAAF	450	49.74	[MFSD5]

FLJ90205AAAF	143	15.43	[HM13]
FLJ90206AAAF	401	44.84	[CANT1]
FLJ90207AAAF	263	28.1	[PIK3IP1]
FLJ90208AAAF	259	29.06	[RAB34]
FLJ90210AAAF	456	49.85	[ENTPD6]
FLJ90213AAAN	442	49.69	[SPRYD3]
FLJ90217AAAF	627	70.73	[SRP68]
FLJ90219AAAF	530	57.52	[GHDC]
FLJ90225AAAF	476	52.26	[SEC61A1]
FLJ90230AAAN	320	36.29	[HHIP]
FLJ90238AAAN	1106	124.69	[ERCC6L]
FLJ90251AAAF	561	62.89	[TMEM209]
FLJ90254AAAF	433	47.26	[TPRN]
FLJ90258AAAF	376	42.94	[CHST14]
FLJ90275AAAF	153	17.37	[ORMDL1]
FLJ90279AAAF	183	21.16	[JAGN1]
FLJ90280AAAF	419	45.82	[NFATC2IP]
FLJ90281AAAF	147	16.87	[POLE3]
FLJ90288AAAF	310	35.02	[JAM3]
FLJ90289AAAF	331	35.77	[SPON2]
FLJ90290AAAF	394	43.96	[STRADA]
FLJ90291AAAF	478	53.67	[POC1B]
FLJ90292AAAF	446	49.56	[CLEC18A]
FLJ90293AAAF	389	44.6	[ZNF670]
FLJ90294AAAN	852	94.65	[LONP2]
FLJ90296AAAF	439	50.36	[MRPS30]
FLJ90300AAAF	144	16.7	[CNIH1]
FLJ90302AAAF	585	64.33	[PRR14]
FLJ90304AAAF	453	49.18	[ARMCX1]
FLJ90309AAAF	420	46.02	[TPBG]
FLJ90311AAAF	650	72.17	[MTMR14]
FLJ90312AAAF	325	34.54	[NPDC1]
FLJ90313AAAF	439	49.94	[TSEN2]
FLJ90315AAAN	762	83	[THBS3]
FLJ90318AAAF	454	52.54	[DAPK3]
FLJ90321AAAF	237	25.88	[TGIF2]
FLJ90322AAAF	807	91.1	[SPON1]
FLJ90323AAAF	840	92.89	[MASTL]
FLJ90326AAAF	672	74.94	[CAPN10]
FLJ90328AAAF	748	83.46	[HJURP]
FLJ90329AAAF	355	39.15	[SIRT6]
FLJ90341AAAF	362	42.29	[EPHX4]
FLJ90342AAAF	494	54.53	[SLC37A3]
FLJ90354AAAF	760	88	[SEC63]
FLJ90360AAAF	131	14.25	[LEPROT]
FLJ90363AAAF	338	36.84	[TMEM8B]
FLJ90364AAAF	300	33.78	[NUDT9]
FLJ90365AAAF	461	52.79	[ZNF627]

FLJ90374AAAF	636	72.08	[GLB1L2]
FLJ90378AAAF	323	34.24	[ZNF784]
FLJ90385AAAF	385	42.97	[TRIM11]
FLJ90386AAAF	519	55.4	[FAM98A]
FLJ90389AAAF	555	63.43	[TMEM87A]
FLJ90391AAAF	402	46.31	[ZNF253]
FLJ90393AAAF	725	82.57	[POMT1]
FLJ90396AAAF	576	66.8	[ZNF791]
FLJ90398AAAF	544	62.18	[MOXD1]
FLJ90408AAAF	299	35.29	[ELOVL5]
FLJ90409AAAF	338	36.83	[UBIAD1]
FLJ90411AAAF	632	71.45	[ICK]
FLJ90412AAAN	308	33.43	[PIDD1]
FLJ90415AAAF	500	56.91	[ZNF689]
FLJ90416AAAF	426	48.06	[PANX1]
FLJ90428AAAF	649	73	[FLRT3]
FLJ90429AAAF	450	49.33	[RGMA]
FLJ90436AAAF	421	47.42	[WSB1]
FLJ90438AAAF	664	71.47	[RBBP8NL]
FLJ90446AAAF	507	54.54	[SLC2A6]
FLJ90450AAAF	431	48.31	[SYT11]
FLJ90453AAAF	237	27.64	[KCTD6]
FLJ90454AAAF	669	76.07	[CLPTM1]
FLJ90456AAAF	525	59.39	[NETO2]
FLJ90458AAAF	444	52.26	[FADS2]
FLJ90461AAAF	532	62.52	[ZFP82]
FLJ90462AAAF	324	34.25	[SLC39A1]
FLJ90473AAAN	265	30.8	[TBC1D1]
FLJ90475AAAF	466	51.71	[CS]
FLJ90478AAAF	503	57.01	[LEMD2]
FLJ90479AAAN	484	53.45	[PLXNA2]
FLJ90481AAAF	224	25.17	[TMED7]
FLJ90483AAAF	566	62.76	[DONSON]
FLJ90486AAAF	229	25.73	[SCAMP4]
FLJ90490AAAF	629	69.23	[DYRK1B]
FLJ90491AAAF	269	30.35	[CD200]
FLJ90495AAAF	267	29.74	[MTG1]
FLJ90502AAAF	543	57.79	[FAM134A]
FLJ90503AAAF	541	62.22	[WLS]
FLJ90504AAAF	400	43.18	[RNF149]
FLJ90508AAAF	126	14.28	[C1orf213]
FLJ90514AAAF	502	55.08	[UBAP1]
FLJ90518AAAF	254	29.51	[PSME3]
FLJ90520AAAF	365	41.55	[TUFT1]
FLJ90521AAAF	314	35.39	[SLC25A44]
FLJ90522AAAF	543	61.49	[NEFL]
FLJ90526AAAN	475	54.81	[ZMPSTE24]
FLJ90528AAAF	174	19.89	[RBM8A]

FLJ90532AAAF	275	29.74	[NECAP1]
FLJ90533AAAF	619	67.17	[SLC7A3]
FLJ90536AAAF	259	28.87	[UBE2J2]
FLJ90541AAAF	317	36.44	[METTL9]
FLJ90542AAAF	398	45.04	[ABHD12]
FLJ90543AAAF	406	46.97	[ERP44]
FLJ90546AAAF	171	19.01	[TM2D2]
FLJ90547AAAF	196	22.22	[LOH12CR1]
FLJ90552AAAF	381	40.48	[KREMEN2]
FLJ90559AAAF	214	23.48	[HES6]
FLJ90565AAAF	631	69.4	[SLC34A2]
FLJ90569AAAF	163	18.82	[ARL2BP]
FLJ90575AAAF	555	62.71	[CCDC96]
FLJ90580AAAF	220	23.96	[WFDC1]
FLJ90581AAAF	417	47.75	[ZNF114]
FLJ90583AAAF	303	32.41	[RELL2]
FLJ90584AAAN	269	29.17	[NOTCH2NL]
FLJ90588AAAF	238	26.96	[TMBIM4]
FLJ90591AAAF	245	27.53	[TSPAN6]
FLJ90603AAAF	435	48.51	[RECQL5]
FLJ90605AAAF	212	23.62	[PLAC1]
FLJ90610AAAF	154	17.16	[GLIPR2]
FLJ90619AAAF	333	37.53	[CTSL]
FLJ90626AAAF	339	37.77	[STK26]
FLJ90627AAAF	364	40.18	[ECI2]
FLJ90628AAAF	358	41.42	[CLN5]
FLJ90629AAAF	243	27.18	[VNN3]
FLJ90631AAAF	210	22.35	[ABHD14B]
FLJ90634AAAF	452	50.58	[DPH7]
FLJ90637AAAF	246	25.16	[NEXN-AS1]
FLJ90639AAAF	420	47.58	[UXS1]
FLJ90648AAAF	696	79.42	[RHOBTB1]
FLJ90649AAAF	355	41.42	[GPR1]
FLJ90652AAAF	244	26.48	[INO80E]
FLJ90659AAAF	288	31.68	[FCN3]
FLJ90666AAAF	448	50.18	[FBLN5]
FLJ90668AAAF	326	36.71	[PSG4]
FLJ90672AAAF	399	44	[VSIG4]
FLJ90675AAAF	298	33.81	[GOLPH3]
FLJ90686AAAF	287	31.74	[DLX3]
FLJ90697AAAF	429	47.14	[SCCPDH]
FLJ90699AAAF	230	25.71	[CRACR2B]
FLJ90702AAAF	530	58.62	[MFSD2A]
FLJ90707AAAF	295	31.65	[PLSCR3]
FLJ90711AAAF	630	71.13	[EIF2AK1]
FLJ90716AAAF	255	30.12	[ELF5]
FLJ90721AAAN	172	18.2	[SYT8]
FLJ90722AAAF	649	72.7	[UBASH3B]

FLJ90726AAAF	271	29.45	[PPAPDC3]
FLJ90730AAAF	406	45.07	[CHPT1]
FLJ90732AAAF	540	61.07	[RIPK2]
FLJ90735AAAF	312	34.32	[HSD17B12]
FLJ90746AAAF	183	20.09	[EMC4]
FLJ90761AAAF	434	48.77	[C16orf89]
FLJ90763AAAF	398	42.97	[STOML1]
FLJ90764AAAF	560	63.32	[ZNF619]
FLJ90766AAAF	261	30.39	[GJB6]
FLJ90767AAAF	606	65.32	[SLC26A11]
FLJ90768AAAF	287	31.27	[SLC25A10]
FLJ90771AAAF	380	42.69	[APLNR]
FLJ90775AAAF	639	70.38	[GGA1]
FLJ90778AAAF	222	25.14	[C9orf9]
FLJ90780AAAF	610	66.66	[RETSAT]
FLJ90783AAAF	156	17.61	[SYS1]
FLJ90784AAAF	581	63.07	[PYROXD2]
FLJ90789AAAF	687	77.1	[GPR56]
FLJ90791AAAF	752	82.52	[MARK4]
FLJ90793AAAF	410	45.51	[CNPPD1]
FLJ90798AAAF	241	26.95	[ZCCHC24]
FLJ90799AAAF	486	52.64	[APBB3]
FLJ90800AAAF	233	25.86	[MIS18A]
FLJ90804AAAF	350	38.56	[INHBE]
FLJ90805AAAF	493	55.6	[TMEM145]
FLJ90806AAAF	197	22.44	[SPC24]
FLJ90808AAAF	407	45.03	[POC1A]
FLJ90810AAAF	432	47.66	[TXNDC5]
FLJ90811AAAF	303	33.23	[PUSL1]
FLJ90815AAAF	232	26.07	[CCNDBP1]
FLJ90818AAAF	247	26.64	[MPDU1]
FLJ90820AAAF	257	29.43	[NOL7]
FLJ90823AAAF	305	34.09	[CYB5R1]
FLJ90824AAAF	597	66.6	[KLHL21]
FLJ90825AAAF	1050	116.85	[TRIM24]
FLJ90826AAAF	444	49.59	[TUBB4A]
FLJ90827AAAF	210	23.08	[AKIP1]
FLJ90833AAAF	468	52.95	[SCG3]
FLJ90834AAAF	251	27.89	[C11orf72]
FLJ90835AAAF	467	51.71	[IGSF21]
FLJ90840AAAF	519	58.27	[BBS4]
FLJ91004AAAF	326	36.12	[DEDD2]
FLJ91005AAAF	220	23.28	[CLDN6]
FLJ91009AAAF	190	21.03	[PTGDS]
FLJ91018AAAF	393	42.24	[TP53I13]
FLJ91025AAAF	271	30.2	[AQP11]
FLJ91027AAAF	300	32.94	[HSD17B11]
FLJ91028AAAF	406	43.86	[GLMP]

FLJ91030AAAN	141	14.73	[ATP5G2]
FLJ91040AAAF	159	17.04	[SDHD]
FLJ91048AAAF	253	28.6	[CLDND1]
FLJ91053AAAF	230	24.55	[CLDN2]
FLJ91054AAAF	295	33.49	[SFRP2]
FLJ91055AAAF	464	52.49	[ALG1]
FLJ91060AAAF	474	50.61	[MFSD9]
FLJ91061AAAF	730	78.64	[SLC27A3]
FLJ91063AAAF	433	47.74	[RNF26]
FLJ91066AAAF	350	39.02	[ATP6AP2]
FLJ91067AAAF	523	59.62	[UGT3A2]
FLJ91068AAAF	770	83.48	[SEMA4F]
FLJ91070AAAF	860	96.79	[TTC13]
FLJ91073AAAF	189	21.07	[PLA2G12A]
FLJ91074AAAF	740	83.56	[NAALAD2]
FLJ91080AAAF	390	41.18	[ESAM]
FLJ91082AAAF	467	52.39	[TINAGL1]
FLJ91084AAAF	543	61.08	[HPSE]
FLJ91087AAAF	76	8.54	[HSBP1]
FLJ91089AAAF	349	38.95	[TMX4]
FLJ91095AAAF	258	27.56	[FGFBP3]
FLJ91099AAAF	494	54.67	[PCYOX1L]
FLJ91105AAAF	208	21.53	[TMEM123]
FLJ91113AAAF	555	61.64	[PIGS]
FLJ91115AAAF	358	40.51	[DNAJB11]
FLJ91116AAAF	196	21.42	[CRH]
FLJ91117AAAF	550	59.52	[ALPP]
FLJ91118AAAF	476	54.16	[CPVL]
FLJ91125AAAF	297	33.01	[TMEM178A]
FLJ91127AAAF	240	25.58	[SHISA5]
FLJ91129AAAF	322	35.21	[LYVE1]
FLJ91130AAAF	392	46.19	[POGLUT1]
FLJ91131AAAN	571	63.92	[ISM2]
FLJ91139AAAF	443	49.39	[EFEMP2]
FLJ91140AAAF	346	39.64	[CYSLTR2]
FLJ91143AAAF	432	47.44	[SLC35B2]
FLJ91146AAAF	374	40.51	[SUMF1]
FLJ91149AAAF	465	52.97	[SRPX2]
FLJ91151AAAF	346	35.97	[LYPD3]
FLJ91155AAAF	496	53.7	[SLC2A11]
FLJ91156AAAF	271	29.31	[RELL1]
FLJ91157AAAF	578	65.7	[PIGT]
FLJ91158AAAF	463	49.37	[PI16]
FLJ91160AAAF	406	45.14	[ANGPTL4]
FLJ91162AAAF	269	30.73	[PGAP3]
FLJ91164AAAF	353	37.82	[TSKU]
FLJ91165AAAF	301	33.86	[SUMF2]
FLJ91168AAAF	682	76.66	[SV2A]

FLJ91176AAAF	657	75.39	[GALNT7]
FLJ91179AAAF	286	32.06	[RNASEH1]
FLJ91186AAAF	153	16.92	[NDFIP1]
FLJ91191AAAF	576	64.13	[ACSF3]
FLJ91192AAAF	343	37.54	[ARMC10]
FLJ91193AAAF	283	29.12	[TMEM119]
FLJ91194AAAF	443	48.65	[CADM1]
FLJ91197AAAF	323	36.22	[TMEM59]
FLJ91199AAAF	435	50	[PIGU]
FLJ91206AAAF	111	13.08	[CXCL14]
FLJ91207AAAF	323	36.92	[MRPS35]
FLJ91210AAAN	410	42.05	[SHARPIN]
FLJ91225AAAF	365	39.74	[FCGRT]
FLJ91229AAAF	211	24.27	[FIBIN]
FLJ91231AAAF	248	28.31	[CNPY4]
FLJ91233AAAF	423	46.46	[BACE2]
FLJ91235AAAF	622	71.64	[COLGALT1]
FLJ91236AAAF	401	45.33	[GOLM1]
FLJ91237AAAF	743	81.06	[CCDC142]
FLJ91238AAAF	287	32.45	[OLFML3]
FLJ91240AAAF	345	37.42	[LDLRAD3]
FLJ91252AAAF	349	38.68	[DRAXIN]
FLJ91254AAAF	496	53.96	[ADPGK]
FLJ91257AAAF	971	109.52	[EMC1]
FLJ91258AAAF	157	16.79	[FAM174B]
FLJ91259AAAF	192	20.42	[FAM210B]
FLJ92001AAAF	105	11.56	[C10orf32]
FLJ92002AAAF	266	30	[RPL7A]
FLJ92003AAAF	172	20.78	[NDUFB10]
FLJ92005AAAF	231	25.95	[NQO2]
FLJ92006AAAF	44	5.05	[TMSB4X]
FLJ92007AAAF	153	17.56	[SAP18]
FLJ92008AAAF	128	14.79	[RPL22]
FLJ92009AAAF	68	7.52	[GNG7]
FLJ92010AAAF	112	12.32	[ARPP19]
FLJ92011AAAF	86	9.73	[SNRPF]
FLJ92012AAAF	142	16.71	[GMFB]
FLJ92013AAAF	100	11.31	[VAMP3]
FLJ92014AAAF	127	14.4	[SUB1]
FLJ92015AAAF	124	13.62	[LAMTOR3]
FLJ92016AAAF	111	13.53	[UQCRB]
FLJ92017AAAF	107	12.33	[SH3BGRL2]
FLJ92018AAAF	132	14.91	[PMP2]
FLJ92019AAAF	128	15.16	[MRPS14]
FLJ92020AAAF	137	15.75	[RPL28]
FLJ92022AAAF	154	17.86	[UBE2L3]
FLJ92023AAAF	130	15.04	[PEA15]
FLJ92025AAAF	140	15.86	[C14orf1]

FLJ92026AAAF	116	12.5	[DNAJC19]
FLJ92027AAAF	130	14.09	[HIST1H2AI]
FLJ92028AAAF	148	16.56	[RPL27A]
FLJ92029AAAF	239	25.36	[PSMB6]
FLJ92030AAAF	79	9.66	[CKS1B]
FLJ92031AAAF	216	24.39	[RAB11A]
FLJ92033AAAF	143	15.81	[RPS23]
FLJ92035AAAF	176	20.75	[RPL18A]
FLJ92036AAAF	125	14.49	[RPL31]
FLJ92037AAAF	130	15.07	[RPS24]
FLJ92038AAAF	147	15.89	[CARHSP1]
FLJ92039AAAF	128	14.18	[CD59]
FLJ92041AAAF	140	15.09	[PFN2]
FLJ92042AAAF	75	8.39	[GNG4]
FLJ92045AAAF	164	18.65	[CNRIP1]
FLJ92046AAAF	130	14.14	[HIST1H2AB]
FLJ92047AAAF	145	16.06	[RPS19]
FLJ92050AAAF	178	20.52	[ARPC3]
FLJ92051AAAF	89	10.37	[DYNLL1]
FLJ92052AAAF	75	8.78	[COX6C]
FLJ92053AAAF	136	15.77	[RPL27]
FLJ92054AAAF	181	20.42	[ARL1]
FLJ92055AAAF	194	22.13	[RPS7]
FLJ92056AAAF	160	18.56	[RPL21]
FLJ92057AAAF	213	23.28	[ATP5O]
FLJ92058AAAF	165	18.9	[RPS10]
FLJ92059AAAF	102	13.05	[PRM2]
FLJ92060AAAF	52	6.11	[PLN]
FLJ92061AAAF	128	14.17	[NHP2L1]
FLJ92063AAAF	152	17.07	[DEFB125]
FLJ92064AAAF	144	16.16	[SRP19]
FLJ92065AAAF	145	17.26	[RPL26]
FLJ92067AAAF	151	17.41	[SPA17]
FLJ92069AAAF	119	13.37	[RPS20]
FLJ92070AAAF	158	18.43	[RPS11]
FLJ92071AAAF	172	18.88	[ZG16B]
FLJ92072AAAF	130	14.11	[HIST1H2AC]
FLJ92073AAAF	62	7.3	[STATH]
FLJ92076AAAF	93	10.14	[HIGD1A]
FLJ92077AAAF	178	20.25	[RPL11]
FLJ92078AAAF	184	20.09	[ESM1]
FLJ92080AAAF	178	20.37	[NAA20]
FLJ92081AAAF	81	8.97	[AKAP7]
FLJ92083AAAF	127	14.48	[NUTF2]
FLJ92084AAAF	169	18.15	[SPRR3]
FLJ92086AAAF	147	16	[HBB]
FLJ92087AAAF	132	14.51	[RPS12]
FLJ92088AAAF	97	11.08	[RPL37]

FLJ92089AAAF	184	21.4	[RPL17]
FLJ92091AAAF	148	16.87	[CRCP]
FLJ92092AAAF	148	16.36	[PLA2G1B]
FLJ92093AAAF	127	14.48	[POLR2F]
FLJ92094AAAF	110	12.06	[PVALB]
FLJ92095AAAF	122	14.33	[POLR1D]
FLJ92097AAAF	126	14.87	[UQCC2]
FLJ92098AAAF	105	11.75	[CYCS]
FLJ92100AAAF	95	10.87	[SUMO2]
FLJ92101AAAF	144	15.56	[MED21]
FLJ92102AAAF	103	11.34	[TCTA]
FLJ92103AAAF	101	11.56	[SUMO1]
FLJ92104AAAF	90	9.54	[HMGN4]
FLJ92105AAAF	121	13.57	[RPA3]
FLJ92106AAAF	193	21.71	[AP3S1]
FLJ92107AAAF	153	16.71	[MAL]
FLJ92109AAAF	68	7.74	[SEC61G]
FLJ92111AAAF	101	11.53	[ENY2]
FLJ92112AAAF	169	19.58	[COX4I1]
FLJ92113AAAF	136	14.28	[ATP5G1]
FLJ92114AAAF	126	13.9	[HIST1H2BJ]
FLJ92115AAAF	175	20.27	[NDUFAF4]
FLJ92116AAAF	116	13.06	[DYNLT3]
FLJ92117AAAF	125	13.92	[CXCL9]
FLJ92120AAAF	208	24.21	[RPS8]
FLJ92121AAAF	99	10.58	[C19orf53]
FLJ92122AAAF	135	15.16	[FABP5]
FLJ92124AAAF	196	23.47	[RPL19]
FLJ92125AAAF	180	19.88	[RNF5]
FLJ92126AAAF	215	24.94	[CSNK2B]
FLJ92128AAAF	113	12.73	[EIF1]
FLJ92129AAAF	288	32.7	[RPL6]
FLJ92130AAAF	209	24.03	[HMGB2]
FLJ92131AAAF	198	22.28	[APOO]
FLJ92132AAAF	150	16.38	[DNAJC15]
FLJ92133AAAF	132	14.89	[FABP7]
FLJ92134AAAF	106	11.78	[GLRX]
FLJ92135AAAF	175	19.45	[MYL7]
FLJ92136AAAF	158	17.94	[TMEM50B]
FLJ92137AAAF	148	16.52	[RAMP3]
FLJ92138AAAF	84	9.7	[GCHFR]
FLJ92139AAAF	158	17.62	[SRGN]
FLJ92141AAAF	131	15.81	[MED31]
FLJ92142AAAF	103	11.71	[KCNE3]
FLJ92143AAAF	156	17.42	[NDUFAB1]
FLJ92145AAAF	80	9.16	[COX7B]
FLJ92146AAAF	202	22.77	[RGS16]
FLJ92147AAAF	122	14.28	[LYRM1]

FLJ92148AAAF	114	13.21	[S100A9]
FLJ92149AAAF	125	13.74	[RPS25]
FLJ92150AAAF	159	17.75	[RPL29]
FLJ92151AAAF	140	16.15	[TRAPPC2L]
FLJ92152AAAF	103	11.97	[MYCBP]
FLJ92154AAAF	58	6.46	[USMG5]
FLJ92155AAAF	73	8.55	[UBL5]
FLJ92156AAAF	258	29.4	[MRPS18B]
FLJ92157AAAF	165	19.05	[EIF1AD]
FLJ92158AAAF	150	16.68	[MYL1]
FLJ92159AAAF	238	25.64	[CD63]
FLJ92160AAAF	205	22.78	[HSPB1]
FLJ92161AAAF	263	28.48	[PSMB5]
FLJ92162AAAF	187	21.69	[TNNI1]
FLJ92165AAAF	108	12.59	[ATP5J]
FLJ92166AAAF	123	13.84	[RABIF]
FLJ92167AAAF	134	14.65	[CCL21]
FLJ92168AAAF	97	10.74	[CARD16]
FLJ92172AAAF	133	15.25	[EPPIN]
FLJ92173AAAF	162	17.59	[KRTCAP2]
FLJ92174AAAF	125	14.58	[SF3B6]
FLJ92175AAAF	135	15.86	[RPL32]
FLJ92177AAAF	128	14.19	[SH2D1A]
FLJ92178AAAF	126	13.18	[C12orf57]
FLJ92180AAAF	127	14.3	[MPC2]
FLJ92181AAAF	165	18.43	[UBD]
FLJ92182AAAF	224	25.18	[FKBP3]
FLJ92183AAAF	103	11.37	[HIST4H4]
FLJ92184AAAF	142	16.39	[BIRC5]
FLJ92185AAAF	137	15.35	[ORAOV1]
FLJ92186AAAF	144	15.25	[TMEM170A]
FLJ92187AAAF	138	15.43	[GOLT1B]
FLJ92188AAAF	134	15.03	[CPLX1]
FLJ92189AAAF	113	12.82	[EIF1B]
FLJ92190AAAF	137	15.17	[CTSC]
FLJ92191AAAF	178	20.69	[MRPL13]
FLJ92192AAAF	69	7.84	[RPS28]
FLJ92194AAAF	187	21.06	[PEBP1]
FLJ92195AAAF	126	14.47	[CBY1]
FLJ92197AAAF	130	14.12	[HIST3H2A]
FLJ92198AAAF	151	16.54	[NPC2]
FLJ92199AAAF	100	11.8	[SMIM19]
FLJ92200AAAF	75	8.3	[GNG3]
FLJ92201AAAF	69	7.58	[COX8A]
FLJ92202AAAF	126	13.91	[HIST1H2BG]
FLJ92203AAAF	96	10.4	[LSM8]
FLJ92204AAAF	207	23.17	[TIMP1]
FLJ92205AAAF	204	22.88	[RPS5]

FLJ92206AAAF	72	7.97	[SPRR2A]
FLJ92207AAAF	172	19.74	[CETN2]
FLJ92215AAAF	152	17.1	[PTGES]
FLJ92216AAAF	88	10.45	[LYRM2]
FLJ92217AAAF	179	19.62	[UBE2C]
FLJ92219AAAF	135	15.55	[RPS17]
FLJ92220AAAF	44	5.03	[TMSB10]
FLJ92222AAAF	115	11.66	[RPLP2]
FLJ92223AAAF	159	17.39	[SNRPC]
FLJ92224AAAF	221	24.52	[ALKBH7]
FLJ92225AAAF	157	17.68	[NUDCD2]
FLJ92226AAAF	109	11.77	[RPRM]
FLJ92232AAAF	89	10.02	[BANF1]
FLJ92233AAAF	153	16.79	[EIF5A2]
FLJ92234AAAF	97	11.06	[SMIM8]
FLJ92236AAAF	162	18.29	[CAV2]
FLJ92237AAAF	192	21.54	[TMEM11]
FLJ92239AAAF	91	10.74	[UQCRH]
FLJ92240AAAF	175	19.72	[IMMP2L]
FLJ92241AAAF	124	13.69	[RPP14]
FLJ92243AAAF	93	10.62	[CCL22]
FLJ92244AAAF	171	18.93	[CD3D]
FLJ92245AAAF	116	13.08	[CGA]
FLJ92246AAAF	277	30.15	[CLPP]
FLJ92247AAAF	222	25.1	[CHMP2A]
FLJ92248AAAF	146	16.56	[THRSP]
FLJ92250AAAF	135	15.91	[RBP5]
FLJ92251AAAF	173	18.91	[GCSH]
FLJ92252AAAF	177	19.21	[PPIH]
FLJ92253AAAF	245	26.02	[C1QA]
FLJ92255AAAF	113	12.74	[RNF7]
FLJ92257AAAF	129	13.42	[LOC84214]
FLJ92259AAAF	117	13.19	[SUPT4H1]
FLJ92260AAAF	51	6.82	[PRM1]
FLJ92261AAAF	134	14.92	[ID2]
FLJ92263AAAF	105	12.25	[RPL36]
FLJ92264AAAF	136	15.4	[HIST1H3H]
FLJ92265AAAF	157	17.78	[RPL24]
FLJ92266AAAF	181	20.56	[MCTS1]
FLJ92268AAAF	135	15.71	[GCSAML]
FLJ92271AAAF	67	7.65	[POLR2L]
FLJ92272AAAF	110	12.07	[TUSC2]
FLJ92273AAAF	142	14.69	[ATP5G3]
FLJ92274AAAF	189	19.89	[PARK7]
FLJ92275AAAF	103	11.78	[MRPL36]
FLJ92277AAAF	142	17.02	[AP2S1]
FLJ92278AAAF	244	28.07	[C14orf166]
FLJ92280AAAF	76	8.79	[TRIAP1]

FLJ92281AAAF	138	15.69	[CRABP2]
FLJ92282AAAF	261	29.95	[EMC3]
FLJ92283AAAF	109	12.15	[COX6A1]
FLJ92286AAAF	118	12.58	[EIF4EBP1]
FLJ92287AAAF	152	17.31	[UBE2B]
FLJ92288AAAF	166	18.79	[MYL2]
FLJ92289AAAF	159	18.1	[JCHAIN]
FLJ92290AAAF	109	12.51	[GTF2A2]
FLJ92292AAAF	108	12.2	[CISD1]
FLJ92293AAAF	98	11.33	[SNAPC5]
FLJ92294AAAF	103	11.37	[HIST1H4A]
FLJ92296AAAF	116	12.77	[SMCP]
FLJ92297AAAF	133	15.51	[PDRG1]
FLJ92298AAAF	206	21.2	[PTPRCAP]
FLJ92301AAAF	248	27.89	[PSMA7]
FLJ92303AAAF	134	15.54	[RBP7]
FLJ92305AAAF	103	12.32	[MGP]
FLJ92306AAAF	176	19.13	[MAL2]
FLJ92308AAAF	76	7.85	[ADIRF]
FLJ92309AAAF	128	13.81	[GYPC]
FLJ92311AAAF	100	11.18	[APOA2]
FLJ92313AAAF	83	9.33	[APOC1]
FLJ92314AAAF	73	8.48	[GNG11]
FLJ92316AAAF	102	11.15	[PAGE4]
FLJ92317AAAF	139	16.09	[LGALS14]
FLJ92318AAAF	155	18.86	[SNRNP27]
FLJ92319AAAF	237	26.83	[REXO2]
FLJ92320AAAF	244	27.54	[GSTT2]
FLJ92321AAAF	195	21.93	[MYL3]
FLJ92322AAAF	128	15.16	[NDUFA6]
FLJ92324AAAF	97	10.63	[CYSTM1]
FLJ92325AAAF	164	17.6	[TMEM234]
FLJ92326AAAF	172	19.57	[CETN1]
FLJ92327AAAF	76	7.91	[PKIG]
FLJ92331AAAF	125	14.2	[TRMT112]
FLJ92332AAAF	147	15.89	[TTR]
FLJ92333AAAF	126	13.89	[HIST1H2BH]
FLJ92334AAAF	56	6.57	[UQCR11]
FLJ92335AAAF	137	14.26	[SRXN1]
FLJ92336AAAF	127	14.17	[HIST1H2BA]
FLJ92337AAAF	280	31.63	[SDHB]
FLJ92338AAAF	166	18.66	[REG1B]
FLJ92339AAAF	112	13.19	[FAM32A]
FLJ92340AAAF	134	15.3	[PFDN4]
FLJ92341AAAF	151	17.22	[RPS13]
FLJ92343AAAF	121	13.87	[C1orf21]
FLJ92344AAAF	99	10.71	[TMEM14A]
FLJ92346AAAF	123	13.01	[FGF5]

FLJ92347AAAF	170	19.79	[NTS]
FLJ92348AAAF	305	33.97	[GNPTG]
FLJ92349AAAF	81	9.37	[ATP6V0E1]
FLJ92350AAAF	81	9.24	[NNAT]
FLJ92351AAAF	84	9.46	[RPS27]
FLJ92353AAAF	157	18.25	[UXT]
FLJ92355AAAF	264	28.88	[GZMK]
FLJ92356AAAF	134	14.12	[SMR3A]
FLJ92358AAAF	188	21.04	[PRADC1]
FLJ92360AAAF	86	10.14	[SF3B5]
FLJ92361AAAF	112	12.38	[CNFN]
FLJ92362AAAF	195	21.53	[TMEM126A]
FLJ92363AAAF	114	12.65	[PYURF]
FLJ92365AAAF	125	14.23	[MRPS6]
FLJ92366AAAF	128	15.32	[BEX2]
FLJ92367AAAF	145	15.95	[MRPL14]
FLJ92369AAAF	90	10.11	[S100A6]
FLJ92370AAAF	122	11.54	[IFI27]
FLJ92371AAAF	189	21.28	[APOD]
FLJ92372AAAF	79	9.12	[COX7A1]
FLJ92373AAAF	176	20.75	[VPS25]
FLJ92376AAAF	89	10.38	[TIMM9]
FLJ92378AAAF	126	13.91	[HIST1H2BI]
FLJ92379AAAF	31	3.76	[SLN]
FLJ92380AAAF	123	13.71	[VPREB3]
FLJ92381AAAF	58	6.66	[C14orf2]
FLJ92383AAAF	99	11.26	[ACYP1]
FLJ92384AAAF	115	12.78	[RPL30]
FLJ92385AAAF	152	16.52	[MGST3]
FLJ92386AAAF	102	10.93	[HSPE1]
FLJ92388AAAF	146	16.36	[JTB]
FLJ92389AAAF	180	20.53	[ARF5]
FLJ92390AAAF	114	12.85	[MSMB]
FLJ92391AAAF	202	22.09	[CCDC85B]
FLJ92392AAAF	164	18.18	[RARRES3]
FLJ92393AAAF	68	7.32	[GNG5]
FLJ92394AAAF	147	17.28	[CST9L]
FLJ92395AAAF	146	17.16	[MAGOH]
FLJ92396AAAF	126	13.95	[HIST1H2BL]
FLJ92397AAAF	127	13.89	[PHLDA3]
FLJ92398AAAF	154	15.94	[SOD1]
FLJ92399AAAF	97	11	[TIMM8A]
FLJ92400AAAF	141	15.79	[POMP]
FLJ92401AAAF	70	8.22	[RPL38]
FLJ92402AAAF	127	14.28	[CCL28]
FLJ92405AAAF	156	17.7	[RPL23A]
FLJ92407AAAF	89	10.35	[DYNLL2]
FLJ92408AAAF	80	9.13	[LSM6]

FLJ92409AAAF	130	14.14	[HIST1H2AD]
FLJ92411AAAF	249	27.36	[NDUFV2]
FLJ92412AAAF	92	10.8	[SNRPE]
FLJ92413AAAF	172	19.29	[POLR2G]
FLJ92414AAAF	273	31	[ZNF32]
FLJ92415AAAF	98	11.14	[CSTB]
FLJ92416AAAF	88	9.56	[SMPX]
FLJ92417AAAF	82	8.86	[NUPR1]
FLJ92418AAAF	154	17.55	[ZNHIT1]
FLJ92419AAAF	206	22.12	[BLVRB]
FLJ92420AAAF	227	25.86	[TCEAL2]
FLJ92421AAAF	67	7.95	[GNG13]
FLJ92423AAAF	86	10.19	[COX6B1]
FLJ92424AAAF	127	13.33	[SNCG]
FLJ92425AAAF	107	11.44	[SMDT1]
FLJ92426AAAF	63	6.92	[COX17]
FLJ92428AAAF	76	9.2	[SPTSSB]
FLJ92430AAAF	98	11.08	[SCRG1]
FLJ92434AAAF	118	13.16	[MLANA]
FLJ92435AAAF	129	14.58	[PFDN6]
FLJ92436AAAF	158	18.73	[AP1S1]
FLJ92437AAAF	94	10.55	[S100A1]
FLJ92440AAAF	83	9.4	[COX7A2]
FLJ92441AAAF	75	8.74	[BRK1]
FLJ92442AAAF	97	10.85	[NPY]
FLJ92444AAAF	124	14.29	[TAF13]
FLJ92445AAAF	133	15.18	[LSM1]
FLJ92446AAAF	126	13.32	[C15orf40]
FLJ92447AAAF	135	14.72	[LGALS1]
FLJ92448AAAF	171	20.02	[SAT1]
FLJ92449AAAF	130	14.1	[HIST2H2AA3]
FLJ92450AAAF	128	13.29	[LY6D]
FLJ92454AAAF	140	15.05	[PFN1]
FLJ92455AAAF	83	9.34	[TIMM8B]
FLJ92457AAAF	219	25.18	[PRELID1]
FLJ92459AAAF	205	23.75	[MND1]
FLJ92460AAAF	293	31.32	[RPS2]
FLJ92464AAAF	116	12.74	[SST]
FLJ92466AAAF	151	16.27	[RPS14]
FLJ92467AAAF	57	6.6	[COX14]
FLJ92468AAAF	212	24.24	[NME5]
FLJ92469AAAF	130	14.81	[SAA4]
FLJ92470AAAF	96	10.99	[CCL1]
FLJ92471AAAF	105	11.72	[S100A11]
FLJ92472AAAF	149	16.83	[OSTC]
FLJ92475AAAF	106	12.52	[NDUFS5]
FLJ92476AAAF	102	11.83	[LSM3]
FLJ92477AAAF	104	12.75	[WBP5]

FLJ92478AAAF	89	9.9	[SPRR1B]
FLJ92479AAAF	132	14.33	[SLPI]
FLJ92482AAAF	222	25.25	[PRG2]
FLJ92484AAAF	163	18.62	[RARRES2]
FLJ92487AAAF	247	26.56	[PRSS1]
FLJ92488AAAF	255	28.42	[SNRPA1]
FLJ92489AAAF	109	12.35	[MPC1]
FLJ92490AAAF	225	24.96	[RAB32]
FLJ92493AAAF	297	33.6	[TATDN1]
FLJ92494AAAF	117	13.92	[GABARAP]
FLJ92495AAAF	125	14.52	[POLR2I]
FLJ92497AAAF	273	28.94	[PSMB10]
FLJ92498AAAF	202	22.02	[PTTG1]
FLJ92499AAAF	77	7.78	[LTB]
FLJ92500AAAF	249	28.72	[PSME1]
FLJ92502AAAF	146	15.8	[CST3]
FLJ92503AAAF	119	13.37	[ATP6V1F]
FLJ92504AAAF	230	25.45	[TREM2]
FLJ92505AAAF	119	13.13	[CCL24]
FLJ92506AAAF	136	15.51	[HIST3H3]
FLJ92507AAAF	165	17.36	[C20orf141]
FLJ92508AAAF	110	12.34	[CHCHD5]
FLJ92511AAAF	84	9.29	[SPINK2]
FLJ92512AAAF	281	29.27	[PTCRA]
FLJ92513AAAF	130	14.09	[HIST1H2AG]
FLJ92514AAAF	77	8.84	[SS18L2]
FLJ92518AAAF	146	16.57	[PIP]
FLJ92520AAAF	165	18.23	[ALG13]
FLJ92521AAAF	161	18.49	[ATP5H]
FLJ92522AAAF	153	17.03	[IGF1]
FLJ92523AAAF	128	13.91	[HIST1H2AH]
FLJ92524AAAF	101	12.03	[PSENEN]
FLJ92525AAAF	665	73.25	[KIF22]
FLJ92527AAAN	508	57.31	[CYP17A1]
FLJ92529AAAF	294	35.13	[SULT1E1]
FLJ92530AAAF	677	78.27	[CHGB]
FLJ92533AAAF	305	35.38	[TSPAN12]
FLJ92534AAAF	573	61.06	[HSPD1]
FLJ92536AAAF	414	46.65	[IDH1]
FLJ92537AAAF	727	83.5	[PLOD1]
FLJ92544AAAF	348	39.69	[LMAN2L]
FLJ92545AAAF	205	23.26	[RGS4]
FLJ92546AAAF	758	83.69	[IMMT]
FLJ92547AAAF	354	40.53	[GNAI3]
FLJ92548AAAF	531	57.94	[PKM]
FLJ92549AAAF	562	61.4	[PGM1]
FLJ92550AAAF	724	83.26	[HSP90AB1]
FLJ92552AAAF	666	74.82	[SEMA7A]

FLJ92553AAAN	615	70.73	[LBR]
FLJ92554AAAF	765	86.15	[SEC23A]
FLJ92556AAAF	308	34.99	[FSTL1]
FLJ92557AAAF	497	54.55	[TXNRD1]
FLJ92560AAAF	1041	119.26	[DHX16]
FLJ92561AAAF	184	20.7	[GREM1]
FLJ92562AAAF	774	86.75	[LOXL2]
FLJ92563AAAF	427	48.29	[VDR]
FLJ92565AAAF	549	60.91	[SLC33A1]
FLJ92566AAAF	417	46.92	[LOX]
FLJ92568AAAF	795	87.32	[ALDH18A1]
FLJ92570AAAF	371	41.45	[ELF3]
FLJ92574AAAF	253	27.59	[PRNP]
FLJ92575AAAF	530	58.65	[GP2]
FLJ92578AAAF	560	62.61	[GPNMB]
FLJ92579AAAF	417	44.61	[PGK1]
FLJ92580AAAF	364	39.46	[ALDOC]
FLJ92581AAAF	381	42.67	[CKB]
FLJ92583AAAF	350	39.35	[GYG1]
FLJ92588AAAF	561	62.24	[SH3BP2]
FLJ92591AAAF	616	65.34	[RFX5]
FLJ92593AAAF	456	50.23	[RUVBL1]
FLJ92595AAAF	485	53.21	[AGT]
FLJ92596AAAF	650	72.1	[APLP1]
FLJ92598AAAF	469	51.32	[POLD2]
FLJ92599AAAF	350	38.44	[STRAP]
FLJ92600AAAF	466	52.3	[MPP1]
FLJ92602AAAF	517	57.4	[PPAT]
FLJ92603AAAF	511	57.21	[ARCN1]
FLJ92604AAAF	463	52.34	[PNMA3]
FLJ92607AAAF	697	78.46	[PRKCG]
FLJ92608AAAF	512	56.12	[ALDH1A3]
FLJ92609AAAF	542	59.57	[SLC1A3]
FLJ92611AAAF	971	110.42	[CSE1L]
FLJ92613AAAF	844	95.15	[GABBR1]
FLJ92615AAAF	806	89.31	[VCP]
FLJ92616AAAF	475	53.16	[LPL]
FLJ92619AAAF	359	41.52	[SCD]
FLJ92629AAAF	484	53.91	[SUFU]
FLJ92630AAAF	144	16.46	[EIF1AX]
FLJ92632AAAF	672	74.48	[ZBTB33]
FLJ92633AAAF	998	114.2	[CEBPZ]
FLJ92634AAAF	203	23.06	[RAB30]
FLJ92636AAAF	382	43.01	[GJA1]
FLJ92639AAAF	520	56.1	[OXCT1]
FLJ92643AAAF	327	37.19	[PPP1CB]
FLJ92645AAAF	334	36.43	[MDH1]
FLJ92650AAAF	920	99.56	[BRD8]

FLJ92651AAAF	592	67.57	[CANX]
FLJ92652AAAF	1268	141.38	[HDLBP]
FLJ92654AAAF	298	32.87	[SLC25A6]
FLJ92656AAAF	361	37.83	[SSBP2]
FLJ92658AAAF	1014	113.09	[PARP1]
FLJ92659AAAF	277	30.08	[PLP1]
FLJ92660AAAF	638	70.57	[PCSK2]
FLJ92661AAAF	281	31.98	[MAPRE3]
FLJ92662AAAF	249	27.57	[TSPAN7]
FLJ92663AAAF	212	23.55	[RAB2A]
FLJ92664AAAF	144	15.68	[TSC22D1]
FLJ92665AAAF	509	54.18	[DLD]
FLJ92666AAAF	204	23.19	[ARHGDIA]
FLJ92668AAAF	217	24.01	[GCA]
FLJ92669AAAF	103	11.64	[SUMO3]
FLJ92670AAAF	254	28.8	[PGAM1]
FLJ92676AAAF	197	20.92	[CD164]
FLJ92678AAAF	482	55.95	[IFIT5]
FLJ92679AAAF	171	19.09	[HMP19]
FLJ92681AAAF	935	101.53	[MTHFD1]
FLJ92684AAAF	557	65.62	[IK]
FLJ92685AAAF	423	46.8	[TMEM130]
FLJ92687AAAF	334	37.55	[MAT2B]
FLJ92688AAAF	750	84.33	[FOLH1]
FLJ92689AAAF	374	39.72	[ADH5]
FLJ92690AAAF	274	30.87	[NQO1]
FLJ92691AAAF	607	68.57	[RPN1]
FLJ92692AAAF	329	36.41	[CNN3]
FLJ92694AAAF	184	20.82	[RAP1B]
FLJ92695AAAF	511	56.5	[ATP6V1B2]
FLJ92697AAAF	355	40.44	[CX3CR1]
FLJ92698AAAF	460	51.42	[CHRM1]
FLJ92700AAAF	718	82.25	[EXT2]
FLJ92701AAAF	657	73.86	[PTPRR]
FLJ92703AAAF	638	69.81	[SRPR]
FLJ92705AAAF	208	23.46	[RAB6B]
FLJ92706AAAF	820	95.58	[DDX23]
FLJ92707AAAF	986	109.85	[EPHA4]
FLJ92709AAAF	399	45.3	[LANCL1]
FLJ92713AAAF	379	42.52	[KCNJ10]
FLJ92714AAAF	219	24.98	[TMED10]
FLJ92716AAAF	496	53.92	[SLC2A3]
FLJ92717AAAF	316	36.2	[TNFAIP1]
FLJ92718AAAF	563	61.27	[TPP1]
FLJ92720AAAF	535	57.84	[ALDH6A1]
FLJ92721AAAF	862	95.79	[PMS2]
FLJ92722AAAF	630	70.81	[PLS3]
FLJ92724AAAF	627	70.29	[LCP1]

FLJ92728AAAF	471	51.93	[HSPA13]
FLJ92729AAAF	826	92.69	[HIF1A]
FLJ92732AAAF	352	39.96	[SH3GL2]
FLJ92734AAAF	434	47.27	[ENO2]
FLJ92735AAAF	492	54.08	[SLC2A1]
FLJ92736AAAF	492	55.18	[GABRA3]
FLJ92739AAAF	84	9.22	[CDC42SE2]
FLJ92740AAAF	597	68.48	[RNGTT]
FLJ92742AAAF	713	78.86	[LRRN2]
FLJ92743AAAF	131	14.69	[MMGT1]
FLJ92745AAAF	754	84.96	[NBN]
FLJ92747SAAN	706	81.86	[PRKCQ]
FLJ92752AAAF	1049	114.48	[ITGA5]
FLJ92753AAAF	510	54.64	[FH]
FLJ92755AAAF	414	44.78	[TARDBP]
FLJ92756AAAF	640	73.22	[GBP4]
FLJ92759AAAF	841	94.59	[NEK4]
FLJ92760AAAF	683	77.3	[ZNF263]
FLJ92761AAAF	457	51.21	[FKBP5]
FLJ92762AAAF	785	88.21	[USP1]
FLJ92764AAAF	784	87.6	[CDH5]
FLJ92767AAAF	639	73.06	[GALNT15]
FLJ92770AAAF	165	19.25	[CHIC2]
FLJ92772AAAF	478	51.5	[SHPK]
FLJ92773AAAF	785	86.25	[PCSK7]
FLJ92777AAAF	1043	118.41	[MYO1A]
FLJ92778AAAF	285	31.12	[DDAH1]
FLJ92779AAAF	406	44.74	[NIPAL3]
FLJ92780AAAF	941	105.74	[PDE2A]
FLJ92781AAAF	685	78.09	[RNF6]
FLJ92782AAAF	1140	126.97	[DDB1]
FLJ92783AAAF	660	73.18	[DDX3Y]
FLJ92784AAAF	888	97.46	[HMGCR]
FLJ92785AAAF	662	73.82	[AKAP10]
FLJ92787AAAF	311	35.12	[PPAP2B]
FLJ92788AAAF	255	29.18	[STX6]
FLJ92790AAAF	418	46.29	[PELI1]
FLJ92791AAAF	315	36.11	[EIF2S1]
FLJ92794AAAF	551	59.21	[CPSF6]
FLJ92795AAAF	357	38.31	[SORD]
FLJ92796AAAF	440	49.61	[OMG]
FLJ92797AAAF	315	37.11	[CALU]
FLJ92798AAAF	488	52.64	[ANXA7]
FLJ92802AAAF	381	41.47	[CD55]
FLJ92803AAAF	736	79.61	[HSD17B4]
FLJ92804AAAF	94	11.45	[HOXA10]
FLJ92806AAAF	440	47.69	[SQSTM1]
FLJ92807AAAF	617	70.94	[SCG2]

FLJ92808AAAF	206	23.41	[RALB]
FLJ92809AAAF	451	52.33	[FARS2]
FLJ92810AAAF	546	59.24	[PPM1G]
FLJ92811AAAF	504	54.64	[TAB1]
FLJ92812AAAF	317	36.47	[RLBP1]
FLJ92813AAAF	482	53.86	[C3AR1]
FLJ92814AAAF	511	55.37	[ALDH7A1]
FLJ92815AAAF	355	41.57	[GNA14]
FLJ92816AAAF	198	22.07	[CDKN1B]
FLJ92819AAAF	496	55.69	[SLC19A3]
FLJ92820AAAF	265	29.17	[SYNPR]
FLJ92821AAAF	216	24.42	[RAN]
FLJ92823AAAF	326	36.9	[RPRD1B]
FLJ92825AAAF	198	22.29	[SAR1A]
FLJ92827AAAF	847	94.17	[STAT6]
FLJ92829AAAF	473	51.19	[MEF2C]
FLJ92833AAAF	319	35.58	[OR7A5]
FLJ92834AAAF	423	47.87	[COPS3]
FLJ92835AAAF	512	57.53	[FRS2]
FLJ92839AAAF	244	27.1	[CLDN12]
FLJ92840AAAF	354	40.28	[HOMER1]
FLJ92842AAAF	279	31.88	[CMSS1]
FLJ92843AAAF	354	38.58	[ARG2]
FLJ92845AAAF	445	50.66	[GDI2]
FLJ92848AAAF	915	102.16	[GRM7]
FLJ92849AAAF	732	83.42	[LSS]
FLJ92851AAAF	493	56.02	[CDKL2]
FLJ92853AAAF	474	52.39	[GSS]
FLJ92854AAAF	382	42.81	[S1PR1]
FLJ92856AAAF	270	30.35	[PRKAB1]
FLJ92858AAAF	318	35.72	[BST1]
FLJ92861AAAF	398	44	[SERPINE2]
FLJ92862AAAF	500	53.91	[SLC16A1]
FLJ92865AAAF	536	59.7	[GBA]
FLJ92867AAAF	505	55.21	[RTCB]
FLJ92868AAAF	556	61.89	[HIRIP3]
FLJ92870AAAF	376	42.61	[ACTR1A]
FLJ92871AAAF	198	21.64	[SDC4]
FLJ92872AAAF	350	37.37	[MCL1]
FLJ92873AAAF	412	45.51	[BHLHE40]
FLJ92875AAAF	437	49.03	[ETF1]
FLJ92881AAAF	349	37.6	[GPD1]
FLJ92884AAAF	355	40.92	[GNAZ]
FLJ92885AAAF	277	31.54	[RSU1]
FLJ92887AAAF	504	54.97	[SIRPA]
FLJ92888AAAF	185	21.12	[REEP5]
FLJ92889AAAF	708	81.85	[EXOC5]
FLJ92890AAAF	977	112.07	[TRPC4]

FLJ92891AAAF	182	20.46	[ARL3]
FLJ92892AAAF	173	19.82	[PTP4A1]
FLJ92896AAAF	953	105.84	[PSMD1]
FLJ92897AAAF	435	49.97	[PTPN1]
FLJ92898AAAF	309	35.78	[AASDHPPT]
FLJ92899AAAF	486	56.19	[PPP2R5A]
FLJ92900AAAF	467	52.67	[PSEN1]
FLJ92901AAAN	246	28.05	[PMM2]
FLJ92902AAAF	734	82.29	[MCM5]
FLJ92907AAAF	201	23.03	[RAB35]
FLJ92908AAAF	722	79.27	[GNE]
FLJ92910AAAF	469	51.63	[PDCD4]
FLJ92912AAAF	496	56.88	[ANGPT2]
FLJ92913AAAF	344	37.24	[CEACAM6]
FLJ92915AAAN	356	40.56	[RFC3]
FLJ92917AAAF	328	37.15	[BUB3]
FLJ92918AAAF	412	47.91	[SUV39H1]
FLJ92921AAAN	748	84.08	[ADAM10]
FLJ92922AAAF	747	85.65	[TPX2]
FLJ92923AAAF	190	21.49	[LZIC]
FLJ92924AAAF	882	97.46	[CDH1]
FLJ92925AAAF	330	36.29	[ALAD]
FLJ92926AAAF	164	19.33	[SRSF3]
FLJ92927AAAF	829	91.43	[CDH3]
FLJ92928AAAF	350	39.63	[RP2]
FLJ92929AAAF	614	69.5	[PAPSS2]
FLJ92930AAAF	662	74.73	[ALOX15]
FLJ92931AAAF	246	29.1	[TFAM]
FLJ92933AAAF	262	29.41	[TTC33]
FLJ92935AAAF	439	48.53	[OAT]
FLJ92940AAAF	993	109.15	[IGHMBP2]
FLJ92941AAAF	640	70.58	[ALAS1]
FLJ92942AAAF	682	74.06	[TBR1]
FLJ92943AAAF	393	43.71	[TP53]
FLJ92944AAAF	352	39.59	[TMOD3]
FLJ92945AAAF	562	66.2	[ZMYND11]
FLJ92946AAAF	425	47.66	[RBBP4]
FLJ92947AAAF	466	52.16	[G3BP1]
FLJ92949AAAN	427	45.2	[ACAT1]
FLJ92950AAAF	821	92.89	[MCM6]
FLJ92951AAAF	898	102.19	[KIF18A]
FLJ92953AAAF	384	44.39	[NPY1R]
FLJ92954AAAF	251	27.59	[TAF9B]
FLJ92955AAAF	923	104.23	[TNPO3]
FLJ92956AAAF	752	83.27	[C2]
FLJ92957AAAF	195	22.46	[CHP1]
FLJ92958AAAF	194	20.86	[H1F0]
FLJ92959AAAF	276	31.16	[SLA]

FLJ92960AAAF	372	42.78	[INPP5K]
FLJ92962AAAF	370	41.99	[GPR85]
FLJ92963AAAF	157	17.4	[TMEM50A]
FLJ92965AAAF	247	27.59	[STC1]
FLJ92966AAAF	268	30	[MAPRE1]
FLJ92967AAAF	452	52	[GLRA2]
FLJ92968AAAF	577	64.37	[RUNX1T1]
FLJ92972AAAF	739	81.68	[SLC26A2]
FLJ92973AAAF	586	69.4	[EZR]
FLJ92974AAAF	250	27.36	[MMAB]
FLJ92975AAAF	375	42.8	[NAP1L4]
FLJ92979AAAF	334	36.73	[GPR12]
FLJ92980AAAF	586	66.41	[LMNB1]
FLJ92982AAAF	556	62.41	[GPC4]
FLJ92988AAAF	756	84.57	[MLH1]
FLJ92992AAAF	750	83.03	[MUT]
FLJ92993AAAF	436	48.35	[FBXL20]
FLJ92994AAAF	154	16.01	[HN1]
FLJ92996AAAF	340	37.3	[GNB1]
FLJ92997AAAF	185	21.42	[CBX1]
FLJ92998AAAF	375	42.67	[DEK]
FLJ93000AAAF	215	23.66	[RAB5A]
FLJ93002AAAF	266	30.5	[RBM7]
FLJ93004AAAF	231	23.66	[SNRPB]
FLJ93005AAAF	445	49.9	[TUBB2A]
FLJ93006AAAF	198	22.42	[YKT6]
FLJ93007AAAF	469	54.04	[PFKFB4]
FLJ93008AAAF	340	38.04	[DNAJB1]
FLJ93009AAAF	210	24.61	[POLR2E]
FLJ93010AAAF	500	55.15	[SERPING1]
FLJ93011AAAF	452	50.91	[IDH2]
FLJ93013AAAF	421	46.59	[ACADM]
FLJ93015AAAF	557	63.47	[IFNAR1]
FLJ93016AAAF	377	42.56	[CCNI]
FLJ93018AAAF	299	35.3	[SC5D]
FLJ93019AAAF	359	40.8	[PEX12]
FLJ93020AAAF	532	57.83	[ICAM1]
FLJ93021AAAF	356	39.39	[PRPSAP1]
FLJ93022AAAF	295	33.04	[F3]
FLJ93025AAAF	387	43.1	[MFGE8]
FLJ93029AAAF	443	51.62	[COPS2]
FLJ93030AAAF	320	35.94	[ANXA5]
FLJ93032AAAF	208	22.55	[ZFAND6]
FLJ93033AAAF	212	24.54	[KDELR1]
FLJ93035AAAF	272	29.8	[PHB]
FLJ93036AAAF	246	28.12	[YWHAH]
FLJ93038AAAF	450	51.89	[BECN1]
FLJ93041AAAF	349	38.15	[CEACAM8]

FLJ93042AAAF	286	32.19	[SSR1]
FLJ93044AAAN	464	52.6	[SERPINC1]
FLJ93045AAAF	522	57.95	[GALNS]
FLJ93047AAAF	582	65.85	[MMP14]
FLJ93048AAAF	220	25	[VAMP7]
FLJ93049AAAF	418	47.37	[ACTR3]
FLJ93051AAAF	268	29.99	[SNAI2]
FLJ93053AAAF	332	37.53	[CHORDC1]
FLJ93054AAAF	233	26.14	[FCGR3B]
FLJ93056AAAF	360	40.76	[CXCR2]
FLJ93058AAAF	526	59.79	[NCF2]
FLJ93060AAAF	350	39.79	[CXCR1]
FLJ93063AAAF	361	42.1	[RSAD2]
FLJ93064AAAF	219	25.07	[CLEC4E]
FLJ93065AAAF	439	50.23	[FGL2]
FLJ93066AAAF	372	42.19	[SELL]
FLJ93067AAAF	489	54.38	[IFNGR1]
FLJ93069AAAF	127	14.81	[MRFAP1L1]
FLJ93070AAAF	267	29.61	[CD82]
FLJ93071AAAF	738	83.21	[COG2]
FLJ93072AAAF	520	58.76	[MAOB]
FLJ93074AAAF	346	39.22	[TBCC]
FLJ93075AAAF	338	38.43	[LUM]
FLJ93076AAAF	499	56.83	[PPP5C]
FLJ93078AAAF	413	46.25	[GOT1]
FLJ93079AAAF	322	35.27	[MYADM]
FLJ93080AAAF	505	57.03	[UAP1]
FLJ93081AAAF	543	60.83	[CYP1B1]
FLJ93082AAAF	658	73.78	[CPT2]
FLJ93084AAAF	277	30.97	[M6PR]
FLJ93086AAAF	374	42.13	[SPOP]
FLJ93089AAAF	377	42.91	[NCK1]
FLJ93091AAAF	228	25.85	[CMPK1]
FLJ93092AAAF	407	44.24	[ELK3]
FLJ93093AAAN	906	99.74	[CDH2]
FLJ93094AAAF	259	28.45	[DKK2]
FLJ93095AAAF	391	43.77	[WNT2B]
FLJ93096AAAN	1093	122.86	[TMF1]
FLJ93097AAAF	357	41.48	[LRPAP1]
FLJ93098AAAF	1241	137.01	[SLC4A2]
FLJ93100AAAF	641	74.59	[CAPN6]
FLJ93101AAAF	449	51.69	[GLRA1]
FLJ93102AAAF	249	28.59	[ANP32A]
FLJ93103AAAF	796	87.5	[NTRK1]
FLJ93104AAAF	381	41.98	[PTX3]
FLJ93105AAAF	318	34.69	[PRPS1]
FLJ93107AAAF	297	33.01	[DSCR3]
FLJ93108AAAF	298	33.21	[JAM2]

FLJ93109AAAF	1050	119.53	[BUB1B]
FLJ93112AAAF	660	73.79	[MMP2]
FLJ93116AAAF	966	108.3	[MAP3K13]
FLJ93118AAAF	557	64.12	[ARIH1]
FLJ93119AAAF	348	38.74	[APOL4]
FLJ93122AAAF	443	48.88	[COX10]
FLJ93123AAAF	632	70.57	[SLC6A11]
FLJ93124AAAF	474	54.23	[GABRB1]
FLJ93125AAAF	227	26.22	[NUDT21]
FLJ93126AAAF	1218	133.86	[JAG1]
FLJ93128AAAF	324	35.58	[DNAJB2]
FLJ93130AAAF	213	22.73	[ZMYM5]
FLJ93132AAAF	574	63.83	[CDC7]
FLJ93135AAAF	190	21.77	[MRPS23]
FLJ93136AAAF	468	51.5	[IL6R]
FLJ93137AAAF	188	21.61	[ARL6IP5]
FLJ93139AAAF	498	55.38	[MYOT]
FLJ93140AAAF	221	25.25	[MXD1]
FLJ93141AAAF	732	83.24	[F13A1]
FLJ93143AAAF	843	93.55	[C7]
FLJ93144AAAF	679	73.7	[SLC20A1]
FLJ93145AAAF	870	100.4	[RASA1]
FLJ93148AAAF	299	33.62	[RQCD1]
FLJ93149AAAF	325	36.18	[FRZB]
FLJ93151AAAF	738	84.79	[PLOD3]
FLJ93153AAAF	424	48.51	[KRT20]
FLJ93156AAAF	341	36.69	[CREB1]
FLJ93157AAAF	707	76.34	[SHKBP1]
FLJ93162AAAF	359	40.02	[CYTIP]
FLJ93164AAAF	555	61.38	[PRF1]
FLJ93166AAAF	643	70.96	[HSPA6]
FLJ93167AAAF	207	22.04	[PPIF]
FLJ93168AAAF	717	82.08	[ST5]
FLJ93169AAAF	621	67.59	[GPAA1]
FLJ93170AAAF	238	27.67	[MMD]
FLJ93171AAAF	380	42.59	[FEN1]
FLJ93174AAAF	173	17.42	[LOC162137]
FLJ93175AAAF	375	42.11	[SERPINB5]
FLJ93176AAAF	495	53.53	[CRNN]
FLJ93177AAAF	461	51.15	[ODC1]
FLJ93181AAAF	241	27.46	[FAM71C]
FLJ93184AAAF	509	57.89	[PRAME]
FLJ93185AAAF	417	44.8	[PGK2]
FLJ93186AAAF	514	58.78	[SARS]
FLJ93190AAAF	431	48.36	[CSTF1]
FLJ93191AAAF	475	54.52	[DHCR7]
FLJ93194AAAF	419	47.97	[MOK]
FLJ93195AAAF	321	36.09	[NDN]

FLJ93197AAAF	499	54.72	[CDC20]
FLJ93198AAAF	248	27.74	[SRSF1]
FLJ93199AAAF	543	61.36	[ACRBP]
FLJ93203AAAF	688	77.07	[ZBTB48]
FLJ93205AAAF	229	26.02	[TMED5]
FLJ93207AAAF	749	83.98	[MAP7]
FLJ93210AAAF	870	98.91	[WWP2]
FLJ93211AAAF	617	69.33	[SLC6A2]
FLJ93213AAAF	787	86.35	[PCDHB15]
FLJ93218AAAF	398	43.92	[B4GALT1]
FLJ93220AAAF	378	42.25	[S1PR3]
FLJ93223AAAF	293	34.96	[ELMOD2]
FLJ93228AAAF	192	21.99	[PMVK]
FLJ93230AAAF	751	86.24	[ZNF184]
FLJ93232AAAF	349	37.22	[TRUB1]
FLJ93235AAAF	334	37.37	[CTSV]
FLJ93236AAAF	790	91.8	[NCBP1]
FLJ93237AAAF	291	33.76	[IMP4]
FLJ93240AAAF	1019	117.81	[IDE]
FLJ93241AAAF	515	58.84	[GJA9]
FLJ93242AAAF	427	48.72	[EDNRA]
FLJ93244AAAF	476	50.3	[EGR2]
FLJ93246AAAF	413	44.12	[FAM8A1]
FLJ93248AAAF	572	63.71	[GPC5]
FLJ93255AAAF	629	67.65	[SLC7A1]
FLJ93257AAAF	699	79.79	[ECM2]
FLJ93259AAAF	448	48.59	[EVI2B]
FLJ93260AAAF	277	30.19	[IMPA1]
FLJ93262AAAF	538	59.1	[IL21R]
FLJ93263AAAF	547	59.52	[ICAM3]
FLJ93265AAAF	458	51.11	[CD4]
FLJ93267AAAF	330	37.91	[GRAP2]
FLJ93269AAAF	296	33.41	[MRPL15]
FLJ93270AAAF	319	35.63	[GPA33]
FLJ93272AAAF	824	88.71	[ADAM8]
FLJ93273AAAF	514	58.46	[CDADC1]
FLJ93274AAAF	221	24.87	[RAB27A]
FLJ93275AAAF	419	47.67	[KCNJ5]
FLJ93277AAAF	568	63.48	[SLC30A9]
FLJ93280AAAF	69	7.75	[GNGT2]
FLJ93282AAAF	590	65.26	[IRAK2]
FLJ93285AAAF	268	28.98	[CDCA3]
FLJ93287AAAF	221	22.35	[HIST1H1D]
FLJ93288AAAF	620	71.83	[ITK]
FLJ93289AAAF	319	34.76	[BTN3A2]
FLJ93290AAAF	374	42.51	[F2RL2]
FLJ93291AAAF	135	14.96	[NHLH2]
FLJ93293AAAF	201	23.6	[SNX10]

FLJ93294AAAF	387	45.02	[PHKG1]
FLJ93295AAAF	438	50.89	[GCNT3]
FLJ93296AAAF	504	55.7	[SRP54]
FLJ93297WAAF	386	41.48	[ADH7]
FLJ93299AAAF	547	59.02	[SCP2]
FLJ93300AAAF	467	53.15	[IRF6]
FLJ93301AAAF	480	53.76	[EDIL3]
FLJ93302AAAF	1053	119.38	[ZBTB11]
FLJ93303AAAF	551	61.12	[IL2RB]
FLJ93304AAAF	504	57.58	[DNAJC3]
FLJ93305AAAF	790	89.61	[TNFAIP3]
FLJ93307AAAF	477	55.1	[ETV1]
FLJ93308AAAF	960	110.43	[ERAP2]
FLJ93310AAAF	211	22.74	[CLDN1]
FLJ93311AAAF	439	51.96	[MFAP1]
FLJ93312AAAF	244	26.43	[ADIPOQ]
FLJ93313AAAF	510	57.52	[LMAN1]
FLJ93314AAAF	578	66.67	[GALNT4]
FLJ93317AAAF	832	92.15	[CDH17]
FLJ93319AAAF	525	59.11	[RPS6KB1]
FLJ93321AAAF	471	53.56	[FMO2]
FLJ93323AAAF	487	55.68	[HRH1]
FLJ93326AAAF	217	24.28	[GRPEL1]
FLJ93328AAAF	830	90.82	[SELP]
FLJ93329AAAF	1038	119.52	[IPO7]
FLJ93330AAAF	1244	134.85	[PITPNM1]
FLJ93331AAAF	188	20.94	[MOCS2]
FLJ93334AAAF	270	30.82	[GJB3]
FLJ93335AAAF	683	77.54	[PRPF3]
FLJ93336AAAF	247	28.18	[MOG]
FLJ93337AAAF	185	20.42	[ADM]
FLJ93338AAAF	480	52.56	[KLF10]
FLJ93340AAAF	330	37.47	[EXTL2]
FLJ93341AAAF	455	52.56	[BLMH]
FLJ93343AAAF	890	100.31	[WFS1]
FLJ93344AAAF	480	54.47	[CTSA]
FLJ93346AAAF	268	29.32	[RPP30]
FLJ93347AAAF	313	35.74	[ASPA]
FLJ93348AAAF	605	66.65	[PPM1D]
FLJ93349AAAF	583	68.56	[RDX]
FLJ93350AAAF	218	24.95	[PRRG1]
FLJ93352AAAF	280	31.79	[TMX1]
FLJ93353AAAF	389	43	[WNT10B]
FLJ93354AAAF	656	74.62	[MTHFR]
FLJ93355AAAF	399	45.58	[GPR137B]
FLJ93356AAAF	289	33.16	[STX3]
FLJ93357AAAF	592	67.76	[STXBP3]
FLJ93359AAAF	383	42.63	[CHI3L1]

FLJ93361AAAF	331	37.73	[GDE1]
FLJ93362AAAF	295	34.07	[CCNG1]
FLJ93363AAAF	568	63.38	[MLLT3]
FLJ93364AAAF	731	83.65	[GNL2]
FLJ93365AAAF	443	48.23	[KHDRBS1]
FLJ93367AAAF	476	54.6	[TINAG]
FLJ93369AAAF	472	53.05	[CD36]
FLJ93370AAAF	182	20.65	[CNPY2]
FLJ93371AAAF	463	50.82	[RXRG]
FLJ93372AAAF	207	21.99	[CLDN11]
FLJ93375AAAF	779	88.84	[ZW10]
FLJ93376AAAF	193	21.74	[DDIT4L]
FLJ93377AAAF	353	40.5	[A4GALT]
FLJ93383AAAF	582	65.33	[SEMG2]
FLJ93384AAAF	261	28.74	[KLK3]
FLJ93386AAAF	185	21.08	[SSR3]
FLJ93387AAAF	684	77.13	[TGM4]
FLJ93389AAAF	487	55.12	[MINPP1]
FLJ93390AAAF	744	80.85	[REPS1]
FLJ93391AAAF	548	63.97	[EIF3D]
FLJ93392AAAF	398	45.24	[LIPF]
FLJ93394AAAF	175	20.01	[AGR2]
FLJ93397AAAF	177	20.19	[ITGB3BP]
FLJ93398AAAF	327	37.08	[CD1A]
FLJ93399AAAF	460	52.54	[NAP1L2]
FLJ93400AAAF	412	44.71	[ADORA2A]
FLJ93403AAAF	664	73.5	[SLC5A1]
FLJ93404AAAF	496	53.7	[MATN1]
FLJ93406AAAF	618	70.11	[GRHL1]
FLJ93409AAAF	349	39.35	[IRF2]
FLJ93411AAAF	376	41.88	[ACTG2]
FLJ93412AAAF	270	29.26	[RPA2]
FLJ93413AAAF	557	61.86	[GATB]
FLJ93416AAAF	472	54.46	[NR1H4]
FLJ93417AAAF	431	48.55	[PLAU]
FLJ93419AAAF	302	33.52	[DHRS3]
FLJ93421AAAF	301	32.94	[SLC25A20]
FLJ93422AAAF	467	52.31	[SMAD2]
FLJ93423AAAN	432	48.55	[CCNA2]
FLJ93424AAAF	494	56.52	[CYP2A6]
FLJ93425AAAF	622	70.07	[F2]
FLJ93426AAAF	810	90.54	[PLG]
FLJ93427AAAF	503	57.34	[CYP3A4]
FLJ93428AAAF	478	54.31	[VTN]
FLJ93430AAAF	691	76.45	[SLCO1B1]
FLJ93435AAAF	548	62.03	[GTF2H1]
FLJ93436AAAF	201	22.76	[TMED2]
FLJ93437AAAF	506	56.89	[HARS2]

FLJ93438AAAF	162	18.88	[SNX12]
FLJ93439AAAF	260	29.25	[CA2]
FLJ93441AAAF	248	27.04	[SECTM1]
FLJ93443AAAF	346	37.19	[AGA]
FLJ93444AAAF	437	48.08	[PLIN2]
FLJ93445AAAF	428	44.89	[ELK1]
FLJ93446AAAF	376	42.83	[TMEM183A]
FLJ93448AAAF	622	68.55	[FANCG]
FLJ93449AAAF	677	76.09	[GLB1]
FLJ93451AAAF	296	34.02	[TMX2]
FLJ93452AAAF	423	44.89	[NUPL2]
FLJ93453AAAF	374	41.74	[GPN1]
FLJ93455AAAF	218	24.49	[RAB11B]
FLJ93456AAAF	347	39.47	[DAO]
FLJ93460AAAF	305	34.9	[PEX2]
FLJ93461AAAF	181	20.58	[ATF3]
FLJ93462AAAF	500	55.74	[PLXDC1]
FLJ93464AAAF	203	23.18	[RAB29]
FLJ93465AAAF	328	37.56	[CA10]
FLJ93467AAAF	438	47.64	[CLN3]
FLJ93468AAAF	745	82.36	[ZP2]
FLJ93469AAAF	225	25.49	[SNRPB2]
FLJ93470AAAF	211	24.18	[PITHD1]
FLJ93471AAAF	415	45.47	[NEU1]
FLJ93473AAAF	180	20.51	[ARF4]
FLJ93474AAAF	504	55.31	[ZNF205]
FLJ93476AAAF	246	27.4	[PSMA6]
FLJ93477AAAF	590	67.78	[GRK5]
FLJ93479AAAF	524	57.04	[SLC1A1]
FLJ93480AAAF	137	14.29	[BOLA1]
FLJ93481AAAF	395	45.25	[PIGK]
FLJ93482AAAF	478	54.29	[SCARB2]
FLJ93486AAAF	224	25.5	[TIMP4]
FLJ93487AAAF	296	33.33	[FBXO2]
FLJ93489AAAF	468	53.92	[CHN2]
FLJ93492AAAF	359	39.23	[PDHB]
FLJ93493AAAF	313	36.57	[RRAGA]
FLJ93495AAAF	333	38.39	[EIF2S2]
FLJ93497AAAF	763	83	[HADHA]
FLJ93498AAAF	388	42.79	[CADM4]
FLJ93501AAAF	313	33.85	[SYP]
FLJ93502AAAF	412	45.75	[DNAJA2]
FLJ93503AAAF	303	33.33	[MTCH2]
FLJ93504AAAF	148	17.26	[SSBP1]
FLJ93506AAAF	740	81.39	[DAXX]
FLJ93507AAAF	351	40.32	[ATP6V0D1]
FLJ93508AAAF	200	21.6	[LHFP]
FLJ93510AAAF	320	35.45	[AIMP2]

FLJ93513AAAF	354	40.76	[CLVS1]
FLJ93514AAAF	399	46.38	[CASQ2]
FLJ93516AAAF	436	49.43	[SPOCK3]
FLJ93518AAAF	512	57.74	[ASIC2]
FLJ93519AAAF	481	52.8	[GPR37L1]
FLJ93520AAAF	585	66.15	[MPP3]
FLJ93521AAAF	377	41.24	[PEX14]
FLJ93522AAAF	380	43.97	[ST8SIA3]
FLJ93523AAAF	284	33.08	[SULT4A1]
FLJ93525AAAF	176	18.78	[ADCYAP1]
FLJ93526AAAF	243	26.69	[RPS3]
FLJ93532AAAF	421	49.42	[OMD]
FLJ93534AAAF	482	55.02	[ATE1]
FLJ93535AAAF	380	42.66	[OPRK1]
FLJ93536AAAF	170	19.51	[UBE2G1]
FLJ93537AAAF	298	32.97	[REM1]
FLJ93539AAAF	196	22.45	[CHP2]
FLJ93541AAAF	434	48.16	[SMOC1]
FLJ93543AAAF	271	31.47	[PITPNB]
FLJ93545AAAF	592	64.63	[ATIC]
FLJ93547AAAF	290	33.11	[TSNAX]
FLJ93551AAAF	259	29.46	[SRD5A1]
FLJ93556AAAF	162	17.94	[PLA2G16]
FLJ93557AAAF	621	70.11	[FBXL4]
FLJ93559AAAF	410	46.64	[PAFAH1B1]
FLJ93561AAAF	772	87.23	[TCEB3]
FLJ93562AAAF	543	59.59	[CSRNP2]
FLJ93564AAAF	222	24.75	[SOD2]
FLJ93566AAAF	292	33.87	[RWDD2A]
FLJ93567AAAF	1011	115.52	[RAPGEF4]
FLJ93569AAAF	671	76.02	[SYTL4]
FLJ93570AAAF	369	40.88	[PRPSAP2]
FLJ93573AAAF	480	52.65	[UQCRC1]
FLJ93576AAAF	589	65.68	[ARSE]
FLJ93577AAAF	532	56.96	[SLC18A3]
FLJ93579AAAF	318	36.15	[ADORA3]
FLJ93580AAAF	425	47.79	[TANK]
FLJ93581AAAF	423	48.75	[GAL3ST1]
FLJ93583AAAF	370	42.19	[TPST1]
FLJ93584AAAF	461	49.08	[CREB3L3]
FLJ93585AAAF	519	57.08	[PDZK1]
FLJ93586AAAF	829	89.94	[CDH16]
FLJ93589AAAF	234	26.39	[TREM1]
FLJ93590AAAF	429	46.79	[SMAP2]
FLJ93591AAAF	414	47.78	[TGFB2]
FLJ93593AAAF	265	29.35	[CEACAM7]
FLJ93594AAAF	387	42.79	[HSD17B2]
FLJ93597AAAF	237	26.04	[TSPAN8]

FLJ93598AAAF	272	30.66	[CAPZB]
FLJ93599AAAF	230	26.84	[ADTRP]
FLJ93603AAAF	587	64.63	[ALAS2]
FLJ93605AAAF	288	32.8	[HMOX1]
FLJ93608AAAF	254	28.61	[HLA-DRA]
FLJ93609AAAF	342	39.24	[FUT7]
FLJ93611AAAF	591	64.87	[SLC19A1]
FLJ93613AAAF	758	87.53	[GGCX]
FLJ93614AAAF	372	41.35	[LPAR5]
FLJ93616AAAF	746	86.25	[EXT1]
FLJ93618AAAF	359	41.89	[FUT6]
FLJ93619AAAF	522	58.45	[PRPF4]
FLJ93620AAAF	640	73.17	[CAPN5]
FLJ93621AAAF	1232	139.88	[KIF4A]
FLJ93622AAAF	167	19.2	[EMP2]
FLJ93625AAAF	295	33.73	[CCND1]
FLJ93627AAAF	707	78.37	[MMP9]
FLJ93629AAAF	520	52.66	[MARCO]
FLJ93630AAAF	154	17.44	[RNF11]
FLJ93631AAAF	225	26.13	[BCAS2]
FLJ93632AAAF	633	70.9	[HNRNPR]
FLJ93633AAAF	189	20.68	[FUNDC2]
FLJ93634AAAF	213	24.09	[MOSPD1]
FLJ93638AAAF	487	55.83	[CYB5R4]
FLJ93639AAAF	235	25.32	[TIRAP]
FLJ93640AAAF	645	72.96	[DYNC1I1]
FLJ93641AAAF	317	36.45	[PPP1R3C]
FLJ93644AAAF	413	46.42	[ADRB2]
FLJ93645AAAF	559	61.52	[CHAF1B]
FLJ93646AAAF	540	60.87	[GRB14]
FLJ93647AAAF	263	29.74	[ITM2A]
FLJ93649AAAF	296	32.95	[CAMLG]
FLJ93650AAAF	685	78.84	[SLC3A1]
FLJ93653AAAF	240	25.32	[IGFBP6]
FLJ93654AAAF	415	46.61	[SERPINB2]
FLJ93655AAAF	194	22.51	[FGF7]
FLJ93656AAAF	211	23.35	[SNAP23]
FLJ93657AAAF	407	46.27	[RPL3L]
FLJ93658AAAF	528	56.81	[ALPI]
FLJ93660AAAF	597	67.03	[C4BPA]
FLJ93661AAAF	198	22.17	[SOCS2]
FLJ93662AAAF	630	70.25	[SLC6A4]
FLJ93663AAAF	425	47.03	[SH3BP5]
FLJ93665AAAF	256	27.69	[PRDX3]
FLJ93668AAAF	333	36.58	[GPR146]
FLJ93669AAAF	416	44.32	[LAMP3]
FLJ93671AAAF	354	40.44	[FIGF]
FLJ93673AAAF	126	13.95	[PPP1R11]

FLJ93674AAAF	484	52.51	[BPIFB1]
FLJ93675AAAF	661	74.17	[CD180]
FLJ93677AAAF	404	42.8	[AGER]
FLJ93678AAAF	248	26.23	[SFTPA2]
FLJ93679AAAF	443	50.51	[CPM]
FLJ93680AAAF	217	24.83	[RPL10A]
FLJ93681AAAF	282	31.31	[SNRPA]
FLJ93682AAAF	418	47.37	[PSMC4]
FLJ93683AAAF	642	73.91	[NDC80]
FLJ93684AAAF	359	41.06	[MAB21L1]
FLJ93685AAAF	445	52.26	[EIF3E]
FLJ93686AAAF	542	59.91	[SLC22A8]
FLJ93687AAAF	539	56.33	[SLC39A5]
FLJ93688AAAF	469	53.7	[GBA3]
FLJ93689AAAF	529	60.72	[UGT2B7]
FLJ93690AAAF	482	53.49	[DBT]
FLJ93691AAAF	640	72.66	[SCNN1B]
FLJ93692AAAF	296	34.88	[SULT1C2]
FLJ93693AAAF	513	56.86	[ATP6V1B1]
FLJ93694SAAN	625	70.11	[F11]
FLJ93695AAAF	427	48.53	[SERPINA4]
FLJ93696AAAF	532	60.03	[FMO3]
FLJ93697AAAF	605	66.06	[IGFALS]
FLJ93698AAAF	336	38.61	[CD226]
FLJ93699AAAF	308	34.38	[GTF2H3]
FLJ93700AAAF	365	41.14	[CD244]
FLJ93701AAAF	208	23.07	[HBEGF]
FLJ93702AAAF	170	18.42	[KCNE4]
FLJ93703AAAF	252	27.94	[PNO1]
FLJ93705AAAF	337	37.81	[DNAJB4]
FLJ93707AAAF	847	97.07	[PYGL]
FLJ93708AAAF	373	41.47	[NFE2]
FLJ93709AAAF	333	38.44	[P2RY13]
FLJ93711AAAF	407	45.85	[MNDA]
FLJ93713AAAF	387	44.5	[HCAR3]
FLJ93714AAAF	259	27.49	[TNFRSF10C]
FLJ93715AAAF	295	31.4	[AQP9]
FLJ93716AAAN	524	57.49	[GK]
FLJ93717AAAF	676	77.53	[PRKCD]
FLJ93723AAAF	178	20.02	[DCTD]
FLJ93725AAAN	589	61.56	[TAF15]
FLJ93726AAAF	244	27.34	[RND3]
FLJ93727AAAF	320	35.2	[NPL]
FLJ93728AAAF	270	30.06	[TIGAR]
FLJ93731AAAF	264	29.1	[SNAI1]
FLJ93732AAAF	577	63.48	[IGF2BP1]
FLJ93733AAAN	510	54.99	[CD3EAP]
FLJ93735AAAF	154	16.63	[CYYR1]

FLJ93736AAAF	368	42.03	[POLDIP2]
FLJ93737AAAF	200	22.62	[ARL4A]
FLJ93738AAAF	410	45.98	[TDG]
FLJ93739AAAF	326	36.95	[BMI1]
FLJ93740AAAF	449	50.93	[ZBTB14]
FLJ93741AAAF	343	38.8	[TRIB2]
FLJ93742SAAN	596	66.9	[KLHL41]
FLJ93743AAAF	479	53.18	[RHCG]
FLJ93744AAAF	564	60.03	[KRT6C]
FLJ93745AAAF	745	83.87	[RPS6KA6]
FLJ93746AAAF	356	42.21	[CHST10]
FLJ93747AAAF	700	77.56	[ACOX3]
FLJ93748AAAF	475	50.68	[CABYR]
FLJ93752AAAF	318	34.67	[PRPS2]
FLJ93754AAAF	465	53.4	[TRIM38]
FLJ93756AAAF	390	44.5	[CASQ1]
FLJ93757AAAF	350	40.21	[TGDS]
FLJ93758AAAF	543	61.13	[BTD]
FLJ93759AAAF	364	39.23	[PDLIM3]
FLJ93760AAAF	260	29.56	[CA3]
FLJ93761AAAF	297	33.17	[CNN1]
FLJ93762AAAF	426	48.27	[PSG9]
FLJ93765AAAF	383	41.14	[DLK1]
FLJ93768AAAF	403	45.33	[IDO1]
FLJ93769AAAF	235	26.93	[TFPI2]
FLJ93770AAAF	400	44.09	[KRT19]
FLJ93771AAAF	427	47.86	[DDB2]
FLJ93772AAAF	232	26.67	[VTI1B]
FLJ93774AAAF	399	44.92	[P2RX1]
FLJ93775AAAF	180	19.28	[NBL1]
FLJ93776AAAF	284	30.68	[HOXB13]
FLJ93778AAAF	300	34.31	[CD38]
FLJ93780AAAF	370	41.09	[PRLHR]
FLJ93781AAAF	622	69.51	[PRLR]
FLJ93782AAAF	304	32.61	[HEY1]
FLJ93783AAAF	190	22.83	[APOBEC3C]
FLJ93785AAAF	177	20.31	[TNFSF18]
FLJ93788AAAF	220	23.32	[CLDN3]
FLJ93789AAAF	375	40.05	[CD14]
FLJ93790AAAF	228	26.21	[CACYBP]
FLJ93791AAAF	218	24.71	[SCN1B]
FLJ93792AAAF	345	39.3	[TMOD4]
FLJ93795AAAF	346	40.76	[MRPL38]
FLJ93796AAAF	296	32.94	[MMADHC]
FLJ93797AAAF	339	38.3	[DHRS7]
FLJ93798AAAF	261	28.77	[PCNA]
FLJ93799AAAF	242	26.95	[MYF6]
FLJ93800AAAF	224	25.7	[APOBEC2]

FLJ93801AAAF	194	20.97	[CSRP3]
FLJ93802AAAF	333	35.08	[SUCLG1]
FLJ93805AAAF	419	47.53	[CKMT2]
FLJ93806AAAF	377	42.01	[ACTA2]
FLJ93807AAAF	467	52.9	[MAP3K8]
FLJ93808AAAF	545	62.59	[TRIP10]
FLJ93809AAAF	513	58.52	[KAT5]
FLJ93810AAAF	488	50.79	[HLX]
FLJ93812AAAF	445	50.47	[BCKDHA]
FLJ93815AAAF	293	31.83	[TNFRSF13B]
FLJ93816AAAF	305	35.5	[DNASE1L3]
FLJ93818AAAF	258	29.16	[HLA-DPB1]
FLJ93819AAAF	386	43.33	[LPXN]
FLJ93820AAAF	845	98.19	[VAV1]
FLJ93822AAAF	171	19.21	[BTG1]
FLJ93823AAAF	477	53.98	[MMP3]
FLJ93826AAAF	344	39.51	[CCRL2]
FLJ93828AAAF	220	25.07	[CD28]
FLJ93830AAAF	442	49.77	[CRLF3]
FLJ93831AAAF	197	22.23	[CLEC3A]
FLJ93833AAAF	331	36.52	[DFFA]
FLJ93834AAAF	247	28.35	[PEX11A]
FLJ93837AAAF	140	15.1	[ATG12]
FLJ93838AAAF	315	35.09	[MAGEA9]
FLJ93839AAAF	529	59.11	[ENTPD3]
FLJ93841AAAF	327	35.93	[NIT1]
FLJ93843AAAF	440	49.18	[PSMC1]
FLJ93846AAAF	450	50.85	[LANCL2]
FLJ93847AAAF	444	47.94	[TGFB1I1]
FLJ93849AAAF	402	45.86	[ZNF485]
FLJ93853AAAF	505	55.47	[FAM114A2]
FLJ93856AAAF	500	55.08	[RANBP9]
FLJ93857AAAF	160	17.96	[TMEM191A]
FLJ93858AAAF	631	70.03	[PABPC3]
FLJ93860AAAF	492	53.86	[TMPRSS2]
FLJ93861AAAF	451	51.17	[TUBG1]
FLJ93862AAAF	291	32.96	[MARCH8]
FLJ93864AAAF	516	57.37	[PLAT]
FLJ93865AAAF	428	49.87	[GCNT1]
FLJ93866AAAF	517	57.28	[ALDH1B1]
FLJ93868AAAF	478	52.2	[SLC16A7]
FLJ93871AAAF	319	35.2	[MAGEB2]
FLJ93873AAAF	348	39.08	[CYLC2]
FLJ93874AAAF	968	112.14	[STK10]
FLJ93875AAAF	241	26.41	[PSMA5]
FLJ93876AAAF	1184	132.61	[CILP]
FLJ93878AAAF	605	68.87	[BTRC]
FLJ93879AAAF	698	81.4	[TSGA10]

FLJ93882AAAF	558	61.43	[GLUD2]
FLJ93884AAAF	229	26.26	[CCDC34]
FLJ93886AAAF	396	43.61	[DHH]
FLJ93887AAAF	152	17.02	[OARD1]
FLJ93890AAAF	364	40.75	[DRG2]
FLJ93892AAAF	390	43.06	[ILF2]
FLJ93893AAAF	248	29.23	[RPL7]
FLJ93894AAAF	369	42.02	[CCR9]
FLJ93897AAAF	178	20.93	[GCSAM]
FLJ93898AAAF	357	39.51	[ADPRH]
FLJ93901AAAF	452	50.98	[FLI1]
FLJ93902AAAF	786	87.66	[CCNF]
FLJ93903AAAF	509	54.76	[SLC2A4]
FLJ93906AAAF	360	40.82	[GPR15]
FLJ93908AAAF	335	34.71	[SLC39A11]
FLJ93909AAAF	318	35.05	[PLSCR1]
FLJ93910AAAF	638	71.37	[KLKB1]
FLJ93911AAAF	597	67.72	[PTPN6]
FLJ93913AAAF	454	50.4	[TAT]
FLJ93914AAAF	525	59.51	[HRG]
FLJ93915AAAF	591	67.05	[C8B]
FLJ93916AAAF	452	51.84	[PAH]
FLJ93917AAAF	574	63.92	[SQLE]
FLJ93918AAAF	372	43.01	[USP18]
FLJ93919AAAF	303	33.48	[MEOX2]
FLJ93920AAAF	245	27.63	[CRISP3]
FLJ93921AAAF	517	57.13	[LAD1]
FLJ93923AAAF	633	72.58	[GALNT3]
FLJ93924AAAF	642	72.14	[SLC6A14]
FLJ93925AAAF	584	65.46	[ME2]
FLJ93927AAAF	188	21.4	[MRPL32]
FLJ93928AAAF	690	76.07	[LNX2]
FLJ93930AAAF	95	10.97	[C2orf88]
FLJ93931AAAF	896	98.66	[EPS15]
FLJ93932AAAF	465	52.26	[SMAD5]
FLJ93933AAAF	957	99.4	[COL21A1]
FLJ93934AAAF	897	102.08	[CASK]
FLJ93935AAAF	201	22.28	[TM4SF18]
FLJ93937AAAF	202	22.57	[PQLC3]
FLJ93938AAAF	490	55.71	[CYP2C18]
FLJ93940AAAF	176	20.32	[PRND]
FLJ93942AAAF	271	29.23	[ATF1]
FLJ93943AAAF	153	16.82	[CSDC2]
FLJ93947SAAN	1106	117.96	[GLI1]
FLJ93948AAAF	298	32.87	[C6orf106]
FLJ93949AAAF	302	34.54	[NEK7]
FLJ93950AAAF	957	109.27	[ENPEP]
FLJ93951AAAF	372	42.08	[HSD3B2]

FLJ93953AAAF	285	33.78	[SULT2A1]
FLJ93956AAAF	89	10.11	[PYDC1]
FLJ93958AAAF	197	22.63	[VBP1]
FLJ93959AAAF	317	35.61	[NFKBIA]
FLJ93961AAAF	387	44.71	[BBOX1]
FLJ93962AAAF	317	35.98	[HSD17B6]
FLJ93964AAAF	201	22.72	[RAB9B]
FLJ93965AAAF	402	46.55	[GCNT2]
FLJ93967AAAF	797	87.15	[PCDHB11]
FLJ93968AAAF	301	34.28	[PDCL]
FLJ93970AAAF	459	52.07	[RORB]
FLJ93971AAAF	179	21.83	[NDUFB9]
FLJ93972AAAF	512	57.49	[CYP26B1]
FLJ93973AAAF	493	57.82	[ARIH2]
FLJ93974AAAF	218	24.58	[HPRT1]
FLJ93975AAAF	455	52.93	[EPHX1]
FLJ93976AAAF	209	23.2	[COPS8]
FLJ93977AAAF	437	49.85	[SGCE]
FLJ93978AAAF	316	35.85	[AKR1B1]
FLJ93979AAAF	172	19.47	[NUDT3]
FLJ93985AAAF	225	26.56	[GSTM3]
FLJ93987AAAF	810	89.61	[NELL1]
FLJ93988AAAF	618	69.96	[BIRC2]
FLJ93989AAAF	145	16.36	[UBE2V2]
FLJ93990AAAF	152	17.3	[NME2]
FLJ93995AAAF	796	86.77	[PCDHB3]
FLJ93996AAAF	228	26.26	[THAP2]
FLJ93998AAAF	498	57.9	[ZNF596]
FLJ93999AAAF	417	46.49	[HYAL3]
FLJ94000AAAF	619	70.08	[SLC27A6]
FLJ94001AAAF	209	23.85	[GLTP]
FLJ94002AAAF	149	16.7	[DSCR9]
FLJ94004AAAF	345	38.84	[KLF3]
FLJ94006AAAF	504	55.77	[SLC38A3]
FLJ94007AAAF	606	71.04	[ZNF214]
FLJ94008AAAF	367	41.47	[HS3ST2]
FLJ94009AAAF	579	64.64	[NR1D2]
FLJ94012AAAF	792	86.78	[HCFC2]
FLJ94013AAAF	427	48.76	[IL13RA1]
FLJ94016AAAF	500	56.29	[KCNV1]
FLJ94017AAAF	502	57.02	[CHRNB2]
FLJ94019AAAF	257	28.35	[THAP10]
FLJ94020AAAF	242	27.4	[TRH]
FLJ94021AAAF	283	32.02	[GJB1]
FLJ94022AAAF	211	23.31	[TAF11]
FLJ94023AAAF	175	19.9	[PTHLH]
FLJ94028AAAF	637	72.77	[GCLC]
FLJ94029AAAF	472	54.41	[RGS6]

FLJ94030AAAF	794	87.37	[PCDHB6]
FLJ94032AAAF	254	27.68	[CDC42EP3]
FLJ94035AAAF	414	48.41	[CHST12]
FLJ94039AAAF	391	44.31	[SERPINB13]
FLJ94041AAAF	530	59.73	[UGT1A8]
FLJ94042AAAF	470	50.08	[KLF4]
FLJ94043AAAF	289	32.15	[PNP]
FLJ94044AAAF	326	36.94	[CDK6]
FLJ94045AAAF	456	52.34	[RAPGEFL1]
FLJ94046AAAF	402	44.55	[STAC]
FLJ94047AAAF	854	96.22	[PARP9]
FLJ94049AAAF	886	97.72	[EMR1]
FLJ94050AAAF	328	36.87	[CD84]
FLJ94051AAAF	301	34.09	[STX5]
FLJ94052AAAF	430	47.66	[ACADL]
FLJ94053AAAF	543	60.34	[KCNK10]
FLJ94060AAAF	261	27.45	[EMCN]
FLJ94061AAAN	409	44.21	[RHAG]
FLJ94062AAAF	191	22.22	[CBX5]
FLJ94063AAAF	483	51.75	[ATF7]
FLJ94066AAAF	330	35.4	[PDLIM4]
FLJ94067AAAF	204	21.98	[CSF3]
FLJ94068AAAF	530	59.94	[UGT1A9]
FLJ94069AAAF	602	68.01	[SLC6A13]
FLJ94070AAAF	681	76.83	[ACOX2]
FLJ94071AAAF	216	25.09	[MOB1B]
FLJ94072AAAF	335	36.66	[HOXA1]
FLJ94073AAAF	423	47.68	[SMPD2]
FLJ94074AAAF	404	45.17	[CASP1]
FLJ94075AAAF	398	43.32	[SIRPB1]
FLJ94076AAAF	439	49.2	[PSMC3]
FLJ94077AAAF	456	49.72	[PLA1A]
FLJ94078AAAF	272	31.44	[TIPRL]
FLJ94079AAAF	494	56.9	[CHRNA6]
FLJ94080AAAF	401	44.56	[SLC35B3]
FLJ94081AAAF	260	30.52	[DCK]
FLJ94082AAAF	426	48.38	[IRF8]
FLJ94084AAAF	365	40.03	[CXADR]
FLJ94085AAAF	219	24.44	[MAGEH1]
FLJ94086AAAF	386	41.82	[TNFRSF10D]
FLJ94087AAAF	998	112.1	[EPHA7]
FLJ94090AAAF	538	60.58	[ZKSCAN3]
FLJ94093AAAF	419	47.58	[GPR63]
FLJ94096AAAF	239	25.87	[NKX2-8]
FLJ94098AAAF	219	24.27	[RAB3D]
FLJ94099AAAF	349	39.52	[GPR21]
FLJ94100AAAF	575	61.36	[TFE3]
FLJ94101AAAF	256	28.91	[ATP5F1]

FLJ94103AAAF	129	14.64	[KCNE1]
FLJ94104AAAF	316	36.07	[BRCC3]
FLJ94105AAAF	454	51.27	[GABPA]
FLJ94106AAAF	347	38.79	[TFEC]
FLJ94107AAAF	327	37.28	[C3orf38]
FLJ94108AAAF	339	39.72	[TRMT10A]
FLJ94110AAAF	374	40.35	[P2RY11]
FLJ94111AAAF	250	27.6	[HLA-DOA]
FLJ94113AAAF	313	34.65	[UNG]
FLJ94114AAAF	167	18.62	[LEP]
FLJ94116AAAF	258	28.53	[MPZ]
FLJ94117AAAF	901	103.25	[ACTN3]
FLJ94118AAAF	359	40.62	[HEXIM1]
FLJ94121AAAF	1162	134.06	[STAG2]
FLJ94122AAAF	321	35.89	[TSTA3]
FLJ94125AAAF	304	35	[TFPI]
FLJ94126AAAF	445	49.97	[HGD]
FLJ94128AAAF	412	47.33	[TGFB3]
FLJ94130AAAF	1228	138.11	[MMRN1]
FLJ94133AAAF	226	24.58	[TMEM98]
FLJ94134AAAF	354	39.18	[WNT11]
FLJ94136AAAF	623	69.6	[SYNCRIP]
FLJ94137AAAF	256	27.44	[POU2AF1]
FLJ94139AAAF	361	42.16	[FUT3]
FLJ94140AAAF	479	57.67	[ZRSR1]
FLJ94141AAAF	557	61.19	[CD19]
FLJ94142AAAF	393	44.7	[CRTAM]
FLJ94144AAAF	150	17.2	[RNASE6]
FLJ94145AAAF	403	47.17	[PTEN]
FLJ94146AAAF	219	24.34	[CD53]
FLJ94147AAAF	229	26.03	[CD79B]
FLJ94149AAAF	404	45.78	[CD209]
FLJ94150AAAF	330	37.51	[PPP1CA]
FLJ94152AAAF	961	105.9	[THBS4]
FLJ94153AAAF	384	43.2	[GRPR]
FLJ94157AAAF	373	41.12	[MAGEC2]
FLJ94161AAAF	373	39.47	[DMRT1]
FLJ94162AAAF	226	26.23	[GJB2]
FLJ94165AAAF	435	50.28	[ORC5]
FLJ94167AAAF	508	59.91	[CCDC181]
FLJ94168AAAF	588	67.23	[NME8]
FLJ94169AAAF	388	41.95	[SLC30A3]
FLJ94170AAAF	222	25.66	[GSTA2]
FLJ94171AAAF	301	32.79	[SLC25A15]
FLJ94173AAAF	387	42.61	[EGR3]
FLJ94174AAAF	345	37.31	[HSDL2]
FLJ94176AAAF	280	31.96	[XRCC2]
FLJ94179AAAF	430	49.66	[TEKT2]

FLJ94184AAAF	351	39.45	[CD2]
FLJ94186AAAF	576	62.61	[COIL]
FLJ94187AAAF	185	18.95	[CD99]
FLJ94188AAAF	796	91.82	[TLR6]
FLJ94189AAAF	234	26.02	[TNFSF8]
FLJ94190AAAF	560	62.77	[CDC6]
FLJ94192AAAF	524	57.49	[SLC2A2]
FLJ94193AAAF	534	60.01	[UGT1A4]
FLJ94195AAAF	481	53.38	[LBP]
FLJ94197AAAF	341	36.28	[PAX9]
FLJ94198AAAF	417	48.71	[CPA3]
FLJ94200AAAF	690	79.01	[CAPN9]
FLJ94202AAAF	244	27.23	[KLF9]
FLJ94205AAAF	150	16.41	[CEBPG]
FLJ94206AAAF	371	41.41	[CREB3]
FLJ94208AAAF	1445	161.61	[CD109]
FLJ94211AAAF	376	41.63	[SLC30A7]
FLJ94213AAAF	1482	163.87	[PZP]
FLJ94214AAAF	795	87.23	[PCDHB4]
FLJ94216AAAF	399	45.78	[MTERF1]
FLJ94217AAAF	502	56.93	[BMPR1B]
FLJ94219AAAF	337	38.54	[CYSLTR1]
FLJ94220AAAF	597	66.46	[ESR1]
FLJ94221AAAF	186	20.89	[TBPL1]
FLJ94225AAAF	462	51.7	[HPX]
FLJ94227AAAF	265	29.67	[COQ4]
FLJ94229AAAF	558	60.25	[HNRNPL]
FLJ94230AAAF	289	32.24	[TXNL1]
FLJ94232AAAF	427	45.18	[NGFR]
FLJ94233AAAF	612	71.41	[ZNF189]
FLJ94236AAAF	118	13.33	[EIF4E3]
FLJ94237AAAF	484	53.37	[CTSF]
FLJ94241AAAF	411	47.66	[CRHR2]
FLJ94243AAAF	196	21.68	[SPRYD7]
FLJ94245AAAF	531	61.05	[MGAT3]
FLJ94247AAAF	230	26.18	[UCHL3]
FLJ94249AAAF	301	32.44	[ANKRD29]
FLJ94250AAAF	211	24.38	[RGS2]
FLJ94252AAAF	405	45.68	[RUNDC3A]
FLJ94253AAAF	389	44.17	[PSMC6]
FLJ94257AAAF	377	44.05	[GNA13]
FLJ94260AAAF	351	37.22	[ROM1]
FLJ94263AAAF	295	34.29	[STAP1]
FLJ94264AAAF	711	80.54	[ALOXE3]
FLJ94265AAAF	564	64.87	[NOX1]
FLJ94266AAAF	368	40.67	[CXCR3]
FLJ94267AAAF	241	27.54	[GSTO1]
FLJ94268AAAF	219	21.89	[HIST1H1E]

FLJ94274AAAF	229	25.72	[RAB33B]
FLJ94275AAAF	648	72.78	[DDX43]
FLJ94276AAAF	381	43.66	[KRR1]
FLJ94278AAAF	159	17.12	[GADD45G]
FLJ94280AAAF	614	69.42	[SLC6A12]
FLJ94281AAAF	551	60.59	[CBS]
FLJ94282AAAF	237	27.19	[FAM133B]
FLJ94285AAAF	604	67.11	[ME3]
FLJ94287AAAF	475	51.84	[RRP9]
FLJ94288SAAF	345	38.81	[BAG1]
FLJ94289AAAF	339	40.24	[FAM50A]
FLJ94291AAAF	250	27.88	[TMEM106C]
FLJ94293AAAF	133	14.62	[NHLH1]
FLJ94296AAAF	188	21.52	[CLEC5A]
FLJ94297AAAF	276	30.61	[NIT2]
FLJ94299AAAF	389	42.85	[SH2D2A]
FLJ94300AAAF	216	24.64	[F8]
FLJ94301AAAF	487	53.48	[SLC7A9]
FLJ94302AAAF	827	92.67	[VIL1]
FLJ94306AAAF	354	40.17	[HAPLN1]
FLJ94307AAAF	371	39.39	[HPDL]
FLJ94309AAAF	346	40.02	[ANGPTL7]
FLJ94310AAAF	195	21.45	[EXOSC1]
FLJ94314AAAF	271	29.35	[FOSL1]
FLJ94315AAAF	389	44.88	[RRM2]
FLJ94316AAAF	525	56.17	[SLC18A1]
FLJ94321AAAF	120	13.01	[EIF4EBP2]
FLJ94324AAAF	273	30.43	[CHODL]
FLJ94325AAAF	461	54.46	[PIK3R3]
FLJ94328AAAF	399	46.29	[CYTH3]
FLJ94330AAAF	281	32.2	[NSL1]
FLJ94332AAAF	223	24.63	[CTLA4]
FLJ94333AAAF	251	28.79	[ANP32B]
FLJ94334AAAF	313	34.95	[GAS2]
FLJ94336AAAF	140	16.44	[TRAPPC2]
FLJ94337AAAF	347	35.88	[JUNB]
FLJ94339AAAF	230	24.67	[LYPLA1]
FLJ94340AAAF	360	40.15	[WARS2]
FLJ94341AAAF	481	54.3	[HTR2B]
FLJ94342AAAN	406	44.61	[TFDP1]
FLJ94344AAAF	161	18.4	[TNNC1]
FLJ94345AAAF	191	21.33	[RHOH]
FLJ94347AAAF	354	38.56	[KTI12]
FLJ94348AAAF	494	53.51	[KRT12]
FLJ94349AAAF	449	47.97	[PCOLCE]
FLJ94350AAAF	449	49.33	[TFAP2B]
FLJ94351AAAF	400	40.32	[SPN]
FLJ94355AAAF	342	39.28	[CXCR6]

FLJ94356AAAF	476	55.43	[ZNF563]
FLJ94357AAAF	350	40.12	[PLEK]
FLJ94358AAAF	457	50.72	[CHGA]
FLJ94359AAAF	194	21.63	[AK1]
FLJ94360AAAF	342	38.07	[HOXC10]
FLJ94361AAAF	405	45.09	[SERPINA6]
FLJ94363AAAF	808	87.25	[TAP1]
FLJ94364AAAF	588	66.58	[CCIN]
FLJ94365AAAF	755	86.69	[SENP5]
FLJ94370AAAF	301	34.73	[CPXCR1]
FLJ94371AAAF	346	38.92	[MAGEB4]
FLJ94372AAAF	507	57.03	[XPNPEP3]
FLJ94375AAAF	647	70.86	[LRWD1]
FLJ94378AAAF	246	28.46	[BRMS1]
FLJ94379AAAF	241	26.3	[TSPAN1]
FLJ94382AAAF	324	35.92	[FNDC8]
FLJ94383AAAF	166	19.21	[FAM9C]
FLJ94384AAAF	226	26.53	[TXNDC9]
FLJ94385AAAF	600	65.39	[MED26]
FLJ94386AAAF	497	55.4	[SLC19A2]
FLJ94387AAAF	789	86.62	[ARNT]
FLJ94390AAAF	448	48.59	[EDAR]
FLJ94391AAAF	374	42.75	[SERPINB8]
FLJ94392AAAF	257	28.08	[TMEM55A]
FLJ94393AAAF	260	30.07	[NSA2]
FLJ94394AAAF	472	49.15	[FOXA1]
FLJ94397AAAF	260	29.66	[UPK1B]
FLJ94398AAAF	610	66.6	[SLC5A8]
FLJ94401AAAF	1089	122.68	[PDGFRA]
FLJ94402AAAF	706	79.3	[FZD6]
FLJ94403AAAF	175	20.08	[ARF6]
FLJ94404AAAF	736	81.96	[BACH1]
FLJ94408AAAF	118	13.39	[TMEM243]
FLJ94410AAAF	237	26.69	[RAB23]
FLJ94415AAAN	488	54.73	[F10]
FLJ94416AAAF	356	39.89	[NEUROD1]
FLJ94420AAAF	264	30.24	[NDUFS3]
FLJ94421AAAF	286	31.45	[NAT6]
FLJ94422AAAF	292	33.3	[HNMT]
FLJ94423AAAF	153	17.8	[MRPL23]
FLJ94425AAAF	218	24.39	[RAB4A]
FLJ94427AAAF	801	89.03	[CDH20]
FLJ94428AAAF	315	34.72	[SPRY2]
FLJ94429AAAF	349	39.1	[ABHD5]
FLJ94431AAAF	502	57	[CYP3A5]
FLJ94434AAAF	392	44.07	[PAFAH2]
FLJ94435AAAF	259	29.89	[RRP36]
FLJ94436AAAF	345	39.04	[PDGFC]

FLJ94439AAAF	551	62.09	[EIF2AK2]
FLJ94440AAAF	531	57.97	[CCT6A]
FLJ94441AAAN	446	45.21	[SOX3]
FLJ94442AAAF	183	20.14	[SSR2]
FLJ94443AAAF	189	21.65	[NDUFB5]
FLJ94444AAAF	217	24.67	[RIT2]
FLJ94445AAAF	338	38.27	[AHSA1]
FLJ94446AAAF	264	29.19	[PSMB4]
FLJ94449AAAF	205	23.51	[MAD2L1]
FLJ94454AAAF	389	42.77	[OXTR]
FLJ94455AAAF	685	74.62	[DLL4]
FLJ94458AAAF	317	36.88	[RCN2]
FLJ94459AAAF	366	41.27	[SMS]
FLJ94460AAAF	222	25.24	[TMEM27]
FLJ94461AAAF	673	74.22	[FXR2]
FLJ94463AAAF	345	38.33	[APOH]
FLJ94465AAAF	370	40.92	[HAO1]
FLJ94467AAAF	271	28.68	[COLEC11]
FLJ94468AAAF	279	32.19	[FHL2]
FLJ94469AAAF	737	83.68	[PRKCE]
FLJ94474AAAF	414	47.91	[STK32B]
FLJ94475AAAF	272	30.85	[IL2RA]
FLJ94476AAAF	288	31.65	[PDCD1]
FLJ94478AAAF	258	28.66	[CISH]
FLJ94479AAAF	336	37.54	[LGALS12]
FLJ94481AAAF	222	25.03	[CACNG1]
FLJ94482AAAF	250	26.83	[WISP2]
FLJ94483AAAF	242	28.16	[MRPS7]
FLJ94486AAAF	264	29.91	[MYOZ2]
FLJ94487AAAF	408	43.52	[ADRB3]
FLJ94489AAAF	1049	120.92	[TLR7]
FLJ94490AAAF	422	47.5	[TMPRSS11E]
FLJ94492AAAF	241	26.94	[IGSF6]
FLJ94493AAAF	282	31.05	[SIAH1]
FLJ94497AAAF	787	88.02	[ADAM32]
FLJ94500AAAF	461	52.29	[C9orf43]
FLJ94501AAAF	433	49.76	[SMYD2]
FLJ94503AAAF	449	52.39	[CLU]
FLJ94504AAAF	415	45.23	[ACTL7B]
FLJ94505AAAF	388	42.93	[PDHA2]
FLJ94506AAAF	251	27.17	[MYOZ3]
FLJ94512AAAF	648	68.93	[FOXN1]
FLJ94514AAAF	512	58.17	[CYP1A1]
FLJ94515AAAF	199	22.61	[RERG]
FLJ94516AAAF	895	99.84	[CTNNA3]
FLJ94517AAAF	497	56.62	[XIAP]
FLJ94520AAAF	255	27.9	[HOXD4]
FLJ94522AAAF	167	17.64	[FAM163A]

FLJ94525AAAF	450	52.76	[BFAR]
FLJ94526AAAF	215	24.33	[SCN2B]
FLJ94527AAAF	225	26.19	[KCTD14]
FLJ94530AAAF	229	24.55	[SYNGR3]
FLJ94532AAAF	478	55.24	[HTR3A]
FLJ94533AAAF	477	52.95	[DRD5]
FLJ94535AAAF	419	47.48	[MAP2K7]
FLJ94538AAAF	400	44.8	[OPRM1]
FLJ94539AAAF	381	44.31	[GNAL]
FLJ94541AAAF	345	38.15	[TOB1]
FLJ94543AAAF	422	47.55	[STYK1]
FLJ94544AAAF	193	22.43	[HPCA]
FLJ94545AAAF	472	52.86	[CNR1]
FLJ94547AAAF	372	42.15	[METTL18]
FLJ94548AAAF	182	19.99	[PLLP]
FLJ94550AAAF	272	28.23	[RFXAP]
FLJ94551AAAF	243	26.19	[TMEM109]
FLJ94553AAAF	128	15.01	[C3orf14]
FLJ94554AAAF	149	17.31	[CLEC2B]
FLJ94557AAAF	459	51.83	[FKBP4]
FLJ94559AAAF	373	42.06	[P2RY1]
FLJ94560AAAF	313	34.46	[HOXA11]
FLJ94561AAAF	160	17.87	[GPSM3]
FLJ94562AAAF	350	39.91	[ACKR4]
FLJ94563AAAF	375	42.43	[ST8SIA2]
FLJ94564AAAF	233	25.87	[MARCH9]
FLJ94565AAAF	543	60.33	[ASIC3]
FLJ94566AAAF	363	41.18	[AGTR2]
FLJ94568AAAF	370	40.76	[PPID]
FLJ94572AAAF	740	83.74	[RPS6KA3]
FLJ94573AAAF	740	82.43	[DDX1]
FLJ94574AAAF	149	16.89	[CALML3]
FLJ94575AAAF	240	25.9	[SOD3]
FLJ94576AAAF	248	28.2	[TIMM21]
FLJ94577AAAF	601	66.65	[DYRK2]
FLJ94581AAAF	437	47.51	[E2F2]
FLJ94583AAAF	465	51.16	[PNLIP]
FLJ94584AAAF	377	42.24	[P2RY2]
FLJ94585AAAF	318	35.16	[MAGEA8]
FLJ94587AAAF	323	36.38	[ANXA3]
FLJ94588AAAF	163	18.57	[CD247]
FLJ94589AAAF	280	31.59	[GEMIN2]
FLJ94590AAAF	299	33.61	[CFDP1]
FLJ94591AAAF	262	29.75	[PMM1]
FLJ94593AAAF	367	40.99	[GPR17]
FLJ94596AAAF	419	46.37	[FAH]
FLJ94597AAAF	214	23.08	[NEUROG3]
FLJ94599AAAF	372	41.85	[GMDS]

FLJ94601AAAF	346	39.3	[HCAR1]
FLJ94602AAAF	195	21.67	[PGRMC1]
FLJ94603AAAF	364	41.69	[PARVB]
FLJ94604AAAF	211	22.39	[CLDN7]
FLJ94605AAAF	470	54	[MMP12]
FLJ94606AAAF	476	54.15	[MMP10]
FLJ94607AAAF	739	82.86	[ADAM18]
FLJ94609AAAF	558	66.39	[NSRP1]
FLJ94613AAAF	182	20.47	[CD3G]
FLJ94614AAAF	541	62.3	[IL18R1]
FLJ94615AAAF	255	29.48	[SKA1]
FLJ94617AAAF	191	21.85	[ANKRD22]
FLJ94618AAAF	462	51.47	[NFIL3]
FLJ94619AAAF	574	62.98	[IL22RA1]
FLJ94620AAAF	458	52.29	[CPN1]
FLJ94621AAAF	292	32.4	[HSD11B1]
FLJ94625AAAF	204	23.4	[RRAS2]
FLJ94626AAAF	210	23.77	[EMC8]
FLJ94628AAAF	428	47.84	[CCKAR]
FLJ94629AAAF	191	21.8	[KCNMB1]
FLJ94630AAAF	288	33.02	[STX1A]
FLJ94632AAAF	357	40.48	[G6PC]
FLJ94635AAAF	118	13.29	[BET1]
FLJ94636AAAF	239	27.61	[PDCL3]
FLJ94638AAAN	644	66.02	[KRT1]
FLJ94641AAAF	147	16.55	[ANG]
FLJ94642AAAF	663	75.74	[CTCFL]
FLJ94643AAAF	917	104.36	[NSMAF]
FLJ94644AAAF	242	27.68	[CASP14]
FLJ94646AAAF	337	36.08	[C5AR2]
FLJ94649AAAF	474	55.34	[CALCR]
FLJ94650AAAF	463	52.98	[GLP1R]
FLJ94651AAAF	423	48.45	[KCNJ6]
FLJ94652AAAF	248	28.15	[MLF2]
FLJ94653AAAF	254	26.64	[TNFSF9]
FLJ94654AAAF	280	32.74	[ING2]
FLJ94655AAAF	265	30.76	[CLEC12A]
FLJ94657AAAF	329	37.01	[DNTTIP1]
FLJ94659AAAF	337	37.81	[IFNGR2]
FLJ94661AAAF	430	49.46	[SPZ1]
FLJ94662AAAF	261	30.51	[SRSF12]
FLJ94663AAAF	523	58.3	[KCNA3]
FLJ94665AAAF	368	41.09	[ANKRD40]
FLJ94666AAAF	552	62.33	[PRKAA2]
FLJ94669AAAF	238	26.74	[SMNDC1]
FLJ94671AAAF	241	26.92	[CLIC1]
FLJ94675AAAF	296	33.95	[GEM]
FLJ94677AAAF	360	40.53	[KCNJ13]

FLJ94678AAAF	457	49.6	[BAG4]
FLJ94679AAAF	277	29.47	[TMEM55B]
FLJ94680AAAF	400	44.51	[GFRA3]
FLJ94683AAAF	325	36.5	[IRF1]
FLJ94686AAAF	569	64.39	[CFHR5]
FLJ94687AAAN	355	39.75	[PON1]
FLJ94688AAAF	349	38.09	[SLC10A1]
FLJ94689AAAF	175	20.16	[CRYAB]
FLJ94690AAAF	600	64.79	[KRT84]
FLJ94691AAAF	446	49.29	[DRD1]
FLJ94692AAAF	815	92.15	[RBM5]
FLJ94694AAAF	583	65.49	[STS]
FLJ94695AAAF	246	26.26	[TMUB1]
FLJ94698AAAF	145	16.86	[TRAPPC1]
FLJ94699AAAF	691	76.89	[SLC28A3]
FLJ94700AAAF	596	67.74	[IRAK3]
FLJ94701AAAF	713	77.74	[STRN3]
FLJ94702AAAF	326	34	[ZFP36]
FLJ94704AAAF	201	22.61	[TAGLN]
FLJ94709AAAF	153	17.36	[ORMDL2]
FLJ94710AAAF	653	72.72	[LRRC4]
FLJ94713AAAF	365	42.28	[SGMS2]
FLJ94715AAAF	498	56.62	[FSCN3]
FLJ94716AAAF	320	35.49	[OR51E2]
FLJ94717AAAF	947	108.24	[MYCBPAP]
FLJ94719AAAF	702	80.57	[CAPN11]
FLJ94720AAAF	519	57.99	[TCP11L2]
FLJ94721AAAF	356	39.21	[GPANK1]
FLJ94723AAAF	169	19.17	[DDIT3]
FLJ94725AAAF	180	19.18	[MCAT]
FLJ94729AAAF	543	60.83	[YES1]
FLJ94734AAAF	196	21.78	[CHMP1B]
FLJ94737AAAF	163	19.17	[CEP19]
FLJ94739AAAF	519	57.06	[CREB3L1]
FLJ94743AAAF	678	75.43	[OVGP1]
FLJ94745AAAN	325	34.98	[FOXB1]
FLJ94747AAAF	564	62.31	[COASY]
FLJ94749AAAF	217	24.19	[DUSP19]
FLJ94750AAAF	412	46.42	[SLC35G2]
FLJ94754AAAF	427	48.27	[KCNJ2]
FLJ94756AAAF	169	19.01	[MYLPF]
FLJ94759AAAF	517	57.91	[SNTG1]
FLJ94761AAAF	317	34.78	[THOC6]
FLJ94763AAAF	404	46	[KRT33A]
FLJ94765AAAF	471	54.68	[PFKFB1]
FLJ94771AAAF	797	90.4	[RASGRP1]
FLJ94772AAAF	151	16.38	[LECT2]
FLJ94773AAAF	1148	128.93	[SCAF11]

FLJ94778AAAF	142	15.33	[NRN1]
FLJ94780AAAF	347	40.16	[GBGT1]
FLJ94785AAAF	214	22.96	[N6AMT1]
FLJ94788AAAF	291	33.87	[POPDC3]
FLJ94792AAAF	96	11.31	[COA4]
FLJ94793AAAF	358	38.99	[WDR53]
FLJ94794AAAF	110	12.07	[PTMA]
FLJ94795AAAF	192	21.54	[SEC11C]
FLJ94797AAAF	244	28	[ULBP1]
FLJ94799AAAF	286	32.69	[NT5C3A]
FLJ94801AAAF	95	10.43	[DEXI]
FLJ94802AAAF	282	31.14	[ACBD6]
FLJ94803AAAF	420	45.42	[CRELD1]
FLJ94805AAAF	218	24.61	[RAB27B]
FLJ94806AAAF	422	49.21	[B3GALT2]
FLJ94809AAAF	200	22.93	[CNTF]
FLJ94810AAAF	192	21.49	[ARL4C]
FLJ94811AAAF	328	34.09	[HOXD1]
FLJ94816AAAF	334	37.64	[HSF2BP]
FLJ94819AAAF	133	14.86	[FABP3]
FLJ94821AAAF	513	57.85	[ACVR2A]
FLJ94822AAAF	656	74.07	[CHML]
FLJ94824AAAF	240	26.49	[SOX14]
FLJ94825AAAF	190	20.75	[DCTN6]
FLJ94826AAAF	200	23.13	[RCVRN]
FLJ94827AAAF	332	36.94	[MC4R]
FLJ94828AAAF	155	17.77	[C10orf111]
FLJ94830AAAF	471	52.6	[HTR2A]
FLJ94832AAAF	370	41.9	[LPAR4]
FLJ94833AAAF	172	20.11	[NDUFA8]
FLJ94834AAAF	331	37.04	[NEUROD4]
FLJ94837AAAF	206	23.63	[ICT1]
FLJ94840AAAF	140	14.67	[LY6H]
FLJ94841AAAF	313	35.72	[TYMS]
FLJ94843AAAF	323	35.69	[CLEC11A]
FLJ94847AAAF	207	22.91	[UBE2E3]
FLJ94848AAAF	179	20.63	[SEC11A]
FLJ94853AAAF	158	17.71	[IL36A]
FLJ94856AAAF	540	59.4	[ZP4]
FLJ94859AAAF	56	6.11	[ARHGAP5-AS1]
FLJ94864AAAF	166	18.74	[CFL2]
FLJ94867AAAF	604	67.13	[CBFA2T2]
FLJ94869AAAF	271	30.48	[ZCCHC9]
FLJ94870AAAF	369	40.71	[MAGEA10]
FLJ94872AAAF	156	16.93	[IER3]
FLJ94873AAAF	160	19	[MPHOSPH6]
FLJ94874AAAF	315	34.34	[WDR83]
FLJ94877AAAF	351	38.23	[TMEM115]

FLJ94879AAAF	354	38.03	[FOXF1]
FLJ94880AAAF	314	34.84	[MAGEA12]
FLJ94883AAAF	912	101.7	[PRKD1]
FLJ94886AAAF	178	21.01	[CRYGS]
FLJ94888AAAF	855	94.75	[ST14]
FLJ94895AAAF	671	75.82	[RIPK1]
FLJ94897AAAF	134	15.39	[CPLX2]
FLJ94902AAAF	685	79.38	[RNF103]
FLJ94903AAAF	680	77.31	[GNPAT]
FLJ94904AAAF	557	61.99	[CPNE6]
FLJ94906AAAF	862	99.27	[RABEP1]
FLJ94907AAAF	919	102.45	[PWP2]
FLJ94908WAAF	1581	168.32	[MED1]
FLJ94911AAAF	883	98.74	[GRIA2]
FLJ94912AAAF	441	49.11	[SGPP1]
FLJ94913AAAF	726	80.79	[HLCS]
FLJ94915AAAF	815	90.79	[SLC9A1]
FLJ94916AAAF	1044	119.51	[PIK3CD]
FLJ94917AAAF	791	89.08	[DGKG]
FLJ94919AAAF	1030	115.33	[PPP1R12A]
FLJ94921AAAF	710	80.73	[PREP]
FLJ94922AAAF	1029	118.85	[ZNF197]
FLJ94924AAAF	911	101.39	[ITIH1]
FLJ94925AAAF	444	51.96	[FADS1]
FLJ94926AAAF	499	57.07	[SERPIND1]
FLJ94930AAAN	745	84.64	[CHUK]
FLJ94931AAAF	794	88.92	[ZNF148]
FLJ94932AAAF	557	60.94	[PXN]
FLJ94933AAAF	509	57.28	[CYP51A1]
FLJ94934AAAF	536	60.35	[KPNA5]
FLJ94935AAAF	617	71.33	[ZNF221]
FLJ94938AAAF	904	103.86	[TLR3]
FLJ94941AAAF	919	101.74	[LIG1]
FLJ94942AAAN	464	50.52	[CEACAM1]
FLJ94944AAAF	225	24.77	[SOCS3]
FLJ94946AAAF	1156	126.3	[CILP2]
FLJ94947AAAF	610	66.63	[SELE]
FLJ94950AAAF	150	16.97	[HSPB3]
FLJ94953AAAF	524	59.86	[JRKL]
FLJ94954AAAF	325	37.25	[LIMS1]
FLJ94955AAAF	480	52.54	[DNAJA3]
FLJ94957AAAF	1358	153.19	[PIK3R4]
FLJ94959AAAF	561	58.82	[EFS]
FLJ94960AAAF	574	63.17	[LOXL1]
FLJ94962AAAF	915	104.6	[FAM13B]
FLJ94964AAAF	356	37.5	[PCBP1]
FLJ94965AAAF	1025	117.35	[LNPEP]
FLJ94967AAAF	383	42.55	[MICB]

FLJ94968AAAF	525	56.59	[NAB2]
FLJ94973AAAF	738	85.46	[ZNF84]
FLJ94975AAAF	753	80.96	[MTF1]
FLJ94976AAAF	723	78.06	[DLL1]
FLJ94979AAAF	1088	120.14	[LATS2]
FLJ94981AAAF	980	104.06	[PEX6]
FLJ94984AAAF	219	25.14	[RIT1]
FLJ94986AAAF	432	47.49	[ACADSB]
FLJ94987SAAN	495	56.3	[NUFIP1]
FLJ94988AAAF	1150	130.25	[PPP2R3A]
FLJ94989AAAF	335	37.2	[SLAMF1]
FLJ94990AAAF	461	53.3	[CDS1]
FLJ94991AAAF	1170	128.87	[ITGAL]
FLJ94992SAAF	477	54.01	[GCGR]
FLJ94993AAAF	282	31.29	[NPTN]
FLJ94995AAAF	677	75.85	[GPSM2]
FLJ94996AAAF	384	41.81	[DUSP9]
FLJ94998AAAF	747	86.43	[AMPD1]
FLJ94999AAAF	813	92.62	[CAPN7]
FLJ95000AAAF	999	110.18	[MERTK]
FLJ95001AAAF	740	83.23	[ABCD2]
FLJ95002AAAF	1167	127.6	[ITGA10]
FLJ95003AAAF	480	55.72	[AKT1]
FLJ95005AAAF	1056	119.13	[KIF11]
FLJ95006AAAF	658	72.91	[AGPS]
FLJ95007AAAF	777	86.58	[BARD1]
FLJ95010AAAF	1417	158.96	[BLM]
FLJ95012AAAF	508	56.91	[UGP2]
FLJ95014AAAF	513	57	[VNN1]
FLJ95021AAAF	441	50.29	[HTR3B]
FLJ95022AAAF	553	63.02	[GLP2R]
FLJ95024AAAF	289	31.64	[OTX2]
FLJ95025AAAF	450	49.14	[TFAP2C]
FLJ95028AAAF	607	69.52	[MMP16]
FLJ95030AAAF	193	21.1	[CBLN1]
FLJ95031AAAF	333	38.49	[XCR1]
FLJ95032AAAF	548	58.7	[ERF]
FLJ95033AAAF	444	50.68	[HCRTR2]
FLJ95034AAAF	237	25.65	[NEUROG1]
FLJ95037AAAF	577	65.26	[ACSM1]
FLJ95039AAAF	626	69.67	[PEX5L]
FLJ95040AAAF	956	107.23	[GRIK4]
FLJ95041AAAF	551	64.32	[TPTE]
FLJ95042AAAF	108	11.3	[TP53AIP1]
FLJ95045AAAF	962	107.92	[USO1]
FLJ95047AAAF	707	81.04	[EZH2]
FLJ95049AAAF	789	90.88	[TAX1BP1]
FLJ95052AAAF	589	68.05	[POF1B]

FLJ95053AAAF	1020	112.31	[ATP1A2]
FLJ95054AAAF	292	31.94	[MTFR1L]
FLJ95055AAAF	499	55.1	[KCNK5]
FLJ95057AAAF	377	44.22	[CCDC113]
FLJ95058AAAF	773	88.34	[CPT1A]
FLJ95061AAAF	565	61.91	[BIN2]
FLJ95063AAAF	741	84.16	[MFN1]
FLJ95064AAAF	412	46.64	[NOB1]
FLJ95066AAAF	335	38.04	[MAGT1]
FLJ95067AAAF	941	106.91	[PRPF6]
FLJ95073AAAF	1002	111.65	[CLMN]
FLJ95076AAAF	616	64.44	[CSTF2T]
FLJ95077AAAF	581	66.15	[TRIP4]
FLJ95081AAAF	399	44.74	[SGPP2]
FLJ95083AAAF	372	42.53	[B3GNT3]
FLJ95084AAAF	395	45.27	[CHKB]
FLJ95085AAAF	591	67.15	[GBP2]
FLJ95090AAAF	622	67.81	[TAF6L]
FLJ95099AAAF	172	19.22	[VOPP1]
FLJ95100AAAF	922	104.03	[URGCP]
FLJ95101AAAF	379	43.35	[GALT]
FLJ95103AAAF	919	100.58	[ATP2C1]
FLJ95109AAAF	766	84.44	[BRAF]
FLJ95115AAAF	511	55.99	[SLC7A7]
FLJ95117AAAF	243	26.56	[SSPN]
FLJ95126AAAF	1230	136.38	[CAND1]
FLJ95128AAAF	595	66.56	[SNX9]
FLJ95130AAAF	819	92.03	[AVIL]
FLJ95131AAAF	699	73.6	[NOLC1]
FLJ95133AAAF	504	54.54	[FGFRL1]
FLJ95135AAAF	791	83.29	[KANSL3]
FLJ95136AAAF	532	59.65	[GRB7]
FLJ95137AAAF	302	33.35	[ICOSLG]
FLJ95140AAAF	1052	118	[UBA6]
FLJ95145AAAF	195	21.47	[NRSN1]
FLJ95147AAAF	914	100.2	[CLCA1]
FLJ95149AAAF	765	84.28	[SNRK]
FLJ95150AAAF	896	97	[PCDH10]
FLJ95151AAAF	298	33.3	[MLX]
FLJ95153AAAF	177	19.46	[CNBP]
FLJ95154AAAF	770	82.47	[DAB2]
FLJ95156AAAF	392	42.81	[HS1BP3]
FLJ95159AAAF	536	58.4	[XYLB]
FLJ95160AAAF	828	94.07	[COG3]
FLJ95163AAAF	390	43.48	[PTRF]
FLJ95164AAAF	403	46.1	[SEC14L2]
FLJ95166AAAF	248	27.06	[APOLD1]
FLJ95169AAAF	677	77.34	[FERMT1]

FLJ95171AAAF	727	80.36	[ZFAND4]
FLJ95172AAAF	698	76.65	[CNTN4]
FLJ95174AAAF	348	38.28	[GALE]
FLJ95176AAAF	521	57.71	[UTP18]
FLJ95177AAAF	709	79.78	[FBXO40]
FLJ95178AAAF	975	113.66	[RNF20]
FLJ95179AAAF	496	54.55	[DFNA5]
FLJ95182AAAF	213	24.89	[TMEM186]
FLJ95184AAAF	540	59.15	[STAM]
FLJ95189AAAF	334	38.38	[LCMT1]
FLJ95192AAAF	153	16.84	[MYLK]
FLJ95194AAAF	601	63.87	[UBQLN4]
FLJ95197AAAF	475	51.07	[SLC46A2]
FLJ95198AAAF	574	64.66	[TRAF3IP2]
FLJ95200AAAF	372	41	[PPM1K]
FLJ95207AAAF	427	47.25	[IRF3]
FLJ95208AAAF	195	21.63	[PYCARD]
FLJ95212AAAF	344	40.04	[B4GALT4]
FLJ95217AAAF	422	47.88	[CCBL1]
FLJ95220AAAF	398	45.38	[ATG4A]
FLJ95222AAAF	1156	128.98	[NUP133]
FLJ95223AAAF	534	58.16	[NAGS]
FLJ95232AAAF	1008	114.76	[DHX36]
FLJ95237AAAF	302	33.36	[KLF7]
FLJ95240AAAF	428	48.66	[FBXL3]
FLJ95242AAAF	314	34.26	[HADH]
FLJ95243AAAF	329	37.62	[UCHL5]
FLJ95245AAAF	609	66.29	[AIFM1]
FLJ95246AAAF	759	85.23	[LIMA1]
FLJ95251AAAF	592	67.34	[TTLL2]
FLJ95252AAAF	491	56	[KCNS3]
FLJ95253AAAF	697	77.68	[ACSL6]
FLJ95254AAAF	505	56.64	[PCYOX1]
FLJ95255AAAF	468	50.14	[NUP50]
FLJ95256AAAF	474	51.29	[HADHB]
FLJ95258AAAF	447	50.17	[FBXO5]
FLJ95259AAAF	158	18.09	[PIGP]
FLJ95260AAAF	774	86.89	[LOC81691]
FLJ95261AAAF	300	33.86	[PLEKHA3]
FLJ95263AAAF	697	79.75	[TMEM168]
FLJ95264AAAF	222	25.08	[MDM1]
FLJ95267AAAF	735	83.09	[STON1]
FLJ95269AAAF	854	95.88	[PARP8]
FLJ95270AAAF	568	65.58	[RIOK1]
FLJ95272AAAF	528	60.59	[ACBD3]
FLJ95274AAAF	422	46.68	[PAX6]
FLJ95279AAAF	842	97.04	[PYGM]
FLJ95281AAAF	950	103.39	[ADAMTS1]

FLJ95282AAAF	551	61.66	[IL17RE]
FLJ95286AAAF	593	64.88	[ARSD]
FLJ95288AAAF	555	59.92	[FLVCR1]
FLJ95290AAAF	331	37.53	[CTSS]
FLJ95293AAAF	838	97.07	[HELLS]
FLJ95295AAAF	454	49.66	[GOPC]
FLJ95303AAAF	300	35.37	[MAK16]
FLJ95307AAAF	559	62.55	[PHACTR3]
FLJ95309AAAF	879	100.72	[AMPD2]
FLJ95310AAAF	312	34.75	[NAPG]
FLJ95311AAAF	407	46.99	[TRIM13]
FLJ95312AAAF	475	51.86	[SLC29A3]
FLJ95313AAAF	304	34.49	[GULP1]
FLJ95315AAAF	377	41.77	[ACTRT2]
FLJ95320AAAF	1012	115.31	[PHF20]
FLJ95321AAAF	703	77.99	[ATG7]
FLJ95323AAAF	332	37.81	[TOR1A]
FLJ95327AAAF	214	24.84	[PCTP]
FLJ95328AAAF	538	62.1	[ZNF155]
FLJ95330AAAF	542	59.85	[PGM3]
FLJ95332AAAF	215	24.7	[SCN3B]
FLJ95334AAAF	651	71.84	[PIAS1]
FLJ95340AAAF	379	42.29	[UEVLD]
FLJ95343AAAF	541	60.94	[PLA2G4C]
FLJ95344AAAF	376	42.53	[WBP4]
FLJ95353AAAF	228	24.97	[SCN4B]
FLJ95355AAAF	359	40.54	[TMOD1]
FLJ95356AAAF	941	105.53	[ANKMY1]
FLJ95357AAAF	923	102.9	[CCAR2]
FLJ95359AAAF	595	66.13	[SLC13A1]
FLJ95360AAAF	290	32.97	[CA8]
FLJ95362AAAF	639	70.27	[CNOT4]
FLJ95364AAAF	604	68.96	[ENTPD7]
FLJ95369AAAF	635	68.7	[SLC5A6]
FLJ95370AAAF	210	23.71	[NDUFS8]
FLJ95372AAAF	392	44.29	[BCAT2]
FLJ95374AAAF	351	40.62	[PRKACB]
FLJ95379AAAF	188	21.51	[MRPL35]
FLJ95380AAAF	854	98.55	[VPS41]
FLJ95388AAAF	586	68.36	[SLU7]
FLJ95393AAAF	470	53.92	[ZNHIT6]
FLJ95394AAAF	290	33.28	[CD274]
FLJ95398AAAF	312	35.72	[RPRD1A]
FLJ95399AAAF	411	44.67	[PLAGL1]
FLJ95400AAAF	381	42.03	[CYR61]
FLJ95411AAAF	793	89.84	[UHRF1]
FLJ95413AAAF	883	100.05	[ECT2]
FLJ95415AAAF	706	80.15	[SLC44A2]

FLJ95417AAAF	453	50.38	[ALDH3A1]
FLJ95421AAAF	621	71.93	[MTMR6]
FLJ95422AAAF	478	55.36	[IFIT1]
FLJ95429AAAF	538	60.67	[TROVE2]
FLJ95431AAAF	453	49.57	[ASB16]
FLJ95432AAAF	333	35.57	[AMMECR1]
FLJ95434AAAF	105	12.22	[LBH]
FLJ95435AAAF	711	80.49	[GZF1]
FLJ95437AAAF	150	17.07	[AIF1L]
FLJ95438AAAF	579	65.52	[CDC40]
FLJ95441AAAF	839	95.7	[ZNF347]
FLJ95444AAAF	1049	115.6	[KIAA0319L]
FLJ95445AAAF	960	107.33	[RBM19]
FLJ95446AAAF	924	101.93	[PCDH20]
FLJ95449AAAF	870	98.81	[POLK]
FLJ95451AAAF	1166	126.95	[TNKS2]
FLJ95456AAAF	568	63.52	[SGPL1]
FLJ95457AAAF	450	50.4	[TUBB3]
FLJ95458AAAF	479	52.77	[STAU2]
FLJ95459AAAF	687	77.33	[TGM2]
FLJ95460AAAF	481	55.77	[AKT2]
FLJ95461AAAF	805	87.79	[TACC1]
FLJ95462AAAF	720	80.35	[ACSL3]
FLJ95463AAAF	760	84.47	[DMTF1]
FLJ95464AAAF	484	57.3	[CCDC65]
FLJ95466AAAF	532	61.29	[CSGALNACT1]
FLJ95468AAAF	866	101.3	[NAA15]
FLJ95470AAAF	924	104.08	[EXOC2]
FLJ95471AAAF	178	18.92	[PCNP]
FLJ95473AAAF	438	51.62	[ZNF277]
FLJ95477AAAF	205	23.69	[EDARADD]
FLJ95480AAAF	609	69.28	[RASGRP2]
FLJ95481AAAF	440	50.11	[SCTR]
FLJ95483AAAF	385	43.79	[CTBS]
FLJ95484AAAF	739	83.26	[AP4B1]
FLJ95485AAAF	798	87.24	[PCDHB2]
FLJ95487AAAF	663	74.69	[PADI1]
FLJ95489AAAF	649	71.43	[DBN1]
FLJ95491AAAF	533	60.22	[FMO5]
FLJ95493AAAF	461	53.2	[FUCA1]
FLJ95494AAAF	280	32.33	[RRP7A]
FLJ95495AAAF	495	56	[CYP21A2]
FLJ95496AAAF	502	55.44	[ADAT1]
FLJ95500AAAF	840	93.7	[MCM8]
FLJ95503AAAF	172	18.99	[LGALSL]
FLJ95508AAAF	574	63.4	[NT5E]
FLJ95509AAAF	487	53.45	[SCHIP1]
FLJ95510AAAF	736	82.8	[PAPOLG]

FLJ95512AAAF	399	45.42	[LIPA]
FLJ95513AAAF	341	39.25	[CCNY]
FLJ95514AAAF	292	32.98	[GIMAP6]
FLJ95515AAAF	452	50.24	[EIF2B3]
FLJ95527AAAF	373	41.37	[CEP41]
FLJ95530AAAF	593	68.49	[PDE9A]
FLJ95535AAAF	1124	124.06	[ZEB1]
FLJ95536AAAF	592	64.4	[SLC13A2]
FLJ95537AAAF	520	59.91	[CYP4F8]
FLJ95539AAAF	273	29.34	[CUTC]
FLJ95540AAAF	369	42.08	[ENDOU]
FLJ95541AAAF	747	84.7	[NOX5]
FLJ95543AAAF	514	57.2	[AGXT2]
FLJ95552AAAF	335	37.38	[CTSH]
FLJ95553AAAF	828	91.51	[NLGN3]
FLJ95556AAAF	981	108.93	[EML4]
FLJ95559AAAF	406	44.82	[DCTN2]
FLJ95561AAAF	554	64.98	[PIGB]
FLJ95562AAAF	587	64.97	[KLHL3]
FLJ95568AAAF	359	39.73	[C4orf17]
FLJ95570AAAF	197	21.97	[AES]
FLJ95572AAAF	461	51.03	[CORO1A]
FLJ95573AAAF	958	108.98	[DIS3]
FLJ95575AAAF	482	49.92	[TYMP]
FLJ95576AAAF	128	14.73	[UBA52]
FLJ95577AAAF	886	101.27	[GTF3C3]
FLJ95578AAAF	401	46.56	[CRTAP]
FLJ95579AAAF	297	34.36	[RPL5]
FLJ95580AAAF	718	78.08	[ABCB8]
FLJ95582AAAF	870	93.4	[BCAR1]
FLJ95585AAAF	684	76.48	[TXLNB]
FLJ95587AAAF	384	43.2	[UPB1]
FLJ95590AAAF	425	47.35	[AADAT]
FLJ95591AAAF	186	21.12	[DCTN3]
FLJ95596AAAF	1102	123.62	[ADNP]
FLJ95597AAAF	412	46.37	[FBXW4]
FLJ95598AAAF	866	96.84	[DMGDH]
FLJ95599AAAF	96	10.86	[LINC01554]
FLJ95600AAAF	329	37.46	[ST3GAL4]
FLJ95601AAAF	485	53.64	[WDR13]
FLJ95602AAAF	199	21.76	[COMMD4]
FLJ95606AAAF	132	15.3	[SH2D1B]
FLJ95607AAAF	913	99.64	[BCAS3]
FLJ95608AAAF	461	52.32	[PORCN]
FLJ95609AAAF	309	35.12	[RSPH1]
FLJ95612AAAF	243	27.19	[PLEKHB1]
FLJ95613AAAF	228	25.2	[EIF4H]
FLJ95614AAAF	731	80.47	[RFWD2]

FLJ95619AAAF	617	66.76	[RSC1A1]
FLJ95622AAAF	657	75.64	[THOC1]
FLJ95623AAAF	631	73.93	[DEF6]
FLJ95624AAAF	548	58.8	[WFIKKN1]
FLJ95626AAAF	196	21.13	[SIT1]
FLJ95627AAAF	592	66.58	[HPSE2]
FLJ95630AAAF	381	41.95	[KCMF1]
FLJ95631AAAF	1024	116.16	[NLRC4]
FLJ95632AAAF	333	37.15	[ANKRD2]
FLJ95633AAAF	709	78.41	[NCSTN]
FLJ95634AAAF	313	35.99	[LUZP4]
FLJ95635AAAF	653	71.22	[CBFA2T3]
FLJ95638AAAF	651	74.11	[RRN3]
FLJ95640AAAF	674	74.85	[DLG5]
FLJ95641AAAF	531	60.58	[CDC73]
FLJ95644AAAF	652	70.44	[SLC20A2]
FLJ95645AAAF	353	38.7	[GNB5]
FLJ95647AAAF	1023	112.87	[ATP1A1]
FLJ95648AAAF	343	38.82	[RNF113A]
FLJ95649AAAF	617	68.3	[ATP6V1A]
FLJ95650AAAF	876	97.18	[KPNB1]
FLJ95654AAAF	573	66.45	[ZNF10]
FLJ95655AAAF	907	103.57	[TUBGCP3]
FLJ95658AAAF	615	68.61	[PRICKLE3]
FLJ95660AAAF	220	25.42	[POP4]
FLJ95661AAAF	700	78.76	[MYBL2]
FLJ95664AAAF	329	36.04	[PDLIM1]
FLJ95666AAAF	609	69.39	[ALB]
FLJ95667AAAF	660	75.31	[RARS]
FLJ95668AAAF	446	52.23	[CALCOCO2]
FLJ95673AAAF	794	88.33	[CDH12]
FLJ95674AAAF	282	32.69	[TRA2A]
FLJ95676AAAF	289	30.68	[C1QTNF7]
FLJ95677AAAF	617	68.51	[ETFDH]
FLJ95679AAAF	390	44.56	[SERPINB3]
FLJ95687AAAF	317	35.91	[RNF41]
FLJ95688AAAF	380	39.83	[VASP]
FLJ95691AAAF	342	39.79	[PRPF18]
FLJ95692AAAF	464	49.26	[SF3A2]
FLJ95693AAAF	605	67.83	[NFE2L2]
FLJ95694AAAF	609	68.66	[AFP]
FLJ95695AAAF	372	40.95	[ARPC1B]
FLJ95698AAAF	394	42.95	[DUSP4]
FLJ95699AAAF	519	58.19	[ZNF35]
FLJ95704AAAF	513	56.8	[SPINT1]
FLJ95705AAAF	480	50.53	[GATA2]
FLJ95707AAAF	571	62.54	[SLC40A1]
FLJ95712AAAF	153	17.83	[HOXC6]

FLJ95714AAAF	327	33.73	[IER5]
FLJ95716AAAF	732	82.82	[KEL]
FLJ95721AAAF	659	76.8	[CUL4A]
FLJ95723AAAF	926	105.9	[PTPN4]
FLJ95724AAAF	349	40.26	[TAF7]
FLJ95726AAAF	663	74.05	[PADI4]
FLJ95728AAAF	100	11.02	[PIN1P1]
FLJ95729AAAF	340	35.83	[EFNB3]
FLJ95730AAAF	376	42.4	[SERPINB9]
FLJ95736AAAF	911	103.95	[LIG4]
FLJ95737AAAF	780	85.45	[ACO2]
FLJ95739AAAF	372	40.09	[YBX3]
FLJ95740AAAF	394	44.85	[UGCG]
FLJ95742AAAF	555	64.43	[ZNF57]
FLJ95743SAAF	183	20.66	[UBE2H]
FLJ95746AAAF	352	38.23	[INHBC]
FLJ95748AAAF	276	31.47	[METTL1]
FLJ95750AAAF	240	27.17	[THEM4]
FLJ95751AAAF	1098	123.56	[TDRD7]
FLJ95752AAAF	193	21.4	[UBE2E1]
FLJ95753AAAF	501	55.42	[SLC7A11]
FLJ95758AAAF	243	28.74	[SYF2]
FLJ95759AAAF	382	44.91	[B4GALT6]
FLJ95760AAAF	199	22.75	[CRADD]
FLJ95762AAAF	825	89.74	[IL4R]
FLJ95765AAAF	272	31.74	[CH25H]
FLJ95767AAAF	420	49.96	[PRIM1]
FLJ95768AAAF	326	35.08	[FCN1]
FLJ95770AAAF	380	41.61	[SASH3]
FLJ95771AAAF	873	99.87	[ENPP1]
FLJ95772AAAF	211	24.14	[TIMP3]
FLJ95773AAAF	221	23.68	[TRIM7]
FLJ95774AAAF	233	26.41	[CHMP4C]
FLJ95776AAAF	467	51.14	[SIGLEC7]
FLJ95777AAAF	310	33.54	[USF1]
FLJ95778AAAF	444	50.67	[SERPINA10]
FLJ95779AAAF	334	35.54	[SNAPC2]
FLJ95780AAAF	277	30.37	[CBR1]
FLJ95782AAAF	297	32.64	[EDA2R]
FLJ95784AAAF	242	26.47	[EMC7]
FLJ95786AAAF	540	59.36	[GPR75]
FLJ95787AAAF	914	98.76	[AR]
FLJ95788AAAF	656	75.75	[CXXC1]
FLJ95789AAAF	238	26.67	[PROCR]
FLJ95790AAAF	172	18.76	[ZNF580]
FLJ95792AAAF	392	42.27	[GALK1]
FLJ95793AAAF	270	30.02	[HHEX]
FLJ95794AAAF	326	35.37	[APOF]

FLJ95795AAAF	170	19.3	[PPP3R1]
FLJ95796AAAF	198	22.25	[DUSP14]
FLJ95798AAAF	453	51.36	[GPR39]
FLJ95799AAAF	265	28.63	[UROS]
FLJ95800AAAF	244	26.86	[KLK6]
FLJ95801AAAF	523	58.97	[RORA]
FLJ95802AAAF	317	36.15	[APOE]
FLJ95803AAAF	208	22.63	[NOL3]
FLJ95804AAAF	229	26.62	[CWC15]
FLJ95805AAAN	245	27.79	[YWHAQ]
FLJ95806AAAF	142	16.79	[TXNL4A]
FLJ95808AAAF	533	59.72	[ARSB]
FLJ95809AAAF	493	54.26	[KRT83]
FLJ95811AAAF	303	33.25	[TEF]
FLJ95813AAAF	267	30.79	[PENK]
FLJ95815AAAF	274	31.11	[BPHL]
FLJ95816AAAF	349	39.02	[PTER]
FLJ95817AAAF	766	88.17	[ZER1]
FLJ95818AAAF	272	30.93	[RSPO3]
FLJ95819AAAF	242	27.48	[IL34]
FLJ95822AAAF	266	29.66	[RASL12]
FLJ95823AAAF	241	27.77	[PHOSPHO2]
FLJ95825AAAF	370	41.34	[CAMK1]
FLJ95827AAAF	447	48.28	[AMBN]
FLJ95831AAAF	373	41.6	[ASMT]
FLJ95832AAAF	142	14.99	[KCNE5]
FLJ95833AAAF	275	32.3	[MSANTD3]
FLJ95834AAAF	259	27.9	[IGFBP1]
FLJ95838AAAF	250	26.19	[LGALS3]
FLJ95839AAAF	470	52.28	[NKD1]
FLJ95840AAAF	303	33.87	[CTSZ]
FLJ95841AAAF	369	42.3	[IL2RG]
FLJ95843AAAF	368	42.97	[SNAPC1]
FLJ95848AAAF	226	25.33	[YAE1D1]
FLJ95849AAAF	261	29.15	[TMEM187]
FLJ95851AAAF	462	48.81	[INTS12]
FLJ95852AAAF	426	46.88	[ACTL6B]
FLJ95854AAAF	248	27.51	[RASL11B]
FLJ95855AAAF	390	43.53	[KCNJ11]
FLJ95856AAAF	492	55.8	[FUT11]
FLJ95858AAAF	284	32.76	[GPN3]
FLJ95860AAAF	305	33.3	[MRPL2]
FLJ95861AAAF	390	42.96	[MEIS1]
FLJ95862AAAF	234	25.47	[TK1]
FLJ95863AAAF	201	22.16	[ARL4D]
FLJ95864AAAF	401	44.96	[RBM17]
FLJ95865AAAF	190	20.71	[NTPCR]
FLJ95866AAAF	508	56.5	[CYP27B1]

FLJ95867AAAF	481	48.96	[AGFG2]
FLJ95869AAAF	416	43.99	[KLF15]
FLJ95870AAAF	429	47.31	[GDF2]
FLJ95872AAAF	355	40.84	[CCR8]
FLJ95874AAAF	169	19.04	[EREG]
FLJ95876AAAF	404	42.98	[NR2F6]
FLJ95878AAAN	527	59.54	[POLE2]
FLJ95879AAAF	269	29.43	[HOXB5]
FLJ95882AAAF	359	42.04	[FUT9]
FLJ95883AAAF	361	39.44	[POU3F4]
FLJ95884AAAF	320	34.53	[MYOD1]
FLJ95885AAAF	245	26.6	[EIF6]
FLJ95886AAAF	194	21.17	[CCDC53]
FLJ95889AAAF	706	78.75	[TMC4]
FLJ95890AAAF	309	32.71	[SLC39A2]
FLJ95892AAAF	335	38.18	[POLB]
FLJ95893AAAF	347	39.04	[MAGEB1]
FLJ95894AAAF	282	32.6	[SYCE1]
FLJ95895AAAF	309	34.34	[MAGEA1]
FLJ95896AAAF	408	44.5	[GAPDHS]
FLJ95897AAAF	435	48.64	[ACTL7A]
FLJ95899AAAF	410	43.14	[POU4F2]
FLJ95901AAAF	831	96.19	[RSPH10B2]
FLJ95902AAAF	753	83.95	[TCEB3B]
FLJ95906AAAF	199	22.62	[ICOS]
FLJ95911AAAF	293	33.93	[FBXO6]
FLJ95913AAAF	352	40.42	[KERA]
FLJ95917AAAF	165	18.34	[GADD45A]
FLJ95918AAAF	272	30.57	[IGFBP5]
FLJ95919AAAF	301	31.78	[AQP10]
FLJ95921AAAF	336	36.86	[RDH14]
FLJ95922AAAF	227	25.52	[TEX30]
FLJ95924AAAF	514	55.71	[SLC18A2]
FLJ95925AAAF	1034	117.88	[NLRP3]
FLJ95926AAAF	508	58.42	[FBXW11]
FLJ95927AAAF	267	31.57	[ACER3]
FLJ95929AAAF	750	87.33	[STAT1]
FLJ95931AAAF	994	113.4	[NLRP4]
FLJ95932AAAF	1007	117.48	[PMFBP1]
FLJ95939AAAF	977	109.79	[ZC3H7B]
FLJ95940AAAF	1199	136.97	[TAF2]
FLJ95942AAAF	847	96.58	[FTSJ3]
FLJ95943AAAF	507	53.59	[ARSA]
FLJ95944AAAF	492	54.24	[ANKH]
FLJ95945AAAF	491	56.12	[SYT9]
FLJ95948AAAF	437	48.51	[FJX1]
FLJ95953AAAF	318	35.82	[FYTTD1]
FLJ95954AAAF	639	73.45	[PARN]

FLJ95956AAAF	594	63.21	[FAM71A]
FLJ95962AAAF	523	56.8	[SLC7A10]
FLJ95967AAAF	437	48.9	[VPS4A]
FLJ95971AAAF	1184	128.99	[PCDH12]
FLJ95972AAAF	1368	156.47	[MED23]
FLJ95974AAAF	281	31.88	[WBSCR22]
FLJ95975AAAF	693	78.91	[ZBED5]
FLJ95977AAAF	204	22.15	[TSPAN13]
FLJ95978AAAF	364	38.53	[YBX2]
FLJ95982AAAF	913	103.98	[CUL4B]
FLJ95985AAAF	476	52.99	[TOM1L1]
FLJ95986SAAF	925	106.6	[DDX58]
FLJ95988AAAF	252	28.94	[KCNIP2]
FLJ95990AAAF	837	94.46	[SLITRK4]
FLJ95993AAAF	576	65.55	[MPP7]
FLJ96000AAAF	253	28.77	[CLIC4]
FLJ96001AAAF	394	45.64	[CYTH4]
FLJ96006AAAF	793	86.71	[PCDHB7]
FLJ96007AAAF	1310	141.54	[CDHR2]
FLJ96008AAAF	770	88.15	[CMTR2]
FLJ96011AAAF	1187	131.86	[BAIAP3]
FLJ96012AAAF	584	61.71	[IRF2BP1]
FLJ96016AAAF	586	66.62	[GBP5]
FLJ96018AAAF	323	37.31	[FAM49A]
FLJ96021AAAF	372	39.92	[MTCH1]
FLJ96025AAAF	240	27.37	[TP53INP1]
FLJ96027AAAF	971	106.39	[RECK]
FLJ96029AAAF	791	85.84	[ATRIP]
FLJ96033AAAF	441	49.33	[TCP11]
FLJ96035AAAF	295	33.2	[HLF]
FLJ96037AAAF	417	46.44	[WNT10A]
FLJ96038AAAF	430	49.48	[C3orf58]
FLJ96041AAAF	498	56.91	[NMT2]
FLJ96042AAAF	495	55.45	[ZNF212]
FLJ96043AAAF	852	94.58	[THSD1]
FLJ96045AAAF	354	40.46	[PAQR8]
FLJ96047AAAF	486	53.37	[DPEP2]
FLJ96049AAAF	647	71.33	[KCND1]
FLJ96050AAAF	412	46.53	[ASS1]
FLJ96053AAAF	552	62.29	[MID1]
FLJ96054AAAF	485	54.85	[ALDH3A2]
FLJ96055AAAF	508	55.07	[EPOR]
FLJ96058AAAF	532	60.31	[FMO1]
FLJ96060AAAF	431	44.34	[HOXB3]
FLJ96062AAAF	530	58.65	[FAM126B]
FLJ96065AAAF	933	105.19	[PPP4R1]
FLJ96067AAAF	645	72.58	[SLC9A9]
FLJ96070AAAF	300	33.58	[PHF13]

FLJ96071AAAF	331	38.21	[ST3GAL6]
FLJ96072AAAF	343	38.86	[ASPHD2]
FLJ96073AAAF	1331	148.13	[CNTNAP2]
FLJ96075AAAF	220	24.99	[DGCR6]
FLJ96081AAAF	889	100.29	[EDEM3]
FLJ96083AAAF	453	50.2	[PRUNE]
FLJ96084AAAF	415	45.72	[PCOLCE2]
FLJ96085AAAF	285	31.91	[STAR]
FLJ96087AAAF	399	42.69	[CIPC]
FLJ96091AAAF	306	34.37	[GIMAP1]
FLJ96092AAAF	542	62.92	[ZCCHC7]
FLJ96093AAAF	763	84.62	[AOC3]
FLJ96098SAAN	907	100	[LGR5]
FLJ96101AAAF	957	110.96	[SNX13]
FLJ96105AAAF	329	36.08	[FTSJ1]
FLJ96106AAAF	183	20.62	[RAP2A]
FLJ96108AAAF	271	31.11	[NXPH1]
FLJ96110AAAF	1030	114.33	[SEMA6A]
FLJ96113AAAF	805	92.47	[ACE2]
FLJ96118AAAF	796	91	[DPP10]
FLJ96120AAAF	327	36.58	[CACNG4]
FLJ96122AAAF	78	8.47	[PKIB]
FLJ96128AAAF	292	31.78	[OSER1]
FLJ96129AAAF	423	46.32	[CECR5]
FLJ96131AAAF	255	28.43	[PSMA3]
FLJ96134AAAF	507	55.44	[NUP54]
FLJ96136AAAF	336	39.21	[GAS7]
FLJ96138AAAF	555	62.23	[GRHL3]
FLJ96141AAAF	481	52.87	[PNPLA3]
FLJ96143AAAF	387	42.06	[PALM]
FLJ96145AAAF	216	23.73	[TMEM139]
FLJ96146AAAF	583	61.83	[ADAD2]
FLJ96152AAAF	409	46.29	[DEPTOR]
FLJ96153AAAF	506	57.57	[NAP1L3]
FLJ96154AAAF	679	73.65	[HSPA9]
FLJ96158AAAF	700	80.08	[CAPN2]
FLJ96159AAAF	256	30.18	[MRPL28]
FLJ96162AAAF	306	34.28	[YIPF1]
FLJ96167AAAF	330	36.34	[WDR5B]
FLJ96171AAAF	694	75.21	[GAS2L3]
FLJ96172AAAF	262	29.38	[NRM]
FLJ96176AAAF	916	100.31	[CDH4]
FLJ96177AAAF	692	79.11	[ZNF180]
FLJ96178AAAF	260	29.25	[HOXC9]
FLJ96181AAAF	369	38.92	[TREX1]
FLJ96182AAAF	111	13.01	[ASCC3]
FLJ96185AAAF	619	70.49	[GUCY1B3]
FLJ96187AAAF	803	87.9	[ZBTB17]

FLJ96188AAAF	1032	117.41	[KIF5A]
FLJ96195AAAF	394	44.76	[ACTR2]
FLJ96196AAAF	478	52.04	[ZNF410]
FLJ96197AAAF	349	38.01	[CTGF]
FLJ96198AAAF	480	55.34	[CFLAR]
FLJ96200AAAF	354	39.37	[PON2]
FLJ96205AAAF	862	98.68	[ITCH]
FLJ96207AAAF	1203	133.24	[NOS3]
FLJ96210AAAF	875	100.89	[DDX10]
FLJ96211AAAF	701	74.47	[MAMLD1]
FLJ96216AAAF	584	61.73	[BCAS1]
FLJ96220AAAF	283	31.86	[KLF6]
FLJ96222AAAF	406	44.97	[BHMT]
FLJ96223AAAF	315	34.77	[FCGR2A]
FLJ96225AAAF	641	70.38	[HSPA1L]
FLJ96226AAAF	418	46.98	[AP3M2]
FLJ96227AAAF	363	40.92	[APOA5]
FLJ96228AAAF	427	47.88	[KNG1]
FLJ96229SAAF	741	83.54	[RNASEL]
FLJ96231AAAF	451	51.83	[PTK6]
FLJ96234AAAF	822	94.64	[FER]
FLJ96235AAAF	225	25.01	[PSPH]
FLJ96236AAAF	693	76.76	[TGM3]
FLJ96239AAAF	569	63.91	[CD96]
FLJ96240AAAF	487	55.63	[STK4]
FLJ96241AAAF	703	76.13	[USHBP1]
FLJ96242AAAF	517	58.24	[GTF2F1]
FLJ96246AAAF	403	44.13	[PEX13]
FLJ96251AAAF	395	45.32	[MRPS31]
FLJ96253AAAF	411	45.49	[KCNK2]
FLJ96254AAAF	894	103.92	[ACTN2]
FLJ96255AAAF	459	51.57	[IL7R]
FLJ96256AAAF	500	56.85	[LIPG]
FLJ96264AAAF	237	26.22	[CLNS1A]
FLJ96269AAAF	218	25.67	[GSTM5]
FLJ96270AAAF	478	56.36	[GAS8]
FLJ96271AAAF	292	33.53	[TTC1]
FLJ96273AAAF	160	17.74	[C22orf24]
FLJ96274AAAF	493	54.97	[CSAD]
FLJ96276AAAF	499	55.73	[GSPT1]
FLJ96281AAAF	330	38.94	[DUSP11]
FLJ96284AAAN	191	21.31	[RHOG]
FLJ96285AAAF	332	36.86	[KCNK17]
FLJ96287AAAF	244	27.95	[ULBP3]
FLJ96288AAAF	241	27.45	[ODF1]
FLJ96292AAAF	580	63.13	[SLC5A7]
FLJ96293AAAF	308	34.49	[COPE]
FLJ96295AAAF	395	44.45	[GTF2H2]

FLJ96296AAAF	1099	122.09	[SLC12A6]
FLJ96299AAAF	544	60.67	[PAK3]
FLJ96300AAAF	836	94.13	[TMTC2]
FLJ96301AAAF	338	37.29	[LSAMP]
FLJ96302AAAF	1115	125.12	[EIF2AK3]
FLJ96307AAAF	344	39.24	[MBIP]
FLJ96311AAAF	553	62.51	[IL20RA]
FLJ96315AAAF	423	46.32	[IVD]
FLJ96317AAAF	517	58.9	[CHRND]
FLJ96319AAAF	302	34.28	[AP1AR]
FLJ96321SAAN	249	28.11	[STX10]
FLJ96325AAAF	551	60.86	[SLC22A13]
FLJ96326AAAF	619	71.45	[CDC16]
FLJ96327AAAF	839	97	[VAV2]
FLJ96331AAAF	460	53.62	[ANGPTL3]
FLJ96333AAAF	626	70.9	[MAP3K3]
FLJ96334AAAF	499	55.88	[LIPC]
FLJ96336AAAF	668	73.35	[ZBTB20]
FLJ96337AAAF	955	107.01	[CLSTN2]
FLJ96339AAAF	382	42.05	[FETUB]
FLJ96342AAAF	202	22.01	[LIF]
FLJ96343AAAF	473	54.34	[GABRB3]
FLJ96344AAAF	457	51.84	[CHRNA1]
FLJ96347AAAF	515	57.05	[NLK]
FLJ96348AAAF	449	50.09	[TUBA8]
FLJ96349AAAF	594	66.73	[SLC22A14]
FLJ96350AAAF	332	38.27	[CGRRF1]
FLJ96352AAAF	297	33.93	[MC2R]
FLJ96353AAAF	406	47.67	[UST]
FLJ96356AAAF	233	26	[LIN7A]
FLJ96359AAAF	369	41.94	[PCYT1B]
FLJ96361AAAF	390	43.6	[HTR1B]
FLJ96362AAAF	223	24.61	[CFC1]
FLJ96363AAAF	299	33.39	[RNASEH2A]
FLJ96365AAAF	254	28.39	[SRD5A2]
FLJ96367AAAF	487	53.89	[BPI]
FLJ96370AAAF	501	58.06	[CYP8B1]
FLJ96371AAAF	392	43.14	[AGXT]
FLJ96373AAAF	633	69.54	[XRCC1]
FLJ96374AAAF	201	22.17	[RAB1B]
FLJ96375AAAF	575	65.39	[IL1RL2]
FLJ96376AAAF	1039	113.39	[ITGA2B]
FLJ96377AAAF	427	47.1	[AKAP5]
FLJ96378AAAF	226	25.04	[CD79A]
FLJ96380AAAF	585	64.48	[FZD5]
FLJ96381AAAF	361	38.78	[GPR25]
FLJ96382AAAF	356	40.52	[ST8SIA1]
FLJ96383AAAF	471	53.76	[MMP13]

FLJ96385AAAF	465	52.35	[KYNU]
FLJ96388AAAF	512	55.18	[KLF11]
FLJ96392AAAF	391	42.55	[RAD9A]
FLJ96395AAAF	527	59.71	[CAT]
FLJ96396AAAF	467	53.52	[ERO1LB]
FLJ96397AAAF	187	21.05	[CENPBD1]
FLJ96398AAAF	122	14.21	[PFDN1]
FLJ96399AAAF	216	24.21	[RAB2B]
FLJ96400AAAF	180	20.46	[NIP7]
FLJ96402AAAF	181	20.92	[RGS5]
FLJ96403AAAF	360	39.58	[DNASE2]
FLJ96405AAAF	533	56.65	[PHGDH]
FLJ96407AAAF	265	29.23	[C5orf15]
FLJ96411AAAF	187	20.88	[EID1]
FLJ96414AAAF	223	24.84	[UCHL1]
FLJ96415AAAF	222	25.7	[GSTA4]
FLJ96416AAAF	191	21.87	[PDCD6]
FLJ96417AAAF	111	12.96	[NGFRAP1]
FLJ96418AAAF	593	66.03	[KLHL2]
FLJ96420AAAF	178	19.26	[PRAF2]
FLJ96421AAAF	297	32.48	[TACO1]
FLJ96423AAAF	235	27.58	[RGS18]
FLJ96425AAAF	368	41.01	[RIC3]
FLJ96427AAAF	535	57.24	[ALDH5A1]
FLJ96428AAAF	324	37.01	[PSMD7]
FLJ96429AAAF	183	20.33	[C11orf58]
FLJ96431AAAF	319	36.67	[CXorf38]
FLJ96432AAAF	221	24.15	[TSPAN2]
FLJ96433AAAF	356	39.35	[SERTAD4]
FLJ96434AAAF	502	57.58	[CYP2J2]
FLJ96435AAAF	492	57.23	[PPIL4]
FLJ96436AAAF	223	24.65	[PSMD9]
FLJ96439AAAF	77	8.38	[HMGN3]
FLJ96442AAAF	532	59.78	[CPNE2]
FLJ96443AAAF	151	16.93	[NGB]
FLJ96445AAAF	385	41.44	[CIART]
FLJ96446AAAF	432	49.88	[GFAP]
FLJ96447AAAF	489	54.23	[CORO1B]
FLJ96450AAAF	251	28	[HDHD3]
FLJ96451AAAF	268	30.62	[MLF1]
FLJ96453AAAF	234	26.65	[STARD3NL]
FLJ96456AAAF	194	20.62	[MRPS11]
FLJ96457AAAF	124	13.73	[TAX1BP3]
FLJ96458AAAF	68	8.21	[SPTSSA]
FLJ96461AAAF	308	36.03	[TECR]
FLJ96463AAAF	128	13.55	[H2AFZ]
FLJ96466AAAF	326	35.12	[FOSL2]
FLJ96469AAAF	434	47.17	[ENO1]

FLJ96471AAAF	117	14.07	[GABARAPL1]
FLJ96472AAAF	287	31.41	[LAIR1]
FLJ96474AAAF	419	46.53	[BRF2]
FLJ96475AAAF	292	32.52	[CCND3]
FLJ96477AAAF	461	53.88	[NUCB1]
FLJ96479AAAF	275	32.34	[BRMS1L]
FLJ96484AAAF	305	33.25	[KIAA1191]
FLJ96485AAAF	799	88.03	[ITGB5]
FLJ96486AAAF	743	84.41	[DHX32]
FLJ96487AAAF	163	19.62	[RSL24D1]
FLJ96490AAAF	279	31.71	[MRPL46]
FLJ96491AAAF	448	50.14	[MAP2K5]
FLJ96492AAAF	563	61.33	[GMEB1]
FLJ96493AAAF	256	29.23	[KCNIP3]
FLJ96494AAAF	494	56.04	[USP14]
FLJ96496AAAF	259	30.01	[BPGM]
FLJ96498AAAF	257	29.84	[MRPS15]
FLJ96499AAAF	239	26.93	[EFHD1]
FLJ96500AAAF	504	56.97	[MYOC]
FLJ96501AAAF	210	22.48	[CDC42EP2]
FLJ96502AAAF	418	45.68	[FUZ]
FLJ96503AAAF	183	21.11	[C15orf41]
FLJ96504AAAF	472	51.89	[CPQ]
FLJ96505AAAF	201	23	[MRPS10]
FLJ96506AAAF	760	87.69	[FAP]
FLJ96510AAAF	573	66.25	[FAM200A]
FLJ96512AAAF	131	14.56	[FAM86C1]
FLJ96515AAAF	309	34.41	[THAP7]
FLJ96516AAAF	116	13.46	[NDUFA5]
FLJ96517AAAF	267	30.24	[MRPL9]
FLJ96519AAAF	243	26.29	[TMEM174]
FLJ96522AAAF	320	36.2	[ZNF330]
FLJ96523AAAF	202	21.4	[TM4SF4]
FLJ96524AAAF	662	75.53	[MX1]
FLJ96525AAAF	215	24.22	[C5orf28]
FLJ96527AAAF	256	29.48	[RNASET2]
FLJ96529AAAF	337	37.67	[CA14]
FLJ96531AAAF	640	69.46	[WRNIP1]
FLJ96532AAAF	505	56.78	[PDIA3]
FLJ96533AAAF	775	87.75	[GCC1]
FLJ96537AAAF	416	46.16	[TRAF1]
FLJ96538AAAF	483	54.86	[ERLEC1]
FLJ96541AAAF	250	28.06	[HOXB9]
FLJ96546AAAF	300	33.79	[CENPO]
FLJ96547AAAF	549	61.99	[GNL3]
FLJ96550AAAF	505	57.68	[ZNF649]
FLJ96551AAAF	269	30.39	[SURF4]
FLJ96552AAAF	445	51.42	[CDS2]

FLJ96553AAAF	536	60.03	[KPNA6]
FLJ96554AAAF	622	69.84	[DDX41]
FLJ96556AAAF	51	5.78	[ATP5E]
FLJ96558AAAF	205	22.68	[RAB1A]
FLJ96559AAAF	327	38.17	[VPS26A]
FLJ96560AAAF	220	24.07	[CREG1]
FLJ96562AAAF	146	16.45	[RPS16]
FLJ96563AAAF	495	55.69	[DCAF4]
FLJ96565AAAF	245	24.27	[MAPK1IP1L]
FLJ96567AAAF	703	77.41	[PCCA]
FLJ96571AAAF	489	52.08	[DPH2]
FLJ96574AAAF	455	50.5	[TNFRSF1A]
FLJ96576AAAF	321	36.08	[ANXA4]
FLJ96579AAAF	463	50.33	[SUCLA2]
FLJ96581AAAF	188	21.25	[RWDD4]
FLJ96582AAAF	329	36.65	[SCAMP2]
FLJ96583AAAF	362	41.83	[SDF4]
FLJ96585AAAF	351	39.92	[MCU]
FLJ96587AAAF	640	71.18	[UBA2]
FLJ96588AAAF	351	39.51	[MOAP1]
FLJ96590AAAF	200	22.41	[UBE2K]
FLJ96591AAAF	501	54.38	[CAMKV]
FLJ96593AAAF	515	59.38	[FAR1]
FLJ96594AAAF	247	27.4	[RNF182]
FLJ96595AAAF	313	34.29	[MGLL]
FLJ96598AAAF	344	39.42	[MGME1]
FLJ96599AAAF	564	66.73	[EIF3L]
FLJ96600AAAF	418	47.61	[ACTR3B]
FLJ96602AAAF	464	54.12	[CEP55]
FLJ96605AAAF	260	28.84	[RDH13]
FLJ96611AAAF	251	28.8	[DERL1]
FLJ96613AAAF	596	67.63	[VPS33A]
FLJ96614AAAF	269	29.08	[MPZL1]
FLJ96615AAAF	761	83.57	[SEMA4A]
FLJ96617AAAF	543	61.16	[EHD2]
FLJ96618AAAF	608	68.45	[TDP1]
FLJ96619AAAF	720	76.61	[GTSE1]
FLJ96622AAAF	482	53.35	[MKRN1]
FLJ96625AAAF	669	73.64	[FEM1A]
FLJ96626AAAF	684	77.79	[FOXRED2]
FLJ96627AAAF	714	81.9	[CAPN1]
FLJ96628AAAF	847	92.62	[MAP3K11]
FLJ96630AAAF	478	52.89	[METAP2]
FLJ96631AAAF	655	71.84	[TNFRSF21]
FLJ96633AAAF	383	43	[PRSS23]
FLJ96634AAAF	469	53	[ETS2]
FLJ96637AAAF	217	25.21	[GRB2]
FLJ96639AAAF	392	43.09	[FAM53C]

FLJ96640AAAF	339	37.78	[CIAO1]
FLJ96642AAAF	769	83.4	[GPHN]
FLJ96643AAAF	798	87.13	[USP10]
FLJ96646AAAF	309	35.17	[FAM26E]
FLJ96647AAAF	362	40.17	[MFAP3]
FLJ96650AAAF	396	45.78	[SNIP1]
FLJ96651AAAF	270	29.06	[TMEM176B]
FLJ96653AAAF	242	27.32	[VAPA]
FLJ96655AAAF	440	47.71	[SMAP1]
FLJ96656AAAF	346	38.91	[ERLIN1]
FLJ96657AAAF	172	19.84	[BLOC1S6]
FLJ96658AAAF	797	90.77	[TRPC4AP]
FLJ96662AAAF	247	27.16	[TM2D3]
FLJ96664AAAF	792	91.21	[KIFAP3]
FLJ96665AAAF	336	36.39	[TMEM19]
FLJ96666AAAF	421	47.3	[PIP4K2C]
FLJ96667AAAF	684	77.23	[KBTBD7]
FLJ96670AAAF	274	30.28	[TOLLIP]
FLJ96671AAAF	520	57.29	[HMGCS1]
FLJ96672AAAF	285	32.77	[GOLPH3L]
FLJ96673AAAF	301	33.81	[SCO1]
FLJ96675AAAF	518	57.66	[MFSD8]
FLJ96681AAAF	113	12.45	[DYNLT1]
FLJ96682AAAF	171	18.02	[TIMM17A]
FLJ96683AAAF	197	22.01	[ZNF581]
FLJ96685AAAF	106	12.25	[ATPIF1]
FLJ96686AAAF	278	31.86	[HAUS1]
FLJ96687AAAF	120	13.19	[TMEM230]
FLJ96688AAAF	196	22.45	[RGS1]
FLJ96689AAAF	188	21.01	[GGCT]
FLJ96690AAAF	216	24.13	[GSTZ1]
FLJ96691AAAF	180	20.27	[TRAPPC3]
FLJ96693AAAF	746	83.59	[BOP1]
FLJ96694AAAF	366	38.34	[ZNF385A]
FLJ96695AAAF	208	23.86	[THEM6]
FLJ96697AAAF	610	68.04	[WDR46]
FLJ96698AAAF	68	7.91	[NREP]
FLJ96699AAAF	609	69.78	[XRCC6]
FLJ96700AAAF	334	38.84	[PRMT8]
FLJ96701AAAF	424	46.78	[RBM23]
FLJ96704AAAF	156	18	[NCBP2]
FLJ96706AAAF	619	67.93	[ZNF668]
FLJ96708AAAF	346	38.45	[SAE1]
FLJ96710AAAF	180	20.32	[PTTG1IP]
FLJ96711AAAF	167	18.82	[GEMIN6]
FLJ96712AAAF	223	23.8	[LRRC29]
FLJ96713AAAF	587	65.08	[ASB2]
FLJ96715AAAF	555	62.13	[MON1A]

FLJ96716AAAF	508	57.12	[P4HB]
FLJ96718AAAF	288	33.68	[TRA2B]
FLJ96720AAAF	456	50.7	[DDOST]
FLJ96722AAAF	270	31.29	[SLBP]
FLJ96723AAAF	371	42.92	[LRRC2]
FLJ96725AAAF	228	25.69	[RNF114]
FLJ96727AAAF	279	31.93	[NMNAT1]
FLJ96729AAAF	385	42.18	[IDH3B]
FLJ96731AAAF	472	52.39	[SELENBP1]
FLJ96735AAAF	378	41.54	[B3GALT4]
FLJ96740AAAF	384	46.41	[APOBEC3G]
FLJ96742AAAF	520	58.14	[SPPL2A]
FLJ96744AAAF	313	33.75	[KCNK6]
FLJ96745AAAF	312	34.25	[TRADD]
FLJ96746AAAF	417	45.01	[HPN]
FLJ96747AAAF	282	28.99	[CD320]
FLJ96748AAAF	226	24.68	[ARL6IP6]
FLJ96749AAAF	423	48.99	[TTLL1]
FLJ96750AAAF	367	40.54	[DRG1]
FLJ96752AAAF	339	39.2	[IGBP1]
FLJ96753AAAF	496	55.82	[FSD1]
FLJ96754AAAF	518	58.2	[RORC]
FLJ96756AAAF	453	50.91	[CHMP7]
FLJ96757AAAF	321	35.34	[LRRC46]
FLJ96761AAAF	291	31.67	[IGFBP3]
FLJ96762AAAF	578	65.51	[RARS2]
FLJ96764AAAF	465	52.6	[SNX8]
FLJ96766AAAF	287	33.29	[IQCK]
FLJ96768AAAF	256	29.72	[NSMCE1]
FLJ96769AAAF	142	15.94	[COTL1]
FLJ96771AAAF	460	49.98	[AZIN2]
FLJ96772AAAF	396	42.45	[MVK]
FLJ96773AAAF	620	68.3	[CFAP52]
FLJ96774AAAF	341	36.77	[C1orf56]
FLJ96776AAAF	206	23.31	[SOSTDC1]
FLJ96777AAAF	586	67.29	[CLHC1]
FLJ96782AAAF	214	24.48	[N6AMT2]
FLJ96784AAAF	336	35.33	[HIBADH]
FLJ96787AAAF	423	48.11	[AP1M2]
FLJ96790AAAF	341	39.87	[CAB39]
FLJ96791AAAF	403	46.11	[RPL3]
FLJ96792AAAF	149	16.78	[CALM2]
FLJ96793AAAF	156	17.96	[RPS27A]
FLJ96795AAAF	712	81.16	[USP44]
FLJ96797AAAF	641	72.56	[RFX4]
FLJ96799AAAF	589	65.31	[PPP2R1A]
FLJ96800AAAF	526	60.2	[GMCL1P1]
FLJ96801AAAF	212	24.42	[KDELR2]

FLJ96802AAAF	551	60.32	[RCBTB2]
FLJ96803AAAF	584	63.48	[GTPBP1]
FLJ96805AAAF	672	75.63	[SHCBP1]
FLJ96806AAAF	349	38.7	[CRYZL1]
FLJ96807AAAF	341	37.65	[CD2BP2]
FLJ96809AAAF	480	54.49	[SESN2]
FLJ96811AAAF	931	103.32	[GPATCH1]
FLJ96813AAAF	437	48.77	[FNTB]
FLJ96814AAAF	540	59.69	[CNOT2]
FLJ96816AAAF	251	27.56	[CYB561]
FLJ96817AAAF	297	34.18	[STX4]
FLJ96820AAAF	462	50.56	[ERRFI1]
FLJ96821AAAF	523	58.86	[SLC35F5]
FLJ96824AAAF	108	12.27	[RBX1]
FLJ96825AAAF	493	54.53	[FSCN1]
FLJ96826AAAF	268	30.34	[TSPAN5]
FLJ96827AAAF	490	55.42	[KATNAL1]
FLJ96832AAAF	420	46.44	[PELI2]
FLJ96834AAAF	330	36.84	[GINM1]
FLJ96835AAAF	735	82.72	[RPS6KA1]
FLJ96839AAAF	260	29.19	[CD27]
FLJ96847AAAF	566	63.92	[FBXW5]
FLJ96848AAAF	187	20.84	[MRPS28]
FLJ96850AAAF	291	31.87	[EXOSC7]
FLJ96853AAAF	184	20.73	[REEP6]
FLJ96854AAAF	269	29.14	[WBP1]
FLJ96855AAAF	267	30.8	[ZDHHC12]
FLJ96856AAAF	634	65.57	[ZBTB22]
FLJ96857AAAF	429	46.94	[FCRL1]
FLJ96858AAAF	359	40.31	[NANS]
FLJ96861AAAF	223	25.02	[TWSG1]
FLJ96863AAAF	220	25.28	[ATG10]
FLJ96864AAAF	83	9.05	[C9orf16]
FLJ96865AAAF	176	18.43	[C19orf43]
FLJ96866AAAF	153	17.77	[UBE2L6]
FLJ96869AAAF	208	22.49	[CRIP2]
FLJ96870AAAF	148	16.07	[MRPL27]
FLJ96871AAAF	218	24.58	[ARL6IP4]
FLJ96872AAAF	292	31.54	[AQP3]
FLJ96875AAAN	258	28.97	[SNAP29]
FLJ96877AAAF	614	70.97	[FRMD6]
FLJ96878AAAF	210	23.05	[TSPAN31]
FLJ96879AAAF	327	37.03	[MAPRE2]
FLJ96880AAAF	394	43.52	[TMEM79]
FLJ96884AAAF	249	28.4	[UTP23]
FLJ96885AAAF	307	34.95	[SEC22A]
FLJ96888AAAF	579	64.23	[MAP3K7]
FLJ96890AAAF	119	13.71	[B2M]

FLJ96894AAAF	188	21.17	[TMCO1]
FLJ96896AAAF	504	56.45	[TRMT2B]
FLJ96899AAAF	545	61.58	[ZKSCAN4]
FLJ96900AAAF	247	28.26	[ATP6V1D]
FLJ96901AAAF	632	70.97	[RACGAP1]
FLJ96903AAAF	254	29.46	[CCDC90B]
FLJ96905AAAF	269	28.69	[GATAD1]
FLJ96906AAAF	495	54.62	[SLC17A5]
FLJ96908AAAF	311	35.23	[PPIL6]
FLJ96911AAAF	282	31.52	[HMGN5]
FLJ96915AAAF	303	32.54	[PECR]
FLJ96916AAAF	628	70.07	[NTN4]
FLJ96919AAAF	270	29.29	[CELA3B]
FLJ96920AAAF	213	24.15	[VHL]
FLJ96926AAAF	617	68.67	[FEM1C]
FLJ96928AAAF	399	44.16	[PDSS2]
FLJ96929AAAF	763	84.39	[TAS1R1]
FLJ97017SAAN	153	17.26	[BLOC1S1]
FLJ97334AAAN	708	80.97	[ZRANB1]
FLJ97373SAAN	308	35.1	[TSPY2]
FLJ97900SAAN	1181	129.3	[ITGA2]
FLJ98110SAAN	706	78.85	[BCL6]
FLJ98231SAAN	353	40.13	[LPAR3]
FLJ98329SAAN	364	39.2	[SSTR5]
FLJ98356SAAN	1118	122.29	[DSG2]
FLJ99613SAAN	545	57.73	[AIRE]
FMG_00325AAAN	1132	130.67	[JAK2]
N0500-A06-2_FDD_603[illegible]	1143	130.9	[PTPRC]
N0751-B02-2_FDD_001	491	52.41	[PRCC]
N0751-C01-7_FDD_001[illegible]	854	94.48	[AKAP4]
N0751-C11-1_FDD_001[illegible]	1174	133.9	[POLR2B]
N0751-D07-2_FDD_001[illegible]	972	107.98	[CSF1R]
N0751-E01-1_FDD_001[illegible]	193	20.53	[BBC3]
N0751-E12-1_FDD_001[illegible]	370	40.98	[WNT1]
N0751-F01-1_FDD_001[illegible]	239	26.89	[FGF3]
N0752-A02-2_FDD_005[illegible]	440	46.95	[HTR6]
N0752-A05-1_FDD_005[illegible]	375	42.19	[NPY4R]
N0752-B12-1_FDD_006[illegible]	90	9.04	[MUCL1]
N0752-C08-3_FDD_006[illegible]	389	42.13	[UTS2R]
N0752-C09-1_FDD_006[illegible]	595	60.03	[GATA6]
N0752-D01-1_FDD_006[illegible]	358	39.76	[PTGER2]
N0752-D02-1_FDD_006	1461	160.02	[NEO1]
N0752-D05-2_FDD_006	604	67.75	[NTN1]
N0752-D10-1_FDD_006[illegible]	398	42.61	[KISS1R]
N0752-D11-1_FDD_006[illegible]	385	41.13	[F2RL3]
N0752-E02-2_FDD_006[illegible]	330	37.14	[FFAR2]
N0752-E03-2_FDD_006[illegible]	1336	143.73	[GRIN2D]
N0752-E11-1_FDD_006[illegible]	1078	120.57	[CASR]

N0752-F01-1_FDD_006	300	31.44	[FFAR1]
N0752-F05-2_FDD_006	1194	136.14	[PLCB4]
N0752-F06-2_FDD_006	388	41.89	[SSTR4]
N0752-F07-2_FDD_006	323	36.04	[MC3R]
N0752-F11-1_FDD_006	253	28.29	[IL12A]
N0752-G02-1_FDD_006	491	54.57	[FBXL7]
N0752-G05-1_FDD_006	146	15.79	[IL13]
N0752-G06-1_FDD_006	520	56.84	[ADRA1B]
N0752-G08-2_FDD_006	365	41.68	[HTR1E]
N0752-H11-1_FDD_006	328	36.1	[NPBWR1]
N0753-A07-1_FDD_006	360	39.71	[CNR2]
N0753-A10-1_FDD_006	1180	128.87	[GRM5]
N0753-A11-1_FDD_006	638	69.97	[GAB2]
N0753-A12-2_FDD_007	479	53.05	[CHRM4]
N0753-B03-2_FDD_006	858	97.77	[TLR5]
N0753-B06-1_FDD_006	277	29.34	[TNFRSF4]
N0753-B07-2_FDD_006	140	16.25	[FGF8]
N0753-B08-2_FDD_006	430	47.82	[NPFFR1]
N0753-B09-1_FDD_006	732	81.75	[GUCY1A2]
N0753-C12-1_FDD_007	108	12.45	[GHRH]
N0753-D03-1_FDD_007	208	22.9	[FGF6]
N0753-D05-1_FDD_007	2871	312.3	[FBN1]
N0753-D10-1_FDD_007	450	49.95	[ADRA2B]
N0753-E02-2_FDD_007	465	52.2	[TACR3]
N0753-F03-1_FDD_007	185	20.89	[GDNF]
N0753-H01-2_FDD_007	345	35.69	[GAS1]
N0753-H02-1_FDD_007	188	21.58	[IFNA2]
N0754-A08-2_FDD_007	301	32.12	[HOXB1]
N0754-B05-1_FDD_007	366	41.71	[HTR1F]
N0754-B10-2_FDD_007	343	37.43	[TBXA2R]
N0754-B12-1_FDD_007	424	48.05	[BMP10]
N0754-C08-1_FDD_008	211	23.5	[FGF20]
N0754-C09-1_FDD_008	189	22.11	[IFNA7]
N0754-D05-2_FDD_008	339	39.09	[TAAR1]
N0754-D08-1_FDD_008	189	22.06	[IFNA14]
N0754-D10-1_FDD_005	1614	179.03	[LRP5]
N0756-E12-1_FDD_500	197	21.76	[IL17C]
N0756-F03-4_FDD_500	115	12.86	[PTH]
N0756-F08-2_FDD_500	169	18.72	[IL36G]
N0756-H07-1_FDD_500	251	27.95	[FGF23]
N0757-B06-1_FDD_500	353	38.87	[S1PR2]
N0757-B09-1_FDD_500	364	40.57	[OPN1LW]
N0757-F10-2_FDD_501	680	76.13	[ANOS1]
N0761-A08-2_FDD_003	494	54.88	[PIK3R5]
N0761-A09-2_FDD_003	1167	131.21	[PLCB3]
N0761-B08-2_FDD_003	298	34.26	[AZGP1]
N0761-D05-1_FDD_003	1168	130.62	[ADCY6]
N0761-D10-1_FDD_003	801	89.81	[PI4KB]

N0761-E06-1_FDD_003	1188	133.19	[INPP5D]
N0761-F03-1_FDD_003	620	67.37	[DTX1]
N0761-F04-1_FDD_003	148	15.21	[CDC42EP5]
N0767-C08-1_FDD_103	1562	172.24	[ARHGEF11]
N0770-F09-2_FDD_105	155	17.5	[IL17A]
N0775-G04-2_FDD_106	184	18.57	[CITED4]
N0781-C05-1_FDD_113	263	27.66	[FSTL3]
N0783-G04-1_FDD_115	565	63.55	[FZD2]
N0783-H05-1_FDD_115	597	66.63	[UNC93B1]
N0796-H01-1_FDD_512	782	86.04	[VPS51]
N0796-H06-1_FDD_514	1028	108.53	[COL6A1]
N0802-D07-2_FDD_301	170	19.3	[CAMP]
N0807-C07-1_FDD_502	88	10.6	[SYCE3]
N0807-D10-1_FDD_502	100	11.2	[PTH2]
N0807-E09-1_FDD_502	97	10.5	[DEFA4]
N0807-G10-1_FDD_502	101	10.84	[PF4]
N0808-B12-1_FDD_503	142	15.45	[RLN3]
N0808-C05-1_FDD_503	120	12.92	[GNRH2]
N0808-D11-1_FDD_503	140	15.56	[FABP12]
N0808-F04-1_FDD_503	118	13.92	[ATP6V1G3]
N0808-F07-1_FDD_503	126	13.25	[NPPC]
N0808-F12-1_FDD_503	147	16.85	[MAP1LC3C]
N0809-C12-1_FDD_504	210	24.01	[TNNI3]
N0809-D04-1_FDD_503	170	19.66	[FGF22]
N0809-D08-1_FDD_504	189	21.99	[IFNA8]
N0809-F07-1_FDD_504	185	21.7	[CSN1S1]
N0810-B11-2_FDD_505	274	29.04	[CCS]
N0810-C04-2_FDD_504	235	26.92	[TICAM2]
N0810-C09-2_FDD_505	231	26.98	[IL22RA2]
N0810-D09-2_FDD_505	263	30.12	[RAET1E]
N0810-H03-1_FDD_504	231	26.56	[KLRF1]
N0811-H03-2_FDD_505	304	33.58	[KIR2DS4]
N0812-F09-1_FDD_507	325	36.6	[MC5R]
N0813-A11-1_FDD_508	454	50.83	[NCR3LG1]
N0813-C02-2_FDD_507	350	40.04	[GNAT1]
N0813-E04-2_FDD_507	374	41.12	[RXFP4]
N0813-G11-1_FDD_508	462	49.52	[ADRA2C]
N0813-H03-2_FDD_507	365	40.8	[WNT16]
N0814-C03-1_FDD_508	516	56.94	[KPNA7]
N0816-A02-1_FDD_509	65	7.62	[MRPL33]
N0816-A09-1_FDD_510	103	11.47	[MRPS36]
N0816-A11-1_FDD_510	113	12.55	[NDUFA7]
N0816-C09-2_FDD_510	103	11.46	[BBIP1]
N0816-F10-1_FDD_510	112	12.11	[MRPL53]
N0816-F12-1_FDD_510	123	14.08	[LSM10]
N0816-G03-1_FDD_510	76	8.73	[NDUFC1]
N0816-G05-1_FDD_510	88	10.06	[CENPW]
N0816-H05-1_FDD_510	90	10.39	[COX19]

0817-B09-1_FDD_511	201	23.6	[ORM2]
0817-C08-1_FDD_511	188	21.74	[IL32]
0817-E07-1_FDD_511	183	20.88	[SAP30L]
0817-E09-2_FDD_511	205	21.41	[ATP6V0B]
0817-F01-1_FDD_510	130	14.5	[MRPS17]
0817-F09-1_FDD_511	205	22.81	[MRPL21]
0817-F10-1_FDD_511	216	24.91	[MRPL24]
0817-H02-1_FDD_510	142	15.85	[MRPS18C]
0818-A01-1_FDD_511	253	29.84	[DNAJC8]
0818-A07-1_FDD_512	348	36.45	[CTU1]
0818-B01-1_FDD_511	254	27.72	[PAGR1]
0818-D01-1_FDD_511	256	28.53	[PSMA8]
0818-E05-1_FDD_512	333	35.08	[ETFA]
0818-G09-1_FDD_512	431	49.15	[ECSIT]
0818-H03-1_FDD_511	300	32.68	[TNFRSF6B]
0819-B08-1_FDD_513	96	10.92	[DYNLRB1]
0819-C07-1_FDD_514	741	83.54	[NUP88]
0819-D05-1_FDD_513	376	42.09	[CTSW]
0819-E05-1_FDD_513	644	70.21	[INPP5E]
0819-G08-1_FDD_513	262	29.85	[FITM2]
0819-G11-2_FDD_513	535	59.53	[SOCS6]
0819-H07-1_FDD_513	258	29.06	[EIF3J]
0820-A07-1_FDD_513	272	28.24	[EXOSC6]
0820-B01-1_FDD_513	635	66.86	[LRFN4]
0820-D03-1_FDD_514	973	111.41	[TRPC5]
0823-E08-2_FDD_206	384	44	[PROKR2]
0823-G04-4_FDD_206	372	41.96	[CXCR5]
0831-D05-1_FDD_211	1076	116.6	[LIPE]
0838-B11-1_FDD_221	347	38.45	[HP]
0841-C10-1_FDD_704	281	31.98	[WASF2]
0842-H10-1_FDD_704	500	55.02	[ENDOD1]
0843-E08-1_FDD_704	563	65.62	[CALD1]
0844-B12-1_FDD_704	631	71.69	[RAD21]
0846-C10-1_FDD_222	239	27.57	[DERL2]
0846-E01-1_FDD_222	130	14.76	[TRAPPC6B]
0846-F04-2_FDD_222	101	10.7	[PRPF40A]
0847-A08-1_FDD_223	1720	194.81	[POLR1A]
0847-B08-4_FDD_223	1572	175.74	[HECW1]
0847-C09-1_FDD_223	217	25.02	[CSH1]
0847-C10-1_FDD_223	896	103.17	[NUP107]
0847-D12-1_FDD_223	208	23.79	[RBSN]
0847-G04-1_FDD_223	254	28.39	[PDYN]
0848-A03-1_FDD_223	1329	152.99	[KTN1]
0848-A11-1_FDD_224	151	17.26	[CAV3]
0848-C12-3_FDD_210	853	94.81	[AKAP3]
0848-G06-1_FDD_224	1051	116.61	[ITGA3]
0849-A02-1_FDD_224	199	22.68	[PLCB2]
0849-C08-1_FDD_225	336	38.46	[DOCK2]

N0849-H01-1_FDD_224	165	18.67	[LY6K]
N0850-F10-1_FDD_226	311	34.21	[SRRM2]
N0850-G06-1_FDD_225	314	35.39	[SFRP1]
N0851-B07-1_FDD_226	120	13.56	[BRWD1]
N0851-C08-1_FDD_226	1336	135.51	[COL18A1]
N0851-G02-1_FDD_226	221	25.13	[IFIH1]
N0851-H03-1_FDD_226	356	41.02	[IL1RAP]
N0851-H08-1_FDD_227	566	63.64	[POLR2A]
N0852-C04-1_FDD_227	835	93	[MSH5]
N0852-H01-1_FDD_227	530	59.2	[PCM1]
N0853-A01-1_FDD_228	273	30.82	[HLA-DOB]
N0853-B06-1_FDD_228	325	36.91	[MRPL1]
N0853-C02-1_FDD_228	405	46.27	[ERCC2]
N0853-D07-1_FDD_228	331	36.92	[RABGGTB]
N0853-F03-1_FDD_701	963	104.9	[PACS1]
N0853-G02-1_FDD_228	144	16.7	[NDUFA13]
N0853-G08-1_FDD_228	916	103.7	[CHERP]
N0854-C05-1_FDD_229	122	13.64	[CC2D2A]
N0854-E03-1_FDD_229	967	105.81	[NUP210]
N0854-F01-1_FDD_229	276	32.17	[TRAPPC10]
N0856-G01-1_FDD_233	311	36.22	[PAQR3]
N0857-G05-1_FDD_309	1203	137.06	[CGN]
N0858-G03-1_FDD_234	840	96.36	[ATP6V0A4]
N0859-B01-1_FDD_310	184	20.04	[COPRS]
N0860-C06-1_FDD_236	1462	165.19	[IFT140]
N0860-G10-1_FDD_236	481	52.99	[SIGLEC16]
N0863-A03-1_FDD_237	793	89.45	[KIF3C]
N0863-C07-1_FDD_311	515	56.11	[CTU2]
N0870-A07-1_FDD_515	368	40.97	[RAE1]
N0870-F07-1_FDD_515	396	43.97	[SIGLEC14]
N0870-G07-1_FDD_515	398	45.57	[DAP3]
N0870-H12-1_FDD_515	726	82.79	[KIF3A]
N0871-A06-1_FDD_516	118	12.94	[GAGE3]
N0871-C10-2_FDD_518	192	21.5	[LCN12]
N0871-D01-1_FDD_514	790	88.31	[CDH6]
N0871-G11-1_FDD_515	210	23.95	[KCNMB4]
N0871-H06-1_FDD_516	142	16.31	[POLR2D]
N0872-F07-1_FDD_311	365	41.51	[GTF3A]
N0872-G01-1_FDD_516	231	26.16	[KLRC2]
N0872-G04-1_FDD_516	306	32.75	[PABPN1]
N0872-H01-2_FDD_517	235	25.28	[EXOSC5]
N0873-B04-1_FDD_516	542	59.72	[CPNE1]
N0873-H09-1_FDD_516	731	82.98	[GOLGA5]
N0874-A08-1_FDD_514	306	35.46	[DOK5]
N0874-A09-1_FDD_514	599	67.31	[ABCE1]
N0874-E03-1_FDD_515	1474	163.28	[A2M]
N0876-F05-1_FDD_520	81	9.07	[NEDD8]
N0876-F07-1_FDD_520	113	12.44	[NPFF]

N0877-A01-1_FDD_521(	189	21.01	[OAZ2]
N0877-D03-1_FDD_521(	228	25.41	[OAZ1]
N0877-E03-1_FDD_519!	228	23.41	[NDUFA11]
N0877-F03-1_FDD_519(	232	26.45	[RNF125]
N0878-C03-1_FDD_241!	393	44.77	[PROKR1]
N0878-E10-1_FDD_519!	489	53.16	[LILRA1]
N0878-F03-1_FDD_520	398	46.42	[CATSPER3]
N0881-F08-1_FDD_241:	1040	113.39	[CNTN2]
N0882-A11-1_FDD_519(	1125	116.64	[COL28A1]
N0882-D04-1_FDD_312	1419	156.04	[USP19]
N0882-E08-1_FDD_518	358	38.67	[GPR20]
N0882-E11-3_FDD_240	1375	151.34	[NID2]
N0882-F04-1_FDD_519!	1485	171.3	[AQR]
N0882-G04-1_FDD_241	1522	170.68	[TOPBP1]
N0882-H02-1_FDD_519:	1257	140	[L1CAM]
N0882-H07-1_FDD_518!	175	20.11	[NDUFS4]
N0883-E02-1_FDD_519:	376	41.25	[NOXO1]
N0883-F12-1_FDD_314	716	80.46	[IKBKE]
N0884-C06-1_FDD_243	1114	125.03	[ARHGAP39]
N0884-E04-1_FDD_243	991	109.68	[SMG8]
N0884-G05-1_FDD_243	1090	119.57	[PPP1R13B]
N0885-E04-1_FDD_243:	1273	145.18	[SIN3A]
N0886-A11-3_FDD_513	285	32.99	[ISY1]
N0886-B08-1_FDD_226	1204	136.31	[XPO5]
N0886-C07-1_FDD_226	678	64.37	[COL9A1]
N0886-C12-1_FDD_513	309	35.82	[MNAT1]
N0886-D12-1_FDD_513	525	57.06	[ARSG]
N0886-E05-1_FDD_225!	386	43.26	[PPP1R12B]
N0886-F11-3_FDD_513:	242	27.01	[DKKL1]
N0886-G01-2_FDD_225	745	82.3	[FBXO30]
N0886-G04-4_FDD_225	522	58.99	[CASP10]
N0886-G08-3_FDD_227	249	26.52	[CHTOP]
N0886-G12-1_FDD_513	575	61.45	[APBA3]
N0887-B02-1_FDD_513!	376	40.88	[PARD6G]
N0887-G08-1_FDD_228	834	91.96	[MAGED1]
N0888-A04-1_FDD_523!	472	53.82	[CWC27]
N0888-C07-1_FDD_523(	209	24	[CLEC6A]
N0888-D05-1_FDD_523(	194	22.26	[CLEC2D]
N0888-E11-2_FDD_523	329	35.83	[RCE1]
N0888-F06-1_FDD_315	1073	119.87	[SEMA6D]
N0888-F12-1_FDD_245(	1066	122.57	[WDR60]
N0888-G01-1_FDD_523	1154	133.28	[JAK1]
N0888-H11-1_FDD_315	363	41.83	[RPP40]
N0889-B10-1_FDD_524:	1074	120.62	[SEMA5A]
N0890-A08-1_FDD_522	194	21.56	[CD300LD]
N0890-D05-1_FDD_522!	1230	138.62	[POLRMT]
N0890-G08-1_FDD_521	211	24.34	[SPP2]
N0891-A04-1_FDD_517!	291	33.58	[ZC3H8]

N0891-B11-1_FDD_517[illegible]	1157	129.55	[DHX37]
N0891-D06-1_FDD_516	178	19.96	[EDN2]
N0891-D07-4_FDD_512[illegible]	1000	110.45	[C2CD5]
N0891-E05-1_FDD_516[illegible]	522	55.97	[PLIN1]
N0892-F03-1_FDD_523[illegible]	1490	164.15	[CDK12]
N0892-H01-1_FDD_523[illegible]	1258	138.6	[INPPL1]
N0893-F01-1_FDD_524[illegible]	509	55.04	[SHB]
N0894-A02-1_FDD_523[illegible]	286	33.37	[ALKBH3]
N0894-A06-1_FDD_523[illegible]	769	85.63	[ITGB8]
N0894-G03-1_FDD_523	537	59.14	[LGI4]
N0895-C07-1_FDD_517[illegible]	456	50.71	[RRP8]
N0895-G06-1_FDD_228	347	35.93	[EMILIN1]
N0896-A12-1_FDD_225[illegible]	766	84.14	[KIRREL3]
N0899-C01-1_FDD_524[illegible]	189	21.94	[IFNA5]
100000017_F	537	59.98	[CERK]
100000090_F	717	80.31	[SUN2]
100000150_N	903	97.98	[CSF2RB]
100000217_F	476	52.57	[DGCR14]
100000233_F	473	50.71	[RTN4R]
100000248_F	605	68.23	[NCAPH2]
100000265_F	601	65.71	[HMGXB4]
100000379_N	931	98.89	[MKL1]
100000383_N	1960	226.53	[MYH9]
100000410_F	333	36.32	[RAB36]
100000426_F	266	29.75	[SCO2]
100000432_F	793	88.89	[SF3A1]
100000443_F	369	41.33	[ST13]
100000453_F	862	96.66	[TOP3B]
100000510_N	587	63.54	[RANGAP1]
100000544_N	427	47.48	[TCN2]
100000569_N	866	96.12	[IL17RA]
100000572_N	939	103.53	[AP1B1]
100001645_F	520	58.38	[FIP1L1]
100001716_F	362	41.52	[RNF32]
100001744_F	308	34.9	[PPT2]
100001768_F	837	91.78	[TELO2]
100001793_F	1148	129.05	[MYBPC1]
100001831_F	195	19.53	[MARCKSL1]
100002037_F	368	40.78	[HAUS7]
100002050_F	200	22.98	[HMGB3]
100002065_F	514	57.67	[DKC1]
100002149_F	546	61.89	[TXLNA]
100002211_F	828	93.86	[GPAM]
100002251_F	979	110.16	[USP37]
100002254_F	1222	134.33	[VCPIP1]
100002301_F	1086	113.97	[NNT]
100002310_F	660	74.61	[ACOX1]
100002326_F	1205	133.84	[ATP2B4]

100002331_F	675	77.25	[MPP5]
100002341_F	969	107.8	[EPHA5]
100002506_F	574	65.01	[SENP3]
100002535_F	905	102.19	[KIAA1524]
100002622_F	933	105.95	[PLD2]
100002693_F	372	41.62	[TRIOBP]
100003282_N	458	49.59	[KRT13]
100003311_N	533	60.19	[LCP2]
100003312_N	325	36.57	[AKR1A1]
100003351_N	429	48.77	[GLA]
100003363_N	472	51.65	[KRT14]
100003385_N	564	61.42	[DPYSL5]
100003412_N	87	9.77	[FCER1G]
100003436_N	521	57.08	[PNKP]
100003470_N	702	76.78	[CEACAM5]
100003523_N	715	80.32	[POLI]
100003641_N	355	40.75	[NDUFA10]
100003673_N	603	68.53	[PAPSS1]
100003744_N	493	54.55	[PEPD]
100003885_N	845	95.94	[ABCF1]
100003887_N	934	104.84	[C6]
100003889_N	936	104.74	[MSH4]
100003898_N	395	41.03	[TSC22D4]
100003912_N	370	42.4	[ATXN3]
100003937_N	473	51.27	[KRT16]
100003944_N	228	25.42	[CD9]
100004040_N	559	63.17	[C9]
100004048_N	732	81.22	[APEH]
100004234_N	655	76.06	[RALBP1]
100004299_N	534	61.05	[P4HA1]
100004345_N	729	81.44	[MARK3]
100004421_N	468	50.06	[TNFRSF10A]
100004518_N	51	6.41	[RPL39]
100004551_N	216	23.5	[RPL14]
100004573_N	297	33.58	[COPS6]
100004630_N	224	24.83	[CD300C]
100004717_N	1246	137.79	[SKIV2L]
100004725_N	410	44.96	[LAMP2]
100004840_N	460	50.84	[GFRA1]
100004849_N	515	59.26	[G6PD]
100004885_N	381	43.87	[ATP6V1C2]
100004909_N	176	19.73	[MPV17]
100004934_N	623	71.29	[ABCF2]
100005031_N	1202	135.64	[PPFIA1]
100005033_N	98	10.41	[KRTAP3-2]
100005091_N	767	88.2	[GOLGA1]
100005168_N	639	70.02	[HSPA2]
100005175_N	729	80.36	[BAP1]

100005194_N	140	14.46	[SNCA]
100005225_N	256	29.62	[SURF2]
100005246_N	424	48.08	[STAMBP]
100005274_N	521	57.81	[KPNA3]
100005280_N	271	29.27	[ICAM4]
100005321_N	379	41.69	[FLOT2]
100005325_N	316	35.68	[POLR3F]
100005354_N	478	54.42	[GLTSCR2]
100005375_N	541	61.18	[EHD4]
100005424_N	390	44.85	[SERPINB4]
100005454_N	201	22.84	[RAB9A]
100005569_N	650	73.95	[FAF1]
100005660_N	835	93.31	[USP5]
100005713_N	354	39.04	[SDCCAG3]
100005716_N	61	6.09	[MT1F]
100005837_N	370	40.69	[OPRL1]
100005864_N	445	48.18	[SCLY]
100005870_N	475	53.49	[ATXN10]
100005872_N	483	53.12	[SHMT1]
100005888_N	569	62.58	[EEFSEC]
100005895_N	310	32.52	[SDC1]
100005922_N	724	79.7	[COMP]
100005986_N	584	58.83	[KRT10]
100005992_N	350	38.96	[IZUMO1]
100006033_N	514	55.8	[IMPDH2]
100006054_N	314	35.86	[ATG3]
100006109_N	1443	160.88	[CPSF1]
100006121_N	895	97.54	[DAG1]
100006201_N	1464	139.01	[COL1A1]
100006210_N	81	8.85	[RPS21]
100006232_N	903	97.36	[AKAP1]
100006251_N	1269	144.75	[FLII]
100006288_N	580	65.62	[GPC3]
100006297_N	412	43.91	[CBX6]
100006306_N	1120	124.39	[NFX1]
100006370_N	1214	132.88	[SMARCC2]
100006403_N	503	56.13	[UHRF2]
100006419_N	103	11.6	[LSM7]
100006467_N	917	102.38	[HK2]
100006477_N	371	40.49	[COQ2]
100006526_N	152	17.14	[UBE2N]
100006530_N	629	70.32	[PLS1]
100006560_N	217	22.35	[GAR1]
100006571_N	755	85.85	[HTATSF1]
100006579_N	1058	117.85	[UBA1]
100008637_F	745	82.94	[ABCD1]
100008999_F	333	36.05	[GIPC1]
100009136_F	890	98.7	[LPIN1]

100009887_F	298	32.24	[ISOC1]
100010672_F	125	13.51	[LAMTOR2]
100014383_F	505	57.46	[ASIC5]
100014441_F	339	38.95	[USP50]
100014471_F	188	21.59	[SSX7]
100014585_F	1706	194.11	[XRN1]
100014724_F	926	104.75	[GPRC6A]
100014736_F	727	81.32	[MTIF2]
100014738_F	1005	111	[EPHA8]
100014786_N	394	44.11	[POTEE]
100014835_N	121	13.02	[SCT]
100014859_N	419	45.12	[PYGO1]
100014915_N	251	28.1	[ANGPTL8]
100014971_N	180	19.92	[SPANXN2]
100015033_N	189	21.71	[IFNA16]
100015157_F	2352	246.75	[ANKRD17]
100015183_F	986	109.87	[EPHB2]
100015195_F	900	100.31	[POLN]
100015198_F	1655	177.74	[SCRIB]
100015293_F	1328	146.68	[SYNGAP1]
100015317_F	833	91.3	[MAP4K1]
100015352_F	1170	128.49	[ANKFY1]
100015375_F	2223	251.74	[SPAG17]
100015431_F	562	63.53	[ARSH]
100015523_F	70	7.54	[DEC1]
100015600_F	1568	175.74	[PLXNC1]
100015769_N	754	86.45	[RXFP2]
100015798_N	288	32.2	[POTEH]
100015816_N	213	23.88	[EFNA2]
100015843_N	559	62.06	[MLLT1]
100015876_N	419	42.7	[POU4F1]
100015923_N	343	36.1	[HOXD13]
100015951_F	1040	115.28	[NOD2]
100015981_F	132	15.09	[FABP9]
100016057_F	560	60.96	[GP5]
100016154_F	616	66.03	[TNFRSF11A]
100016177_F	1544	173.23	[ARHGEF12]
100016192_F	1447	160.54	[PTCH1]
100016213_F	1989	226.36	[ZFC3H1]
100016218_F	348	38.49	[KIR2DL2]
100016255_N	1049	113.75	[DSG1]
100016312_N	1256	145.52	[CSPP1]
100016320_N	1441	163.82	[RSF1]
100016365_N	498	56.17	[POTEA]
100016494_N	215	24.57	[IL19]
100058765_F	405	45.12	[SAG]
100058836_F	1471	166.46	[FANCD2]
100058866_F	538	56.05	[SAMD1]

100058888_F	669	70.03	[SYN1]
100058897_F	1038	114.57	[TNK2]
100058920_F	572	60.46	[ADRA1D]
100058941_F	764	86.84	[TSHR]
100058943_F	848	100.45	[CRNKL1]
100058946_F	1388	141.72	[COL15A1]
100061537_F	2602	278.16	[FLNB]
100061556_F	774	86.03	[HCN3]
100061574_F	1241	134.74	[NPHS1]
100061593_F	1017	115.01	[PHF20L1]
100061612_F	1835	197.39	[ARID2]
100061660_F	1575	171.23	[LAMC3]
100061668_F	504	52.32	[SLC16A8]
100061686_F	530	59.6	[USP17L2]
100061742_F	763	85.58	[DYRK1A]
100061765_F	699	78.64	[LHCGR]
100061766_F	674	70.44	[DMWD]
100061776_F	2288	259.84	[ATRX]
100061779_F	444	49.78	[TUBB8]
100061800_F	1203	129.04	[HCN4]
100061825_F	1938	223.6	[MYH13]
100061836_F	1621	182.66	[TOP2B]
100061853_F	1690	175.96	[TNRC6C]
100061860_F	1041	115.96	[IPO9]
100061863_F	1531	171.59	[ABCC1]
100061875_F	251	27.73	[VPS37D]
100061912_F	25	3.46	[RPL41]
100061956_F	2363	267.29	[TPR]
100062004_F	1855	215.4	[MYO5A]
100062016_N	156	16.6	[PSPN]
100062223_N	847	97.78	[VAV3]
100062246_N	1251	139.68	[CNGB1]
100062330_F	2266	252.5	[AHCTF1]
100062341_F	837	92.86	[APBA1]
100062342_F	1564	184.87	[ZC3H13]
100062345_F	424	46.97	[AVPR1B]
100062366_F	1309	146.67	[CLUH]
100062383_F	1161	126.76	[ITGAD]
100062384_F	1537	164.86	[DOT1L]
100062394_F	434	46.35	[GPR150]
100062400_F	628	64.42	[KRT3]
100062437_F	1901	210.51	[AKAP11]
100062448_F	1873	208.55	[PLXNA1]
100062473_F	1670	161.81	[COL4A3]
100062592_F	465	49.16	[E2F3]
100062605_F	2113	230.63	[DSCAML1]
100062664_F	1871	207.7	[PLXNA3]
100062694_N	1320	151	[MAP4K4]

100062715_N	1460	164.31	[CEP164]
100062738_N	1215	133.74	[HIPK3]
100062765_N	686	75.7	[MASP2]
100062768_N	425	46.45	[SHC2]
100062814_N	535	60.89	[EHD3]
100063578_N	391	42.15	[PAX5]
100063579_N	1612	182	[MLLT4], [AFDN]
100063588_N	158	16.39	[CYS1]
100063957_N	861	97.1	[AGO4]
100063969_N	331	34.27	[TAL1]
100063997_N	304	33.7	[KIR2DS5]
100064002_N	348	39.03	[HPR]
100064128_N	1749	196.04	[NUP188]
100064147_N	109	11.83	[HMGA2]
100064161_N	375	39.82	[GPR27]
100064164_N	1384	156.27	[CNTNAP1]
100064165_N	358	37.56	[CEBPA]
100064170_N	2460	277.42	[USP24]
100064178_N	206	21.72	[GP1BB]
100064196_N	671	74.61	[SRP72]
100064202_N	575	66	[CNGA4]
100064204_N	568	64.93	[NOX3]
100064206_N	557	63.31	[CNOT6]
100066341_N	852	91.8	[NRG2]
100066343_N	558	57.26	[DACH1]
100066362_N	423	47.4	[GHRHR]
100066363_N	304	33.62	[KIR2DS1]
100066366_N	200	23.42	[GUCA1B]
100066368_N	1047	117.02	[NPR2]
100066371_N	754	84.26	[PIK3R6]
100066373_N	276	30.68	[NCR2]
100066374_N	112	12.62	[CCL27]
100066391_N	500	52.86	[NFKBIE]
100066395_N	1623	184.64	[RNF17]
100066403_N	1219	133.63	[FKBP15]
100066407_N	1640	187	[CLTCL1]
100066412_N	2430	268.84	[FAM208B]
100066427_N	584	66.39	[POTED]
100066429_N	1403	161.94	[CEP162]
100066454_N	531	59.9	[PRMT3]
100066455_N	536	59.83	[SRC]
100066480_N	540	57.95	[SNTB2]
100066500_N	1172	129.57	[LAMB3]
100066618_N	1286	148.58	[GIGYF2]
100066662_N	315	35.12	[C11orf54]
100066683_N	923	103.05	[NRP1]
100066713_F	1278	141.52	[DCTN1]
100066747_F	538	59.4	[CCDC8]

100066852_N	177	20.33	[IL25]
100066931_N	563	63.16	[RBM15B]
100067019_N	829	93.06	[MCC]
100067047_F	533	59.5	[FAN1]
100067056_F	413	47.6	[MSL1]
100067060_F	2176	239.55	[FN1]
100067141_N	370	41.57	[ARPC1A]
100067176_N	510	52	[WIPF1]
100067177_N	413	47.27	[ARL13B]
100067184_N	61	6.12	[MT1B]
100067200_N	501	55.41	[GDF5]
100067359_N	240	27.75	[ING5]
100067360_N	332	31.55	[MARCKS]
100067385_N	534	57.29	[KRT4]
100067528_N	198	22.41	[SAR1B]
100067644_N	333	37.06	[ANTXR1]
100067696_N	511	56.88	[BAIAP2L1]
100067700_N	184	20.88	[ARL2]
100067714_F	686	75.66	[TAP2]
100067787_N	362	38.42	[CCR10]
100067788_N	209	23.8	[GUCA1C]
100067792_N	261	28.87	[RPA4]
100067793_N	136	14.94	[QRFP]
100067832_N	824	87.88	[MAPK8IP2]
100068166_N	1085	110.11	[TAF4]
100068168_N	319	35.07	[GPR31]
100068169_N	1234	140.04	[KIF4B]
100068176_N	902	100.87	[GRIA4]
100068194_N	1092	124.11	[FMNL2]
100068201_N	842	92.93	[UNC5A]
100068202_N	762	84.7	[KSR1]
100068203_N	623	62.06	[KRT9]
100068204_N	1604	157.75	[COL16A1]
100068221_N	1064	115.39	[LLGL1]
100068225_N	609	64.89	[NUMBL]
100068230_N	1188	133.47	[ITGA11]
100068231_N	1074	116.55	[ADAMTSL4]
100068234_N	747	85.1	[HNRNPUL2]
100068243_N	1037	119.94	[IPO8]
100068244_N	1651	185.84	[ARHGEF28]
100068253_N	1256	136.92	[MAGI1]
100068254_N	911	100	[LEMD3]
100068264_N	1497	150.42	[COL17A1]
100068268_N	1254	134.93	[SHANK2]
100068271_N	980	109.26	[GRIK5]
100068276_N	1512	164.92	[CDK13]
100068548_N	916	102.48	[NEFM]
100068599_N	468	54.39	[ERO1A]

100068736_N	4563	515.53	[APOB]
100068739_N	3418	384.2	[BRCA2]
100068740_N	2912	314.77	[FBN2]
100068744_N	2016	226.94	[SCN5A]
100068746_N	1621	177.44	[NES]
100068930_F	368	39.57	[GALR3]
100068961_F	419	43.9	[DRD4]
100068962_F	391	39.49	[CACNG8]
100068963_F	3458	388.2	[RELN]
100068970_F	3396	372.82	[VCAN]
100068974_F	2414	264.16	[EP300]
100068983_F	2590	289.62	[POLQ]
100069010_N	677	74.89	[WDR43]
100069016_N	511	54.52	[MUC13]
100069042_N	1130	123.41	[CIITA]
100069050_N	2817	307.85	[AKAP13]
100069056_N	2214	247.75	[SVIL]
100069060_N	929	103.58	[TAF3]
100069063_N	1427	153.6	[ADAMTS13]
100069071_N	1744	192.79	[C4A]
100069073_N	2004	225.03	[KAT6A]
100069077_N	2701	308.06	[ITPR2]
100069078_N	1798	195.98	[LAMB2]
100069079_N	1940	223.9	[MYH3]
100069080_N	1937	222.76	[MYH8]
100069087_N	2472	274.47	[RIF1]
100069092_N	2044	231.32	[PI4KA]
100069094_N	242	24.11	[ZNRF2]
100069100_N	2671	304.11	[ITPR3]
100069114_N	1626	161.15	[COL22A1]
100069117_N	3130	352.78	[REV3L]
100069118_N	3116	350.86	[CEP350]
100069121_N	1872	212.68	[TAF1]
100069125_N	2017	223.69	[KIAA0368]
100069127_N	1469	159.43	[SNAPC4]
100069129_N	1909	206.85	[PLXNB3]
100069130_N	2286	261.52	[POLE]
100069131_N	2440	270.21	[NCOR1]
100069132_N	2390	271.29	[SPTBN2]
100069134_N	1838	205.13	[PLXNB2]
100069135_N	2346	265.08	[PDE4DIP]
100069136_N	1871	208.7	[PDCD11]
100069141_N	1341	152.67	[ANKRD30A]
100069143_N	3075	337.08	[LAMA1]
100069145_N	3051	330.47	[BOD1L1]
100069160_N	858	95.88	[KCNB1]
100069184_N	608	65.85	[CARM1]
100069191_N	2419	280.01	[SPTA1]

100069198_N	1411	162.47	[EEA1]
100069209_N	1455	162.78	[FANCA]
100069213_N	1113	125.3	[WWC1]
100069216_N	2027	231.43	[CIT]
100069224_N	890	98.8	[HCN1]
100069226_N	653	73.48	[CHM]
100069227_N	1641	186.84	[KDM5A]
100069236_N	1938	209.65	[TCF20]
100069241_N	1321	147.34	[KDM3A]
100069257_N	836	90.17	[DACT1]
100069258_N	3122	343.93	[LAMA2]
100069260_N	2479	290.39	[CEP290]
100069261_N	299	31.67	[GFRA4]
100069263_N	2458	276.54	[ACACB]
100069264_N	2120	233.18	[ESPL1]
100069268_N	781	83.78	[CDH24]
100069271_N	794	88.75	[SEL1L]
100069279_N	2146	241.65	[YLPM1]
100069898_F	215	21.84	[HIST1H1A]
100069946_F	226	22.58	[HIST1H1B]
100069951_F	373	39.95	[RASSF7]
100069963_F	376	41.51	[GTF2A1]
100070117_F	234	26.35	[NKX3-1]
100070120_F	345	38.43	[TFDP3]
100070198_F	668	71.36	[FAM189B]
100070237_F	434	48.33	[TEAD4]
100070354_F	158	17.42	[BTG2]
100070362_F	843	92.53	[OTUD7B]
100070363_F	955	108.67	[THRAP3]
100071263_F	111	11.99	[KIAA0101]
100071301_F	199	22.47	[TAGLN3]
100071308_F	68	7.21	[GNG10]
100071323_F	123	14.11	[TEX12]
100071812_F	132	15.21	[FABP2]
100071822_F	377	41.49	[KIR2DL4]
100071824_F	134	15.71	[RBP2]
100071831_F	152	16.28	[LAIR2]
100071848_F	138	15.61	[TSHB]
100071850_F	169	16.28	[KRTAP5-9]
100071851_F	189	21.7	[IFNA13]
100071853_F	265	29.92	[CER1]
100071858_F	94	10.07	[DEFA5]
100071882_F	100	10.98	[DEFA6]
100071894_F	101	11.39	[GAST]
100072244_F	550	62.24	[PTH2R]
100072259_F	130	14.37	[PCBD2]
100072272_F	244	27.32	[CEP104]
100072306_F	816	88.39	[MAPK7]

100072309_F	823	90.78	[COG5]
100072311_F	92	10.58	[S100A12]
100072333_F	62	6.42	[MT4]
100072342_F	99	10.74	[LAMTOR4]
100072346_F	100	10.96	[BGLAP]
100072366_F	115	12.89	[MLN]
100072371_F	142	16.22	[LALBA]
100072379_F	150	16.61	[CCL25]
100072394_F	200	21.9	[IFNL1]
100072404_F	196	21.71	[IFNL3]
100072423_F	212	23.89	[ROPN1B]
100072425_F	221	24.94	[RAB41]
100072437_F	254	27.71	[ODF3]
100072447_F	265	29.8	[CHMP4A]
100072451_F	273	30.9	[PDCD1LG2]
100072505_F	349	37.39	[THPO]
100072509_F	333	37.65	[CD1C]
100072515_F	398	45.08	[TRHR]
100072529_F	395	45.25	[TRIM10]
100072539_F	365	40.32	[WNT9A]
100072556_F	431	48.14	[KRT40]
100072590_F	510	57.28	[OLFM4]
100072620_F	585	65.41	[GAD2]
100072637_F	584	65.16	[C8A]
100072644_F	674	77.98	[ALOX5]
100072645_F	636	71.39	[KCND3]
100072656_F	692	74.81	[NEK8]
100072662_F	655	71.08	[SMPD3]
100072664_F	676	72.19	[LIMD1]
100072689_F	824	93.32	[GRIA3]
100072690_F	837	92.76	[PKP2]
100072698_F	905	101.18	[STON2]
100072704_F	872	95.57	[GRM2]
100072718_F	1153	131.12	[NOS2]
100072719_F	1179	130.09	[ITGAE]
100072720_F	1233	143.23	[SMC1A]
100072721_F	1686	190.68	[PIK3C2A]
100072725_F	99	11.2	[CCL7]
100072769_F	176	20.07	[IL20]
100072775_F	185	21.04	[RLN2]
100072784_F	237	27.49	[CLEC4A]
100072807_F	350	39.37	[MTNR1A]
100072828_F	396	45.4	[APOA4]
100072871_F	496	56.53	[ADCYAP1R1]
100072873_F	488	53.12	[PTGER4]
100072900_F	593	66.36	[PTH1R]
100072908_F	697	78.28	[ZBTB24]
100072946_F	815	86.92	[ATXN1]

100072961_F	1137	127.29	[AP4E1]
100072965_F	1141	128.07	[MYBPC2]
100072966_F	1173	133.7	[PLCB1]
100072967_F	1249	140.88	[BRIP1]
100072968_F	1287	142.95	[STIL]
100072974_F	1648	186.47	[KIF14]
100072978_F	609	68.93	[KMT2E]
100072982_F	703	80.96	[GYS2]
100073061_F	242	27.54	[FTMT]
100073099_F	417	45.38	[TNFRSF25]
100073116_F	676	72.64	[AMOT]
100073141_F	235	26.42	[FLT3LG]
100073173_F	514	55.59	[TBL1XR1]
100073196_F	1429	161.04	[MLH3]
100073936_F	363	39.61	[RAD23A]
100073963_F	600	69.08	[RUFY1]
100073969_F	120	12.82	[LAMA4]
100073980_F	197	21.05	[SFTPC]
40080549_N	715	81.04	[EPX]
40080550_N	421	45.85	[ACR]
40080558_N	482	53.03	[RCOR1]
40080561_N	67	7.7	[DEFB103B]
40080564_N	184	18.86	[TNFRSF13C]
40080646_F	1251	140.12	[ADCY8]
40080651_F	1122	125.77	[PPP1R3A]
40080652_F	437	48.17	[SPAG4]
40080653_F	264	30.41	[SPI1]
40080655_F	539	56.98	[CSPG5]
40080741_F	131	13.36	[HCRT]
40080743_F	769	83.42	[ADAM11]
40080749_F	317	35.15	[IBSP]
40080755_F	506	56.15	[NOS1AP]
40080840_F	1194	132.36	[GRM1]
40080841_F	372	40.37	[OPRD1]
40080842_F	889	96.95	[HCN2]
40080846_F	1321	143.09	[NCAN]
40080850_F	694	73.3	[FZD8]
