# Supplementary material for: Autoantibody Landscape Revealed by Wet Protein Array: Sum of Autoantibody Levels Reflects Disease Status
Source: Front Immunol. 2022 May 4;13:893086. doi: 10.3389/fimmu.2022.893086 (PMC9114879; doi:10.3389/fimmu.2022.893086)
Supplement: Supplementary file 4 [file Table_3.pdf]

**Supplementary Table 3. Targets of autoantibodies detected in proteome-wide wet protein array in malignant melanoma.**

| Gene Symbol | localized | advanced |
|---|---|---|
| TBC1D4 | 5 | 6 |
| CIZ1 | 0 | 6 |
| CTAG1B | 0 | 6 |
| CTAG2 | 0 | 6 |
| KCTD17 | 0 | 6 |
| KCTD5 | 0 | 6 |
| KHDRBS2 | 0 | 6 |
| KHDRBS3 | 0 | 6 |
| LONP2 | 0 | 6 |
| TP63 | 0 | 6 |
| TP73 | 0 | 6 |
| CEP85 | 6 | 5 |
| RBPJ | 5 | 5 |
| KAT2A | 4 | 5 |
| TRIM21 | 3 | 5 |
| PDLIM5 | 2 | 5 |
| SOX14 | 2 | 5 |
| SOX2 | 2 | 5 |
| TRIM21(1-400) | 2 | 5 |
| IER2 | 0 | 5 |
| KHDRBS1 | 0 | 5 |
| MEN1 | 0 | 5 |
| TOX2 | 0 | 5 |
| CCNI2 | 4 | 4 |
| LIMS1 | 3 | 4 |
| PPP2R5D | 3 | 4 |
| SPAG8 | 3 | 4 |
| TROVE2 | 3 | 4 |
| C15orf43 | 2 | 4 |
| SIGLECL1 | 2 | 4 |
| SLCO4C1 | 2 | 4 |
| Sox1 | 2 | 4 |
| BAIAP2 | 0 | 4 |
| CCDC86 | 0 | 4 |
| CDC20 | 0 | 4 |
| DBT | 0 | 4 |
| DNAI1 | 0 | 4 |
| GIMAP1 | 0 | 4 |
| JUND | 0 | 4 |
| RASSF4 | 0 | 4 |
| RGS6 | 0 | 4 |

| | | |
|---|---|---|
| TMEM40 | 0 | 4 |
| TRPV2 | 0 | 4 |
| VCY, VCY1B | 0 | 4 |
| GATAD1 | 4 | 3 |
| HMX2 | 4 | 3 |
| MGC39545 | 4 | 3 |
| ZNF688 | 4 | 3 |
| ACVR2B | 3 | 3 |
| BCL2L11 | 3 | 3 |
| PAK1 | 3 | 3 |
| AGAP3 | 2 | 3 |
| BMP2K | 2 | 3 |
| CCDC136 | 2 | 3 |
| FAM83F | 2 | 3 |
| GPRC5A | 2 | 3 |
| IL21R | 2 | 3 |
| PRRT2 | 2 | 3 |
| SREBF1 | 2 | 3 |
| ATRIP | 0 | 3 |
| BARX1 | 0 | 3 |
| BOP1 | 0 | 3 |
| C12orf43 | 0 | 3 |
| C5orf22 | 0 | 3 |
| C8orf59 | 0 | 3 |
| CBFA2T3 | 0 | 3 |
| CDC7 | 0 | 3 |
| CDK5 | 0 | 3 |
| CHRNA9 | 0 | 3 |
| COL10A1 | 0 | 3 |
| CPXM2 | 0 | 3 |
| CRNN | 0 | 3 |
| DDX53 | 0 | 3 |
| DOK2 | 0 | 3 |
| EDC4 | 0 | 3 |
| EID3 | 0 | 3 |
| ENAH | 0 | 3 |
| EXD3 | 0 | 3 |
| FAN1 | 0 | 3 |
| FKBPL | 0 | 3 |
| GATA4 | 0 | 3 |
| HES5 | 0 | 3 |
| HMBOX1 | 0 | 3 |
| IRX2 | 0 | 3 |
| KIF9 | 0 | 3 |
| LCOR | 0 | 3 |

| | | |
|---|---|---|
| LETM1 | 0 | 3 |
| LIG1 | 0 | 3 |
| MKRN1 | 0 | 3 |
| MRPL48 | 0 | 3 |
| MXRA8 | 0 | 3 |
| MYH7B | 0 | 3 |
| NDC80 | 0 | 3 |
| NPTN | 0 | 3 |
| OTUD4 | 0 | 3 |
| PALLD | 0 | 3 |
| PDE1C | 0 | 3 |
| POLH | 0 | 3 |
| POLR3C | 0 | 3 |
| POMC | 0 | 3 |
| PPAN | 0 | 3 |
| PPP1R15A | 0 | 3 |
| PRKAB2 | 0 | 3 |
| PRRC2B | 0 | 3 |
| PTGS2 | 0 | 3 |
| PTPN2 | 0 | 3 |
| RABL6 | 0 | 3 |
| RNF180 | 0 | 3 |
| SAFB | 0 | 3 |
| SOX3 | 0 | 3 |
| SPACA7 | 0 | 3 |
| SPRED2 | 0 | 3 |
| STK25 | 0 | 3 |
| STX2 | 0 | 3 |
| TMEM31 | 0 | 3 |
| TPD52 | 0 | 3 |
| TRIB1 | 0 | 3 |
| TSNARE1 | 0 | 3 |
| WBP1L | 0 | 3 |
| XIRP2 | 0 | 3 |
| ZCCHC5 | 0 | 3 |
| ZNF177 | 0 | 3 |
| ZNF329 | 0 | 3 |
| CD320 | 4 | 2 |
| CDKN1C | 4 | 2 |
| FAM161B | 4 | 2 |
| IL1A | 4 | 2 |
| RBM23 | 4 | 2 |
| RPP21 | 4 | 2 |
| TWIST1 | 4 | 2 |
| C2orf57 | 3 | 2 |

| | | |
|---|---|---|
| CPLX1 | 3 | 2 |
| CRTC2 | 3 | 2 |
| LBX1 | 3 | 2 |
| MAP7D2 | 3 | 2 |
| MEF2D | 3 | 2 |
| NAP1L3 | 3 | 2 |
| NLRP1 | 3 | 2 |
| PHC1 | 3 | 2 |
| SAP130 | 3 | 2 |
| SLC19A1 | 3 | 2 |
| SOX5 | 3 | 2 |
| SPSB2 | 3 | 2 |
| SUN1 | 3 | 2 |
| ACVR2A | 2 | 2 |
| APEX2 | 2 | 2 |
| CCNE1 | 2 | 2 |
| CDR2 | 2 | 2 |
| CMTM2 | 2 | 2 |
| ETV6 | 2 | 2 |
| JAKMIP1 | 2 | 2 |
| MAGEH1 | 2 | 2 |
| MRPS31 | 2 | 2 |
| NOP2 | 2 | 2 |
| PHLDA2 | 2 | 2 |
| RBFOX2 | 2 | 2 |
| RMDN3 | 2 | 2 |
| SERTAD2 | 2 | 2 |
| SMG9 | 2 | 2 |
| TBC1D22B | 2 | 2 |
| TMEM183A | 2 | 2 |
| VRK3 | 2 | 2 |
| ZBTB32 | 2 | 2 |
| ABCA11P | 0 | 2 |
| ABCF3 | 0 | 2 |
| ALKBH5 | 0 | 2 |
| ALS2CR12 | 0 | 2 |
| ALX4 | 0 | 2 |
| ANK1 | 0 | 2 |
| ANKRD53 | 0 | 2 |
| ARC | 0 | 2 |
| ARFGAP2 | 0 | 2 |
| ARMC2 | 0 | 2 |
| ATF6 | 0 | 2 |
| ATXN7L3 | 0 | 2 |
| B9D1 | 0 | 2 |

| | | |
|---|---|---|
| BARD1 | 0 | 2 |
| BECN1 | 0 | 2 |
| BORA | 0 | 2 |
| BSCL2 | 0 | 2 |
| C10orf71 | 0 | 2 |
| C11orf1 | 0 | 2 |
| C11orf63 | 0 | 2 |
| C14orf177 | 0 | 2 |
| C1orf168 | 0 | 2 |
| C20orf96 | 0 | 2 |
| C2orf44 | 0 | 2 |
| C8orf48 | 0 | 2 |
| CCDC102B | 0 | 2 |
| CCDC121 | 0 | 2 |
| CCDC74B | 0 | 2 |
| CCDC94 | 0 | 2 |
| CDCA8 | 0 | 2 |
| CDK5R1 | 0 | 2 |
| CDK5RAP2 | 0 | 2 |
| CEP128 | 0 | 2 |
| CHST12 | 0 | 2 |
| CORO6 | 0 | 2 |
| CTAGE5 | 0 | 2 |
| DCD | 0 | 2 |
| DCTN4 | 0 | 2 |
| DDX17 | 0 | 2 |
| DDX41 | 0 | 2 |
| DKK3 | 0 | 2 |
| EAPP | 0 | 2 |
| EDA2R | 0 | 2 |
| EFS | 0 | 2 |
| ENOSF1 | 0 | 2 |
| EPB41L3 | 0 | 2 |
| EPHA6 | 0 | 2 |
| ETV3 | 0 | 2 |
| FAM184A | 0 | 2 |
| FAM47E | 0 | 2 |
| FBXO28 | 0 | 2 |
| FILIP1L | 0 | 2 |
| FOXD4L1 | 0 | 2 |
| FRG1 | 0 | 2 |
| FUT10 | 0 | 2 |
| GABRA1 | 0 | 2 |
| GATAD2A | 0 | 2 |
| GCH1 | 0 | 2 |

| | | |
|---|---|---|
| GJD4 | 0 | 2 |
| GRHL2 | 0 | 2 |
| GRIPAP1 | 0 | 2 |
| GTF3C5 | 0 | 2 |
| HES7 | 0 | 2 |
| IGF2 | 0 | 2 |
| IGSF6 | 0 | 2 |
| IMP4 | 0 | 2 |
| IRX5 | 0 | 2 |
| ISX | 0 | 2 |
| JADE2 | 0 | 2 |
| KCNK2 | 0 | 2 |
| KDM1B | 0 | 2 |
| KIAA0100 | 0 | 2 |
| KIF22 | 0 | 2 |
| KLF5 | 0 | 2 |
| LAD1 | 0 | 2 |
| LAMP3 | 0 | 2 |
| LDLRAD4 | 0 | 2 |
| LOC100131831 | 0 | 2 |
| LOC100506127 | 0 | 2 |
| LSM14B | 0 | 2 |
| MAFG | 0 | 2 |
| MAGEA2 | 0 | 2 |
| MAGIX | 0 | 2 |
| MAP2K6 | 0 | 2 |
| MAP3K7 | 0 | 2 |
| MAP4K5 | 0 | 2 |
| MARVELD2 | 0 | 2 |
| MBIP | 0 | 2 |
| MEPCE | 0 | 2 |
| METTL14 | 0 | 2 |
| MLANA | 0 | 2 |
| MRFAP1 | 0 | 2 |
| MRPL16 | 0 | 2 |
| MSANTD2 | 0 | 2 |
| MTUS1 | 0 | 2 |
| MYO1A | 0 | 2 |
| MYSM1 | 0 | 2 |
| NDUFAF4 | 0 | 2 |
| NEDD9 | 0 | 2 |
| NEK5 | 0 | 2 |
| NKX3-1 | 0 | 2 |
| NPAS2 | 0 | 2 |
| NRXN3 | 0 | 2 |

| | | |
|---|---|---|
| NUAK1 | 0 | 2 |
| NUAK2 | 0 | 2 |
| NUF2 | 0 | 2 |
| ORAI2 | 0 | 2 |
| OSBPL5 | 0 | 2 |
| PERM1 | 0 | 2 |
| PEX5L | 0 | 2 |
| PLAGL2 | 0 | 2 |
| POU3F4 | 0 | 2 |
| PPFIA2 | 0 | 2 |
| PPM1E | 0 | 2 |
| PPP2R3B | 0 | 2 |
| PPP2R5E | 0 | 2 |
| PRKCDBP | 0 | 2 |
| PRKG2 | 0 | 2 |
| PRKRIP1 | 0 | 2 |
| RAP1GAP | 0 | 2 |
| RELL2 | 0 | 2 |
| REM1 | 0 | 2 |
| RFX3 | 0 | 2 |
| RFX4 | 0 | 2 |
| RILPL1 | 0 | 2 |
| RPH3A | 0 | 2 |
| RSBN1 | 0 | 2 |
| RTN3 | 0 | 2 |
| SAMD8 | 0 | 2 |
| SH3GL1 | 0 | 2 |
| SH3GL2 | 0 | 2 |
| SLC23A2 | 0 | 2 |
| SLC39A10 | 0 | 2 |
| SMTNL2 | 0 | 2 |
| SNAP29 | 0 | 2 |
| SNX15 | 0 | 2 |
| SNX7 | 0 | 2 |
| SPIN1 | 0 | 2 |
| SSB | 0 | 2 |
| SSTR5 | 0 | 2 |
| SV2B | 0 | 2 |
| SWAP70 | 0 | 2 |
| TAF6L | 0 | 2 |
| TBC1D1 | 0 | 2 |
| TBCA | 0 | 2 |
| TBX20 | 0 | 2 |
| TCEANC2 | 0 | 2 |
| TCOF1 | 0 | 2 |

| | | |
|---|---|---|
| TDP1 | 0 | 2 |
| TESK2 | 0 | 2 |
| THAP3 | 0 | 2 |
| THEG | 0 | 2 |
| TJP3 | 0 | 2 |
| TLDC2 | 0 | 2 |
| TMEM214 | 0 | 2 |
| TMEM255B | 0 | 2 |
| TOX3 | 0 | 2 |
| TXNDC2 | 0 | 2 |
| UBE2E1 | 0 | 2 |
| UBE2J2 | 0 | 2 |
| UBXN1 | 0 | 2 |
| VIPAS39 | 0 | 2 |
| ZBTB39 | 0 | 2 |
| ZC3H8 | 0 | 2 |
| ZG16B | 0 | 2 |
| ZMIZ2 | 0 | 2 |
| ZNF148 | 0 | 2 |
| ZNF26 | 0 | 2 |
| ZNF408 | 0 | 2 |
| ZNF543 | 0 | 2 |
| ZNF550 | 0 | 2 |
| ZNF599 | 0 | 2 |
| ZSCAN20 | 0 | 2 |
| GPRASP2 | 5 | 0 |
| P3H4 | 5 | 0 |
| RRP9 | 5 | 0 |
| SYNPO2 | 5 | 0 |
| BANK1 | 4 | 0 |
| BARX2 | 4 | 0 |
| CCDC15 | 4 | 0 |
| CCNB2 | 4 | 0 |
| NT5C1A | 4 | 0 |
| PSIP1 | 4 | 0 |
| STX1A | 4 | 0 |
| TFAP2A | 4 | 0 |
| C1orf127 | 3 | 0 |
| CAMLG | 3 | 0 |
| COL9A2 | 3 | 0 |
| EPB41L1 | 3 | 0 |
| FAM220A | 3 | 0 |
| IFI44 | 3 | 0 |
| ING2 | 3 | 0 |
| KAT2B | 3 | 0 |

| | | |
|---|---|---|
| KIAA1958 | 3 | 0 |
| KLHL7 | 3 | 0 |
| LENG8 | 3 | 0 |
| LRRC71 | 3 | 0 |
| MACC1 | 3 | 0 |
| MAGEB18 | 3 | 0 |
| METTL22 | 3 | 0 |
| MRPL12 | 3 | 0 |
| PASD1 | 3 | 0 |
| PHACTR4 | 3 | 0 |
| PITX1 | 3 | 0 |
| PPP1R3F | 3 | 0 |
| RARRES1 | 3 | 0 |
| RHOU | 3 | 0 |
| SSH1 | 3 | 0 |
| TFAP2B | 3 | 0 |
| TMEM215 | 3 | 0 |
| TTBK1 | 3 | 0 |
| UNK | 3 | 0 |
| URI1 | 3 | 0 |
| VPS72 | 3 | 0 |
| ZEB1 | 3 | 0 |
| ZNF330 | 3 | 0 |
| ACVR1C | 2 | 0 |
| ANKS1B | 2 | 0 |
| ARHGAP27 | 2 | 0 |
| ARHGAP9 | 2 | 0 |
| ARMCX1 | 2 | 0 |
| ATP4A | 2 | 0 |
| BAG5 | 2 | 0 |
| BNC1 | 2 | 0 |
| BNIPL | 2 | 0 |
| C10orf131 | 2 | 0 |
| C1QL1 | 2 | 0 |
| C1QTNF2 | 2 | 0 |
| C9orf43 | 2 | 0 |
| CCDC155 | 2 | 0 |
| CCDC96 | 2 | 0 |
| CCL28 | 2 | 0 |
| CCSAP | 2 | 0 |
| CDC40 | 2 | 0 |
| CDK19 | 2 | 0 |
| CELF2 | 2 | 0 |
| CFHR1 | 2 | 0 |
| CHRM2 | 2 | 0 |

| | | |
|---|---|---|
| CIART | 2 | 0 |
| CNGA3 | 2 | 0 |
| CPLX2 | 2 | 0 |
| CRTC1 | 2 | 0 |
| CXorf67 | 2 | 0 |
| DNAJC12 | 2 | 0 |
| EIF3F | 2 | 0 |
| ETNK1 | 2 | 0 |
| FAM177A1 | 2 | 0 |
| FAM219A | 2 | 0 |
| FAM83G | 2 | 0 |
| FATE1 | 2 | 0 |
| FOXK2 | 2 | 0 |
| FOXL2 | 2 | 0 |
| GFAP | 2 | 0 |
| GNN | 2 | 0 |
| GOLGA6L2 | 2 | 0 |
| GPRIN1 | 2 | 0 |
| HAGHL | 2 | 0 |
| HEATR6 | 2 | 0 |
| HECTD2 | 2 | 0 |
| HOMER3 | 2 | 0 |
| HTR1A | 2 | 0 |
| IDH1 | 2 | 0 |
| IGF1 | 2 | 0 |
| ING1 | 2 | 0 |
| KAT7 | 2 | 0 |
| KCNMB3 | 2 | 0 |
| KDM2A | 2 | 0 |
| KIAA0753 | 2 | 0 |
| KIF3B | 2 | 0 |
| KLHDC10 | 2 | 0 |
| LARP4 | 2 | 0 |
| LDB3 | 2 | 0 |
| LINC00482 | 2 | 0 |
| LPXN | 2 | 0 |
| LRRC37A8P | 2 | 0 |
| MAGEA1 | 2 | 0 |
| MAGEB2 | 2 | 0 |
| MAGEB6 | 2 | 0 |
| MAPRE2 | 2 | 0 |
| MARCO | 2 | 0 |
| MB21D1 | 2 | 0 |
| MFAP1 | 2 | 0 |
| MNT | 2 | 0 |

| | | |
|---|---|---|
| MRPL45 | 2 | 0 |
| NDUFV3 | 2 | 0 |
| NFIB | 2 | 0 |
| OLIG1 | 2 | 0 |
| PAQR9 | 2 | 0 |
| PHF13 | 2 | 0 |
| PHOX2B | 2 | 0 |
| PI4KAP2 | 2 | 0 |
| PPP3R1 | 2 | 0 |
| PRPF4 | 2 | 0 |
| PRPF40B | 2 | 0 |
| PRR19 | 2 | 0 |
| PTHLH | 2 | 0 |
| RALGDS | 2 | 0 |
| RBFOX1 | 2 | 0 |
| RBMS1 | 2 | 0 |
| RCOR3 | 2 | 0 |
| RNASEH2B | 2 | 0 |
| RTEL1 | 2 | 0 |
| SCAF11 | 2 | 0 |
| SELPLG | 2 | 0 |
| SH2D6 | 2 | 0 |
| SHOX2 | 2 | 0 |
| SIX3 | 2 | 0 |
| SLC35G1 | 2 | 0 |
| SMOC1 | 2 | 0 |
| SOX13 | 2 | 0 |
| SPATA16 | 2 | 0 |
| SPATA9 | 2 | 0 |
| SPECC1L | 2 | 0 |
| SRP14 | 2 | 0 |
| TDRKH | 2 | 0 |
| THAP10 | 2 | 0 |
| TTBK2 | 2 | 0 |
| UTP18 | 2 | 0 |
| VAX1 | 2 | 0 |
| VEGFB | 2 | 0 |
| WBP5 | 2 | 0 |
| ZCCHC10 | 2 | 0 |
| ZIC2 | 2 | 0 |
| ZNF232 | 2 | 0 |
| ZRSR2 | 2 | 0 |

Semi-quantification of signals on proteome-wide wet protein array was conducted as below: higher than positive control's signal strength: 6, higher than 1/2 positive control's signal strength but lower than positive control's signal strength: 5, higher than 1/4 but lower than 1/2 of positive control's signal strength: 4, higher than 1/8 but lower than 1/4 of positive control's signal strength: 3, higher than 1/16 but lower than 1/8 of positive control's signal strength: 2, higher than negative control's signal strength but lower than 1/16 of positive control's signal strength: 1.
